# Systematic review and meta-analysis of school-based obesity interventions in mainland China

**DOI:** 10.1371/journal.pone.0184704

**Published:** 2017-09-14

**Authors:** Lin Feng, Dong-Mei Wei, Shen-Ting Lin, Ralph Maddison, Cliona Ni Mhurchu, Yannan Jiang, Yang Gao, Hai-Jun Wang

**Affiliations:** 1 School of Public Health, Peking University, Beijing, China; 2 Institute of Child and Adolescent Health, Department of Child, Adolescent and Women’s Health, School of Public Health, Peking University, Beijing, China; 3 Health and Family Planning Bureau of Nanshan District, Shenzhen, China; 4 National Institute for Health Innovation, University of Auckland, Auckland, New Zealand; 5 Institute for Physical Activity and Nutrition, Deakin University, Geelong, Australia; 6 Department of Physical Education, Hong Kong Baptist University, Hong Kong, China; Old Dominion University, UNITED STATES

## Abstract

**Background:**

Numerous school-based interventions for childhood obesity have been emerging in mainland China in recent decades, but little is known about the effectiveness of such interventions. This study aims to assess the effectiveness of school-based interventions for childhood obesity conducted in mainland China.

**Methods:**

A systematic search was undertaken in eight databases to identify both randomized and non-randomized controlled trials from January 1990 to December 2015 examining the effectiveness of school-based obesity interventions. A random effects meta-analysis was conducted assessing the impact of included interventions on (body mass index) BMI. The quality of each included studies were assessed using Effective Public Health Practice Project Quality Assessment Tool. A *P* value <0.05 (two-sided) was considered statistically significant.

**Result:**

Of the seventy-six included studies, we found physical activity and health education were the two most common components of interventions. More treatment studies were effective compared with prevention studies (85.0% vs. 58.3%). Comprehensive interventions involving physical activity and health education appeared more effective than the physical activity only interventions in both obesity treatment and prevention studies. The meta-analyses showed comprehensive interventions involving physical activity and health education had larger effect on the change of BMI than physical activity only interventions (treatment studies: -1.80 kg/m^2^ (95% CI: -2.15,-1.44) vs. -0.91 kg/m^2^ (95% CI: -1.15,-0.67); prevention studies: -0.19 kg/m^2^ (95% CI: -0.27, -0.11) vs. +0.05 kg/m^2^ (95% CI: -0.04, +0.15)).

**Conclusions:**

Comprehensive school-based interventions may assist in tackling the rising prevalence of childhood obesity in mainland China.

## Introduction

Childhood obesity is a major threat to public health. Worldwide, the prevalence of childhood obesity has increased significantly over recent decades. The prevalence of overweight and obesity increased from 16.9% in 1980 to 23.8% in 2013 for boys and 16.2% in 1980 to 22.6% in 2013 for girls in high-income countries. In low- and middle-income countries, the overweight and obesity prevalence increased from 8.1% in 1980 to 12.9% in 2013 for boys and 8.4% in 1980 to 13.4% in 2013 for girls [1]. Over the past 25 years in China, there has been a substantial increase in the prevalence of childhood overweight and obesity. Results from the Chinese National Survey on Students’ Constitution and Health (CNSSCH), conducted every five years since 1985, revealed that 23.2% of boys and 12.7% of girls in urban areas were overweight or obese in 2010, compared to 1.3% of boys and 1.5% of girls in 1985. In rural areas, the prevalence of overweight and obesity for boys and girls were 0.5% and 1.6% respectively in 1985, increasing to 13.7% and 8.6% in 2010 [2]. The intervention studies for prevention or treatment of overweight among children and adolescents have been conducted in China and published in Chinese medical journals since the 1990s [3].

Childhood obesity is associated with many adverse health consequences, including asthma, sleep disorders, exercise intolerance, hypertension, chronic inflammation, and negative self-image in childhood [4], and some chronic non-communicable diseases later on in adulthood [5–8]. Intervening early to prevent and control childhood obesity is essential to reduce these negative consequences.

Schools are considered as an ideal environment for delivering obesity interventions to children because students spend most of their waking time at school (at least eight hours per day) [9] and have access to school requisite facilities such as classrooms for health education and facilities for exercise [10]. School policies also have an influence on students' behaviors associated with health [11].

The effectiveness of school-based intervention programs has been studied in several systematic reviews [12–21]. Some reviews have demonstrated that school-based interventions were effective for reducing BMI in children, with a change of BMI ranging from -0.04kg/m^2^ to -3.27kg/m^2^ [13–20]. However, few studies involved in those systematic reviews were conducted in low- and middle-income countries including China [22, 23], resulting in knowledge gaps regarding the effectiveness of interventions in such countries, where childhood obesity has been increasing at a faster rate than that in high income countries. Under this circumstance, the generalizability of these findings in Chinese children is limited.

Two systematic reviews addressed this knowledge gap. Gao et al. conducted a systematic review of community-based obesity interventions in China involving six school-based studies published before June 2006, of which five reported beneficial effects [24]. Li et al. conducted a systematic review of school-based interventions for preventing childhood overweight and obesity published from 1990 to 2006 in mainland China [3]. Of the twenty-two studies included in the review, thirteen were undertaken in primary or secondary schools and most of the studies (n = 10) reported beneficial effects in anthropometric outcomes, while six were in kindergartens and three in colleges. Neither of these two reviews conducted meta-analysis to quantitatively evaluate the effectiveness of the interventions. Since 2006, there has been an increase in the number of school-based interventions studies conducted in mainland China. To address this issue, we conducted a systematic review and meta-analysis of school-based childhood obesity interventions in mainland China published from January 1990 to December 2015.

## Methods

### Literature search

School-based intervention studies published from January 1990 to December 2015 were searched in three most commonly used Chinese databases (China National Knowledge Infrastructure (CNKI), Wanfang, Vip) and five international databases (PubMed, Embase, EBSCO, Springer, the Cochrane Library). Journal articles, Master’s and Doctoral theses were included in this review. The following terms were searched in all field to identify relevant studies: (1) participant-related (child, adolescent, student, boy, girl); (2) intervention-related (e.g. school, intervention, prevention, diet, exercise, physical activity, sedentary, behavior, education, policy, strategy, environment); (3) weight-related (e.g. obesity, overweight, weight, BMI, adiposity, fat); (4) country-related (China, Chinese). Full electronic search strategy was provided in S1 File.

Inclusion criteria were: (1) randomized or non-randomized controlled trials (RCTs or non-RCTs); (2) interventions based on primary and secondary schools in mainland China; (3) outcomes assessed by anthropometric measures including BMI, prevalence of overweight and obesity (weight status), waist or hip circumference, skin fold thickness, body fat percentage(BFP), percentage of over standard weight-for-height (i.e. (measured weight–standard weight-for-height) / standard weight-for-height × 100%, commonly used in Chinese studies); (4) the duration of interventions were at least 3 months; (5) full-text is available. Studies in Hong Kong, Macao and Taiwan were excluded from this study as we were unable to access their local publications. The school system and socioeconomic status are also different from that in mainland China. The studies were also excluded if: (1) interventions were designed specifically for treatment of obesity complications, such as type 2 diabetes, hypertension; (2) interventions involved drug treatments, any clinical operations or treatments of Chinese Traditional Medicine (such as acupuncture and moxibustion, ear point-pressing therapy); (3) the interventions or statistical methods were not described clearly.

Reference lists of both original and review articles were checked manually to identify any additional publications. Registered trials in ClinicalTrails.gov were also searched to identify unpublished studies, but none was found to meet our inclusion criteria.

Two reviewers (Dong-Mei Wei & Lin Feng) independently screened all titles and abstracts, and full-texts of potentially eligible studies were retrieved for further consideration. Discrepancies between the reviewers were discussed with a third reviewer (Hai-Jun Wang) and resolved with consensus.

### Quality assessment

Quality of included studies was assessed by two reviewers using the Effective Public Health Practice Project Quality Assessment Tool [25, 26]. Six components were evaluated, including selection bias, study design, confounders (variables that were associated with the intervention or exposure related to the study outcomes), data collection methods, blinding, and loss to follow up. Each component was rated as strong, moderate and weak quality. Overall quality of a study was rated “strong” if none of the components were rated weak, “moderate” if one component was rated weak, “weak” if two or more components were rated weak. Further classification was applied to those studies rated in weak quality, with weak 1 as having two components rated in weak quality and weak 2 as having three or more components rated in weak quality.

### Data extraction and analyses

The following data were extracted for analyses: author, location, year of publication, participant gender, sample size per group, participant age or school grade, targeted population, intervention duration, intervention components, main anthropometric outcomes, theoretical framework and randomization method.

An effective study was defined as statistically significant improvement in at least one anthropometric outcome (e.g. BMI, weight status, waist or hip circumference, skin fold thickness, body fat percentage, percentage of over standard weight-for-height) between the intervention and control groups (*P*<0.05) [24].

Meta-analysis was conducted for each intervention strategy when there were at least three studies with BMI as the outcome measure. The outcomes from meta-analyses were BMI changes from baseline in intervention groups compared with the changes in control groups. For each study, information on sample size, mean and standard deviation (SD) of change in BMI in both intervention and control groups were extracted for meta-analysis. When the SD was not reported in the study, the authors were first contacted and if no response, the SD of change in BMI was derived from standard error (SE) of the mean, 95% confidence interval (CI), or imputation using the formula described in the Cochrane Handbook (version 5.1.0) [27]. If the SD of BMI at baseline and post-intervention were known, the SD of change in BMI was calculated using the following formula.

$${SD}_{change}=\sqrt{{SD}_{baseline}^{2}+{SD}_{final}^{2}-(2\times Corr\times {SD}_{baseline}\times {SD}_{final})}$$

For calculating SD of change in the intervention and control group, 0.80 and 0.89 were used for *Corr*, respectively, which were based on a similar meta-analysis [15]. For studies with multiple arms, the effect of each intervention was compared with the control in meta-analysis. If the effects of intervention were reported at multiple time points, the data collected immediately after the intervention was used in meta-analysis.

All studies were grouped primarily according to the target population (treatment studies recruited only overweight and/or obese children vs. prevention studies recruited children without weight status restriction). The studies were further stratified by different strategies of intervention component(s) and the duration of intervention (long-term being more than 12 months vs. short-term being 3 to 12 months). Random effects models were used and the percentage of variation due to heterogeneity across the studies were assessed using the *I*^*2*^ statistic. Studies with substantial heterogeneity (i.e. *I*^*2*^ >50% or *P* value <0.1 form Chi-square teats assessing the heterogeneity of effect sizes across interventions) were removed [20]. Meta-analysis was performed using Review Manager 5.3. Heterogeneity test was performed using Stata 13.0. A *P* value <0.05 (two-sided) was considered statistically significant.

## Results

**Fig 1:**
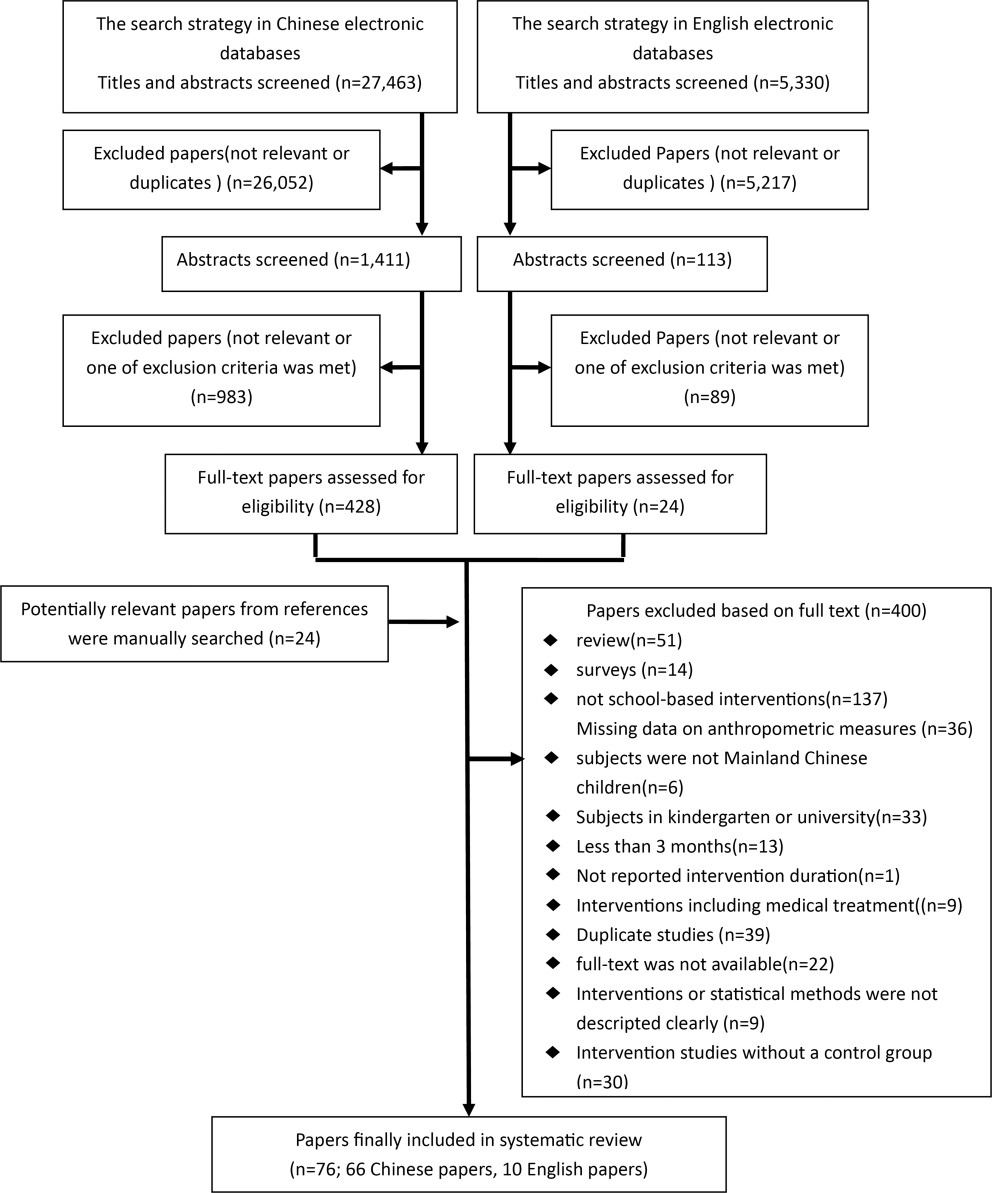
Flow chart of systematic review.

Our literature search initially identified 32,793 potential articles from the electronic databases. Flow chart was presented in Fig 1. Overall seventy-six studies met the inclusion criteria, including ten studies published in English [22, 23, 28–35] and sixty-six published in Chinese. There were fifty-eight journal articles [2, 22, 23, 28–83] and eighteen master theses [84–101]. No significant difference was found in quality between the two types of publications (Fisher’s Exact Test, *P* = 1.00).

### Characteristics of studies

The seventy-six studies included a total of 72620 Chinese students aged 6–19 years. Twenty studies (26.3%) were published before 2007, of which eleven studies [39, 46, 47, 49, 53, 55, 57, 59, 64, 67, 68] were included in Gao’s and Li’s previous reviews, but the other nine studies [36, 42, 48, 51, 56, 60, 65, 77, 84] were not (not due to quality or study design weakness). The remaining fifty-six studies have been published since 2007, including ten studies published in English, after Gao’s and Li’s previous reviews. Only one study included reported theoretical framework, as socio-ecological framework [75].

Table 1 summarized the characteristics of all studies, including study design, duration of intervention, school types and intervention strategies. Of the seventy-six included studies, forty were classified as treatment studies [29, 30, 34, 40–42, 44, 46, 48, 49, 56–63, 65–67, 69, 71–73, 76, 77, 79, 82, 84, 85, 89, 90, 93, 95, 97–101] targeting overweight and / or obese children and thirty-six were prevention studies for all children irrespective of baseline weight [22, 23, 28, 31–33, 35–39, 43, 45, 47, 50–55, 64, 68, 70, 74, 75, 78, 80, 81, 83, 86–88, 91, 92, 94, 96]. More than half of the studies were non-RCTs (60.5%). The majority (75%) of studies was short-term. Forty-eight studies (63.2%) were conducted in primary schools, sixteen studies in secondary schools (21.0%) and the remaining twelve (15.8%) in both settings.

**Table 1:** The summarized characteristics of the included studies.

	Overweight and obese children (n = 40)		All children		Total	
			(n = 36)		(n = 76)	
	N	%	N	%	N	%
**Study design**						
RCT	17	42.5	13	36.1	30	39.5
Non-RCT	23	57.5	23	63.9	46	60.5
**Duration of intervention**						
Short-term	32	80.0	25	69.4	57	75.0
Long-term	8	20.0	11	30.6	19	25.0
**School types**						
Primary	22	55.0	26	72.2	48	63.2
Secondary	12	30.0	4	11.1	16	21.0
Both	6	15.0	6	16.7	12	15.8
**Intervention strategies**						
Single component						
PA	15	37.5	7	19.4	22	29.0
HE	5	12.5	1	2.8	6	7.9
DI	0	0.0	1	2.8	1	1.3
Multiple components						
PA,HE (+others)	18	45.0	19	52.8	37	48.7
HE,WM	0	0.0	4	11.1	4	5.3
PA,DI	2	5.0	0	0.0	2	2.6
HE,PC	0	0.0	2	5.6	2	2.6
Others	0	0.0	2	5.6	2	2.6

RCT, randomized controlled trial; non-RCT, non-randomized controlled trial; PA, physical activity; HE, health education; DI, dietary improvement; WM, weight management; SP, school policies; PC, psychological counseling; PIS, physical infrastructure support; Others: including one study applied DI, SP; and one study applied HE, WM, SP, PIS. No study applied single component as WM, SP, PC nor PIS.

### Intervention strategies

Seven intervention components were identified, including (1) physical activity (PA, including interventions directly improving the intensities and duration of PA within and out of physical education classes in school setting), (2) health education (HE, including providing class lesson seminars, workshops, or relevant materials targeting students, teachers, canteen staffs or catering companies, as well as other forms of HE at school, such as broadcasts, posters, etc), (3) dietary improvement (DI, directly modifying school lunches), (4) weight management (WM, referring to monitoring of the weight-related indicators or keeping guidance records among overweight and obese children), (5) school policies (SP, obesity-related school policies), (6) psychological counseling (PC, a supplementary measure aimed at reducing the psychological problems caused by obesity and encouraging the obese children to perform exercise actively and healthy eating habits), (7) physical infrastructure support (PIS, upgrading sport facilities, expanding sport space or access to sport facilities at school).

As shown in Table 1, the majority of studies (n = 47, 61.8%) have used comprehensive interventions with multiple components. Comprehensive intervention strategy that involved PA and HE with or without other component was the most commonly applied in mainland China (45.0% in treatment studies, 52.8% in prevention studies). For single component interventions, PA only (28.9%) had the largest proportion, followed by HE only (7.9%) and DI (1.3%).

### Quality assessment

Overall most studies (n = 61, 80.3%) were rated as weak in quality, with 90.0% (n = 36) of treatment studies and 69.4% (n = 25) of prevention studies rated as weak, respectively. The remaining four treatment studies (10.0%) and eleven prevention studies (30.6%) were rated as moderate quality. No study was rated as strong quality. A trend was observed, however, that the proportion of studies in moderate quality was higher since 2007 (5.0% before 2007 vs. 25.0% since 2007, Fisher’s Exact Test, *P* = 0.10), indicating the overall quality of studies improved in the recent decade.

Table 2 showed the quality rating scores for treatment and prevention studies. In total, fifteen studies in moderate quality were rated as weak in blinding (n = 12, 80.0%) or selection bias (n = 3, 20.0%). Thirty studies were rated as weak 1 due to insufficient blinding (n = 30, 100%) and selection bias (n = 24, 80.0%). Thirty-one studies were rated weak 2 due to weakness in blinding (n = 31, 100%), selection bias (n = 30, 96.8%), confounders (n = 24, 77.4%) and withdraws (n = 16, 51.6%). The distribution of weakness in quality assessment components was similar between obesity treatment and prevention studies.

**Table 2:** Summary of the quality assessment components for 76 included studies.

		Number of studies	Weak in blinding		Weak in selection bias		Weak in confounders		Weak in withdraws		Weak in study design		Weak in data collection	
			n	%	n	%	n	%	n	%	n	%	n	%
**Treatment studies for overweight and obese children**	**Moderate**	4	2	50.0	2	50.0	0	0	0	0	0	0	0	0
	**Weak1**	19	19	100.0	17	89.5	1	5.3	1	5.3	0	0	0	0
	**Weak2**	17	17	100.0	16	94.1	13	76.5	9	52.9	0	0	0	0
	**Total**	40	38	95.0	35	87.5	14	35.0	10	25.0	0	0	0	0
**Prevention studies for all children**	**Moderate**	11	10	90.9	1	9.1	0	0	0	0	0	0	0	0
	**Weak1**	11	11	100.0	7	63.6	2	18.2	2	18.2	0	0	0	0
	**Weak2**	14	14	100.0	14	100.0	11	78.6	7	50.0	0	0	0	0
	**Total**	36	35	97.2	22	61.1	13	36.1	9	25.0	0	0	0	0
**Total**	**Moderate**	15	12	80.0	3	20.0	0	0	0	0	0	0	0	0
	**Weak1**	30	30	100.0	24	80.0	3	10.0	3	10.0	0	0	0	0
	**Weak2**	31	31	100.0	30	96.8	24	77.4	16	51.6	0	0	0	0
	**Total**	76	73	96.1	57	75.0	27	35.5	19	25.0	0	0	0	0

### Outcomes and effectiveness

#### Treatment studies

The detailed information on each treatment study was provided in S1 Table. Of the forty obesity treatment studies, seventeen (42.5%) were RCTs. Twenty studies (50.0%) have applied single component interventions, while twenty studies (50.0%) used comprehensive interventions with multiple components. There was no significant difference in quality between effective and non-effective obesity treatment studies (Chi-Square test, *P* = 0.374). Overall, thirty-four treatment studies (85.0%) targeting overweight and/or obese children were effective on at least one anthropometric outcome. The comprehensive interventions combining physical activity with health education had a trend to be more effective than that using physical activity only (88.9% vs. 80.0%). (see Table 3)

**Table 3:** Characteristics and effectiveness of 40 school-based obesity treatment studies targeting overweight and obese children.

	Years of publication	Enrolled participants	Quality (moderate/ weak 1/ weak 2)	Number of studies (short-term / long-term)	Number of RCTs (%)	Effective / All (%)							
						Short-term	Long-term	RCTs	non-RCTs	Moderate	Weak 1	Weak 2	Total
**Single component**													
**PA**	1997–2015	1047	0/9/6	15(13/2)	3(20.0)	11/13(84.6)	1/2(50.0)	3/3(100.0)	9/12(75.0)	0/0(0.0)	6/9(66.7)	6/6(100.0)	12/15(80.0)
**HE**	2005–2013	746	0/3/2	5(4/1)	3(60.0)	3/4(75.0)	1/1(100.0)	3/3(100.0)	1/2(50.0)	0/0(0.0)	3/3(100.0)	1/2(50.0)	4/5(80.0)
**Total**	1997–2015	1793	0/12/8	20(17/3)	6(30.0)	14/17(82.4)	2/3(66.7)	6/6(100.0)	10/14(71.4)	0/0(0.0)	9/12(75.0)	7/8(87.5)	16/20(80.0)
**multiple components**													
**PA+HE (+others)**	1997–2015	4636	3/6/9	18(13/5)	9(50.0)	12/13(92.3)	4/5(80.0)	8/9(88.9)	8/9(88.9)	2/3(66.7)	5/6(83.3)	9/9(100.0)	16/18(88.9)
**PA+DI**	2006–2015	114	1/1/0	2(2/0)	2(100.0)	2/2(100.0)	0/0(0.0)	2/2(100.0)	0/0(0.0)	1/1(100.0)	1/1(100.0)	0/0(0.0)	2/2(100.0)
**Total**	1997–2015	4750	4/7/9	20(15/5)	11(55.0)	14/15(93.3)	4/5(80.0)	10/11(90.9)	8/9(88.9)	3/4(75.0)	6/7(85.7)	9/9(100.0)	18/20(90.0)
**Overall**	1997–2015	6543	4/19/17	40(32/8)	17(42.5)	28/32(87.5)	6/8(75.0)	16/17(94.1)	18/23(78.3)	3/4(75.0)	15/19(78.9)	16/17(94.1)	34/40(85.0)

RCT, randomized controlled trial; non-RCT, non-randomized controlled trial; PA, physical activity; HE, health education. No treatment study applied other single component nor other types of multiple components.

Fifteen studies implemented physical activity only, with thirteen short-term studies and two long-term studies, three RCTs and twelve non-RCTs. Eleven of thirteen short-term studies (84.6%) and one of two long-term studies (50.0%) reported effective results. All three RCTs (100%) and nine of twelve non-RCTs (75.0%) reported effective results as well.

**Fig 2:**
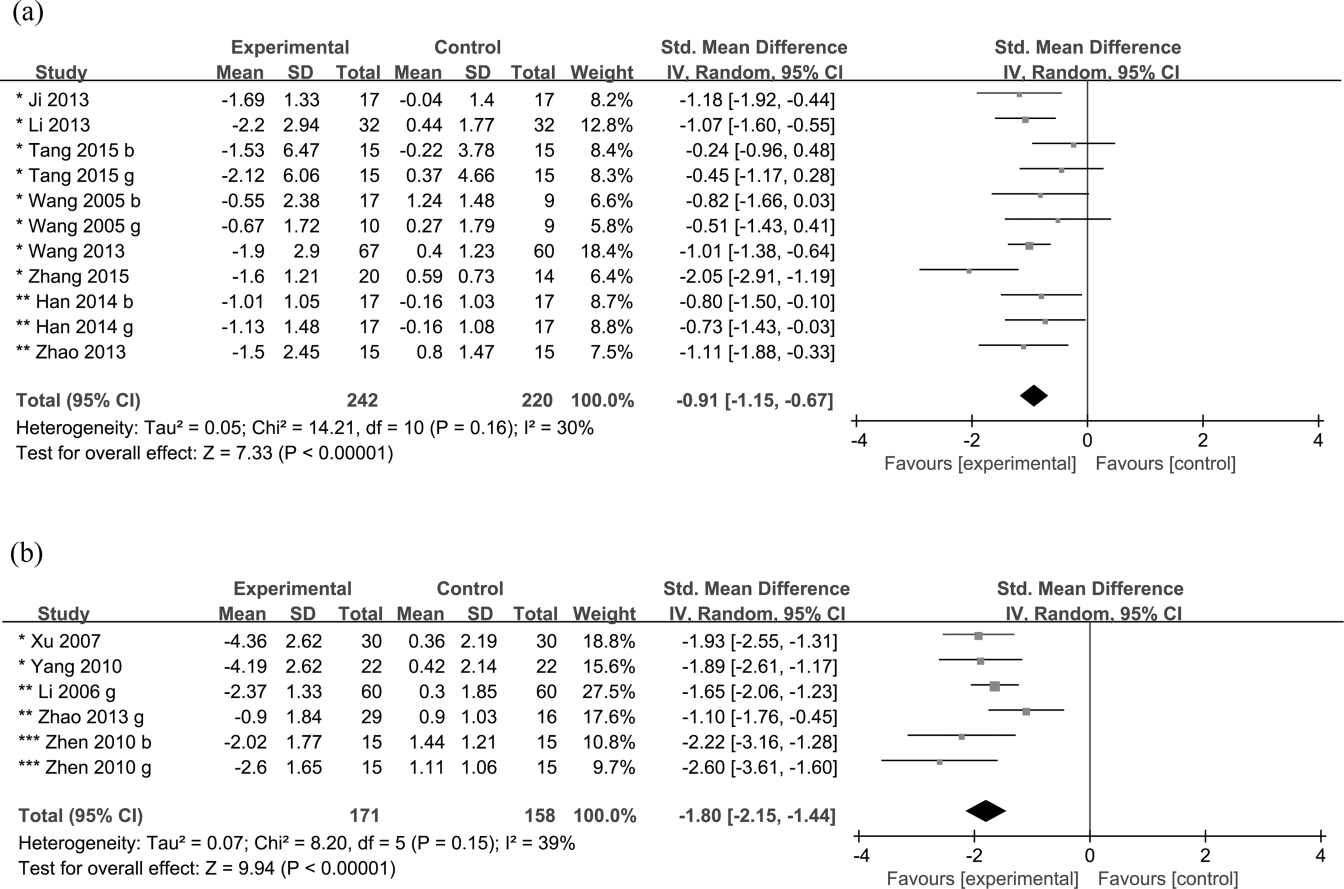
Meta-analyses on the change in body mass index for school-based obesity treatment studies. (a) Change in body mass index for studies using physical activity only. (b) Change in body mass index for studies using both physical activity and health education with or without other components. *, weak 2 quality; **, weak 1 quality; ***, moderate quality. b, boys only; g, girls only.

Eight short-term treatment studies with physical activity only [63, 79, 84, 85, 89, 93, 98, 100] were included in the meta-analysis and showed an overall mean difference of -0.91 kg/m^2^ (95% CI: -1.15,-0.67, *P*<0.001) in BMI in favor of the intervention group, without significant heterogeneity (*P* = 0.16, *I*^*2*^ = 30%) (Fig 2) nor publication bias (Egger’s test, *P* = 0.76, S1 Fig).

Five studies implemented health education only, four of which (80.0%) were assessed to be effective. Three of four short-term studies (75.0%) and the only long-term study showed that health education was effective. All of three RCTs were assessed to be effective.

Eighteen obesity treatment studies applied both physical activity and health education, of which sixteen studies (88.9%) were assessed to be effective. Twelve of thirteen short-term studies (92.3%) and four of five long-term studies (80.0%), eight of nine RCTs (88.9%) and eight of nine non-RCTs reported effective results.

Five short-term treatment studies involving both physical activity and health education [40, 60, 66, 95, 101] were included in the meta-analysis, which showed an overall mean difference of -1.80 kg/m^2^ (95% CI: -2.15,-1.44, *P*<0.001) in BMI in favor of the intervention group, without significant heterogeneity (*P* = 0.15, *I*^*2*^ = 39%) (Fig 2) nor publication bias (Egger’s test, *P* = 0.22, S1 Fig).

Two studies combined physical activity with dietary intervention [49, 62]. Both studies were short-term RCTs and were assessed to be effective.

#### Prevention studies

The detailed information on each prevention study was provided in S2 Table. Of the thirty-six prevention studies, thirteen (36.1%) were RCTs, and the other twenty-three studies (63.9%) were non-RCTs. Nine studies (25.0%) implemented single component interventions, while twenty-seven studies (75.0%) applied comprehensive intervention with multiple components. There was no significant difference in quality between effective and non-effective obesity prevention studies (Chi-Square test, *P* = 0.295). Overall, twenty-one of thirty-six prevention studies (58.3%) were effective on at least one anthropometric outcome. The interventions using multiple components involving physical activity and health education were more likely to be effective than single physical activity component interventions (78.9% vs. 28.6%). (see Table 4)

**Table 4:** Characteristics and effectiveness of 36 school-based obesity prevention studies for all children.

	Years of publication	Enrolled participants	Quality (moderate / weak 1/ weak 2)	Number of studies (short-term / long-term)	Number of RCTs (%)	Effective / All (%)							
						Short-term	Long-term	RCTs	non-RCTs	Moderate	Weak 1	Weak 2	Total
**Single component**													
**PA**	2007–2015	6981	3/0/4	7(7/0)	3(42.9)	2/7(28.6)	0/0(0.0)	1/3(33.3)	1/4(25.0)	1/3(33.3)	0/0(0.0)	1/4(25.0)	2/7(28.6)
**HE**	2011	1718	0/0/1	1(0/1)	0(0)	0/0(0.0)	0/1(0.0)	0/0(0.0)	0/1(0.0)	0/0(0.0)	0/0(0.0)	0/1(0.0)	0/1(0.0)
**DI**	2005	543	0/0/1	1(1/0)	0(0)	0/1(0)	0/0(0.0)	0/0(0.0)	0/1(0.0)	0/0(0.0)	0/0(0.0)	0/1(0.0)	0/1(0.0)
**Total**	2005–2015	9242	3/0/6	9(8/1)	3(33.3)	2/8(25.0)	0/1(0.0)	1/3(33.3)	1/6(16.7)	1/3(33.3)	0/0(0.0)	1/6(16.7)	2/9(22.2)
**Multiple components**													
**PA+HE (+others)**	2002–2015	41223	6/7/6	19(13/6)	6(31.6)	10/13(76.9)	5/6(83.3)	5/6(83.3)	10/13(76.9)	5/6(83.3)	6/7(85.7)	4/6(66.7)	15/19(78.9)
**Others**	2004–2015	15612	2/4/2	8(4/4)	4(50.0)	3/4(75.0)	1/4(25.0)	2/4(50.0)	2/4(50.0)	1/2(50.0)	2/4(50.0)	1/2(50.0)	4/8(50.0)
**Total**	2002–2015	56835	8/11/8	27(17/10)	10(37.0)	13/17(76.5)	6/10(60.0)	7/10(70.0)	12/17(70.6)	6/8(75.0)	8/11(72.7)	5/8(62.5)	19/27(70.4)
**Overall**	2002–2015	66077	11/11/14	36(25/11)	13(36.1)	15/25(60.0)	6/11(54.5)	8/13(61.5)	13/23(56.5)	7/11(63.6)	8/11(72.7)	6/14(42.9)	21/36(58.3)

RCT, randomized controlled trial; non-RCT, non-randomized controlled trial; PA, physical activity; HE, health education; DI, dietary improvement; Others combined health education with weight management, school policies, psychological counseling, or physical infrastructure support. No prevention study applied other single component nor other types of multiple components.

All seven studies implementing physical activity only were short-term studies, two of which (28.6%) were assessed to be effective. One of three (33.3%) RCTs and one of four non-RCTs (25.0%) reported effective results.

**Fig 3:**
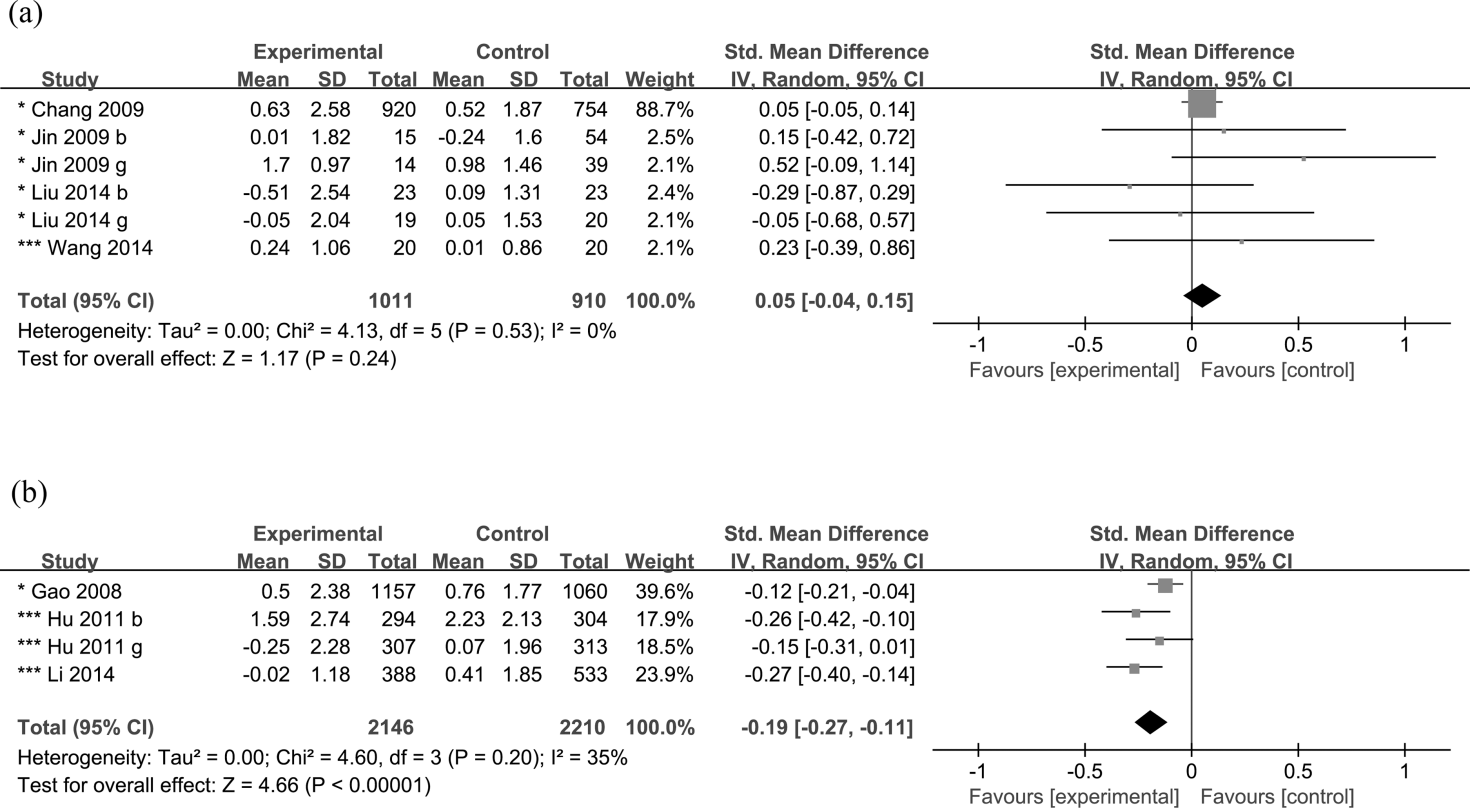
Meta-analyses on the change in body mass index for school-based obesity prevention studies. (a) Change in body mass index for studies using physical activity only. (b) Change in body mass index for studies using both physical activity and health education with or without other components. *, weak 2 quality; **, weak 1 quality; ***, moderate quality. b, boys only; g, girls only.

Four short-term prevention studies with physical activity only [45, 87, 88, 94] were included in the meta-analysis and showed no significant effect (mean difference: +0.05 kg/m^2^, 95% CI: -0.04, +0.15, *P* = 0.24), without significant heterogeneity (*P* = 0.53, *I*^*2*^ = 0%) (Fig 3) nor publication bias (Egger’s test, *P* = 0.70, S2 Fig).

There was one long-term non-RCT that implemented health education only [70] and reported non-effective results.

There was one short-term non-RCT that implemented dietary improvement only [36] and reported non-effective results.

Nineteen obesity preventions studies applied both physical activity and health education, of which fifteen studies (78.9%) were assessed to be effective. Ten of thirteen short-term studies (76.9%) and five of six long-term studies (83.3%), five of six RCT studies (83.3%) and ten of thirteen non-RCTs (76.9%) reported effective results.

Three short-term prevention studies involving both physical activity and health education [31, 38, 91] were included in meta-analysis, which showed an overall mean difference of -0.19 kg/m^2^ (95% CI: -0.27, -0.11, *P*<0.001) in BMI in favor of the intervention group, without significant heterogeneity (*P* = 0.20, I^2^ = 35%) (Fig 3) nor publication bias (Egger’s test, *P* = 0.29, S1 Fig).

Four of eight studies (50.0%) that implemented other intervention components were assessed to be effective. All four effective studies combined health education with weight management. The non-effective studies included combining dietary improvement with school policy (n = 1), combining health education with psychological counseling (n = 2), combination of health education, weight management, school policy and physical infrastructure support (n = 1).

### Sensitivity analysis

When only studies in moderate quality were included in meta-analyses, no treatment study in moderate quality applied PA only, and four treatment studies applied multiple components showed an overall mean difference of -1.26 kg/m^2^ (95% CI: -2.33, -0.20, *P* = 0.02, I^2^ = 91%). Two prevention studies applied PA only showed an overall mean difference of -0.09 kg/m^2^ (95% CI: -0.33, +0.16, *P* = 0.49, I^2^ = 26%). Four prevention studies applied multiple components showed an overall mean difference of -0.50 kg/m^2^ (95% CI: -1.14, +0.14, *P* = 0.13, I^2^ = 99%). Compared with our main results, the difference in change in BMI did not change materially but the efficiency lessened (S2 Fig).

## Discussion

### Main findings of this study

This study systematically reviewed seventy-six obesity interventions in mainland China. The prevention studies were less likely to be effective compared with the treatment studies. And the comprehensive interventions combining physical activity with health education (two most common components applied in school-based obesity interventions in mainland China) were more likely to be effective than the interventions with physical activity only. Although the quality of the included studies was rated as moderate (19.7%) or weak (80.3%), no significant association between quality and effectiveness was found.

### What Chinese studies have contributed to international reviews

There are several international reviews on school-based obesity intervention [12, 15, 18, 19, 102], however, only two obesity prevention studies conducted in primary schools of Beijing [22, 23] were included in those reviews. Both of them reported significant improvements in BMI, weight status or BFP. No school-based obesity treatment study in Chinese children was included in international reviews.

### What this study added

To our knowledge, this is the most comprehensive review of the school-based childhood obesity interventions in mainland China. All school-based interventions (both treatment and prevention studies) with a control group (both RCTs and non-RCTs) were searched in international and national databases. Quality and effectiveness of each study were assessed, and the effectiveness of different intervention components was evaluated. Meta-analysis was conducted for each intervention strategy when there were at least three studies with BMI as the outcome measure. As only two out of seventy-six Chinese studies were included in previous international reviews, this review provided valuable information for future obesity intervention studies in mainland China and international studies involving Chinese populations in other countries. The review also provides evidence for developing policies to prevent and control childhood obesity in mainland China and other countries with a similar setting.

### Intervention strategies

There were differences in school-based childhood obesity intervention strategies between mainland China and high-income countries. Physical activity and health education were two most common components applied in mainland China, while physical activity and dietary improvement were two key components in high-income countries [10, 20]. A recent systematic review identified sixty-one school-based childhood obesity interventions conducted in high-income countries, including forty studies (65.6%) combined physical activity with dietary improvement, eighteen applied physical activity only and the other three applied dietary improvement only, which reported moderate strength of evidence to support the effectiveness in diet interventions [20]. In our review, only twenty included studies (26.3%) involving dietary improvement, of which only one prevention study applied single dietary improvement [36]. The evidence of dietary improvement is limited in Chinese school-based obesity interventions, which should be implemented and evaluated in the future.

### Effectiveness of obesity treatment studies and prevention studies

Treatment studies were more likely to be effective than prevention studies. The effect sizes of treatment studies on BMI were larger than prevention studies, which was also found in international mate-analysis [15]. As overweight and obese children have larger baseline BMI and suffer more adverse health consequences than normal-weight children [4–8], they were more motivated to control weight and more likely to adherent to obesity interventions. While, prevention interventions are more meaningful to protect children from obese adverse consequences and reverse the tide of the childhood obesity epidemic. To improve the effectiveness of prevention studies in mainland China, improving adherence of children and developing more effective intervention components studies, such as parental supervision and supports [10, 19], should be conducted in the future.

### Effectiveness of intervention component(s)

“School Sports Prescription” seemed to be an effective physical activity intervention widely applied in treatment studies, which generally organized forty to sixty minutes physical activities guided by specific prospectus after class in school days, focused on moderate intensity aerobic exercise complementary with strength and fitness training [40, 58, 60, 89, 95, 99–101]. Overweight and obese students were required to take part in these programs at least two to five times a week. “Happy 10 Program” might be another effective physical activity intervention. This program consisted of two daily ten minutes physical activity sessions conducted in the break between classes, which initiated by Chinese government with various physical recreational games, was widely applied as part of comprehensive intervention strategies in prevention studies and seemed to be effective [23, 33, 43, 91].

Health education intervention components were analogous between treatment studies and prevention studies. Lifestyle improvement knowledge covered both healthy diet and proper exercise by intensive classes combined with pamphlets, posters, blackboard newspapers inclined to be effective, in which parents and teachers in charge were generally involved to enhance compliance [31, 35, 44, 61, 67, 77, 83, 91].

According to our results, comprehensive interventions involving physical activity component appeared to be more effective in both treatment and prevention studies, which were supported by international reviews [14, 16, 17, 19, 103]. Therefore, comprehensive interventions with multiple components such as “Happy 10 Program”, “School Sports Prescription” combined with lifestyle improvement health educations are suggested for preventing and controlling childhood obesity in the school setting.

### Limitation

There were some limitations in the study. Firstly, the majority of included studies (80.3%) were rated as weak in quality, mainly due to insufficient blinding and selection bias. Lack of blinding and subjective selection of participants or communities without randomization could introduce both operational and statistical bias. However, as the components of school-based obesity interventions were related to behavior change, material and environmental supports, it was difficult to blind participants and assessors to the treatment allocation. In addition, some Chinese researchers did not provide sufficient information on blinding, randomization method, recruitment strategies and participant withdraws. These publications were therefore rated as weak in relevant components according to the quality assessment tool.

Secondly, the effects of other intervention components, such as dietary improvement with or without physical activity, could not be tested due to insufficient studies. The effects of those intervention components should be evaluated when more studies become available.

Thirdly, the intervention effects estimated from our meta-analyses may not be comparable to international studies, because of the differences in study design, intervention components and contents delivered to school children. Multi-regional controlled trials are suggested to further explore regional or ethnic differences in effectiveness of school-based obesity interventions, which will be important for developing global policy to tackle the epidemic of childhood obesity.

Fourthly, we used the change in BMI to conduct meta-analysis for evaluating the effectiveness of interventions as it was the most commonly reported in the included studies, although BMI z-score would be more preferable in school children. We also didn’t use prevalence of obesity to perform the meta-analysis because the included studies used different BMI cut-points to define it. Both BMI-Z score and prevalence of obesity should be considered in future studies to evaluate the effectiveness of school-based obesity interventions.

## Conclusion

There are a large number of school-based childhood obesity intervention studies which have been conducted in mainland China. More treatment studies seemed to be effective compared with prevention studies. Comprehensive interventions involving physical activity and health education showed larger effect on BMI than physical activity only. Comprehensive school-based interventions may assist in tackling the rising prevalence of childhood obesity in mainland China. The results of meta-analyses provided some evidences for the obesity intervention researchers but should be considered in caution, as the included studies have apparent limitations. More high quality researches should been conducted in the future to confirm the effectiveness of school-based obesity interventions.

## Supporting information

S1 File Full electronic search strategy. (DOCX)

S2 File PRISMA 2009 checklist. (DOC)

S1 Table Characteristics of 40 treatment studies. This file demonstrated author, target population, study design, sample size, types of intervention, intervention duration, study location, main results, study quality and effectiveness of each 40 treatment studies included.
(XLSX)

S2 Table Characteristics of 36 prevention studies. This file demonstrated author, target population, study design, sample size, types of intervention, intervention duration, study location, main results, study quality and effectiveness of each 36 prevention studies included.
(XLSX)

S1 Fig Funnel plots of studies examining the body mass index (BMI) of school-based obesity treatment / prevention studies. (a) Change in BMI for treatment studies using physical activity only. (b) Change in BMI for treatment studies using both physical activity and health education with or without other components. (c) Change in BMI for prevention studies using physical activity only. (d) Change in BMI for prevention studies using both physical activity and health education with or without other components.
(TIF)

S2 Fig Forest plots of moderate quality studies examining the body mass index (BMI) of school-based obesity treatment / prevention studies. (a) Change in BMI for treatment studies using physical activity only (no moderate quality study was found). (b) Change in BMI for treatment studies using both physical activity and health education with or without other components. (c) Change in BMI for prevention studies using physical activity only. (d) Change in BMI for prevention studies using both physical activity and health education with or without other components.
(TIF)

S1 Dataset 76 included studies. (ZIP)

## References

[1] Ng M, Fleming T, Robinson M, Thomson B, Graetz N, Margono C, et al Global, regional, and national prevalence of overweight and obesity in children and adults during 1980–2013: a systematic analysis for the Global Burden of Disease Study 2013. Lancet. 2014;384(9945):766–81. doi: 10.1016/S0140-6736(14)60460-8 24880830 10.1016/S0140-6736(14)60460-8 PMC4624264

[2] J M, Ci-he C, Hai-jun W, Bin D, Yi S, Pei-jin HU, et al The trend analysis of overweight and obesity In Chinese students during 1985–2010. Chin J Prev Med. 2012;46(9):776–80. 23157879

[3] Li M, Li S, Baur LA, Huxley RR. A systematic review of school-based intervention studies for the prevention or reduction of excess weight among Chinese children and adolescents. Obes Rev. 2008;9(6):548–59. doi: 10.1111/j.1467-789X.2008.00495.x 18503504 10.1111/j.1467-789X.2008.00495.x

[4] Ebbeling CB, Pawlak DB, Ludwig DS. Childhood obesity: public-health crisis, common sense cure. Lancet. 2002;360(9331):473–82. doi: 10.1016/S0140-6736(02)09678-2 12241736 10.1016/S0140-6736(02)09678-2

[5] Cheng TO. Cardiovascular health, risks and diseases in contemporary China. Int J Cardiol. 2012;154(2):233–42. 22351957 10.1016/j.ijcard.2011.12.023

[6] Hall ME, Do CJM, Da SAA, Juncos LA, Wang Z, Hall JE. Obesity, hypertension, and chronic kidney disease. Int J Nephrol Renovasc Dis. 2014;7:75–88. doi: 10.2147/IJNRD.S39739 24600241 10.2147/IJNRD.S39739 PMC3933708

[7] Jung UJ, Choi MS. Obesity and its metabolic complications: the role of adipokines and the relationship between obesity, inflammation, insulin resistance, dyslipidemia and nonalcoholic fatty liver disease. Int J Mol Sci. 2014;15(4):6184–223. doi: 10.3390/ijms15046184 24733068 10.3390/ijms15046184 PMC4013623

[8] Whitaker RC, Wright JA, Pepe MS, Seidel KD, Dietz WH. Predicting obesity in young adulthood from childhood and parental obesity. N Engl J Med. 1997;337(13):869–73. doi: 10.1056/NEJM199709253371301 9302300 10.1056/NEJM199709253371301

[9] Li M, Dibley MJ, Yan H. School environment factors were associated with BMI among adolescents in Xi'an City, China. BMC Public Health. 2011;11:792 doi: 10.1186/1471-2458-11-792 21988882 10.1186/1471-2458-11-792 PMC3198712

[10] Story M. School-based approaches for preventing and treating obesity. Int J Obes Relat Metab Disord. 1999;23 Suppl 2:S43–51. 10.1038/sj.ijo.0800859 10340805

[11] Uk CfPH, Uk NCCf. Obesity: The Prevention, Identification, Assessment and Management of Overweight and Obesity in Adults and Children. London: National Institute for Health and Clinical Excellence (UK); 2006. 22497033

[12] Gonzalez-Suarez C, Worley A, Grimmer-Somers K, Dones V. School-based interventions on childhood obesity: a meta-analysis. Am J Prev Med. 2009;37(5):418–27. doi: 10.1016/j.amepre.2009.07.012 19840696 10.1016/j.amepre.2009.07.012

[13] Harris KC, Kuramoto LK, Schulzer M, Retallack JE. Effect of school-based physical activity interventions on body mass index in children: a meta-analysis. CMAJ. 2009;180(7):719–26. doi: 10.1503/cmaj.080966 19332753 10.1503/cmaj.080966 PMC2659836

[14] Katz DL, O'Connell M, Njike VY, Yeh MC, Nawaz H. Strategies for the prevention and control of obesity in the school setting: systematic review and meta-analysis. Int J Obes (Lond). 2008;32(12):1780–9. 19079319 10.1038/ijo.2008.158

[15] Lavelle HV, Mackay DF, Pell JP. Systematic review and meta-analysis of school-based interventions to reduce body mass index. J Public Health (Oxf). 2012;34(3):360–9. 22267291 10.1093/pubmed/fdr116

[16] Martin A, Saunders DH, Shenkin SD, Sproule J. Lifestyle intervention for improving school achievement in overweight or obese children and adolescents. Cochrane Database Syst Rev. 2014;(3):CD009728 doi: 10.1002/14651858.CD009728.pub2 24627300 10.1002/14651858.CD009728.pub2

[17] Oude LH, Baur L, Jansen H, Shrewsbury VA, O'Malley C, Stolk RP, et al Interventions for treating obesity in children. Cochrane Database Syst Rev. 2009;(1):CD001872 doi: 10.1002/14651858.CD001872.pub2 19160202 10.1002/14651858.CD001872.pub2

[18] Silveira JA, Taddei JA, Guerra PH, Nobre MR. The effect of participation in school-based nutrition education interventions on body mass index: a meta-analysis of randomized controlled community trials. Prev Med. 2013;56(3–4):237–43. doi: 10.1016/j.ypmed.2013.01.011 23370048 10.1016/j.ypmed.2013.01.011

[19] Sobol-Goldberg S, Rabinowitz J, Gross R. School-based obesity prevention programs: a meta-analysis of randomized controlled trials. Obesity (Silver Spring). 2013;21(12):2422–8. 23794226 10.1002/oby.20515

[20] Wang Y, Cai L, Wu Y, Wilson RF, Weston C, Fawole O, et al What childhood obesity prevention programmes work? A systematic review and meta-analysis. Obes Rev. 2015;16(7):547–65. doi: 10.1111/obr.12277 25893796 10.1111/obr.12277 PMC4561621

[21] Waters E, de Silva-Sanigorski A, Hall BJ, Brown T, Campbell KJ, Gao Y, et al Interventions for preventing obesity in children. Cochrane Database Syst Rev. 2011;(12):CD001871 doi: 10.1002/14651858.CD001871.pub3 22161367 10.1002/14651858.CD001871.pub3

[22] Jiang J, Xia X, Greiner T, Wu G, Lian G, Rosenqvist U. The effects of a 3-year obesity intervention in schoolchildren in Beijing. Child Care Health Dev. 2007;33(5):641–6. doi: 10.1111/j.1365-2214.2007.00738.x 17725789 10.1111/j.1365-2214.2007.00738.x

[23] Li YP, Hu XQ, Schouten EG, Liu AL, Du SM, Li LZ, et al Report on childhood obesity in China (8): effects and sustainability of physical activity intervention on body composition of Chinese youth. Biomed Environ Sci. 2010;23(3):180–7. doi: 10.1016/S0895-3988(10)60050-5 20708496 10.1016/S0895-3988(10)60050-5

[24] Gao Y, Griffiths S, Chan EY. Community-based interventions to reduce overweight and obesity in China: a systematic review of the Chinese and English literature. J Public Health (Oxf). 2008;30(4):436–48. 17989084 10.1093/pubmed/fdm057

[25] Project EPHP. Quality assessment tool for quantitative studies. 2010.

[26] Armijo-Olivo S, Stiles CR, Hagen NA, Biondo PD, Cummings GG. Assessment of study quality for systematic reviews: a comparison of the Cochrane Collaboration Risk of Bias Tool and the Effective Public Health Practice Project Quality Assessment Tool: methodological research. J Eval Clin Pract. 2012;18(1):12–8. doi: 10.1111/j.1365-2753.2010.01516.x 20698919 10.1111/j.1365-2753.2010.01516.x

[27] Green JPHaS, editor. Cochrane Handbook for Systematic Reviews of Interventions. 5.1.0 ed. The Cochrane Collaboration 2011.

[28] Cao ZJ, Wang SM, Chen Y. A randomized trial of multiple interventions for childhood obesity in China. Am J Prev Med. 2015;48(5):552–60. doi: 10.1016/j.amepre.2014.12.014 25891054 10.1016/j.amepre.2014.12.014

[29] Gong L, Yuan F, Teng J, Li X, Zheng S, Lin L, et al Weight loss, inflammatory markers, and improvements of iron status in overweight and obese children. J Pediatr. 2014;164(4):795–800.e2. doi: 10.1016/j.jpeds.2013.12.004 24518166 10.1016/j.jpeds.2013.12.004

[30] Guo H, Zeng X, Zhuang Q, Zheng Y, Chen S. Intervention of childhood and adolescents obesity in Shantou city. Obes Res Clin Pract. 2015;9(4):357–64. doi: 10.1016/j.orcp.2014.11.006 25596004 10.1016/j.orcp.2014.11.006

[31] Li XH, Lin S, Guo H, Huang Y, Wu L, Zhang Z, et al Effectiveness of a school-based physical activity intervention on obesity in school children: a nonrandomized controlled trial. BMC Public Health. 2014;14:1282 doi: 10.1186/1471-2458-14-1282 25510313 10.1186/1471-2458-14-1282 PMC4320634

[32] Liu AL, Hu XQ, Ma GS, Cui ZH, Pan YP, Chang SY, et al Report on childhood obesity in China (6) evaluation of a classroom-based physical activity promotion program. Biomed Environ Sci. 2007;20(1):19–23. 17458137

[33] Meng L, Xu H, Liu A, van Raaij J, Bemelmans W, Hu X, et al The costs and cost-effectiveness of a school-based comprehensive intervention study on childhood obesity in China. PLoS One. 2013;8(10):e77971 doi: 10.1371/journal.pone.0077971 24205050 10.1371/journal.pone.0077971 PMC3800134

[34] Wang JJ, Lau WC, Wang HJ, Ma J. Evaluation of a comprehensive intervention with a behavioural modification strategy for childhood obesity prevention: a nonrandomized cluster controlled trial. BMC Public Health. 2015;15:1206 doi: 10.1186/s12889-015-2535-2 26635229 10.1186/s12889-015-2535-2 PMC4668691

[35] Xu F, Ware RS, Leslie E, Tse LA, Wang Z, Li J, et al Effectiveness of a Randomized Controlled Lifestyle Intervention to Prevent Obesity among Chinese Primary School Students: CLICK-Obesity Study. PLoS One. 2015;10(10):e0141421 doi: 10.1371/journal.pone.0141421 26510135 10.1371/journal.pone.0141421 PMC4625022

[36] Sun G, Hu Y, Yang J, Wang S, Luo H, Xu H, et al. Study on school lunch intervention among primary and secondary school students in Nanjing City. The sixth Public Nutrition Branch of China Nutrition Association, Dalian,China 2005; Dalian2005.

[37] Jiang H, Yang P, Fan He, Huang H, Si M. Study of Comprehensive Intervention on Obesity Youths in Hongkou Distrizt, Shanghai. Proceedings ofInternational Forum for Public Health,Shanghai 2007; Shanghai2007.

[38] Gao A, Pan Y, Shi X, Cui C. Effect of "Happy 10 minutes" for preventing childhood obesity. Chinese Journal of School Health. 2008;29(11):978–9.

[39] Tian B, Zhang J, Lu S, Qian L, Zhang W, Zhang J. Impact evaluation on obesity control among primary school students in 4 cities in China. Chinese Journal of School Health. 2006;(10):869–71.

[40] Yang C. Effect on sports prescription education of 16 weeks for children obesity with 9–10 years old. Zhejiang Sport Science. 2014;36(1):101–5,28.

[41] Dajiang L, Yongqiang Z, Jie P, Zhigao Z. Personalized exercises intervention for obese children in a school fitness center. Chinese Journal of School Health. 2011;(11):1338–40.

[42] Daying S, Huihua X, Huaiyu L, Guoquan H. Interventions on obesity and malnutrition among primary school students in Luwan district. Chinese Journal of School Doctor. 2006;20(4):361–3.

[43] Fan Y, Wenxian W, Hongwei G, Jian Z, Xuan L, Yanrong L. Effects of Comprehensive Intervention on Overweight or Obese Students of Primary Schools in a District of Shanghai. J Environ Occup Med. 2013;(05):333–7.

[44] Fuyan W. Analysis of effects and change of unhealthy lifestyle in obese pupils of health education. China Practical Preventive Medicine. 2009;16(6):1809–10.

[45] Gai C, Hao L, Yi Y, Jie M, Wenjuan W, Guohong J. Effect of "Take 10!" intervention on the related indexes of obese pupils. Chinese Journal of Prevention and Control of Chronic Non-Communicable Diseases. 2009;(05):505–7.

[46] Fang H, Chen A-q. Exploration of interventions for pubertal simple obesity in Bao'an District of Shenzhen. Chinese Journal of Woman and Child Health. 2006;17(4):255–8.

[47] Li H, Jiang L, Chang X, Wang W. A Mid-term Effectiveness of Project Develop Health Promotion School Taking Obesity Control as Entry Point in Shenzhen City. Chinese Journal of Health Education. 2004;(06):19–22.

[48] Tan H, Wang Z, An A, Ou Y, Wang W. Evaluation of the effects of physical exercise interventions to simple obese children. Practical Preventive Medicine. 2002;(01):78–9.

[49] Dai J, Jiang Z, Zhang B. Exercise and nutrition therapy for simple obesity in children. Chinese Journal of Clinical Rehabilitation. 2006;10(32):20–2.

[50] Duan J, Sun H, Lu O, Song H, Guo X, Zhang H. Impact evaluation on early prevention on adulthood diseases in some schools in Beijing. Chinese Journal of School Health. 2008;(05):404–6.

[51] Chen J. Effectiveness of Project on Taking Obesity Control as Entry Point of Health Promoting School in Xiamen, Fujian. Strait J Prev Med. 2006;(05):8–10.

[52] Liu J, Jin M, Yu Me. Research of community-based obesity intervention for pupils in Laiyang city, Shandong Province. Journal of Hygiene Research. 2012;(05):866–7.

[53] Shi J, Liu X, Tian X, Li Y. An analysis of intervention on obese students in primary schools in Beijing. Chinese Journal of Health Education. 2004;(09):14–7.

[54] Zheng J, Zou S, Du W, Wang J, Tao Y. Investigation on nutrition intervention effects of pupils in Pudong New Area. Chin J Child Health Care. 2010;(03):206–9.

[55] Jiang J, Xia X, Wu G, Tan Z, Song X, Wang L, et al School-based Intervention for Obese Children. Chinese Journal of Child Health Care. 2002;(06):364–7.

[56] Jingxiong J, Xiulan X, Jinghong H, Xianfen C. Comprehensive interventions for obese children. Chinese Mental Health Journal. 1997;(04):51–3+46+66.

[57] Jun-Qing B. A study of comprehensive behavior treatment for obesity in puberty. Chinese Journal of School Health. 1997;(04):305–6.

[58] Laiqiang L, Aming L, Xudong F. Observation effects of movement therapy for fat children. Journal of Harbin Institute of Physical Education. 2007;(01):132–4.

[59] Lan S, Jingjian Y, Hong X, Yuezhen T, Shuixian S, Feihong L. Appraisal of intervention effects on obesity students of primary and secondary schools in Minhang district. Chinese Journal of Public Health. 2005;(03):14–5.

[60] Li M. Anti-obesity effect of comprehensive diet and sports in girl students with simple obesity or overweight. Chinese journal of Clinical Rehablitation. 2006;(32):44–6.

[61] Li W, Zhu-xin W, Fang-fang W. The Effects of Healthy Living Styles on Growth and Development Among Overweight and Obese children. China Practical Preventive Medicine. 2007;28(9):796–7.

[62] Liangmei X, Xiangtian L, Wenjun W, Chao Z, Chengkai S, Weijun F, et al Effects of balanced diet and exercise on simple obesity among middle school students. Chinese Journal of School Health. 2015;(01):51–3.

[63] Li-hua L. Study on developing exercise prescription and improve the physical fitness of obese pupils. China School Physical Education. 2013;(S2):129–30.

[64] Peisheng T, Jinzhi H, Jida X, Xiangming F, Xiaoqin Q, Liangcai Z, et al Evaluation of living environmental control and psychological interventions in obese students. Chinese Journal of School Health. 2006;(11):983–4+6.

[65] Ping L, Gang L. Effects of comprehensive behavior intervention in curing the pure obesity of the juvenile students. Chinese Journal of Behavioral Medical Science. 1999;(02):43–5.

[66] Sheng-Li X. Effect of aerobic exercise comprehensive intervention measures on leptin and blood fat composition in obese children. Maternal & Child Health Care Of China. 2007;(21):2935–7.

[67] Shuiping H, Jihong S, Haixia J, Lang Z, Hu C. Effects of Community Group Intervention on Childhood Simple Obesity. Chinese Journal of School Health. 2005;(11):12–3.

[68] Suqin L, Qinghua Z, Yiling Y, Yuqin L. School-based intervention for obese children. Chinese Journal of School Doctor. 2005;(02):115–7.

[69] Weichan C, Miao F, Haibin H, Shaofen L, Aijun L. Effectiveness of nursing intervention on overweight and obese adolescents lose weight. International Journal of Nursing. 2010;29(10):1491–3.

[70] Weiming Z, Wuping L, Xiujuan T, Jingfang Y, Herong L, Wei J. Effect of nutritional education on dietary behaviors and nutritional state of students. Maternal & Child Health Care of China. 2011;(12):1780–2.

[71] Wen L, Cuiqing C, Xiaoqian Z, Xue J, Zhimin C, Lan X, et al Effect of School-Based Supervised Exercise Intervention on Body Mass Index and Glucolipid Metabolism In Chinese Obese Adolescents. Chinese Journal of Sports Medicine. 2008;(03):329–33.

[72] Xiangjiang R, Jiapei Z, Shiwei Z, Jun M. A study on effects of exercise for simple obesity in young students. Chinese Journal of Rehabilitation Medicine. 2007;(08):702–5.

[73] Xiao-Lin Y. Effect of Combination of Medicine and Sport on Serum Leptin and Lipid Composition of Obese Children. J Appl Clin Pediatr. 2010;(18):1447–8.

[74] Xia-xia J, Chen-jing P, Hong C. Effect of comprehensive intervention on overweight of middle school students. Chinese Journal of School Health. 2010;31(3):265–6,9.

[75] Xinyue C, Tianjiao C, Jun M. Effect of obesity intervention with socio-ecological model on anthropometric measurements of children and adolescents. Journal of Peking University (Health Science). 2015;(03):400–5. 26080866

[76] Xiujuan W, Jian W, Hua D, Yuechun G, Xunbao Z, Jihong S, et al Childhood obesity intervention study in Xuzhou. Modern Preventive Medicine. 2010;(12):2225–6+30.

[77] Xiu-Lian Z. Effect of behavior modification in controlling children's obesity. Chinese Journal of Clinical Rehabilitation. 2005;(11):166–7.

[78] Xue H, Yuanfen C, Peimin L, Hua F, Yang L. A study on nutrition intervention model for primary school pupils in Yangpu District, Shanghai. Shanghai Journal of Preventive Medicine. 2008;20(4):193–5.

[79] Yanping T, Xuxia G. Effect of large class break activity intervention on the body shape of overweight and obese children. Sports Forum. 2015;(11):71–3.

[80] Youlan C, Yukai D, Xiaojian Y, Jihui G, Hong Y, Yueying X. Analysis on influencing factors of childhood obesity at one primary school in Xiamen City. Chinese Journal of Health Education. 2010;(10):756–9+66.

[81] Zhao-Mei L. The factors of childhood overweight and obesity and effects of related interventions. Maternal & Child Health Care Of China. 2015;(09):1393–4.

[82] Zhaoyuan H. Experimental study of the influence of sports homework for obese middle school students on BMI. Sport. 2011;(16):64–5+46.

[83] Zhiqin W, Ying F. Study on comprehensive community intervention for childhood obesity prevention. Chinese Journal of Women and Children Health. 2013;(06):56+8.

[84] Beibei W. The influence of exercise on body composition, body fat distribution and physical fitness in obese children [Master]: Beijing Sport University; 2005.

[85] Chengqiang J. The study of self-consciousness, physical self-esteem and exercise intervention of primary school students in Shanghai [Master]: Shanghai Normal University; 2013.

[86] Dali X. The effects of Spots and health education Joint intervention on children's physical health in mountain areas [Master]: Shan-Xi Normal University; 2013.

[87] Dan W. Experimental study of the martial arts setting-up exercise on the effects of physical fitness of rural junior high school students [Master]: Jishou University; 2014.

[88] Hainan J. Influence of endurance exercise with duration of three-month on aerobic work capacity in the students of grade four [Master]: Shanghai Normal University; 2009.

[89] Jingyan H. The coordination of the late adolescent obese students and the effect of exercise intervention [Master]: Soochow University; 2014.

[90] Kun Z. Knowledge, attitude,behavior interventions of nutrition on obesity primary school students [Master]: Shanghai Normal University; 2013.

[91] Lili H. Effect of dietary-based prevention and control technology on morphological and blood biochemical indicator relatived obesity [Master]: Shandong University; 2011.

[92] Ruifang X. A study for health promotion strategies and the status of knowledge-attitude-practice on obesity in Qingpu District [Master]: Fudan University; 2009.

[93] Wang Z. Study on the effect and possible mechanism of exercise training on obesity in obese children [Master]: Zhejiang University; 2013.

[94] Wenjing L. Study on monitoring the physical fitness of middle school students and delivering intervention to them in Nanjing City [Master]: Nanjing Normal University; 2014.

[95] Xiaoqian Z. Study of sports intervention on obese middle school students in Beijing [Master]: Beijing Sport University; 2013.

[96] Xihai Z. The experimental research of the impact of 24 style Tai Chi on junior's physical health–taking the Wuhan Nanhu middle school for example [Master]: Central China Normal University; 2015.

[97] Xingmin G. Research in motion based integrated interventions affect cognitive health in obese children [Master]: East China Normal University; 2014.

[98] Yanjun Z. Study on the effect of extracurricular physical exercise intervention on physical fitness of simple obese pupils [Master]: Yangzhou University; 2015.

[99] Yanying G. The research about the influence from the exercise intervention to body shape and self-awareness of the children with simple obesity [Master]: Shanghai University of Sport; 2011.

[100] Zhao P. Experimental study on obese pupils by exercise interventio [Master]: Capital University of Physical Education and Sports; 2013.

[101] Zhen L. The effect of exercise-based comprehensive long-term intervention on overweight and obese children [Master]: Capital University of Physical Education and Sport; 2010.

[102] Mei H, Xiong Y, Xie S, Guo S, Li Y, Guo B, et al The impact of long-term school-based physical activity interventions on body mass index of primary school children—a meta-analysis of randomized controlled trials. BMC Public Health. 2016;16:205 doi: 10.1186/s12889-016-2829-z 26931236 10.1186/s12889-016-2829-z PMC4774105

[103] Brown T, Summerbell C. Systematic review of school-based interventions that focus on changing dietary intake and physical activity levels to prevent childhood obesity: an update to the obesity guidance produced by the National Institute for Health and Clinical Excellence. Obes Rev. 2009;10(1):110–41. doi: 10.1111/j.1467-789X.2008.00515.x 18673306 10.1111/j.1467-789X.2008.00515.x

