# Supplementary material for: Systematic review and meta-analysis of school-based obesity interventions in mainland China
Source: PLoS One. 2017 Sep 14;12(9):e0184704. doi: 10.1371/journal.pone.0184704 (PMC5598996; doi:10.1371/journal.pone.0184704)
Supplement: S1 File — (DOCX) [file pone.0184704.s001.docx]

The full electronic search strategy for PubMed was showed below:

Filters：Publication Date from January 1990 to December 2015; Humans; English.

1. child
2. children
3. adolescent
4. adolescence
5. boy
6. boys
7. girl
8. girls
9. youth
10. youths
11. young
12. student
13. students
14. #1 OR #2 OR #3 OR #4 OR #5 OR #6 OR #7 OR #8 OR #9 OR #10 OR #11 OR #12 OR #13
15. intervention*
16. prevent*
17. treat*
18. health promotion
19. behavior change
20. health education
21. diet
22. dietary
23. nutrition*
24. physical exercise
25. physical activit*
26. physical education
27. environment
28. policy
29. political
30. sedentary
31. static
32. #15 OR #16 OR #17 OR #18 OR #19 OR #20 OR #21 OR #22 OR #23 OR #24 OR #25 #26 OR #27 OR #28 OR #29 OR #30 OR #31
33. obesity
34. obese
35. fat
36. fatness
37. overweight
38. weight
39. body mass index
40. BMI
41. adiposity
42. metabolic syndrome
43. MS
44. Mets
45. #33 OR #34 OR #35 OR # 36 OR #37 OR #38 OR #39 OR #40 #41 OR #42 OR #43 OR #44
46. China
47. Chinese
48. #46 OR #47
49. #14 AND #32 AND #45 AND #48 AND school
