# Supplementary figures and images for: Systematic review and meta-analysis of school-based obesity interventions in mainland China
Source: PLoS One. 2017 Sep 14;12(9):e0184704. doi: 10.1371/journal.pone.0184704 (PMC5598996; doi:10.1371/journal.pone.0184704)

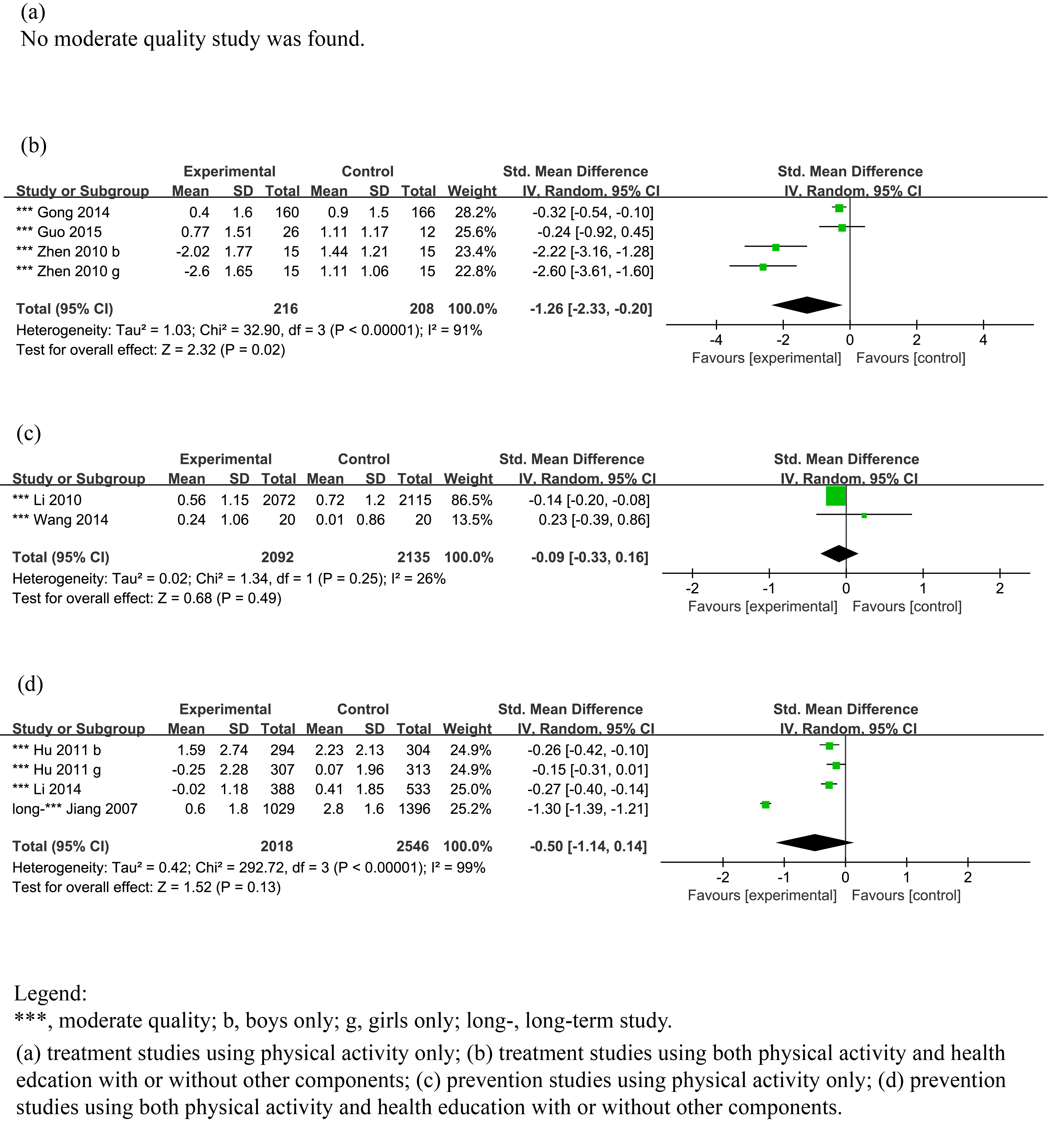

Supplement: S1 Fig — (a) Change in BMI for treatment studies using physical activity only. (b) Change in BMI for treatment studies using both physical activity and health education with or without other components. (c) Change in BMI for prevention studies using physical activity only. (d) Change in BMI for prevention studies using both physical activity and health education with or without other components. (TIF) [file pone.0184704.s005.tif]

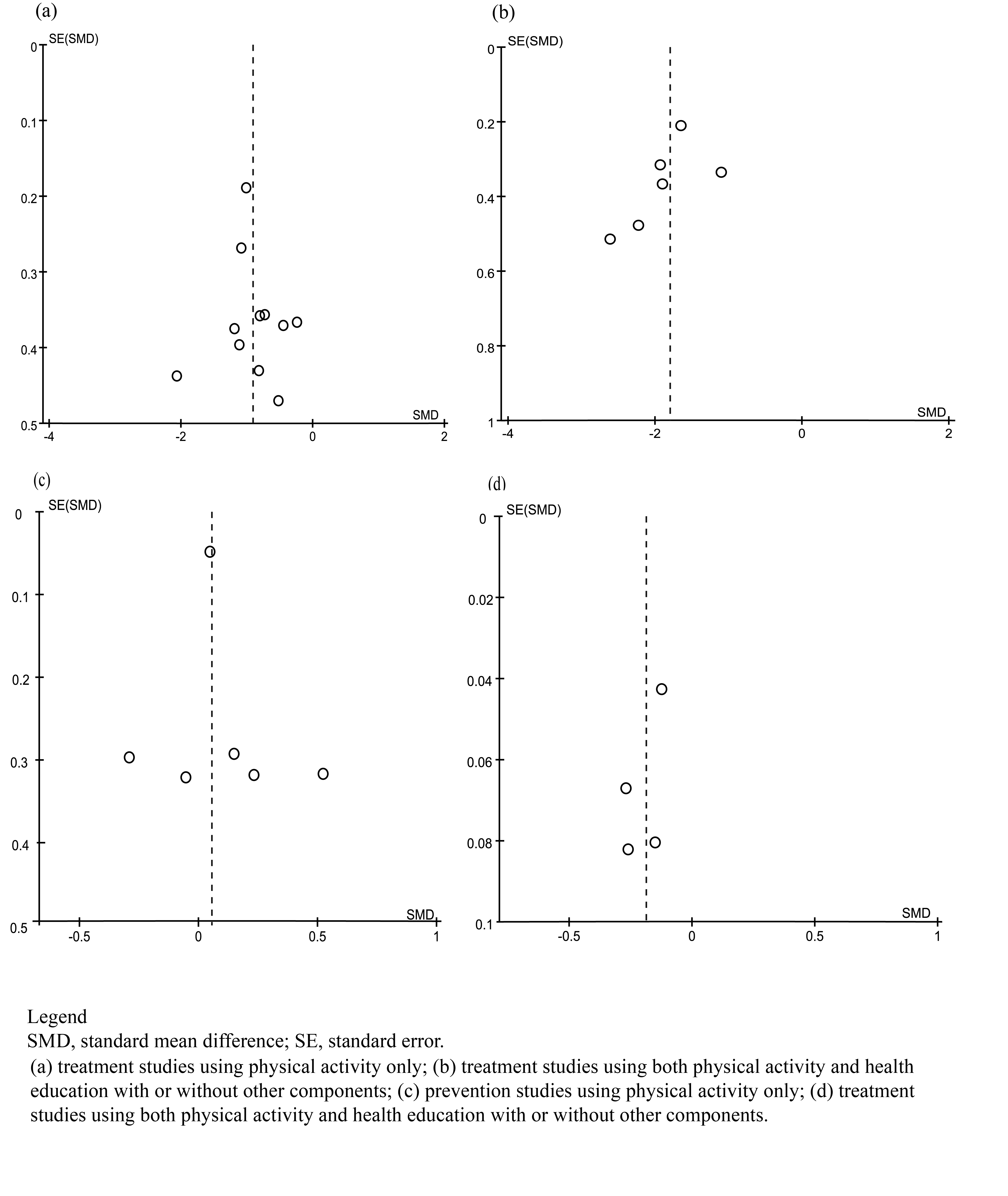

Supplement: S2 Fig — (a) Change in BMI for treatment studies using physical activity only (no moderate quality study was found). (b) Change in BMI for treatment studies using both physical activity and health education with or without other components. (c) Change in BMI for prevention studies using physical activity only. (d) Change in BMI for prevention studies using both physical activity and health education with or without other components. (TIF) [file pone.0184704.s006.tif]
