# Supplementary material for: Systematic review and meta-analysis of school-based obesity interventions in mainland China
Source: PLoS One. 2017 Sep 14;12(9):e0184704. doi: 10.1371/journal.pone.0184704 (PMC5598996; doi:10.1371/journal.pone.0184704)
Supplement: S1 Dataset — (ZIP) [file pone.0184704.s007.zip › S1_dataset/76库/22.pdf]

doi:10.1111/j.1365-2214.2007.00738.x

The effects of a 3-year obesity intervention in schoolchildren in Beijing

J. Jiang,*§ X. Xia,† T. Greiner,‡[1] G. Wu,† G. Lian* and U. Rosenqvist§

*National Center for Women's and Children's Health, China CDC
†Capital Institute of Paediatrics, Beijing, China
‡Department of Women's and Children's Health, Uppsala University, Uppsala, Sweden, and
§Department of Public Health and Caring Science, Uppsala University, Uppsala, Sweden

Accepted for publication 10 November 2006

Abstract

Background Childhood obesity has become a health problem in urban areas in China. Intervention to reduce childhood obesity should be of high priority. School-based intervention programmes are needed to deal with the growing prevalence of childhood obesity in China.

Methods Five primary schools were selected randomly for this study in the Beijing urban area in China; two were allocated to the intervention group and three to the control group. A total of 2425 children (1029 children in intervention schools and 1396 children in control schools) took part in the study for 3 years. In the intervention group, children and their parents were involved in a programme of nutrition education and physical activity. Control school students followed their usual health and physical education curriculum with no extra intervention.

Results After the 3-year intervention, the prevalence of overweight and obesity were significantly lower in the intervention schools than in the control schools (overweight: 9.8% vs. 14.4%, $P < 0.01$; obesity: 7.9% vs. 13.3%, $P < 0.01$). The prevalence of overweight and obesity decreased by 26.3% and 32.5% in intervention schools respectively after intervention. The prevalence of overweight and obesity increased in control schools. There was also significant difference in body mass index between intervention and control schools (18.2 ± 2.6 vs. 20.3 ± 3.4, $P < 0.01$) after intervention. More non-obese children became obese in the control schools (7.0%) than in the intervention schools (2.4%) at end line ($P < 0.01$). Among the children who were obese at baseline, 49.2% remained obese at end line in intervention schools while 61.9% remained obese in control schools ($P < 0.01$).

Conclusions Our study showed that an intervention programme could be feasible in schools in Beijing, China. The prevalence of overweight and obesity was reduced in schoolchildren in Beijing through an intervention focused on nutrition education and physical activity. Overweight and obesity children as well as normal weight children and their parents should be involved in such an intervention programme.

Keywords
childhood obesity, China, intervention, nutritional education, prevalence, school

Correspondence:
Dr Jingxiong Jiang, Department of Child Health Care, National Center for Women's and Children's Health, Building A, no. 13 Dong Tu Cheng Road, Chaoyang District, Beijing 100013, China
E-mail: jiangjingxiong@chinawch.org.cn

[1]Present address: PATH, Washington DC, USA.

Introduction

Childhood obesity appears to be increasing throughout the world (James *et al*. 2001; Rudolf *et al*. 2001; Koletzko *et al*. 2002) and obesity is now becoming a public health problem not only in developed, but also in many developing countries (Ramachandran *et al*. 2002). Obesity in childhood and adolescence is predictive of weight status in adulthood (Whitaker *et al*. 1997)

© 2007 The Authors
Journal compilation © 2007 Blackwell Publishing Ltd

and is a known risk factor for the development of chronic diseases (Geiss *et al.* 2001; Sorof *et al.* 2002; Kiess *et al.* 2003). Once established, obesity is difficult to treat. Therefore, early interventions should be prioritized.

A wide range of behavioural, social and environmental factors have been suggested as potential drivers of the current childhood obesity epidemic. These include changes in the consumption of foods and an increase in sedentary time. Nutritional and activity interventions are likely to be more successful when factors that influence choice related to healthy dietary behaviours and lifestyle are well understood. Many intervention programmes have demonstrated that promotion of physical activity and healthy diet can be effective (Frenn *et al.* 2003a; Steckler *et al.* 2003; Summerbell *et al.* 2005).

China has joined the world epidemic of obesity. An alarming increase in the prevalence of obesity has been documented, not only in adults (Cheng 2001) but also in children (Luo & Hu 2002). By the end of 2000, the obesity rate of male students in Beijing, China reached 15%, approaching that of developed countries (Luo 2002).

Since the late 1990s, the Beijing Education Bureau has identified diseases which should be prevented in the school; obesity is among them. Schools represent potentially important places for intervention programmes. [illegible] large numbers of students and have stru[illegible] support intervention programmes. The [illegible] was to measure the effects of a primary [illegible] tion programme on the prevalence of ob[illegible]

两个区，每区2干2对；2个干预拒绝（每区各1）原文表述有问题，1个对照拒绝。

Methods

Study design and data collection

There are six urban districts in Beijing, China. Two districts were selected randomly as sample areas. Four primary schools were selected randomly as sample schools from a list of all primary schools with over 400 students in each district originally. The four schools were randomly assigned into two intervention schools and two control schools in each district. Among the four selected intervention schools, two schools (from two districts) agreed to join the programme. One school refused to join the programme among the four selected control schools. The five schools which agreed to join the programme were the final sample. They were two intervention schools (from two districts) and three control schools (from two districts). Information describing the intervention programme was sent to all the students and parents in all participating schools. Signed consent forms were received from 89% of potential participants in intervention schools and 94% in control schools. The obesity/non-obesity ratio, gender and age range were similar between the children who participated and those who did not participate the study both in intervention and control schools. This study was approved by the Ethics Committee of the Beijing Health Bureau before it was implemented.

Intervention and control schools were located in different communities but had similar school size, physical education and socio-economic status based on parents' education level, occupation and family income. The students in intervention schools were exposed to the intervention programme for 3 years. Control school students followed their usual health and physical education curriculum with no extra intervention programme.

The intervention programme focused on reducing the prevalence of obesity in the schoolchildren both by reducing obesity among those already obese and by preventing new cases. The main component of the intervention programme was nutrition education aimed at both the children and their parents. A nutritional lecture was given by the researcher in this study team to all the parents once per semester at the routine parents' meetings organized by the school. The contents of these lectures included health consequences of childhood obesity, the food pyramid, and elements of a healthy lifestyle (increasing consumption of vegetables and fruits and physical activity; avoiding overeating, eating out in restaurants frequently and eating in the absence of hunger; reducing consumption of Western fast foods, television viewing and computer games). Educational materials on childhood obesity prevention were distributed to all the parents. They were designed by this study team and included the weight and height references by age and gender, obesity references, and methods of obesity prevention. The attendance rate at these meeting was above 90% every time.

There was no content of obesity prevention in the school curriculum. A text on childhood obesity prevention was designed by researchers in the study team for classroom lessons in the intervention schools. Its contents included risk factors for obesity, healthy dietary habits and lifestyle, and ways of preventing obesity. There were 10 themes in the text in total. Each theme was addressed in one lesson per fortnight. Teachers were trained in its use.

An extra meeting for parents with overweight and obese children was arranged by intervention schools and the study team once per semester. A 'traffic light' food item list was given to the parents to help children decrease their energy intake and consume a balanced diet: 'red light' foods were those high in fat or calories; 'green light' foods were low in fat and calories; and 'yellow light' foods were intermediate. We urged the parents to

© 2007 The Authors

help their children eat less red light foods and more green light foods. We also encouraged the parents to buy more green light foods instead of red light foods. The parents were informed about daily calorie requirements, based on the Chinese Recommended Daily Allowance. We also gave Chinese food composition tables to the parents, so they could calculate the calorie intake of their child every day and compare with the calorie requirements. In order to avoid the feeling of hunger and limit the calorie intake, some dietary behaviours were suggested to the family, including eating slowly, having soup before meals, eating green light foods first, brushing teeth immediately after meals and having meals without staple foods for supper. Parents were also informed of methods to achieve a healthy lifestyle of all family members. For parents who encouraged their overweight or obese children to overeat, we sought to analyse the reasons for this behaviour and to modify it. Parents were taught to limit their child's sedentary time at home and advised to take a walk with their child after supper. We also encouraged these parents to weigh their child every week. The attendance rate of the meeting was above 74% among the potential participants every time.

A meeting was also arranged for all the overweight and obese children once every semester. More than 86% of the children who consented to join the intervention programme attended the meeting every time. The styles of the meeting consisted of lectures, group discussions, question-and-answer sessions and experience sharing. The children were advised to avoid having access to high-fat foods and fast food, and to increase their access to fruits and vegetables. The children were asked whether their parents encouraged them to eat more. We also urged the children to decrease sedentary time, e.g. watching TV and to go for a walk after supper instead.

The intervention aimed to increase physical activity as well. In the afternoon from Monday to Thursday, all the overweight and obese children, along with the children who failed to pass routine school physical education tests were asked to run for 20 min after class. The physical education teachers monitored this activity. The attendance rate was 50–70%, most of time among the children who consented to join the intervention programme.

Measurements taken for data analysis included heights taken without shoes (to the nearest 0.1 cm) and weights taken in light clothes (to the nearest 0.1 kg). After baseline measurement, all children in intervention schools were classified as normal weight, overweight and obesity. Weight and height were measured for all students in the intervention schools twice each semester. This higher frequency of measurement was a part of the intervention programme. The children's weight status (normal weight, overweight, obesity) was defined after each measurement and parents were informed of the results. Throughout the 3-year period, normal weight children who became overweight were involved in an additional intervention programme and the overweight or obese children whose weight recovered to normal then skipped these additional sessions. Thus, the students and parents attending the meetings for overweight and obese children changed somewhat each semester. The weight and height of control children were measured once every year and their parents were also informed of the results. The same types of scales for weight and height were used in all five schools. Weights and heights were measured by the same trained doctors in the same month each semester for both intervention and control children. The data analysed and reported in this study came only from the baseline and end line measurements. Overweight and obesity were defined in this paper according to sex- and age-specific body mass index (BMI) cut-points proposed by the International Obesity Task Force (Cole *et al*. 2000).

Statistical analysis

All data analyses were preformed using SPSS for Windows (version 12.0, SPSS Inc., Chicago, IL, USA). Significance level was set to a random error of $\alpha = 0.05$. Differences of weight, height, BMI and prevalence of overweight and obesity between intervention and control schools were tested by independent *t*-test and chi-square. In regressions to predict obesity at end line, the baseline predictors of obesity (obesity, age, BMI, school and family socio-economic status) were controlled. Boys and girls were estimated using separate regressions.

Results

Sample characteristics

Data were collected from the students in grades 1–4 in intervention and control schools at baseline who consented to take part in the study and from this same cohort when they completed grades 3–6 at end line 3 years later. The study samples included 2489 students (1056 students in intervention schools and 1433 students in control schools) at baseline. Sixty-four students lacked end line data because of either school transfer (24 students in intervention schools and 31 students in control schools respectively) or school absence (three students in intervention schools and six students in control schools). Among the students without end line data, there were 27 students in intervention schools (24 non-obese children and three obese chil-

© 2007 The Authors

非初始人数

Table 1. Characteristics of sample children at baseline and end line in intervention and control schools in Beijing*

	Intervention			Control		
	Total *n* = 1029	Boys *n* = 531	Girls *n* = 498	Total *n* = 1396	Boys *n* = 715	Girls *n* = 681
Baseline						
Age (years)	8.4 ± 1.4	8.4 ± 1.3	8.3 ± 1.4	8.2 ± 1.5	8.3 ± 1.5	8.2 ± 1.4
Weight (kg)	28.4 ± 6.5	28.9 ± 6.8	27.3 ± 5.3	28.8 ± 6.8	29.4 ± 7.1	27.9 ± 6.0
Height (cm)	129.4 ± 5.6	129.2 ± 5.4	129.5 ± 5.8	130.0 ± 5.0	130.2 ± 4.9	129.8 ± 5.2
BMI	17.6 ± 2.9	18.1 ± 3.3	17.0 ± 2.5	17.5 ± 2.7	17.9 ± 2.8	17.2 ± 2.4
Overweight (%)	13.3	15.0	11.4†	12.6	14.4	10.7†
Obesity (%)	11.7	14.5	8.6‡	11.5	14.1	8.7‡
End line						
Age (years)	11.2 ± 1.3	11.2 ± 1.2	11.2 ± 1.3	10.9 ± 1.4	11.0 ± 1.3	10.9 ± 1.4
Weight (kg)	40.3 ± 8.5	40.4 ± 9.2	40.0 ± 6.8	43.9 ± 8.6	44.8 ± 8.9	42.7 ± 8.1
Height (cm)	147.7 ± 6.1	147.5 ± 6.4	147.9 ± 5.3	146.8 ± 7.0	146.8 ± 7.1	146.9 ± 5.8
BMI	18.2 ± 2.6	18.4 ± 2.9	18.1 ± 2.4	20.3 ± 3.4§	20.5 ± 3.5§	19.9 ± 3.0§

*Data presented are mean ± SD except where percentage is indicated in the table.
†Comparing boys with girls, $P < 0.05$.
‡Comparing boys with girls, $P < 0.01$.
§Comparing control group with intervention group, $P < 0.01$.
BMI, body mass index.

dren) and 37 in the control schools (31 non-obese children and six obese children). Their data were eliminated and data from the other 2425 students (1029 in intervention and 1396 in control) were included in this paper. At baseline, there were no significant differences in age, gender ratios and prevalence of overweight and obesity between the intervention and control schools (Table 1).

Change in prevalence of obesity with intervention

At follow-up, the prevalence of overweight and obesity were significantly lower in intervention schools than in control schools (Table 2). The prevalence of overweight decreased 26.3% in intervention schools and increased 14.3% in control schools. The prevalence of obesity decreased 32.5% in intervention schools and increased 15.7% in control schools.

More non-obese children became obese in the control schools than in the intervention schools (7.0% vs. 2.4%, OR 0.338, 95% CI 0.214–0.538, $P = 0.002$). Among the children who were obese at baseline, 49.2% remained obese at end line in intervention schools while 61.9% remained obese in control schools. Remission of obesity was significantly higher in intervention students than in control students (50.8% vs. 38.1%, OR 1.668, 95% CI 1.030–2.610, $P = 0.001$) (Fig. 1).

Safety

Children in the intervention and control schools had a similar linear growth velocity. The mean 3-year height increase was 18.2 cm and 16.9 cm in intervention and control schools respectively. No unintended side effects were observed during the intervention.

Table 2 Prevalence of overweight and obesity at end line in intervention and control schools in Beijing

	Intervention (%)	Control (%)	Adjusted OR*	95% CI	*P*-value§
Overweight					
Total	9.8	14.4	0.614	0.465–0.788	0.001
Boys	11.9	18.5	0.564	0.391–0.756	0.001
Girls	7.6†	10.1‡	0.670	0.461–1.068	0.085
Obesity					
Total	7.9	13.3	0.556	0.413–0.738	0.001
Boys	9.4	16.0	0.502	0.383–0.785	0.001
Girls	6.2	10.4‡	0.568	0.389–0.901	0.036

*Adjusted odds ratio controlled for baseline obesity, age, BMI, school and family socio-economic status. Regression estimates were calculated using the generalized estimating equation method to account for clustering of observations within schools.
†Comparing boys with girls, $P < 0.05$.
‡Comparing boys with girls, $P < 0.01$.
§Significance level, intervention vs. control.
BMI, body mass index.

Discussion

The high prevalence of childhood obesity and recent evidence linking childhood obesity to increased morbidity and mortality (Dietz 1998; Goran 1998) have led to a consensus that childhood obesity interventions should be of high priority (Wilson

© 2007 The Authors

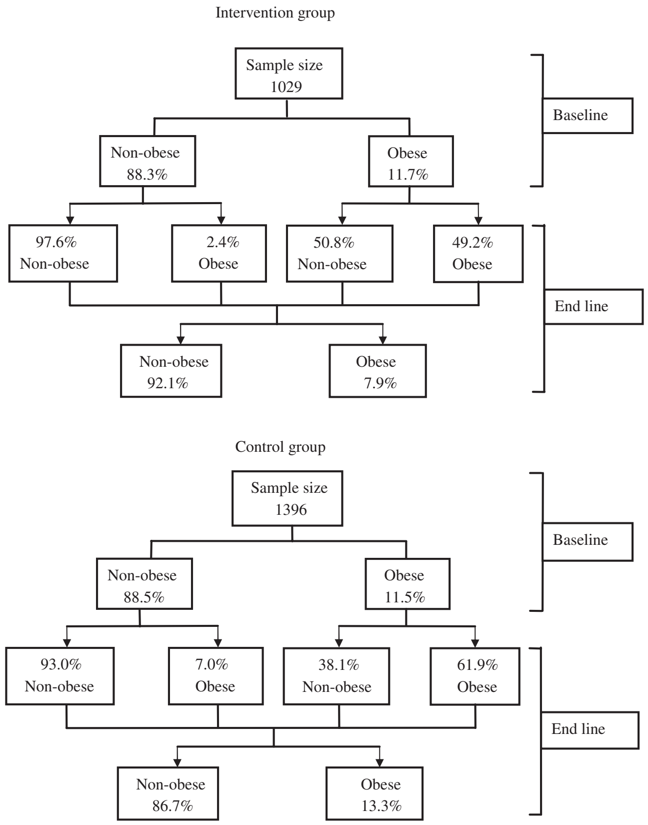

Figure 1. Flow graph of obesity changes from baseline to end line in children in intervention and control schools in Beijing.

et al. 2003; Ells *et al.* 2005). There are only a few studies of obesity interventions focused on the prevalence of obesity in children. Most population-based interventions showed some improvement in health knowledge and health-related behaviours, including consumption of a low-fat diet, more vegetable and fruit consumption, less sedentary time, and more physical activity (Braet *et al.* 1997; Epstein *et al.* 1998; Frenn *et al.* 2003b; Harrell *et al.* 2005). Prevalence of obesity as an indicator of public health problems should be given attention.

Implementation of this school-based intervention was highly successful. The prevalence of overweight and obesity were no different between intervention and control schools at baseline but over the 3-year intervention period decreased significantly among children in intervention schools and increased in control schools, leading to significant differences between the two groups at end line. The incidence of obesity and remissions also indicated statistically significant intervention effects. This suggests that the intervention impacted not only on obese children but also on those at risk of becoming obese.

The prevalence of overweight and obesity were significantly higher in boys than in girls, both in intervention and control children and both at baseline and end line. In Chinese tradition, people treat boys and girls differently. Obese boys are considered to be strong and obese girls are thought to be fat. Obese boys are much more accepted by parents than obese girls. Society also places great importance on weight and body shape in females than in males. Girls want to be like the slim ladies shown in advertisements. These may be among the reasons that the prevalence of obesity was higher in boys than in girls. In another recent study of children, the prevalence of overweight was 27.7% in boys and 14.1% in girls (Iwata *et al.* 2003). More published scientific evidence is still needed to support this hypothesis.

Both treatment and prevention are needed to help overweight and obese children improve their BMI as they grow in height as well as to prevent new cases of obesity. In our intervention programme, a school-based approach was used for all the students as well as special interventions directed at overweight and obese students. The average BMI was no different between intervention and control children at baseline. It was significantly lower in intervention children comparing with control children at end line. It also showed the positive result in prevention of obesity in one aspect. In our experience, regular height and weight measurement was itself a useful intervention. Overweight and obese children can be screened and parents were informed about their child's nutrition status over time. Most urban Chinese families have only one child. Parents tend to overfeed their children if they do not think that their children are overweight. 'Don't encourage your child to eat more when the child feels full' was a major message in the programme, not a particularly difficult behaviour for the parents to implement.

No adverse effects were observed among the children with intervention. The children in intervention schools had a similar linear (height) growth to that of control children.

School-based intervention programmes can be implemented in a school setting, offering regular contact with children and parents. The eagerness of the teachers, students and parents in the intervention schools to participate in the programme was a great asset. We believe that this intervention was more effective than only targeting obese children individually would have been.

This study had several potential limitations. The intervention programme did not cover many schools. Dietary intake, physical activity and obesity-related behaviours were not measured. For the severely obese children, their obesity degree (BMI) did not decrease significantly during the intervention. Even after adjusting for clustering statistically, there is an inevitable potential for bias in allocation at the school level and as a result of non-blinded assessment of outcome.

© 2007 The Authors

Our intervention produced a significant decrease in the prevalence of overweight and obesity, suggesting it may be possible to control or at least reduce this health problem in schools in China. From this study, we feel that interventions directed at all children should be encouraged to reduce the prevalence of obesity. Future interventions should consider social and family support as well as better school education for children.

Acknowledgements

We thank all the parents, children, teachers and co-workers who took part in the study.

References

Braet, C., Van Winckel, M. & Van Leeuwen, K. (1997) Follow-up results of different treatment programs for obese children. *Acta. Paediatrics*, **86**, 397–402.

Cheng, T. O. (2001) An obesity epidemic in modern China. *American Journal of Cardiology*, **88**, 721–722.

Cole, T. J., Bellizzi, M. C., Flegal, K. M. & Dietz, W. H. (2000) Establishing a standard definition for child overweight and obesity worldwide: international survey. *British Medical Journal*, **320**, 1240–1243.

Dietz, W. H. (1998) Health consequences of obesity in youth: childhood predictors of adult disease. *Pediatrics*, **101**, 518–525.

Ells, L. J., Campbell, K., Lidstone, J., Lang, R. & Summerbell, C. (2005) Prevention of childhood obesity. *Best Practice and Research Clinical Endocrinology and Metabolism*, **19**, 441–454.

Epstein, L. H., Myers, M. D., Raynor, H. A. & Saelens, B. E. (1998) Treatment of pediatric obesity. *Pediatrics*, **101**, 554–570.

Frenn, M., Malin, S. & Bansal, N. K. (2003a) Stage-based interventions for low-fat diet with middle school students. *Journal of Pediatric Nursing*, **18**, 36–45.

Frenn, M., Malin, S., Bansal, N., Delgado, M., Greer, Y., Havice, M., Ho, M. & Schweizer, H. (2003b) Addressing health disparities in middle school students' nutrition and exercise. *Journal of Community Health Nursing*, **20**, 1–14.

Geiss, H. C., Parhofer, K. G. & Schwandt, P. (2001) Parameters of childhood obesity and their relationship to cardiovascular risk factors in healthy prepubescent children. *International Journal of Obesity and Related Metabolic Disorders*, **25**, 830–837.

Goran, M. I. (1998) Measurement issues related to studies of childhood obesity: assessment of body composition, body fat distribution, physical activity, and food intake. *Pediatrics*, **101**, 505–518.

Harrell, T. K., Davy, B. M., Stewart, J. L. & King, D. S. (2005) Effectiveness of a school-based intervention to increase health knowledge of cardiovascular disease risk factors among rural Mississippi middle school children. *Southern Medical Journal*, **98**, 1173–1180.

Iwata, F., Hara, M., Okada, T., Harada, K. & Li, S. (2003) Body fat ratios in urban Chinese children. *Pediatrics Internation*, **45**, 190–192.

James, P. T., Leach, R., Kalamara, E. & Shayeghi, M. (2001) The worldwide obesity epidemic. *Obesity Research*, **9**, S228–S233.

Kiess, W., Bottner, A., Raile, K. *et al.* (2003) Type 2 diabetes mellitus in children and adolescents: a review from a European perspective. *Hormone Research*, **59**, S77–S84.

Koletzko, B., Girardet, J. P., Klish, W. & Tabacco, O. (2002) Obesity in children and adolescents worldwide: current views and future directions – Working Group Report of the First World Congress of Pediatric Gastroenterology, Hepatology, and Nutrition. *Journal of Pediatric Gastroenterological Nutrition*, **35**, S205–S212.

Luo, J. & Hu, F. B. (2002) Time trends of obesity in pre-school children in China from 1989 to 1997. *International Journal of Obesity*, **26**, 553–558.

Luo, Z. (2002) Obesity: a warning to Chinese children. *Beijing Review*, **45**, 14–16.

Ramachandran, A., Snehalatha, C., Vinitha, R., Thayyil, M., Kumar, C. K., Sheeba, L., Joseph, S. & Vijay, V. (2002) Prevalence of overweight in urban Indian adolescent school children. *Diabetes Research and Clinical Practice*, **57**, 185–190.

Rudolf, M. C., Sahota, P., Barth, J. H. & Walker, J. (2001) Increasing prevalence of obesity in primary school children: cohort study. *British Medical Journal*, **322**, 1094–1095.

Sorof, J. M., Poffenbarger, T., Franco, K., Bernard, L. & Portman, R. J. (2002) Isolated systolic hypertension, obesity, and hyperkinetic hemodynamic states in children. *Journal of Pediatrics*, **140**, 660–666.

Steckler, A., Ethelbah, B., Martin, C. J. *et al.* (2003) Pathways process evaluation results: a school-based prevention trial to promote healthful diet and physical activity in American Indian third, fourth, and fifth grade students. *Prevention Medicine*, **37**, S80–S90.

Summerbell, C. D., Waters, E., Edmunds, L. D., Kelly, S., Brown, T. & Campbell, K. J. (2005) Interventions for preventing obesity in children. *Cochrane Database of Systematic Reviews*, **20**, CD001871.

Whitaker, R. C., Wright, J. A., Pepe, M. S., Seidel, K. D. & Dietz, W. H. (1997) Predicting obesity in young adulthood from childhood and parental obesity. *New England Journal of Medicine*, **337**, 869–873.

Wilson, P., O'Meara, S., Summerbell, C. & Kelly, S. (2003) The prevention and treatment of childhood obesity. *Quality and Safety in Health Care*, **12**, 65–74.

© 2007 The Authors
