# Supplementary material for: Systematic review and meta-analysis of school-based obesity interventions in mainland China
Source: PLoS One. 2017 Sep 14;12(9):e0184704. doi: 10.1371/journal.pone.0184704 (PMC5598996; doi:10.1371/journal.pone.0184704)
Supplement: S1 Dataset — (ZIP) [file pone.0184704.s007.zip › S1_dataset/76库/23.pdf]

BIOMEDICAL AND ENVIRONMENTAL SCIENCES 23, 180-187 (2010) www.besjournal.com

Report on Childhood Obesity in China (8): Effects and Sustainability of Physical Activity Intervention on Body Composition of Chinese Youth[1]

YAN-PING LI[#,+,†,‡], XIAO-QI HU[#], EVERT G. SCHOUTEN[+], AI-LING LIU[#], SONG-MING DU[#], LIN-ZHONG LI[#], ZHAO-HUI CUI[#], DONG WANG[#], FRANS J KOK[+], FRANK B HU[†], AND GUAN-SHENG MA[#]

[#]*National Institute for Nutrition and Food Safety, Chinese Center for Disease Control and Prevention, Beijing 100050, China;* [+]*Division of Human Nutrition, Wageningen University, The Netherlands;* [†]*Departments of Nutrition and Epidemiology, Harvard School of Public Health, USA;* [‡]*Department of Global Health and Population, Harvard School of Public Health, USA*

Objectives To determine whether a large-scale physical activity intervention could affect body composition in primary school students in Beijing, China. **Methods** The study design was one-year cluster randomized controlled trial of physical activity intervention (20 min of daily exercise in the classroom) with an additional year of follow-up among 4 700 students aged 8-11 years at baseline. **Results** After the one-year intervention, BMI increased by 0.56 kg/m^2 (SD 1.15) in the intervention group and by 0.72 kg/m^2 (SD 1.20) in the control group, with a mean difference of -0.15 kg/m^2 (95% CI: -0.28 to -0.02). BMI z score decreased by -0.05 (SD 0.44) in the intervention group, but increased by 0.01 (SD 0.46) in the control group, with a mean difference of -0.07 (-0.13 to -0.01). After another year of follow up, compared to the control group, children in the intervention group had significantly lower BMI (-0.13, -0.25 to -0.01), BMI z score (-0.05, -0.10 to -0.01), fat mass (-0.27 kg, -0.53 to -0.02) and percent body fat (-0.53, -1.00 to -0.05). The intervention had a more pronounced effect on weight, height, BMI, BMI z score, and body composition among obese children than among normal weight or overweight children. Compared to the control group, the intervention group had a significantly higher percentage of children who maintained or reduced their BMI z score at year 1 (P=0.008) and year 2 (P=0.04). **Conclusions** These findings suggest that 20 min of daily moderate to vigorous physical activity during the school year is a feasible and effective way to prevent excessive gain of body weight, BMI, and body fatness in primary school students.

Key words: Intervention; BMI; School; China

INTRODUCTION

With more than 25% of Chinese adults overweight, China is facing a major epidemic of obesity[1]. In 2000, obesity-related direct (medical care) and indirect (disability, mortality, and sickness) costs accounted for 4.06% of China's gross national product (GNP). That figure is expected to reach 9.23% in 2025[2]. Obesity-related chronic diseases have become the most important preventable causes of death in China[3].

Overweight and its comorbidities have also affected the younger generation. Li *et al.* recently reported that 6.2% of Chinese children, or 16 million, suffer from overweight or obesity[4]. Among this group, 75.9% have at least one metabolic abnormality and 20.4% have the metabolic syndrome[5]. In addition, childhood obesity significantly increases the risk of morbidity and mortality in adulthood[6].

The developmental years of childhood are a critical time for instilling positive behavior and lifestyle habits. However, interventions should not single out children at risk of obesity[7] or contribute to the emotional distress of those who are already overweight or obese[8]. Likewise, they must not interfere with the adequate growth or development of those who are underweight[7]. Physical activity can reduce fat mass and preserve lean body mass by increasing total energy expenditure and fat oxidation[9]. Therefore, increasing physical activity is considered an important strategy for prevention of childhood obesity.

The Happy 10 program was developed as a classroom-based intervention to promote physical activity among primary school students[10]. Prior to its implementation, a pilot study had been conducted in two schools, an intervention and a control[10]. Data

[1]This research was supported by Nutricia Research Foundation.

[2]Correspondence should be addressed to Guan-Sheng MA. Tel: 86-10-67776285. Fax: 86-10-67711813. E-mail: mags@chinacdc.net.cn; or to Yan-Ping LI: liyanping72@yahoo.com

Biographical note of the first author: Yan-Ping LI, female, born in 1972, associate professor.

0895-3988/2010
CN 11-2816/Q
Copyright © 2010 by China CDC

?1994-2015 China Academic Journal Electronic Publishing House. All rights reserved. http://www.cnki.net

indicated that the program was feasible, well-accepted, and effective in reducing weight gain among children who took part in it. Based on these findings, we implemented the Happy 10 program in 20 schools in the same geographic region. This study was designed to evaluate the effectiveness and sustainability of this program among 4 700 primary school students. A positive outcome would justify the implementation of a nationwide school-based effort to prevent childhood obesity in China.

METHODS AND PROCEDURES

Study Design

This was a cluster randomized controlled trial. We randomly selected two districts, DongCheng and ChongWen, from the eight in urban Beijing. Then ten primary schools from each district were randomly chosen and assigned to be either an intervention or control group (Fig. 1). The intervention was conducted during the 2005-2006 school year, with a subsequent 1-year follow-up.

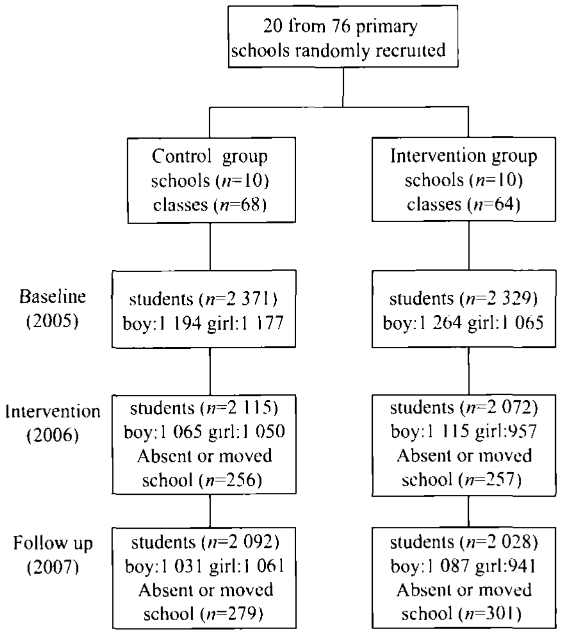

FIG. 1. Flow chart for the intervention study. Ten primary schools from two districts in Beijing were randomly chosen and assigned to be either an intervention or control group. Out of 4 880 students, a total of 4 700 (response rate 96%) participated in the study in 2005. In 2006 and 2007, 513 (10.9%) and 580 (12.3%) students dropped out, respectively.

Teachers organized and implemented the Happy 10 program twice daily for 10 min with 3rd and 4th grade students. No intervention took place in the control schools. The program did not replace other regular activity programs, including physical education and after-school activities. Rather, it was incorporated into the curriculum. The study was approved by the Chinese Ethical Review Committee of the National Institute for Nutrition and Food Safety in China and the Dutch Medical Ethical Review Committee of Wageningen University (METC-WU) in the Netherlands. Both a written consent form from a parent and an oral consent from a child were required for participation.

Intervention

Based on the principle of TAKE10!® (http://www.take10.net), the Happy 10 program was initiated jointly by the National Institute for Nutrition and Food Safety, Chinese Center for Disease Control and Prevention (CDC), and the International Life Science Institute Focal Point in China. It consisted of two daily 10-min physical activity sessions conducted in the break between classes.

The program provided a variety of safe, moderate, age-, and space-appropriate exercises. Teaching materials included activity cards, video demonstrations, tracking posters, and stickers. Each activity card introduced one exercise and explained how to perform it. The videos showed students from the pilot study performing the activities. Teachers could either demonstrate the activity or show it on a video. The tracking poster and stickers were used to illustrate the progress of each class.

There were several activity models directly from TAKE 10! Program, such as "invisible jump rope"; "copy cat"; "all about you"; "stories on the move!"; "stories in space". Clear introduction were colourfully printed in the activity card. Students, teachers and parents were encouraged to develop new activity models, so did the program staffs. Many new programs, much more than that directly from TAKE 10!, were developed, such as "Story in zoo"; "story in farm"; "who is wearing yellow today"; "time like a colt"; "happy and health"; "little frog".

The 10-minute sessions consisted of four parts: 1) the teacher or student selected the cards to determine the activities; 2) several children were chosen to model the exercises in the front of the classroom and the other students followed along (one to three activities were performed at each session); 3) a cool-down period took place after the activities; and 4) the students were taught a health message. If they chose the "invisible jump rope", each student pretended to have an invisible jump rope and began to jump. Teacher called out numbers from 1-10 starting with one. Everyone jumped as they counted up to that number. Starting at 20 and counting backwards, while students did the invisible jump rope backwards. The students jumped more and more quickly as the teacher

?1994-2015 China Academic Journal Electronic Publishing House. All rights reserved. http://www.cnki.net

was increasing the counting speed. Some teachers also combined math calculating into the activities.

The average caloric expenditure for both 10-minute sessions ranged from 60 to 70 kcal/ school day, which translated to 43-50 kcal/day, as measured by physical activity sensors[10]. The average metabolic equivalent task (MET) rate/session ranged from 4.8 to 7.3 kcal $kg^{-1}h^{-1}$[10]. All activities were of moderate to vigorous intensity.

Staff Training

Teachers, usually classroom tutors and/or health educators, attended a half-day training session conducted by the staff of the National Institute for Nutrition and Food Safety, China CDC. They learned how to integrate the program into the school curriculum, and how to perform the activities. Information about childhood obesity, risk factors, health consequences, and prevention were an integral part of the training. Teachers modelled the lessons to ensure that they understood the recommended techniques and strategies for implementation.

Outcome Measures

Measurements were collected in the summers of 2005 (baseline), 2006 (intervention year 1) and 2007 (follow-up year 2). Children fasted the night before and were measured the next morning by trained research staff. Consistent assessment methods were used throughout the study. The research staffs who conducted the measurement were blinded to the intervention assignment.

Height was measured to an accuracy of 1mm with a free-standing stadiometer mounted on a rigid tripod. Fasting body weight was measured to the nearest 0.1 kg on a digital scale. BMI was calculated as weight in kilograms divided by height in meters squared (kg/m^2) The BMI z score, based on age and gender, was calculated for each student using WHO growth references (http://www.who.int/growthref/who2007_bmi_for_age/en/index.html). Each particip ant was classified into 1 of 4 weight categories: underweight (BMI z score < -2 SD), normal weight (BMI z score between -2 SD and 1 SD), overweight (BMI z score between 1 SD and 2 SD), and obese (BMI z score > 2 SD).

Body composition was measured by bioelectrical impedance analysis. Resistance (R) and reactance (Xc) were determined using a four terminal impedance plethysmograph (RJL2System 101 USA, 50 kHz, 800 μA). Body impedance was calculated as the square root of (R^2+Xc^2). Fat free mass (FFM), fat mass, and percent body fat were calculated using the prediction equations suggested by Deurenberg *et al.*[11].

Statistical Methods

Based on data from the pilot study, average one-year BMI change in the control group was 0.6 kg/m^2 with a standard deviation of 1.1 kg/m^2; the intraclass correlation was 0.15. The sample size of 4 700 students from 20 schools had 90% power to detect a mean between-group difference in BMI of 0.35 units (effect size of 0.3). Statistical significance was set at 5% (two-sided).

Anthropometric measurements at baseline were compared using multivariate regression analysis with age and sex in the model. The effects of intervention were analyzed using SAS proc mixed procedure (SAS 8.2e for windows, SAS Institute Inc. Cary, NC.), with BMI changes from baseline as the primary outcome variable. The fixed effects included baseline BMI, age, sex, and the intervention group. While the randomization was at the school level, implementation was at the class level. Thus, the classroom was treated as a random effect variable. Similar mixed models were constructed for other outcome variables, including weight, height, BMI z score, fat free mass, fat mass, and percent body fat.

The analysis was based only on children measured at baseline and again in year 1 or year 2. Participants who were lost to follow-up were excluded from the analysis. We conducted a separate analysis for different weight status at baseline as well as for boys and girls. Statistical interactions between intervention and gender as well as baseline weight status were conducted using the likelihood ratio tests.

RESULTS

Out of the 4 880 students enrolled, a total of 4 700 (response rate 96%) participated in the study in 2005. In 2006 and 2007, 513 (10.9%) and 580 (12.3%) students dropped out, respectively (Fig. 1). No school or class dropped out of the study. Subjects lost to follow-up and those who remained in the program had similar characteristics (Table 1). A[illegible] baseline, there were no significant difference[illegible] between the intervention and control groups i[illegible] weight, height, BMI, BMI z score, fat free mas[illegible] (FFM), fat mass, percent body fat, family income level, or mother's educational background.

no drop out
开始时两组无差异

After the intervention, BMI increased by 0.56 kg/m^2 (SD 1.15) in the Happy 10 group and by 0.72 kg/m^2 (SD 1.20) in the control group, with a mean difference of -0.15 kg/m^2 (95% confidence interval [CI]: -0.28 to -0.02). In the intervention group, BMI z score decreased by -0.05 (SD 0.44), but increased by 0.01 (SD 0.46) in the control group. The mean difference was -0.07 (95% CI; -0.13 to -0.01). Body

?1994-2015 China Academic Journal Electronic Publishing House. All rights reserved. http://www.cnki.net

weight increased in both groups, with a mean difference of -0.33 kg (95% CI; -0.64 to -0.02) (Table 2). Significant effects were sustained one year after the program stopped. Compared to those in the control group, children in the intervention group had significantly lower BMI (-0.13, 95% CI -0.25 to -0.01), BMI z score (-0.05, 95%CI -0.10 to -0.01), fat mass (-0.27 kg, 95% CI -0.53 to -0.02) and percent body fat (-0.53, 95% CI -1.00 to -0.05) in year 2 (Table 2).

The intervention had similar effects on weight, BMI, and BMI z score in boys and girls during the one-year intervention period. It had a significant effect on fat mass and percent body fat in girls, but not in boys. Significant interaction was found between gender and intervention on percent body fat (Table 3). One year after the intervention, the effects were sustained in girls, but not in boys.

The intervention had a more pronounced effect on weight, height, BMI, BMI z score, and body composition among obese children than among normal weight or overweight children at baseline (Table 4). Compared to those in the control group, obese children in the intervention group had significantly lower BMI (-0.35, 95% CI -0.62 to -0.08) and BMI z score (-0.07, 95% CI -0.13 to -0.02). Significant effects were sustained one year after the program stopped. However, no significant interaction was detected between intervention and baseline weight status (P for interaction ranged from 0.07 on weight to 0.98 on fat mass except P for interaction on fat free mass in year 1, which was 0.005).

No significant difference was found between the intervention and control groups in the prevalence, incidence, or remission of overweight and obesity. However, compared to the control group (49.5%), the Happy 10 group (57.3%) had a significantly higher percentage of children who maintained or reduced their BMI z score in both boys and girls after one year intervention (P=0.008) (Fig. 2). During the two-year period, 46.9% of girls in the intervention group and 41.5% of girls in the control group maintained or reduced their BMI z score (P=0.02). This difference was not significant in boys.

TABLE 1

Characteristics of Subject and Lost to Follow-up

		Baseline (2005)	Lost to Follow-up (2006)	Lost to Follow-up (2007)
Sex	Girls	2242	235	240
	Boys	2458	278	340[1]
Group	Intervention	2329	257	301
	Control	2371	256	279
Age (years)		9.3±0.7	9.3±0.8	9.4±0.8[2]
Height (cm)		140.4±7.2	140.5±7.3	140.5±7.1
Weight (kg)		35.4±9.6	35.5±9.7	35.3±9.8
BMI (kg/m^2)		17.8±3.6	17.8±3.7	17.7±3.7
BMI Z Score		0.29±1.47	0.28±1.5	0.24±1.5
Percent Body Fat (%)		25.4±4.9	25.1±5.0	25.0±5.0

Note. Comparison between the subjects available for follow-up and those lost to follow up, [1]χ^2-test, P<0.05; [2]t-test, P<0.05.

TABLE 2

Changes from Baseline in Body Weight, Body Mass Index (BMI), BMI Z Scores, and Body Composition at Year 1 and Year 2

	Intervention	Control	Mean Difference (95% CI)	P Value
**Weight (kg)**				
Baseline[1] (n=4700)	35.5 (9.6)	35.4 (9.6)	-0.09 (-0.62 to 0.43)	0.72
Year 1 Changes[2] (n=4187)	4.4 (2.8)	4.7 (2.9)	-0.33 (-0.64 to -0.02)	0.04
Year 2 Changes[2] (n=4121)	10.0 (5.0)	10.3 (4.7)	-0.31 (-0.63 to 0.02)	0.06
**Height (cm)**				
Baseline[1] (n=4700)	140.4 (7.2)	140.4 (7.3)	-0.12 (-0.50 to 0.26)	0.53
Year 1 Changes[2] (n=4187)	6.0 (1.8)	6.2 (1.8)	-0.03 (-0.26 to 0.20)	0.77
Year 2 Changes[2] (n=4121)	12.1 (2.8)	12.1 (2.9)	-0.04 (-0.24 to 0.23)	0.97
**BMI (kg/m^2)**				
Baseline[1] (n=4700)	17.79 (3.61)	17.74 (3.61)	-0.02 (-0.22 to 0.18)	0.85
Year 1[2] (n=4187)	0.56 (1.15)	0.72 (1.20)	-0.15 (-0.28 to -0.02)	0.03

?1994-2015 China Academic Journal Electronic Publishing House. All rights reserved. http://www.cnki.net

(continued)

	Intervention	Control	Mean Difference (95% CI)	*P* Value
Year 2[2] (*n*=4121)	1.55 (1.84)	1.67 (1.63)	-0.13 (-0.25 to -0.01)	0.04
**BMI Z Scores**				
Baseline[1] (*n*=4700)	0.31 (1.48)	0.28 (1.47)	-0.01 (-0.09 to 0.07)	0.78
Year 1[2] (*n*=4187)	-0.05 (0.44)	0.01 (0.46)	-0.07 (-0.13 to -0.01)	0.03
Year 2[2] (*n*=4121)	0.03 (0.72)	0.08 (0.59)	-0.05 (-0.10 to -0.01)	0.03
**Fat Free Mass (kg)**				
Baseline[1] (*n*=4693)	26.2 (5.9)	26.1 (5.9)	-0.13 (-0.44 to 0.18)	0.42
Year 1[2] (*n*=4163)	4.0 (1.9)	4.3 (2.1)	-0.18 (-0.41 to 0.05)	0.12
Year 2[2] (*n*=4111)	7.7 (3.2)	7.7 (3.2)	-0.03 (-0.30 to 0.24)	0.82
**Fat mass (kg)**				
Baseline[1] (*n*=4693)	9.3 (4.1)	9.3 (4.2)	0.02 (-0.22 to 0.25)	0.87
Year 1[2] (*n*=4163)	0.3 (1.7)	0.5 (1.8)	-0.15 (-0.37 to 0.07)	0.18
Year 2[2] (*n*=4111)	2.3 (3.0)	2.6 (2.5)	-0.27 (-0.53 to -0.02)	0.03
**Percent Body Fat (%)**				
Baseline[1] (*n*=4693)	25.3 (4.9)	25.5 (5.0)	0.11 (-0.17 to 0.39)	0.45
Year 1[2] (*n*=4163)	-2.2 (3.2)	-2.0 (3.2)	-0.22 (-0.70 to 0.24)	0.34
Year 2[2] (*n*=4111)	-0.9 (5.0)	-0.4 (3.7)	-0.53 (-1.00 to -0.05)	0.03

Note. [1]Based on the maximum number of children.[2] Based on children with data at baseline and again at either year 1 (intervention) or year 2 (follow-up).

TABLE 3

Main Intervention Effects (95% CI) for Boys and Girls

	Girls		Boys		*P* Value of
	Mean Difference (95% CI)	*P* Value	Mean Difference (95% CI)	*P* Value	Interaction[3]
**Weight (kg)**					
Baseline[1]	-0.21 (-0.89 to 0.47)	0.55	0.01 (-0.40 to 0.61)	0.69	
Year 1 Changes[2]	-0.37 (-0.71 to -0.03)	0.04	-0.36 (-0.71 to -0.01)	0.046	0.75
Year 2 Changes[2]	-0.44 (-0.85 to -0.04)	0.03	-0.14 (-0.60 to 0.28)	0.51	0.44
**Height (cm)**					
Baseline[1]	-0.35 (-0.91 to 0.21)	0.22	-0.02 (-0.32 to 0.28)	0.88	
Year 1 Changes[2]	-0.01 (-0.27 to 0.26)	0.96	-0.07 (-0.31 to 0.17)	0.57	0.25
Year 2 Changes[2]	0.02 (-0.26 to 0.30)	0.88	0.05 (-0.23 to 0.33)	0.71	1.00
**BMI (kg/m^2)**					
Baseline[1]	-0.02 (-0.28 to 0.25)	0.90	-0.02 (-0.22 to 0.18)	0.85	
Year 1[2]	-0.17 (-0.31 to -0.03)	0.02	-0.15 (-0.31 to -0.001)	0.049	0.75
Year 2[2]	-0.19 (-0.34 to -0.03)	0.02	-0.08 (-0.25 to 0.09)	0.37	0.27
**BMI Z Scores**					
Baseline[1]	0.00 (-0.11 to 0.11)	0.97	-0.02 (-0.14 to 0.10)	0.74	
Year 1[2]	-0.08(-0.14 to -0.02)	0.01	-0.06(-0.12 to 0.01)	0.09	0.29
Year 2[2]	-0.07(-0.13 to -0.01)	0.02	-0.03 (-0.10 to 0.03)	0.29	0.32
**Fat Free Mass (kg)**					
Baseline[1]	-0.20 (-0.62 to 0.22)	0.35	-0.06 (-0.52 to 0.40)	0.79	
Year 1[2]	-0.11 (-0.37 to 0.15)	0.41	-0.25 (-0.52 to 0.01)	0.06	0.12
Year 2[2]	-0.03(-0.33 to 0.28)	0.85	0.03 (-0.29 to 0.36)	0.84	1.00
**Fat Mass (kg)**					
Baseline[1]	-0.03 (-0.32 to 0.27)	0.87	0.06 (-0.30 to 0.42)	0.76	
Year 1[2]	-0.25 (-0.48 to -0.02)	0.03	-0.11 (-0.37 to 0.16)	0.42	0.14
Year 2[2]	-0.45 (-0.70 to -0.20)	<.001	-0.15 (-0.49 to 0.19)	0.39	0.11
**Percent Body Fat (%)**					
Baseline[1]	0.08 (-0.29 to 0.45)	0.67	0.12 (-0.29 to 0.54)	0.56	
Year 1[2]	-0.49 (-1.002 to 0.03)	0.06	-0.09 (-0.63 to 0.44)	0.74	0.02
Year 2[2]	-0.80 (-1.29 to -0.31)	0.002	-0.35(-0.96 to 0.25)	0.24	0.11

Note. [1]Based on the maximum number of children. [2] Based on children with data at baseline and again at either year 1 (intervention) or year 2 (follow up). [3] Interaction between gender and intervention effect, based on (chi-square) the difference of -2 log likelihood between models with and without the interaction (between gender and intervention effect).

?1994-2015 China Academic Journal Electronic Publishing House. All rights reserved. http://www.cnki.net

TABLE 4

Effects of Intervention according to Children's Body Weight Status at Baseline

	Baseline		Intervention			Follow up		
			Change in 1 Year		Intervention Effect[3] (95% CI)	Change in 2 Years[2]		Intervention Effect[3] (95% CI)
	Intervention	Control[1]	Intervention[2]	Control[2]		Intervention[2]	Control[2]	
**Underweight (n=232)**								
Weight (kg)	24.1±2.5	24.5±2.8	2.7±2.0	3.0±1.5	-0.23 (-0.75,0.29)	7.0±5.9	6.9±5.3	0.18(-1.49,1.85)
Height (cm)	135.7±6.1	136.7±6.7	5.4±1.5	5.4±1.3	0.06(-0.36,0.48)	11.2±2.4	11.3±2.3	-0.04(-0.73,0.65)
BMI	13.03±0.44	13.05±0.51	0.35±0.89	0.53±0.66	-0.12(-0.36,0.12)	1.30±2.71	1.22±2.64	0.09(-0.69,0.87)
BMI Z Score	-2.46±0.39	-2.47±0.42	-0.02±0.59	0.17±0.58	-0.12(-0.31,0.07)	0.26±0.96	0.24±0.92	0.04(-0.25,0.33)
Fat Free Mass (kg)	19.2±2.4	19.4±2.5*	2.8±1.2	2.9±1.2	-0.10(-0.46,0.27)	5.6±2.8	5.5±2.6	0.23(-0.62,1.08)
Fat Mass (kg)	4.8±0.8	5.0±0.8	0.0±1.2	0.1±0.9	-0.24(-0.53,0.05)	1.3±3.6	1.4±3.5	-0.06(-1.06,0.96)
Percent Body Fat (%)	20.1±3.4	20.7±3.0*	-2.3±3.2	-1.8±2.8	-0.65(-1.47,0.15)	-0.9±4.5	-0.7±4.9	-0.35(-1.70,0.34)
**Normal Weight (n =2 940)**								
Weight (kg)	31.0±4.8	31.0±4.7	3.9±2.2	4.2±2.5	-0.29(-0.60,0.02)	9.1±3.9	9.3±3.8	-0.26(-0.58,0.06)
Height (cm)	139.2±7.0	139.1±6.9	6.0±1.8	6.2±1.8	-0.06(-0.31,0.17)	12.0±2.9	12.1±2.9	-0.11(-0.38,0.17)
BMI	15.94±1.40	15.93±1.36	0.53±0.87	0.67±1.09	-0.13(-0.26,0.001)	1.50±1.42	1.58±1.38	-0.09(-0.20,0.03)
BMI Z Score	-0.37±0.79	-0.38±0.77	-0.02±0.44	0.05±0.48	-0.06(-0.13,0.01)	0.13±0.59	0.17±0.58	-0.04(-0.09,0.01)
Fat Free Mass (kg)	23.8±3.7	23.7±3.6	3.8±1.7	4.0±1.8	-0.14(-0.37,0.10)	7.2±2.8	7.2±2.8	-0.05(-0.32,0.23)
Fat Mass (kg)	7.2±1.7	7.3±1.8	0.1±1.4	0.3±1.6	-0.17(-0.39,0.05)	1.9±2.2	2.1±2.1	-0.21(-0.43,0.01)
Percent Body Fat (%)	23.3±3.6	23.5±3.8	-2.4±3.2	-2.1±3.3	-0.30(-0.82,0.21)	-0.8±3.9	-0.3±3.7	-0.45(-0.90,0.01)
**Overweight (n =830)**								
Weight (kg)	40.5±4.7	40.4±4.9	5.2±3.1	5.4±2.8	-0.23 (-0.75,0.27)	11.4±4.8	11.5±4.4	-0.09(-0.76,0.57)
Height (cm)	142.1±6.2	141.8±6.8	6.1±1.8	6.3±1.8	-0.15(-0.45,0.16)	12.3±2.8	12.3±3.0	0.04(-0.39,0.48)
BMI	19.98±1.08	20.03±1.08	0.74±1.39	0.80±1.26	-0.09(-0.31,0.14)	1.74±1.96	1.78±1.80	-0.06(-0.33,0.22)
BMI Z Score	1.46±0.28	1.47±0.28	-0.06±0.41	-0.04±0.41	-0.04(-0.10,0.03)	-0.08±0.77	-0.06±0.57	-0.02(-0.12,0.07)
Fat Free Mass (kg)	28.9±3.4	28.8±3.7	4.5±1.9	4.6±1.9	-0.14(-0.50,0.22)	8.6±3.0	8.3±2.9	0.31(-0.12,0.74)
Fat Mass (kg)	11.6±2.0	11.6±2.0	0.7±1.9	0.8±1.8	-0.11(-0.41,0.20)	2.7±3.2	3.1±2.7	-0.40(-0.89,0.10)
Percent Body Fat (%)	28.5±3.3	28.7±3.4	-1.8±2.8	-1.7±3.1	-0.17(-0.65,0.32)	-1.3±6.4	-0.4±3.9	-0.85(-1.77,0.07)
**Obese (n =698)**								
Weight (kg)	51.5±9.0	52.1±8.6	5.7±3.9	6.7±4.0	-0.83(-1.51,-0.15)*	13.3±7.0	14.2±5.4	-0.87(-1.86,0.13)
Height (cm)	145.0±6.9	145.7±6.5	6.1±1.5	6.2±1.6	-0.12(-0.43,0.19)	12.4±2.7	12.1±2.7	0.33(-0.09,0.75)
BMI	24.36±2.70	24.40±2.61	0.56±1.73	0.93±1.65	-0.35(-0.62,-0.08)*	1.63±2.72	2.09±1.87	-0.14(-0.81,-0.07)*
BMI Z Score	2.67±0.57	2.62±0.51	-0.20±0.40	-0.12±0.34	-0.07(-0.13,-0.02)*	-0.32±0.90	-0.17±0.38	-0.13(-0.24,-0.03)*
Fat Free Mass (kg)	35.1±5.7	35.3±5.2	4.8±2.3	5.5±2.6	-0.63(-1.05,-0.20)*	9.7±4.1	9.9±3.8	-0.14(-0.81,0.52)
Fat Mass (kg)	16.5±3.8	16.8±3.9	1.0±2.6	1.2±2.6	-0.22(-0.65,0.21)	3.6±4.3	4.2±2.9	-0.60(-1.18,-0.01)*
Percent Body Fat (%)	31.8±3.1	32.0±3.3	-1.6±3.2	-1.7±3.0	0.0(-0.50,0.50)	-1.3±6.9	-0.5±3.2	-0.97(-1.80,-0.15)*

Note. [1]Between-group-difference in baseline, t-test, *P<0.05 (two sides); [2] Within-group changes from baseline (growth); data are unadjusted means and sd. [3]Estimates for intervention effect (95% confidence interval) reflect the between-group difference, mixed model, adjusted baseline measurements, age, and clustering effect among classrooms, *P<0.05 (one side).

DISCUSSION

The overall objective of this study was to evaluate whether the Happy 10 program could prevent childhood obesity if implemented on a large scale. Over the course of one school year, we observed a moderately lower body weight, BMI, and BMI z score among children who participated in the intervention. Most of the intervention effects persisted one year after the intervention had stopped, especially among girls.

Cross-sectional studies showed that children who spent more time on moderate-to-vigorous physical activity were less likely to be obese than their inactive counterparts[12-13]. Prospective epidemiological studies also indicated that increasing physical activity was associated with lower weight gain in children[14].

However, school-based obesity prevention programs have yielded mixed results. In a systematic review, Doak *et al.*[7] found that simple physical activity intervention programs were more effective in preventing obesity in children and adolescents than complex intervention programs. In this trial, the Happy 10 program, with a small lifestyle change, did result in decreased weight, BMI, and BMI z scores in children. The intervention, however, had no effect on prevalence of obesity. This might be due to small changes in BMI z scores (effect size of 0.3) in a short period of time[15]. The happy 10 group had a

?1994-2015 China Academic Journal Electronic Publishing House. All rights reserved. http://www.cnki.net

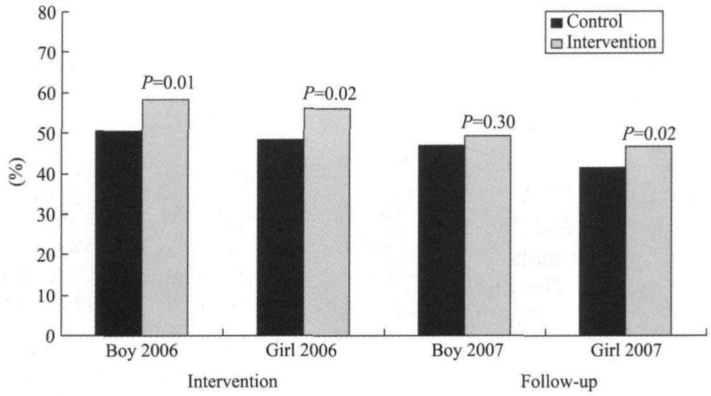

FIG. 2. Percentage of children in intervention and control groups who maintained or reduced their BMI z score[1]. [1]Adjusted for baseline BMI z score, age, and clustering within classrooms.

significantly high percentage of children who maintained or reduced their BMI for age (z score) and it was sustained one year after the intervention stopped. Such efforts can lead to appreciable reduction in body weight in the long run[16].

Girls and boys do not necessarily respond in the same way to a given intervention. Studies from France[17] and Crete[18] found that programs were effective in both boys and girls. However, the Planet Health[19] in the United States and the Kiel Obesity Prevention Study in Germany[20] showed a significant effect in girls only. On the other hand, studies in Chile[21] reported positive outcomes for boys only. Our intervention produced similar effects in boys and girls during the intervention period, but the significant effects were only sustained in girls one year after the program had stopped. Because girls usually pay more attention to body weight than boys[22], they might have kept on with exercises after the program had stopped. In a previous study, we found significant gender differences in body satisfaction among Chinese children, with girls wanting to be thinner and boys wanting to be heavier[22]. Chinese boys also tended to be more accepting or tolerant of overweight or obesity than Chinese girls. However, inconsistent findings on gender difference from various intervention studies warrant further research in this area.

Potential for harm caused by weight loss in underweight children is a concern with obesity intervention programs. An Italian study showed that obesity intervention increased the prevalence of underweight in school children[23]. However, a recent report in the United States found no adverse outcomes in underweight children[24]. In a Chilean obesity intervention study, underweight children increased their BMI z score[21]. Our intervention did not produce significant changes in either the prevalence of underweight or the BMI z scores of underweight children.

This study has several strengths. As a randomized controlled trial, it eliminated confounding by covariates. The large sample size provided sufficient power to detect a relatively small effect, and the length of follow-up allowed us to assess long-term outcomes. The relatively high rate of follow-up reduced potential bias due to loss to follow-up. Our outcome measures included changes in BMI and BMI z scores as well as changes in body composition.

This study also has several limitations. Inadequate daily documentation by teachers prevented us from quantifying adherence to the Happy 10 protocol. On occasions, visiting CDC staff found that teachers skipped some daily physical activities. This may, at least in part, have weakened the effect of the intervention. Another limitation is that we did not evaluate the children's diets. However, we do not expect that our physical activity intervention would influence dietary intakes of the children.

The Happy 10 program is a simple and practical intervention. The activities are designed to be implemented in the classroom. As such, they address the issue of limited sports space (a main reason cited by 38.7% of the children for lack of activity)[25]. In general, the program was easy to implement and well-accepted by the schools, teachers, and students. The data suggest that 20 min of moderate physical activity per day during the school year is a feasible and appropriately effective approach to prevent excessive gain of body weight, BMI, and BMI z scores in 3rd and 4th grade children in Beijing, China. The modest results also persisted one year after the intervention. These findings suggest that the Happy 10 program can be used for primary prevention of childhood obesity in China. Nutrition education and dietary intervention should be considered to be integrated into the Happy 10 program in order to strengthen the intervention effects.

?1994-2015 China Academic Journal Electronic Publishing House. All rights reserved. http://www.cnki.net

ACKNOWLEDGEMENT

We thank all the students and their parents for their participation in this study. We appreciate the cooperation of the Center for Disease Control and Prevention of Beijing, Dongcheng Middle and Primary School Student Healthcare Center in Beijing, and the Center for Disease Control and Prevention of Chongwen District in Beijing. The authors declared no conflict of interest to disclose. The clinical trial registration number is ChiCTR-TRC-00000053 (Register URL: http://www.chictr.org).

REFERENCES

1. Wu Y (2006). Overweight and obesity in China. *BMJ* **333**, 362-363.
2. Popkin B M, Kim S, Rusev E R, *et al.* (2006). Measuring the full economic costs of diet, physical activity and obesity-related chronic diseases. *Obes Rev* 7, 271-293.
3. Gu D, He J, Duan X, *et al.* (2006). Weight and mortality among men and women in China. *JAMA* **295**, 776-783.
4. Li Y, Hu X, Zhao J, *et al.* (2009). Application of the WHO Growth Reference (2007) to Assess the Nutritional Status of Children in China. *Biomed Environ Sci* **22**, 130-135.
5. Li Y, Yang X, Zhai F, *et al.* (2008) Prevalence of the metabolic syndrome in Chinese adolescents. *Br J Nutr* **99**, 565-570.
6. Wabitsch M (2000). Overweight and obesity in European children: definition and diagnostic procedures, risk factors and consequences for later health outcome. *Eur J Pediatr* **159** (Suppl 1), S8-13.
7. Doak C M, Visscher T L, Renders C M, Seidell J C (2006). The prevention of overweight and obesity in children and adolescents: a review of interventions and programmes. *Obes Rev* 7, 111-136.
8. Gibson L Y, Byrne S M, Blair E (2008). Clustering of psychosocial symptoms in overweight children. *Aust N Z J Psychiatry* **42**(2), 118-125.
9. Epstein L H, Goldfield G S (1999). Physical activity in the treatment of childhood overweight and obesity: current evidence and research issues. *Med Sci Sports Exerc* **31**, 553-559.
10. Liu A, Hu X, Ma G, *et al.* (2008). Evaluation of a classroom-based physical activity promoting programme. *Obes Rev* **9**(Suppl 1), 130-134.
11. Deurenberg P, van der Kooy K, Leenen R (1991). Sex and age specific prediction formulas for estimating body composition from bioelectrical impedance: a cross validation study. *Int J Obes* **15**(1), 17-25.
12. Andersen R E, Crespo C J, Bartlett S J (1998). Relationship of physical activity and television watching with body weight and level of fatness among children: results from the Third National Health and Nutrition Examination Survey. *JAMA* **279**, 938-942.
13. Hernandez B, Gortmaker S L, Colditz G A (1999). Association of obesity with physical activity, television programs and other forms of video viewing among children in Mexico City. *Int J Obes Relat Metab Disord* **23**, 845-854.
14. Kimm S Y, Glynn N W, Obarzanek E (2005). Relation between the changes in physical activity and body-mass index during adolescence: a multicentre longitudinal study. *Lancet* **366**, 301-307.
15. Chiolero A, Maximova K, Paradis G (2008). Changes in BMI: an important metric for obesity prevention. *Pediatrics* **122**, 683.
16. Hill J O (2009). Can a small-changes approach help address the obesity epidemic? A report of the Joint Task Force of the American Society for Nutrition, Institute of Food Technologists, and International Food Information Council. *AJCN* **89**, 477-484.
17. Lazaar N, Aucouturier J, Ratel S, *et al.* (2007). Effect of physical activity intervention on body composition in young children: influence of body mass index status and gender. *Acta Paediatr* **96**, 1315-1320.
18. Manios Y, Kafatos A, Mamalakis G (1998). The effects of a health education intervention initiated at first grade over a 3 year period: physical activity and fitness indices. *Health Educ Res* **13**, 593-606.
19. Gortmaker S L, Peterson K, Wiecha J, *et al.* (1999). Reducing obesity via a school-based interdisciplinary intervention among youth: Planet Health. *Arch Pediatr Adolesc Med* **153**(4), 409-418.
20. Danielzik S, Pust S, Landsberg B, Muller M J (2005). First lessons from the Kiel Obesity Prevention Study. *Int J Obes (Lond)* **29**(Suppl 2), S78-S83.
21. Kain J, Albala R U, Vio F, *et al.* (2004). School-based obesity prevention in Chilean primary schoolchildren: methodology and evaluation of a controlled study. *Int J Obes Relat Metab Disord* **28**, 483-493.
22. Li Y, Hu X, Ma W, *et al.* (2005). Body image perceptions among Chinese children and adolescents. *Body Image* **2**, 91-103.
23. Simonetti D'Arca A, Tarsitani G, Cairella M, *et al.* (1986). Prevention of obesity in elementary and nursery school children. *Public Health* **100**, 166-173.
24. Foster G D, Sherman S, Borradaile K E, *et al.* (2008). A policy-based school intervention to prevent overweight and obesity. *Pediatrics* **121**, e794-802.
25. Guan Y (2005). The physical activity of teenagers and influence factors in big cities in China. *J TjIPE* (*Tian Jin Ti Yu Xue Yuan Xue Bao*) **20**, 28-31. (In Chinese)

(*Received March* 12, 2010 *Accepted June* 9, 2010)

?1994-2015 China Academic Journal Electronic Publishing House. All rights reserved. http://www.cnki.net
