# Supplementary material for: Systematic review and meta-analysis of school-based obesity interventions in mainland China
Source: PLoS One. 2017 Sep 14;12(9):e0184704. doi: 10.1371/journal.pone.0184704 (PMC5598996; doi:10.1371/journal.pone.0184704)
Supplement: S1 Dataset — (ZIP) [file pone.0184704.s007.zip › S1_dataset/76库/29.pdf]

Weight Loss, Inflammatory Markers, and Improvements of Iron Status in Overweight and Obese Children

Liya Gong, MD[1], Feifei Yuan, MD[1], Jianhua Teng, MD[1,2], Xue Li, MD[1], Sining Zheng, MD[1], Liqun Lin, MD[1], Haoyuan Deng, MD[1], Guansheng Ma, PhD[3], Changhao Sun, PhD[1], and Ying Li, PhD[1]

Objective To assess the effect of a weight-loss program on improving iron status in overweight and obese school-aged children.

Study design The data were analyzed in overweight and obese children (7-11 years of age; 114 girls and 212 boys) with body mass index-for-age z-scores (BAZ) >1 from a weight-loss program. Schools were randomly divided into 2 groups: intervention and control. Children in the intervention group underwent a 1-year, nutrition-based comprehensive intervention weight-loss program. Anthropometric, dietary intake, and physical activity data were collected at baseline and follow-up (1 year). Iron status and inflammatory markers were assessed within a month.

Results In the intervention group, BAZ decreased more than that in the control group (-0.4 ± 0.7 vs -0.1 ± 0.6, $P < .0001$); and iron profiles and inflammation status were improved at follow-up. In multivariable linear regression models, a greater decrease of BAZ and inflammation factors predicted a better improvement of iron status. After adjustment of ΔBAZ, ΔC-reactive protein was significantly associated with Δserum ferritin (β: 1.89; 95% CI, 0.70-3.09; $P = .002$) and Δsoluble transferrin receptor (β: 0.88; 95% CI, 0.16-0.59; $P = .017$); Δinterleukin-6 was significantly associated with Δserum ferritin (β: 1.22; 95% CI, 0.64-1.79; $P < .0001$).

Conclusions Iron status and inflammation were improved by weight reduction. The improvement in inflammatory markers during weight reduction was independently associated with improvements of iron status. *(J Pediatr 2014;164:795-800)*.

See related article, p 928

Iron deficiency (ID) is the most common and prevalent micronutrient deficiency in the world.[1] Although the current worldwide prevalence of ID is unavailable in children, it has been estimated that 33% of children between 5 and 15 years of age are iron deficient.[2] It is the only micronutrient deficiency that is prevalent in developing and industrialized countries[3] and has been linked to obesity in both adults and children.[4,5] Recent studies have reported that obese children are more susceptible to ID than normal-weight children.[6,7]

The etiology of ID in overweight and obese children is unclear. However, there are some explanations of the origin of this condition, including insufficient iron intake,[8] increased iron requirement as the result of larger blood volumes,[9] and poor iron absorption.[6] Cross-sectional studies have reported that there are no differences in the iron intake between obese and normal-weight children[7] and between obese and normal-weight adults of poor iron status even when consuming high levels of heme iron.[10] These studies, therefore, suggest that neither the amount nor the type of iron contribute to ID.

In addition, iron supplementation has been less effective in children with high body mass index-for-age z-scores (BAZ),[11] indicating that iron fortification or supplementation may not be an optimal way of improving iron status in obese children. In contrast, weight loss had significant improvements in the iron status of 20 morbidly obese premenopausal female patients who had restrictive bariatric surgery[12] and of 15 obese children and adolescents

BAZ	Body mass index-for-age z-scores	MLRM	Multivariable linear regression model
BF	Body fat	NISCOC	Nutrition-based comprehensive intervention study on childhood obesity
BMI	Body mass index		
China CDC	China Center for Disease Control and Prevention	SF	Serum ferritin
CRP	C-reactive protein	sTfR	Soluble transferrin receptor
Hb	Hemoglobin	TF	Transferrin
ID	Iron deficiency	WHO	World Health Organization
IL-6	Interleukin-6	WHtr	Waist-to-height ratio
MET	Metabolic equivalent task		

From the [1]Departments of Nutrition and Food Hygiene, School of Public Health, Harbin Medical University, China; [2]Institute for Tuberculosis Control and Prevention of Harbin, Harbin, China; and [3]National Institute for Nutrition and Food Safety, Chinese Center for Disease Control and Prevention, Beijing, China

Supported by National Science and Technology Pillar Program during the Eleven Five-Year Plan Period (2008BAI58B05), the Program for New Century Excellent Talents in University of Ministry of Education of China (NCET-10-0148), National Institute for Nutrition and Food Safety, and China Centers for Disease Control and Prevention. The authors declare no conflicts of interest.

Registered with Chinese clinical trial registry (http://www.chictr.org): ChiCTR-TRC-09000402.

0022-3476/$ - see front matter. Crown Copyright © 2014 Published by Mosby Inc. All rights reserved.
http://dx.doi.org/10.1016/j.jpeds.2013.12.004

who underwent a 6-month weight loss program.[13] Furthermore, we assessed the relationships between weight-loss, inflammation, and iron status in children.

Methods

The nutrition-based comprehensive intervention study on childhood obesity (NISCOC) program was a multicenter, randomized, parallel control trial for the prevention of obesity among 9750 primary students in China.[14] The NISCOC program consisted of 4 components, which are shown in **Table I**. Children in the control group received no intervention.

There were 1474 children who resided in Harbin and participated in the NISCOC program, provided fasting blood samples, and completed all of the questionnaires. In our study, we analyzed only the data of children with BAZ >1 according to World Health Organization (WHO) criteria[15] (n = 371) in this program. There were 1103 children with normal body weight, 10 children declined to participate, and 35 children with other reasons (acute infections before blood sampling or did not have lunch in school) in the overweight/obese children group were excluded at baseline. Finally, a total of 326 children (intervention group, 58 girls, 102 boys; control group, 56 girls, 110 boys) met inclusion criteria and entered this study; all of them completed the study (**Figure 1**; available at www.jpeds.com). The study was approved by the Ethics Committee of the National Institute for Nutrition and Food Safety of the Chinese Center for Disease Control and Prevention (China CDC) and adhered to Declaration of Helsinki principles. Signed written consent forms were obtained.

The detailed data collection of anthropometric measurements, dietary intake assessment, and physical activity and fitness measurements has been reported elsewhere.[14] The description of how the outcomes (body composition, diet, physical activity, physical fitness, iron status, and inflammation markers) have been assessed and the reasons why they have been selected were presented in **Table II** (available at www.jpeds.com).[16-28] Data were collected from June 2009 (baseline) through June 2010 (follow-up) by trained personnel and supervised by professionals. Investigators who conducted the measurement were blinded. Blood samples were collected following an overnight fast. Serum (5 mL) was obtained by centrifuging the blood samples at 3000 rpm (1000 g) for 10 min (TDL-5-A, Anting Scientific Instrument Factory, Shanghai, China). Serum samples were divided into aliquots and stored at −80°C. All the blood tests were analyzed within a month after blood collection. The indicators of iron status and inflammation markers were selected according to WHO/China CDC expert consultation[24] and WHO report[21] recommendation and previous study.[22,26,27]

Every tenth serum sample was measured twice (correlation coefficient of duplicate measurements was 0.98). The analytical sensitivity was 0.6 ng/mL for serum ferritin (SF), 1.5 ng/ml for transferrin (TF), 0.5 nmol/L for soluble transferrin receptor (sTfR), 0.1 mg/L for C-reactive protein (CRP), and 0.7 pg/mL for interleukin-6 (IL-6), respectively. The coefficients of variation (intra-assay) were <10% for SF, 4.3%-6.2% for sTfR, and 1.7%-4.4% for IL-6, respectively.

Statistical Analyses

Statistical analyses were performed by the use of IBM SPSS Statistics Version 20.0 software (IBM Corporation, Armonk, New York). Independent sample *t*-tests were used to assess differences in anthropometric measurements, dietary intake

Table I. Components of the NISCOC program

Component	Objective	Description
Physical activity	To encourage students to engage in more physical activities	One physical activity training class for teachers
	To increase energy expenditure	Under the guidance of trained teachers, children participated in the "Happy 10" campaign,* which consisted either of 2 times/day with 10 min of physical activity/time or of 1 time/day with 20 min of physical activity/time. This campaign also encouraged after-school physical activities.
Nutrition education	To create a pleasant atmosphere for children to learn about nutrition and physical activities	Four lectures about nutrition and health for teachers.
	To encourage children to learn about nutrition	One class per month (8 times/year) with organized essay competitions on nutrition and nutrition-knowledge contests.
	To promote healthy eating behaviors	Ten classes about the relationship between nutrition, physical activity, and health. These 40-min classes were held on a monthly basis. Children were provided with nutrition handbooks.†
Food service personnel	To teach the concept of balanced meals	Four lectures about nutrition.
	To reduce the consumption of cooking oil by 2-5 g	Cooking oil consumption was reduced by decreasing the preparation of fried foods. Additionally, school meals were frequently monitored by nutritionists.
Parents	To support the children about healthy diet behaviors learned at school	Three lectures about nutrition and health. Parents were provided with nutrition brochures.
	To reduce the consumption of cooking oil	Parents were provided with a limited amount of cooking oil.

Children in the intervention group participated in the intervention for 1 school year; children in the control group received no intervention.
*The "Happy 10" campaign is a classroom-based intervention developed to promote physical activity in school-aged children.
†The nutrition education handbook had a comic style and was developed by China CDC.

assessments, physical activity measurements, iron measures, and inflammatory markers between intervention and control groups at baseline or follow-up. To study the correlations between BAZ, iron measures, and inflammatory markers, a delta (Δ) value was calculated (Δ value = follow-up value − baseline value) and was assessed by using Pearson correlations. Bonferroni corrections were employed to reduce type I errors during the multiple correlations (**Table III**; available at www.jpeds.com). Iron measures, which were both significantly correlated with BAZ and inflammatory markers, were used as independent variables in multivariable linear regression models (MLRMs). MLRMs were used to assess the effect of ΔBAZ and inflammatory markers (ΔCRP and ΔIL-6) from baseline to follow-up (ie, 1 year) on iron status. Age, sex, and group were considered as potential confounders and were adjusted in the MLRM. The interactions between ΔBAZ, ΔCRP, and ΔIL-6 were not significant. Further adjustments for Δ average score of 3 physical fitness tests, Δ metabolic equivalent task (MET), and energy and iron intakes did not affect the results; therefore, we did not include them in the MLRM. $P < .05$ was considered to be statistically significant.

Results

Table IV shows the baseline, follow-up, and the Δ (change from baseline to follow-up) characteristics of anthropometric, dietary intake, and physical activity. Baseline characteristics, anthropometric measurements, and the number of overweight and obese children were comparable between the 2 groups. After the intervention, weight, waist, and body mass index (BMI) increased in both groups as a function of normal child growth and development. However, weight and BMI in the intervention group were significantly lower than in the control group at follow-up (45.5 ± 9.1 vs 47.6 ± 9.4, $P = .042$ and 21.9 ± 2.5 vs 22.6 ± 2.8, $P = .011$, respectively). The reduction of waist-to-height ratio (WHtr) and body fat (BF)% were significantly lower compared with the control group (−0.01 ± 0.04 vs 0.0 ± 0.04, $P < .0001$ and −3.4 ± 7.8 vs −1.5 ± 8.6, $P = .037$, respectively). After 1 year of nutrition education and physical activity, BAZ in the intervention group decreased by 0.4. BAZ was significantly lower in the intervention than in the control group at follow-up (1.6 ± 0.9 vs 2.0 ± 1.0, $P = .001$).

The fat, energy, and iron intakes between the 2 groups were comparable at baseline. However, fat intake in the intervention group was significantly lower than in the control group at follow-up (64.4 ± 28.6 g vs 71.3 ± 29.8 g, $P = .033$). Although there were no differences in the standing long jump physical test, the intervention group performed significantly better in the 2 other physical fitness tests (50-m sprint and 50 m*8 shuttle run) than the control group at follow-up (10.1 ± 1.4 seconds vs 10.9 ± 1.3 seconds, $P < .0001$; 123.3 ± 22.8 seconds vs 142.8 ± 25.8 seconds, $P < .0001$, respectively). MET, which is defined as the ratio of metabolic rate during a specific physical activity to a reference metabolic rate, was greater (7.5 ± 2.8 METs-h/d vs 6.1 ± 3.1 METs-h/d, $P < .0001$) in the intervention group than in the control group (**Figure 2**; available at www.jpeds.com).

Table V shows the iron measures and inflammation factors at baseline, follow-up, and the Δ (change from baseline to follow-up). Iron status and inflammation factors were comparable between the 2 groups at baseline. However, at follow-up, there was a significant improvement in iron profiles and inflammation status in the intervention group compared with the control group. At follow-up, the hemoglobin (Hb) and Ts% was significantly greater (12.4 ±

Table IV. Characteristics of the intervention and control groups at baseline, follow-up, and the change from baseline to follow-up

	Baseline		Follow-up		Δ				
	Intervention, n = 160	Control, n = 166	Intervention, n = 160	Control, n = 166	Intervention, n = 160	Control, n = 166	P*	P†	P‡
Anthropometric data									
Age, y	8.8 (1.3)	8.9 (1.3)	9.8 (1.3)	9.9 (1.3)	1	1	.963	.963	.963
Female/male	58/102	56/110	58/102	56/110			.634§	.634§	.634§
Weight, kg	40.8 (8.9)	41.7 (8.3)	45.5 (9.1)	47.6 (9.4)	4.7 (3.4)	5.9 (3.3)	.313	.042	.003
Waist, cm	69.6 (8.8)	70.2 (9.6)	72.6 (9.7)	74.2 (10.4)	3.0 (5.2)	4.0 (5.2)	.128	.156	.102
WHtr	0.50 (0.05)	0.51 (0.06)	0.49 (0.06)	0.51 (0.06)	−0.01 (0.04)	0.0 (0.04)	.206	.001	<.0001
BF %	30.2 (6.1)	30.0 (6.1)	26.8 (5.1)	28.5 (5.1)	−3.4 (7.8)	−1.5 (8.6)	.850	.003	.037
BMI, kg/m^2	21.5 (2.6)	21.7 (2.6)	21.9 (2.5)	22.6 (2.8)	0.4 (1.6)	0.9 (1.5)	.383	.011	.004
BAZ	2.0 (1.1)	2.0 (1.00)	1.6 (0.9)	2.0 (1.0)	−0.4 (0.7)	−0.1 (0.6)	.853	.001	<.0001
Dietary									
Fat, g/d	67.5 (29.4)	68.2 (29.5)	64.4 (28.6)	71.3 (29.8)	−3.1 (40.1)	3.1 (42.0)	.975	.033	.134
Energy intake, Kcal/d	1955.9 (461.5)	1900.8 (500.3)	1991.9 (457.8)	2025.1 (437.7)	35.9 (652.8)	124.3 (670.2)	.302	.504	.229
Iron intake, mg/d	19.0 (12.7)	20.9 (12.9)	22.7 (12.2)	21.6 (11.8)	3.8 (12.2)	0.7 (17.2)	.818	.427	.525
Physical activity									
AS	60.1 (22.9)	60.5 (28.1)	62.9 (24.8)	51.0 (25.9)	2.8 (28.3)	−9.5 (26.0)	.918	<.0001	<.0001
MET, met-h/d	6.2 (3.0)	6.0 (3.2)	7.5 (2.8)	6.1 (3.1)	1.2 (4.1)	0.1 (4.5)	.511	<.0001	.019

*Differences between intervention and control at baseline, by independent sample *t* test.
†Differences between intervention and control at follow-up, by independent *t* test.
‡Differences between intervention and control at the change from baseline to follow-up, by independent sample *t* test.
§χ^2 test.

Table V. Iron status and inflammatory markers in the intervention and control groups at baseline, follow-up, and the change from baseline to follow-up

	Baseline		Follow-up		Δ				
	Intervention, n = 160	Control, n = 166	Intervention, n = 160	Control, n = 166	Intervention, n = 160	Control, n = 166	P*	P†	P‡
Iron measurements									
Hb, g/dL	11.8 (1.3)	11.6 (1.4)	12.4(1.7)	12.1 (1.4)	0.6 (1.0)	0.5 (0.6)	.117	.037	.157
SI, μmol/L	19.7 (6.3)	20.0 (7.4)	21.5 (8.7)	20.1 (8.7)	1.8 (10.5)	0.04 (11.7)	.693	.134	.158
SF, ng/mL	43.8 (20.0)	43.9 (18.1)	40.8(17.6)	45.1 (18.2)	−3.0 (7.9)	1.2 (8.1)	.957	.030	<.0001
TF, mg/dL	257.1 (38.3)	256.7 (41.3)	244.4 (38.2)	261.2 (46. 6)	−12.7 (52.3)	4.4 (61.4)	.931	<.0001	.007
TS, %	31.3 (11.3)	31.9 (12.8)	36.0 (15.6)	31.5 (14.9)	4.7 (18.9)	−0.34 (19.9)	.692	.008	.022
sTfR, nmol/L	22.0 (3.7)	21.7 (3.0)	20.7 (4.1)	21.7 (4.1)	−1.3 (4.9)	−0.02 (4.4)	.470	.026	.013
Inflammation factors									
CRP, mg/mL	1.5 (0.8)	1.5 (0.8)	1.4 (0.8)	1.5 (0.8)	−0.1 (0.8)	0.02 (0.9)	.658	.033	.134
IL-6, pg/mL	3.8 (1.5)	3.9 (1.3)	3.5 (1.3)	4.0 (1.7)	−0.2 (1.5)	−0.1 (1.8)	.272	.004	.082

*Differences between intervention and control at baseline, by independent sample *t* test.
†Differences between intervention and control at follow-up, by independent *t* test.
‡Differences between intervention and control at the change from baseline to follow-up, by independent sample *t* test.

1.7 g/dL vs 12.1 ± 1.4 g/dL, $P = .037$ and 36.0 ± 15.6 vs 31.5 ± 14.9 $P = .008$, respectively). SF, TF, and sTfR were significantly lower in the intervention group than in the control group (40.8 ± 17.6 ng/mL vs 45.1 ± 18.2 ng/mL $P = .030$, 244.4 ± 38.2 mg/dL vs 261.2 ± 46.6 mg/dL $P < .0001$, and 20.7 ± 4.1 nmol/L vs 21.7 ± 4.1 nmol/L $P = .026$, respectively). Chronic inflammation was assessed by measuring CRP and IL-6 levels. The intervention group had reduced levels of CRP and IL-6 at follow-up. The levels of CRP and IL-6 in the intervention group were significantly lower than in the control group at follow-up (1.4 ± 0.8 mg/mL vs 1.5 ± 0.8 mg/mL $P = .033$ and 3.5 ± 1.3 pg/mL vs 4.0 ± 1.7 pg/mL $P = .004$, respectively).

The relationship between change values of BAZ, inflammatory markers, and iron measures were assessed by Pearson correlations (Table III). According to the Pearson correlations, ΔBAZ was significantly correlated with iron measures (ΔHb, $r = -0.17$, $P = .002$; ΔSF, $r = 0.21$, $P < .0001$; and ΔsTfR, $r = 0.27$, $P < .0001$) and inflammation markers (ΔCRP, $r = 0.23$, $P < .0001$ and ΔIL-6, $r = 0.18$, $P = .001$) and each of the correlations was still significant after Bonferroni correction (Table III). The correlations between ΔBAZ and ΔTF, ΔTs, and Δserum iron were not significant. ΔCRP was significantly correlated with ΔHb ($r = -0.14$, $P = .011$), ΔSF ($r = 0.24$, $P < .0001$), and ΔsTfR ($r = 0.18$, $P = .001$). ΔIL-6 was positively and significantly correlated with ΔSF ($r = 0.24$, $P < .0001$) and ΔsTfR ($r = 0.15$, $P = .002$). In Pearson correlation analyses, the correlations between ΔBF and ΔCRP, ΔBF and ΔIL-6, ΔBF and ΔBAZ, and ΔBF and Δiron measures were not significant. Because there were no significant correlations between ΔBF and changes of inflammatory and iron measures, ΔBF was not included in the MLRM. Although the correlations between ΔWHtr were significantly with ΔBAZ ($r = 0.527$, $P < .0001$), ΔCRP ($r = 0.277$, $P < .0001$), and ΔIL-6 ($r = 0.121$, $P = .029$). However, ΔWHtr was not a significant predictor in the 3 models of MLRM, so it was not included it in the MLRM.

MLRM were conducted to assess the effect of ΔBAZ and inflammatory markers (ΔCRP and ΔIL-6) from baseline to follow-up on iron measures (ΔSF, ΔsTfR, and ΔHb), which were correlated with ΔBAZ, ΔCRP, and ΔIL-6 (Table VI). The MLRM were adjusted for age, sex, and group. Because ΔIL-6 was not correlated with ΔHb in the Pearson correlation analyses and the correlation between ΔCPR and ΔHb was not significant after Bonferroni correction, we only included ΔBAZ in MLRM 1.

Discussion

The results of our study revealed that iron status was improved in the intervention group. Amato et al[13] found an increased iron absorption in obese children who underwent a 6-month weight-loss program but the iron measures did not significantly improve. However, in our study, we found a significantly improved iron profile. Hb, an indicator of ID anemia, increased in the intervention group and was significantly greater than in the control group at follow-up. SF levels, which represent total body iron stores[29] in the intervention group, were lower at follow-up than baseline. In addition, SF levels in the intervention group were significantly lower compared with those in the control group. However, this result does not indicate that the intervention group had lower iron body stores. It has been reported that SF was significantly greater in obese children,[30] and a reduction in SF levels reflects a reduction in inflammation.[31] To diagnose ID, sTfR levels are more sensitive and reliable than SF levels.[32] In the intervention group, sTfR levels were lower at follow-up than baseline and were significantly lower compared with those in the control group, which reflects the lower need of iron in tissue.[19] Recent work also found that SF and sTfR were greater in obese children compared with nonobese children.[33]

In the MLRM model 1, ΔBAZ was negatively correlated with ΔHb and positively correlated with ΔSF and ΔsTfR. These results suggest that a reduction in BAZ predicts an improvement in the iron status of overweight and obese children. The correlation between SF and BMI has been reported in young adults,[34] middle-aged women,[29] and in the third

Table VI. Multivariate linear regression analyses of ΔBAZ, ΔCRP, and ΔIL-6 on iron measurements (ΔSF, ΔsTfR, and ΔHb), adjusting for covariates

	Model 1		Model 2		Model 3	
	β (95%CI)	P	β (95%CI)	P	β (95%CI)	P
ΔSF						
ΔBAZ	3.52 (2.06-4.97)	<.0001	NA	NA	1.85 (0.23-3.44)	.022
ΔCRP	NA	NA	2.47 (1.36-3.58)	<.0001	1.89 (0.70-3.09)	.002
ΔIL-6	NA	NA	1.35 (0.78-1.92)	<.0001	1.22 (0.64-1.79)	<.0001
ΔsTfR						
ΔBAZ	2.27 (1.42-3.11)	<.0001	NA	NA	1.64 (0.68-2.59)	.001
ΔCRP	NA	NA	1.35 (0.68-2.03)	<.0001	0.88 (0.16-1.59)	.017
ΔIL-6	NA	NA	0.34 (−0.004 to −0.69)	.053	0.24 (−0.11 to −0.59)	.173
ΔHb						
ΔBAZ	−0.19 (−0.34 to 0.03)	.018	NA	NA	NA	NA

NA, not available.

National Health and Nutrition Examination Survey of the US.[35] The authors of a study on 100 postmenopausal women (50 obese and 50 normal weight)[36] and another study of 121 overweight children[7] reported that BMI values were independent predictors of sTfR.

The question that still remains: why is a reduction in BAZ associated with an improvement in the iron measures? We hypothesized that the improvement of inflammation may be involved.[37] Because obesity is considered to result in low-grade inflammation,[38] which stimulates hepcidin synthesis,[39] and, in turn, decreases iron absorption in intestinal in humans[40] and animals[41] and inhibit iron release from macrophagocyte,[42] this might be the mechanisms of ID in obese subjects.

We speculate that the improvement in iron status with weight loss may be due to the improvement of inflammation status. CRP and IL-6 were lower compared with the control group at follow-up, which suggests a reduction of inflammation in the intervention group. Our results suggest that a reduction in inflammation was associated with lower BAZ, lower WHtr, and increased physical activity. It is supported by the significant correlation between ΔBAZ, markers of inflammation (ΔCRP and ΔIL-6), ΔWHtr, and markers of inflammation (ΔCRP and ΔIL-6), and additionally, the significant increase in MET in the intervention group from baseline to follow-up. The increased physical activity may have reduced inflammation, which has been reported by previous studies.[28,30] In our study, physical activity was guided and supervised by trained teachers.

In the MLRM model 2, the correlations between Δ inflammation markers (ΔCRP and ΔIL-6) and Δ iron indicators (ΔSF and ΔsTfR) were significant. The correlations remained significant in MLRM model 3 after further adjustment of ΔBAZ in the model compared with model 2, which suggests that low levels of inflammatory markers predict an improvement in the iron status of children (SF and sTfR) independent of ΔBAZ.

Limitations of our study included the lack of hepcidin measurements, the lack of iron absorption measurements and the lack of Pubertal Stage assessment. Another limitation was that we did not select a criterion measure of body adiposity in this NISCOC program. The significant correlation between change in inflammation and change in iron status, independent of the change in adiposity should be interpreted with caution.

The iron status and inflammation in overweight and obese children were improved with weight management. The improvements in inflammatory markers during weight reduction are independently associated with improvements of iron status. ■

We thank local the China CDC for the help of delivering the intervention and collecting data. We appreciate the support and dedication of the 6 schools, teachers, food services, and all subjects who were involved in this study.

Submitted for publication Jul 29, 2013; last revision received Nov 27, 2013; accepted Dec 3, 2013.

Reprint requests: Ying Li, PhD, Departments of Nutrition and Food Hygiene, School of Public Health, Harbin Medical University, No. 157 Baojian Road, Harbin, 150081, China. E-mail: liying_helen@163.com

References

1. Schneider JM, Fujii ML, Lamp CL, Lonnerdal B, Dewey KG, Zidenberg-Cherr S. Anemia, iron deficiency, and iron deficiency anemia in 12-36-mo-old children from low-income families. Am J Clin Nutr 2005;82:1269-75.
2. McLean E, Cogswell M, Egli I, Wojdyla D, de Benoist B. Worldwide prevalence of anaemia, WHO Vitamin and Mineral Nutrition Information System, 1993-2005. Public Health Nutr 2009;12:444-54.
3. Baltussen R, Knai C, Sharan M. Iron fortification and iron supplementation are cost-effective interventions to reduce iron deficiency in four subregions of the world. J Nutr 2004;134:2678-84.
4. Harris RJ. Nutrition in the 21st century: what is going wrong. Arch Dis Child 2004;89:154-8.
5. Nead KG, Halterman JS, Kaczorowski JM, Auinger P, Weitzman M. Overweight children and adolescents: a risk group for iron deficiency. Pediatrics 2004;114:104-8.
6. Zimmermann MB, Zeder C, Muthayya S, Winichagoon P, Chaouki N, Aeberli I, et al. Adiposity in women and children from transition countries predicts decreased iron absorption, iron deficiency and a reduced response to iron fortification. Int J Obes 2008;32:1098-104.
7. Aeberli I, Hurrell RF, Zimmermann MB. Overweight children have higher circulating hepcidin concentrations and lower iron status but have dietary iron intakes and bioavailability comparable with normal weight children. Int J Obes 2009;33:1111-7.

8. Pinhas-Hamiel O, Newfield RS, Koren I, Agmon A, Lilos P, Phillip M. Greater prevalence of iron deficiency in overweight and obese children and adolescents. Int J Obes Rel Metab Disord 2003;27:416-8.
9. Newman BH. Vasovagal reaction rates and body weight: findings in high- and low-risk populations. Transfusion 2003;43:1084-8.
10. Menzie CM, Yanoff LB, Denkinger BI, McHugh T, Sebring NG, Calis KA, et al. Obesity-related hypoferremia is not explained by differences in reported intake of heme and nonheme iron or intake of dietary factors that can affect iron absorption. J Am Diet Assoc 2008;108:145-8.
11. Baumgartner J, Smuts CM, Aeberli I, Malan L, Tjalsma H, Zimmermann MB. Overweight impairs efficacy of iron supplementation in iron-deficient South African children: a randomized controlled intervention. Int J Obes 2013;37:24-30.
12. Tussing-Humphreys LM, Nemeth E, Fantuzzi G, Freels S, Holterman AX, Galvani C, et al. Decreased serum hepcidin and improved functional iron status 6 months after restrictive bariatric surgery. Obesity 2010;18:2010-6.
13. Amato A, Santoro N, Calabro P, Grandone A, Swinkels DW, Perrone L, et al. Effect of body mass index reduction on serum hepcidin levels and iron status in obese children. Int J Obes 2010;34:1772-4.
14. Li Y, Hu X, Zhang Q, Liu A, Fang H, Hao L, et al. The nutrition-based comprehensive intervention study on childhood obesity in China (NISCOC): a randomised cluster controlled trial. BMC Public Health 2010;10:229.
15. De Onis M , World Health Organization, Department of Nutrition for Health and Development. WHO child growth standards: length/height-for-age, weight-for-age, weight-for-length, weight -for-height and body mass index-for-age: methods and development. Geneva: World Health Organization; 2006.
16. Ainsworth BE, Haskell WL, Herrmann SD, Meckes N, Bassett DR Jr, Tudor-Locke C, et al. 2011 Compendium of Physical Activities: a second update of codes and MET values. Med Sci Sports Exer 2011;43:1575-81.
17. Liu AL, Ma GS, Zhang Q, Ma WJ. Reliability and validity of a 7-day physical activity questionnaire for elementary students [in Chinese]. Zhonghua Liu Xing Bing Xue Za Zhi 2003;24:901-4.
18. Andrews NC, Schmidt PJ. Iron homeostasis. Ann Rev Physiol 2007;69:69-85.
19. Cook JD, Skikne BS, Baynes RD. Serum transferrin receptor. Ann Rev Med 1993;44:63-74.
20. von Bonsdorff L, Lindeberg E, Sahlstedt L, Lehto J, Parkkinen J. Bleomycin-detectable iron assay for non-transferrin-bound iron in hematologic malignancies. Clin Chem 2002;48:307-14.
21. World Health Organization. Assessing the iron status of populations: including literature reviews: report of a Joint World Health Organization/Centers for Disease Control and Prevention Technical Consultation on the Assessment of Iron Status at the Population Level. 2004:108. http://www.who.int/nutrition/publications/micronutrients/anaemia_iron_deficiency/9789241596107/en/. Accessed December 11, 2103.
22. Nemeth E, Rivera S, Gabayan V, Keller C, Taudorf S, Pedersen BK, et al. IL-6 mediates hypoferremia of inflammation by inducing the synthesis of the iron regulatory hormone hepcidin. J Clin Invest 2004;113:1271-6.
23. de Onis M, Onyango AW, Borghi E, Siyam A, Nishida C, Siekmann J. Development of a WHO growth reference for school-aged children and adolescents. Bull World Health Org 2007;85:660-7.
24. World Health Organization. Office of the Spokesperson. WHO/CDC expert consultation agrees on best indicators to assess iron deficiency, a major cause of anaemia. Geneva: World Health Organization; 2004.
25. Beguin Y. Soluble transferrin receptor for the evaluation of erythropoiesis and iron status. Clin Chim Acta 2003;329:9-22.
26. del Giudice EM, Santoro N, Amato A, Brienza C, Calabro P, Wiegerinck ET, et al. Hepcidin in obese children as a potential mediator of the association between obesity and iron deficiency. J Clin Endocrinol Metab 2009;94:5102-7.
27. Siegrist M, Hanssen H, Lammel C, Haller B, Halle M. A cluster randomised school-based lifestyle intervention programme for the prevention of childhood obesity and related early cardiovascular disease (JuvenTUM 3). BMC Public Health 2011;11:258.
28. Rosenbaum M, Nonas C, Weil R, Horlick M, Fennoy I, Vargas I, et al. School-based intervention acutely improves insulin sensitivity and decreases inflammatory markers and body fatness in junior high school students. J Clin Endocrinol Metab 2007;92:504-8.
29. Cade JE, Moreton JA, O'Hara B, Greenwood DC, Moor J, Burley VJ, et al. Diet and genetic factors associated with iron status in middle-aged women. Am J Clin Nutr 2005;82:813-20.
30. Halle M, Korsten-Reck U, Wolfarth B, Berg A. Low-grade systemic inflammation in overweight children: impact of physical fitness. Exerc Immunol Rev 2004;10:66-74.
31. Yanoff LB, Menzie CM, Denkinger B, Sebring NG, McHugh T, Remaley AT, et al. Inflammation and iron deficiency in the hypoferremia of obesity. Int J Obes 2007;31:1412-9.
32. Punnonen K, Irjala K, Rajamaki A. Serum transferrin receptor and its ratio to serum ferritin in the diagnosis of iron deficiency. Blood 1997;89:1052-7.
33. Hamza RT, Hamed AI, Kharshoum RR. Iron homeostasis and serum hepcidin-25 levels in obese children and adolescents: relation to body mass index. Hormone Res Paediatr 2013;80:11-7.
34. Williams MJ, Poulton R, Williams S. Relationship of serum ferritin with cardiovascular risk factors and inflammation in young men and women. Atherosclerosis 2002;165:179-84.
35. Ausk KJ, Ioannou GN. Is obesity associated with anemia of chronic disease? A population-based study. Obesity 2008;16:2356-61.
36. Lecube A, Carrera A, Losada E, Hernandez C, Simo R, Mesa J. Iron deficiency in obese postmenopausal women. Obesity 2006;14:1724-30.
37. Cepeda-Lopez AC, Osendarp SJ, Melse-Boonstra A, Aeberli I, Gonzalez-Salazar F, Feskens E, et al. Sharply higher rates of iron deficiency in obese Mexican women and children are predicted by obesity-related inflammation rather than by differences in dietary iron intake. Am J Clin Nutr 2011;93:975-83.
38. Dandona P, Aljada A, Bandyopadhyay A. Inflammation: the link between insulin resistance, obesity and diabetes. Trends Immunol 2004;25:4-7.
39. Nemeth E, Valore EV, Territo M, Schiller G, Lichtenstein A, Ganz T. Hepcidin, a putative mediator of anemia of inflammation, is a type II acute-phase protein. Blood 2003;101:2461-3.
40. Ruivard M, Laine F, Ganz T, Olbina G, Westerman M, Nemeth E, et al. Iron absorption in dysmetabolic iron overload syndrome is decreased and correlates with increased plasma hepcidin. J Hepatol 2009;50:1219-25.
41. Laftah AH, Ramesh B, Simpson RJ, Solanky N, Bahram S, Schumann K, et al. Effect of hepcidin on intestinal iron absorption in mice. Blood 2004;103:3940-4.
42. Nemeth E, Tuttle MS, Powelson J, Vaughn MB, Donovan A, Ward DM, et al. Hepcidin regulates cellular iron efflux by binding to ferroportin and inducing its internalization. Science 2004;306:2090-3.

Table II. Description of outcomes

	Outcomes (instruments or methods)	Assess for
Body composition	Weight (RGT-140; Weighing Apparatus Co. Ltd. Changzhou Wujin, China); Waist circumference; BF (ImpDF50; Impedimed Pty Ltd., Queensland, Australia); BMI, BAZ, and WHtr were calculated.	Reflects the body composition
Diet	Fat, energy, and iron intake (3–day, 24-hour dietary recall).	Reflects the dietary intake situations
Physical activity and physical fitness	MET was calculated by 2011 Compendium of Physical Activities.[16] (physical activities were collected by a validated 7-day physical activity questionnaire[17]).	Reflects the energy cost of physical activities
	AS was calculated based on the 3 physical fitness tests.	The average physical fitness level
	Standing long jump	Muscle explosive strength
	50-m sprint	Movement speed
	50 m * 8 shuttle run	Cardiorespiratory fitness
Iron status	SI (AA-660 flame atomic absorption spectrophotometer; Shimadzu, Kyoto, Japan).	The amount of circulating iron
	SF (ELISA kits; RayBio, Norcross, Georgia)	Indicator of iron storage[18]
	TF (ELISA kits; Abcam, Cambridge, United Kingdom)	Binds the iron absorbed by intestinal[18]
	sTfR (Quantikine IVD immunoassay, R&D Systems, Minneapolis, Minnesota)	Reflects reliably the degree of the tissue iron supply[19]
	Hb (ELISA kits, Bethyl Laboratories Montgomery, Texas)	Used as indicators of the ID recommended by WHO/China CDC expert consultation
	TS was calculated = [SI (μmol/L)/serum TF (g/L)] $\times$ 3.98[20]	Reflects the amount of iron bound to TF
Inflammation markers	CRP (ELISA kits; Biocheck Inc, Foster City, California)	Quickly respond to inflammation[21]
	IL-6 (ELISA kits; R&D System Europe, Abingdon, United Kingdom)	Induces transcription of hepcidin gene in hepatocytes[22]

AS, average scores of the 3 physical fitness tests; *ELISA*, enzyme-linked immunosorbent assays; *SI*, serum iron; *TS*, transferrin saturation.
BMI was calculated from the weight (kg) divided by height (m^2). BAZ was calculated using the WHO 2007 growth references for children in the 5- to 19-year age group.[23]
SF and Hb are the most useful indicators of the ID according to WHO/China CDC expert consultation.[24] Because ferritin will be affected by inflammation, sTfR is also recommended by the consultation and clinical study.[25]
SI, TF, and TS were selected according to previous study.[26] CRP and IL-6 also were referred to the former studies[27,28] as indicators of inflammation.

Table III. Pearson correlation between Δiron measurements (SF, sTfR, Hb, TF, SI, and TS%) and ΔBAZ, Δ iron measurements, and ΔCRP, and Δiron measurements and ΔIL-6

	SF	sTfR	Hb	TF	SI	Ts%
BAZ	0.21*	0.27*	−0.17*	0.04	−0.14	−0.17
CRP	0.24*	0.18*	−0.14	0.03	−0.004	−0.02
IL-6	0.24*	0.15*	−0.11	0.02	0.05	0.01

The correlations were performed separately (ie, SF with BAZ, SF with CRP, and SF with IL-6; each iron parameter done 3 times of Pearson correlations).
*Significant differences at a Bonferroni-corrected *P* value of $<.05/18 \sim .003$.

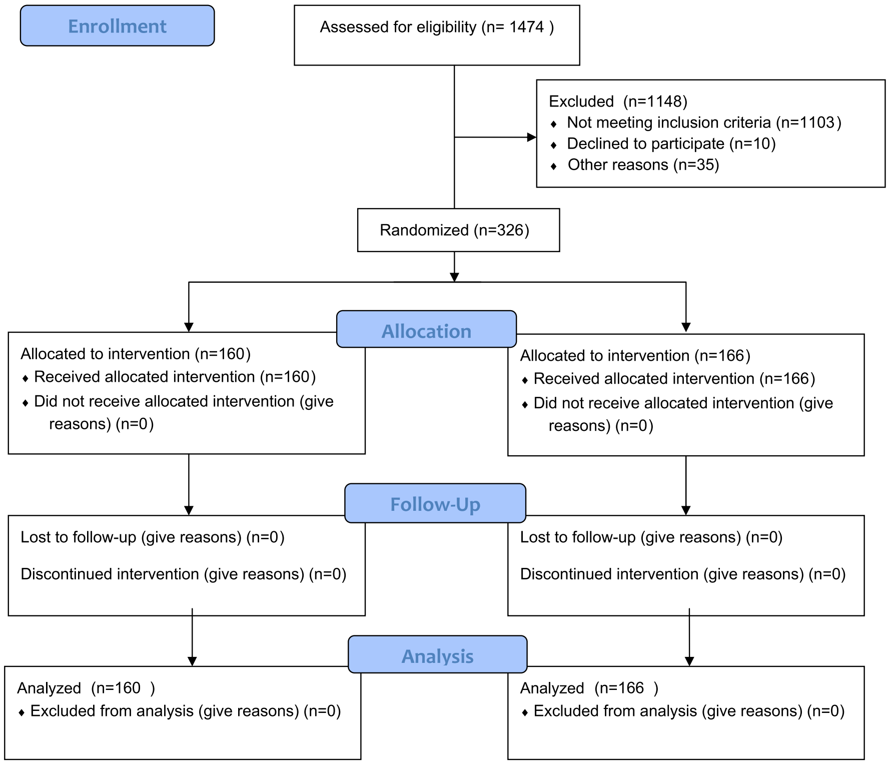

Figure 1. CONSORT 2010 flow diagram.

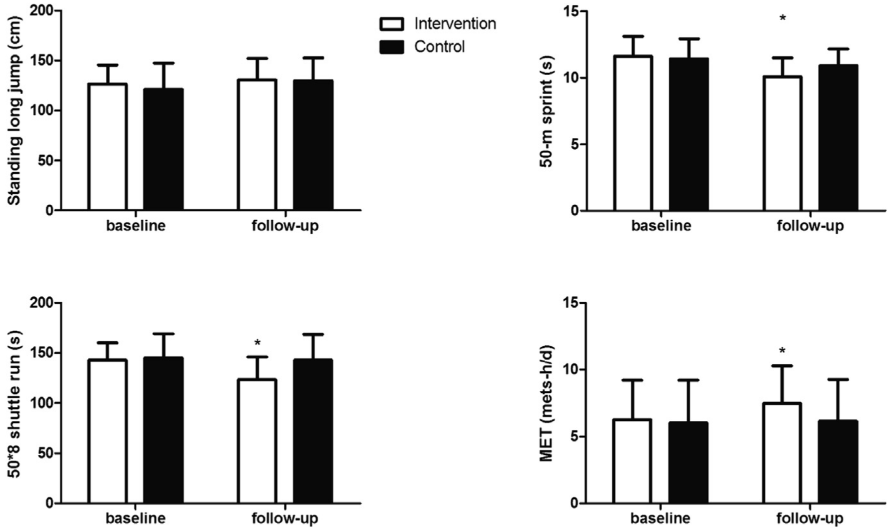

Figure 2. The result of 3 physical fitness tests and physical activity at baseline and follow-up. Significant differences are denoted by an asterisk at a $P < .0001$.
