# Supplementary material for: Systematic review and meta-analysis of school-based obesity interventions in mainland China
Source: PLoS One. 2017 Sep 14;12(9):e0184704. doi: 10.1371/journal.pone.0184704 (PMC5598996; doi:10.1371/journal.pone.0184704)
Supplement: S1 Dataset — (ZIP) [file pone.0184704.s007.zip › S1_dataset/76库/30.pdf]

Obesity Research & Clinical Practice (2015) xxx, xxx.e1—xxx.e8

ELSEVIER

ORIGINAL ARTICLE

Intervention of childhood and adolescents obesity in Shantou city☆

Haiting Guo[a,b], **Xiaoshan Zeng**[b,c], **Qiaoyu Zhuang**[a], **Yubin Zheng**[b,d], **Shenren Chen**[a,*]

[a] *Department of Endocrinology, The 2nd Affiliated Hospital of SUMC, Shantou, Guangdong Province 515043, China*
[b] *Shantou University Medical College, Shantou, Guangdong Province 515043, China*
[c] *Shantou Central Hospital, Shantou, Guangdong Province 515043, China*
[d] *Yue Bei People's Hospital, Shaoguan, Guangdong Province 512026, China*

Received 16 June 2014; received in revised form 12 November 2014; accepted 28 November 2014

KEYWORDS
Children;
Adolescents;
Obesity;
Intervention

Summary
Objectives: The purpose of this study was to evaluate the effects of multicomponent school based intervention constituted of diet modification, regular exercise and psychosocial consultation on body status in overweight and obese children and adolescents. And to come up with an appropriate intervention protocol for controlling children and adolescents obesity in Shantou city.
Methods: Two schools were randomised to intervention group and control group respectively. A total of 41 students enrolled were diagnosed as overweight or obese. Twenty-six students of the intervention group completed the one-year intervention programme consisted of diet modification, regular exercise and psychological consultation except two of them were transferred to another school. The differences of BMI, anthropometric measures, metabolic profile and the scores of questionnaire and the scale were compared to evaluate the effects of the intervention programme.
Results: After one-year intervention, it observed in the intervention group that BMI-Z score, WHR and WHtR had significant improvements, and there was a nonsignificant trend ($P = 0.053$) for a decrease in BMI-P. Fasting plasma glucose, cholesterol (CH) and low-density-lipoprotein cholesterol (LDL-C) levels in the intervention group showed nonsignificant trend for a decrease (PFPG = 0.084, PCH = 0.057, PLDLC = 0.098), compared with a significant increase of triglycerides (TG) and LDL-C levels in the control group (PTG = 0.041, PLDL-C = 0.038). There were some positive dietary, physical activity, or sedentary behaviour changes found in the students of the intervention group as the scores of the questionnaire got significant improvement ($P = 0.04$).

☆ *Financial support*: This study was supported by the 2012 Shantou Medical Science and Technology Project.
* Corresponding author at: Department of Endocrinology, The 2nd Affiliated Hospital of Shantou University Medical College, Shantou, Guangdong Province 515043, China. Tel.: +86 075488915836; fax: +86 0754 88346543.
E-mail address: chen-shenren@163.com (S. Chen).

http://dx.doi.org/10.1016/j.orcp.2014.11.006
1871-403X/© 2014 Asian Oceanian Association for the Study of Obesity. Published by Elsevier Ltd. All rights reserved.

Please cite this article in press as: Guo H, et al. Intervention of childhood and adolescents obesity in Shantou city. Obes Res Clin Pract (2015), http://dx.doi.org/10.1016/j.orcp.2014.11.006

Conclusions: Our one-year multicomponent school-based intervention programme did have positive effects to some extents on health state and lifestyle behaviour of overweight and obese children and adolescents, which indicated that it is feasible and important to implement such a school-based intervention programme in Shantou city.
© 2014 Asian Oceanian Association for the Study of Obesity. Published by Elsevier Ltd. All rights reserved.

Data from World Health Organization (WHO) indicated that obesity has become epidemic all over the world. Between 1980 and 2008, the prevalence of obesity has almost doubled. Moreover, there is a tendency that obesity is harassing younger people and spreading to low- and middle-income countries [1]. The childhood and adolescent obesity epidemic has become one of the most urgent global health challenges of the 21st century. Recently, China has also been significantly affected [2]. Figures form the Chinese national of the nutrition and health survey in 2002 showed the prevalence of childhood overweight and obesity was 4.5% and 2.1% respectively [3]. The average prevalence of overweight and obesity in Shantou among students aged 6—18 years from 1997 to 2006 was 6.85% and 6.20%, respectively, with an increasing trend [4].

Different countries worldwide are facing not only the problem of globally rising obesity prevalence, but also chronic diseases associated with obesity, such as hypertension, type 2 diabetes, dyslipidemia, obstructive sleep apnoea and respiratory problems, osteoarthritis and cancer [1]. Based on the figure of WHO, at least 2.8 million people died of overweight or obesity each year, which kill more people than malnutrition and increase the medical burden of diabetes, coronary heart disease and cancer. Overweight and obese children are likely to become obese adults and are at higher risks for developing chronic non-infectious diseases [5]. Despite the rigorous obesity prevalence, overweight and obesity, as well as their related diseases, are largely preventable. Recent years, many large-scale multicomponent intervention of children and adolescents obesity are conducted all over the world, which show lifestyle intervention have some assignable effects. Results of a meta-analysis [6] and some school-based health diet and physical activity promotion interventions [7—9] reveal the intervention is feasible and effective. They are beneficial to improve the physical activity, lipid profile and body mass index (BMI). However, most intervention programmes have been conducted in Western countries, thus have limited generalisability to the Chinese context due to the cultural differences. A Happy 10 programme conducted by Chinese CDC and International Life Sciences Institute Focal Point-China on the promotion of physical activity has a great influence in China, and has positive effects in some areas [10]. Although there are many successful examples, the childhood and adolescents obesity intervention research still has a long way to go in Shantou city with regional culture.

Base on the eating habits and culture characteristics of Chaoshan area, in order to control the epidemic of childhood obesity and reduce the occurrence of adults obesity and the mortality of the related chronic diseases, we implement a school-based childhood and adolescents obesity intervention programme to evaluate the effects on body status and reducing the risk of having chronic diseases. We hope to come up with an appropriate and feasible intervention protocol for controlling children and adolescents obesity in Shantou city.

Sampling and population

Two similar schools from two districts of Shantou, China were randomised, one as an intervention school and the other as a control school. The BMI of all the students in grades 3—5 of the two schools were calculated. Based on BMI-For-Age Growth Charts and the table of cut-off points of the charts, adjusted by the Centers for Disease Control Prevention (CDC), participants were divided into four groups: malnourished, normal weight, overweight, and obese [11]. A total of 41 overweight or obese students (25 boys, 16 girls) and their parents were included in the intervention programme; 26 from the intervention school and 15 from the control school. Students with malnutrition, students in grades 1, 2 and 6, secondary obesity students and students who used to have diseases that are known to affect habitus, insulin sensitivity, physical activity and diet intake were excluded.

Please cite this article in press as: Guo H, et al. Intervention of childhood and adolescents obesity in Shantou city. Obes Res Clin Pract (2015), http://dx.doi.org/10.1016/j.orcp.2014.11.006

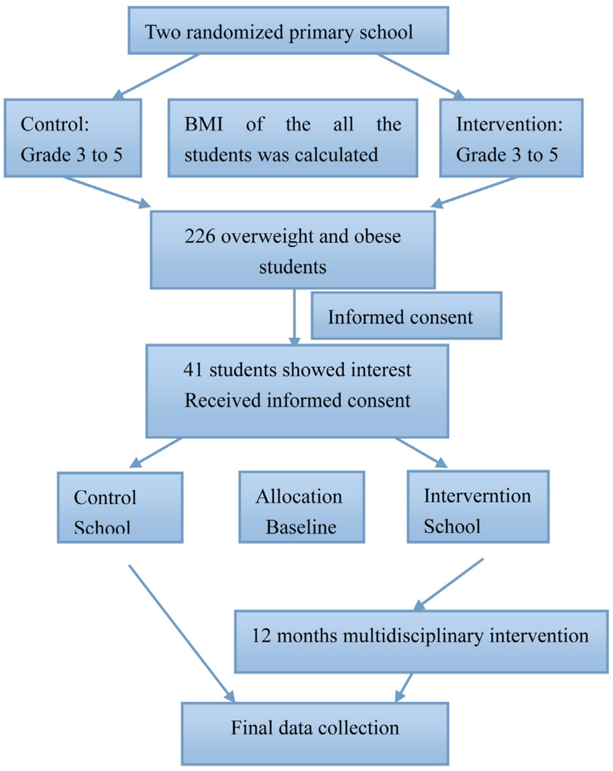

Figure 1 Flow chart of enrolment.

Informed consent

All the physical examination and the questionnaire survey were completed after the signature of informed consent. The study protocol conformed to the ethical guidelines of the 1975 Declaration of Helsinki as reflected in *a priori* approval by the review committee of Shantou University Medical College. The study was approved by the Shantou Ethics Review Committee and informed consent was obtained from all participants (Fig. 1).

Personnel training

Before the conduction of the intervention trial, all the investigators had systemic training. All the staffs clearly known the procedure, method and guidelines of the intervention.

Intervention programme

Psychological fitness was established to implement 12-month education and consultation of diet, exercise and psychology to the intervention school of this project.

Nutritional education session

An hour long nutrition education lectures were given to intervention school students monthly, which were consisted of 40 min of teaching and 20 min of interaction including questions asking and homework assignments. After the lectures, brochures designed according to Chinese residents dietary guidelines were dispense to disseminate the knowledge of healthy diet. The general principles of diet intervention are intake of the least amount of calories and fat and getting the maximum nutritional value.

Exercise session

According to the advice of WHO, all the intervention school students were guided to have moderate to vigorous activity for at least 60 min with most of which is aerobic exercise [12]. Students took part in a 1-h session of exercise and activities with the intent to help establishing regular, safe and effective exercise pattern so as to work out a suitable plan. Students were motivated to exercise at 70—80% of their maximal heart rate.

Psychological intervention session

An hour long psychological health education and consultation lectures were given to intervention school students monthly. The educations and consultations were given by psychiatrist. Based on the all the characteristics of the children and adolescents mental development, all the lectures were guided by Social Cognitive Theory (SCT) [13], the most influential healthy behaviour changing theory.

Fun activity session

During summer and winter vacation, there were 1—2 outdoor activities held in contesting form.

Telephone follow-up

Telephone follow-up was conducted once every two weeks during the intervention trial.

End points measures

All children of the two schools underwent a general physical exam. A full set of anthropometric and body composition measurements and blood test were collected at baseline and after 12-month follow-up. The primary endpoints were nutritional

status indicators, which included height, weight, BMI, BMI-P, and BMI-Z. The secondary endpoints were other body composition measurements, laboratory indicators and the score of the questionnaire and Piers-Harris Child's Self-concept Scale.

Height, weight and BMI

Height and weight were measured using standard weighing scales while the participants were standing straight and wearing light clothing and no shoes. Their BMI was also calculated (weight in kilograms divided by the square of the height in metres). The age- and sex-related percentile (BMI-P) and Z-score (BMI-Z) were calculated using online professional software (http://stokes.chop.edu/web/zscore/) according to BMI.

Other measurements (WC, HC, WHR, WHtR)

All the measurements were performed solely to ensure the protection of the students' privacy. Students stood erect with both arms prolapsed naturally to the body and both feet together. The waist circumference (WC) was measured over the naked skin at the middle point between the top of the hipbone and the bottom of the rib cage, while hip circumference (HC) was measured over the naked skin at the widest part of the hip. Two measurements were obtained, and the average was recorded. Waist-to-hip ratio (WHR) and waist-to-height ratio (WHtR) were calculated as WC (cm)/HC (cm) and WC (cm)/height (cm), respectively.

Laboratory blood test

An over night fasting blood samples was taken to screen impaired glucose regulation (IGR) and dyslipidemia in two weeks after anthropometric measures were conducted. Laboratory tests included tests to determine the levels of fasting plasma glucose (FPG), cholesterol (CH), triglycerides (TG), low-density-lipoprotein cholesterol (LDL-C), and high-density-lipoprotein cholesterol (HDL-C). All the samples were analysed by the Second Affiliated Hospital of Shantou University Medical College with strict quality control.

No students of IGR or dyslipidemia were found in the intervention school.

Questionnaire

Based on the international scales of obesity management [14,15], a self-assessment matching questionnaire, including separate sections for children and their parents, was designed to evaluate the risk factors for obesity. Reliability and validity of the questionnaire were estimated before the survey. The questionnaire was composed of 8 parts. (Appendix 1)

Students of both schools have finished the questionnaire and the information of the diet and exercise habits of the students was collected.

Self-concept scale

Drew the reference of the research results of the children and adolescents' psychological characteristics, Piers-Harris Child's Self-concept Scale was used to evaluate the self awareness of the students form two school at baseline and 12-month follow-up.

Quality control and statistics analysis

Strict quality control was conducted during all steps of the study. Results were presented as mean ± standard deviation (SD). Variables were analysed using *t*-test, Fisher's exact test, and chi-square test. $P < 0.05$ was considered statistically significant, and $P < 0.01$ was considered remarkably statistically significant. All statistical analyses were performed using a statistical analysis system software (SAS 17.0 version).

Drop-out rate and compliance

Two of the 26 students of the intervention school could not finish the whole trial because of transferring to another school and one students did not have physical examination and blood test but filling out the questionnaire and self-concept scale. There was an 11.6% drop-out rate of the intervention school and the compliance was 88.5%. The drop-out rate of the control school was 13.3%, with two students transferred to another school and could not finished the postintervention measurements and blood test. The intervention school had a compliance of 86.7%.

Baseline comparison

There was no significant difference of gender, age, BMI-Z, BMI-P, WC and other laboratory indicators between the intervention school and the control school at baseline except we found higher level of TG and LDL-C in the intervention school.

Please cite this article in press as: Guo H, et al. Intervention of childhood and adolescents obesity in Shantou city. Obes Res Clin Pract (2015), http://dx.doi.org/10.1016/j.orcp.2014.11.006

Table 1 Self-comparison of anthropometric characteristics and laboratory values at baseline (mean ± SD).

Variables	Intervention school			Control school		
	Baseline	Postintervention	*P*-value[a]	Baseline	12-Month follow-up	*P*-value[a]
Height	140.67 ± 7.18	147.81 ± 7.19	0.000	142.08 ± 9.04	147.06 ± 8.36	0.000
Weight	44.13 ± 7.27	50.35 ± 8.18	0.000	44.88 ± 8.49	50.51 ± 8.95	0.000
BMI	22.18 ± 2.13	22.95 ± 2.53	0.002	22.05 ± 2.57	23.16 ± 2.39	0.013
BMI-Z	1.56 ± 0.33	1.47 ± 0.44	0.036	1.45 ± 0.35	1.48 ± 0.31	0.617
BMI-P	93.04 ± 4.04	91.13 ± 7.40	0.053	91.62 ± 4.31	92.15 ± 4.49	0.587
WC	76.39 ± 7.24	77.77 ± 7.24	0.182	75.35 ± 8.01	77.6 ± 6.82	0.003
HC	81.23 ± 5.27	85.40 ± 5.82	0.000	80.58 ± 7.73	86.73 ± 7.00	0.000
WHR	0.94 ± 0.045	0.91 ± 0.054	0.006	0.94 ± 0.07	0.89 ± 0.04	0.008
WHtR	0.54 ± 0.044	0.53 ± 0.047	0.030	0.53 ± 0.04	0.53 ± 0.04	0.612
FPG	5.08 ± 0.31	4.92 ± 0.40	0.084	5.19 ± 0.63	5.06 ± 0.59	0.472
TG	1.32 ± 0.98	1.51 ± 0.86	0.294	0.84 ± 0.27	1.31 ± 0.56	0.041
CH	4.70 ± 0.76	4.50 ± 0.70	0.057	4.55 ± 0.57	5.00 ± 1.20	0.174
HDL-C	1.29 ± 0.30	1.34 ± 0.27	0.218	1.14 ± 0.17	1.11 ± 0.27	0.692
LDL-C	2.90 ± 0.45	2.74 ± 0.48	0.098	2.41 ± 0.31	3.02 ± 0.87	0.038
Questionnaire score	93.61 ± 16.66	99.96 ± 9.86	0.040	96.0 ± 14.50	96.23 ± 12.60	0.944
Score of the self scale	50.51 ± 9.45	52.58 ± 8.57	0.283	49.85 ± 10.05	50.46 ± 10.43	0.821

Abbreviations: BMI, body mass index; BMI-Z, BMI Z score; BMI-P, BMI percentile; WC, waist circumference; HC, hip circumference; WHR, waist-to-hip ratio; WHtR, waist-to-height ratio; FPG, fasting plasma glucose; CH, cholesterol; TG, triglycerides; HDL-C, high-density-lipoprotein cholesterol; LDL-C, low-density-lipoprotein cholesterol.

[a] *t*-Test for equality of means.

Intervention results

In regards to the effect of the intervention on nutritional status indicators, our data showed some apparent changes in some parameters tested (Table 1). The intervention school had significant ($P < 0.05$) changes between pre- and postintervention values, such as height, weight, BMI, BMI-Z, HC, WHR and WHtR. Among them, BMI-Z, WHR and WHtR had significant improvement after 12-month intervention, and there was a nonsignificant trend ($P = 0.053$) for a decrease in pre- *vs.* postintervention values for BMI-P. In the control school, we noticed significant ($P < 0.05$) changes between baseline and 12-month follow-up values are as follows: height, weight, BMI, WC, HC and WHR. However, all the above variables did not show any improvements.

Pre- and postintervention data for the laboratory blood indicators are also presented in Table 1. There were no significant differences between pre- and postintervention values for TG and HDL-C ($P > 0.005$), with a nonsignificant decline trend for FPG ($P = 0.084$), CH ($P = 0.057$) and LDL-C ($P = 0.098$). However, we found that the intervention school had a significant increase of the one-year follow-up values for TG ($P = 0.0041$) and LDL-C ($P = 0.0038$), while there were no significant difference for the other laboratory blood indicators ($P > 0.005$).

As can be seen in Table 1, the intervention school students had significant improvement ($P = 0.04$) in the questionnaire score, while there was no significant difference between baseline and 12-month follow-up value for the questionnaire score of the control school. Both school had no significant difference for the score of the Piers-Harris Child's Self-concept Scale ($P > 0.05$).

Discussion

So far, BMI was still widely used as an important criterion of nutritional status. However, BMI alone was not enough to well predict obesity and its related diseases risk and mortality [16], thus, the additional use of other anthropometric parameters, which indicate body fat distribution, such as WC, HC, WHR, WHtR and skinfold is recommended [16].

We noticed significant increase of BMI in the two schools instead of decrease of BMI, however, this was not an unexpected result, in the view of the results observed in previous study [17]. A recent meta-analysis has shown that lifestyle interventions could have limited effects in reducing weight [18]. Children and adolescents are at the peak period of growth and development and it is likely to mislead the study results analysis when using BMI to assess body status when considering the ratio of muscle mass and the amount of fat. There were studies

suggested BMI-Z to be a more reliable parameter [19], which was calculated according to the median and the standard deviation of age- and sex-specific reference values. Besides, with consideration of the effect of the normal growth and development of children and adolescents, BMI-Z score could better monitor the body fat percentage of the school-age children [20]. The intervention school had significant ($P < 0.05$) changes between baseline and postintervention values for BMI-Z and there was a nonsignificant trend for a decline for BMI-P. Thus, lifestyle intervention could be beneficial to improve the nutritional status of the overweigh and obesity students.

It has witnessed an increase values for HC and no declination of WC after 12-month follow-up. This phenomenon may due to the growing of bone and muscle mass when students being in the stage of puberty, which could be supported by the improvement of postintervention values for WHR and WHtR of the intervention school. Some studies have noticed the significant decrease of WC of the overweigh and obesity children after 10-week and 12-week intervention [21,22]. In our intervention school, there was no significant promotion of postintervention values for WC, but the intervention programme stabilise the growth of WC when compared to the control school. WC is closely related to central obesity and cardiovascular diseases and could be used to evaluate the amount of visceral fat. Decrease of WC means the reduction of visceral fat. However, the measurement of WC was affected by the amount of muscle. So, some studies recommended magnetic resonance imaging (MRI) to evaluate the distribution of visceral fat more precisely [23]. Another central obesity related parameter, WHtR, was found to be better predict mortality of obesity and its related chronic diseases [16]. Therefore, it is possible that the students in this study reduced the risk of cardiovascular and other chronic noninfectious diseases by carrying on the lifestyle intervention.

Compared to the normal weight peers, all the obese children had a significantly higher risk of developing chronic diseases [24,25]. If a child' obesity status tracks into puberty, the risk of being obese as an adult is greater [9]. Blood pressure, FPG, WC and blood lipid are metabolic syndrome related parameters, and also the risk factors of cardiovascular and other chronic diseases. There was no significant improvement of the lipid profile after the intervention, but we observed a nonsignificant trend (PCH = 0.057, PLDL-C = 0.098) for CH and LDL-C levels to be decreased after the intervention in the intervention school. In contrast, the control school had significant increase of the 12-month follow-up values for TG and LDL-C levels (PTG = 0.041, PLDL-C = 0.038). Therefore, the control school students could have greater risk of developing chronic diseases, which was likely to be reduced after the intervention. For the FPG values, none of the children had elevated FPG levels indicative of impaired glucose regulation. Nevertheless, the intervention school had a nonsignificant decline trend of the postintervention values for FPG, which was similar to that of Farris' study [9]. We did not measure insulin sensitivity, but other studies have found that insulin sensitivity has significantly improvement even if FPG levels exhibit little or no change after exercise interventions in children or adolescents [21,26]. Shaibi et al. [27] claimed that although FPG and fasting insulin levels are not significantly improved, insulin sensitivity could be found to improve significantly. In conclusion, our intervention programme could improve the health state of the overweigh and obesity children and adolescents by reducing the risk factors of chronic diseases.

Inappropriate diet and lack of physical activity level lead to the imbalance of energy intake and consumption, which is the underlying cause of obesity. Therefore, changing of unhealthy diet habit and increasing practice of physical activity are two important components of obesity intervention programme. Change of lifestyle should be the foundation and key point of obesity intervention, and lifestyle modification should consider the interaction of one's behaviour and environmental factors [13]. At present, even if school-based lifestyle interventions could be beneficial to maintaining a healthy weight, but results differed from each other [28], and the reason may be intervention could not sustain the effect of long-term positive behavioural change. Cox et al. [29] put forward that the off-campus physical activity is the major part of the overall activity level. An effective intervention should not only improve the body state measurements, but also pay more attention to the behavioural change. We used questionnaire to evaluate the diet and exercise habit of the students and observed significant promotion ($P = 0.04$) of the diet and exercise habit after the intervention, which implied that the overweigh and obesity students had positive change of their diet and exercise habits. Therefore, our study illustrated that the overweigh and obesity students could study and benefit from building a healthy lifestyle. Considering the overweight and obesity children are more prone to have low self-esteem, anxiety, depression and other mental diseases than their normal weight peers [30], we included the social

Please cite this article in press as: Guo H, et al. Intervention of childhood and adolescents obesity in Shantou city. Obes Res Clin Pract (2015), http://dx.doi.org/10.1016/j.orcp.2014.11.006

psychological consultation to help these students solving the aspects of the psychological problems. Piers-Harris Child's Self-concept Scale was applied to evaluate self-awareness of the students. The postintervention values for score of the scale had nonsignificant enhancement, the reason may be the level of obesity was not serious enough to induce mental disorder. The other reasons may be the low psychological intervention frequency, the short duration of intervention and the small sample size, which resulted in the nonsignificant outcomes.

There are a few limitations that should be considered when performing data analysis. First, our study was school-based intervention which could not control students' diet and exercise behaviour out of school. Secondly, as the first intervention programme conducted in Shantou city, most students and parents could not concede such intervention study so that they could not be cooperative. Therefore, most of the parents did not sign the inform consents which resulted in the small sample size. Moreover, the short period of intervention lead to the nonsignificant results. Finally, we did not include the exposure time of intervention as one of the analytical parameters, and the duration of exposure to the intervention could have unpredictable effects to overweight and obesity children and adolescents behavioural change.

Conclusion

We presented here a population-based cross-sectional study and novel one-year multicomponent school-based intervention programme conducted in the Shantou city, which could be effective at improving health related status and lifestyle behaviour of overweight and obese children and adolescents, which indicated that it is feasible and important to implement such a school-based intervention programme in Shantou city.

Acknowledgements

We thank the students and their parents in this study and thank the volunteers for assisting the survey. We also thank the Bureau of Education for arranging the physical examination.

Appendix 1. Questionnaire sample

Group	Content
1. Feeding Strategy	*''Parents are always telling me to eat more vegetables and fruits''*, *''Parents are always telling me to eat more meat''*, etc.
2. Parenting behaviour towards the child's physical activity	*''My parents always ask me to do exercises (walking, jogging, and playing basketball)''*, etc.
3. Feeding behaviours	*''Eat at least 3 servings of vegetables a day (1 serving is about a half bow of boiled vegetables)''*, *''Eat at least 2 servings of fruits a day (1 serving is an apple or a banana)''*, etc.
4. Eating environment provided by the parents	*''Everyday, we eat on time''*, *''We watch TV during a meal''*, etc.
5. Food availability	*''We store the fruits at home''*, *''We store the beverages at home''*, etc.
6. Home sedentary environment	*''My parents allow me to watch as much TV as I want''*, *''My parents try to get me to play outside when it is nice''*, etc.
7. Student's physical activity level	*''No physical activity at all, spend most of your time sitting or sleeping. (0 score)''*, etc.
8. Parents' physical activity levels	*''No physical activity at all, spend most of your time sitting or sleeping. (0 score)''*, etc.

Please cite this article in press as: Guo H, et al. Intervention of childhood and adolescents obesity in Shantou city. Obes Res Clin Pract (2015), http://dx.doi.org/10.1016/j.orcp.2014.11.006

References

[1] Wang Y, Lim H. The global childhood obesity epidemic and the association between socio-economic status and childhood obesity. Int Rev Psychiatry 2012;24(3):176—88.
[2] Ji CY, Cheng TO. Prevalence and geographic distribution of childhood obesity in China in 2005. Int J Cardiol 2008;131(1):1—8.
[3] Chen CM, Li YP, Ma GS. Control and prevention of obesity: learn lessons from the United State. Guo Wai Yi Xue Wei Sheng Xue Fen Ce 2006;(5):278—81.
[4] Chen W, Chen JD, Xu HZ. Nutritional status of primary and middle schools students in Shantou, 1997—2006. Appl Prev Med 2007;13(5):304—6.
[5] WHO. Obesity and overweight; 2012 http://www.who.int/mediacentre/factsheets/fs311/en/
[6] Khambalia AZ, Dickinson S, Hardy LL, Gill T, Baur LA. A synthesis of existing systematic reviews and meta-analyses of school-based behavioural interventions for controlling and preventing obesity. Obes Rev 2012;13(3):214—33.
[7] Brug J, Velde SJ, Chinapaw MJ, Bere E, de Bourdeauhuij I, Moore H, et al. Evidence-based development of school-based and family-involved prevention of overweight across Europe: the ENERGY-project's design and conceptual framework. BMC Public Health 2010;10:276.
[8] Singh AS, Chin A, Paw MJ, Brug J, van Mechelen W. Dutch obesity intervention in teenagers: effectiveness of a school-based program on body composition and behavior. Arch Pediatr Adolesc Med 2009;163(4):309—17.
[9] Farris JW, Taylor L, Williamson M, Robinson C. 12-Week interdisciplinary intervention program for children who are obese. Cardiopulm Phys Ther J 2011;22(December (4)):12—20.
[10] Liu A, Hu X, Ma G, Cui Z, Pan Y, Chang S, et al. Evaluation of a classroom-based physical activity promoting programme. Obes Rev 2008;9(Suppl. 1):130—4.
[11] McDowell MA, Fryar CD, Ogden CL. Anthropometric reference data for children and adults: United States, 2003—2006, National health statistics reports; no. 10. Hyattsville, MD: National Center for Health Statistics; 2008.
[12] The NHMRC. Clinical practice guidelines for the management of overweight and obesity in children and adolescents. Canberra: Commonwealth Department of Health and Ageing; 2003 http://www.obesityguidelines.gov.au
[13] Campbell KJ, Crawford DA, Ball K. Family food environment and dietary behaviors likely to promote fatness in 5—6 year-old children. Int J Obes 2006;30(8):1272—80.
[14] WHO. Global strategy on diet, physical activity and health; 2010 http://www.who.int/dietphysicalactivity/factsheet_young_people/en/index.html
[15] Bandura A. Health promotion by social cognitive means. Health Educ Behav 2004;31(2):143—64.
[16] Schneider HJ, Friedrich N, Klotsche J, Pieper L, Nauck M, John U, et al. The predictive value of different measures of obesity for incident cardiovascular events and mortality. J Clin Endocrinol Metab 2010;95(April (4)):1777—85.
[17] Burguera B, Colom A, Pinero E, Yanez A, Caimari M, Tur I, et al. ACTYBOSS: activity, behavioral therapy in young subjects — after-school intervention pilot project on obesity prevention. Obes Facts 2011;4(5):400—6.
[18] Kropski JA, Keckley PH, Jensen GL. School-based obesity prevention programs: an evidence-based review. Obesity (Silver Spring) 2008;16(5):1009—18.
[19] Kuczmarski RJ, Ogden CL, Guo SS, Grummer-Strawn LM, Flegal KM, Mei Z, et al. CDC growth charts for the United States: methods and development. Vital Health Stat 2002;11(246):1—190.
[20] Inokuchi M, Matsuo N, Takayama JI, Hasegawa T. BMI z-score is the optimal measure of annual adiposity change in elementary school children. Ann Hum Biol 2011;38(6):747—51.
[21] Sun MX, Huang XQ, Yan Y, Li BW, Zhong WJ, Chen JF, et al. One-hour after-school exercise ameliorates central adiposity and lipids in overweight Chinese adolescents: a randomized controlled trial. Chin Med J (Engl) 2011;124(3):323—9.
[22] Robertson W, Friede T, Blissett J, Rudolf MC, Wallis M, Stewart-Brown S. Pilot of ''Families for Health'': community-based family intervention for obesity. Arch Dis Child 2008;93(11):921—6.
[23] Samara A, Ventura EE, Alfadda AA, Goran MI. Use of MRI and CT for fat imaging in children and youth: what have we learned about obesity, fat distribution and metabolic disease risk? Obes Rev 2012;13(8):723—32.
[24] Freedman DS, Katzmarzyk PT, Dietz WH, Srinivasan SR, Berenson GS. Relation of body mass index and skinfold thicknesses to cardiovascular disease risk factors in children: the Bogalusa Heart Study. Am J Clin Nutr 2009;90(1):210—6.
[25] Botton B, Heude A, Kettaneh SR, Borys IM, Lommez A, Bresson JL, et al. Cardiovascular risk factor levels and their relationships with overweight and fat distribution in children: the Fleurbaix Laventie Ville Sante II study. Metabolism 2007;56(5):614—22.
[26] van der Heijden GJ, Wang ZJ, Chu ZD, Sauer PJ, Haymond MW, Rodriguez LM, et al. A 12-week aerobic exercise program reduces hepatic fat accumulation and insulin resistance in obese, Hispanic adolescents. Obesity (Silver Spring) 2010;18(2):384—90.
[27] Shaibi GQ, Davis JN, Weigensberg MJ, Goran MI. Improving insulin resistance in obese youth: choose your measures wisely. Int J Pediatr Obes 2011;6(2-2):e290—6.
[28] Brown T, Summerbell C. Systematic review of school-based interventions that focus on changing dietary intake and physical activity levels to prevent childhood obesity: an update to the obesity guidance produced by the National Institute for Health and Clinical Excellence. Obes Rev 2009;10(1):110—41.
[29] Cox M, Schofield G, Greasley N, Kolt GS. Pedometer steps in primary school-aged children: a comparison of school-based and out-of-school activity. J Sci Med Sport 2006;9(1/2):91—7.
[30] Hollar D, Lombardo M, Lopez-Mitnik G, Hollar TL, Almon M, Agatston AS, et al. Effective multi-level, multi-sector, school-based obesity prevention programming improves weight, blood pressure, and academic performance, especially among low-income, minority children. J Health Care Poor Underserved 2010;21(2 Suppl.):93—108.

Available online at www.sciencedirect.com
ScienceDirect
