# Supplementary material for: Systematic review and meta-analysis of school-based obesity interventions in mainland China
Source: PLoS One. 2017 Sep 14;12(9):e0184704. doi: 10.1371/journal.pone.0184704 (PMC5598996; doi:10.1371/journal.pone.0184704)
Supplement: S1 Dataset — (ZIP) [file pone.0184704.s007.zip › S1_dataset/76库/32.pdf]

BIOMEDICAL AND ENVIRONMENTAL SCIENCES **20**, 19-23 (2007)

Report on Childhood Obesity in China (6) Evaluation of a Classroom-based Physical Activity Promotion Program[1]

AI-LING LIU, XIAO-QI HU, GUAN-SHENG MA[2], ZHAO-HUI CUI, YONG-PING PAN[*], SU-YING CHANG[#], WEN-HUA ZHAO[#], AND CHUN-MING CHEN[#]

National Institute for Nutrition and Food Safety, Chinese Center for Disease Control and Prevention, 29 Nanwei Road, Beijing 100050, China; [*]Dongcheng School Healthcare Institute, Beijing 100007, China; [#]International Life Science Institute Focal Point-China, Beijing 100050, China

Objectives To evaluate the effect of Happy 10 program on the promotion of physical activity, physical growth and development of primary-school students, and on obesity control and prevention. **Methods** Two similar primary schools from one district of Beijing, China were selected, one as an intervention school and the other as a control school. Happy 10 program was implemented at least once every school day in the intervention school for two semesters, whereas no intervention was adopted in the control school. The information on energy expenditure and duration of physical activity was collected by a validated 7-day physical activity questionnaire. Height and weight were measured by trained investigators following the standardized procedure. Energy expenditure and intensity of each Happy 10 session was measured by a physical activity monitor. **Results** The average energy expenditure and duration of total physical activity per day among students in the intervention school increased significantly from 15.0 to 18.2 kcal/kg, and 2.8 to 3.3 h respectively, whereas the figures significantly decreased in the control school. There was a significant difference in change of weight and BMI between girls in the intervention and control school (2.4 kg *vs* 4.6 kg, -0.47 kg/m^2 *vs* 0.66 kg/m^2). The prevalence of overweight and obesity in the intervention school decreased by 0.4%-5.6%, as compared to the increase by 0.6%-4.5% in the control school. The average energy expenditure and intensity per 10-minute session ranged from 25.0-35.1 kcal, 4.8-6.2 kcal/kg/h respectively in grades 1-5. **Conclusion** Happy 10 program provides a useful strategy to promote physical activity among school children and also plays a positive role in building up physical growth and development of girls.

Key words: Happy 10; Physical activity; Obesity; Intervention

INTRODUCTION

Regular participation in physical activity is an essential component of healthy lifestyle, which helps to prevent certain chronic childhood conditions, including hypertension, diabetes, obesity, abnormal lipid profiles, as well as depression[1]. However, physical inactivity among children and adolescents is becoming a major concern in China. Children and adolescents spend more time on sedentary activities, such as viewing TV, using internet and playing computer games, instead of doing physical activities. It is indicated that only 9.2% of children aged 6-12 years are doing exercise more than three times/week with duration of more than 20 minutes[2], while 41.4% of them spend at least two hours on watching television[3].

To respond to the need for increasing physical activity level among school-aged children, a classroom-based physical activity promotion program—Happy 10 was initiated by the National Institute for Nutrition and Food Safety, Chinese Center for Disease Control and Prevention, based on the principle of TAKE 10!™[4]. The Happy 10 program was implemented in urban Beijing since 2004. The purpose of the present study is to evaluate the effect of the program on the promotion of physical activity, physical growth and development of school children, and on obesity control and prevention among them.

SUBJECTS AND METHODS

Two similar primary schools were selected from the same district of urban Beijing, one school (14 classes) as an intervention group and the other (12

[1]This research was funded by International Life Science Institute Focal Point-China.

[2]Correspondence should be addressed to Guan-Sheng MA. Tel: 86-10-83132572. Fax: 86-10-83132021. E-mail: mags@chinacdc.net.cn

Biographical note of the first author: Ai-Ling LIU, female, born in 1972, professor, majoring in nutrition, physical activity of children and adolescents and chronic disease control and prevention.

0895-3988/2007
CN 11-2816/Q
Copyright © 2007 by China CDC

?1994-2015 China Academic Journal Electronic Publishing House. All rights reserved. http://www.cnki.net

classes) as a control group.

Happy 10 program is a classroom-based physical activity program for primary-school students which is based on the principle of "TAKE 10!TM" Program developed by the Health Promotion Center of International Life Science Institute in USA. Many safe and age- and space-appropriate physical activities are included in the program materials. Happy 10 program was actually organized and implemented by the teachers among students in grades 1-5 in the intervention school, taking about 10 minutes at least once every school day. Tracking posters and stickers were used to illustrate progress of each grade. The program covered in total two semesters from October 2004 to June 2005. No intervention was implemented in the control school.

Information on age, gender, height, weight and physical activity patterns of all students in both intervention and control schools were collected pre- and post-intervention. The height and weight were measured by the trained investigators following the standardized procedure. Information on physical activity patterns was collected using a validated 7-day physical activity questionnaire which was interviewed administrated for students of grade 1-2 and self-administrated for students of grade 3-5. The average energy expenditure and duration of total physical activity per day were calculated from the questionnaire[5]. Body mass index (BMI) was calculated as weight (kg) divided by square of height (m). The overweight and obesity was defined by age- and sex-specific BMI, which is recommended by Group of China Obesity Task Force[6].

The energy expenditure of each session of Happy 10 was measured using a physical activity monitor (Zhi-Ji UX-01, Beijing YHKI Sci-Tech Development Co. LTD) worn on left side of the front waist. A total of 80 students (8 boys and 8 girls from each grade) were selected in the intervention school. Each student was evaluated for five consecutive schooldays. The monitors were worn just before each session of Happy 10 and removed after the session was over, and the teachers recorded the type and duration of activities implemented and figures displayed by the monitors at the end of each session. The energy expenditure per session was read directly from the monitors and the metabolic rate (MET) was calculated as energy expenditure divided by weight, which was used to assess the intensity of the session.

The mean and standard deviation were used to describe continual variables. An analysis of variance (ANOVA) and Chi-square test were used to compare the difference in daily energy expenditure and duration of physical activity, height, weight, BMI and prevalence of overweight and obesity among various groups. All *P*-values were two-sided. A level $P<0.05$ was accepted as statistical significance.

RESULTS

A total of 753 students (357 boys, 396 girls) aged 6-12 years were investigated. Three hundred and twenty eight students (150 boys and 178 girls) were from the intervention school, while 425 students (207 boys and 218 girls) were from the control school.

Comparison of Change of Daily Physical Activity Level Between the Intervention and Control School

Table 1 shows the change of daily energy expenditure and duration of physical activity before and after the intervention. Significant increases in the average daily physical activity energy expenditure (PAEE) and duration among the students in the intervention school were found before and after the intervention (15.0 kcal/kg *vs* 18.2 kcal/kg, 2.8 h *vs* 3.3 h respectively). In the contrast, significant decreases of these two variables were found in the control school (24.3 kcal/kg *vs* 14.7 kcal/kg, 4.4 h *vs* 2.9 h respectively). There was significant difference in change of energy expenditure and duration of physical activity between the intervention school and the control school.

TABLE 1

Change of Energy Expenditure and Duration of Physical Activity of Students Pre- and Post-intervention[1]

Variables	Intervention School (*n*=328)			Control School (*n*=425)		
	Pre-intervention	Post-intervention	Diff	Pre-intervention	Post-intervention	Diff
PAEE (kcal/kg/d)	15.0	18.2	3.1*	24.3	14.7	-9.6*,#
Moderate	5.0	6.7	1.7 *	8.1	4.9	-3.2*,#
Vigorous	8.8	10.3	1.6*	14.2	7.2	-7.0*,#
PA Time (h/d)	2.8	3.3	0.5*	4.4	2.9	-1.5*,#
Moderate	1.2	1.5	0.4*	1.8	1.5	-0.3*,#
Vigorous	1.1	1.4	0.3*	1.9	0.9	-0.8*,#

Note. PAEE: Physical activity energy expenditure; PA: Physical activity; [1]analysis of variance (ANOVA); * there was significant difference pre- and post-intervention within the group; # there was significant difference of change between the intervention school and the control school after the intervention.

?1994-2015 China Academic Journal Electronic Publishing House. All rights reserved. http://www.cnki.net

Comparison of Change of Height, Weight, and BMI Between the Intervention and Control School

It can be seen from Table 2 that there was a significant increase of height and weight after eight month intervention in both the intervention and control schools. But there was no significant difference in the change of height and weight between the intervention and control school except the change of weight between the intervention girls and the control girls (2.4 kg *vs* 4.6 kg). The BMI of boys in both the intervention and control schools and girls in the control school increased significantly after the intervention. But the figure of girls in the intervention school decreased significantly from 18.63 kg/m^2 to 18.16 kg/m^2, and there was a significant difference in the change of BMI between the intervention girls and the control girls.

TABLE 2

Change of Height, Weight, and BMI of Students Pre- and Post-intervention[1]

Variables		Intervention School (*n*=328)			Control School (*n*=425)		
		Pre-intervention	Post-intervention	Diff	Pre-intervention	Post-intervention	Diff
Height (cm)	Boys	139.5	144.1	4.6*	136.4	142.1	5.7*
	Girls	136.6	142.7	6.1*	133.6	140.8	7.2*
Weight (kg)	Boys	35.7	40.1	4.4*	33.8	38.4	4.6*
	Girls	35.0	37.4	2.4*	29.7	34.3	4.6$^{*,\#}$
BMI (kg/m^2)	Boys	18.09	18.95	0.86*	17.96	18.68	0.72*
	Girls	18.63	18.16	-0.47*	16.42	17.08	0.66$^{*,\#}$

Note. [1]Analysis of variance (ANOVA). *There was significant difference pre- and post-intervention within the group; $^\#$there was significant difference in change between the intervention school and the control school after the intervention.

Comparison of Change of Prevalence of Overweight and Obesity Between the Intervention and Control School

The prevalence of overweight and obesity in the intervention school was 20.9% and 15.0% for boys, and 15.3% and 16.9% for girls before the intervention, which decreased to 17.1% and 14.6%, 12.0% and 11.3% respectively after two-semester intervention. On the contrast, the prevalence of overweight and obesity in the control school increased by 0.6%-4.5% during the same period of time. There was no significant difference in change of the prevalence of overweight and obesity between the intervention school and the control school (Table 3).

TABLE 3

Change in the Prevalence of Overweight and Obesity Among Students Pre- and Post-intervention (%)[1]

		Intervention School (*n*=328)			Control School (*n*=425)		
		Pre-intervention	Post-intervention	Diff	Pre-intervention	Post-intervention	Diff
Overweight	Boys	20.9	17.1	-3.8	17.8	22.3	4.5
	Girls	15.3	12.0	-3.3	8.0	11.7	3.7
Obesity	Boys	15.0	14.6	-0.4	17.2	17.8	0.6
	Girls	16.9	11.3	-5.6	7.3	8.0	0.7

Note. [1]Chi-square test.

Average Energy Expenditure and Intensity for One Session of Happy 10

Table 4 indicates the energy expenditure level and intensity of children for each session of Happy 10. The average caloric expenditure per session of each grade ranged from 25.0 to 35.1 kcal. There was a significant difference in energy expenditure among grades, and it was highest among the students in grade 3. After adjustment for body weight, the average METs value per session of each grade ranged from 4.8 to 6.2 kcal/kg/h. All sessions were associated with MET value in the moderate (3$\leqslant$METs<6) to vigorous (METs$\geqslant$6). Students in grade 3 were most active among students in the intervention school. They had 4 days with vigorous activities (METs 7.3 kcal/kg/h). The METs of students in grade 5 during the five-day session ranked the lowest.

?1994-2015 China Academic Journal Electronic Publishing House. All rights reserved. http://www.cnki.net

TABLE 4

Energy Expenditure and Intensity per Happy 10 Session by Grade

Grade	*n*	Energy Expenditure (kcal)		Intensity (kcal/kg/h)					
				Moderate		Vigorous		Total	
		Mean	STD	Days	METs	Days	METs	Days	METs
1	16	25.0	7.5	3	5.8	2	6.7	5	5.8
2	16	32.5	16.6	3	5.7	2	6.4	5	5.7
3	16	35.1	13.5	1	5.6	4	7.3	5	6.2
4	16	30.0	11.2	2	4.7	3	6.1	5	5.4
5	16	33.7	15.0	5	4.8	0	-	5	4.8

DISCUSSION

Classroom-based physical activity program provides a unique opportunity to enhance the current health status of children during their critical periods of growth and development because virtually all children attend school. In addition, physical activity is a lifetime course tracking from one's childhood to adulthood[7]. That is why researchers and medical practitioners recommend that school based physical activity interventions be developed. Happy 10 is a classroom-based physical activity intervention for Chinese primary-school students. This study is designed to evaluate effectiveness of the Happy 10 intervention in promoting physical activity level among children and to examine its influence on physical growth and development, as well as on obesity control. The findings of the present study have confirmed that Happy 10 is a useful classroom-based physical activity intervention.

Our findings have indicated that Happy 10 is effective in increasing physical activity level among students in grades 1-5. Participants in individual Happy 10 session burned a modest number of kcal, ranging from 25.0 to 35.1 per 10-minute session, which was similar to the TAKE 10! activities[8]. Participants achieved exercise intensities in the moderate to vigorous range at the activity session. Exercise intensity varied by grade levels because of the different activities involved and different rhythms and extents of the same activity. Students in lower grades were more active than those in higher grades. Energy expenditure and intensity per session did not mean single Happy 10 activity's energy expenditure and intensity. Two to three activities were actually involved in one session because students liked this pattern, which was more attractive than doing only one activity at each session. Although a relatively small number of energy was expended per session, the most important thing was that students were encouraged to participate in more physical activities using the skills and knowledge learned from Happy 10 on other occasions. Total daily energy expenditure increased by 3.1 kcal/kg/d after the intervention among students in the intervention group, while it decreased among their counterparts. Energy expenditure accumulated in such a way may create a positive impact on maintaining healthy body weight and preventing the development of overweight and obesity[9].

There is significant difference in change of weight and BMI after the intervention only between the intervention girls and the control girls. Such results can be explained by the following factors. First, more attention is paid on body shape by girls and girls' body dissatisfaction scores is significantly higher than boys'[10], therefore they may keep more active during Happy 10 than boys. Second, the physical activity level of boys is higher than that of girls[11], so the Happy 10 program exerts relatively less influence on the physical activity level of the former. The program's effect on physical development may be insignificant.

Although overweight and obesity data showed no significant differences between the intervention and control school, there was a positive change taken place pre- and post-intervention. The prevalence of overweight and obesity among the intervention students decreased, while the figure among the control students increased at the same period. No significant difference was likely due to the influence of other factors, such as short time, dietary practice and small sample size.

Findings of the present study suggest that Happy 10 can increase the physical activity level of children and can positively influence their health status. Moreover, it is well accepted by the participants. Teachers involved in implementing Happy 10 indicate that the program is easy to implement and activities are enjoyable and attractive to their students. They will be interested to implement this program in the next semester. Students are active to participate in all sessions. Parents also support their children to take part in the program. In conclusion, it is feasible to implement Happy 10 program in China.

?1994-2015 China Academic Journal Electronic Publishing House. All rights reserved. http://www.cnki.net

REFERENCES

1. U. S. Department of Health and Human Services (1996). Physical Activity and Health: A Report of the Surgeon General. Atlanta, GA: U. S. Department of Health and Human Services, Centers for Disease Control and Prevention, National Center for Chronic Disease Prevention and Health Promotion, International Medical Publishing, 85-142.
2. Wu Y F, Ma G S, Hu Y H, *et al*. (2005). The current prevalence status of body overweight and obesity in China: data from the China National Nutrition and Health Survey. *Chin J Prev Med* **39**(5), 316-320.
3. Ma G S, Liu A L, Cui Z H, *et al*. (2006). The situation of television viewing of people in China. *Chin J Health Educa* **22**(3), 167-170.
4. http://www.take10.net.
5. Liu A L, Ma G S, Zhang Q, *et al*. (2003). Reliability and validity of a 7-day physical activity questionnaire for elementary students. *Chin J Epidemiol* **24**(10), 901-904.
6. Group of China Obesity Task Force (2004). Body mass index reference norm for screening overweight and obesity in Chinese Children and adolescents. *Chin J Epidemiol* **25**(2), 97-102. (In Chinese)
7. Pate R R, Baranowski T, Dowda M, *et al*. (1996). Tracking of physical activity in young children. *Med Sci Sports Exerc* **28**, 82-96.
8. Stewart J A, Dennison D A, Kohl H W, *et al*. (2004). Exercise level and energy expenditure in the Take 10!® In-class physical activity program. *J Sch Health* **174**(10), 397-400.
9. Hill J O, Wyatt H R, Reed G W, *et al*. (2003). Obesity and the environment: where do we go from here? *Science* **199**(5608), 853-855.
10. Hu X Q, Wang D, Cui Z H, *et al*. (2006). Analysis on body image perception among pupils in urban Beijing. *Chin J School Health* **27**(1), 22-25.
11. Sallis J F, Strikmiller P K, Harsha D W, *et al*. (1996). Validation of interviewer- and self-administered physical activity checklists for fifth grade students. *Med Sci Sports Exerc* **28**(7), 840-851.

(*Received November* 8, 2006 *Accepted December* 19, 2006)

?1994-2015 China Academic Journal Electronic Publishing House. All rights reserved. http://www.cnki.net
