# Supplementary material for: Systematic review and meta-analysis of school-based obesity interventions in mainland China
Source: PLoS One. 2017 Sep 14;12(9):e0184704. doi: 10.1371/journal.pone.0184704 (PMC5598996; doi:10.1371/journal.pone.0184704)
Supplement: S1 Dataset — (ZIP) [file pone.0184704.s007.zip › S1_dataset/76库/36.pdf]

南京市中小学生营养午餐膳食干预研究

孙桂菊　胡永祯　杨瑾　王少康　罗海燕　徐华珠　盛军利　陈国威　谢莹　杨立刚

东南大学公共卫生学院营养与食品卫生系，南京 210009

摘要：**目的** 在南京市中小学生中开展营养午餐膳食干预，并对干预效果进行初步评价，为南京市大规模开展营养午餐提供理论和实践依据。**方法** 分别选取一所中学和一所小学，由午餐供应部门在三个月干预期间每次提前把下一周的食谱送给我们，我们按照营养午餐营养素的标准（依据为中国营养学会 2001 年修订的《中国居民膳食营养素参考摄入量》（DRIs）标准的 40%）进行评价，指出其中的不足之处，提出改进建议，午餐供应部门负责人采纳后付之实施。并分别设立对照组，不进行任何的干预。在开展营养午餐干预前后分别对中学的初一学生，小学的四年级学生进行膳食调查，调查学生午餐膳食摄入情况，同时对学生进行体检，项目包括身高、体重和血红蛋白以及头发中部分无机元素的检测。对调查结果进行综合分析，从而评价干预效果。**结果** 学生午餐营养素摄入能量、钙、维生素 C 在干预后有较大增加，与干预前比较有显著性差异，但是三大热能营养素供热比例失调依然存在；学生贫血比例显著性下降，学生超重、肥胖比例有所下降，但是差异没有显著性，学生头发中无机元素含量偏低比例有所下降，但是变化没有显著性。**结论** 本研究结果表明，本次膳食营养午餐干预对南京市中小学生平衡膳食以及身体素质改善起到了一定的效果，但如何更好的改善超重、肥胖状况还有待进一步研究。本课题提供了一种膳食营养午餐干预方案策略，为南京市中小学校以后开展营养午餐提供了一定的理论和实践依据。

关键词：营养午餐；干预；中小学生

合理膳食和营养健康教育是预防营养相关性疾病的基础[1]。学龄儿童青少年大都在学校食堂吃午餐，学生集体用餐营养的好坏，直接关系到学生的身体健康，但是多项调查显示学校午餐存在营养不平衡状况[2-4]，因此国内外均广泛的在学校开展营养午餐。学生营养午餐问题在我国近几年来已引起了有关部门和社会各界有识之士的重视。《中国营养改善行动计划》明确提出："有计划、有步骤地普及学生营养午餐"[5]，肯定了发展营养餐的方向。目前国内杭州、北京、上海等城市均先后在中小学开展了营养午餐，而南京中小学校到目前还没有真正的开展。为此，本研究希望通过在南京学校开展营养午餐干预试点，为南京市中小学校以后开展营养午餐提供科学依据。

1 研究对象与方法

1.1 研究对象

本研究选择位于南京市白下区的行知实验中学、火瓦巷小学作为干预试点学校；选择分别位于南京市下关区第 12 中学和白下区的光华东街小学作为对照学校。对行知实验中学初一年级所有学生一共 217 人（男生 110 人，女生 107 人）和火瓦巷小学四年级所有学生一共 94 人（男生 53 人，女生 41 人）进行营养健康教育，而取第 12 中学初一年级学生 118 人（男生 66 人，女生 42 人）和光华东街小学四年级所有学生一共 114 人（男生 62 人，女生 52 人）作对照组。

1.2 方法

1.2.1 膳食午餐食谱干预

行知实验中学学生由学校食堂供应午餐，而火瓦巷小学由快餐企业送餐。两个午餐供应部门在三个月干预期间每次提前把下一周的食谱送给我们，我们按照营养午餐营养素的标准（依据为中国营养学会2001年修订的《中国居民膳食营养素参考摄入量》（DRIs）标准的40%）进行评价，指出其中的不足之处，提出改进建议，午餐供应部门负责人采纳后实施。

1.2.2 膳食调查

调查人员在学生午餐前到达学生就餐地点，结合学校午餐食谱食物用量、就餐人数、学生添加饭菜、剩饭菜情况，由调查人员在学生就餐时进行现场调查，记录午餐学生摄入食物种类及数量，另外发给学生调查表格，嘱咐他们将当天午餐后到第二天午餐前摄入所有食物及零食的种类及数量详细记录在表格中。使用中国CDC营养与食品安全所设计的营养计算器计算每人每日能量和营养素摄入量，依据中国营养学会2001年修订的《中国居民膳食营养素参考摄入量》（DRIs）标准进行评价，午餐依据每日标准的40%进行评价。

1.2.3 营养状况调查

测量身高、体重，按照国际生命科学学会中国肥胖工作组推荐的中国学生超重、肥胖BMI筛查标准进行评价[6]。血红蛋白测定用氰化高铁血红蛋白法，依据检测结果，按WHO标准筛查贫血：6～13岁Hb<120g/L即为贫血[7]。

1.2.4 头发中元素硒、铁、锌及钙的测定

电感耦合等离子体发射光谱法测定头发中钙、铁和锌；氢化物发生原子吸收分光光度法测定头发中硒。

2 结果

2.1 午餐干预前后学生膳食营养状况分析

2.1.1 学生各种营养素每日午餐摄入量占参考摄入量的百分比（%）

2.1.1.1 干预学校与对照学校中学生各种营养素每日午餐摄入量占参考摄入量百分比（%）的前后比较见表1和表2。

表1 干预学校与对照学校中学男生各种营养素每日午餐摄入量占参考摄入量百分比（%）的前后比较

营养素	干预学校		对照学校	
	前	后	前	后
能量(kJ)	51.2±22.5	68.7±29.6*	54.4±25.8	56.4±24.1
蛋白质（g）	98.8±45.6	100.7±44.2	98.1±47.5	96.0±51.3
维生素A (μgRE)	78.1±121.3	76.4±121.5	77.1±125.1	75.4±123.6
硫胺素（mg）	86.7±58.6	84.8±58.4	88.7±55.8	86.1±57.1
核黄素（mg）	64.4±32.9	70.2±41.8	68.7±40.2	67.2±37.7
尼克酸（μg）	192.2±81.7	188.7±75.3	142.3±70.2	139.1±70.7
维生素C (mg)	88.9±68.1	91.1±72.4	88.5±77.6	90.3±73.4
维生素E (mg)	66.5±72.3	79.4±84.3*	70.1±76.8	81.0±101.2*

钙（mg）	39.2±32.9	63.2±36.8*	41.1±36.8	39.3±31.7
磷（mg）	97.5±40.6	98.1±42.3	95.2±36.4	94.5±40.7
钾（mg）	110.8±48.7	104.1±55.4	100.5±40.3	112.1±63.1
镁（mg）	71.8±29.9	70.2±27.5	72.4±35.1	68.4±29.5
铁（mg）	169.4±97.5	158.3±94.4	162.3±68.5	154.1±75.3
锌（mg）	79.1±33.7	80.5±35.6	75.5±32.1	72.4±34.2
硒（μg）	95.1±55.4	98.7±64.2	92.4±65.8	90.2±54.6
铜（mg）	81.2±59.8	83.0±55.7	87.6±58.2	81.2±45.2

* 前后比较，t 检验，$p<0.05$

从表 1 中可以看出，干预学校中学男生能量、维生素 E 以及钙的午餐摄入有显著性提高，接近参考摄入量；而对照学校中学男生只有维生素 E 的摄入有显著性提高。

表 2 干预学校与对照学校中学女生各种营养素每日午餐摄入量占参考摄入量百分比（%）的前后比较

营养素	干预学校		对照学校	
	前	后	前	后
能量(kJ)	55.8±31.4	72.3±25.8*	55.4±24.5	57.3±26.3
蛋白质（g）	97.5±50.0	94.7±43.1	97.2±50.4	96.5±48.7
维生素A (μgRE)	93.4±111.5	96.5±107.0	95.4±104.3	90.5±105.4
硫胺素（mg）	80.8±61.5	81.4±57.2	83.4±54.2	82.4±53.8
核黄素（mg）	68.5±36.7	70.4±42.2	67.1±33.4	65.7±34.6
尼克酸（μg）	177.6±72.4	182.5±74.7	166.4±70.5	163.1±68.7
维生素C (mg)	97.1±73.8	94.5±70.8	98.1±73.4	96.5±69.9
维生素E (mg)	80.6±124.2	82.5±96.5	79.4±115.8	81.2±124.5
钙（mg）	38.7±35.7	65.4±37.8*	39.4±35.7	41.5±31.1
磷（mg）	94.2±42.3	94.3±41.8	90.2±47.8	90.8±42.5
钾（mg）	111.1±67.2	107.8±57.6	113.1±62.2	112.5±67.9
镁（mg）	68.9±24.7	70.7±26.3	70.2±32.8	69.4±28.0
铁（mg）	132.1±88.7	121.3±97.2	140.4±87.3	151.3±100.5
锌（mg）	86.3±37.1	85.4±31.9	82.3±45.4	71.5±35.5*
硒（μg）	93.7±51.2	95.5±63.0	93.3±60.2	91.7±57.4
铜（mg）	77.3±45.4	78.1±45.3	79.2±35.8	80.8±45.7

* 前后比较，t 检验，$p<0.05$

从表 2 中可以看出干预学校中学女生能量以及钙的午餐摄入有显著性提高，接近参考摄入量；而对照学校中学女生锌的摄入更显不足。

2.1.1.2 干预学校与对照学校小学生各种营养素每日午餐摄入量占参考摄入量百分比（%）的前后比较

见表 3 和表 4。

表 3 干预学校与对照学校小学男生各种营养素每日午餐摄入量占参考摄入量百分比（%）的前后比较

营养素	干预学校		对照学校	
	前	后	前	后
能量(kJ)	49.1±15.7	71.1±21.2*	51.2±21.5	62.3±19.7*
蛋白质（g）	98.7±52.1	95.3±60.2	87.4±35.8	90.4±44.2
维生素A (μgRE)	71.2±71.2	82.5±92.3*	74.1±70.5	72.4±71.0
硫胺素（mg）	81.2±62.7	78.7±66.5	77.2±65.7	80.7±60.2
核黄素（mg）	65.8±31.1	64.4±38.7	67.1±39.2	68.2±45.3
尼克酸（μg）	174.1±83.2	175.4±76.6	166.1±74.3	160.2±53.4
维生素C (mg)	77.8±61.1	76.2±54.8	76.6±62.1	61.2±49.8*
维生素E (mg)	71.4±88.5	77.2±95.4	75.2±88.1	77.0±58.4
钙（mg）	42.1±37.3	66.2±40.5*	51.4±25.5	49.5±31.2
磷（mg）	99.1±46.7	102.3±58.7	93.5±57.6	96.7±40.5
钾（mg）	95.1±44.6	98.2±51.2	96.4±66.1	93.4±63.8
镁（mg）	70.4±30.2	83.1±44.5*	75.3±37.7	76.2±33.4
铁（mg）	128.4±66.1	121.4±54.7	141.5±88.7	133.5±75.6
锌（mg）	82.1±34.1	72.4±37.2	78.2±33.2	77.4±42.5
硒（μg）	89.7±44.2	88.2±44.5	88.7±42.4	80.7±55.4
铜（mg）	87.2±70.4	84.3±65.7	82.4±79.4	78.9±67.2

* 前后比较，t 检验，$p<0.05$

从表 3 中可以看出干预学校小学男生能量、维生素 A、钙以及镁的午餐摄入有显著性提高，接近参考摄入量；对照学校小学男生只有能量的摄入有显著提高，而维生素 C 的摄入显著减少。

表 4 干预学校与对照学校小学女生各种营养素每日午餐摄入量占参考摄入量百分比（%）的前后比较

营养素	干预学校		对照学校	
	前	后	前	后
能量(kJ)	50.6±14.8	74.6±18.2*	52.1±15.4	57.5±18.9
蛋白质（g）	107.5.1±44.2	104.7±52.1	106.5±44.7	103.4±42.7
维生素A (μgRE)	82.1±74.7	75.8±73.5	81.2±74.5	78.6±66.0
硫胺素（mg）	78.7±62.9	80.6±57.4	82.4±74.3	80.1±66.5
核黄素（mg）	71.3±32.4	67.2±31.0	70.1±33.2	69.4±35.8
尼克酸（μg）	154.6±72.1	147.3±87.1	178.4±79.8	164.5±51.8*
维生素C (mg)	88.6±61.5	90.2±77.8	88.1±67.3	90.5±71.1
维生素E (mg)	62.1±88.7	74.8±73.9*	65.2±84.1	62.1±77.2
钙（mg）	44.5±34.1	66.8±42.1*	45.9±37.4	46.4±37.2
磷（mg）	95.3±37.8	96.9±56.7	95.7±41.9	89.7±51.6
钾（mg）	98.7±46.7	101.2±59.6	104.3±66.8	98.8±46.3
镁（mg）	70.2±30.5	75.7±42.8	71.6±28.7	70.2±35.4
铁（mg）	135.6±67.7	127.4±72.2	127.8±61.9	131.0±55.1
锌（mg）	87.5±37.0	90.6±33.2	87.6±33.2	89.1±33.4
硒（μg）	82.1±43.6	83.7±53.6	82.1±52.1	79.4±38.7
铜（mg）	85.4±62.5	88.7±61.4	82.1±58.4	86.4±75.4

* 前后比较，t 检验，$p<0.05$

从表 4 中可以看出干预学校小学女生能量、维生素 E 以及钙的午餐摄入有显著性提高，接近参考摄入量。

2.1.2 学生午餐膳食三大热能营养素供热比

干预学校中学男生前后膳食中碳水化合物供热比分别为 52.2%、59.1%，经 t 检验有统计学差异（$p<0.05$）。其余各组学生膳食中蛋白质、脂肪与碳水化合物供热比同全天类似分别在 15%～23%、22%～29%、49%～56%之间波动，经统计学检验，差异无显著性。说明学生在营养教育前后高蛋白饮食仍然存在，没有得到根本改善。

2.2 学生干预前后体检结果比较

2.2.1 中学生贫血、超重和肥胖情况 见表 5 和表 6。

表 5 干预学校与对照学校中学男生贫血、超重和肥胖情况前后比较

	干预学校		对照学校	
	前	后	前	后
贫血人数（率）	27（24.5%）	11（10.0%）*	12（18.2%）	11（16.7%）
超重人数（率）	25（22.7%）	21（19.1%）	11（16.7%）	13（19.7%）
肥胖人数（率）	12（10.9%）	8（7.3%）	4（6.1%）	5（7.6%）

* 前后比较，$\chi2=8.14$，$p<0.05$

表 6 干预学校与对照学校中学女生贫血、超重和肥胖情况前后比较

	干预学校		对照学校	
	前	后	前	后
贫血人数（率）	33（30.8%）	21（19.6%）	9（21.4%）	10（23.8%）
超重人数（率）	19（17.8%）	16（14.9%）	8（19.0%）	8（19.0%）
肥胖人数（率）	7（6.5%）	6（5.6%）	2（4.8%）	1（2.4%）

经统计学检验，各组差异均无显著性。

2.2.2 小学生贫血、超重和肥胖情况 见表 7 和表 8（干预学校小学男生有一人未参加体检）。

表 7 干预学校与对照学校小学男生贫血、超重和肥胖情况前后比较

	干预学校		对照学校	
	前	后	前	后
贫血人数（率）	14（26.9%）	6（11.5%）*	12（19.4%）	11（17.7%）
超重人数（率）	15（28.8%）	12（23.1%）	16（25.8%）	14（22.6%）
肥胖人数（率）	6（11.5%）	6（11.5%）	7（11.3%）	6（9.7%）

* 与干预前比较，$\chi2=3.96$，$p<0.05$

表 8 干预学校与对照学校小学女生贫血、超重和肥胖情况前后比较

	干预学校		对照学校	
	前	后	前	后
贫血人数（率）	14（34.1%）	5（12.2%）*	9（17.3%）	10（19.2%）
超重人数（率）	11（26.8%）	8（19.5%）	11（21.2%）	12（23.1%）
肥胖人数（率）	4（9.8%）	3（7.3%）	4（7.7%）	4（7.7%）

* 与干预前比较，$\chi2=5.55$，$p<0.05$

2.3 干预学校与对照学校小学生头发中无机元素含量以及偏低情况前后比较 （见表 9、表 10、表 11 和表 12）

（中学生干预以及对照后因故未作检测，另外由于部分小学生头发过短，故实际例数为火瓦巷小学学生 91 人，其中男生 50 人，女生 41 人，光华东街小学 110 人，其中男生 58 人，女生 52 人）

表 9 干预学校与对照学校小学男生头发中无机元素含量前后比较

	干预学校		对照学校	
	前	后	前	后
钙（μg/g）	674.5±173.0	708.9±188.5	666.8±171.0	665.7±168.2
铁（μg/g）	29.0±10.9	31.1±12.0	30.6±16.2	30.7±16.2
锌（μg/g）	163.7±15.0	173.5±19.0	169.9±45.2	173.2±45.5
硒（μg/g）	0.60±0.10	0.60±0.11	0.55±0.14	0.57±0.14

经统计学检验，干预学校与对照学校小学男生头发中无机元素含量前后比较差异均无显著性。

表 10 干预学校与对照学校小学女生头发中无机元素含量前后比较

	干预学校		对照学校	
	前	后	前	后
钙（μg/g）	752.6±204.4	770.0±45.8	721.3±157.0	749.6±169.3
铁（μg/g）	30.7±12.2	31.6±12.4	33.0±18.1	34.5±18.3
锌（μg/g）	158.9±22.2	169.4±24.9	179.1±53.5	178.7±55.7
硒（μg/g）	0.56±0.12	0.59±0.14	0.54±0.13	0.53±0.12

经统计学检验，干预学校与对照学校小学女生头发中无机元素含量前后比较差异均无显著性。

表 11 干预学校与对照学校小学男生头发中无机元素偏低比例前后比较

	干预学校		对照学校	
	前	后	前	后
钙	14（28.0%）	10（20.0%）	17（29.3%）	17（29.3%）
铁	9（18.0%）	7（14.0%）	8（13.8%）	6（10.3%）
锌	2（4.0%）	2（4.0%）	2（3.4%）	3（5.2%）

经统计学检验，干预学校与对照学校小学男生头发中无机元素偏低比例前后比较差异均无显著性。

表 12 干预学校与对照学校小学女生头发中无机元素偏低比例前后比较

	干预学校		对照学校	
	前	后	前	后
钙	11（26.8%）	8（19.5%）	10（19.2%）	8（15.4%）
铁	7（17.1%）	5（12.2%）	8（15.4%）	9（17.3%）
锌	1（2.4%）	1（2.4%）	2（3.8%）	2（3.8%）

经统计学检验，干预学校与对照学校小学女生头发中无机元素偏低比例前后比较差异均无显著性。

3 讨论

3.1 干预后学生营养状况有所改善

经过三个月的午餐膳食食谱干预后，各干预组能量与钙的午餐摄入与参考摄入量相比仍然有一定差距，但是前后比较有较大改善，说明膳食午餐干预发挥了一定作用。而对照学校除了小学男生的能

量也有较大改善之外，其他组别都维持在基线水平，差异没有显著性。另外干预组缺乏比较严重的维生素 E、维生素 A 等营养素摄入量也有显著性增加，接近参考摄入量，而相应的对照组都维持在基线水平没有太大变化。说明经过干预研究，午餐质量有所改善。

另外我们也注意到，在干预前后三大热能营养素供热比比例失调依然存在，蛋白质、脂肪和碳水化合物三大物质供热比的推荐比例分别为 12%、20%～30%和 55%～65%，不难看出干预后学生仍然是维持高蛋白饮食，碳水化合物摄入比例普遍偏低，与 Montgomery[8]报道的结果类似。而 Demas[9]研究表明学生乐于改变不良膳食行为，进行低脂肪膳食摄入，同时还能够带动家长改变高脂肪膳食摄入习惯。

3.2 学生身体健康状况干预后有一定好转

经为期三个月的膳食午餐干预，从结果中我们可以看出，各干预组的贫血患病率都有下降，除中学女生组外，其他各组与干预前比较都有显著性差异。而各对照组都维持在基线水平，没有显著性变化。表明膳食午餐干预对改善学生的贫血状况起到了一定的效果。缺铁性贫血是影响世界人民健康，尤其是儿童青少年的主要疾病之一。Samira[10]指出儿童青少年贫血的发生与其母亲的营养知识不足有很大关系，因此建议对儿童青少年母亲进行营养健康宣教，以便更好的降低儿童青少年贫血的发生。

我们从结果中不难看出各干预组的超重以及肥胖状况在干预后有一定的改善，但经统计学检验差异均无显著性，与孙兰[11]报道结果类似。可能原因是由于学习负担的不断加重、活动场所设施的匮乏以及电脑的普及，使得国内中小学生更倾向于静坐的生活方式。同时学生家长的支持是十分重要的，因为学生的早餐及晚餐均由家长支配，特别是对父母依赖性较强的小学生进行肥胖干预时，要更加注重学生家长的培训，调动家长们的积极性，取得他们的支持，以共同完成好干预计划。因此，应该将本次以学校为基础的肥胖干预计划向社区和家庭推广[12]，防治重点放在超重学生，同时尽量增加学生体育运动[13-14]，将有效地控制肥胖学生的增长。另外肥胖的控制还需要注意改善后经常会反弹这问题，Laflamme 等[15]研究表明缓慢的减低体重或者是降低热能的摄入将有效的解决这问题。

对干预学校小学生头发中无机元素进行前后比较可以看出，干预学校小学生头发中四种无机元素含量都有所增加，头发中元素偏低比例均有所降低，但是与干预前比较差异均无显著性，对照学校前后同样没有显著性变化，表明膳食午餐干预以及营养健康教育对降低学生头发中的无机元素偏低比例起到了一定的作用，本研究中学生头发中无机元素的含量没有显著性增加，可能是与两次相隔时间过短有关。

4 参考文献

[1] Tateda, Yukakoa; Kawamura, Taikob; Yoshida, Tomoko.et al.Health education as part of health promotion and prevention of chronic lifestyle diseases in an international cooperation project[J].International Congress Series,2004,1267(complete):51-58

[2] 李申生. 关于学生集体午餐的调查与分析[J]. 湖北预防医学杂志，2002，13，(4)：19-20

[3] 赵渝，赵冰. 上海地区中学生午餐营养状况的调查[J]. 现代预防医学，2003，30（1）：4-5

[4] Michelle M; Zive, R.D; John P. Elder. et al. Sources of dietary fat in middle schools[J]. Preventive Medicine, 2002,35:376–382

[5] 王明萱. 改善学生营养提倡科学饮食[J]. 中国食物与营养，1998，(3)：4

[6] 季成叶. 中国学生超重肥胖 BMI 筛查标准的应用[J]. 中国学校卫生，2004，25（1）：125-128

[7] 季成叶. 中国学生贫血状况的动态观察[J]. 中华预防医学杂志，2002，36(2)：81-83

[8] Montgomery, D.H.a; Kelder, LD, S.H.a; Scaife, B.a. et al. The Effect of A Food Service Intervention (catch Eat Smart) on School Meal Cost. Journal of the American Dietetic Association，1996，96(9)：A09

[9] Antonia Demas. Low-Fat school lunch programs :Achieving Acceptance [J].Am J Cardiol，1998，82：80-82

[10] Gupta, Samira; Venkateswaran, Rajia; Gorenflo, Daniel W.a. et al. Childhood iron deficiency anemia, maternal nutritional knowledge, and maternal feeding practices in a high-risk population. preventive medicine[J] preventive medicine，1999，29，(3)：152-156

[11] 孙兰，姚经建，,夏红等. 闵行区中小学学生单纯性肥胖干预效果评价[J]. 中国公共卫生，2005，2(3)：266-267

[12] Fitzgibbon, Marian L.; Stolley, Melinda R.; Dyer, Alan R..et al. A Community-Based obesity prevention program for minority children: rationale and study design for Hip-Hop to health Jr[J].preventive medicine,2002,34(2):289-297

[13] Baranowski, Tom; Mendlein, James; Resnicow, Ken.et al. Physical activity and nutrition in children and youth: an overview of obesity prevention [J]. preventive medicine，2000，31(2)：1-10

[14] RIPPE, JAMES M.; CROSSLEY, SUELLYN; RINGER, RHONDA.et al. Obesity as a chronic disease: modern medical and lifestyle management [J]. Journal of the American Dietetic Association，1998，98(10)：9-15

[15] Laflamme, DP; Kuhlman, G. The effect of weight loss regimen on subsequent weight maintenance in dogs. [J] Nutrition Research，1995，15(7)：1019-1028

万方数据 WANFANG DATA 文献链接

南京市中小学生营养午餐膳食干预研究

作者： 孙桂菊， 杨立刚， 胡永祯， 杨瑾， 王少康， 罗海燕， 徐华珠， 盛军利， 陈国威， 谢莹
作者单位： 东南大学公共卫生学院营养与食品卫生系,南京,210009

引用本文格式：孙桂菊.杨立刚.胡永祯.杨瑾.王少康.罗海燕.徐华珠.盛军利.陈国威.谢莹 南京市中小学生营养午餐膳食干预研究[会议论文] 2005
