# Supplementary material for: Systematic review and meta-analysis of school-based obesity interventions in mainland China
Source: PLoS One. 2017 Sep 14;12(9):e0184704. doi: 10.1371/journal.pone.0184704 (PMC5598996; doi:10.1371/journal.pone.0184704)
Supplement: S1 Dataset — (ZIP) [file pone.0184704.s007.zip › S1_dataset/76库/37.pdf]

【疾病预防与控制】

虹口区青少年肥胖综合干预措施的研究

蒋骅，杨平，范宏恩，黄慧理，司梅

摘要：[目的]通过综合干预，降低学校学生的肥胖发生率。[方法]选取虹口区A学校和B学校二所九年一贯制学校分别作为干预与对照学校，开展群体性干预研究。对于干预学校实施为期2年综合干预措施，以家庭行为为基础，包括运动训练和饮食控制、健康教育。[结果]干预组学校干预前后的学生肥胖发生率分别为19.84%和13.18%，差异具有显著性；经过综合干预，干预组学校肥胖率下降了33.57%，对照组学校的学生肥胖率上升了3.53%，两校肥胖率的改变存在显著差异。[结论]通过综合干预，可以显著提高学生及家长控制肥胖相关的健康行为形成率，对控制学生肥胖的发生率有一定的效果。

关键词：青少年；肥胖；干预

Study of Comprehensive Intervention on Obesity Youths in Hongkou District, Shanghai *JIANG Hua, YANG Ping, FAN Hong-en, HUANG Hui-li, SI Mei* (*Shanghai Hongkou District Center for Disease & Prevention, Shanghai* 200082, *China*)

Abstract: [Objective] To reduce the prevalence of obesity among students in the school. [Methods] Two nine-year successive schools, School A and School B in Hongkou District were selected to implant the cluster intervention research. They were divided into the intervention school and the control one. The intervention school received two years comprehensive family-based interventions which include exercise training, diet control, and health education. [Results] The prevalence of obesity of the intervention school was 19.84%, and the control school 13.18%. The difference of the prevalence of obesity between the two groups was statistically significant. After the intervention implementation, the obesity rate of the intervention group dropped 33.57%, while the obesity rate of the control school raised 3.53%. Two schools' obesity rate changed different, and the difference was statistical significance. [Conclusions] It was effective to promote the parents' to adopt obesity-control related health behavior through comprehensive intervention implementation, and to control the prevalence of obesity among the students as well.

Key Words: youth; obesity; intervention

青少年肥胖是成年后肥胖和许多慢性疾病的危险因素。2001~2003学年虹口区学生体质健康监测点学校7~14岁学生肥胖率均高于20%，为了对这一状况有所控制，我们拟在虹口区A学校开展以家庭行为为基础，包括运动训练和饮食控制、健康教育在内的干预措施，希望可形成一套行之有效的、可操作的肥胖综合防治措施。

1 对象与方法

1.1 对象

干预学校：A学校共有学生1 633名，其中男生841名、女生792名；学生年龄分布于5~16岁。

对照学校：B学校共有学生787名，其中男生404名，女生383名；学生年龄分布于6~17岁。

1.2 方法

1.2.1 干预措施 这两所学校的体制均为九年一贯制，通过均衡性评价两校的学生在性别、年龄构成上没有显著差异，健康教育、体育锻炼均按照教委大纲统一布署，两校都有自办食堂（责任承包制），基本情况无明显差异，可在此基础上开展干预。从2003年9月至2005年6月，对A校实施干预，对B校不采取任何的干预措施。

首先对两所学校学生进行身高、体重的测量，筛检出A校的肥胖儿童作为重点干预对象，向每位肥胖儿童的家长下发一份告知书有关各项控制措施，并由家长签署同意配合的回执；组建以班级为单位的体锻小组。具体干预措施如下：

1.2.1.1 健康宣教 由课题组提供相关资料及宣传版面，组织多样形式的宣教活动，如每周20分钟的学校卫生广播、向全校师生分发对不同年龄段各有宣传侧重点的合理膳食资料、以班级为单位进行营养知识版面巡展、针对肥胖儿童及其家长接受营养知识的侧重面不同邀请营养专家对肥胖学生和家长作营养知识的讲座等。

1.2.1.2 膳食干预 课题组对校方食堂进行三日膳食营养分析，根据《中国居民平衡膳食宝塔》及《中国居民膳食营养素参考摄入量》及时调整膳食结构，并提供营养菜谱，供其自由选择。

1.2.1.3 体育锻炼 每个体锻组组长负责督促组内同学每日在课外时间内（包括双休日）进行多种形式的体育锻炼，要求一周至少锻炼4次，每次30~60分钟，心率应达到每分钟150次。

1.2.1.4 假期干预 为了使学校中的干预效果在假期内得以持续，在寒、暑假中课题组与社区、教育、科协等部门合作在假期中也组织一系列的干预延续活动。

作者简介：蒋骅（1978），女，本科，学士，医师，研究方向：学校卫生
作者单位：上海市虹口区疾病预防控制中心，上海 200082

1.2.2　身高、体重确定　按《学校卫生工作指南》操作程序[1]测定。

1.2.3　营养评定标准　采用WHO推荐的身高标准体重法评估肥胖程度[2]。体重在标准体重的91%~110%为正常，111%~120%为超重，高于120%为肥胖，低于91%为营养不良。

1.2.4　膳食调查　对A学校进行膳食调查。采用称重法与计帐法相结合，共调查3 d，将食物重量（净食用部分）经电脑营养计算程序处理后计算出该校学生平均每日食物构成和各种营养素摄入量，并与《中国居民平衡膳食宝塔》及《中国居民膳食营养素参考摄入量》（DRIs）相比较[3]。

2　结果与分析

2.1　营养状况

干预期前两校学生肥胖率的差异有显著性（$\chi^2=4.804$，$P<0.05$），A校学生的肥胖率显著高于B校；两校学生的男女生肥胖率也有显著差异（$\chi^2_{A校}=17.966$，$P<0.05$；$\chi^2_{B校}=9.389$，$P<0.05$）（表1），男性学生的肥胖率均高于女性。

表1　干预期前两校学生肥胖情况比较

性别	A学校（干预学校）			B学校（对照学校）		
	检测人数	肥胖人数	肥胖率%	检测人数	肥胖人数	肥胖率%
男	841	201	23.90	404	81	20.05
女	792	123	15.53	383	46	12.01
合计	1 633	324	19.84	787	127	16.14

2.2　膳食调查

A学校食堂每周制定一次菜谱，能基本做到荤素、米面、干稀的搭配，从食物结构来看粮谷类与蔬菜类不足，动物性食品和植物油过高，从而导致能量摄入过高，钙、锌及部分维生素（维生素B1、B2和维生素C）摄入严重不足。

2.3　干预效果

对A校进行了为期二年的综合干预，以家庭行为为基础，包括运动训练、饮食控制和健康教育。为了检验干预的效果，课题组成员分别在04年9月和05年6月对A学校进行了两次身高体重检测，结果如下：

表2　干预期前后A学校资料比较

项目	性别	年级	检测人数	肥胖人数	肥胖率%
基线资料	男	1~8	841	201	23.90
	女	1~8	792	123	15.53
干预中期	男	2~9	839	158	18.83
	女	2~9	792	92	11.62
干预终期	男	2~9	839	137	16.33
	女	2~9	792	78	9.85

合计共107名肥胖儿童跳出肥胖行列［其中男生62名（201-137-2），有2名肥胖男生转学；女生45名（123-78）］。干预期结束时A学校学生1 631名（2名转校），肥胖215名，肥胖率13.18%，比干预前下降了6个多百分点。

我们将干预后A学校与B学校的同期资料进行了比较，结果见表3：

表3　干预期后两校的学生肥胖情况比较

性别	A学校（干预学校）			B学校（对照学校）		
	检测人数	肥胖人数	肥胖率%	检测人数	肥胖人数	肥胖率%
男	839	137	16.33	410	82	20.00
女	792	78	9.85	344	44	12.79
合计	1 631	215	13.18	754	126	16.71

从上表我们可以看到A学校在干预前后肥胖率有了显著的下降，而B学校没有很大的变化；而且在干预后A学校的学生肥胖率显著低于B学校（$\chi^2=5.240$，$P<0.05$），结合在干预前A学校的学生肥胖率高于B学校这一情况，可以认为我们的干预措施是有一定效果的。

3　讨论

“儿童肥胖的干预”属于一个世界性的难题，迄今没有行之有效的解决方案，目前国内外很少有干预效果明显的报道，而此次我们的措施使干预学校的肥胖发生率有所下降，可能与以下几方面原因有关。

首先，在干预措施实施之前，我们对肥胖学生及其家长做了有关科学营养膳食和运动方面的预调查，发现学生及家长对儿童肥胖与成年期慢性病的关系、正常体重判定标准、适度体育锻炼、合理膳食的知晓率均达80%以上，可是由于体型肥胖或害怕被人嘲笑等原因肥胖儿童的运动时间和积极性还是明显少于体重正常儿童。根据调查结果，我们在干预过程中将肥胖学生参与干预活动的自觉自愿性放在首位，用自我管理的方法提高其参与干预活动的积极性和持久性，可避免因为其他同学的嘲笑而产生自卑心理、抵触情绪，从家庭到学校形成了一个较好的氛围，使我们的措施能顺利开展。

其次在膳食干预中，我们为A学校学生膳食做了营养分析，结果显示食物结构多样，但谷薯类、蔬菜类含量过低，动物性食品过多，烹饪过程中用油太多，导致脂肪产能比过高，随着生活改善，这样的饮食习惯也普遍存在于绝大多数家庭中。故一方面我们向学校食堂提出了调整菜谱的建议，如适量使用油脂、增加蔬菜量的供应、以鱼虾禽肉来代替畜肉，并提供营养菜谱以做参考；另一方面我们邀请肥胖儿童及其家长一同接受营养专家有关肥胖儿童科学膳食的讲座，让家长共同参与饮食控制。

在以往的调查研究中，显示儿童与其父母的体质指数BMI有很强的相关性，说明肥胖受遗传和家庭成员共同生活习惯的影响比较明显。由于成人的生活习惯、行为方式是他们成长过程中逐渐形成的[4]，与其被迫试图改变早已建立的习惯不如早期预防那些有害健康行为的形成[5]，因此我们把促进家庭内的健康行为的有效形成和长久保持以及营造社区健康氛围的支持环境也作为了这次干预的一个重点，从学校－家庭－社区三个环节全面涵盖了肥胖儿童生活学习的各个层面。在家庭中，家长积极配合做到各项控制措施；在社区中，我们建立了一支假期志愿者队伍，负责督促肥胖儿童的体育锻炼，以便使学校中的干预效果在暑寒假期内得以持续。

而且在进行干预中期评估时我们看到一方面综和干预措施对于肥胖儿童的体重控制是有成效的，但是另一方面我们不

能仅仅只把基线资料中的324名肥胖儿作为干预对象，新增的肥胖儿童除了新转入生外，有相当一部分是由原来的超重儿甚至体重正常儿童转变而来的，故应把全体学生同时纳入宣教干预措施下，不光要看见肥胖学生的好转，更要注意降低肥胖的新增率，以此来达到降低全体肥胖率的目的。因此在干预实施过程中我们及时的把新增的肥胖儿童也编入体锻小组，在评估中也特意的统计了这部分新增肥胖儿童的动向，结果我们发现在经过了一阶段的体育锻炼，男生有38名、女生有6名跳出了肥胖行列。

由于本次课题得到了学校、社区、家庭和肥胖学生本人的支持，再者干预对象为正处于生长发育高峰期的青少年，控制肥胖相关的健康行为形成率的提高体现在肥胖的控制上是有一定效果的。但是社会对教育的偏见、对教学质量评估的片面理解、一味追求升学率等，使部分家长和老师不能十分积极持久的实施控制儿童肥胖的相关措施，而且由于当今独生子女大都是家庭的核心，学校、社区等部门在组织学生开展文体活动时，担心意外伤害事故的发生，都心存顾虑，因此预防青少年肥胖需要学校、家庭、社会的共同努力，否则前景堪忧。

参考文献：

[1]贝品联.学校卫生工作指南.第1版.上海：上海科学技术文献出版社，2000：57-59.

[2]贝品联.学校卫生工作指南.第1版.上海：上海科学技术文献出版社，2000：336-341.

[3]中国营养学会.中国居民膳食营养素参考摄入量.中国学校卫生，2002，23(3)：4.

[4]Smith C, Roberts C, Nutbeam D, et al. The Health Promoting School: Progress and Future Challenges in Welsh Secondary Schools[J]. Health Promotion International, 1992, 7(7): 171-179.

[5]Alexander D. Adolescents and Young Adults: Overview[J].Preventive Medicine, 1994, 23: 653-654.

万方数据 WANFANG DATA 文献链接

虹口区青少年肥胖综合干预措施的研究

作者： 蒋骅， 杨平， 范宏恩， 黄慧理， 司梅
作者单位： 上海市虹口区疾病预防控制中心,上海 200082

本文读者也读过(10条)

1. 蒋竞雄.吴光驰.夏秀兰.谈藏文.郭素怡 儿童肥胖学校群体干预三年追踪观察[会议论文]-2003
2. 戴荣明.沈蕙.滕臣刚.李海.潘晓群.史祖民 苏州市青少年肥胖状况调查[期刊论文]-医学动物防制2007,23(3)
3. 李慧.蒋丽娟.常小芳.王文艺 深圳市以肥胖控制为切入点发展健康促进学校项目中期效果评价[期刊论文]-中国健康教育2004,20(6)
4. 刘素芹.张清华.岳亿玲.李玉芹.LIU Su-Qin.ZHANG Qing-hua.YUE Y-ling.LI Yu-qin 儿童单纯肥胖症群体综合干预研究[期刊论文]-中国校医2005,19(2)
5. 陈健.CHEN Jian 厦门市健康促进学校控制儿童肥胖项目效果评价[期刊论文]-海峡预防医学杂志2006,12(5)
6. 田本淳.吕书红.钱玲.张巍.张继彬 我国四城市部分小学生肥胖控制效果评价[期刊论文]-中国学校卫生2006,27(10)
7. 罗春燕.彭宁宁.冯小刚.朱佳佩 2004～2008年上海市青少年肥胖相关饮食行为分析[会议论文]-2008
8. 任蓓麟 上海市某中学青少年肥胖的病因研究[会议论文]-2002
9. 徐英.李晓雯.徐耘.周秀丽.冯伟.秦万芬 青少年肥胖群体干预模式的探讨[期刊论文]-中国慢性病预防与控制2004,12(1)
10. 蒋竞雄.夏秀兰.吴光驰.谈藏文.宋小芳.王力.郭素怡.闫桂风 学龄儿童单纯肥胖症的群体干预研究[期刊论文]-中国儿童保健杂志2002,10(6)

引用本文格式：蒋骅.杨平.范宏恩.黄慧理.司梅 虹口区青少年肥胖综合干预措施的研究[会议论文] 2007
