# Supplementary material for: Systematic review and meta-analysis of school-based obesity interventions in mainland China
Source: PLoS One. 2017 Sep 14;12(9):e0184704. doi: 10.1371/journal.pone.0184704 (PMC5598996; doi:10.1371/journal.pone.0184704)
Supplement: S1 Dataset — (ZIP) [file pone.0184704.s007.zip › S1_dataset/76库/38.pdf]

【健康教育】

"快乐 10 min"活动预防儿童肥胖效果观察

高爱钰[1],潘勇平[1],石晓燕[1],崔朝晖[2]

【摘要】 目的 探索儿童肥胖的群体干预效果,为进一步控制儿童肥胖提供依据。方法 采用整群抽样,从北京市东城区选择 10所小学,以二~四年级学生为研究对象,其中 5所学校为干预组(1 157人),5所学校为对照组(1 060人)。干预组进行"快乐 10 min"活动,包括健康教育、增加运动、减少静坐时间等为期 1年的群体干预,对照组除与干预组同期体检外不接受任何干预措施。结果 干预后,2组学生的形态指标均因学生的发育而升高,但与肥胖评价有关的指标如体重、BMI、腰围、皮脂厚度的增加干预组低于对照组。干预组男生肥胖增长速度比对照组低 3.65%;女生超重、肥胖增长速度较对照组分别低 35.80%和 2.09%。干预组肥胖者中有 80.2%继续维持肥胖状态,19.8%转变为非肥胖状态;对照组肥胖者中有 82.1%继续维持肥胖状态,17.9%转变为非肥胖状态,但差异未达到显著性水平。结论 "快乐 10 min"活动对超重、肥胖儿童的干预具有一定的效果,但不显著。肥胖干预应全社会共同参与,从低龄儿童抓起。

【关键词】 肥胖症;体型;干预性研究;综合预防;儿童

【中图分类号】 R 179 G 479 【文献标识码】 A 【文章编号】 1000-9817(2008)11-0978-02

Effect of "Happy 10 minutes" for Preventing Childhood Obesity/*GAO Ai-yu, PAN Yong-ping, SHI Xiao-yan, et al. Dongcheng School Health Care Institute, Beijing(100007), China*

【Abstract】 **Objective** To explore the effect of population-based intervention for childhood obesity and provide evidence for the further control. **Methods** The students of 2 - 4 grade were selected by cluster sampling from 10 primary schools of Dongcheng district of Beijing, of which 5 schools(1 157 students) were classified into the intervention group, the other 5 schools(1 060 students) as the control group. The intervention lasted for one year, including health education, increasing physical exercises, reducing sedentary time. **Results** After one year of intervention, although the levels of bodily form indices of the students in 2 groups were elevated along with their development, the levels of obesity-related indices such as weight, body mass index, waist and skinfold thickness of the intervention group increased less than those of the control group. The increase of obesity rate in male students of the intervention group was less than that of the control group by 3.65%. And the increases of overweight and obesity rates in female students of the intervention group were lower than those of control group by 35.80% and 2.09%, respectively. In the intervention group, 80.2% obese students remained obese, and 19.8% turned to being overweight or normal. And in control group, 82.1% obese students remained obese, and 17.9% turned to being overweight or normal. **Conclusion** "Happy 10 minutes" has certain effects on intervention of childhood overweight and obesity, but not significant. Obesity intervention requires participation of the whole society and should start among early-aged children.

【Key words】 Obesity; Somatotypes; Intervention studies; Universal precautions; Child

儿童肥胖无论在发达国家,还是发展中国家都呈迅速上升趋势,已成为全球瞩目的公共卫生问题。根据 WHO报告,目前全球儿童超重率接近 10%,肥胖率为 2%~3%;欧美发达国家儿童超重率高达 20%~30%,肥胖率为 5%~15%。我国儿童肥胖检出率也正在逐年增长,7~22岁城市学生超重、肥胖检出率男生由 1995年的 10.15%上升到 2005年的 24.64%,城市女生由 8.03%上升到 13.74%[1]。儿童时期是肥胖形成的关键时期,是生活方式和饮食习惯形成的时期,也是预防肥胖的最好时机。因此,本研究自 2005年在北京市东城区 10所小学开展以学校为基础的肥胖干预活动,探索儿童肥胖的群体干预方法。

1 对象与方法

1.1 对象 采用整群抽样方法,从北京市东城区抽取办学条件、师资力量基本相似的 10所小学的二~四年级学生为研究对象,干预学校和对照学校各 5所。共调查学生 2 217名,其中干预组 1 157名(男生 594名,女生 563名),对照组 1 060名(男生 547名,女生 513名)。

1.2 方法

1.2.1 身体测量及超重、肥胖、高血压的判定 对所有研究对象进行身高、体重、肺活量、腰围、臀围、皮脂厚度、握力、血压等测量。身体测量方法按照《2005年中国学生健康与体质调研检测细则》进行,并严格进行质量控制,根据《中国学龄儿童青少年超重肥胖 BMI筛查分类标准》判定超重和肥胖[2]。

1.2.2 干预方法 干预组进行为期 1学年的群体干预,对照组除与干预组同期体检外不接受任何干预措施。干预措施包括:(1)体力活动干预:持续 1学年的每天 2次"快乐 10 min"活动("快乐 10 min"是一种内容丰富、趣味性强、活动形式多样且适合学生特点的活动形式,不受场地和设施的限制),实施过程受中国疾病预防控制中心、有关保健所及校医会的监督;限制儿童看电视时间和使用电脑时间每天在 1 h以内;干预学校每周多 1 h的户外活动。(2)健康教育:对学生、家长进行膳食、肥胖与健康的教育。

1.2.3 统计方法 使用 EpiData软件建立数据库,SPSS 11.5统计软件进行分析,并采用 χ^2 检验。

2 结果

2.1 干预前后 2组学生形态指标变化情况 干预前干预组与对照组学生的身高、体重、BMI、腰围、臀围、皮脂厚的均值比较,差异均无统计学意义(P值均 >0.05)。经过 1年的干预活动,2组学生的形态指标均因学生的发育而升高,但与肥胖评价有关的指标,如体重、BMI、腰围、皮脂厚度的增加干预组低于对照组,但差异均无统计学意义(P值均 >0.05)。见表 1。

【作者简介】 高爱钰(1967-),女,山东烟台人,大学本科,主治医师,主要从事学校卫生工作。

【作者单位】 1 北京市东城区中小学卫生保健所,100007;
2 中国疾病预防控制中心营养与食品安全所。

© 1994-2010 China Academic Journal Electronic Publishing House. All rights reserved. http://www.cnki.net

表 1 干预前后两组学生形态指标变化情况比较 ($\bar{x}\pm s$)

组别	时间	人数	身高 /cm	体重 /kg	BMI/(kg·m⁻²)	腰围 /cm	臂围 /cm	肱三头肌皮褶厚度 /mm	肩胛下皮褶厚度 /mm
干预组	干预前	1 157	140.35 ±7.18	35.29 ±9.57	17.72 ±3.67	61.87 ±9.98	74.31 ±28.43	15.79 ±6.92	13.20 ±9.29
	干预后	1 157	146.23 ±7.71	39.41 ±10.97	18.22 ±3.88	64.44 ±10.70	81.03 ±56.90	18.08 ±8.02	14.64 ±10.19
	增值		5.88	4.12	0.50	2.57	6.72	2.29	1.44
对照组	干预前	1 060	140.82 ±7.53	35.48 ±10.04	17.66 ±3.63	62.31 ±10.15	74.08 ±8.31	15.54 ±6.94	13.11 ±9.46
	干预后	1 060	146.49 ±8.32	40.05 ±11.50	18.42 ±3.88	64.92 ±10.86	79.53 ±42.80	18.66 ±8.35	14.69 ±10.41
	增值		5.67	4.57	0.76	2.61	5.45	3.12	1.58

2.2 干预前后学生超重、肥胖检出情况变化比较 干预前后学生超重、肥胖检出情况变化见表 2。干预前干预组男生超重、肥胖检出率分别为 11.4%和 21.7%,对照组男生超重、肥胖检出率分别为 12.4%和 21.9%。干预 1年后,干预组男生超重检出率上升了 2.9个百分点,增长速度为 25.43%;肥胖检出率下降了 2.9个百分点,增长速度为 -13.24%。对照组男生超重检出率上升了 1.4个百分点,增长速度为 11.29%;肥胖检出率下降了 2.5个百分点,增长速度为 -9.59%。差异均无统计学意义(P值均 >0.05)。干预组肥胖增长速度比对照组低 3.65%。

干预前干预组女生超重、肥胖检出率分别为 12.8%和 10.0%,对照组女生超重、肥胖检出率分别为 8.6%和 10.3%。干预 1年后,干预组女生超重检出率下降了 2.5个百分点,增长速度为 -19.52%;肥胖检出率下降了 0.5个百分点,增长速度为 -5.00%。对照组女生超重检出率上升了 1.4个百分点,增长速度为 16.28%;肥胖检出率下降了 0.3个百分点,增长速度为 -2.91%。干预组超重、肥胖增长速度较对照组分别低 35.80%和 2.09%。

表 2 干预前后 2组学生超重肥胖检出情况变化比较 /%

干预前后	男生干预组		男生对照组		女生干预组		女生对照组	
	超重	肥胖	超重	肥胖	超重	肥胖	超重	肥胖
干预前	11.4	21.7	12.4	21.9	12.8	10.0	8.6	10.3
干预后	14.3	18.8	13.8	19.8	10.3	9.5	10.0	10.0
χ^2值	2.064	1.499	0.414	0.750	1.664	0.062	0.559	0.038
P值	0.151	0.221	0.520	0.386	0.197	0.804	0.455	0.846

2.3 干预前后学生营养状况变化情况比较 干预前后学生营养状况变化情况见表 3。经过 1年干预后,干预组肥胖者中有 80.2%继续维持肥胖状态,19.8%转变为非肥胖状态;对照组肥胖者中有 82.1%继续维持肥胖状态,17.9%转变为非肥胖状态。干预组、对照组在干预后非肥胖者转为肥胖和继续维持非肥胖状态比例基本一致。

表 3 干预前后 2组学生营养状况变化情况比较

组别	肥胖者(n=172)		非肥胖者(n=895)	
	维持肥胖	转为非肥胖	维持肥胖	转为非肥胖
干预组	138(80.2)	34(19.8)	15(1.7)	880(98.3)
对照组	133(82.1)	29(17.9)	13(1.6)	798(98.4)

注:()内数字为百分率 /%。

3 讨论

1997年世界卫生组织明确指出,肥胖是一种可怕的疾病,已成为严重的社会问题,是现代生活中的流行病和 21世纪威胁人类健康的"温柔杀手"。高血压、高血脂、糖尿病、冠心病、脑血管病等都与肥胖有密切关系。肥胖对儿童青少年的危害首先是生理危害,表现为体态臃肿、行动不便、容易疲劳等;其次是对学习能力的不良影响,肥胖越严重,对孩子的认知和学习能力损害越大;三是心理行为问题增加,表现为自卑、自暴自弃、暴饮暴食等;四是为高血压、糖尿病等慢性疾病的发生埋下隐患[2]。因此,及早对超重、肥胖儿童采取干预措施,减少对儿童的身心健康影响,具有深远的社会意义。

通过为期 1年的以学校为基础、家庭、儿童共同参与的体力活动与健康教育相结合的干预,收到了一定的效果。结果显示,干预组学生体重、BMI值、腰围、皮脂厚度增长低于对照组,干预组超重、肥胖增长速度低于对照组,尤其是男生的肥胖检出率下降更明显,女生超重增长速度干预组比对照组低 35.8%。在干预过程中,干预组肥胖、超重增长速度均比对照组增长速度慢,说明本次干预具有一定的效果。据蒋竞雄等[3]研究显示,经过 3年干预,干预组肥胖检出率从 16.9%降至 12.1%,对照组肥胖检出率从 17.4%上升为 23.2%。本次干预虽然有一定效果,但不显著的原因可能有:一是干预时间相对短;二是干预人群选择在小学二~四年级,1年后进入三~五年级,而小学四~六年级是儿童肥胖的高发阶段[4],在此阶段进行肥胖干预不是最佳时期,从低年龄抓起,效果可能更好;三是肥胖干预是个系统工程,影响因素多,难度比较大。

肥胖的群体干预与临床治疗最大的区别在于覆盖人群范围不同[3]。本研究不但针对肥胖儿童,而且将非肥胖儿童(尤其是超重儿童)纳入干预的实施对象之中,以减少肥胖新增人数。对干预组儿童进行追踪观察,干预组原来肥胖者中有 80.2%继续维持肥胖状态,非肥胖学生中有 1.7%变为肥胖。对照组原来肥胖者中有 82.1%继续维持肥胖状态,非肥胖学生中有 1.6%变为肥胖。干预组、对照组在干预后非肥胖者转为肥胖和继续维持非肥胖状态比例基本一致。

儿童少年处于生长发育的旺盛时期,要在其身高、体重不断增长中控制向肥胖发展,不宜提倡"限食"措施,且儿童对膳食、生活方式的自控能力较低,需要更多依靠父母、家庭、社会引导和教育[5]。采用体力活动,势必成为安全而有实效的关键措施。本研究所采用的"快乐 10 min"活动,是一种很好的体力活动方式,但关键需要持之以恒。

中国儿童青少年超重、肥胖趋势明显。关心青少年的健康成长,不但关系到每个家庭的幸福,更重要的是关系到国家的富强、民族的兴旺。预防肥胖是一场需要全社会共同参与的持久战,不仅学校、家庭、社区应该参与,政府、保健、食品业和媒体应共同参与,防御儿童肥胖的蔓延,让青少年远离肥胖,走向健康。

4 参考文献

[1] 马军. 儿童代谢综合征. 中国学校卫生, 2006, 27(10): 830.

[2] 季成叶. 中国学龄儿童青少年超重、肥胖 BMI筛查分类标准. 中国学校卫生, 2004, 25(1): 125-128.

[3] 蒋竞雄. 学龄儿童单纯肥胖症的群体干预研究. 中国儿童保健学杂志, 2002, 10(6): 364-367.

[4] 高爱钰, 潘勇平. 北京市东城区 1997-2002年学生肥胖情况. 中国学校卫生, 2005, 26(2): 121-122.

[5] 陈春明, 李艳萍, 马冠生. 儿童肥胖的防治: 从美国的经验教训谈起. 国外医学: 卫生分册, 2006, 33(5): 183-185.

(收稿日期: 2008-09-24)

© 1994-2010 China Academic Journal Electronic Publishing House. All rights reserved. http://www.cnki.net
