# Supplementary material for: Systematic review and meta-analysis of school-based obesity interventions in mainland China
Source: PLoS One. 2017 Sep 14;12(9):e0184704. doi: 10.1371/journal.pone.0184704 (PMC5598996; doi:10.1371/journal.pone.0184704)
Supplement: S1 Dataset — (ZIP) [file pone.0184704.s007.zip › S1_dataset/76库/39.pdf]

【疾病控制】

我国四城市部分小学生肥胖控制效果评价

田本淳, 吕书红, 钱玲, 张巍, 张继彬

【摘要】 目的　探讨学校群体活动模式控制学生肥胖的效果, 为有关干预模式的研究提供依据。方法　在长春、乌鲁木齐、西安和厦门四城市各选择 1 所学校作为干预学校, 三、四年级全体学生为重点干预对象, 选取同一城区条件基本相近的 1 所学校作为对照学校。将肥胖控制与健康促进学校的创建结合起来, 采取以群体干预与个别指导相结合、知识传播与运动/饮食行为干预相结合、学校干预与家庭干预相结合、学生行为改变和学校健康环境改变相结合的综合策略开展肥胖干预。干预前后进行问卷调查。结果　干预学校学生肥胖控制知晓率、正确膳食及运动行为形成率的提高幅度高于对照学校学生, 差异具有统计学意义($P<0.01$); 干预学校肥胖率和超重率分别从 15.6%和 14.1%下降到 10.4%和 14.2%; 其中干预学校三、四年级男生肥胖率从 21.5%下降到 13.6%。结论　学校群体活动控制模式对于小学生的肥胖控制是有效的。

【关键词】 肥胖症; 干预性研究; 学生; 卫生促进
【中图分类号】 R 179　G 478.2　R 589.2 【文献标识码】 A 【文章编号】 1000-9817(2006)10-0869-03

Impact Evaluation on Obesity Control Among Primary School Students in 4 Cities in China/ *TIAN Ben-chun, LV Shu-hong, QIAN Ling, et al. National Health Education Institute, Chinese Center for Disease Control and Prevention, Beijing*(100011), *China*

【**Abstract**】 **Objective**　To probe into the impact of school-based obesity control, and to provide evidence for the prevention model. **Methods**　One school was determined as project school in Changchun, Urumuqi, Xi' an and Xiamen respectively. All the students in grade three and four in these four schools were taken as intervention population. Another four schools in these four cities were selected to be control schools. Strategies were integrated obesity control into the health promoting school establishing, combined group intervention with individual intervention, communicating knowledge with dietary and physical activity intervention, working together with family and by improving the physical environment to promote the students' health behavior. Questionnaire survey was conducted before and after the intervention. **Results**　The increasing of awareness and positive behavior of dietary and physical activity in four project schools was much higher than control schools ($P<0.01$). The prevalence rates of obesity and overweight were declined from 15.6% and 14.1% to 10.4% and 14.2%, especially among boys in grade three and four in project schools, the obesity rate was decreased from 21.5% to 13.6%. **Conclusion** The strategies model of group activity are effective to obesity control among primary school students.

【**Key words**】 Obesity; Intervention studies; Students; Health promotion

近 10 a 来我国儿童青少年肥胖率迅速上升, 2000 年 7～18 岁年龄组男女学生肥胖检出率较 1985 年迅速增加, 远远高于发达国家儿童青少年肥胖的增长速度。且进入青春期后(10 岁以后), 肥胖的检出率和增长速度均达到较高水平。如果这种趋势不能得到有效控制, 到 2010 年我国儿童青少年肥胖率将达到 18.46%(男)和 9.18%(女), 甚至超过发达国家儿童青少年肥胖率的水平[1]。为探讨学校群体活动模式控制学生肥胖的效果, 笔者自 2004 年 3 月起在长春、乌鲁木齐、西安和厦门四城市的 4 所小学中开展了以肥胖控制为切入点发展健康促进学校的肥胖干预活动。结果报道如下。

1　对象与方法

1.1　对象　在长春、乌鲁木齐、西安和厦门四城市分别选择学生肥胖率在 10%以上的白山小学、十三小、莲湖小学和厦门外国语学校作为干预学校, 重点干预对象为三、四年级全体学生, 干预前调查学生 1 728 名, 年龄为(9.84±1.10)岁; 干预后调查学生 1 701 名, 年龄为(10.84±0.96)岁。分别在同一城区选择学校规模和学生肥胖率等条件基本相近的 1 所学校作为对照学校, 干预前调查学生 1 911 名, 年龄为(9.49±1.41)岁; 干预后调查学生 2 179 名, 年龄为(10.65±1.05)岁。

1.2　干预策略

1.2.1　以群体干预和个别指导相结合　在全面开展教育和干预的同时, 特别针对肥胖和超重学生进行有针对性的干预, 如建立超重肥胖档案, 加强对超重肥胖学生每月体检检测工作; 鼓励并组织超重和肥胖学生参加集体活动, 增加体育锻炼时间, 提高运动的质量, 进行每日运动量的监测; 填写《学生个人管理手册》, 及时了解肥胖和超重学生的能量摄入和消耗情况, 由责任老师对这些学生的饮食情况和运动情况给予具体指导。

1.2.2　知识传播和运动饮食行为干预相结合　利用广播、宣传栏等宣传肥胖危害方面的知识, 邀请有关专家向学生家长讲授肥胖控制的相关知识, 并针对肥胖学生在运动和饮食方面存在的问题开展干预活动。

1.2.3　学校干预和家庭干预相结合　学校制定相应的肥胖控制干预策略, 如增加肥胖及超重学生的运动时间、组织以增加运动为主要内容的健康促进小组等; 要求各科教师将肥胖控制知识与各自课程内容有机结合; 通过座谈会、家长会、给家长的一封信等多种形式的活动, 形成家校合作, 共同对肥胖学生进行运动和饮食的调控, 尤其注重假期家庭在肥胖控制中的作用, 充分利用假期开展多种干预活动, 预防假期里体重反弹。

1.2.4　学生行为改变和学校健康环境改变相结合　改善体育设施和扩大运动场所, 创造各种运动场所, 为学生增加运动提供环境支持。

1.3　调查方法　干预实施前后分别对四城市干预和对照学校三、四年级全体学生(实施 1 a 后的四、五年级)进行问卷调查和身高体重测量。采用统一编制的问卷, 由课题城市负责人及经过培训的健康教育专业人员组织学生填写, 检查无遗漏后收回。学生填写的内容主要包括: 膳食营养、运动等肥胖控制相关的知识、态度和行为; 身高和体重由经过培训的校医或有关人员按照统一要求测量。超重和肥胖的判定标准采用中国学龄儿童青少年超重、肥胖筛查 BMI 分类标准[2]。

1.4　数据录入和统计方法　回收问卷由专人核查后, 用 EPI Data 2.0 录入, 经逻辑检错后, 用 SPSS 11.5 软件包统计分析。

【作者简介】 田本淳(1946—　), 男, 湖北大悟人, 大学本科, 研究员, 主要研究方向为健康教育。
【作者单位】 中国疾病预防控制中心健康教育所, 北京　100011。

?1994-2015 China Academic Journal Electronic Publishing House. All rights reserved.　http://www.cnki.net

1.5 质量控制 首先由国家级课题组对4个干预城市项目负责人和学校负责人员进行统一培训，使其掌握实施方案，并由各责任单位完成对具体实施调查工作的人员的培训。由接受培训的人员到学校和班级进行调查，调查前由班主任集体签署知情同意书。并在班主任和教师不在场的条件下进行调查。调查完毕收表前由调查员对调查表进行核查。接受调查学生的身高体重由项目所在地的学校卫生保健所负责组织人员统一进行测量。

2 结果

2.1 肥胖控制相关知识 第1次调查对照学校学生除"蔬菜提供的营养素"、"每天合适的运动量"和"控制体重的正确措施"的知晓率低于干预学校、差异有统计学意义外，其余知识知晓率与对照学校差异无统计学意义。1 a后的调查结果显示，干预学校和对照学校的学生对肥胖控制相关知识的知晓率均有明显提高，干预前后差异具有统计学意义($P<0.01$)，但干预学校学生的相关知识知晓率均高于对照学校学生，干预学校学生知晓率的提高幅度高于对照学校学生，差异有统计学意义($P<0.01$)。见表1。

表1 四城市干预和对照学校学生控制肥胖相关膳食、运动知识干预前后知晓率比较 η/%

相关知识	干预学校		对照学校	
	干预前	干预后	干预前	干预后
正确的食物结构	2.0	44.7**	0.2	3.2**△△
蔬菜提供的营养素	19.6	78.0**	11.5△	29.3**△
多吃甜食对身体有害	89.9	98.6**	86.3	94.9**△
吃多油食物对身体有害	85.9	98.2**	84.3	95.6**△
洋快餐的营养特点	0.9	42.9**	0.7	2.1**△△
吃得过饱对身体的影响	27.7	88.5**	26.0	47.0**△△
每天合适的运动量	60.1	95.4**	47.1△△	70.6**△△
肥胖对健康的影响	5.3	79.5**	4.5	11.9**△△
控制体重的正确措施	21.1	62.8**	11.1△△	22.0**△△

注：干预前后比较，** $P<0.01$；干预校与对照校比较，△ $P<0.05$，△△ $P<0.01$。

2.2 肥胖控制相关态度 第1次调查干预学校和对照学校在肥胖控制正确态度持有率上差异无统计学意义。第2次调查显示干预学校和对照学校学生均有明显提高，但干预学校提高幅度高于对照学校，差异有统计学意义($P<0.01$)。见表2。

表2 四城市干预和对照组学校学生肥胖控制正向态度持有率干预前后比较 η/%

相关态度	干预学校		对照学校	
	干预前	干预后	干预前	干预后
愿意学习营养方面的知识	90.7	98.8**	90.1	94.5**△
愿意参加运动锻炼	93.3	99.4**	92.0	96.1**△
愿意放弃不健康的食品	85.7	97.7**	87.5	93.2**△
愿意吃不爱吃但益于健康的食品	83.2	96.4**	84.9	89.6**△△
愿意每天锻炼身体	81.7	98.5**	80.4	84.3**△△

注：干预前后比较，** $P<0.01$；干预校与对照校比较，△ $P<0.05$，△△ $P<0.01$。

2.3 肥胖控制相关行为

2.3.1 膳食行为 活动实施1 a后，干预学校学生正确饮食行为形成率均大幅度提高，干预前后差异有统计学意义($P<0.01$)，而对照学校尽管也有明显提高($P<0.01$)，但提高幅度小于干预学校。第2次调查结果显示目前干预学校学生正确膳食行为形成率高于对照学校，差异有统计学意义($P<0.01$)。见表3。

2.3.2 运动等相关行为 干预学校学生运动等相关行为正确率明显提高，干预前后差异有统计学意义($P<0.01$)，而对照学校干预前后差异无统计学意义($P>0.05$)。见表3。

表3 四城市干预和对照学校学生肥胖控制相关正确行为具有率干预前后比较 η/%

相关行为	干预学校		对照学校	
	干预前	干预后	干预前	干预后
平时吃得最多的为米面制品	35.5	93.8**	39.9	54.7**△△
以白水、茶水为首选饮料	76.9	91.4**	73.4	82.7**△△
不吃或基本不吃零食	28.8	78.7**	21.7△	31.9**△△
油炸食品每周2次以下	78.9	91.2**	64.9△	81.7**△△
不吃或基本不吃肥肉	81.5	91.7**	66.0△△	82.8**△△
少吃或基本不吃洋快餐	52.8	79.0**	45.3△	57.6**△△
每天吃较多的蔬菜	59.0	70.9**	47.1△	51.5*△△
玩游戏看电视不超过1 h	35.0	67.5**	27.2△	27.7△△
每天运动1 h以上	29.3	69.5**	23.5△	22.1△△
运动到合适的程度	27.3	67.4**	33.3△	30.3△△

注：干预前后比较，* $P<0.05$，** $P<0.01$；干预校与对照校比较，△ $P<0.05$，△△ $P<0.01$。

2.4 肥胖和超重率的变化情况 干预学校肥胖率从15.6%下降到10.4%，超重率从14.1%改变为14.2%；对照学校肥胖率从13.3%下降到11.1%，超重率从15.4%改变为15.6%。其中以干预学校三、四年级男生肥胖率下降最为显著，从21.5%下降到13.6%。

3 讨论

儿童肥胖正成为一个日趋严重的、全球关注的公共卫生问

?1994-2015 China Academic Journal Electronic Publishing House. All rights reserved. http://www.cnki.net

题。9～11 岁儿童肥胖率增高明显[3]，我国儿童青少年中男女性肥胖检出率最高年龄组分别在 10～12 岁和 11～13 岁[1]。学校是儿童青少年健康知识获得和正确行为养成的重要场所，学龄期，尤其在小学阶段是预防和控制学生肥胖的关键阶段，因此有专家认为应将目前的高危人群——城市小学和初中生作为重点防治对象[4]。

该项干预研究把学生肥胖控制与创建"健康促进学校"相结合，采取以小学三～四年级的全体学生作为重点干预对象，与学校创建"健康促进学校"的整体策略相结合，并将社区和家庭参与的策略也纳入整体策略之中，是该研究的重要特点。第 2 个特点是将知识传授与行为干预相结合，以行为干预为主要方法。如通过学生自己填写《个人管理手册》增加学生自我控制摄食行为和运动行为的意识，并由指导教师熟悉和掌握超重及肥胖学生的膳食和运动方面的动态信息，以便对肥胖和超重学生的不利于控制体重的行为进行有针对性的干预。

静坐式的生活方式，如长时间看电视、玩电脑，课外活动时间过短是肥胖的重要危险因素[5]，减少久坐少动行为（看电视、音像、计算机游戏等）和增加体力活动可以达到减少肥胖超重百分比的效果。干预学校学生玩计算机游戏和看电视不超过 1 h 的比例由干预前的 35.0%提高到 67.5%，这一比例远高于对照学校的 27.7%，也高于我国城市儿童平均每天看电视少于 1 h 的比例（35.2%）[6]；而每天运动 1 h 以上、运动量和强度适中的比例明显提高，与对照学校的差异有统计学意义。

该研究有 2 点不足之处，一是干预时间短，对在学校建立良好的健康机制后学生的肥胖控制情况缺少观察；二是 4 所学校在干预方法上没有各自的特色，统一的干预模式虽然有利于干预效果的统一评价，但是不利于对干预方法的研究。因此下一步应该对该组干预学生体重和肥胖率情况进一步进行观察，以评价健康促进学校在肥胖控制方面的持续效果。今后在类似课题中可以采用不同的干预模式，以探索更加有效的方法。

4　参考文献

[1] 徐勇，谭琪. 我国儿童青少年肥胖的现状及发展趋势研究. 中国卫生事业管理，2003，17(3)：166－167.

[2] 中国肥胖问题工作组. 中国学龄儿童青少年超重、肥胖筛查体重指数值分类标准. 中华流行病学杂志，2004，25(2)：97－102.

[3] MATSUSHITA Y, YOSHIIKE N, KANEDA F, et al. Trend in childhood obesity in Japan over the last 25 years from the national nutrition survey. Obes Rea, 2004, 12(2): 205－214.

[4] 季成叶，孙军玲. 中国学生超重、肥胖流行现状与 15 年流行趋势. 北京大学学报：医学版，2004，36(2)：194－197.

[5] 吕书红. 儿童肥胖流行趋势及干预对策探讨. 中国健康教育，2002，18(8)：526－528.

[6] 马冠生，李艳平，胡小琪. 我国城市儿童少年看电视时间的研究. 中国健康教育，2002，18(7)：411－413.

（收稿日期：2005-11-14）

【校医园地】

少腹逐瘀汤加味治疗原发性痛经 62 例效果观察

仇锦珠

【文献标识码】 B
【中图分类号】 G 478.2　R 271.11⁺3
【文章编号】 1000-9817(2006)10-0871-01
【关键词】 痛经；药物疗法；治疗结果

痛经指妇女在行经前后或经期，出现下腹及腰骶部疼痛，严重者腹痛剧烈，面色苍白，手足冰冷，甚至昏厥。痛经常持续 1～2 h 或数小时，一般经血畅流后，腹痛缓解。该病在高中女生中较为常见。痛经的治疗方法很多，但效果均不太满意或者有些治疗方法副作用较大。近 2 a 来，笔者应用少腹逐瘀汤加味治疗原发性痛经 62 例，疗效满意。现报道如下。

1　对象与方法

1.1　对象　观察组 62 例，年龄为 16～18 岁，平均 17.3 岁，病程 6 个月～3 a，既往经过其他治疗，均不满意；对照组 31 例，年龄及病程与观察组差异无统计学意义。

1.2　方法　观察组采用少腹逐瘀汤加味治疗，处方为小茴香 6 g，白芍 12 g，元胡 12 g，干姜 10 g，官桂 6 g，苍术 12 g，白术 12 g，茯苓 12 g，当归 12 g，川芎 6 g，生蒲黄 9 g，川椒 1.5 g。于经前 3～5 d，口服此方 3～5 剂，连续 3 个周期。对照组：口服芬必得，经前 1 d，每次 1 粒，每日 2 次，服 2 d，连续 3 个周期。

2　结果

观察组与对照组比较，痊愈率高，总有效率高，无效率低，其差异均有统计学意义。见表 1。

表 1　观察组与对照组疗效比较

组别	人数	痊愈	显效	好转	无效	总有效
观察组	62	30(48.39)	20(32.26)	9(14.52)	3(4.84)	59(95.16)
对照组	31	8(25.81)	11(35.48)	5(16.13)	7(22.58)	24(77.42)
χ^2 值		4.36	0.10	0.04	5.06	5.06
P 值		<0.05	>0.05	>0.05	<0.05	<0.05

注：( )内数字为百分率/%。

3　讨论

痛经多因寒凝胞宫、气血运行不畅所致。正如《妇人大全良方》所说："妇人经来腹痛，由风冷客于胞络冲任。"寒湿之邪，重浊凝滞，客于冲任，胞中与经血搏结，使经血运行不畅，故于经前一二日或经期小腹冷痛。治宜温经散寒，化瘀止痛。少腹逐瘀汤加味方中川椒、官桂、小茴香、干姜温经散寒；苍术、白术、茯苓健脾除湿；当归、川芎、元胡、蒲黄养血活血、化瘀止痛。诸药合用共奏温经散寒，养血活血，化瘀止痛之功效。在临床上应用取得了较为满意的疗效。而对照组口服芬必得，除痊愈率和总有效率均较低外，尚有 6 例出现轻度胃部不适及胃痛（停药后缓解）。因此，用少腹逐瘀汤加味治疗原发性痛经值得在临床推广应用。

（收稿日期：2006-01-10）

【作者简介】 仇锦珠（1971－　），女，江苏姜堰人，大学本科，主治医师，主要从事学校卫生工作。
【作者单位】 江苏省姜堰中学，225500.

?1994-2015 China Academic Journal Electronic Publishing House. All rights reserved.　http://www.cnki.net
