# Supplementary material for: Systematic review and meta-analysis of school-based obesity interventions in mainland China
Source: PLoS One. 2017 Sep 14;12(9):e0184704. doi: 10.1371/journal.pone.0184704 (PMC5598996; doi:10.1371/journal.pone.0184704)
Supplement: S1 Dataset — (ZIP) [file pone.0184704.s007.zip › S1_dataset/76库/40.pdf]

文章编号:1004-3624(2014)01-0101-05

16 周处方式体育教学对 9—10 岁肥胖儿童体质的影响

杨春华
(陇东学院 体育学院,甘肃 庆阳 745000)

摘要:目的:通过 16 周处方式体育教学对 9—10 岁肥胖儿童体质的影响,探究在阳光体育政策导向下能有效预防和控制儿童肥胖的现代体育教学手段。方法:按身高大于标准体重的 20%的标准,在两所小学四年级同学中各自抽出 40 名肥胖儿童作为实验组和对照组,查阅已有的文献资料,把一学期的体育课教学任务与运动处方相结合,从运动强度、运动内容、运动频度和运动时间上进行了科学的选定,委任通过培训的体育教师对实验组同学进行处方式教学,对控制组同学进行常规教学,采用常规的体质测试方法、SCL—90 量表和 t 检验对两组受试者的测试指标进行比较分析。结果:实验组和对照组教学前各项指标无差异($p>0.05$),实验组教学前后指标变化明显,其中 BMI 指数、1min 爬行次数和 1min 跳绳次数有显著变化($p<0.05$),SCL—90 所测定的各项因子中,在人际关系、抑郁、偏执因子上实验组处方前后有显著变化($p<0.05$)。对照组在教学前后指标有变化,但无显著性差异,实验组和对照组在处方后各项指标都有显著变化,其中 BMI 指数、皮褶厚度、围度、1min 爬行、1min 跳绳有显著变化($p<0.05$),在人际关系、抑郁、偏执因子上实验组处方前后有显著变化($p<0.05$)。结论:该处方式教学适宜在同年龄段肥胖儿童体育教学中推广应用,能有效的预防和治疗肥胖,改进儿童的心理健康状态。

关键词:处方式教学;肥胖儿童;体质;心理健康
中图分类号:G804.49　　**文献标识码:**A

Effect on Sports Prescription Education of 16 Weeks for Children Obesity with 9—10 Years Old

YANG Chun-hua
(Longdong University of Physical Education, Qingyang 745000, China)

Abstract: Objective: Through 16 weeks' physical teaching predisposition for obesity in children with 9—10 years old, this paper explored the modern sports teaching methods for effective prevention and control of childhood obesity in the sunshine sports policy orientation. Method: Standard height was greater than 20% of the standard weight, each of the two fourth—grade students out of 40 obese children as the experimental group and the control group, referring to the existing literature. One semester of physical education teaching tasks and exercise prescription was a combination of science selected frequency from the exercise intensity sports content, sports and exercise time, appointed by the training of sports teachers in the experimental group of students at the way teaching and conventional control group of students teaching, using conventional physical test methods, SCL—90 scale, and t—test to compare the two groups of test indicators. Results: All indexes of experimental and control groups before teaching were no difference ($p>0.05$). Before and after the teaching, experimental group indicated significantly changed ($p<0.05$), including BMI, one minute crawl, and one minute of jump rope. Views SCL—90 factor measured before and after the experimental group prescription interpersonal sensitivity, depression, paranoid ideation significantly changed ($p<0.05$). Control group, there was a change in teaching before and after the index, but no significant difference between the experimental group and the control group in the prescription after the indicators have significant minute rope skipping significant change ($p<0.05$), interpersonal sensitivity, depression, paranoid ideation significant change ($p<0.05$) before and after the experimental group prescription. Changes in body mass index (BMI), skinfold thickness, circumference, one minute crawl. Conclusion: The way of teaching was suitable for popularization and application in the same age

收稿日期:2013-07-26
作者简介:杨春华(1979—),男,甘肃庆阳人,硕士,研究方向:学校体育与运动康复项目.

?1994-2014 China Academic Journal Electronic Publishing House. All rights reserved.　http://www.cnki.net

group of obese children in sports teaching. It could affect the prevention and treatment of obesity and improve children's mental health status.

Key words: sports prescription; obese children; physique; mental health

儿童时期是人体生长发育的关键阶段,各种身体素质的敏感期都发生在这个阶段。但近十年的统计资料表明,目前肥胖已成为威胁我国儿童少年健康的严重问题。在生活水平日益提高的今天,中国儿童肥胖检出率日趋提高,儿童的身体素质在急剧下降,众多的专家认为导致这种现象出现的主要原因是儿童运动的时间越来越少了,家长望子成龙心切,给孩子报各种各样的学习班多了,学校迫于升学率的压力轻视了体育课的质量,现代高科技使得儿童的户外活动时间减少,饮食的不节制,营养不均衡,促使了小胖墩日益增多。伴随着世界儿童肥胖的日趋严重,众多的专家学者对肥胖儿童的预防和治疗做出了大量的工作,有行为、饮食、情感、心理、药物、电刺激等各种减肥手段的研究,综观前人的研究,儿童处于生长发育期,运动治疗是儿童减肥的最佳治疗手段[1]。现代教育体制使儿童 50%的活动时间都在学校度过,而阳光体育的教学,保证了儿童每天都有 1h 的运动时间,每周四节体育课,保证了孩子能有组织、循序渐进的学习。但是小胖墩为何还是有增无减呢?本研究采用了有针对性的处方式教学,以期通过体育教学方式的革新,能有效改善儿童肥胖日益增多这一现状。

1　研究对象及方法

1.1　研究对象

按身高大于标准体重的 20%定位肥胖标准[2],从庆阳市西峰区南街小学四年级学生中抽取肥胖学生 40 名作为实验组,进行 16 周处方式体育课程教学;从北街小学同年龄级别组中抽取小学生 40 名作为控制组,进行 16 周常规体育课程教学(他们体质在教学前无差异),对教学前后选取的各项指标进行测试。

1.2　研究方法

1.2.1　文献资料法。通过中文期刊全文数据库、中国期刊网优秀博硕士论文数据库、万方数据库等进行大量检索,查阅儿童运动干预和处方式教学对儿童体质影响的相关文献,并对其进行分类整理,为本研究提供理论支持。

1.2.2　测量法。参考《国民体质测定标准》、教育部下发的《2010 年全国学生体质健康调研实施方案》[3,4]和有关专家在这方面的研究[5,6],采用常规体质测量方法测试形态、机能和素质三类指标,具体内容如下:身高,体重,皮褶厚度(肱三头肌部、肩胛下部、腹部),围度(胸围、腰围、大腿围),握力,1min 斜卧撑,1min 爬行,立定跳远,坐位体前屈,5min 跑。

1.2.3　问卷调查法。本研究采用 SCL—90 心理健康症状自评量表,对实施处方前后一周实验组和对照组 80 名同学分别进行心理健康问卷测试。此表包括 9 个因子,每一个因子反映出测试者的某方面症状情况[7],通过因子分可了解症状分布特点。

1.2.4　实验法。根据现有的研究成果、本年龄段学生体质的基本特征、学生体育课的基本任务和认知教育理论,制定了如下的处方式教学内容:a 快走和慢跑 5min 热身,放松 1min;B 高抬腿跑、侧身跑、后踢腿跑(每组 3min,两组,组间歇 1min)8min;C 四肢匀速爬行(1min+30s 间歇共四组)6min;D 交叉跳+兔跳(30 次/min,两组,组间歇 30s)5min;E 跪撑、斜卧撑(各两组、每组 1min,组间休息 20s)5min;F 双人摇船(腹肌锻炼,30 次/min,3 组,组间歇 30s)5min;G、韵律操 5min,休息 1min;运动总时间为 32min,利用间歇期 8min 进行运动认知教育(类容包括对肥胖儿童参与运动的充分的鼓励和表扬;肥胖对儿童目前和今后在心理、生理、行为和其在事业家庭等各方面造成的危害;科学合理运动给儿童带来的各种益处;正确的饮食、行为习惯对儿童成长与成才的帮助),每周一至周四下午 3∶45~4∶25,委任经过培训的两名专职教师,在本校的室内活动中心进行,前两周组织学生学习各种动作和流程,3~18 周处方式体育课教学,教学采用遥测心率表和颈动脉法,调整每组运动的频度,使心率保持在 135~165 次/min 这一区间。

1.2.5　比较法。通过对比研究方法对实验组与对照组、实验组内、对照组内处方式教学前后测试数据进比较分析。

1.2.6　数理统计法。研究中采用 Excel 对数据进行整理,采用 T 检验进行分析,两组之间采用组间 T 检验,组内采用独立样本 T 检验。数据用(t±s)表示,显著性水平用*表示(*为 $P<0.05$,**为 $P<0.01$)。

?1994-2014 China Academic Journal Electronic Publishing House. All rights reserved.　http://www.cnki.net

2 测试结果与分析

2.1 两组儿童处方式教学前后体质指标的组内比较

两组儿童在 16 周处方式教学前后各项测试指标如表 1 所示:对照组的各项指标在教学前后无明显差异(P>0.05),但在教学前后 BMI 指数、皮褶厚度和围度有增加的趋势,说明随着时间的推移对照组肥胖情况日趋严重,立定跳远、斜撑和 5min 跑也有提高,但变化不明显,表明肥胖儿童随着时间的推移,他们的身体素质有自然增长的趋势,但增长幅度比较小。实验组各项测试指标在处方式教学前后存在明显差异,其中 BMI、1min 爬行和 5min 跑在处方前后变化最明显(P<0.01),立定跳远、斜撑、皮褶厚度、围度指标也有变化(P<0.05),表明处方式教学一方面控制了儿童的肥胖,另一方面也提高了儿童的体质。

表 1 两组儿童在处方式教学前后组内体质指标比较(t±s)

指标	实验组		对照组	
	教学前	教学后**	教学前	教学后
BMI	24.47±2.2	21.58±1.7**	23.98±1.8	24.78±1.8
皮褶厚度(mm)	62.56±7.8	58.42±5.2*	63.48±8.2	66.56±8.2
围度(cm)	162.56±14.8	150.56±11.2*	164.8±13.6	166.8±14.6
1min 斜撑(次)	30±3	35±3*	30±3	32±3
立定跳远(m)	1.2±0.2	1.35±0.3*	1.2±0.3	1.29±0.3
1min 爬行(次)	55±6	72±6**	57±5	60±5
1min 跳绳(次)	58±6	75±8	57±5	61±5
5min 跑(m)	737±75	789±75**	736±66	745±66

注:* P<0.05 ,** P<0.01;下同

2.2 两组儿童处方式教学前后体质指标组间的比较

两组儿童在处方式教学前后各项测试指标组间比较如表 2 所示:在处方式教学前,两组各项体质都无差异(P>0.05);在处方式教学后,实验组和对照组各项测试指标之间有明显变化,其中 BMI、皮褶厚度和围度变化最明显(P<0.01),表明处方式教学能有效的控制儿童肥胖,1min 爬行次数、1min 跳绳次数和 5min 跑成绩明显提高(P<0.01),表明处方式教学明显的改善了学生的耐力素质和下肢力量。

表 2 两组儿童在处方式教学前后组间比较(t±s)

指标	教学前		教学后	
	实验组	对照组	实验组	对照组
BMI	24.47±2.2	23.98±1.8	21.58±1.7**	24.78±1.8
皮褶厚度(mm)	62.56±7.8	63.48±8.2	58.42±5.2**	66.56±8.2
围度(cm)	162.56±14.8	164.8±13.6	150.5±11.2**	166.8±14.6
1min 斜撑(次)	30±3	30±3	35±3	32±3
立定跳远(m)	1.2±0.2	1.2±0.3	1.35±0.3*	1.29±0.3
1min 爬行(次)	55±6	57±7	72±6**	60±6
1min 跳绳(次)	58±6	57±5	75±8**	61±5
5min 跑(m)	737±75	736±66	789±75*	745±66

2.3 处方式教学对儿童心理健康的影响

肥胖对儿童心理健康的影响已成了众多学者共同关注的问题,胖儿童的自尊心、自信心下降,社会适应能力、活动能力、社交及学习能力降低[8]。本研究采用 SCL— 90 心理健康症状自评量表,此表包括 9 个因子,每一个因子反映出测试者的某方面症状情况,通过对这些因子分析可了解其主要症状。实施处方式教学对肥胖儿童心理健康的影响如表

?1994-2014 China Academic Journal Electronic Publishing House. All rights reserved. http://www.cnki.net

3，实验组学生实验后各因子得分都有下降，在人际关系、抑郁、偏执因子上明显下降，与实验前相比呈显著性差异（P＜0.05），在焦虑因子上与实验前相比有差异。对照组实验后在人际关系、抑郁因子上与实验前相比有差异，其他各因子项目均无明显变化。说明实验组在这些因子上的进步与处方式教学对儿童心理的改善有直接关系。一方面，处方式教学组的人文处境完全相同，他们相互之间的交往就很轻松和谐，摆脱了其他同学嘲笑、讽刺给他们造成的心理压力；另一方面，运动处方教学对参与者的激励与表扬，参与者之间的默契配合与共同进步，培养了儿童的自信心、交际能力和进取精神[9,10]。

表3 实施运动处方教学对儿童心理健康的影响（t±s）

因子项目	实验组		对照组	
	实验前	试验后	实验前	试验后
躯体化	1.32±0.28	1.29±0.31	1.31±0.32	1.30± 0.36
强迫症状	1.82±0.49	1.83±0.43	1.81± 0.41	1.79±0.37
人际关系	1.88±0.41	1.65±0.37*	1.89± 0.52	1.81±0.41
抑郁	1.60±0.42	1.45± 0.41*	1.62± 0.35	1.53± 0.32
焦虑	1.48±0.33	1.38± 0.32	1.46±0.48	1.43±0.42
敌对	1.59±0.55	1.54± 0.46	1.58±0. 37	1.52±0.26
恐怖	1.40± 0.39	1.33±0.36	1.38±0.40	1.37±0.43
偏执	1.63± 0.31	1.41±0. 32*	1.61±0.34	1.55± 0.33
精神病性	1.44±0.33	1.38± 0.36	1.45±0. 38	1.43±0.31

3 讨 论

3.1 肥胖儿童的的运动疗法

儿童期单纯性肥胖症已是21世纪严重的健康社会问题。从20世纪八十年代初开始，不论是发达国家还是发展中国家，在世界范围内各国儿童的单纯性肥胖患病率不断增高。单纯性肥胖症对儿童心理、生理都有严重危害：生理上可迟滞儿童的有氧发育能力，提前动用心肺储备功能，降低体质健康水平，心理上影响其正常潜能发挥，使自尊心、自信心下降，影响其性格塑造、气质培养、健康行为习惯形成。远期内儿童期单纯肥胖症可发展为成年期肥胖，是成人期心血管疾病、糖尿病、高血压的重要危险因素，因此防治儿童单纯性肥胖，保护儿童的身体健康，提高未来人群的素质，成了儿童保健的一个重要课题。世界卫生组织和各国政府在这方面都非常关注并做出了许多实际的工作，例如阳光体育的实施，儿保专家们在这方面也取得了大量的科研成果，有临床、膳食、心理、行为治疗，综合干预和健康促进学校等手段都应运而生[11]。而在众多的研究成果中，运动干预是所有学者都认可的一种方式[12]。谭思洁等[6]对30名9～10岁肥胖儿童做过为期8周的运动处方实验，在无饮食干预下并取得了显著的效果，说明了科学合理的运动能有效地治疗肥胖，他们设计的运动处方适宜在同年龄阶段的儿童中推广。

3.2 处方式教学在体育教学中的应用

处方式教学则是体育教学不断革新的成果，不同于一般体育课教学，它是一种即可以锻炼身体、又能培养学生能力的新型教学模式，以处方式教学作为主要手段去完成体育教学中的主要任务，并以处方教学为主要方式解决众多与体质发展相关的问题，处方式教学模式可在提高学生身体素质的同时培养自觉锻炼能力[13]。由于学生身体素质、体质、技术和心理水平的差异，采用统一模式教学，无法更好地培养他们的体育兴趣、调动他们体育学习的积极性，无法更有效地提高他们的健康。因此，将运动处方引进教学，结合学生身体素质、心理水平进行教学改革，建立处方式体育教学体系，势必会拓宽体育教学视野，为体育教学注入新的生机和活力。目前在体育教学中有广泛的应用，而且在健身和减肥方面的研究比较多，但研究对象年龄都是以中学和大学为主。关于小学生进行处方式教学研究的目前比较少。一方面是儿童对运动认识不足，自我感知能力差，他们对自身的运动强度难以掌控。另一方面是肥胖儿童缺乏耐性、对运动容易失去兴趣，从而在思想上产生消极抵抗情绪。因此本处方教学在运动强度控制和运动种类选择上参考杨春华[14]等的研究成果，在每一次处方式教学的最后几分钟采取不同的方式对肥胖儿童进行运动认知教育，从心理、认知、行为、饮食和思想上引导肥胖儿童树立科学的运动观和人生观。

?1994-2014 China Academic Journal Electronic Publishing House. All rights reserved. http://www.cnki.net

3.3　肥胖儿童处方式教学的制定和实施

目前关于肥胖儿童运动减肥方面的研究已经有许多成果,谭思洁、杨春华[6]用乳酸无氧阈拐点发做过 30 名 9～10 岁儿童运动的安全心率,并用能量代谢车做过儿童运动减肥的几种主要运动方式的能量消耗,他们的研究方法科学、合理,研究结果为肥胖儿童运动处方的制定奠定了坚实的理论基础,因此本处方式教学中采用了他们的运动强度,把心率控制在 135～165 次/min,他们所测定的爬行、5min 跑、斜卧撑、跳绳、双人摇船和 高抬腿也是儿童常见的运动方式,而且能量消耗已知,本处方式教学内容选定了这几种方式,另外秦琴和李劲勇[15,16]做过小学生健美操的运动处方也取得了很大的成效,因此本处方也选定了 5min 健美操做为处方式教学的一部分。总体上说本处方式教学的各项内容和教学中强度把控,都是前人研究的结果,本研究只是把这些研究结果引入到儿童肥胖体育教学中来,并结合本次课的任务,把儿童思想、情感、行为等认知教学恰当地融入到体育课教学中,由专门培训的体育教师进行一学期的处方式教学,结果如表 2 和表 3 所示。

3.3　肥胖儿童处方式教学的效果评价

16 周处方式教学对儿童肥胖的干预效果如表 2 和表 3 所示,教学前两组儿童所测得各项指标无差异,教学后两组所测定的各项指标有很大的差异,其中实验组的 BMI 指数、总的皮褶厚度、围度都明显减少($p<0.01$),BMI 指数由 24.47±2.2 降低为 21.58±1.7,接近于正常儿童身高体重标准,说明实验组肥胖指数减小,肥胖得到了明显控制;对照组 BMI 指数由 23.98±1.8 上升为 24.78±1.8,说明对照组肥胖儿童的肥胖指数处于一种自然增长的状态,肥胖度继续增加,本处方式教学能有效改善儿童的肥胖状况。实验组 1min 跳绳次数、1min 爬行次数、立定跳远和 5min 跑与对照组在处方后差异明显($p<0.01$),说明实验组下肢力量的到了改善,有氧运动能力增强,间接地也反映了他们的呼吸循环系统得到了改善,说明了实验组体质状况的到了改善,实验组与对照组斜撑起变化不是非常明显,这可能与处方式教学内容偏重下肢和腰腹练习,而上至练习安排比较少有关。另一方面处方式教学对儿童心理的干预如表 3,实验组学生实验后各因子得分布都有下降,在人际关系、抑郁、偏执因子上明显下降,与实验前相比呈显著性差异($p<0.05$),这同张伟[17]等对大学生进行处方教学取得效果一致,张伟研究认为处方式教学对改善大学生的人际关系、抗焦虑及偏执等方面有明显效果,有助于学生心理健康的发展和良好个性的培养。本处方式教学也取得了同样的收获,通过处方式教学不仅有效地的防止了儿童肥胖,改善儿童体质健康状况,而且明显的改善了肥胖儿童心理健康。

3.4　肥胖儿童处方式教学的应用前景

在以人为本、健康第一的现代学校体育教学思想引领下和每天锻炼一小时,幸福生活一辈子的阳光体育指导下。儿童肥胖已引起众多家长、教育家和的社会人士的广泛关注,从认知、行为和运动方面进行治愈是众多的儿保专家的共识。处方式教学可以充分考虑这些因素,借助学校教学环境、体育课这一平台,把肥胖儿聚集在一起从认知、行为和运动上进行有针对性地教学,一方面可以有效控制儿童肥胖,保证儿童的健康成长,减轻家长的忧虑;另一方面能有效地完成学校体育教学的任务,解决摆在教育者面前的儿童肥胖日增这一社会难题。本处方式教学在是在校方协商后进行的一次体育课程教学改革,一学期的处方式教学后得到了校方的充分肯定,同时也赢得了家长的极力的支持,最主要的是肥胖儿童由对体育课发愁,到对体育课产生了浓厚的兴趣,他们对运动认知观念的改变是本次处方式教学最大的收获。

4　小　　结

4.1　经过了一学期的处方式教学,实验组 BMI 指数、总的皮褶厚度、围度都明显减少($P<0.01$),而对照组这些指数有继续增加的趋势,说明实验组的肥胖状况的到了控制,对照组肥胖有自然增长的趋势,另一方面实验组 5min 跑的距离、1min 跳绳次数、1min 爬行次数和立定跳远成绩明显提高($P<0.01$),说明本处方式教学能有效的提高儿童体质,在人际关系、抑郁、偏执因子上显著变化($P<0.05$),说明本处方式教学能有效的改善儿童的心理健康。

4.2　本处方式教学有效地防止了儿童肥胖,提高了儿童体质,改善了肥胖儿童的心理状况,实现了预期的教学目标,说明了本处方式教学合理、科学,肥胖儿童对运动的认知、行为和心理方面的改善,表明本处方式教学是现代体育教学理念上的一次突破,适宜在同年龄段肥胖儿童教学中推广。

参考文献

[1]　闫淑娟.我国儿童肥胖干预方法研究现状与分析[J].中国妇幼保健,2006,21(17):2463—6.

[2]　谭思洁,刘洵.健康评价与运动处方[M].天津:天津科学技术出版社,2005.

(下转第 128 页)

?1994-2014 China Academic Journal Electronic Publishing House. All rights reserved.　http://www.cnki.net

太极文化的道统渊源。因此,学习、传承和体悟中华传统的文化精髓,使习练者得到“苟日新,日日新”的惊喜,体悟“生生不已”的人生和天地之道。

4.4.2 开启美的智慧。太极拳具备通往生命心灵自由的内在精神美,使得太极拳的内容、气韵表现和习练者的修养内涵都带给人们悦目、悦志、悦心的快乐享受,激励人们不懈追求生命质量,提高生活境界和意趣,不断体验心灵神往自然的历程,直至实现人的和谐与自由之美。与此同时,太极拳自身所携带的和谐、平衡、空灵等美学思想也让习练者得到最大的实惠和受益。

4.4.3 开启思维的智慧。学习中华传统文化、学习太极拳理,感知身体筋骨皮的放松和意气的流动,愈练愈精后便能融会贯通文化精髓,特别是触类旁通的智慧就容易被开启。借助太极拳“中正”、“支撑八面”与“舍己从人”等思想,解答现实生活中遇到的问题时就能作更恰当的选择。经过对太极拳的持续揣摩,容易让人形成太极拳特有的逆向思维和发散想象思维,从而有助于思维的开拓、智慧的增益。

5 结束语

在生命生老病死的过程中,生命的精气学说、阴阳学说、心物一元学说、五行学说都从不同的角度对生命的变化做出不同的阐释和解析。不管生命的解析如何,变化如何,太极拳自创始以来始终像个益友伴随在生命的身旁,给予生命最大的支持和帮助。太极拳独有的运动形式和运动文化铸就了生命益寿延年的养生功效、防护生命的安全功能、提高生命个体的人格修养作用,并开启了人思维的智慧,所有这些生命智慧都将给生命带来丰富而有意义的收获。

因此,用太极拳文化所特有的智慧来优化人生,应当成为当代人的一个重要选择。通过太极拳之理的体悟,太极拳之相的锤炼,太极拳之用的实践,由内达外,从而开启真正的大悟性、大智能、大光明,并在潜移默化中,让身心性命得以获得真正自我感知、超越和优化。

参考文献

[1] 余功保.中国太极拳名家对话录 上善若水[M].北京:人民体育出版社,2008.

[2] 余功保.中国太极拳名家对话录 盈虚有象[M].北京:人民体育出版社,2006.

[3] 余功保.中国太极拳名家对话录 随曲就伸[M].北京:人民体育出版社,2005.

[4] 祝大彤,薛秀英.自然太极拳[M].北京:人民体育出版社,2006.

[5] 王壮弘口述,杨云天等整理. 上善若水[M].海口:海南出版社,2009.

[6] 《中国武术百科全书》编撰委员会.中国武术百科全书[M].北京:中国大百科全书出版社,1998.

[7] 谢华.黄帝内经[M].北京:中医古籍出版社,2000.

[8] 解守德.太极内功心法[M].北京:人民体育出版社,2006.

[9] 林正范.大学心理学[M].杭州:浙江大学出版社,2004:4.

[10] 刘力红.思考中医[M].桂林:广西师范大学出版社,2006.

[11] 蓝晟.国学与太极拳[M].北京:当代中国出版社,2011.

[12] 宗白华.艺境[M].北京:北京大学出版社,1999.

(上接第105)

[3] 中国学生体质与健康研究组.2000年中国学生体质与健康调研报告[M].北京:高等教育出版社,2002.

[4] 中共中央国务院.关于加强青少年体育增强青少年体质的意见.2007.

[5] 李石庄.体育锻炼对青少年体质健康影响的研究[D].西南大学,2010.

[6] 谭思洁,杨春华.9—10岁肥胖儿童减肥运动处方的研制及效果观察[J].中国运动医学杂志,2011,30(1):11—17.

[7] 沃建中.走向心理健康发展篇[M].北京:华文出版社,2006.

[8] 刘军祥,黄宗能,苏红卫.儿童肥胖现状及影响因素调查[J].中国公共卫生,2006,22(9).

[9] 孙乡,戴剑松.大学生体质与心理健康的关系研究[J].中国体育科技,2007(9).

[10] 斯图尔特·比尔德.心理学在锻炼及健康相关的身体活动中的应用[J].体育科学,2000,20(4):71—74

[11] 肖夕君,刘玉江.肥胖研究进展及减肥运动处方的实践应用[J].湖南人文科技学院学报,2006(3):112—114.

[12] 薛长勇.肥胖和膳食的关系及其膳食治疗[J],现代康复,2001,9(5):10—11.

[13] 彭军,吕平.普通高校体育课健身运动处方的实效性研究[J].成都体育学院学报,2002,28(3):41—44.

[14] 杨春华,谭思洁.10—11岁肥胖儿童主要减肥手段能量代谢率的研究[J].辽宁体育科技,2012(7):61—63.

[15] 秦琴.昆明市城区小学健美操课对学生身心发展影响的实验研究一以新迎第二小学为例[D].云南师范大学,2008.

[16] 李劲勇.小学生有氧健身操运动处方的研制与效果观察[J].阴山学刊,2009,23(3):104—106.

[17] 张伟,吕玉军.运动处方教学与大学生身心健康的研究[J].体育科技,2011,32(1):63—65.

?1994-2014 China Academic Journal Electronic Publishing House. All rights reserved. http://www.cnki.net
