# Supplementary material for: Systematic review and meta-analysis of school-based obesity interventions in mainland China
Source: PLoS One. 2017 Sep 14;12(9):e0184704. doi: 10.1371/journal.pone.0184704 (PMC5598996; doi:10.1371/journal.pone.0184704)
Supplement: S1 Dataset — (ZIP) [file pone.0184704.s007.zip › S1_dataset/76库/41.pdf]

【健康促进】

肥胖儿童学校运动俱乐部干预效果评价

陆大江[1],张永强[2],平杰[1],朱志高[3]

1.上海体育学院运动人体科学学院,上海 200438;2.郑州71中学;3.上海市二联小学

【摘要】 目的 探索学校运动俱乐部对超重、肥胖儿童的干预效应,为建立有效可行的肥胖健康教育及干预模式提供依据。方法 抽取上海二联小学7~9岁儿童,采用中国儿童青少年超重肥胖体质指数标准为评价标准,共筛出超重和肥胖儿童85名。根据自愿原则分为干预组(66名)和对照组(19名)。对干预组儿童进行学校运动俱乐部群体干预,对照组不给予任何干预,1.5 a后对2组儿童躯体形态指标进行测量并比较。结果 干预后肥胖和超重儿童所占比例分别下降25.8和1.5个百分点;干预组男童身高平均增长值明显高于对照组,干预组女童体重平均增长值明显低于对照组(P值<0.05);肥胖超重儿童在运动俱乐部活动中运动步数基本稳定在每天4 000步,达到运动干预规定的运动量,运动强度稳定在140次/min的最佳运动心率水平上。结论 采用计步器和心率表对运动干预过程实施监控,建立肥胖儿童个人运动管理档案,合理调节运动处方,是科学管理干预运动的有效手段。

【关键词】 肥胖症;运动活动;健身中心;干预性研究;儿童

【中图分类号】 R 725.892.5 R 455 G 479 【文献标识码】 A 【文章编号】 1000-9817(2011)11-1338-03

Personalized exercises intervention for obese children in a school fitness center/*LU Da-jiang**, *ZHANG Yong-qiang*, *PING Jie*, *et al.** *Shanghai Physical Education Institute*, *Shanghai*(200438), *China*

【**Abstract**】 **Objective** To research an effective and practical model for establishing a personalized exercises education and intervention methods among obese and overweight children in a school fitness club setting. **Methods** A total of 85 obese or overweight pupils(7-9 years old), from obesity screening program of Second Allies Primary School of Shanghai, participated the study, and were divided into two groups: Intervention group (66 children) and the control group(19 children). The personalized obesity education and exercises intervention was 18 months. **Results** 25.8% Obesity rate reduction and 1.5% overweight rate reduction were observed in intervention group. The average height increase among the boys in the intervention group was higher than the boys in the control group. The average weight increase among girls in intervention group was lower than the girls in the control group(P<0.05). The pedometer reading at intervention group stabilized around 4 000 steps per day with exercises intensity at the heart rate around 140/min. **Conclusion** Using pedometer and heart rate for monitoring exercises intervention is a simple, practical and effective way to conduct a personalized exercises intervention among school children.

【**Key words**】 Obesity; Motor activity; Fitness centers, Intervention studies; Child

儿童肥胖是多因素综合作用的结果,其中饮食过量和运动缺乏是导致肥胖的主要因素[1]。儿童单纯性肥胖严重影响儿童青少年的生长发育,不仅导致其体态臃肿、活动能力差,很多还伴有平足、膝内翻、下肢弯曲、脊柱损害和缺钙等。更严重者会损害心肺功能,导致有氧运动能力和心脏储备降低。肥胖还会造成儿童心理问题和社会交往能力的障碍,如自卑感强、孤僻和消沉等,缺乏参与竞争所必需的自信心[2]。随着我国经济的发展,人民生活水平不断提高,肥胖发生率也呈逐年上升的趋势。针对目前儿童肥胖的状况,如何采取有效的方法对其进行干预,已成为社会广泛关注的课题。

1 对象与方法

1.1 对象 抽取上海市杨浦区二联小学二~三年级年龄7~9岁的儿童350名,根据体检资料进行筛选,排除服用激素、内分泌异常等导致的病理性肥胖。共筛选出超重儿童36名,肥胖儿童49名,其中男生59名,女生26名。根据自愿原则,将超重和肥胖儿童分为干预组和对照组。干预组为66名,其中超重儿童23名,肥胖儿童43名;男生49名,女生17名。对照组为19名,其中超重13名,肥胖6名;男生10名,女生9名。

1.2 方法

1.2.1 身体形态指标测试 测试指标:身高、体重、BMI、体脂率、上臂放松围、腰围、臀围、上臂部皮褶厚度、肩胛部皮褶厚度、腹部皮褶厚度。体格测量使用标准量具,由专人进行测量。测定方法按1993年出版《实用体质学》[3]中体格测量方法与评价标准进行。

1.2.2 工具 体质测试全套仪器(北京东腾红体育有限公司),OMRON HBF-301体脂率仪,Polar S810i心率表(芬兰博能公司),OMRON HJ-005计步器(欧姆龙公司)。

1.2.3 肥胖、超重判断方法 以体质指数(BMI)=体重(kg)/身高(m^2)为公式计算BMI值。以2004年发表的中国儿童青少年超重肥胖体质指数标准为评价标准[4],进行超重和肥胖儿童的筛选和判断。

1.2.4 肥胖、超重儿童学校运动俱乐部活动 利用放学后1 h的时间,建立肥胖、超重儿童运动俱乐部,保证每周运动2次。运动的强度控制在儿童最大心率的60%~80%。以有氧运动为主,包括有氧操、慢跑、跑楼梯、跳绳等,并结合小负荷阻力运动与垫上运动。考虑到儿童的心理特点和兴趣,增加体育游戏和其他运动项目如足球、篮球等的比重,同时每周从肥胖、超重儿童中征集运动内容以做参考。每周的活动内容由专家指导、审核,最终以电子书的形式提前发放到体育教师和儿童手中。《肥胖儿童干预手册》包括活动名称、图示、组织、活动方法、运动时间、运动组数等。俱乐部活动由经验丰富的体育教师组织,便于肥胖、超重儿童的管理和活动内容的实施,保证运动的有效性。每次活动由专人进行运动监控,运动效果由专家进行评价。

【基金项目】 2009年国家科技部支撑课题(2009BAK62B02);2010年上海市高校重点学科资助(S30802)。

【作者简介】 陆大江(1960-),男,广西省人,硕士,副教授,主要从事体质与健康、运动处方及健康促进工作。

DOI:34-1092/R.20111116.1552.024

?1994-2015 China Academic Journal Electronic Publishing House. All rights reserved. http://www.cnki.net

2 结果

2.1 干预组干预后肥胖度的变化 通过运动综合干预肥胖儿童的比例由干预前的65.2%下降为39.4%,比干预前降低了25.8个百分点;超重儿童的比例由干预前的34.8%下降为33.3%,比干预前降低了1.5个百分点。27.3%的超重、肥胖儿童恢复到正常体重,差异有统计学意义($\chi^2=23.22,P<0.01$)。根据肥胖、超重儿童肥胖等级变化情况将其分为改善组和保持组,肥胖等级有所下降的为改善组,肥胖等级保持不变的为保持组。对2组干预对象的体脂率、腰围、3个皮褶厚度(上臂、肩胛、腹部)、臀围和上臂围7个形态指标进行比较,结果显示,改善组在体脂率、腰围、臀围和上臂围4个围度指标上明显低于保持组(P值均<0.01)。改善组的上臂、肩胛和腹部皮褶厚度3个指标虽低于保持组,但差异无统计学意义(P值均>0.05)。

2.2 对照组和干预组男生干预前后形态指标增长值比较 经过为期1.5 a的干预,干预组身高平均增长值高于对照组,但差异无统计学意义($P>0.05$);干预组体重平均增长值明显低于对照组($P<0.05$)。

表1 对照组和干预组干预后男生形态发育指标增长值比较($\bar{x}\pm s$)

组别	人数	身高/cm	体重/kg	腰围/cm	体脂率/%
对照组	10	7.49±2.38	6.89±1.21	3.78±3.73	5.18±2.86
干预组	49	8.35±1.18	5.80±2.56	3.40±4.35	3.72±5.87
t值		1.112	-2.226	-0.313	-0.763
P值		>0.05	<0.05	>0.05	>0.05

2.3 对照组和干预组女生干预前后形态指标增长值比较 经过为期1.5 a的干预,干预组身高平均增长值明显高于对照组($P<0.05$)。干预组体重、腰围及体脂率增长值均低于对照组,但差异无统计学意义(P值均>0.05)。见表2。

表2 对照组和干预组干预后女生形态发育指标增长值比较($\bar{x}\pm s$)

组别	人数	身高/cm	体重/kg	腰围/cm	体脂率/%
对照组	9	8.04±1.49	7.49±3.79	5.21±4.82	4.01±3.66
干预组	17	9.35±1.46	6.45±2.32	4.78±3.72	2.66±2.86
t值		2.150	-0.753	-0.252	-1.039
P值		<0.05	>0.05	>0.05	>0.05

2.4 超重、肥胖儿童运动俱乐部干预监控

2.4.1 俱乐部活动的运动量 从图1可见,前7周干预对象的每周的日平均运动步数总体呈上升趋势,运动量增大,8~13周由于学校的其他活动比较集中,影响到俱乐部活动的运动量,运动步数有所下降。但此后运动量逐渐恢复到4 000步左右。19周后俱乐部活动的日平均运动步数基本稳定在4 000步,达到运动干预规定的运动量,并且有逐渐增长的趋势。在整个干预过程中,干预对象的运动步数与活动内容有很大关系,受活动内容的趣味性和活动组织情况影响较大,表现出一定的波动。

2.4.2 俱乐部活动的运动强度 从图2可见,干预对象的平均运动心率在前13周,波动较大,1~6周呈下降趋势,此后的平均运动心率呈逐步上升趋势,并基本保持140次/min的水平。

对肥胖、超重儿童在俱乐部活动中的运动心率进行抽样调查发现,运动心率在160次/min区间内时间,占全部活动时间的59.2%,运动心率超过120次/min区间的时间占全部活动时间的23.8%,运动心率低于120~160次/min区间的时间占全部活动时间的16.9%。见图3。

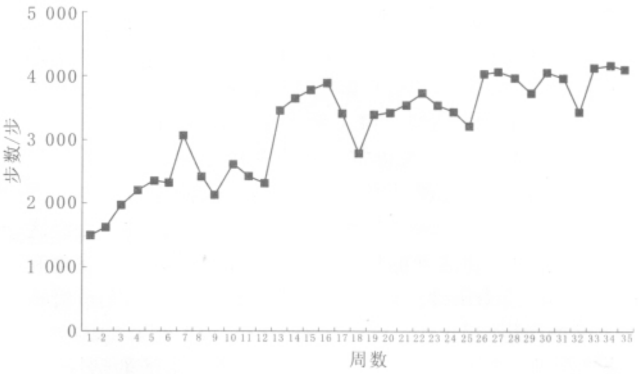

图1 儿童第1~35周每周日平均运动步数

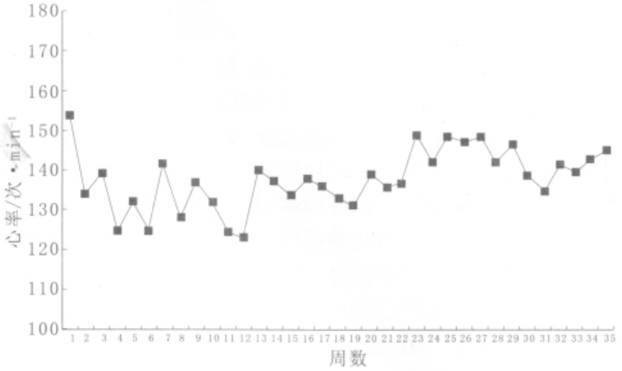

图2 儿童第1~35周平均运动心率

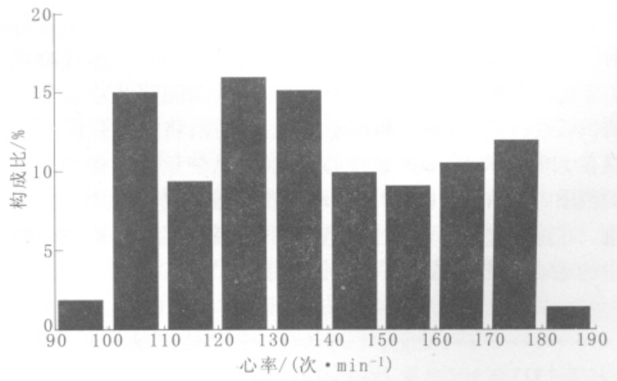

图3 儿童每次活动课的不同运动心率构成

3 讨论

3.1 运动对控制儿童单纯性肥胖的影响 儿童正处在生长发育的高峰期,需要丰富的营养以保证正常的生长,而节食减肥和药物减肥对儿童的生长发育会产生不良的影响。采用运动干预对肥胖、超重儿童进行治疗可以避免节食和药物对儿童的伤害。运动消耗能量,可减轻低脂到高脂饮食造成的正脂肪平衡,抑制过度进食所引起的脂肪组织的增加。运动不但使机体消耗能量,而且影响安静代谢率及食物的特殊动力作用。虽然单一运动的减肥效果仍存在争议,但运动消耗内脏脂肪,改善脂肪在体内的分布,在控制肥胖中有着不可代替的作用[5]。运动干预结合饮食调整、行为调整和健康教育对肥胖、超重儿童进行综合治疗,能够有效控制体重,限制体重反弹,达到减肥的目的。国内外大量研究显示,采用运动综合干预的方法能有效控制和治疗儿童肥胖。

通过为期1.5 a的以学校运动俱乐部的体力活动与健康教

?1994-2015 China Academic Journal Electronic Publishing House. All rights reserved. http://www.cnki.net

育相结合的干预,干预组学生体重、腰围及体脂率平均增长值均低于对照组,其中干预组男生体重平均增长值明显低于对照组,干预组女生身高平均增长值明显高于对照组。28.8%的干预组儿童干预后肥胖等级下降1个等级,12.1%干预组儿童由干预前的肥胖状态恢复为正常。表明本研究对儿童肥胖的控制取得了一定效果,实现了控制肥胖、超重儿童体重的目的。

3.2 儿童肥胖与学校运动干预 学校是儿童成长过程必经之处,也是儿童就餐和活动的场所,教师具备对儿童进行饮食和运动教育的最有力条件。因此,学校拥有运动场地和人员管理等多方面的优势,充分利用学校有利于控制学生肥胖的人文环境和物质环境,使肥胖儿童在不脱离学生日常生活的情况下,通过适合于儿童身心健康运动项目的设计,激发儿童参与并坚持运动的积极性,增强对肥胖危害的认识,使其逐渐在自然状态下养成良好的饮食和运动习惯,进而从根本上达到控制体重、促进健康的目的。研究表明,各种针对高危儿童青少年的以学校为基础的肥胖干预计划是成功的,并且能普及到大量需要预防肥胖的儿童。运动干预儿童肥胖在运动方式上,应选择长时间、全身参与的有氧运动项目,如长跑、散步、游泳、踢球、跳绳、接力跑、骑自行车和娱乐性比赛等[6]。

在运动强度的要求上国内外的研究虽没有一致的要求,但都选择以运动心率作为评价运动强度的标准,运动心率可精确地反映身体工作状况和运动强度,并且心率容易测定监控。总体上,肥胖儿童进行减肥运动时的运动心率的下限都控制在120次/min,上限都在160次/min,也有专家采用靶心率对肥胖儿童运动强度进行控制[7]。这些研究都取得很好的效果,实现了控制儿童肥胖的目的。

本研究利用学校的资源优势和肥胖儿童集中的优点,开展肥胖儿童俱乐部活动,在放学后1 h的时间内对肥胖儿童进行运动干预。由体育教师组织管理,有效利用时间;以体育游戏为主要运动内容,根据儿童的个性特点,调动肥胖儿童参加运动的积极性;利用心率表和记步器进行监控,将运动强度严格控制在120~160次/min最佳心率范围内,使运动量保持在合理的范围内。结果表明,学校运动干预有效控制了肥胖儿童的体重。在这个过程中为学生创造了良好的锻炼氛围,调动全校学生的运动积极性,收到了很好的效果。

3.3 儿童肥胖与家庭运动干预 家庭是儿童生活的主要场所,家庭环境是影响儿童肥胖发生、发展的最重要因素,是预防和治疗儿童肥胖的一个关键。肥胖儿童体重控制依赖于家庭成员的整体参与,在学校运动干预活动中取得的效果也需要家庭的干预活动进行保持。家庭支持在预防和治疗儿童肥胖中具有重要作用,尤其是在对低年龄组儿童肥胖的防治过程中。家庭运动干预的内容应该多安排一些家庭成员共同参与的体育运动,例如社区和学校组织的亲子运动会、周末户外活动等;家庭成员共同参与家庭运动干预,在对肥胖儿童进行干预的同时也在一定程度上改变了肥胖儿童家庭的生活方式和健康观念,使整个肥胖儿童家庭朝着健康的方向发展。控制儿童肥胖的关键是将对肥胖儿童干预活动转变成为肥胖儿童自觉参与的生活习惯,家庭运动在这个过程中起着决定性的作用。

综上所述,儿童肥胖与学校、家庭关系密切。虽然采取学校运动俱乐部干预效果良好,但由于肥胖是多种因素所致,实施综合干预方法应包括学校、社会、家庭的共同努力,才能真正达到防治儿童肥胖的实效。

4 参考文献

[1] 杨志勇,侯洪,任建苹. 影响儿童肥胖症的因素及对策探讨. 张家口医学院学报,2001,6:23-25.
[2] 叶超群,康玉华. 肥胖症的运动疗法. 现代康复,2001,5(9):7-9.
[3] 陈明达,主编. 实用体质学. 北京:北京医科大学有、中国协和医科大学联合出版社,1993.
[4] 中国肥胖问题工作组. 中国学龄儿童青少年超重、肥胖筛查体重指数分类标准. 中华流行病学杂志,2004,25(1):97-102.
[5] 杨红山. 少年儿童单纯性肥胖及其预防. 井冈山医专学报,2001,8(3):62-63.
[6] HAMMER R L, BARRIER C A, ROUNDY E S, et al. Calorie-restricted low-fat diet and exercise in obese women. Am J Clin Nutr, 1989,(49):77-85.
[7] BOUCHARD C A, TREMBLAY JP, NADEAU A, et al. Long-term exercise training with constant energy intake: Effect on bodycomposition and selected metabolic variables. Int J Obes, 1990(14):57-73.

收稿日期:2011-04-02;修回日期:2011-05-25

(上接第1337页)

[11] WANG Y. Is obesity associated with early sexual maturation? A comparison of the association in American boys versus girls. Pediatrics, 2002,110(5):903-910.
[12] HARRIS MA, PRIOR JC, KOEHOORN M. Age at menarche in the Canadian population: Secular trends and relationship to adulthood BMI. J Adolesc Health, 2008,43(6):548-554.
[13] BORDINI B, ROSENFIELD RL. Normal pubertal development: Part I: The endocrine basis of puberty. Pediatr Rev, 2011,32(6):223-229.
[14] ADAIR LS, GORDON-LARSEN P. Maturational timing and overweight prevalence in US adolescent girls. Am J Public Health, 2001, 91(4):642-644.
[15] 刘莉,姚兴家,白英龙,等. 不同体成分10岁男童性发育与体脂及睾酮水平追踪研究. 中国学校卫生,2006,27(4):289-290.
[16] DELYPERE JP, VERDONCK L, VERMEULEN A. Fat tissue: A steroid reservoir and site of steroid metabolism. J Clin Endocrinol Metab, 1985,61(3):564-570.
[17] 乔晓红,俞建,谢晓恬. 青春期发育提前女童行为问题的病例对照研究. 中国心理卫生杂志,2008,22(4):249-252.
[18] MOTL RW, BIRNBAUM AS, KUBIK MY, et al. Naturally occurring changes in physical activity are inversely related to depreesive symptoms during early adolescence. Psychosom Med, 2004,66(3):336-342.
[19] TROST SG, SALLIS JF, PATE RR, et al. Evaluating a model of parental influence on youth physical activity. Am J Prev Med, 2003,25(4):277-282.
[20] 郝加虎,陶芳标. 青春发动时相提前与终身健康. 中国学校卫生, 2008,29(5):388-390.
[21] 刘宝林. 儿童少年生长发育研究的回顾与展望. 中国学校卫生, 2006,27(1):1-2.

收稿日期:2011-08-01

?1994-2015 China Academic Journal Electronic Publishing House. All rights reserved. http://www.cnki.net
