# Supplementary material for: Systematic review and meta-analysis of school-based obesity interventions in mainland China
Source: PLoS One. 2017 Sep 14;12(9):e0184704. doi: 10.1371/journal.pone.0184704 (PMC5598996; doi:10.1371/journal.pone.0184704)
Supplement: S1 Dataset — (ZIP) [file pone.0184704.s007.zip › S1_dataset/76库/42.pdf]

[文章编号]　1001－7062(2006)04－0361－03　[中图分类号]　R 153.2　[文献标识码]　B

[调查研究]

卢湾区小学生肥胖与营养不良干预研究

盛大膺, 徐慧华, 刘淮玉, 胡国泉

[摘要]　目的　通过对肥胖、营养不良学生的调查, 进行综合性干预研究, 并对预防、控制小学肥胖与营养不良提出建议。　方法　选取卢湾区两所小学 1～5 年级学生, 通过营养评价筛选出肥胖、营养不良学生。采取综合干预方法, 对干预组学生进行干预, 研究干预后的效果。　结果　干预组全体学生的营养知识知晓率干预前为 77.25%, 干预后知晓率上升到 99.23%。　结论　综合干预后干预组学生的营养知识各项知晓率均高于对照组, 干预组通过综合干预, 肥胖及营养不良得到改善, 实验方法切实可行。

[关键词]　肥胖; 营养障碍; 干预研究; 儿童保健服务

随着我国人民生活水平的逐步提高, 学生营养状况得到很大改善, 但问题依然存在, 不但营养不良没有得到根本解决, 而且出现新的问题, 近年来城市学生中体质量超标、肥胖呈快速增长趋势。儿童青少年的肥胖、营养不良问题目前已是全社会共同关注的问题。为控制这一倾向, 制定有效的干预措施, 我们进行了实验观察, 现报告如下。

1　对象和方法

1.1　研究对象　选择辖区内二所学生生源及学校类型相当的中心小学, 随机选择其中一组为干预组学校, 另一组为对照组学校。将两所学校 1～5 年级共 1 750 名年龄 7～11 岁的学生作为研究对象。在体检的基础上, 按照不同年龄与不同性别, 根据 WHO 营养评价参考标准(身高标准体重法)对两组进行营养评价, 判定学生是否营养不良与肥胖, 选择单纯性肥胖和营养不良的学生作为此次研究人群。

1.2　研究方法　对干预组学校肥胖、营养不良学生进行为期 6 个月的营养知识教育、膳食营养、体育运动等综合性干预[1]。通过营养知识问卷测试, 评价干预前后营养知识知晓水平; 评价干预前后学生营养摄入变化; 通过血清学检查[2], 评价干预前后水平; 通过体检, 评价干预前后营养状况改善情况。

1.3　统计方法　使用 Epidata 6.0 建立数据库, 并用 Excel 进行整理分析。最终数据用 SPSS for Windows 12.0 统计软件进行统计分析。

2　结果

2.1　基本情况　小学生肥胖率为 23.77%(其中男生肥胖率 31.97%, 女生肥胖率 14.95%), 差异有极显著意义($\chi^2=69.90$, $P<0.01$), 男生肥胖率显著高于女生; 小学生营养不良发生率为 7.20%(其中男生营养不良发生率 4.96%, 女生营养不良发生率 9.61%, 差异有极显著意义($\chi^2=15.72$, $P<0.01$), 女生营养不良发生率显著高于男生(表 1)。

表 1　干预前干预组与对照组学校学生肥胖与营养不良现况

对象	受检人数		肥胖人数		肥胖率(%)		营养不良人数		营养不良率(%)	
	男	女	男	女	男	女	男	女	男	女
干预组	577	543	205	90	35.53	16.57	22	42	3.81	7.73
对照组	330	300	85	36	25.76	12.00	23	39	6.97	13.00
合计	907	843	290	126	31.97	14.95	45	81	4.96	9.61

2.2　营养知识知晓水平分析　在营养知识知晓方面, 通过问卷调查, 小学生干预组全体学生的知晓率干预前为 77.25%, 通过健康教育、座谈会等系列干预后知晓率上升到 99.23%, 差异有极显著意义($u=16.04$, $P<0.01$)。其中习惯嗜好行为认知从 87.16% 提高到 99.44%, 肥胖认知从 70.49% 提高到 98.92%, 营养知识从 70.37% 提高到 99.36%, 运动认知从 88.12% 提高到 99.12%, 差异均有极显著意义(u 值分别为 4.50、10.93、12.45、2.85、16.04; P 均<0.01)。尤其营养知识认知率提高特别显著。对照组问卷调查知晓率为 77.29%, 经常规教育后知晓率上升到 80.64%, 差异无显著意义($u=1.97$, $P>0.05$, 表 2)。

表 2　学生营养知识认知干预前后比较(百分率)

项目	干预组			对照组		
	前	后	u 值	前	后	u 值
习惯嗜好行为	87.16	99.44	4.50	85.35	90.28	1.42
肥胖认知	70.49	98.92	10.93	70.97	71.35	0.12
营养知识	70.37	99.36	12.45	70.02	75.95	7.16
运动认知	88.12	99.12	2.85	91.95	91.69	0.05
全校(均数)	77.25	99.23	16.04	77.29	80.64	1.97

2.3　膳食与体锻习惯分析　对干预组 124 名肥胖及营养不良儿童进行膳食嗜好等内容调查, 喜欢油炸

[基金项目]　上海市家庭教育研究会重点立项课题
[作者单位]　上海市卢湾区疾病预防控制中心(上海　200025)
[收稿日期]　2006－01－04

© 1994-2014 China Academic Journal Electronic Publishing House. All rights reserved.　http://www.cnki.net

食品、甜食、零食、饮料者分别占 70.30%，55.76%，70.30%，73.33%，居较高水平。认为肥胖、营养不良对生活学习有负面影响者占 68.48%，认为锻炼身体重要者占 92.73%，有锻炼身体习惯者占57.58%（表3）。

表3 干预组肥胖及营养不良学生膳食习惯及体锻情况

项目	人数	百分率%
喜欢油炸食品者	116	70.30
喜欢甜食者	92	55.76
有吃夜宵习惯者	19	11.52
喜欢吃零食者	116	70.30
喜欢喝饮料者	121	73.33
吃营养保健食品者	44	26.67
认为锻炼身体重要者	153	92.73
有锻炼身体习惯者	95	57.58
认为肥胖或营养不良对生活学习有负面影响者	113	68.48

2.4 营养不良、肥胖改善情况 表4 可见，干预组通过干预后的肥胖改善率为 16.88%、营养不良改善率为11.11%；而对照组肥胖改善率为 6%，营养不良改善率为 0%。干预组肥胖改善率与对照组相比差异有显著意义（$\chi^2 = 4.10$，$P < 0.05$）。

表4 二组学生营养不良、肥胖干预前后变化情况（%）

组别	性别	肥胖改善	肥胖未改善	营养不良改善	营养不良未改善
干预组	男	20.71	79.29	0.00	100.00
	女	8.33	91.67	18.18	91.66
	合计	16.88	84.81	11.11	88.89
对照组	男	9.00	90.14	0.00	0.00
	女	0.00	100.00	0.00	100.00
	合计	6.00	93.21	0.00	100.00

2.5 血清学检查 在征得学生家长的同意下对 219 名肥胖、营养不良学生进行空腹血液检查（其中干预组 107 人，对照组 112 人）。有 58 名学生在血糖、血脂、总胆固醇3 项指标中有一项或一项以上超标，异常率为26.48%。其中肥胖学生 47 名（占 81.03%），营养不良学生 11 名（占 18.97%），肥胖学生的血清学检查异常人数大于营养不良学生，在 3 项指标中血糖异常所占比例为最高（表 5）。

表5 219 名营养不良、肥胖学生血清学检查情况

组别	营养不良学生异常率（%）			肥胖学生异常率（%）		
	血糖	血脂	胆固醇	血糖	血脂	胆固醇
干预组	27.59	3.45	0	24.36	16.67	0
对照组	11.76	0	0	13.68	2.10	0

3 讨论

在世界范围内迅速增加的儿童青少年体质量超标与肥胖，不仅对儿童青少年的健康和成长，而且对成年人的健康已构成严重威胁。我国儿童青少年肥胖增势迅猛，在学生人群中营养状况出现两极分化，一方面肥胖、体质量超标发生率显著增长，一方面营养不良依然普遍存在。肥胖发生率已从 2000 年的16.13%上升到 2004 年的 23.77%，而营养不良发生率盘垣在7.09%。实验研究干预组通过了健康教育，膳食指导，运动指导，综合干预等一系列措施后所取得的效果，是本次实验研究的重点。

3.1 健康教育 开展以提高学生生活技能为核心的健康教育活动，帮助他们尽快建立健康的生活行为方式。我们在营养知识测试的基础上，针对知识贫乏的程度，编写培训资料、宣传资料、健康指导等。提高学校领导和教师对合理营养与健康的体格发育关系到学生智能发育的认识，教育学生学习营养知识、体力活动是学校教师的责任。通过各种媒体，进行科学“减肥”指导，干预后知晓率上升到 99.23%。

3.2 膳食指导 儿童青少年处于生长发育旺盛时期，要在保证体格的不断发育中控制和预防向肥胖发展，是一个比较复杂的问题。本次调查显示，肥胖学生的脂肪、蛋白质摄入显著高于《中国居民膳食营养素参考摄入量》提供的“摄入参考标准”。营养不良学生则蛋白质摄入显著高于“摄入参考标准”，总热能的摄入显著低于“摄入参考标准”。为此，从改善饮食结构着手，根据《中国居民膳食营养素参考摄入量》编制合理可行膳食处方，请家长密切配合。同时，对肥胖、营养不良学生及其家长讲解膳食摄入原则、热量控制的方法，培养学生养成良好的饮食习惯。

3.3 运动指导 儿童青少年的肥胖除父母肥胖遗传外，静态的生活方式、课余生活的单调即缺乏体育锻炼动机及体育锻炼的不足、户外活动少是造成肥胖的主要原因[3]。综合干预首推儿童饮食干预、体育锻炼干预。我们结合学校的体育活动时间，在有效的锻炼时间内，对干预组学生增加锻炼的强度和密度。并编制运动处方，由家长督促让学生回家锻炼，做好记录。健康行为方式的养成、经常锻炼身体的良好习惯则有预防疾病、提高生活质量的意义。干预组肥胖改善率与对照组相比有显著差异。

3.4 血清学检测 如果在儿童时期不能有效的控制肥胖，可以导致糖尿病、高血压及心血管疾病危险因素聚集。本次在征得学生家长的同意下对 219 名肥胖、营养不良学生进行空腹血液检查血糖、总三酰甘油、总胆固醇，3 项指标中有一项或一项以上超标，异常率为 26.48%。主要为血糖异常，占异常人数的72.41%，尤以低血糖为主，但也不可忽视占血糖异常人数的 9.52%的高血糖的存在；总三酰甘油异常占27.59%。干预组肥胖学生总三酰甘油异常明显高于

© 1994-2014 China Academic Journal Electronic Publishing House. All rights reserved. http://www.cnki.net

营养不良学生，亦高于对照组，差别有极显著意义(χ^2 =11.19，P<0.01)。

[参 考 文 献]

[1] 季成叶. 中国青少年生长与营养状况变化的改善策略措施研究[J]. 北京大学学报(医学版)，2002，34(4)：525—529.

[2] 翟凤英，张李伟，王春荣，等. 国际生命科学会中国肥胖问题工作组推荐体重指数分类标准的血脂谱验证[J]. 中国流行病学杂志，2004，25(2)：117—119.

[3] 张江虹，王芳芳，许言午. 小学生饮食知识、态度、行为干预实验研究[J]. 中国公共卫生，2003，19(2)：1401—1401.

[编辑]　樊继忠

[文章编号]　1001—7062(2006)04—0363—01　[中图分类号]　R445.1　[文献标识码]　D

[疾病控制]

某学院职工体检腹部超声检查结果分析

李明霞

[关键词]　健康状况；超声检查

近年来随着人们生活水平的不断提高和对自身健康的重视，健康体检越来越受到重视。为了掌握职工的健康状况，提高健康水平，做到早预防早治疗，我校职工于2005年10月在南通大学附属医院体检中心做了全面的检查。在体检中，肝、胆、脾、肾的许多隐匿性疾病通过腹部超声检查被发现。本文对腹部超声检查结果进行了分析，结果报告如下。

1　对象和方法

1.1　对象　2005年10月我校职工515人参加健康体检，其中男性280人，女性235人，最大年龄61岁，最小年龄23岁。

1.2　方法　由南通大学附属医院体检中心B超室对腹部肝、胆、脾、肾进行常规检查。

2　结果

2.1　超声对腹部疾病检出情况　腹部超声检查出肝脏疾患3种，占57.08%，检出胆囊疾患4种占71.26%，肾脏疾患占11.84%，脾脏疾患1种占1.74%(表1)。

表1　南通航院教职工腹部B超主要疾患检出率

病种	检出例数	检出率(%)
脂肪肝	213	41.36
肝囊肿	63	12.23
肝血管肿	18	3.49
胆结石	64	12.43
胆囊胆固醇沉着症	35	6.79
胆囊息肉	7	1.35
胆囊炎	261	50.67
肾囊肿	8	1.55
肾结石	52	10.09
肾积水	1	0.19
脾肿大	9	1.74

3　讨论

本次体检人员是在职职工。由于人们生活习惯的改变，社会活动的增加以及环境因素的影响，大部分所谓健康人群其实已处于亚健康状态。从检查结果来看，我校职工脂肪肝的患病率为41.36%，该患病率明显高于上海市成人脂肪肝病患率20.82%的报道[1]。这可能是由于受检人群不同的结果，本文受检者为大专院校的职工，而前者的受检人群是社区的一般居民。有长期饮酒、喜食动物内脏、高脂肪食品习惯的人群易患脂肪肝。本文显示胆囊炎的患病率为50.67%，急性囊炎是常见的急腹症之一，细菌感染、胆石梗阻、缺血和胰液反流是本病的主要诱因。肝血管瘤是肝脏最常见的良性肿瘤，本文资料患病率为3.49%。小血管瘤无症状，如有肝区不适、胀痛等症状常提示血管瘤增大。本文肾结石的患病率为10.09%。一般认为肾结石引起尿路阻塞或肾盂、输尿管平滑肌强烈收缩则产生肾绞痛。肾石常与肾盏或肾盂扩张合并存在，并可继发尿路感染。此次体检发现的一例肾积水病例就是由肾结石梗阻而引起的。本文脾肿大的患病率为1.74%。脾肿大的病因很多，感染、脾淤血、血液病、脾肿瘤与脾囊肿等，均可引起脾脏急性、暂时性和慢性、持久性肿大。本文肝囊肿、肾囊肿的患病率分别为12.23%和1.55%。一般所指的囊种均为先天性囊肿，是生理性退行性病变。

本文结果分析表明，由于工作压力过大，行业竞争激烈，在健康人群中，许多人多已处于亚健康状态。定期进行健康检查尤其是腹部超声检查能及时发现许多隐匿性病灶。使广大职工能够得到及时的预防和治疗。

[参 考 文 献]

[1] 范建高，朱军. 上海市成人脂肪肝患病率及其危险因素的流行病学调查[J]. 中华肝病杂志，2005，13(2)：83—88.

[编辑]　李虎

[作者单位]　南通航运职业技术学院(江苏　南通　226006)

[收稿日期]　2006—02—22

© 1994-2014 China Academic Journal Electronic Publishing House. All rights reserved.　http://www.cnki.net
