# Supplementary material for: Systematic review and meta-analysis of school-based obesity interventions in mainland China
Source: PLoS One. 2017 Sep 14;12(9):e0184704. doi: 10.1371/journal.pone.0184704 (PMC5598996; doi:10.1371/journal.pone.0184704)
Supplement: S1 Dataset — (ZIP) [file pone.0184704.s007.zip › S1_dataset/76库/43.pdf]

文章编号：1006-3617(2013)05-0333-05 中图分类号：R179 文献标志码：A

【论著】

上海市某区小学生超重肥胖的干预效果

杨凡[1]，王文娴[1]，郭红卫[1]，朱检[2]，骆旋[2]，李彦荣[1]

摘要：［目的］评价儿童超重肥胖的干预效果，培养小学生健康的生活方式。［方法］采用整群随机抽样方法，在上海市松江区抽取 4 所小学，其中 3 所作为干预校实施干预措施，1 所学校不进行干预作为对照。在每所学校 2~4 年级中，每年级随机抽取 2 个班的小学生做效果评价。对 1 067 名学生（干预组 833 人，对照组 234 人）实施为期 1 年的干预。干预前、后对所有研究对象及其家长、教师进行肥胖相关知信行问卷调查，同时对小学生进行体格检查。［结果］干预组小学生的肥胖情况得到一定改善，干预前、后干预组的肥胖率分别是 11.5% 和 9.4%。干预组小学生血红蛋白平均值上升了 1.25 g/L。干预组小学生、家长及教师的肥胖相关知识、态度、行为得分的增长率高于对照组，干预组的得分增长率分别为：小学生 8.2%、9.7%、10.0%；家长 4.6%、2.6%、5.7%；教师 8.6%、6.9%、11.0%。［结论］营养健康教育及身体活动干预对控制小学生肥胖可起到一定作用，但需长期坚持并评价其效果。

关键词：儿童肥胖；学校；营养健康教育；评价

Effects of Comprehensive Intervention on Overweight or Obese Students of Primary Schools in a District of Shanghai

YANG Fan[1], *WANG Wen-xian*[1], *GUO Hong-wei*[1], *ZHU Jian*[2], *LUO Xuan*[2], *LI Yan-rong*[1] (1.*Key Laboratory of Public Health Safety of Ministry of Education/School of Public Health, Fudan University, Shanghai* 200032, *China*; 2.*Songjiang District Center for Disease Control and Prevention, Shanghai* 201620, *China*). *Address correspondence to GUO Hong-wei, E-mail: hwguo@shmu.edu.cn* · *The authors declare they have no actual or potential competing financial interests.*

Abstract: [Objective] To evaluate the comprehensive intervention effects on children's overweight and obesity, and develop a healthy lifestyle for children. [Methods] Four primary schools were chosen from Songjiang District of Shanghai with stratified cluster random sampling, in which three schools were assigned as the intervention groups, and the left one was the control group. A total of 1 067 children (833 in the intervention group, 234 in the control group) from 2 classes at grades 2 to 4 of each school took part in the intervention project which lasted for 1 year. All the subjects and their parents and teachers were invited to complete a questionnaire on obesity-related knowledge, attitudes, practice (KAP), and physical examinations were conducted to the participated students before and after the intervention project. [Results] After the intervention, the obesity prevalence in the intervention group was lowered from 11.5% to 9.4%. The mean value of hemoglobin increased by 1.25 g/L in the intervention group. The score increment rates of obesity-related knowledge, attitudes, practice of primary school students (8.2%, 9.7%, 10.0%), parents (4.6%, 2.6%, 5.7%), and teachers (8.6%, 6.9%, 11.0%) in the intervention group were higher than those of the control group. [Conclusion] Nutrition and health education and physical intervention have worked effectively to improve obesity control in primary school students, and a longer term of intervention is required for further effect evaluation.

Key Words: children obesity; school; nutrition and health education; evaluation

儿童肥胖无论在发达国家还是发展中国家都呈迅速上升趋势[1]，已成为全球瞩目的公共卫生问题。2004 年国际肥胖专家工作组（International Obesity Taskforce，IOTF）在提交给世界卫生组织的报告中估计，全球范围有 10% 的学龄儿童超重或肥胖，单独肥胖率为 2%~3%[2]。随着国民经济发展和人民生活水平的提高，我国大陆儿童少年的营养状况有很大改善，同时由于膳食结构和生活方式的变化致使儿童超重肥胖人数大幅度上升，已经逐渐影响到儿童的身心健康，以往某些在成年后才出现的慢性疾病如高血压、血脂异常、糖尿病等已开始在儿童中发生。如不及时采取有效的干预措施，将会严重影响国民身体素质和社会发展。因此，对儿童肥胖的研究应及时转移到切实可行的肥胖防控方面。有学者对 57 项儿童肥

[作者简介] 杨凡（1988—），女，硕士生；研究方向：小学生营养健康教育；E-mail：10211020025@fudan.edu.cn
[通信作者] 郭红卫教授，E-mail：hwguo@shmu.edu.cn
[作者单位] 1. 复旦大学公共卫生学院，教育部公共卫生安全重点实验室，上海 200032；2. 松江区疾病预防控制中心，上海 201620

?1994-2015 China Academic Journal Electronic Publishing House. All rights reserved. http://www.cnki.net

胖干预研究进行系统综述，发现儿童肥胖的单一干预措施效果不明显，而综合干预效果较好[3-4]。本课题以学校为平台开展以营养健康教育和"快乐十分钟"身体活动为主要干预措施的研究，并在此基础上评价其干预效果和可行性，探索对儿童肥胖的有效干预模式。

1 对象与方法

1.1 研究对象

采用整群随机抽样方法，在上海市松江区抽取 4 所小学，在每所学校 2~4 年级中，每年级随机抽取 2 个班的小学生作为评价对象。采用随机对照干预试验实施干预，其中 3 所作为干预学校进行营养健康教育和身体活动干预，一所作为对照学校不实施任何干预。

1.1.1 抽样方法 在上海市松江区，用抽签随机法随机抽取 4 所小学。纳入标准：(1) 选取非寄宿制公立小学；(2) 学校中学生超重肥胖率达到 10% 以上；(3) 学校采用统一供餐且学生在校吃午餐的比例超过 50%。

在选中的小学中，用抽签随机法从各年级（2~4 年级）中随机抽取 2 个班学生。剔除标准：(1) 患有严重疾病，如先天性心脏病，体内有固定支架或进行过关节置换手术等，不能承受剧烈身体活动和膳食控制；(2) 过去 1 年内参加过或将来 1 年内拟参加其他干预研究项目，如营养健康教育、身体活动干预等相关研究。

1.1.2 样本量计算 采用公式 $N=[(Z_{\alpha/2}+Z_{\beta})\sigma/\delta]^2(Q_1^{-1}+Q_2^{-1})$ 计算样本量，其中 α=0.05，β=0.10，σ=6.12，δ=1.26，Q_1=0.75，Q_2=0.25，计算得到 N=986。考虑到 10% 的失访率，每所学校 270 人，在 3 个年级（2~4 年级）中每个年级保证 90 人（2 个班级的人数）。

1.2 研究方法

1.2.1 问卷调查 干预前、后对所有小学生及其家长、教师进行肥胖相关知信行问卷调查。小学生肥胖相关知识、态度、行为（KAP）问卷共分为学生、家长、教师 3 类，由中国疾病预防控制中心营养与食品安全所专家设计，并经预调查。问卷统一编码，经调查员讲解后，学生及教师问卷当场填写并收回，家长问卷于第 2 天收回。收集问卷时由调查员检查问卷完成情况。

1.2.2 体格检查 干预前、后对所有小学生进行体格检查。测量人员进行统一培训后，对小学生进行身高体重测量。同时，每个年级抽取 1 个班，在获得家长知情同意书后，进行血红蛋白（Hb）检测。

1.2.3 干预内容 干预措施包括营养健康教育和身体活动干预两方面，干预时间为 1 年。在干预学校的干预措施包括：每天五分钟营养知识广播，每天"快乐十分钟"身体活动 1 次，每周 1 次营养知识黑板报，2 周 1 次营养健康教育课，每月 1 次营养知识贴画，每学期 2 次营养知识讲座，干预期间进行 1 次征文比赛和 1 次知识竞赛；每学期对教师和家长进行 1 次营养知识讲座，发放营养与健康相关宣传材料。在干预前，对教师进行营养健康教育课和"快乐十分钟"身体活动的培训。在干预期间，不定期地对所选学校进行干预措施的督导，并填写督导记录表。

1.3 肥胖标准

根据"中国学龄儿童青少年超重、肥胖筛查体质指数分类标准"判断本次调查对象的超重、肥胖状况[5]。

1.4 统计分析

用 EpiData 3.0 建立数据库并双录入研究数据，用 SPSS 16.0 进行统计学分析。对于基本情况进行统计描述，计数资料、有关率的比较运用卡方检验。对于干预效果评价，数值型变量进行 t 检验，首先进行 Levene 方差齐性检验，若方差不齐采用方差不齐条件下的 t 检验；分类变量资料采用卡方检验。

2 结果

2.1 两组基本状况

干预组研究对象 833 人，男生 464 人，女生 369 人；92 人超重，96 人肥胖，超重肥胖率为 22.6%。干预后，剔除转学、体检当天请假导致身高体重信息缺失等情况的学生外，研究对象共计 806 人，失访 27 人，失访率 3.2%。对照组研究对象 234 人，男生 132 人，女生 102 人；18 人超重，12 人肥胖，超重肥胖率为 12.8%；无失访。干预组与对照组小学生性别、年龄、超重肥胖状况构成差异无统计学意义（P>0.05），两组均衡性较好，见表 1。

2.2 两组小学生干预后超重肥胖状况变化

由表 2 可见，干预后体质指数正常小学生所占比例在干预组中上升 1.3%，而在对照组中下降 5.9%；干预组中超重小学生比例上升 0.9%，肥胖率下降 2.1%，而对照组中超重小学生比例上升 5.5%，肥胖率上升 0.4%，但差异均无统计学意义。经过 1 年干预，干预组小学生 Hb 平均值上升了 1.25 g/L。

?1994-2015 China Academic Journal Electronic Publishing House. All rights reserved. http://www.cnki.net

表 1　上海市某区小学生超重肥胖干预前基本状况比较

Table 1　Comparison of baseline information of overweight and obese students of primary schools in a district of Shanghai

组别 (Group)	性别 (Gender) 男 (Male)	女 (Female)	年龄 (Age, $\bar{x}\pm s$, years)	体质指数 (BMI, $\bar{x}\pm s$)
对照组 (Control group)	132	102	8.17±0.92	16.06±2.14
干预组 (Intervention group)	464	369	8.13±0.96	16.56±2.67
χ^2	0.037		0.593	2.160
P	0.847		0.553	0.062

表 2　上海市某区小学生干预前、后超重肥胖状况比较 (%)

Table 2　Comparison of overweight and obesity condition before and after intervention of primary school students in a district of Shanghai

超重肥胖情况 Overweight and obesity condition	对照组 Control group				干预组 Intervention group			
	干预前 Pre-internvetion (n=234)	干预后 Post-internvetion (n=234)	χ^2	P	干预前 Pre-internvetion (n=833)	干预后 Post-internvetion (n=806)	χ^2	P
正常 (Normal)	87.2	81.3	2.743	0.098	77.4	78.7	0.438	0.508
超重 (Overweight)	7.7	13.2	1.204	0.272	11.0	11.9	0.333	0.564
肥胖 (Obesity)	5.1	5.5	0.042	0.837	11.5	9.4	1.938	0.164

2.3　干预对小学生肥胖相关 KAP 得分的影响

由表 3 可见，干预前，干预组知识、态度得分略高于对照组 (P<0.01) ；而干预组与对照组间行为得分差异无统计学意义。干预期间，干预组 KAP 得分分别增长了 0.84、3.72、1.79 分，对照组得分增长均低于干预组。干预后，干预组与对照组小学生 KAP 得分间差异均具有统计学意义。干预组干预前、后比较，各项得分差异具有统计学意义，对照组行为得分干预前后差异无统计学意义。问卷调查还显示，干预组小学生在干预前获得营养知识的途径排列前三位的分别是 ：家长 (64.7%)、健康教育课 (62.5%) 和电视广播 (57.8%) ；而干预后分别是 ：健康教育课 (84.8%)，报刊、杂志、书籍 (70.0%) 及家长 (69.5%) ；对照组小学生在干预前后获得营养知识的途径排列前三位的均是家长、电视广播和健康教育课。以上结果表明学校健康教育课的开展使学生获得了较多的营养知识。

表 3　上海市某区小学生干预前、后肥胖相关 KAP 得分

Table 3　Comparison of KAP scores before and after intervention of primary school students in a district of Shanghai

项目 Items	对照组 Control group (n=229)				干预组 Intervention group (n=786)			
	干预前 (分) Pre-internvetion	干预后 (分) Post-internvetion	提高量 (分) Score increment	提高率 (%) Growth rate	干预前 (分) Pre-internvetion	干预后 (分) Post-internvetion	提高量 (分) Score increment	提高率 (%) Growth rate
知识 (Knowledge)	9.59±1.31	10.19±1.21$^{\triangle}$	0.60	6.3	10.23±1.50*	11.07±0.88$^{*\triangle}$	0.84	8.2
态度 (Attitude)	36.67±5.76	38.63±4.07$^{\triangle}$	1.96	5.1	38.17±5.39*	41.89±3.20$^{*\triangle}$	3.72	9.7
行为 (Practice)	17.60±4.56	18.18±4.33	0.58	3.3	17.84±3.65	19.63±3.87$^{*\triangle}$	1.79	10.0

[注] * ：与对照组比较 (Compared with the control group)，P<0.01 ；△ ：与干预前比较 (Compared with pre-intervention)，P<0.01。

2.4　干预对家长肥胖相关 KAP 得分的影响

完成干预前和干预后问卷调查的家长共有 1 012 人 (干预组 790 人，对照组 222 人)。干预前，干预组与对照组的家长构成差异无统计学意义 (P>0.05)，见表 4。文化背景以初中居多 (51.2%)，其次是高中/中专 (30.0%)。所从事职业以生产运输设备操作人员及有关人员居多(38.8%)，其次为商业、服务人员(34.5%)。两组间家长文化背景、从事职业构成差异无统计学意义 (P>0.05)。由表 5 可知，干预前，干预组 KAP 得分与对照组差异均无统计学意义。干预期间，干预组 KAP 得分分别增长了 0.49、1.03、0.98 分，对照组得分增长均低于干预组。干预后，干预组与对照组小学生态度、行为得分间差异具有统计学意义。干预组干预前后比较各项得分差异具有统计学意义，对照组仅有知识得分差异有统计学意义。

表 4　上海市某区小学生超重肥胖干预前家长类型构成比较

Table 4　Comparison of baseline information of the parents of primary school students in a district of Shanghai

组别 Group	父亲 Father	母亲 Mother	父母共同 Parents	其他 Others	χ^2	P
对照组 (人) Control group	86	103	22	11	6.484	0.090
干预组 (人) Intervention group	322	393	49	26		

?1994-2015 China Academic Journal Electronic Publishing House. All rights reserved.　http://www.cnki.net

表5　上海市某区小学生家长干预前、后肥胖相关 KAP 得分比较

Table 5　Comparison of KAP scores before and after intervention of the parents of primary school students in a district of Shanghai

项目 Items	对照组 Control group (n=222)				干预组 Intervention group (n=790)			
	干预前（分）Pre-internvetion	干预后（分）Post-internvetion	提高量（分）Score increment	提高率（%）Growth rate	干预前（分）Pre-internvetion	干预后（分）Post-internvetion	提高量（分）Score increment	提高率（%）Growth rate
知识（Knowledge）	10.42±1.32	10.84±1.07△	0.42	4.0	10.54±1.25	11.03±1.02△	0.49	4.6
态度（Attitude）	39.19±3.87	39.57±3.39	0.38	1.0	39.89±4.26	40.92±3.27*△	1.03	2.6
行为（Practice）	16.80±4.36	17.00±3.81	0.20	1.2	17.34±3.83	18.32±4.23*△	0.98	5.7

［注］*：与对照组比较（Compared with the control group），P<0.01；△：与干预前比较（Compared with pre-intervention），P<0.01。

2.5　干预对教师肥胖相关 KAP 得分的影响

共有 235 名教师完成了干预前和干预后的问卷调查，男性 78 人，女性 157 人，平均年龄（37.62±8.86）岁。干预前，干预组与对照组教师在性别、年龄、受教育程度上构成差异无统计学意义（P>0.05），见表 6。由表 7 可见，干预前，干预组 KAP 得分与对照组差异均无统计学意义。干预期间，干预组 KAP 得分分别增长了 0.90、2.76、1.70 分，对照组得分增长均低于干预组。干预后，干预组与对照组小学生知识、行为得分差异具有统计学意义（P<0.01）。干预组干预前后比较各项得分差异具有统计学意义（P<0.01），对照组各项得分差异均无统计学意义。

表6　上海市某区小学教师干预前性别、年龄、教育程度构成比较

Table 6　Comparison of gender, age, and educationa at baseline of the teachers of primary school students in a district of Shanghai

组别 Group	性别 Gender		年龄（$\bar{x}\pm s$，岁）Age（$\bar{x}\pm s$，years）	受教育程度 Education		
	男 Male	女 Female		中专 Technical Secondary school	大专 College	本科及以上 Bachelor and above
对照组 Control group	24	32	35.54±8.97	2	11	43
干预组 Intervention group	54	125	37.03±9.03	5	32	142
χ^2	8.424		0.654	0.141		
P	0.054		0.730	0.932		

表7　上海市某区小学教师干预前、后肥胖相关 KAP 得分比较

Table 7　Comparison of KAP scores before and after intervention of the teachers of primary school students in a district of Shanghai

项目 Items	对照组 Control group (n=56)				干预组 Intervention group (n=179)			
	干预前（分）Pre-internvetion	干预后（分）Post-internvetion	提高量（分）Score increment	提高率（%）Growth rate	干预前（分）Pre-internvetion	干预后（分）Post-internvetion	提高量（分）Score increment	提高率（%）Growth rate
知识（Knowledge）	10.85±1.01	10.96±0.87	0.11	1.0	10.47±1.35	11.37±0.73*△	0.9	8.6
态度（Attitude）	41.44±3.01	42.13±2.28	0.69	1.7	39.77±2.29	42.53±1.95△	2.76	6.9
行为（Practice）	15.09±3.19	15.71±3.92	0.62	4.1	15.42±4.31	17.12±3.89*△	1.70	11.0

［注］*：与对照组比较（Compared with the control group），P<0.01；△：与干预前比较（Compared with pre-intervention），P<0.01。

3　讨论

此次调查是由中国疾病预防控制中心营养与食品安全所牵头，联合全国各地共 7 个单位合作，共同开展以膳食营养为主的综合干预关键技术随机对照研究。作为全国调查的一部分，本课题是上海市某区的调查结果，可能不能完全代表上海市的情况，旨在评估干预技术对儿童营养知识、态度，饮食行为及肥胖率的作用，为全国儿童肥胖的防控提供科学依据和政策建议。

肥胖不仅给儿童的日常生活、学习带来诸多不便，还可对儿童健康产生各方面的影响。本研究结果显示，干预组和对照组超重率都有所上升，但对照组上升幅度（5.5%）明显大于干预组（0.9%）。干预组肥胖率下降了 2.1%，而对照组上升了 0.4%，表明干预措施有一定的减慢肥胖率上升的作用。干预前、后比较无统计学差异，可能由于小学生正处于生长发育期，身高、体重等指标受发育影响显著，因此，超重肥胖率改变情况不明显；也可能与干预时间不够长有关。结果提示，以学校为基础开展营养健康教育和“快乐十分钟”身体活动来干预防控儿童超重和肥胖有一定作用，但需要长期坚持，并需要继续探索其效果和可行性。此外，干预组小学生 Hb 平均值上升了 1.25 g/L，而贫血的主要原因之一是膳食不平衡，加上孩子们的偏食、挑食的习惯，使得某些营养素摄入不足。说明通过营养健康教育，这些情况有了一定程度的改善。

?1994-2015 China Academic Journal Electronic Publishing House. All rights reserved.　http://www.cnki.net

学校作为儿童获得营养知识和养成正确行为的重要场所，对提高儿童的营养健康水平起着至关重要的作用[6]。但教育方式及内容形式上需根据该年龄段学生的特点进行。考虑到小学生的年龄小，理解力和接受与自控力差，在营养健康教育课和营养知识讲座的课件中插入了动画、卡通图片，尽量采用通俗、浅显易懂的语言，并注意讲课内容的重复性，积极与学生互动，并按时巩固复习[7]。

小学生在营养知识得分上，干预组增长量略高于对照组，其中干预后膳食宝塔知晓情况的人数百分比（97.1%）显著高于干预前（49.3%），差异有统计学意义，而对照组的差异无统计学意义。同时，干预组小学生在干预后获得营养知识最主要的途径由家长转变为健康教育课，而对照组小学生在干预前后均为家长。结果表明，营养健康教育课对小学生获得营养知识有重要的作用。有关态度和行为得分方面，干预组小学生的增长率（9.7%、10.0%）显著高于对照组（5.1%、3.3%），其中一周吃早餐次数、一个月吃西餐次数干预组干预前后的差异均有统计学意义，而对照组差异无统计学意义。以上结果说明通过一系列的干预活动，在改善学生的营养知识、态度、行为方面已经取得了一定的效果。

在对小学生进行营养健康教育的同时，对干预组家长及教师也分别展开了每学期一次的营养知识讲座和培训，并发放营养健康相关的宣传材料。对家长及教师的肥胖相关 KAP 问卷调查结果显示，在营养知识、态度、行为得分上干预组的增长率均高于对照组。干预组家长在炒菜用油、膳食宝塔知晓情况、为孩子准备早餐情况、1 个月吃西餐次数、1 周是否参加中等强度活动等方面的前后差异均有统计学意义。干预组教师在膳食宝塔知晓情况、1 周饮奶次数、1 个月吃西餐次数等也有了不同程度的改善。由于家长和教师是小学生行为的师者，因此对家长和教师宣传营养健康知识的意义不可忽视，并可以通过他们对小学生进行营养健康知识的传播，从而加深儿童的记忆和理解。

调查显示：仅仅通过 1 年的干预还不足以完全改变小学生所有的不良饮食习惯和生活方式，但干预的效果已经初步显现出来。大部分儿童肥胖综合干预研究的效果评价是在干预刚结束或者干预后的 1 年之内，也有研究对儿童肥胖干预的长期效果进行评价[8-9]。应当将遏制儿童肥胖作为一项长期任务，将营养健康教育和体育活动干预结合起来，以学校为基础平台，借助政府、媒体和医疗力量，通过家长及教师配合等，多管齐下，控制儿童肥胖的快速增长，提高我国儿童的健康水平。

（志谢：本次研究得到中国营养学会“中国儿童营养教育项目”资助，在此表示衷心感谢！）

·作者声明本文无实际或潜在的利益冲突。

参考文献：

[1] 陈芳芳，米杰. 儿童肥胖的评价标准及流行现状 [J]. 实用儿科临床杂志，2007，22（23）：1837-1840.

[2] LOBSTEIN T，BAUR L，UAUY R，et al. Obestiy in children and young people：a crisis in public health [J]. Obes Rev，2004，5（suppl 1）：4-104.

[3] FLODMARK C E，LISSAU I，MORENO L A，et al. New insights into the field of children and adolescents' obesity：the European perspective [J]. Int J Obes Relat Metab Disord，2004，28（10）：1189-1196.

[4] VAN SLUIJS E M F，MCMINN A M，GRIFFIN S J. Effectiveness of interventions to promote physical activity in children and adolescents：systematic review of controlled trials [J]. BMJ，2007，335（7622）：703.

[5] 中国肥胖问题工作组. 中国学龄儿童青少年超重、肥胖筛查体重指数值分类标准 [J]. 中华流行病学杂志，2004，25（2）：97-102.

[6] 石建辉，刘秀荣，田向阳，等. 北京市小学生肥胖干预效果分析 [J]. 中国健康教育，2004，20（9）：782-785.

[7] 王芸，袁媛，徐贵发. 以学校为平台的儿童肥胖防控效果评价 [J]. 中国卫生事业管理，2012，29（4）：317-319.

[8] FITZGIBBON M L，STOLLEY M R，DYER A R，et al. A community based obesity prevention program for minority children：rationale and study design for Hip- Hop to Health Jr [J]. Prev Med，2002，34（2）：289-297.

[9] FITZGIBBON M L，STOLLEY M R，SCHIFFER L，et al. Two-year follow-up results for Hip2-Hop to Health Jr：a randomized controlled trial for overweight prevention in preschool minority children [J]. J Pediatr，2005，146（5）：618-625.

（收稿日期：2012-09-27）

（英文编审：金克峙；编辑：张晶；校对：洪琪）

?1994-2015 China Academic Journal Electronic Publishing House. All rights reserved.　http://www.cnki.net
