# Supplementary material for: Systematic review and meta-analysis of school-based obesity interventions in mainland China
Source: PLoS One. 2017 Sep 14;12(9):e0184704. doi: 10.1371/journal.pone.0184704 (PMC5598996; doi:10.1371/journal.pone.0184704)
Supplement: S1 Dataset — (ZIP) [file pone.0184704.s007.zip › S1_dataset/76库/45.pdf]

【调查研究】

文章编号：1004-6194(2009)05-0505-03

快乐十分钟干预对小学生肥胖相关指标的影响

常改，刘昊，杨溢，马洁，王文娟，江国虹

摘要：目的　探讨"快乐十分钟"活动对小学生肥胖相关指标的影响。方法　于 2007 年选取天津市 3 所小学，其中 2 所为干预校，1 所为对照校。在干预校中开展为期 1 a 的"快乐十分钟"干预活动，并于干预前后进行体格测量和实验室检查，通过指标比较来评价干预效果。结果　干预后干预校学生的收缩压、舒张压、腰臀比、空腹血糖、甘油三酯和胰岛素水平低于对照组 (P<0.05)；干预校体重正常学生的比例上升 (P<0.05)，轻、重度肥胖比例下降 (P<0.05)，血清低密度脂蛋白下降而高密度脂蛋白水平上升 (P<0.05)。结论　在学校中采用"快乐十分钟"干预，可以改善小学生肥胖相关指标。

关键词：肥胖；指标；干预；小学生；快乐十分钟

中图分类号：R589.2　　文献标识码：A

Effect of "Take 10! " Intervention on the Related Indexes of Obese Pupils *CHANG Gai, LIU Hao, YANG Yi, et al.*

Tianjin Center for Disease Control and Prevention, Tianjin 300011, China

Abstracts:Objective To observe the effect of "Take 10!" intervention on the related indexes of obese pupils. **Methods** Of Three elementary schools selected in Tianjin were, two were intervention schools and one was control school. "Take 10! " interventions were implemented in intervention schools for one year and the intervention results were observed at the end of the intervention. **Results** The levels of systolic pressure, diastolic pressure, WHR, fast blood glucose, triglyceride, insulin in intervention schools were obviously lower than those in control school after intervention (P<0.05). The proportion of normal body weight of students in intervention schools increased obviously (P<0.05), but ratios of the mild and severe obesity reduced obviously (P<0.05). The LDL level reduced, but HDL increased(P<0.05). **Conclusion** It is an effective and feasible method to improve the related indexes of obesity with "TAKE 10! "interventions on pupils.

Key Words：Obesity; Related indexes; Intervention;Pupil; "TAKE 10! "

儿童期肥胖是导致成人期心脑血管疾病的独立危险因素之一，大量研究显示，肥胖与遗传、缺乏体力活动、不合理的膳食结构、社会生活环境等因素相关[1]。我国学龄儿童经常参加体育锻炼的比例仅为 5.4%，而 6~17 岁学龄儿童久坐少动者占 94.1%，导致我国儿童肥胖率呈逐年上升趋势[2]。因此，开展儿童肥胖干预、控制儿童体重对于预防成人心脑血管疾病具有重要意义。笔者于 2007 年 5 月—2008 年 5 月在天津市 3 所小学校中开展了"快乐十分钟"活动，旨在进行小学生肥胖相关指标的干预研究，并对 1 a 后干预效果进行了分析。

作者单位：天津市疾病预防控制中心 非传染性疾病预防控制所，天津 300011

作者简介：常改 (1960-)，女，主任医师，从事慢性非传染性疾病研究。

本次调查结果显示，男性吸烟率和现在吸烟率高于 2002 年全国或江苏省男性平均水平，与北京调查的结果相似[2-5]。女性吸烟率较低，但高于同期在该省其他城市四类人群 (医生、教师、公务员和服务人员) 中的调查结果 (0.3%) [6]。戒烟率较低，其中 40 岁以上成人的戒烟率高于<40 岁者，与其他研究结果相似[7]。无论性别、年龄或文化程度，成人被动吸烟率均在 50%以上，与 2002年全国平均水平相似，高于北京的调查结果[2-5]。

对吸烟危害相关知识的调查发现，90%以上的被访者均知晓"吸烟或者被动吸烟有害健康"，然而对于被动吸烟与具体危害的知晓率却相对较低，仅有 53.2%的被访者完全了解其危害。支持全部室内尤其是酒吧和餐厅禁烟的比例则更低。杨功焕[8]建议应通过各种途径向大众传播吸烟危害知识，其中，在烟草包装上的健康警语采用图片的形式，可以较直观地传达吸烟有害健康的知识，提高吸烟者 (包括低文化水平者) 对其健康风险的认识[9]。此外，保护人们免受烟草烟雾危害也是重要策略之一。从我国各地的公共场所禁止吸烟规定来看，大部分地区的规定里涉及的公共场所范围尚未不包括办公室、餐厅和酒吧等被动吸烟现象较严重的场所[10,11]。

本研究还发现，正确的认知和态度可以减少吸烟率，而被动吸烟率仅与正确的态度有关，说明从认知到态度还需要一个过程，需要来自政府、社会、个人等各方面的长期努力。

参考文献：

〔1〕世界卫生组织. 世界卫生组织烟草框架公约〔R〕. 瑞士:日内瓦, 2003:8.
〔2〕李玉清, 刘秀荣, 韩梅, 等. 北京市公共场所成人被动吸烟及其危害认知情况〔J〕. 中国慢性病与预防与控制, 2008,16(3):461-464.
〔3〕李玲, 陈秋霖, 贾瑞雪, 等. 我国的吸烟模式和烟草使用的疾病负担研究〔J〕. 中国卫生经济 2008,27(1):26-30.
〔4〕顾东风, Jiang H, 吴锡桂, 等. 中国成年人主要死亡原因及其危险因素〔J〕. 中国慢性病预防与控制, 2006,14(3):149-154.
〔5〕杨功焕, 马杰民, 刘娜. 中国人群 2002 年吸烟和被动吸烟的现状调查〔J〕. 中华流行病学杂志, 2005, 26(2):77-83.
〔6〕覃玉, 潘晓群, 向全永, 等. 江苏省不同职业人群吸烟行为、认知和态度分析〔J〕. 中国慢性病预防与控制,2008,16(5):465-468.
〔7〕杨焱, 姜垣, 杨小丽, 等. 影响戒烟成功因素的分析——中国 2002年戒烟竞赛一年随访研究〔J〕. 卫生研究,2004,33(4):478-480.
〔8〕杨功焕. 烟草对健康的危害及控烟策略〔J〕. 中国慢性病预防与控制,1999,7(3):97-99.
〔9〕2008 年世界卫生组织全球烟草流行报告. MPOWER 系列政策[R]. 日内瓦: 世界卫生组织, 2008.
〔10〕杨焱, 姜垣, 吴曦, 等. 我国公共场所禁烟政策及其执行状况分析〔J〕. 中国健康教育, 2008,24(9):657-660.
〔11〕覃玉, 周明浩, 许国强,等. 2007 年江苏省部分市县公共场所禁止吸烟规定及现状调查〔J〕. 中国健康教育 ,2008,24(9):693-695.

(收稿日期：2009-07-22 修回日期：2009-08-17)

(本文编辑：黄丽媛)

?1994-2014 China Academic Journal Electronic Publishing House. All rights reserved. http://www.cnki.net

1 对象与方法

1.1 对象 在天津市城区选取规模相当、课程设置(包括体育课)和课间安排相同的 3 所市区小学,其中河西区闽侯路小学和塘沽区广州道小学为干预校,河东区互助道小学为对照校。研究对象为 3 所小学校中 1~5 年级学生。

1.2 方法

1.2.1 体检方法 于干预前后对 3 所学校所有 1~5 年级学生进行身高、体重、腰围、臀围、血压的检查,计算腰臀比(WHR),同时测量生物电阻,计算体脂含量。并在每所学校各随机抽取 90 名学生,由三级甲等医院医务人员采集晨起空腹血 5 ml,进行空腹血糖(FBG)、甘油三酯(TG)、总胆固醇(TC)、高密度脂蛋白(HDL)、低密度脂蛋白(LDL)、胰岛素、血红蛋白(HB)的生化检测。采用由中国疾病预防控制中心提供的生物电阻抗仪(RJLsystem 101 USA)对学生进行生物电阻的测定,计算体脂含量,公式如下:

生物电阻=(电阻抗 ^{2}+电容抗 ^{2})×0.5

去脂体重=0.406×身高(cm)^2/生物电阻+0.360×体重(kg)+5.580×身高(cm)/100+0.56×性别(男=1,女=0)−6.48

体脂百分比=(体重−去脂体重)/体重

1.2.2 干预方法 按照 2007 年中国疾病预防控制中心《快乐十分钟工作手册》对干预校小学生进行行为干预,由教师带领进行的一些简单、中高等强度的体力活动,也可由学生自主创造参与编制(需经审核,微微出汗,达到中高等强度),每个学习日至少开展 1 次,不代替学校原有的体育课或课间操。干预时间为 1 a。

1.3 指标判定标准 超重、肥胖:采用中国肥胖问题工作组中国学龄儿童青少年超重、肥胖筛查体质指数值(BMI)分类标准[3];按照儿童青少年体脂肪含量判定肥胖标准将干预对象判定分成正常、轻度、中度、重度肥胖组[4];儿童高血压:目前我国还没有统一标准,本次研究采用美国疾病预防控制中心推荐的儿童高血压诊断标准,收缩压(SBP)≥120 mm Hg 或舒张压(DBP)≥80 mm Hg[5]。

1.4 质量控制 对体检人员统一培训,统一调查方法和调查表格,统一测量仪器。在"快乐十分钟"干预过程中由班主任带作;每班选出活动监查员在特定的活动记录卡中记录;组织班级间的互评和观摩活动;监察队定期对各班干预活动进行评比检查并收集反馈意见;在干预活动开展的中期对决策者(学校领导)、执行者(教师)、家长、学生及相关部门的相关人员(教委、保健所、校医等)进行问卷访谈,了解干预活动开展情况,发现问题及时解决,以保证干预的质量。

1.5 统计学分析 采用 SPSS 13.0 进行分析。同一组干预前后比较采用配对 t 检验,干预前及干预后二组间比较采用独立样本 t 检验,两组间率的比较用 χ^2 检验。

2 结果

2.1 研究对象的基本情况 干预前共有研究对象 1 707 人(对照组 762 人,干预组 945 人),超重率为13.4%,肥胖率为 15.5%;其中男生 860 名,女生 847 名,男生超重率和肥胖率分别为13.4%,16.8%,女生超重率和肥胖率分别为11.6%,13.1%。男生肥胖率高于女孩(χ^2=26.081,P=0.000)。

因终末调查时有人失访,经过整理、保留基线和终末调查前后两次资料均齐全的小学生共 1 674 人,其中对照组 754 人,干预组 920 人,两组间性别、年龄差别无统计学意义(分别为 χ^2=0.224,P=0.636;χ^2=4.279,P=0.118)。

2.2 两组干预前后超重率及肥胖率 由表 1 可见,两组干预前超重率和肥胖率差别无统计学意义(P>0.05),干预后超重率和肥胖率亦无统计学意义(P>0.05),且干预前后各组自身对照差别亦无统计学意义(P>0.05)。

表 1 两组干预前后超重率与肥胖率 (%)

组别	超重率	肥胖率
干预组(n=920)		
干预前	12.1	14.7
干预后	12.5	14.3
对照组(n=754)		
干预前	12.7	15.2
干预后	10.7	14.6

干预前干预组的体重正常学生比例低于对照组(P<0.05),中、重度肥胖学生的比例高于对照组(P<0.05)。对照组干预后重度肥胖学生比例升高(χ^2=7.765,P<0.05),干预组正常学生比例上升(χ^2=23.977,P<0.05)、轻、重度肥胖学生比例下降(χ^2 值分别为 4.534,5.167, P<0.05),见表 2。

表 2 两组干预前后肥胖水平分布情况 (%)

组别	体重正常	轻度肥胖	中度肥胖	重度肥胖
干预组(n=920)				
干预前	39.5	42.0	8.9	9.5
干预后	37.1	37.9	10.7	14.2^a
对照组(n=754)				
干预前	23.9^b	43.9	14.2^b	17.9^b
干预后	36.6^a	39.0^a	11.8	12.5^a

注:^{a}与同组干预前比较,^{b}与同期对照组比较,经 χ^2 检验,P<0.05。

2.3 两组干预前后体格测量指标 干预前,干预组和对照组的高血压患病率分别为 5.9%和 5.6%,干预后分别为 6.1%,7.7%,差别无统计学意义(P>0.05)。由表 3 可见,干预组身高、体重及 BMI 比干预前升高(t 值分别为−81.669,−32.991,−7.283,P<0.05),SBP 和 WHR 下降(t 值分别为 2.721,3.134,P<0.05);对照组身高、体重、BMI、SBP、DBP 比干预前升高(t 值分别为−84.849,−27.55, −8.278, −5.762, −19.169,P<0.05)。干预组干预后的 SBP、DBP、WHR 低于对照组(t 值分别为 2.349,2.446,−15.095,P<0.05)。

2.4 两组干预前后血生化指标 干预前共检测 270 人(对照组 90 人,干预组 180 人),干预后干预组失访 13 人,共检测 257 人(对照组 90 人,干预组 167 人)。两组性别、年龄差别无统计学意义(分别为 χ^2=0.027,P=0.870;χ^2=4.650,P=0.589)。

由表 4 可见,干预前,超重和肥胖儿童的 TC、LDL、TG、胰岛素水平高于体重正常儿童(t 值分别为−3.903,−3.822,−4.182, −4.544, P<0.05),而 HDL 低于体重正常儿童(t=5.700,P<0.05)。

由表 5 可见,与干预前比较,干预组学生的 LDL 下降(t=9.626,P<0.05),HDL 升高(t=−2.039,P<0.05),而对照组学生 FBG、TG 升高(t 值分别为−4.611,−2.201, P<0.05)。干预后干预组FBG、TG、胰岛素低于对照组(t 值分别为 3.433,2.613,3.923,P<0.05)。

3 讨论

本研究显示,男生肥胖率高于女生,可能与男生膳食中摄入的高能量食品较多有关;干预组和对照组的高血压患病率分别为 5.9%和 5.6%,低于北京地区小学生高血压患病率 11.18%[6]。超重和肥胖儿童的 TC、LDL、TG、胰岛素水平高于体重正常儿童(P<0.05),HDL 低于体重正常儿童(P<0.05),进一步证明了肥胖与血脂异常患病的高风险关系,与其他研究结果一致[7]。

?1994-2014 China Academic Journal Electronic Publishing House. All rights reserved. http://www.cnki.net

表 3 两组干预前后体格测量指标 ($\bar{x}\pm s$)

组别	身高(cm)	体重(kg)	BMI(kg/m²)	WHR	SBP(mm Hg)	DBP(mm Hg)	体脂百分比(%)
干预组(n=920)							
干预前	136.8±11.1	34.0±12.0	17.74±4.09	0.84±0.06	94±11	56±10	22.3±8.6
干预后	143.1±11.3[a]	38.5±13.2[a]	18.37±4.11[a]	0.86±0.06	96±11[a]	64±8[a]	24.0±8.3[a]
对照组(n=754)							
干预前	136.7±10.2	34.4±11.1	18.05±3.79	0.85±0.07	96±11	62±8[b]	25.2±8.0[b]
干预后	144.1±10.9[a]	39.3±12.7[a]	18.57±4.11[a]	0.80±0.06[ab]	94±12[ab]	62±8[b]	24.0±8.0[a]

注：[a]与同组干预前比较，[b]与同期对照组比较，经 t 检验，P<0.05。

表 4 干预前不同 BMI 水平学生的血生化指标 ($\bar{x}\pm s$)

组别	FBG(mmol/L)	TC(mmol/L)	HDL(mmol/L)	LDL(mmol/L)	TG(mmol/L)	HB(g/L)	胰岛素(uIU/ml)
体重正常(n=111)	4.67±0.46	4.34±0.80	1.74±0.36	2.45±0.78	0.86±0.41	136±13	8.77±3.51
超重和肥胖(n=146)	4.65±0.50	4.80±1.02[a]	1.49±0.34[a]	2.86±0.90[a]	1.25±0.92[a]	138±24	12.22±6.36[a]

注：[a]与体重正常者比较，经 t 检验，P<0.05。

表 5 两组干预前后血生化指标 ($\bar{x}\pm s$)

组别	FBG(mmol/L)	TC(mmol/L)	HDL(mmol/L)	LDL(mmol/L)	TG(mmol/L)	HB(g/L)	胰岛素(uIU/ml)
干预组(n=167)							
干预前	4.52±0.40	4.67±0.71	1.63±0.63	2.63±0.63	1.07±0.57	140±11	10.21±11.46
干预后	4.88±0.58[a]	4.69±1.10	1.52±0.43	2.52±0.43	1.30±1.17[a]	140±12	10.54±5.46
对照组(n=90)							
干预前	4.73±0.51[b]	4.73±1.04	1.61±0.39	2.72±0.98	1.08±0.86	137±12	9.66±8.33
干预后	4.65±0.46[b]	4.70±1.01	1.66±0.39[a]	1.88±0.50[a]	1.03±0.48[b]	135±23	9.75±5.4[b]

注：[a]与同组干预前比较，[b]与同期对照组比较，经 t 检验，P<0.05。

在进行 1 a 的“快乐十分钟”干预后，无论是干预组还是对照组，学生身高、体重、BMI 均升高，两组指标间差别无统计学意义，与国内其他城市的学生干预(1~4 a 干预时间)效果相同[7]；干预后干预组学生的 WHR、血压、体脂百分比有了明显下降，而对照组干预后 SBP、DBP、体脂百分比均有不同程度的升高。与肥胖相关的生化指标如 TG、LDL、HDL、FBG、胰岛素水平，干预组均优于对照组，表明本次研究采用的干预方法对学生血脂、血糖、胰岛素水平具有明显的改善作用。

本次研究采用了体脂含量的测定指标，避免了因骨骼大、肌肉多、体型粗壮等造成的超重、肥胖的误判，而且该指标具有较好的预测作用。Koutoubi 等[8]和 Pecoraro 等[9]分别用体成分分析仪测量人群体脂和 BMI 值，得出在冠心病风险预测上体脂百分比比 BMI 更有意义。按照儿童青少年体脂肪含量判定肥胖标准将干预对象判定分成正常、轻度、中度、重度肥胖组，干预后干预组中体重正常学生比例上升，轻、重度肥胖比例下降；对照组重度肥胖学生比例升高。

有研究表明，体力活动过少比摄食过多更易引起肥胖[7]。有氧运动可以增加能量消耗，促进脂肪分解，减少体内脂肪的积蓄[10]。本研究采用的“快乐十分钟”活动为中高等强度的体力活动，虽然只有短短的 10 min，1a 后超重率和肥胖率没有明显变化，但 WHR、血压、体脂肪含量、血糖、血脂、胰岛素水平均得到了改善。相信如果坚持更长的时间，超重率、肥胖率会有明显的改变。总之，本研究提示在学校中增加课间体力活动能够改善小学生肥胖相关指标，是减少肥胖率的有效措施之一。

参考文献：

〔1〕刘艳，黄中夯，彭楠，等. 国内外儿童肥胖的病因及评价标准研究〔J〕. 中国校医，2006，20(1)：107.
〔2〕万燕萍，徐仁应，陈之琦，等. 合理营养干预对儿童青少年肥胖病伴高血压的治疗效果〔J〕. 中国临床营养杂志，2006, 14(2): 115-116.
〔3〕中国肥胖问题工作组. 中国学龄儿童青少年超重、肥胖筛查 BMI 值分类标准〔J〕. 中华流行病学杂志，2004，25(2):97-102.
〔4〕叶广俊. 现代儿童少年卫生学〔M〕. 北京：人民卫生出版社，1999：473.
〔5〕诸福堂. 实用儿科学〔M〕. 第 6 版. 北京：人民卫生出版社，1996：1355-1357.
〔6〕王文绢，王克安，陈春明，等. 北京地区儿童青少年体重指数与血压关系的研究〔J〕. 中华流行病学杂志，2004,25 (2):112.
〔7〕田本淳，吕书红，钱玲，等. 我国四城市部分小学生肥胖控制效果评价〔J〕. 中国学校卫生，2006，27 (10)：869-871.
〔8〕Koutoubi S, Huffman FG. Body composition assessment and coronary heart disease risk factors among college students of three ethnic groups〔J〕. J Natl Med Assoc, 2005, 97:386-389.
〔9〕Pecoraro P, Guida B, Caroli M. Body mass index and skinfold thickness versus bioimpedance analysis: BF mass prediction in children〔J〕. Acta Diabetol, 2003, 32:386-389.
〔10〕Keshava C, Frye BL, Wolff MS, et al. Waf-1 (p21) and P53 Poly-mor-phisms in breast cancer〔J〕. Cancer Epidemiol Biomarkers Prev, 2001, 165:59.

(收稿日期：2009-02-15 修回日期：2009-07-01)
(本文编辑：黄丽媛)

?1994-2014 China Academic Journal Electronic Publishing House. All rights reserved. http://www.cnki.net
