# Supplementary material for: Systematic review and meta-analysis of school-based obesity interventions in mainland China
Source: PLoS One. 2017 Sep 14;12(9):e0184704. doi: 10.1371/journal.pone.0184704 (PMC5598996; doi:10.1371/journal.pone.0184704)
Supplement: S1 Dataset — (ZIP) [file pone.0184704.s007.zip › S1_dataset/76库/46.pdf]

孩子相处时间、每日室外活动时间与次数成相关性。这说明气质可受环境因素的影响。而智力发展却只与每日室外活动时间与次数成相关性,与家庭、父母文化背景无明显相关性。考虑这与本文研究对象年龄偏小有关,外界环境刺激作用尚未表现出来,但家庭教养方式对婴儿智力发展的影响却已体现出来,而气质对智力的作用可能更多的是通过影响家庭教养方式起作用。因此,父母应尽早了解儿童的气质特点,根据儿童的气质特点,调整抚育方式、改善亲子关系、减少紧张因素,与儿童进行有效的交流,以促进他们的智力正常发展。

[参考文献]

[1]万国斌,李学荣,龚颖萍. 气质对6~8月龄婴儿智力发展的影响[J]. 中国临床心理学杂志,1997,5(1):17-20.
[2]沈晓明,金星明. 发育和行为儿科学[M]. 南京:江苏科学技术出版社,2003.405.
[3]姚凯南.儿童气质测量及其临床应用[J]. 国外医学妇幼保健分册,2002,13(6):249-251.
[4] Maziade M, Cote, Boutin P. Temperament and intellectual development: a longitudinal study from infancy to four years[J]. Am J Psychiatry, 1987,44(2):144-150.
[5]翁晓萍.婴儿气质、家庭环境与发育的相关研究[J].中国儿童保健杂志,2003,11(2)79-81.
[6]翁晓萍. 婴儿气质[J].国外医学妇幼保健分册,2000,11(3):175-177.

[责任编辑:孙晓勉 刘黎明]

宝安区青春期单纯性肥胖症干预措施的探讨

方 虹,陈爱群
(深圳市宝安区妇幼保健院妇保科,广东 深圳 518133)

[摘 要]**目的** 探讨宝安区青春期单纯性肥胖症的干预措施。**方法** 对宝安区清华实验学校2 041名9~19岁青少年进行调查分析,筛查出单纯性肥胖者328例,将肥胖者随机分为干预组与非干预组,并选正常体重者作对照组,采用饮食控制与调整、体育锻炼、行为矫正以及心理健康指导等综合干预措施进行体重控制两年。**结果** 经过两年干预,干预组的肥胖程度显著下降($P<0.01$),身高增长值与正常对照组相比差异无显著性($P>0.05$);总胆固醇、甘油三脂、低密度脂蛋白、aopB及血压、体重均明显下降($P<0.01$),高密度脂蛋白及aopA则明显升高($P<0.01$)。**结论** 采用饮食控制与调整、体育锻炼、行为矫正及心理健康指导等综合行为干预措施治疗与预防青春期单纯性肥胖症具有重要的临床意义,值得临床广泛推广应用。

[关键词]青春期;单纯性肥胖症;干预措施;探讨
[中图分类号]R179;R723.14 [文献标识码]A [文章编号]1673-5293(2006)04-0255-04

Exploration of interventions for pubertal simple obesity in Bao'an District of Shenzhen city

FANG Hong, CHEN Ai-qun
(*Department of Maternal Health, The Maternal and Child Health Hospital of Bao'an District of Shenzhen, Guangdong Shenzhen* 518133, *China*)

[**Abstract**] **Objective** To explore interventions for pubertal simple obesity in Bao'an district, Shenzhen city. **Methods** 2 041 students from Qinghua experimental school in Bao'an district, Shenzhen city aged 9~19 years old were surveyed. Among them 328 students with pubertal simple obesity were identified and were divided into intervention group and non-intervention group randomly. Meanwhile, the students with normal weight were as the control group. Various measures had been adopted such as dietotherapy, exercise, behavior rectification and psychological guidance to control the weight of obese students for two years. **Results** After being intervened for two years, the obesity degree of students in the intervention group was significantly improved ($P<0.01$). In increase in height, there was no significant difference between the two groups ($P>0.05$). Blood total cholesterol, triglyceride, low density lipoprotein cholesterol, aopB, blood pressure and weight of students in the intervention group lowered significantly ($P<0.01$). While blood high density lipoprotein cholesterol and aopA elevated markedly ($P<0.01$). **Conclusion** The comprehensive interventions including dietotherapy, exercise, behavioral rectification and psychological guidance are of important clinical significance for treating and preventing pubertal simple obesity and can be popularized in clinical work.

[**Key words**] puberty; simple obesity; intervention; exploration

目前肥胖已成为全球性的21世纪青少年期严重的健康问题,肥胖对青少年的身心损害已引起世界各国医学界的重视。近年来由于人们生活水平的不断提高和生活方式的改变,使青少年肥胖症的发生率迅速增高而成为社会广泛关注的焦点,青少年单纯性肥胖症不仅威胁着青少年健康,而且还是导致成年期心血管疾病和内分泌疾病的主要危险因素。由于肥胖对青少年健康带来近期和远期的危害,使得肥胖的预防及其治疗显得极为重要。宝安区处于经济发达城区,生活水平高,青少年肥胖发生率有逐年增加的趋势。为了探索有效的干预及治疗方法,控制我区青少年肥胖的发展,减少其相关疾病的发生,2003年对宝安区清华实验学校2 041名9~19岁的青少年进行调查研究,筛查出单纯性肥胖者328例,经过

[收稿日期]2006-03-17
[作者简介]方 虹(1958-),女,香港九龙人,大专,主治医师,主要从事妇女保健工作。

?1994-2015 China Academic Journal Electronic Publishing House. All rights reserved. http://www.cnki.net

对其进行两年的综合干预研究后，取得一定效果，现将结果报道如下：

1 对象与方法

1.1 对象

选择深圳市宝安区清华实验学校61个班9～19岁的2041名青少年作为调查对象，按WHO标准筛查出单纯性肥胖者328例（肥胖检出率为16.07%）。将肥胖青少年随机分为干预组（165例）及非干预组（163例），两组的基础体重、身高基本匹配，同时选择同班级、同性别、年龄相仿的正常体重的青少年163例作为正常对照组。肥胖诊断及分类是参照WHO肥胖诊断标准，大于标准体重20%为轻度肥胖；大于标准体重30%为中度肥胖；大于标准体重50%为重度肥胖。

1.2 方法

1.2.1 相关项目的测量

严格按专人专项原则测量身高、体重，并根据体重指数（BMI）=体重（kg）/身高^2（cm），计算BMI与肥胖度。脉搏、血压的测量按照全国学生体质健康状况调研检测细则进行。生化检验取清晨空腹静脉血，用生化酶法测定血清总胆固醇（TC），甘油三脂（TG）；高密度脂蛋白（HDL）采用磷钨酸镁沉淀法测；载脂蛋白A和B（apoA、apoB）采用免疫透射比浊法；低密度脂蛋白（LDL）按Firdewaid公式计算得出。并将测量数据资料输入电脑建立个人档案，有专人管理。

1.2.2 制定干预计划

成立减肥领导小组，由区妇保院医生、校医、家长代表组成。通过体格检查及调查问卷等方法了解基础情况，针对每个肥胖生分析其肥胖原因，对轻、中、重度肥胖青少年制定相应减肥计划，根据家庭条件、学校情况、青少年饮食习惯和爱好制定饮食方案、运动方案、行为矫正方案等综合干预措施和目标：轻度肥胖者保持体重不增即可，中、重度肥胖制定短期减肥计划（1个月内减轻体重2kg）与长期减肥计划（2年内减轻体重至正常体重以上10%～20%）。并以区妇保院为指导中心，分学校家庭两级管理，对青少年饮食、运动和生活方式进行监督检查指导。建立定期反馈制度，检查体重变化及计划完成情况，按照循序渐进的总原则随时调整方案，并解释减肥过程中出现的问题，尤其是心理问题。非干预肥胖组与正常对照组按其日常习惯进行饮食和体育锻炼。

1.2.3 干预措施

1.2.3.1 饮食控制与调整　饮食方案的制定与调整采取个体化原则，由专人定期检查。分发日常食物得热卡含量表，根据中国营养学会推荐居民平衡膳食，根据各年龄阶段生长发育的特点，制定膳食表，以高蛋白、低脂肪、低糖饮食为原则，每日能量控制在800～1000千卡之间。合理安排平衡膳食，能量分配为2/6、3/6、1/6，每日三餐，尽量不吃夜宵。并指导家长及青少年饮食以蔬菜、水果、米饭为主，限制肥肉、油炸食品，不吃纯热量食品，如巧克力、糖果、点心等。并适当限盐，多食用绿叶蔬菜，豆腐等体积大热量少的食物。

1.2.3.2 体育锻炼　根据肥胖程度、年龄、性别、健康状况以及社会经济、文化背景等不同情况，按表1进行运动方式选择指导。运动量一般以轻至中度为宜，同时根据肥胖者本人可以耐受的减肥速度，分阶段、循序渐进地提高运动量，要求每人每天至少进行30～60分钟的全身性有氧运动。运动强度可用最大心率来估计，要求为最大心率的65%，最大心率=220－年龄。运动后即测试脉搏应为110次/分左右。同时将各项运动热量消耗表发给肥胖者，要求自行掌握，做好日常活动安排，制定体育锻炼记录，包括日期、运动方式、运动时间、休息时间等内容，并作好记录，由学校及家长进行监督完成。

表1　运动强度及运动方式

运动强度	消耗IU热量所需时间（min）	运动项目
最轻度	30	散步、站立乘车、家务、购物等
轻　度	20	步行、洗澡、体操、自行车等
中　度	10	慢跑、上楼、登山、滑雪等
大强度	5	快跑、跳绳、球类、击剑、健美操等

1.2.3.3 行为矫正　通过与肥胖者、家长、教师交谈及观察分析，寻找致肥胖的主要危险因素，确定需纠正的靶行为，如睡前进食、暴饮暴食，看电视进食。喜欢静坐，不喜欢门外活动等不良行为。

1.2.3.4 心理健康指导　让肥胖者了解肥胖的发病原因及有关影响因素，取得对肥胖症的正确认识，消除可能存在的病理心理状态，建立起康复的自信心，通过心理转化的方式，使肥胖者消除自卑、胆怯、孤独等不良情绪，建立良好的心境。采用强减肥行为的方式，对不认真执行减肥方案者个给予批评教育，消除过分依赖思想。

1.3 统计分析

调查及监测数据经检查核对后，全部输入微机，建立数据库，采用Spss11.0统计软件进行统计学处理分析。

2 结果

2.1 宝安区青少年肥胖症的检出率

宝安区青少年肥胖发生率为16.07%，男生高于女生，差异具有统计学意义（$P<0.01$），特别在轻度肥胖上男女差异更大（见表2）。

表2　宝安区青少年肥胖症的检出情况

性别	检测人数（n）	肥胖症检出人数及检出率（%）			
		轻度	中度	重度	合计
男	998	87（8.72）	68（6.81）	33（3.31）	188（18.84）
女	1043	56（5.37）	59（5.66）	25（2.40）	140（13.42）
合计	2041	143（7.01）	127（6.22）	58（2.84）	328（16.07）
χ^2		8.78	1.17	1.53	11.09
P		<0.01	>0.05	>0.05	<0.01

2.2 肥胖组青少年与正常对照组青少年各项检测指标比较

肥胖组青少年TG、TC、体重、BMI、血压、LDL、apoB均较正常对照组青少年明显增高，而HDL、apoA明显降低，两组比较有显

?1994-2015 China Academic Journal Electronic Publishing House. All rights reserved.   http://www.cnki.net

著性($P<0.01$),两组身高相仿,无统计学意义(见表3)。

表3 肥胖组与正常对照组青少年各项检测指标比较($\bar{X}\pm S$)

观测指标	肥胖组(n=328人)	正常对照组(n=163人)	U	P
TG(mmol/L)	2.27±0.71	0.80±0.12	26.23	<0.01
TC(mmol/L)	5.42±1.72	3.08±0.30	23.92	<0.01
HDL(mmol/L)	1.18±0.13	1.99±0.31	-31.99	<0.01
LDL(mmol/L)	3.09±1.37	2.01±0.58	12.24	<0.01
apoA(mmol/L)	131.04±8.82	159.39±11.12	-28.41	<0.01
apoB(mmol/L)	80.16±8.48	70.29±5.61	15.37	<0.01
收缩压(kPa)	16.44±1.65	14.31±1.21	16.20	<0.01
舒张压(kPa)	11.38±1.93	9.81±1.23	10.93	<0.01
体重(kg)	67.71±17.25	40.67±8.51	23.26	<0.01
体重指数	28.92±4.13	21.09±2.90	24.33	<0.01

2.3 干预后肥胖度

肥胖干预组青少年经综合性干预后,其肥胖度较干预前明显下降,差异显著,($P<0.05$)。而肥胖非干预组无明显下降($P>0.05$)。两组比较差异显著($P<0.01$)。

同时干预组体重呈缓慢增长趋势。肥胖干预组、肥胖非干预组和正常对照组身高增长幅度基本一致($P>0.05$)(见表4)。

表4 肥胖干预组与非干预组肥胖度变化人数比较

组别	观测人数	轻度	中度	重度
干预组	165			
干预前		75	65	25
干预后		21	33	11
U		6.40	3.78	2.33
P		<0.01	<0.01	<0.05
非干预组	163			
干预前		68	62	33
干预后		56	56	24
U		0.93	0.47	0.74
P		>0.05	>0.05	>0.05
两组比较				
χ^2		42.38	23.67	4.96
P		<0.01	<0.01	<0.05

2.4 干预前后各项检测指标的变化

肥胖干预组综合干预后TC、TG、LDL、apoB、血压、体重均较干预前明显降低,而HDL、apoA明显升高,差异有显著性($P<0.01$)。而肥胖非干预组及正常对照组上述指标变化不明显($P>0.05$)。肥胖干预组综合干预后各项检测指标与正常对照组比较,差异无显著性,无统计学意义($P>0.05$);而与肥胖对照组各值比较,则差异有显著性统计学意义($P<0.01$)(见表6)。

表5 三组干预前后检测项目的变化($\bar{X}\pm S$)

组别	体重(kg)	TG(mmol/L)	TC(mmol/L)	HDL(mmol/L)	LDL(mmol/L)	ApoA(mmol/L)	ApoB(mmol/L)	收缩压(kPa)	舒张压(kPa)
干预组									
干预前	63.71±11.23	2.33±0.94	5.34±1.53	1.19±0.27	3.06±1.45	129.06±6.72	80.76±8.32	15.71±1.42	11.24±1.36
干预后	42.73±5.32	1.02±0.13	3.34±0.21	1.79±0.63	2.16±0.33	151.13±6.33	70.84±5.02	13.83±1.54	9.78±1.28
U	21.69	17.73	16.63	-11.24	7.36	-30.71	13.11	11.31	10.04
P	<0.01	<0.01	<0.01	<0.01	<0.01	<0.01	<0.01	<0.01	<0.01
非干预									
干预前	61.37±10.31	2.17±0.56	5.47±1.37	1.15±0.33	3.13±1.09	133.13±0.53	80.03±7.86	15.34±1.51	11.53±1.27
干预后	60.41±10.57	2.34±0.67	5.33±1.62	1.12±0.48	3.34±1.61	134.31±6.31	81.02±8.01	15.91±1.72	11.67±1.42
U	0.83	-2.49	-0.36	0.66	-1.38	-2.38	-1.13	-3.18	-0.94
P	>0.05	>0.05	>0.05	>0.05	>0.05	>0.05	>0.05	>0.05	>0.05
对照组									
干预前	40.67±8.51	0.80±0.12	3.08±0.30	1.99±0.31	2.01±0.58	159.39±11.21	70.29±5.61	14.31±1.21	9.81±1.23
干预后	41.56±7.31	0.85±0.31	3.17±0.29	1.97±0.27	2.04±0.61	60.11±10.2	70.67±5.83	14.11±1.34	9.73±1.13
U	-1.04	-1.24	-2.75	0.62	-1.06	-0.61	-0.60	1.414	0.61
P	>0.05	>0.05	>0.05	>0.05	>0.05	>0.05	>0.05	>0.05	>0.05

注:肥胖非干预组与正常对照组均口头给予健康教育干预

2.5 行为矫正

干预组肥胖青少年对有关肥胖的认知度及行为等方面均有不同程度的提高。卫生保健知识增加,不良行为习惯、饮食习惯有了改善。改掉懒惰行为,增强自信心,克服自卑心理,保持良好的心理健康状态;同时体育成绩也相应提高。而肥胖对照组以上各项变化均不显著,未有一定规律可循。

3 讨论

近年来,青少年肥胖症的发病率在全球范围呈明显上升的趋势。统计资料显示青少年单纯性肥胖症有80%将过渡到成人肥胖症,是动脉粥样硬化的高危人群[1]。在如今的疾病谱和死亡谱中,与肥胖有关的疾病已经高居榜首[2],因此对肥胖青少年进行干预治疗,被视为减少成人疾病发生率和死亡率的重要预防措施。目前寻找切实可行的干预措施,是医务工作者的方向,我国学者在青少年的干预方面进行了许多有益的探索[3]。经调查表明,宝安区青少年肥胖症主要是由不良饮食习惯、运动少以及遗传因素等所致。为控制青少年肥胖症的发展趋势,探讨其干预方法,本研究采用饮食控制与调整、体育锻炼、行为矫正、心理健康指导等综合干预方法进行为期两年的行为干预。经过两年综合行为干预,结果表明,干预组肥胖度明显下降($P<0.01$),体重呈缓慢增长趋势。而肥胖非干预组肥胖度无明显变化。因此,综合行为干预可以起明显减肥作用。肥胖青少年的血压、TG、TC、LDL、apoB均比正常对照组青少年明显增高,HDL、apoA则降低,经过两年综合干预后,干预组体重、血压、TG、TC、LDL、apoB均明显下降;HDL及apoA则增高,P均小于0.01;而非干预组无明显变化,两组比较,差异有显著性($P<0.01$)。可见综合行为干预可以降低肥胖者的血压、血脂等指标,从而减少肥胖所致的心血管疾病发生的危险因素。身高作为生长发育的观察指标,三组的身高增长比较,差异均无统计学意义($P>0.05$)。显示本干预措施并不因饮食调控而影响被干预学生的生长发育。饮食调整只是使青少年膳食结构更趋合理和科学,而不同于成人饥饿疗法[4],从而保证了干预期青少年的生长发育。同时通过综合行为干预,给肥胖青少年带来多方面的教育,随着卫生常识的增加,青少年选择良好的生活方式,摒弃诸如饮食过量、睡前进食、喜油炸油腻食物、爱吃零食等饮食行为上的不良习惯,改变懒惰行为,爱好体育锻炼,不仅增强了体质,而且有利于减轻心理压力,保持良好心理状态。同时在干预过程中,能给学生带来积极心态、希望和信念,使信心、毅力等个性品质得到培养。

青少年单纯性肥胖症最主要的原因使摄入的能量过多,而消耗的能量少,多余的能量转化成脂肪,储存于体内,造成肥胖。所以治疗上必须以综合治疗为主要方法,既要控制摄入量,又要增加消耗,达到一种负平衡,才能控制体重,但青少年处于生长发育阶段,不能像成人一样严格控制饮食,应给予足够的蛋白质,维生素及微量元素,维持其生长发育的需要,制定好饮食处方,每天热量控制在3 360~4 200KJ,减少高脂肪高热量的饮食,多食高纤维、高蛋白并富含维生素和微量元素的食物,再根据情况制定运动处方,每天坚持有氧运动至少30~60分钟。有氧运动能够有效地控制脂肪的合成并增加脂肪的供能,从而减少脂肪的合成,促进脂肪的消耗[5],所以有氧运动在肥胖运动疗法中占有重要地位。同时在综合干预治疗中行为指导以及心理健康教育同样起很大作用,使肥胖青少年改变不良行为习惯,提高对肥胖症的认知度,保持良好的心理状态,有利于青少年健康成长。

同时在实施干预治疗过程中,强调家长的参与和支持,由于青少年自制力差,要改变不良的行为及生活习惯,必须根据

?1994-2015 China Academic Journal Electronic Publishing House. All rights reserved. http://www.cnki.net

不同家庭的生活环境以及儿童的爱好等方面去制定治疗方案。在饮食方面要制定一个可行的合适青少年生长发育的饮食方案,使之即能控制体重,又能满足儿童生长发育的营养需要,使体重随年龄稳定增长;在行为矫正过程中,在家长协助下创造和建立新的正确行为环境,对青少年进行潜移默化的教育,使之养成良好的行为习惯;在运动锻炼时,要督促青少年掌握一定的运动量,达到一定运动强度,鼓励其坚持进行锻炼,有效地控制肥胖的发展。可见在干预中,家长的参与也相当重要,只有家长参与其中,干预治疗才能持久。

通过本研究结果可见,针对引起我区青少年肥胖的主要因素与遗传、饮食习惯、运动少等有关,要控制及预防青少年的发展,应采用行为矫正、饮食控制与调整、体育锻炼、心理健康教育、以家庭为单位,以日常生活为控制场所,肥胖青少年、家长、教师、医务人员共同参加等多层次的综合行为干预措施。综合行为干预可以有效减低肥胖度,减低肥胖所致心血管疾病发生的危险因素,矫正不良行为习惯,有效控制体重缓慢增长,且不影响青少年的生长发育,具有学生容易接受、疗效确切、安全性好的优点,值得临床推广应用。

[参考文献]

[1]张美云,李立芳. 单纯性肥胖儿童血脂、血液流变学观察和分析[J]. 山西医科大学学报,2001,32(5):466-457.
[2]尚汉冀,朱宝宽. 关于中国人肥胖标准的探讨[J]. 医学研究通讯,2002,31(1):45-48.
[3]冯帮胜,张慧敏,钟群英,等. 健康教育对低龄儿童单纯性肥胖的早期干预研究[J]. 中国妇幼保健杂志,2004,19(10):23-25.
[4]张柏青,刘永鹏,于芳明,等. 儿童单纯性肥胖症防治措施的研究[J]. 中国儿童保健杂志,2001,9(6):373-376.
[5]房冬梅. 单纯性肥胖与有氧运动[J]. 中国临床康复,2003,7(30):4128.

[责任编辑:黄燕苹]

西安市10年孕产妇死亡分析

王晓云,苟文丽
(西安交通大学医学院第一附属医院,陕西 西安 710061)

[摘 要]目的 通过孕产妇死亡资料分析。寻求制定行之有效的干预措施,降低孕产妇的死亡率。**方法** 对西安市1995年~2004年度孕产妇死亡资料进行回顾性研究分析。**结果** 西安市10年内产妇总数为440 890例,孕产妇死亡221例,孕产妇死亡率平均为50.12/10万。孕产妇死亡前三位原因依次是:产科出血、妊娠期高血压疾病、羊水栓塞。**结论** 建立健全急诊产科救治体系是降低孕产妇死亡率的重要措施。

[关键词]产妇死亡率;死因;产科急救体系;预防
[中图分类号]R714 **[文献标识码]**A **[文章编号]**1673-5293(2006)04-0258-03

An analysis of maternal mortality in Xi' an city over a ten-years period.

WANG Xiao-yun, GOU Wen-li
(*The First Affiliated Hospital of Medical College, Xi' an Jiaotong University, Shaanxi Xi' an* 710061, *China*)

[Abstract] Objective To identify risk factors of maternal death and to provide a scientific basis for preventive intervention. **Methods** A retrospective study was conducted to analyze data from maternal death audit of Xi' an city over a period from 1995 to 2004. **Results** The average maternal mortality rate(MMR) over the period from 1995 to 2004 in Xi' an city was 50. 12 /100000(1221 died among 440 890 parturients) . The first three causes of the death were orderly obstetric hemorrhage, pregnancy-induced hypertension and amniotic fluid embolism. **Conclusion** The United Nations Process Indicators for emergency obstetric care (EmOC) should be introduced and used extensively in our country to reduce MMR in Xi' an city and China.

[Key words] maternal mortality rate; death cause; emergency obstetric care system; prevention

母亲和儿童是家庭与社会的基础,他们的健康状况甚至决定着社会的发展进程。孕产妇死亡率 maternal mortality rate, MMR 和儿童死亡率水平是衡量国家经济社会发展和卫生事业发展的重要指标[1]。本文对西安市10年来的产妇死亡情况逐个审核后输入计算机进行数据分析,分析产妇死亡的主要原因,寻求降低产妇死亡率的方法。

1 资料与方法

1.1 研究对象

凡1994年9月至2004年9月户口在西安市的死亡产妇进行逐个分析。孕产妇死亡定义采用WHO标准:妇女从妊娠开始至妊娠结束后42天内死亡者,不论妊娠时间和部位,包括计划外妊娠,但不包含意外死亡。

1.2 方法

由经过培训的妇幼保健医生负责本辖区的孕产妇死亡调查,并填写死亡报告卡,上报市妇保院,由市妇保院专人负责调查审核汇总,每年进行一次漏报调查,市卫生局每年组织专家对死亡病例进行评审,确定死因诊断,提出整改措施。将所有数据输入计算机,用Excel表格进行分析。

[收稿日期]2006-06-20
[作者简介]王晓云(1971-),女,主治医师,在读硕士研究生,主要从事高危妊娠的研究。

?1994-2015 China Academic Journal Electronic Publishing House. All rights reserved. http://www.cnki.net
