# Supplementary material for: Systematic review and meta-analysis of school-based obesity interventions in mainland China
Source: PLoS One. 2017 Sep 14;12(9):e0184704. doi: 10.1371/journal.pone.0184704 (PMC5598996; doi:10.1371/journal.pone.0184704)
Supplement: S1 Dataset — (ZIP) [file pone.0184704.s007.zip › S1_dataset/76库/47.pdf]

深圳市以肥胖控制为切入点发展健康促进学校项目中期效果评价

李　慧[1]，蒋丽娟[1]，常小芳[2]，王文艺[1]

【中图分类号】R151. 1，R193　【文献标识码】A　【文章编号】1002－9982（2004）06－0496－04

【摘要】　目的　通过以肥胖控制为切入点发展健康促进学校模式，逐步摸索出适合中国国情的预防和控制儿童肥胖的途径和方法。方法　按照健康促进学校的六大方面要求，在项目学校中进行创建活动和并重点进行肥胖干预，于干预前后对学生进行问卷和肥胖率调查。结果　与对照学校相比，项目学校学生在知识、态度和行为水平上均有显著提高；一年来项目学校学生肥胖率维持在14.4%，而对照学校学生肥胖率从9.6%上升到13.4%，增长了3.8%（$P<0.05$）。结论　创建活动和肥胖干预工作对学生产生了良好的教育和引导作用，同时控制了学生肥胖率的进一步发展。对学生的肥胖干预是一项长期而艰巨的工程，而来自政府、社会、学校、社区、家庭、个人等多方面的支持必不可少。以肥胖控制为切入点发展健康促进学校模式非常值得作为一项可持续发展的工作继续推行。

【关键词】　健康促进学校；儿童肥胖；中期；效果

A Mid-term Effectiveness of Project *Develop Health Promotion School Taking Obesity Control as Entry Point* in Shenzhen City　*LI Hui, JIANG Li-juan, CHANG Xiao-fang, WANG Wen-yi. Center for Disease Control and Prevention of Shenzhen, Shenzhen* 518020, *China*

【Abstract】　**Objective**　To find out an effective way and method to prevent and control children obesity for China by establishing health promoting school（HPS）. **Methods**　Basing on the six rules of HPS, we have developed HPS and implemented student obesity intervention in the project school and have analyzed the data of questionnaire survey and obesity rate of pupil before and after intervention in the two schools. **Results**　Comparing with the contrast school, the pupil's level of knowledge, attitude and behavior have increased significantly in project school. The obesity rate of pupil maintained about 14.4% in project school but the rate went up from 9.6% to 13.4% in contrast school, the range was 3.8%（$P<0.05$）**Conclusion**　The work of developing HPS and obesity intervention has bought nice education and induction to pupils, meanwhile the obesity rate of pupil in project school has been controlled well. It is a hard work and a long term system project to intervene pupil obesity. The supports from government, society, school, community, family and individual are necessary. The mode of developing HPS and taking obesity control as an entry point is worth considering as a long term work to be carried out.

【Key words】　Health Promotion School；Children Obesity；Mid-term；Effectiveness

中国/WHO以肥胖控制为切入点发展健康促进学校项目于2002年3月正式启动，至2003年3月，该项目实施至中期评估节段，现将深圳地区的部分调查结果报告如下。

对象与方法

1. 对象　项目就深圳市福田区南华小学四、五年级学生，共458名。（基线调查时为468名），对照学校福田区皇岗小学学生。

2. 方法　选择深圳市福田区南华小学为项目实施学校，将该校三、四年级学生（中期调查时为四、五年级）作为重点目标人群。选择同在皇岗小学作为项目对照学校。

（1）通过创建健康促进学校树立健康促进理念，改善学校的物质和健康环境，提高学生的健康知识水平和自我保健能力，改变不健康的生活方式，控制学生肥胖发生和发展，达到提高健康水平的目的。项目学校按照健康促进学校的6大方面要

【作者单位】1　深圳市疾病预防控制中心，广东　深圳　518020；
2　深圳市福田区南华小学，广东　深圳　518000
【作者简介】李　慧（1967－），女，四川成都人，硕士，副主任技师，研究方向：营养与食品卫生、儿童肥胖研究。

?1994-2015 China Academic Journal Electronic Publishing House. All rights reserved.　http://www.cnki.

求进行创建工作，重点落实超重和肥胖学生的干预控制。对照学校只进行调查，不进行干预。

（2）培训：对项目学校和对照学校三、四年级的校医、班主任进行调查前培训。

（3）问卷调查：由中国疾病预防控制中心健康教育所统一提供问卷。由班主任组织学生当堂完成，逐份检查问卷有无遗漏并督促学生补齐后再交给校医，由校医复核并统一编号。所有问卷收齐后交本市项目负责人，由项目负责人安排人员进行问卷录入。问卷回收率为 100％。

（4）重点进行超重和肥胖学生的营养健康知识教育、生活膳食指导、体能训练、体重监测并及时与家长进行沟通商酌。

（5）问卷录入以及分析：在 Epi6.0 系统中进行问卷录入并建立数据库。采用 SPSS10.0 统计软件包进行分析。

结　果

1. 学生营养状况　按照学生营养状况判断标准（北京大学儿少研究所制定）对目标年级学生的营养状况进行评价和比较，结果（见表 1）：

在基线调查中，项目学校目标年级学生肥胖率明显高于对照学校（$P<0.05$），而干预之后，项目学校的肥胖率与对照学校相比差异已经没有显著性（$P>0.05$），但项目学校超重率仍大于对照学校（$P<0.05$）。项目学校在干预后肥胖率和超重率都没有明显变化（$P>0.05$），两个学校的学生营养不良率都有明显下降，而正常体重率明显提高（$P<0.05$）。对照学校学生一年后肥胖率增加 3.8％（$P<0.05$）。

2. 项目学校和对照学校干预前后学生知识、态度、行为的比较。

（1）知识：在学生问卷中关于膳食营养、运动以及肥胖危害等知识题共 13 道（见表 2）。

表 1　学生营养状况比较（％）

营养状况	项目学校—对照学校		基线调查—中期调查	
	基线调查	中期调查	项目学校	对照学校
营养不良	5.8—6.0	2.0—1.6	5.8—2.0*	6.0—1.6*
较低体重	34.7—37.0	25.3—30.1	34.7—25.3*	37.0—30.1
正常体重	38.8—43.0	50.4—50.9	38.8—50.4*	43.0—50.9
超重	6.0—4.5	7.9—4.0*	6.0—7.9	4.5—4.0
肥胖	14.8—9.6*	14.4—13.4	14.8—14.4	9.6—13.4*

注：*经卡方检验，$P<0.05$。

表 2　学生认识水平比较（％）

知　识	项目学校正确率		对照学校正确率	
	干预前	干预后	干预前	干预后
食物金字塔	1.12	29.98**	1.36	1.23
蔬菜主要给身体提供什么营养素	16.67	78.60**	20.00	22.98
经常吃甜食对身体好不好	90.38	98.25**	91.64	90.68
经常吃多油的食物对身体好不好	89.32	98.03**	86.87	89.44
多吃蔬菜对身体好不好	97.22	99.34*	97.61	97.20
挑食的习惯为什么不好	62.82	93.89**	59.40	66.77*
吃粗粮杂粮有什么好处	38.03	84.50**	46.87	52.17
从营养上水果能否代替蔬菜	32.50	91.50**	36.10	28.90*
洋快餐食品的营养学特点	1.50	70.96**	1.19	1.24
吃得过饱对身体的影响	26.07	62.88**	30.15	26.40
每天合适的运动量是多少	13.89	5.90**	20.30	17.81
肥胖对身体的影响	7.27	70.52**	1.49	4.97*
控制体重和减肥的正确措施	14.74	74.02**	8.06	15.53**

注：**表示经卡方检验，$P<0.01$；*表示经卡方检验，$P<0.05$。

?1994-2015 China Academic Journal Electronic Publishing House. All rights reserved.　http://www.cnki.

项目学校学生在干预后，12/13 问题的知晓率都有显著提高，特别是“蔬菜主要给身体提供什么营养素”、“洋快餐食品的营养学特点”以及“肥胖对身体的影响”、“控制体重和减肥的正确措施”等四题分别从 16.67％、1.50％、7.27％和 14.74％上升到 78.60％、70.96％、70.52％和 74.02％；但“每天合适的运动量是多少”略有下降，从 13.89％下降到 5.90％。而对照学校只有 3/13 的问题知晓率有所提高，并且幅度明显低于项目学校。

（2）态度：从两个学校学生态度持有情况来看，表示愿意积极主动学习和选择健康饮食和运动者为大多数，但在干预之后项目学校相比之对照学校正确态度的持有率仍然有明显的提高（见表 3）。

（3）行为：项目学校学生在干预后有 13/15 的题目应答正确率都有所提高，特别是“平时吃最多的食品是米面制品”和“平时不吃或基本不吃零食”的提高程度较大。而对照学校只有一个问题即“吃饭速度适中”略有改善（见表 4）。

(4)学生问卷总分结构比较：学生问卷中涉及到知识态度行为共 33 道题，我们按良好(80 分以上)、中等(60—80 分)、差等(60 分以下)来比较。项目学校在干预后，76.20％学生的答卷分数可以达到 80 分以上，而干预之前只有 5.34％的学生可以达到这一水平($P<0.01$)；而差等的学生比率从干预前的 37.18％下降到干预后的 5.46％($P<0.01$)。对照学校干预前达到 80 分以上的占 5.97％，干预后为 9.32％($P>0.05$)；而总分为差等的学生干预前占 46.87％，干预后仍为 44.41％($P>0.05$)。

表 3　态度持有率比较（％）

态　度	实施学校持有率		对照学校持有率	
	干预前	干预后	干预前	干预后
愿意学习有关营养的知识	88.25	97.82**	87.16	86.96
愿意参加运动锻炼	91.45	97.60**	91.05	87.27
愿意放弃以前常吃的不健康食品	79.10	96.70**	78.20	78.30
愿意吃平时不喜欢的健康食品	74.15	96.29**	63.28	74.84**
愿意每天坚持运动锻炼	81.84	95.20**	77.01	67.08**

注：**表示经卡方检验，$P<0.01$；*表示经卡方检验，$P<0.05$。

表 4　行为形成比较（％）

行　为	实施学校形成率		对照学校形成率	
	干预前	干预后	干预前	干预后
平时吃最多的食品是米面制品	33.97	88.45**	44.48	38.82
平时喝最多的饮料是白开水	84.19	97.60**	75.82	78.57
平时不吃或基本不吃零食	26.28	84.93**	24.18	28.26
每周吃油炸食品少于 1～2 次	84.83	96.07**	88.36	85.09
每周吃肥肉少于 12 次	81.20	95.85**	84.78	84.47
很少或基本不吃洋快餐	55.13	85.37**	41.49	46.27
每天吃较多的蔬菜	59.19	85.15**	54.33	54.35
每天都吃蔬菜	79.27	90.83**	56.12	48.45*
临睡前基本不吃或偶尔吃东西	91.03	97.16**	92.54	95.03
吃饭速度适中	65.81	92.14**	60.90	70.81**
周末和假期睡眠时间 9～11 小时	72.65	94.54**	65.97	72.67
每天在家学习时间 2 小时内	77.14	93.45**	89.85	87.89
体育课外的运动时间在 1 小时/天以上	32.26	41.70**	30.45	30.12
假期看电视和玩电脑在 1.5 小时/以上	31.20	34.93	28.06	26.71
运动量适中	40.81	38.21	43.58	31.68**

注：**表示经卡方检验，$P<0.01$；*表示经卡方检验，$P<0.05$。

?1994-2015 China Academic Journal Electronic Publishing House. All rights reserved.　http://www.cnki.

讨 论

1. 肥胖干预状况 以学校为主体的肥胖干预方式覆盖的人群范围广泛，不仅将肥胖学生作为目标人群，还将超重和非肥胖学生以及学校教职工、学生家长和社区成员也纳入到实施对象之中，这正是健康促进学校的目的所在。在中期调查时，虽然项目学校尚未达到蒋竞雄等人三年期研究中学生肥胖率从 16.9%下降至 12.1%的效果[4]，但比较对照学校肥胖率的大幅飙升，足以说明这种群体肥胖干预产生了一定的效果。值得注意的是两所学校在中期调查时均呈现学生营养状况向体重增加方向发展的趋势，这警示我们学生的肥胖态势仍未得到有效控制。另外，儿童肥胖的日趋低龄化必将给以学校为主战场的学生肥胖干预工作增加极大的难度。

2. 两校学生知、信、行水平比较 过去人们往往生硬地用单一的评价指标——肥胖率的下降来评价措施的干预效果，这是十分片面和不科学的，其实不良饮食和运动行为的改变以及对干预控制方法的有效掌握远远较体重下降更为重要，因为学生正确意识的树立和健康生活方式的形成是控制肥胖和保证效果的前提和基础[5]。

在实施干预后，项目学校学生的知识水平有明显提高，特别是对于蔬菜和洋快餐的认识有大幅度转变。由于西方饮食文化的渗透以及快节奏生活方式的影响，许多家庭已经倾向于饮食的西化而并没有意识到其带来的种种负面影响。我们针对这种情况大力对学生进行营养宣教并指导其行为。另外，项目学校学生在如何控制体重方面认识也有大幅度提高，这无疑给肥胖干预工作打下良好的基础。值得我们注意的是学生们对“食物金字塔”和“每天合适的运动量应该是多少”的了解仍然不够，这说明我们在干预教育中仍有盲点，对学生的干预工作还需要做深做细，否则会事倍功半甚至引起一些负面影响。

在态度方面，项目学校在干预之后有明显提高，所有态度题的正确持有率都达到95%以上，说明项目学校学生在选择健康生活方式，摒弃不良饮食方面有非常积极主观的态度。

在行为方面，项目学校学生们在日常生活的诸多方面已经有了可喜的转变，特别在饮食方式上的转变比较明显；但是在看电视和玩电脑的时间上没有减少，这提示我们电视电脑对学生肥胖的影响深远；同时大部分学生仍然不会选择正确的运动量。

一年来，对照学校学生在知识、态度、行为方面上并没有明显的变化和提高，这也可以从问卷的总分比较中看出来，说明我们的干预工作对学生产生了良好的教育和引导作用。总的来讲，上述结果与该项目在其他两座城市的五所小学所进行的调查结果有比较好的一致性。

值得我们重视的是学生行为方式的形成和转变遵循知→信→行的规律，学生可塑性大，这是肥胖干预的有利条件，但学生又容易受其自身意志力、家庭配合度、环境影响力等多因素作用，其行为要固定下来往往需要好几年的坚持和努力，这也是为什么我们在短短的一年后无法观察到学生肥胖率明显下降的原因。我们在对学生进行肥胖干预时切忌违背客观事实而采取高强度、急功近利式的做法，容易给学生生长发育造成不良影响并引起学生心理逆反，而最终导致干预工作的失败。

综上所述，对学生肥胖的干预和治疗长期以来是医学上的难点，国内外许多学者均在探索各种安全有效的方法，不管是以个体、家庭还是群体为主体进行干预，都需要坚持较长时间，比如 3 年甚至更长才能观察到明显的效果[6,7]，因此，在我们的工作中必须意识到对肥胖的干预控制是一项长期而艰巨的工程，不可懈怠。以发展健康促进学校的方式来进行学生肥胖干预是一种新尝试，其优势是动员政府、学校、社区、家庭、个人广泛的参与和支持，非常值得作为一项可持续发展的工作继续推行。

【参考文献】

[1] 吕书红. 儿童肥胖流行趋势及干预对策探讨 [J]. 中国健康教育，2002，18（8）：526－528.
[2] 李 慧，黄 坚，常小芳，等. 深圳市学龄儿童 BMI 肥胖标准参考值的建立 [J]. 现代预防医学，2001，28（2）：138－140.
[3] 马洪亮，徐岫茹. 金奖之路——来自中国/WHO 健康促进学校项目的报告 [M]. 北京：中国科学技术出版社，2000.10.
[4] 蒋竞雄，夏秀兰，吴光驰，等. 学龄儿童单纯性肥胖症的群体干预研究 [J]. 中国儿童保健杂志，2002，10（6）：364－367.
[5] Smith JC，Sorey WH，Quebedeau D，et al. Use of body mass index to monitor treatment of obese adolescents [J]. J Adolesc Health，1997，20：466－469.
[6] 蒋竞雄，夏秀兰，惠京红. 家庭因素对儿童肥胖治疗的影响 [J]. 中国儿童保健杂志，1999，37（1）：2－7.
[7] 丁宗一，刘玺诚，樊征红等. 儿童期单纯肥胖症的行为治疗 [J]. 中华儿科杂志，1997，35（3）：128－131.

[收稿日期] 2004－02－15 [本文编辑] 周 玮

?1994-2015 China Academic Journal Electronic Publishing House. All rights reserved. http://www.cnki.
