# Supplementary material for: Systematic review and meta-analysis of school-based obesity interventions in mainland China
Source: PLoS One. 2017 Sep 14;12(9):e0184704. doi: 10.1371/journal.pone.0184704 (PMC5598996; doi:10.1371/journal.pone.0184704)
Supplement: S1 Dataset — (ZIP) [file pone.0184704.s007.zip › S1_dataset/76库/48.pdf]

文章编号: 1006- 3110(2002)01- 0078- 02

单纯性肥胖儿童体育锻炼的效果评价

谭 晖,王震维,安爱华,欧阳凤秀,王文英

摘要: 本研究对 76名 7~ 11岁单纯性肥胖儿童进行为期 2年半的中等强度体育锻炼,并评价肥胖儿童体重控制的效果。结果显示: 较长期中等强度体育锻炼可有效控制肥胖儿童体重,干预组 2年后已有 8人(13.79%)体重恢复至正常,而对照组仅有 1人(2.22%)体重恢复正常;结果也显示: 体育锻炼的近期效果更好。提示: 在儿童中开展锻炼控制体重是行之有效的方法,但如何长期坚持下去,还需要校领导、监督老师、家长及儿童本人的配合支持。

关键词: 儿童;单纯性肥胖;体育锻炼;控制体重

中图分类号: R339.31 文献标识码: B

近年来,西方国家儿童单纯性肥胖的发生率为 10%~20%[1],我国也在逐年增加。许多研究证实,儿童期肥胖不仅有损于儿童的形态、机能及心理发育[2,3],更可延续到成人期,导致成人期肥胖,增加了高血压、冠心病、糖尿病、肾病等慢性病的发病率和死亡率[4,5]。本研究采用以体育老师监督的有规律的体育锻炼对肥胖儿童进行控制体重的干预性研究,并对其效果作初步评价。

1 对象与方法

1.1 研究对象 整群抽取上海市卢湾区经济、教学条件相似的两所小学的 1~ 4年级,年龄 7~ 11岁的儿童,根据体检资料筛查出肥胖度≥ 10%的儿童,再由调查者复测,排除因疾病等引起肥胖者,将满足超重及肥胖标准者纳入研究,共筛选出 167例。以其中一所学校的肥胖儿童作为干预组,共 76名,另一所学校 91名作为对照组,两组儿童的年龄、性别分布均无显著性差异。

1.2 肥胖的判断标准 采用 WHO推荐的身高标准体重法评估肥胖程度[8]。以肥胖度 < 10%为正常,≥ 10%为超重,≥ 20%为肥胖。

1.3 测量方法 定期测量两组儿童形态及机能指标,包括身高、体重、胸围、三角肌及肩胛下皮褶厚度、左臂围以及收缩压、舒张压;并计算总皮褶厚度、体重指数(BMI)、体脂率等指标。各指标均按全国学生体质调研统一标准测定,各专项指标由专人负责测量。

1.4 干预方法 对筛选出的 76名干预组肥胖儿童及其家长分别进行肥胖危害及控制知识讲座;并由体育老师监督干预组儿童每天早晨进行半小时运动,内容包括跑步、踢毽子、跳绳等,要求运动后儿童脉博达到 120次/分。干预时间为 2年半。

2 结果与分析

2.1 干预前后两组肥胖儿童的构成分析 表 1对两组中肥胖儿童的构成进行了分析,结果显示: 干预组在干预 1年后,肥胖儿构成由 80.3%降至 58.6%,并有 8名(13.8%)儿童体重恢复至正常范围;随后的一年,这种构成基本无改变。对照组随着时间的推移,肥胖儿的构成基本无变化,2年的时间内仅有 1名(2.2%)儿童体重恢复正常。提示: 体育锻炼对儿童控制肥胖程度,恢复正常体重具有积极的作用。

表 1 不同干预时期两组肥胖儿构成

时间	干预组 n= 76			对照组 n= 76		
	正常(%)	超重(%)	肥胖(%)	正常(%)	超重(%)	肥胖(%)
干预前	—	15(19.7)	61(80.30)	—	18(19.8)	73(80.2)
1年后	8(13.8)	16(27.6)	34(58.6)a	—	9(14.8)	54(85.2)b
2年后	7(12.1)	17(29.3)	34(58.6)	1(2.2)	12(26.7)	32(71.1)c
χ^2		14.921)			6.18	

注: 1) $P < 0.01$ a干预组 1年后毕业 18人 b对照组 1年后毕业 26人,转学 2人 c对照组 2年后毕业 18人

2.2 不同时期形态指标及肥胖指标的变化 以总皮褶厚度、体脂率、肥胖度、BMI 4种指标来反映肥胖程度和体脂含量,对两组中随访满 2年的儿童,比较不同干预时期这 4种指标及形态指标的改变。2年中干预组儿童身高增长值(9.9 cm)和胸围增长值(5.7 cm)均高于对照组(其增长值分别为 5.1 cm - 0.4 cm),尤其是胸围的增长表现得更为明显。这说明体育锻炼对儿童的生长发育有效明显的促进作用。干预组反映肥胖程度和体脂含量的指标如皮脂厚度、体脂率、肥胖度等在干预 1年后均明显降低,但干预 2年后的测定值又有不同程度的回升;对照组各指标没有出现这种起伏变化,随时间推移,均有明显增高。图 1反映干预组儿童在 2.5年的干预期内肥胖程度和体脂含量的变化。在干预的前期,各指标均快速下降,干预半年时达最低点,此后半年趋于稳定;至第 2年却迅速回升并趋于稳步增加。

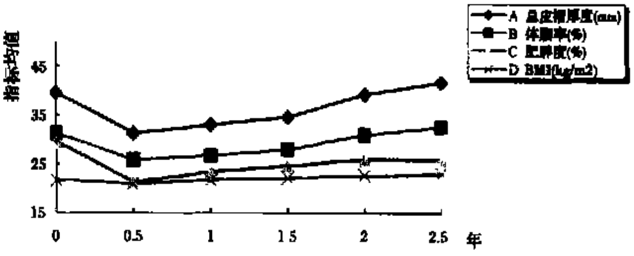

图 1 干预组不同干预时期肥胖指标变化

3 讨论

肥胖的成因复杂,受遗传、环境、文化、经济、生理条件等诸多因素的影响。在过去的二十多年中,随着肥胖发生率的不断

作者单位: 复旦大学公共卫生学院儿童少年卫生学教研室(中国上海 200032)

?1994-2015 China Academic Journal Electronic Publishing House. All rights reserved. http://www.cnki.net

文章编号: 1006- 3110(2002) 01- 0079- 01

【方法介绍】

非操作性误差引起的假性血小板减少

袁 浩

中图分类号: R446. 11 文献标识码: D

临床上有一些假性血小板减少者[1],多数是由于检测血小板计数时操作误差所致,但也有一些非操作误差而产生的假性血小板减少,常见标本中含有 EDTA依赖性抗体和病人本身可能含有冷凝集抗体,也可导致血小板发生明显簇集而显著减少,现报告如下。

1 材料与方法

1. 1 *病例来源* 选择 1997~ 2000年期间病人和门诊病人中由非操作性误差引起的血小板减少,其周围血片中发现有明显血小板簇集现象者,共 72例。

1. 2 *抗凝管制备* (1) 10% EDTA- K_2溶液 2滴置于试管中; (2) 1 mg/ml肝素钠溶液 0. 5 ml置于试管中,将以上两种抗凝管放置在 37℃~ 40℃温箱中烘干备用,每管可抗凝血液 5 ml。

1. 3 *按全国临床检验操作规程要求*[2] 采集病人静脉血 2 ml加入抗凝管内,分别计数病人血标本在两种抗凝剂情况下的血小板总数。

2 结果

将 72例病人血标本加入含 K_2- EDTA抗凝管中,计数血小板数量显著减少且外周血片上有明显簇集者共 45例。但是将此 45例病人血标本加入肝素钠抗凝管中,血小板总数有显著升高,接近或达到正常水平,同时将该管抗凝血涂片未发现有血小板簇集现象,由此可见此 45例病人含有 EDTA依赖性抗体,占总数的 62. 5%。 72例病人中发现含有冷凝集抗体的患者 27例,占总数的 37. 5%。将此 27例病人全血标本加入肝素钠抗凝管内,血小板未见有明显升高,且周围血片中仍然有明显血小板簇集,可见并非由 EDTA引起,为此作者将所有试剂与计数工具均在 37℃水浴中预热 30 min,并在 5 min内计数完毕,其血小板总数显著增加,接近或达到正常水平,同时在计数池内未见到有血小板簇集现象。

作者单位: 湖南省人民医院检验科 (中国湖南 长沙 410002)

3 讨论

血小板在体外明显簇集不多见,但一旦发生即可引起血小板减少。据国外文献报告,当患者仅有血小板减少而无其他任何血液学异常时,则需考虑特异质性或抗血小板抗体性药物反应[3]。

[参考文献]

[1] 丁训杰,林宝爵.实用血液病学 [M].上海: 上海医科大学出版社, 1992. 224.
[2] 叶应妩,王毓三.全国临床检验操作规程 [M].第二版.南京: 南京东南大学出版社, 1997. 265- 267.

(收稿日期: 2001- 10- 15)

增加、肥胖危害的逐渐明朗化,对控制肥胖、减轻体重的方法进行了多种尝试,目前比较常用的有: ① 节制饮食,② 增加运动, ③ 行为- 生活方式的改变,④ 药物治疗,⑤ 综合治疗。但无论哪一种方法都没有起到立竿见影的效果[5]。单纯性儿童肥胖不同于成人肥胖症,其干预方法也不能简单地等同于减肥,干预期间的一切手段必须在不影响儿童正常心身发育的前提下实施。在诸多控制体重的方法中,体育锻炼有益于生长发育,副作用几乎不存在,在儿童群体中也较易实施管理[6,7]。因此,本研究选择了体育锻炼作为干预措施,希望能通过增加有氧运动,促进脂肪代谢,增加能量消耗,而控制儿童体内脂肪沉积。从结果来看,也取得了一定的效果,干预后 1年儿童肥胖程度和体脂含量均显著降低;但中、后期效果不佳。究其原因: 干预初期是在进行知识教育的基础上开展体育锻炼的行为干预,不仅体育老师监督较严,儿童锻炼努力,而且调动了家长的积极性,使其参与了儿童饮食、行为的改变,因此,初期干预效果较理想;随着时间的推移,家长对肥胖相关知识已淡忘,儿童学习任务繁重及锻炼依从性降低,体育老师监督也有所放松等诸多不利因素的影响,使干预效果降低。这也是提示我们在儿童中开展体育锻炼控制体重,要注重取得校领导、班主任老师的支持、注重调动家长的积极性,并对取得进步的儿童进行多种形式的奖励表扬。此外,本研究还发现: 肥胖儿童经过寒暑假期后,肥胖程度均有明显的“回升”现象,因此在寒暑期中,鼓励儿童多参加户外活动,积极开展夏令营等儿童乐于参加,家长和社会易于接受的活动,是十分必要的。

[参考文献]

[1] Jeor ST, Brownell KD, Atkinson RL, et al. Obesity [J]. Workshop Ⅲ. Circulation, 1993, 88(3): 1391- 1396.
[2] 丁宗一,蒋竞雄,许金华,等.肥胖儿童的有氧能力损伤 [J].中华儿科杂志, 1990, 28(6): 341- 343.
[3] 万同斌,李重荣.单纯性肥胖儿童自我意识水平、社会适应能力与行为问题研究 [J].中国心理卫生杂志, 1993, 7(1): 1- 3.
[4] Must A, Jacques PF, Dallal Ge, et al. Long- term morbidity and mortality of overweight adolescents: A Follow- up of the 1922- 35 Harvard Growth Study [J]N Engl J Med, 1992, 327: 1350- 1355.
[5] NIH Technology Assessment Conference Panel. Methods for voluntary weight loss and control [J]. Ann Intern Med, 1993, 119 (7pt2): 764- 770.
[6] Dietz WH. Therapeutic strategies in children obesity [J]. Horm Res, 1993, 39(suppl 3): 86- 90.
[7] 丁宗一.儿童期单纯性肥胖症及对策 [J].中华医学杂志, 1992, 72 (3): 129- 130.

(收稿日期: 2001- 11- 06)

?1994-2015 China Academic Journal Electronic Publishing House. All rights reserved. http://www.cnki.net
