# Supplementary material for: Systematic review and meta-analysis of school-based obesity interventions in mainland China
Source: PLoS One. 2017 Sep 14;12(9):e0184704. doi: 10.1371/journal.pone.0184704 (PMC5598996; doi:10.1371/journal.pone.0184704)
Supplement: S1 Dataset — (ZIP) [file pone.0184704.s007.zip › S1_dataset/76库/49.caj]

·临床研究·

运动与营养疗法干预单纯性肥胖儿童的效果

戴 俊,姜 振,张 兵

盐城师范学院体育系,江苏省盐城市 224002
戴 俊,男,1965年生,江苏省盐城市人,汉族,1991年扬州大学毕业,副教授,主要从事体能教学与研究。

中图分类号:R339.4 文献标识码:A 文章编号:1671-5926(2006)32-0020-03
收稿日期:2005-12-21 修回日期:2006-02-04 (05-50-12-9763/W·LL)

Exercise and nutrition therapy for simple obesity in children

Dai Jun, Jiang Zhen, Zhang Bing
Department of Physical Education, Yancheng Teachers' College, Yancheng 224002, Jiangsu Province, China
Dai Jun, Associate professor, Department of Physical Education, Yancheng Teachers' College, Yancheng 224002, Jiangsu Province, China
Received: 2005-12-21 **Accepted:** 2006-02-04

Abstract

AIM: To observe and compare the effect of interventions with exercise, nutrition and exercise mixed with nutrition on the simple obesity in children.

METHODS: From March to May 2005, a total of 131 children with simple obesity, aged from six to eight years, were selected from grade 1 to 5 of four primary schools of Yancheng. The children with body height and mass surpassed 20% of the standards suggested in WHO were regarded as simple obesity. The children were randomly divided into 4 groups: control group (n=31), exercise group (n=33), nutrition group (n=32) and exercise + nutrition group (n=35). There was no significant difference among the children before the experiment. ①All the children were intervened with 12 weeks of sports, nutrition and exercise + nutrition, respectively. Sports content: exercise density: 50% maximum oxygen consumption and 65% maximum heart rate for each child; exercise frequency: 1 hour daily, 5 days every week. Nutrition control: the weight-lost speed was 0.3-0.5 kg every week. The caloric content proportion for 3 meals: the nutrition group: breakfast was 25%, lunch 50% and supper 25%; the exercise + nutrition group: breakfast was 25%, lunch 45% and supper 30%. Food proportion: fat 25%-30%; protein 20%-25%; sugar in staple (carbohydrate) 50%. The control group was not given any intervention. ②The body mass, vital capacity, electrocardiogram (ECG), B-ultrasound, heart rate, blood pressure, the glucose, total cholesterol (TC), triglyceride (TG), high and low density lipoprotein cholesterol (HDL-C and LDL-C) in serum were detected before and after treatment, respectively.

RESULTS: All 131 children were involved in the result analysis. ①The methods of exercise and exercise + nutrition control reduced the body mass and improved the units of the body. The body mass of the children in the exercise group decreased 3.1kg averagely, and the exercise + nutrition group decreased 2.2 kg averagely. ②The fitness of body (vital capacity, grip strength, sitting trunk flexion, sitting up, standing long jump and step test indexes) was markedly improved by the exercise only or exercise + nutrition control compared with the control group and nutrition group. ③The percentage of B ultrasonic abnormity in exercise group and exercise + nutrition group were decreased compared with before intervention; the percentage of abnormity of the ECG and B ultrasound in the nutrition group were obviously decreased than before intervention (P < 0.01). ④The content of glucose, TG, TC and LDL-C in serum were markedly decreased, while the HDL-C was increased distinctly after exercise.

CONCLUSION: ① Exercise and exercise mixed with nutrition can effectively modulate the body mass and appearance of the obesity children, and the effect of exercise + nutrition control is better than the other. ②The better method to reduce the body mass is to use the integrated exercise prescription. Besides the traditional method to reduce the body mass and improve the cardiopulmonary function, the strength and exercise ability of obesity children should be enhanced to make them form a good habit of exercise.

Dai J, Jiang Z, Zhang B.Exercise and nutrition therapy for simple obesity in children. *Zhongguo Linchuang Kangfu* 2006;10(32):20-2(China)
戴俊,姜振,张兵.运动与营养疗法干预单纯性肥胖儿童的效果[J]. 中国临床康复,2006,10(32):20-2 [www.zglckf.com]

摘要

目的:观察比较单纯运动、单纯营养及运动+营养3种方法干预单纯性肥胖儿童的效果。

方法:选择2005-03/05盐城市4所小学1-5年级单纯性肥胖儿童131名,年龄8~12岁,单纯性肥胖标准以体质量超过WHO推荐的身高标准体质量值20%以上,随机分为4组,对照组31名、运动(干预)组33名、营养(干预)组32名、运动+营养(干预)组35名。实验前各组的基本情况无显著性差异。①各干预组分别进行12周的运动、营养、运动+营养的干预措施。运动处方内容:运动强度,每个儿童最大氧消耗50%或最大心率65%。运动频度,1 h/d,5 d/周。营养控制方案:确定减重速率,0.3~0.5 kg/周。3餐热量分配:营养组早餐25%,中餐50%,晚餐25%;运动+营养组早餐25%,中餐45%,晚餐30%。食物类别比例:脂肪25%~30%;蛋白质20%~25%;主食糖类(碳水化物)50%。对照组不加任何干预控制。②实验前后分别测定体质量、肺活量等及心电图、B超、心率、血压、血清葡萄糖、血清总胆固醇、血清三酰甘油、血清高密度脂蛋白胆固醇、血清低密度脂蛋白胆固醇。

结果:131名肥胖儿童全部进入结果分析。①单纯运动与运动+营养调控能使肥胖儿童体质量减轻,改进身体组成成分,其中运动组体质量下降平均达3.1kg,运动+营养组平均下降2.2 kg。②运动组、运动+营养组儿童身体素质(肺活量、握力、坐位体前屈、仰卧起坐、立定跳远、台阶实验指数)较对照组与营养组有明显提高。③运动组、运动+营养组B超异常百分率比实验前明显减低;营养组实验后心电图和B超异常百分率均比实验前明显减低(P < 0.01)。④运动能有效降低血清中的葡萄糖、三酰甘油、总胆固醇、低密度脂蛋白胆固醇含量,显著增加高密度脂蛋白胆固醇含量。

结论:①运动与运动+营养组在调控肥胖儿童体质量和形态方面具有较好效果,其中运动+营养组效果更好。②肥胖儿童应采用综合运动处方,在传统的减去体质量、增加心肺功能的同时,要考虑到增强肥胖儿童的力量素质和整体活动能力,养成良好的体育锻炼习惯。

主题词:运动疗法;营养;体重;儿童;肥胖症

0 引言

儿童肥胖导致的不良后果直接表现在:智力降低,动作操作能力和运动协调性差,性格孤僻、缺乏自信心,影响青少年正常的性发育。此外,青少年慢性疾病的发病率也显著上升。在国内肥胖儿童的问题已得到了普遍关注,对单纯采用体育锻炼和合理营养减肥的研究均有报道[1]。本文旨在采用不同形式的体育锻炼结合营养控制指导肥胖儿童的减肥,同时采用多种指标对上述干预的效果进行评价。

1 对象和方法

设计:随机对照观察。

单位:盐城师范学院。

对象:选择2005-03/05盐城市一小、盐城市二小、盐城市城区实验小学、盐城市城东小学1~5年级单纯性肥胖儿童131名,男68名,女63名(由于研究对象的年龄均在8~12岁之间,其形态结构、生理功能

上的差异较小,因而未对男女进行分组),单纯性肥胖标准以体质量超过 WHO 推荐的身高标准体质量值 20%以上,随机分为 4 组,对照组 31 名、运动(干预)组 33 名、营养(干预)组 32 名、运动+营养(干预)组 35 名。实验前各组的基本情况无显著性差异。

设计、实施、评估者:设计与评估为第一作者,实验的具体操作与数据收集为第二、三作者,盐城市第一人民医院对实验的过程进行了指导。

方法:

实验法:分组确定后,干预组进行 3 个月(12 周)的运动、营养、运动+营养的干预措施。实验前后分别测定心电图诊断(光电 6511 型心电图仪)、B 超(东芝 240 型超声波仪)、心率、血压、血清葡萄糖、血清总胆固醇、血清三酰甘油、血清高密度脂蛋白胆固醇、血清低密度脂蛋白胆固醇。

运动处方内容:运动强度:每个儿童最大氧消耗 50%或最大心率 65%[2]。运动频度:1 h/d,5 d/周。运动时限:12 周。运动方式:运动平均消耗热量 1 255 J/d。减脂肪预计:1 255 J×5×12/32 217 (J/kg)=2.34 kg。

第 1,2 周:①步行 3~5 min。②广播操和准备活动 5~7 min。③跳绳或踢毽 10 min。④慢跑(游戏性)10~15 min。⑤斜式俯卧撑、立卧撑 10 个左右,做 2 组。⑥整理运动 5 min 左右。第 3,4 周:巩固以上练习,逐步增加要求,重在培养学生的运动兴趣。第 5~8 周:①广播操和准备活动 5~10 min。②俯卧撑、立卧撑 10 个左右,做三四组。③骑自行车、慢跑结合运动 20~25 min。④跳绳或踢毽 10 min。⑤整理运动 5 min 左右。这一阶段运动总时间应保证在 45 min 以上。第 9~12 周:基本内容与要求同上。运动时间保证在 60 min 左右,提高学生的运动能力和运动习惯。

营养控制方案:第一,依据标准体质量原则,基础代谢率指导原则,适应性原则,照应生长量、运动实验消耗与补充的原则。分别制定了具体的营养控制方案[3,4]。确定总的减重速率,0.3~0.5 kg/周(不考虑增长)[5]。确定热值供应分组、分阶段指标值见表 1。第二,明确 1 日 3 餐制热量分配:营养调节组早餐 25%,中餐 50%,晚餐 25%;运动营养组早餐 25%,中餐 45%,晚餐 30%[6]。第三,明确日摄入热量的食物营养 3 类别比例:脂肪 25~30%,< 30%;蛋白质 20~25%,< 25%;主食糖类(碳水化物)50%。另外要求每生每天食用新鲜蔬菜,保证各类维生素、纤维素和矿物质的结合。

表 1 依据四大原则确定热值供应分组、分阶段指标值

组别	第 1 阶段(1,2 周)	第 2 阶段(3~8 周)	第 3 阶段(9~12 周)
营养组	实际体质量×26.4	标准体质量×(28~30)	标准体质量×(26.4~28)
运动+营养组	实际体质量×(28~30)	标准体质量×(30~33)	标准体质量×(28~30)

行为干预措施:对于干预组,在实施干预时,对其进行相应的教育,纠正不良生活行为:减少看电视时间;养成细嚼慢咽的进食习惯,以减少食入量;纠正吃零食的习惯。对照组不加任何干预控制[5]。

主要观察指标:①肥胖儿童干预前后体质量和身体成分、身体素质和机能、心电图、B 超诊断和血压测量异常百分率、血清指标。④不同干预方法对血液指标影响的比较。⑤不同干预方法对身体素质和机能影响的比较。

统计学分析:由第一作者应用 SPSS 10.0 软件完成。计量指标采用配对 t 检验,组间比较采用单因素方差分析,两两比较采用均方差的 t 检验,计数指标换算成百分率进行 χ^2 检验。

2 结果

2.1 参与者数量分析 纳入肥胖儿童 131 名,分为 4 组,无脱落,全部进入结果分析。

2.2 肥胖儿童干预前后各组体质量与健康指标的比较

2.2.1 肥胖儿童干预前后体质量和体成分的比较 通过对实验前后体质量、上臂部皮褶厚度、肩胛下角、腹部皮褶厚度、体脂百分比、脂肪百分比的测定,基本能反映干预因素对体质量和身体成分影响的程度,结果见表 2。

表 2 干预前后肥胖儿童体质量和体成分的比较 ($\bar{x}\pm s$)

指标	对照组(n=31)		运动组(n=33)	
	实验前	实验后	实验前	实验后
体质量(kg)	49.29±6.03	50.14±6.92	49.46±7.86	46.33±7.83^b
上臂部皮褶厚度(mm)	24.76±4.61	25.67±4.65	26.55±6.39	23.90±5.87^a
肩胛下角皮褶厚度(mm)	31.76±6.98	29.81±8.16	32.05±10.37	29.40±8.19
腹部皮褶厚度(mm)	36.81±5.07	38.24±8.86	37.65±9.03	32.53±8.34^b
体脂(%)	37.42±4.60	36.84±4.60	36.53±4.34	34.08±3.81^a
脂肪(%)	0.95±0.04	0.92±0.05	0.94±0.06	0.87±0.05^a
指标	营养组(n=32)		运动+营养组(n=35)	
	实验前	实验后	实验前	实验后
体质量(kg)	47.79±6.01	46.29±9.22	48.28±6.46	46.06±6.20^b
上臂部皮褶厚度(mm)	25.42±6.39^a	23.74±4.78	24.50±3.15	22.17±4.84
肩胛下角皮褶厚度(mm)	31.16±6.11^a	30.32±8.98	31.22±6.49	28.28±9.63
腹部皮褶厚度(mm)	35.68±8.95	34.50±9.18	36.44±5.87	32.72±8.34
体脂(%)	37.96±4.65	35.47±4.79	38.23±4.13	35.12±3.50^a
脂肪(%)	0.95±0.06	0.92±0.06	0.96±0.07	0.90±0.04^a

与实验前比较,$^aP < 0.05$,$^bP < 0.01$

表 2 的结果显示,对照组在实验前后的一段时间内体质量和身体成分变化不明显($P > 0.05$);营养控制组除了在上臂部、肩胛下角皮褶厚度明显下降外,体质量等其他指标无显著变化;运动组除了肩胛下角皮褶厚度下降不具有显著性外,体质量和其他代表肥胖的指标均有显著下降,体质量下降平均达 3.1 kg。运动+营养组则表现出在肩胛下角、腹部的皮褶厚度变化不显著,体质量和其他指标均有显著下降,体质量平均下降 2.2 kg。

2.2.2 肥胖儿童干预前后身体素质和机能的比较 按照国民体质测试方法,对研究对象的肺活量、握力、坐位体前屈、1 min 仰卧起坐次数、立定跳远和台阶指数进行了测量,以便反映儿童肥胖干预因素对儿童身体素质和机能的影响程度,结果见表 3。

表 3 干预前后肥胖儿童身体素质和机能的比较 ($\bar{x}\pm s$)

指标	对照组(n=31)		运动组(n=33)	
	实验前	实验后	实验前	实验后
肺活量 (mL)	1 824.9±456.8	1 775.0±370.7	1 759.8±442.4	1 891.0±411.8[a]
握力 (kg)	16.38±3.22	16.13±3.63	15.71±4.47	19.08±3.86[b]
坐位体前屈 (cm)	3.27±7.58	3.85±7.10	3.75±6.21	6.42±4.72[a]
仰卧起坐(次/min)	18.95±8.56	22.38±8.33[a]	18.90±7.92	25.35±7.26[b]
立定跳远(cm)	109.38±17.69	112.38±13.84	112.75±14.73	125.50±13.47[b]
台阶实验指数	46.81±5.18	49.76±11.03	48.41±11.09	54.82±8.55[a]

指标	营养组(n=32)		运动+营养组(n=35)	
	实验前	实验后	实验前	实验后
肺活量 (mL)	1 759.8±442.4	1 891.0±411.8	1 752.2±462.2	1 859.2±345.4
握力 (kg)	15.71±4.47	19.08±3.86	15.73±2.93	19.67±3.40[b]
坐位体前屈 (cm)	3.75±6.21	6.42±4.72	3.75±6.71	6.36±6.44[a]
仰卧起坐(次/min)	18.90±7.92	25.35±7.26	18.56±10.38	25.33±10.98[b]
立定跳远(cm)	112.75±14.73	125.50±13.47	108.61±24.16	122.78±18.73[b]
台阶实验指数	48.41±11.09	54.82±8.55	46.17±7.44	54.78±9.71[b]

与实验前比较，$^aP < 0.05$，$^bP < 0.01$

表 3 的结果显示，实验前后各指标有所变化，1 min 仰卧起坐次数显著增加，其他变化无显著性；运动组各指标均有显著增加，提示 12 周的专门运动能有效提高身体素质和机能；营养控制组各指标的变化也无显著性($P > 0.05$)；运动与营养组在肺活量增加上不具有显著性，其他指标均有显著增加。

2.2.3 心电图、B 超诊断和血压测量异常百分率比较 心电图异常主要表现为窦性心律不齐，B 超异常主要表现为轻度脂肪肝、脂肪肝和胆囊炎结石。结果显示，在肥胖儿童中存在心电图异常和 B 超异常的比例较高，受试者中均无血压异常。干预前后营养组心电图异常出现明显好转，各组 B 超异常均有显著的好转，主要是一些轻度脂肪肝儿童在干预后有了明显的效果，见表 4。

表 4 心电图、B 超诊断和血压测量异常百分率比较 (%)

指标	对照组(n=31)		运动组(n=33)		营养组(n=32)		运动+营养组(n=35)	
	实验前	实验后	实验前	实验后	实验前	实验后	实验前	实验后
心电图异常	14.54	11.69	15.78	12.8	16.13	10.34[b]	16.86	12.61
B 超异常	38.46	40.91	35.8	14.3[a]	37.14	13.79[b]	36.14	17.86[b]
血压异常	0	0	0	0	0	0	0	0

与实验前比较，$^aP < 0.05$，$^bP < 0.01$

2.2.4 肥胖儿童干预前后儿童血清指标的比较 见表 5。

表 5 干预前后儿童血清指标的比较 ($\bar{x}\pm s$,mmol/L)

指标	对照组(n=31)		运动组(n=33)	
	实验前	实验后	实验前	实验后
葡萄糖	4.23±0.34	4.18±0.46	4.15±0.29	3.92±0.45[a]
总胆固醇	4.45±0.77	3.81±0.75	4.38±0.64	3.72±0.60[b]
三酰甘油	1.64±0.72	1.30±0.78	1.63±0.55	1.14±0.55[b]
高密度脂蛋白胆固醇	1.39±0.33	1.45±0.32	1.44±0.28	1.61±0.27[b]
低密度脂蛋白胆固醇	2.33±0.73	2.14±0.64	2.13±0.40	1.91±0.41[b]

指标	营养组(n=32)		运动+营养组(n=35)	
	实验前	实验后	实验前	实验后
葡萄糖	4.23±0.31	4.03±0.37	4.21±0.28	3.97±0.27[b]
总胆固醇	4.62±1.42	3.87±0.77[a]	4.52±0.78	3.70±0.56[b]
三酰甘油	1.55±0.28	1.08±0.65[b]	1.60±0.44	1.08±0.68[b]
高密度脂蛋白胆固醇	1.44±0.32	1.53±0.44	1.41±0.23	1.61±0.26[b]
低密度脂蛋白胆固醇	2.47±1.12	2.13±1.04[b]	2.18±0.60	1.80±0.43[b]

与实验前比较，$^aP < 0.05$，$^bP < 0.01$

运动能有效降低血清中的葡萄糖、三酰甘油、总胆固醇、低密度脂蛋白含量，显著增加高密度脂蛋白含量；营养控制对减少血清中的葡萄糖浓度、增加高密度脂蛋白含量无显著效果。

2.3 不同干预方法对血液指标影响的比较 见表 6。

表 6 不同干预方法对血液指标影响的比较 ($\bar{x}\pm s$,mmol/L)

指标	运动组(n=33)	营养组(n=32)	运动+营养组(n=35)
葡萄糖	3.92±0.45[a]	4.03±0.37	3.97±0.27[a]
总胆固醇	3.72±0.60[a]	3.87±0.77	3.70±0.56[a]
三酰甘油	1.14±0.55[a]	1.08±0.65	1.08±0.68[a]
高密度脂蛋白胆固醇	1.61±0.27[a]	1.53±0.44	1.61±0.26[a]
低密度脂蛋白胆固醇	1.91±0.41[a]	2.13±1.04	1.80±0.43[a]

与营养组比较，$^aP < 0.05$

结果显示运动能降低血清中葡萄糖、总胆固醇、三酰甘油和低密度脂蛋白的浓度，提高高密度脂蛋白的浓度，营养调控的作用不明显。运动组之间差异无显著性。

2.4 不同干预方法对身体素质和机能影响的比较 见表 7。

表 7 不同干预方法对身体素质和机能影响的比较 ($\bar{x}\pm s$)

指标	运动组	营养组	运动+营养组
肺活量 (mL)	1 891.00±411.80[a]	1 769.2±306.9	1 859.2±345.4[a]
握力 (kg)	19.08±3.86[a]	16.95±3.75	19.67±3.40[a]
坐位体前屈 (cm)	6.42±4.72[a]	3.34±8.03	6.36±6.44[a]
仰卧起坐(次/min)	25.35±7.26[a]	22.68±7.56	25.33±10.98[a]
立定跳远(cm)	125.50±13.47[a]	116.26±16.72	122.78±18.73[a]
台阶实验指数	54.82±8.55[a]	50.4±69.84	54.7±89.71[a]

与营养组比较，$^aP < 0.05$

从表 7 的结果中可见，运动组与营养组相比，在身体素质和机能方面有显著增强，而运动组与运动营养组对身体素质和机能的影响差异无显著性。

3 结论与建议

①干预前后营养组心电图异常出现明显好转，各组 B 超异常均有显著的好转，主要是一些轻度脂肪肝儿童在干预后有了明显的效果。干预措施中运动与营养有机调节效果最明显。②运动+营养能有效降低肥胖儿童血清中的葡萄糖、三酰甘油浓度、总胆固醇含量、低密度脂蛋白含量，显著增加高密度脂蛋白含量；营养控制对减少血清中的葡萄糖浓度、增加高密度脂蛋白含量无显著效果。③肥胖儿童应采用综合运动处方，在传统的减去体质量、增加心肺功能的同时，要考虑到增强肥胖儿童的力量素质和整体的活动能力，使其在运动中建立自信心，养成良好的体育锻炼习惯。④建议教育部门和家长要重视肥胖儿童中出现的心电图和 B 超异常，多方协调，通过运动和营养干预减少异常的进一步深化。⑤卫生部门、教育部门和体育部门应组织、动员家长积极参与，共同实施肥胖儿童的干预，从而提高肥胖青少年儿童的健康体质。

4 参考文献

1 叶超群，康玉华，杨俊卿.肥胖、耐力运动对单纯性肥胖少年儿童免疫功能的影响[J].中国运动医学杂志,2000,19(1):45-8

2 彭向峰，康玉华，杨俊卿.单纯性肥胖少儿血内皮素、血脂的改变及耐力运动对其影响的研究[J].中国运动医学杂志,2000,19(2):167-70,173

3 杜熙茹.健身运动对肥胖儿童健康的影响[J].广州体育学院学报, 2003,23(1):37-9

4 蔡斌.影响儿童肥胖运动疗法效果的相关因素分析[J].中国临床康复, 2003,7(21):3021

5 刘幸华.肥胖儿童的健康教育与行为干预[J].浙江临床医学,2004,6(6):529

6 丁宗一.中国儿童单纯肥胖症现状、趋势及防治对策[J].天津体育学院学报, 1999,14(1):12-4
