# Supplementary material for: Systematic review and meta-analysis of school-based obesity interventions in mainland China
Source: PLoS One. 2017 Sep 14;12(9):e0184704. doi: 10.1371/journal.pone.0184704 (PMC5598996; doi:10.1371/journal.pone.0184704)
Supplement: S1 Dataset — (ZIP) [file pone.0184704.s007.zip › S1_dataset/76库/50.pdf]

【健康教育】

# 北京市部分学校成年期疾病早期干预效果评估

段佳丽[1], 孙洪文[2], 吕鸥[3], 宋辉[3], 郭向辉[3], 张海涛[2]

【摘要】 目的 探索在学校开展成年期疾病早期预防的综合防治措施,为制定可行的实施方案提供依据。方法 2004－2006年在北京市大兴区和朝阳区各选1所中学和1所小学,开展为期2 a的成年期疾病早期预防综合干预试点,并在2个区分别设立对照学校。结果 干预后,干预组学生慢病预防相关知识知晓率高于对照组,每天吃早餐、喝牛奶、经常进行体育锻炼等行为发生率高于对照组,差异均有统计学意义;形态指标和生化指标除小学生骨密度值干预组高于对照组($t=9.08$, $P<0.05$)外,其他指标2组间差异均无统计学意义。结论 成年期疾病早期预防是一件艰巨的任务,需要社会各界、学校、家长的共同参与与长期努力。

【关键词】 肥胖症;高血压;高血糖症;健康教育;干预性研究;学生保健服务

【中图分类号】 G479 R179 【文献标识码】 A 【文章编号】 1000-9817(2008)05-0404-03

Impact Evaluation on Early Prevention on Adulthood Diseases in Some Schools in Beijing/DUAN Jia-li*, SUN Hong-wen, LV Ou, et al. *Beijing Center for Disease Prevention and Control, Beijing (100013), China

【Abstract】 Objective To explore the comprehensive measures in schools to prevent adulthood diseases in early stage, and to provide bases for the practicable plans. Methods A two-year early prevention against adulthood diseases was conducted in a middle school and a primary school in the districts of Daxing and Chaoyang of Beijing in 2004－2006. The control schools were also set in the two districts respectively. Results After the intervention, the response rate of knowledge of chronic diseases was higher in intervention group than the control. More students had breakfast, milk and participated sports activities regularly than their counterpart. The value of bone mineral density in intervention group was higher than the control group ($t=9.08$, $P<0.05$) among morphological and biochemical indices. There were no statistical significant difference of other indices between the two groups. Conclusion As the prevention for adulthood diseases in early stage is a hard task, it needs the cooperative and long-term efforts from parents, schools and the whole society.

【Key words】 Obesity; Hypertension; Hyperglycemia; Health education; Intervention studies; Student health services

随着经济社会的快速发展和膳食营养结构的转型,我国居民肥胖患病率迅速增高,高血压、糖尿病、冠心病等慢性非传染性疾病越来越成为威胁人类健康、影响人们生活质量的疾病。以生活方式为重要影响因素的慢性病在儿童青少年中发病率逐年上升,相关的不良生活方式和行为问题也是目前影响儿童健康和慢性病发生的重要因素[1-3]。儿童时期是建立良好行为习惯的最佳时期[4]。从小开展成年期慢性病的预防性综合干预,使儿童养成健康的生活方式,将会起到事半功倍的效果。为探索在学校开展成年期疾病早期预防的综合防治措施,制定可行的实施方案,2004－2006年笔者在北京市大兴区和朝阳区开展了成年期疾病早期预防综合干预试点研究。

## 1 对象与方法

1.1 对象 在北京市朝阳区、大兴区各选择1所中学初一和高一年级学生,1所小学二~四年级的学生为干预组,基线调查中学生637名,小学生572名,终期调查中学生615名,小学生553名;同时在2个区各选择与干预组学校地理位置、经济背景、规模相近的中、小学校各1所的同年级学生为对照组,基线调查中、小学生676名和615名,终期调查中小学生640名和593名,进行2 a的追踪干预研究。

1.2 方法 干预组建立并实施包括开展针对学生、家长和教师在内的健康教育,建立了每0.5 a 1次的健康监测,由学校和家长参与的营养和体育锻炼指导以及常见心理问题指导等成年期疾病早期预防综合防治方案。为期2 a,比较干预前后2组学生相关疾病患病率和行为改变情况,评估干预效果。评价指标包括体格检查指标、血液生化指标、知识知晓率、观念和行为发生率。

1.2.1 体格测量 各项指标的测量均由统一培训的专业人员完成。采用统一测量用具,测量前校准。采用2002年全国居民营养与健康状况调查中规定的仪器和方法测量身高、体重等指标。血压测量采用汞立式血压计、儿童专用袖带,读数以mm Hg为单位,取2次平均值。学龄儿童采用CM 100超声骨密度测量仪测量右足跟骨骨密度。

肥胖和超重采用中国肥胖工作组制定的《中国儿童青少年超重肥胖BMI分类标准》[5]进行评价。以高于1987年北京市儿童青少年第95百分位血压值为血压偏高。

1.2.2 血生化检查 采用罗氏公司提供的快速血糖测定仪测量空腹血糖。血糖偏高指空腹血糖≥6.1 mmol/L。

1.2.3 问卷调查 由中小学生及其家长分别填写学生KAP问卷和家长KAP问卷,调查知识知晓率、观念和行为发生率。

## 2 结果

2.1 干预前后超重率和肥胖率变化情况 干预前后,干预组与对照组比较,儿童超重率和肥胖率差异均无统计学意义($P$值均$>0.05$)。见表1。

2.2 干预前后血压偏高检出率变化情况 干预前后,干预组与对照组比较,儿童血压偏高率差异均无统计学意义($P$值均$>0.05$)。见表2。

2.3 干预前后血糖偏高检出率的变化情况 干预前后,干预

【作者简介】 段佳丽(1971－ ),女,北京市人,大学本科,学士学位,主管医师,主要从事学校卫生和学生常见病防治工作。

【作者单位】 1 北京市疾病预防控制中心,北京 100013;
2 北京市大兴区疾病预防控制中心;
3 北京市朝阳区疾病预防控制中心。

?1994-2015 China Academic Journal Electronic Publishing House. All rights reserved. http://www.cnki.net

组与对照组比较,儿童血糖偏高率差异均无统计学意义(P值均＞0.05)。见表3。

2.4 干预前后儿童骨密度变化情况 干预前后,干预组小学生骨密度增长值明显高于对照组小学生。见表4。

表1 干预组与对照组学生干预前后超重率和肥胖率比较

| 学段 | 组别 | 基线 | | | | 干预终期 | | | |
|---|---|---|---|---|---|---|---|---|---|
| | | 调查人数 | 超重率/% | 肥胖率/% | $\chi^2$值 | 调查人数 | 超重率/% | 肥胖率/% | $\chi^2$值 |
| 小学 | 干预组 | 570 | 12.63 | 15.79 | 1.90 | 553 | 14.10 | 13.02 | 1.94 |
| | 对照组 | 615 | 12.68 | 13.01 | | 593 | 12.65 | 10.96 | |
| 中学 | 干预组 | 637 | 12.24 | 6.59 | 2.10 | 615 | 6.83 | 9.92 | 2.83 |
| | 对照组 | 676 | 10.35 | 8.14 | | 640 | 7.66 | 7.34 | |

表2 干预组与对照组学生干预前后血压偏高检出率比较

| 学段 | 组别 | 基线 | | | | 干预终期 | | | |
|---|---|---|---|---|---|---|---|---|---|
| | | 调查人数 | 检出人数 | 检出率/% | $\chi^2$值 | 调查人数 | 检出人数 | 检出率/% | $\chi^2$值 |
| 小学 | 干预组 | 572 | 53 | 9.27 | 1.43 | 553 | 57 | 10.31 | 0.04 |
| | 对照组 | 615 | 70 | 11.38 | | 593 | 59 | 9.95 | |
| 中学 | 干预组 | 637 | 41 | 6.44 | 0.54 | 615 | 47 | 7.64 | 0.94 |
| | 对照组 | 676 | 37 | 5.47 | | 640 | 40 | 6.25 | |

表3 干预组与对照组学生干预前后血糖偏高检出率比较

| 学段 | 组别 | 基线 | | | | 干预终期 | | | |
|---|---|---|---|---|---|---|---|---|---|
| | | 调查人数 | 检出人数 | 检出率/% | $\chi^2$值 | 调查人数 | 检出人数 | 检出率/% | $\chi^2$值 |
| 小学 | 干预组 | 572 | 1 | 0.17 | 1.07 | 544 | 4 | 0.74 | 0.28 |
| | 对照组 | 615 | 0 | 0 | | 582 | 6 | 1.03 | |
| 中学 | 干预组 | 637 | 3 | 0.47 | 1.13 | 615 | 8 | 1.30 | 0.11 |
| | 对照组 | 676 | 1 | 0.15 | | 640 | 7 | 1.09 | |

表4 干预组与对照组学生干预前后骨密度变化情况/$(m\cdot s^{-1})$

| 学段 | 组别 | 基线 | | | 干预终期 | | | 骨强度增加值 | 值 |
|---|---|---|---|---|---|---|---|---|---|
| | | 检测人数 | 骨强度均值 | 值 | 检测人数 | 骨强度均值 | 值 | | |
| 小学 | 干预组 | 328 | 1 555.25 | 3.46** | 302 | 1 578.03 | 9.08** | 22.78 | 5.05** |
| | 对照组 | 316 | 1 548.70 | | 295 | 1 564.02 | | 15.32 | |
| 中学 | 干预组 | 412 | 1 548.14 | 1.28 | 401 | 1 566.67 | 1.06 | 18.53 | 0.36 |
| | 对照组 | 351 | 1 551.08 | | 299 | 1 569.23 | | 18.15 | |

注:干预组与对照组比较,** $P<0.01$。

2.5 干预前后学生健康行为发生率比较 见表5。干预后,干预组小学生每周参加有效运动多于4 d的比例明显高于对照组,且有更多的小学生通过"锻炼"来达到控制体重的目的。干预组中学生每天吃早餐、喝牛奶的比例明显提高,并且高于对照组,差异有统计学意义(P值均＜0.05);每周参加有效运动多于4 d的比例明显高于对照组。

值得注意的是,由于学业的压力,中、小学生参与体育锻炼的总体比例比基线时明显下降;另外,有0.94%～4.11%的学生曾采用过"超过24 h不吃东西、私自服用减肥药、呕吐或腹泻"等不正确的方法进行减肥和控制体重。

表5 干预组中小学生健康相关行为发生率干预前后比较/%

| 相关行为 | 小学生 | | 中学生 | |
|---|---|---|---|---|
| | 干预前 | 干预后 | 干预前 | 干预后 |
| 饮食行为 | | | | |
| 每天吃早餐 | 86.08 | 76.17 | 40.64 | 82.97** |
| 每天喝牛奶 | 49.10 | 48.78 | 43.11 | 50.47 |
| 吃水果≥3次/周 | 78.89 | 61.74 | 80.18 | 70.94 |
| 每天吃蔬菜 | 87.14 | 65.97 | 87.14 | 73.28 |
| 经常喝碳酸饮料 | 51.30 | 60.94 | 58.92 | 52.95 |
| 运动与静坐 | | | | |
| 有效运动≥4 d/周 | 74.70 | 54.94 | 55.25 | 47.58 |
| 视屏作业≥2 h/d | 52.84 | 69.06** | 50.01 | 72.74** |

注:** $P<0.01$。

2.6 干预后中小学生相关知识知晓情况 经过2 a的干预,干预组学生在肥胖、体育锻炼、膳食搭配等方面相关知识的知晓率明显高于对照组,干预组对于减肥、吃早餐、喝牛奶、吃肉等方面的认识正确率亦高于对照组(P值均＜0.05)。见表6。

表6 干预后中小学生相关态度、知识正确率干预组与对照组比较/%

| 相关知识和态度 | 小学生 | | 中学生 | |
|---|---|---|---|---|
| | 干预组 | 对照组 | 干预组 | 对照组 |
| 态度 | | | | |
| 生长期不能盲目减肥 | 83.42 | 77.79* | 81.18 | 79.62 |
| 早餐必须吃 | 82.23 | 77.06* | 85.43 | 80.21* |
| 喝牛奶要适量 | 77.41 | 73.13 | 85.25 | 79.01* |
| 吃肉要适量 | 79.23 | 75.62 | 82.84 | 74.85** |
| 知识 | | | | |
| 肥胖的危害 | 25.95 | 20.80* | 23.36 | 18.00* |
| 青少年减肥禁忌 | 63.86 | 60.15 | 75.31 | 66.67** |
| 户外活动时间 | 47.85 | 37.48** | 53.70 | 47.56* |
| 体育锻炼适宜强度 | 64.94 | 62.35 | 77.04 | 72.28* |
| 含钙多的食物 | 70.79 | 63.89* | 68.79 | 64.68 |
| 膳食宝塔 | 71.84 | 57.71* | 75.92 | 61.63** |
| 三餐配比 | 77.74 | 70.88* | 85.08 | 80.49 |
| 健康生活方式 | 88.07 | 88.28 | 93.00 | 91.18 |

注:* $P<0.05$,** $P<0.01$。

2.7 干预后2组学生家长相关知识、行为比较 从总体上看,家长对"生长期不能盲目减肥、吃早餐、喝牛奶、吃肉"等方面的

?1994-2015 China Academic Journal Electronic Publishing House. All rights reserved. http://www.cnki.net

认识正确率较高,均超过80%。干预组家长对于营养、健康生活方式、体育活动、肥胖相关知识的知晓率普遍高于对照组,其中小学生家长2组间的差异有统计学意义($P$值均<0.05)。见表7。

表7 干预后学生家长相关知识和行为发生率
干预组和对照组比较 /%

| KAP | 小学生 | | 中学生 | |
|---|---|---|---|---|
| | 干预组 | 对照组 | 干预组 | 对照组 |
| 态度 | | | | |
| 生长期不能盲目减肥 | 93.76 | 89.48* | 93.88 | 89.52* |
| 早餐必须吃 | 96.25 | 91.52** | 85.59 | 86.26 |
| 喝牛奶要适量 | 87.62 | 85.30 | 80.96 | 80.98 |
| 吃肉要适量 | 90.07 | 84.63** | 82.95 | 84.82 |
| 知识 | | | | |
| 肥胖的危害 | 27.65 | 17.52** | 30.62 | 27.93 |
| 青少年减肥禁忌 | 82.10 | 71.13** | 77.88 | 76.04 |
| 户外活动时间 | 61.67 | 43.22** | 46.33 | 45.22 |
| 体育锻炼适宜强度 | 80.68 | 79.45 | 80.39 | 76.45 |
| 含钙多的食物 | 83.62 | 65.53** | 72.48 | 75.59 |
| 膳食宝塔 | 80.66 | 63.85** | 73.02 | 62.69** |
| 三餐配比 | 88.87 | 79.66** | 87.63 | 82.47* |
| 健康生活方式 | 94.69 | 92.99 | 89.90 | 90.49 |
| 行为 | | | | |
| 在孩子面前吸烟 | 38.19 | 39.08 | 42.45 | 39.22 |
| 在孩子面前饮酒 | 42.88 | 44.57 | 42.79 | 44.31 |

注:* $P<0.05$, ** $P<0.01$。

## 3 讨论

成年期疾病的早期预防是一项艰巨的任务,需要社会各界共同参与,长期努力。儿童青少年营养与健康问题不是卫生单一部门所能解决的事,必须采取多部门合作、社会广泛参与的策略。本研究集合了政府部门、卫生保健机构、医院、学校、社区等多方面的力量,建立起成年期疾病早期预防的网络与平台,发挥各自的优势,从多角度开展健康宣教工作,使得本项目在短期内基本显现出初步效果。

有研究表明,生化指标的改变需要长期积累[1,6-8]。日本营养午餐的效果是在学生营养计划实施50 a后显现出了显著成果和发展后劲;北京市学生常见病防治通过10 a努力才对贫血、蛔虫、沙眼等部分常见病显现出显著效果。成年期疾病的预防控制有身体素质的因素,也有行为生活方式因素,更需要长期努力。研究结果显示,干预组学生虽然在患病率以及相关指标等方面改善不明显,但在行为改善和知识知晓率方面提高较为显著。

儿童成长和行为养成离不开社会环境的参与、学校养成教育以及家长的引导和监督。因此,做好成年期疾病早期预防工作,必须由社会各界共同参与、有政策保证等为前提。

应用健康促进的理念对家长开展健康教育和指导,有助于促进儿童膳食结构改善和体育锻炼习惯的养成。父母的生活方式、饮食习惯及对肥胖危害的认识态度是决定其行为的重要因素。儿童青少年(尤其是低年龄段)生活自理能力和自制力较差,其饮食、运动行为主要受家庭的影响;而家长的认知和行为必将影响到儿童日常生活中的饮食、运动行为的态度,影响学龄前儿童习惯的形成和儿童肥胖的发生。通过定期培训和指导,干预组家长相关健康知识知晓率明显高于对照组,这也是干预组学生健康知识和行为提高的基础。

另外,干预组儿童骨密度增加的绝对值均大于对照组。可见体育锻炼对儿童骨密度的增长起到了积极的促进作用,干预项目的实施有利于促进儿童体格改善。

成人慢性病的早期预防应从小抓起,起步越早越好。习惯是从小养成的,良好的生活习惯可使人受益终生。应从加强对家长的健康教育入手,从小培养儿童健康的饮食和运动习惯[6-8]。干预结果显示,小学生健康知识知晓率和健康行为发生率高于中学生,骨密度等形态指标改变优于中学生。可见干预越早,效果越好。

## 4 参考文献


[1] 刘泽军. 2002年北京市居民营养与健康状况调查报告. 北京:中国科学技术出版社, 2006: 106-131.

[2] 陈荣华, 李晓南. 儿童营养现状和改善前景. 中国食物与营养, 1997(3): 35-36.

[3] 吕书红, 儿童肥胖流行趋势既干预对策探讨. 中国健康教育, 2002, 18(8): 526-528.

[4] 季成叶. 儿童少年卫生学. 5版. 北京:人民卫生出版社, 2004: 45-47, 102-104, 108-113.

[5] 季成叶. 中国学生超重肥胖BMI筛查标准的应用. 中国学校卫生, 2004, 25(1): 125-128.

[6] 段佳丽, 刘峥, 任振勇, 等. 2002年北京市居民生长发育现况分析. 中国校医, 2005, 19(4): 334-340.

[7] 王伯英, 王绍丽, 执笔. 近半个世纪来北京市儿童青少年生长发育动向研究: 全国学生体质与健康研究, 北京:人民卫生出版社, 1987: 788-818.

[8] 刘泽军, 王绍丽, 严亦柔, 等. 北京市儿童青少年生长发育的长期变化. 1995年北京市学生体质与健康调研报告论文汇编, 北京, 1997: 1-11.


(收稿日期: 2007-06-05)

(上接第403页)

## 4 参考文献


[1] HAEKSAA E. 在1999年世界无烟日座谈会上的讲话. 中国吸烟与健康通讯, 1999(3): 2.

[2] CROFTON J, SIMPSON D. Tobacco: A global threat. Macmillan Education, 2002: 49.

[3] 杨功焕, 马杰民, 刘娜, 等. 中国人群2002年吸烟和被动吸烟的现状调查. 中华流行病学杂志, 2005, 26(2): 77-83.

[4] 田本淳, 钱玲, 张巍, 等. 我国6城市中学生烟草与健康方面的知信行状况分析. 中国行为医学科学, 2006, 15(4): 363-366.

[5] 文孝忠, 陈维清, 卢次勇, 等. 初中生有关吸烟与健康知识和需求调查. 中国公共卫生, 2006, 22(5): 638-640.

[6] UNGER JB, YAN L, SHAKIB S, et al. Peer influences and access to cigarettes as correlates of adolescent smoking: A cross-cultural comparison of Wuhan, China and California. Prev Med, 2002, 34(4): 476-484.

[7] 柯雪琴, 范雪瑾. 青少年控烟健康教育两种方法的效果比较. 中华预防医学杂志, 2002, 36(2): 144.

[8] AUDREY S, HOLLIDAY J, CAMPBELL R. It's good to talk: Adolescent perspectives of an informal, peer-led intervention to reduce smoking. Soc Sci Med, 2006, 63: 320-334.


(收稿日期: 2008-03-19)

?1994-2015 China Academic Journal Electronic Publishing House. All rights reserved. http://www.cnki.net
