# Supplementary material for: Systematic review and meta-analysis of school-based obesity interventions in mainland China
Source: PLoS One. 2017 Sep 14;12(9):e0184704. doi: 10.1371/journal.pone.0184704 (PMC5598996; doi:10.1371/journal.pone.0184704)
Supplement: S1 Dataset — (ZIP) [file pone.0184704.s007.zip › S1_dataset/76库/51.pdf]

文章编号：1007-2705(2006)05-0008-03 中图分类号：R 193 文献标识码：A

【论著】

厦门市健康促进学校控制儿童肥胖项目效果评价

陈健

摘要：[目的] 通过以肥胖控制为切入点发展健康促进学校模式，逐步摸索出适合国情的预防控制儿童肥胖的途径和方法。[方法] 在项目学校进行肥胖干预，干预前后对学生进行问卷和肥胖率调查。[结果] 干预 1 年后，项目学校学生的知识、态度和行为水平均有显著提高，肥胖发生率从 22.1%下降至 14.5%（$P<0.05$），学生体质指数(BMI)正常率从基线调查时的 60.1%上升到终期的 65.9%；而对照学校学生肥胖率未见下降。[结论] 创建活动和肥胖干预工作对学生有良好的教育和引导作用，但需政府、学校、社区及家庭的支持。以肥胖控制为切入点发展健康促进学校的模式值得推广。

关键词：健康促进学校；肥胖；疾病控制

Effectiveness of Project on Taking Obesity Control as Entry Point of Health Promoting School in Xiamen, Fujian

CHEN Jian (*Xiamen Center for Disease Prevention and Control, Fujian 350001, China*)

Abstract: [**Objective**] To develop an effective way by means of obesity control to establish health promoting school (HPS). [**Methods**] In project school, obesity rate and health behavior of students was investigated and analyzed before and after intervention. [**Results**] The pass rates of knowledge, attitude and behavior of students were increased in project school. The obesity rate decreased from 22.1% to 14.5% ($P<0.05$); The normal rate of BMI was increased from 60.1% to 65.9% ($P<0.05$), but the obesity rate was not changed in contrast school ($P>0.05$). [**Conclusion**] The obesity intervention and HPS establishing have nice education and induction effect for student, and government, school, community and family support are necessary. The model of developing HPS by means of taking obesity control as entry point is worth to be recommended.

Key Words: Health Promotion School (HPS); Obesity; Disease Control

肥胖是目前发达国家和发展中国家带有普遍性的社会问题和健康问题。厦门市参与了中国/WHO 以肥胖控制为切入点发展健康促进学校的项目，希望能够通过该项活动，在项目学校树立“健康第一”的理念，建立预防控制学生常见病、多发病的框架机制。该项目 2004 年 3 月正式启动，2005 年 3 月评估，现将厦门市的调查结果报告如下：

1 对象与方法

1.1 对象 厦门市外国语附属小学在本市学生肥胖率最高，被定为项目干预学校；以厦门大同小学为对照，与项目学校在规模、学生肥胖率、生源和运动场地等情况相仿。项目校和对照校三、四年级的学生为调查对象，两校分别有 408 和 317 名学生(基线调查 407 和 333 名)，男女构成比相近，分别为 51.7%、48.3% 和 52.6%、47.4%。除学生外，家长及全体教师和行政领导均接受基线和终期问卷调查。

1.2 方法

1.2.1 体格检查 分别在基线和终期测量体重、身高，根据中国学龄儿童青少年超重、肥胖筛查 BMI 分类标准[1] 进行营养状况判定。

1.2.2 问卷调查 用国家疾病预防控制中心(CDC)健康教育所统一印制的“膳食与运动知识态度行为调查问卷”，有学生、家长和学校教职工 3 种问卷。内容有一般情况、与肥胖控制相关的知识(13 道题)、态度(5 道题)和行为(15 道题)4 部分。相应合格标准：答对 10 道及以上知识题、4 道及以上态度题、9 道及以上行为题。学生问卷由班主任组织学生当堂完成，逐份检查后编号；家长问卷专门开家长会填写；教职工问卷由学校统一安排完成。

1.3 控制肥胖的干预措施 ①开设学生健康教育课，举办家长培训班，讲授肥胖危害及控制知识。同时

基金项目：中国/WHO 以肥胖控制为切入点发展健康促进学校。
第一作者简介：陈健(1969—)，男，主管医师，多年从事学校卫生工作。
作者单位：厦门市疾病预防控制中心，福建 361004。

?1994-2015 China Academic Journal Electronic Publishing House. All rights reserved. http://www.cnki.net

分发有关健康教育材料。②使用学生健康管理手册，建立超重和肥胖学生个人档案，组织健身俱乐部。对超重、肥胖学生每月测 1 次身高、体重，观察体重变化，及时鼓励和指导；对其他学生也观察身高、体重变化趋势，及时纳入管理的目标学生中。

1 4 质量控制与数据处理分析 监测调查前由国家 CDC 健教所统一培训调查员，收回调查表时由调查员对是否有漏项、误答等问题进行核查。用 EpiData 2 0 软件包建数据库，SPSS 10 0 软件包统计分析。

2 结果

2 1 营养状况和肥胖率 项目学校在终期调查时学生肥胖率为 14 5%，低于基线调查时的肥胖率 22. 1% ($P<0$ 05)，而对照学校肥胖率没有下降 ($P>0$ 05)。项目学校学生体质指数正常率有上升趋势，从基线调查的 60. 1%上升到终期调查的 65. 9%，见表 1。

表 1 项目学校和对照学校学生体质状况比较

Table 1 The pupil' s level of BMI in project school and contrast school

学校	正常		超重		肥胖	
	基线	终期	基线	终期	基线	终期
项目学校	60 1	65 9	17 9	19 6	22 1	14 5 *
对照学校	70 5	70 2	15 1	16 9	14 5	12 9

* 项目学校基线与终期调查比较，$P<0$ 05。

2 2 问卷调查合格率比较 项目学校的学生知识合格率从 2 2%上升到 94. 8% ($P<0$ 01)，态度、行为合格率也分别从基线的 52. 3%和 6. 9%上升到终期的 100 0%和 39 7%，差异有显著性 ($P<0$ 01)。对照学校的学生知识合格率从 0. 3%上升到 10. 1%，前后 2 次调查态度、行为合格率类似 ($P>0$ 05)。学生问卷调查合格人数的增长幅度项目学校远远大于对照学校，见表 2。

表 2 两校学生问卷知识知晓率、态度形成率和行为形成率比较(%)

Table 2 The pupil' s level of knowledge, attitude and behavior in project school and contrast school

学生分组	人数	知识合格	态度合格	行为合格
项目学校基线调查	407	9(2 2)	213(52 3)	28(6. 9)
终期调查	408	387(94 8) *	408(100 0) *	162(39 7) *
对照学校基线调查	333	1(0 3)	152(45 7)	20(6. 0)
终期调查	317	32(10 1)	189(59 6)	37(11. 7)

* 项目学校基线与终期调查比较，$P<0$ 01。

干预后项目学校家长的知识合格率由基线的 1. 2%上升到 56 5% ($P<0.$ 01)，与其学生知识合格率 94 8 %相比增长幅度较慢。态度和行为合格率前后类似 ($P>0.$ 05)。对照学校前后调查知识、态度、行为合格率均类似 ($P>0.$ 05)，见表 3。

表 3 两校家长问卷知识知晓率、态度形成率和行为形成率比较(%)

Table 3 The parent' s level of knowledge, attitude and behavior in project school and contrast school

家长分组	人数	知识合格	态度合格	行为合格
项目学校基线调查	408	5 (1 2)	366 (89 7)	108 (26 5)
终期调查	407	230 (56 5) *	400 (98 3)	153 (37 6)
对照学校基线调查	333	0 (0)	289 (86 8)	69 (20 7)
终期调查	317	5(1 6)	295 (93 1)	57 (17 9)

* 项目学校基线与终期调查比较，$P<0$ 01。

干预后项目学校教师的知识、态度及行为合格率分别由原来的 4 0%、14 7%和 2 7%均上升到终期的 100 0% ($P<0.$ 01)。对照校教师前后调查知识、态度和行为合格率类似 ($P>0$ 05)，见表 4。

表 4 两校教师问卷知识知晓率、态度形成率及行为形成率比较(%)

Table 4 The teacher' s level of knowledge attitude and behavior in project school and contrast school

教师分组	人数	知识合格	态度合格	行为合格
项目学校基线调查	75	3(4 0)	11(14 7)	2(2 7)
终期调查	72	72(100 0) *	72(100 0) *	52(72 2) *
对照学校基线调查	57	5(8 8)	10(17 5)	4(7 0)
终期调查	52	5(9 6)	18(34 6)	4(7 7)

* 项目学校基线与终期调查比较，$P<0$ 01。

3 讨论

3 1 肥胖干预取得显效 终期调查项目学校学生肥胖率从 1 年前的 22 1%下降至 14 5%，比蒋竟雄等人[2] 3 年中学生肥胖率从 16 9%下降至 12 1%的效果更显著。同时学生体质指数正常率有上升趋势。说明肥胖控制干预措施在降低学生肥胖率的过程中发挥了关键作用，也有效控制了超重学生向肥胖发展，部分超重学生体质指数正常。

3 2 健康饮食知识水平提高 实施干预后，项目学校学生的相关知识水平明显提高，知晓率高达 94 9%，特别是对于蔬菜和洋快餐的认识大幅度转变。随着生活节奏加快，许多家庭倾向于饮食的西化而未意识到其负面影响，我们对此进行营养宣教指导其行为。干预后项目学校学生的态度形成率达到 100%，行为形成率也从干预前的 6 9%提高到干预后的 39 7%，但提高幅度低于知识与态度，因学生容易受其自身意志力、家庭配合度、环境影响力等多因素影响。本项目主要以学校为主导，对家长的影响力相对不足，其态度与行为未取得明显改善，在一定程度上也会影响学生行为的转变。学生在肥胖控制过程中处被动地

?1994-2015 China Academic Journal Electronic Publishing House. All rights reserved. http://www.cnki.net

位,须有教师和家长的引导才能收到良好效果。Lindsay 等[3] 认为父母在儿童肥胖干预中起着至关重要的作用。我们也认为在干预过程中应加强对家长的健康知识培训,把实施范围推广到家庭中,只有学校、家长相互配合,干预效果才能事半功倍。

对学生肥胖的干预和治疗长期以来是医学上的难点,国内外许多学者均在探索各种安全有效的方法[4,5]。以发展健康促进学校的方式来进行学生肥胖管理是一种新尝试,其优势是动员政府、学校、社区、家庭、个人广泛参与和支持,可作为一项可持续发展的工作继续推行。

参考文献:

[1] 中国肥胖问题工作组. 中国学龄儿童青少年超重、肥胖筛查体重指数值分类标准[J]. 中华流行病学杂志, 2004, 25(2): 97-102

[2] 蒋竞雄, 夏秀兰, 吴光弛, 等. 学龄儿童单纯性肥胖症的群体干预研究[J]. 中国儿童保健杂志, 2002, 10(6): 364-367.

[3] Lindsay AC, Sussner KM, Kim J, et al. The role of parents in preventing childhood obesity [J]. Future Child, 2006, 16(1): 169-186

[4] Broderick CR, Winter GJ, Allan RM. Sport for special groups[J]. Med J Aust, 2006, 184(6): 297-302

[5] Story M, Kaphingst KM, French S.. The role of schools in obesity prevention [J]. Future Child, 2006, 16(1): 109-142

收稿日期: 2006-06-02

文章编号 1007-2705(2006)05-0010-01 中图分类号: R 155.3 文献标识码: B

【突发事件调查】

一起苦瓠子中毒的调查报告

王国民

关键词: 食物中毒; 苦瓠子; 食品卫生

2005年10月18日,惠安县张坂镇玉田村发生进食自家种的苦瓠子6人急性中毒事件,调查情况报告如下:

1 流行病学调查

2005年10月18日18:20,玉田村村民骆某一家8人共进晚餐,进食20 min后陆续有6人出现呕吐、腹泻和口干等症状。当天晚餐食物为米饭、炖鸭汤、炒苦瓠子(自养自种),配料花生油、盐、味精和酱油等感官性状均正常,且几天来从未间断食用。共餐时,2个未食苦瓠子小孩未发病,进食炒苦瓠子6人均发病。发病前,所有患者身体健康,米饭、鸭汤、厨房炊具未见异常,否认发病前3天有食用不洁不鲜食物史,故可疑食物为炒苦瓠子。

2 临床诊断和治疗

中毒6人(男4人,女2人),年龄23~66岁,平均34岁,潜伏期20 min至3 h,平均55 min,病状类似,主要为呕吐、腹泻、腹痛、头昏、腹胀和乏力,经当地卫生所采用止吐、止痛、导泻和补液等对症治疗,24 h内全部痊愈。

3 动物试验

取苦瓠子样品8条,瓜型瘦长,直径5~15 cm,长20~50 cm,表面光滑呈浅绿色,肉质白嫩。分别切小片用嘴舔,5条有苦味,3条味稍甜。取味苦和味甜的瓠子各1 000 g,煮汤分别喂2组形体相似的小猪,结果进食苦味瓠子的小猪50 min后出现呕吐、腹泻;而进食甜味瓠子的小猪观察24 h未见异常。

4 讨论

本次中毒都有共同吃苦瓠子的过程,进食在3 h内相继出现类似病状,未进食者均无恙,并经动物实验确认是由苦瓠子引起的食物中毒。

瓠子俗称蒲瓜、葫芦瓜,学名 Lagenaria Leucantua Kushg,属葫芦科植物。正常瓠子味甜,但是有的瓠子味苦有毒,误食可中毒。苦瓠子中毒我国医史上早有记载,《金匮要略》载有黍穰能解苦瓠毒,"黍穰煮汁,数服之,解"。《本草纲木》载有瓠有甜有苦二种,"苦瓠气味苦、寒,有毒","浙人食瓠瓜,多吐泻,唯与香茅同食可免"[1],它反映祖国医学对苦瓠子中毒早有预防和治疗对策。近年来,关于苦瓠子中毒湖南省郴州卫生防疫站等单位在上世纪80年代就作过研究,他们从苦瓠子提取出毒素苦葫芦素D与苦葫芦素I,并作了动物实验,小白鼠对苦瓠子 LD_{50} 为(0.249±0.21) g/kg·BW,对提纯结晶体 LD_{50} 为(0.464±0.069) g/kg·BW[2]。

苦瓠子中毒的预防,最根本措施是选好种子,杜绝有毒种子混入种植,对于市售的瓠子是否有毒,从表面上难于辨别,因此最佳方法是煮切前先切一点舔,有苦味即有毒不可食用,煮熟的瓠子如发现有苦味,应连同锅里的其它食物都弃掉。为防止牲畜中毒,废弃的瓠子不要乱丢,更不能用于喂牲畜。对中毒患者目前尚无特殊疗法。只能采取对症治疗。

参考文献:

[1] 宋卫平. 百毒解方[M]. 南宁: 广西民族出版社, 1989.12

[2] 湖南省郴州市卫生防疫站, 中国人民解放军广州部队军事医学研究所. 苦瓠子中毒调查报告[J]. 中华预防医学杂志, 1983, 17(1): 30

收稿日期: 2006-02-28; 修回日期: 2006-08-14

作者简介: 王国民(1965—),男,主管医师,从事流行病学调查研究工作。

作者单位: 惠安县疾病预防控制中心, 福建 362100。

?1994-2015 China Academic Journal Electronic Publishing House. All rights reserved. http://www.cnki.net
