# Supplementary material for: Systematic review and meta-analysis of school-based obesity interventions in mainland China
Source: PLoS One. 2017 Sep 14;12(9):e0184704. doi: 10.1371/journal.pone.0184704 (PMC5598996; doi:10.1371/journal.pone.0184704)
Supplement: S1 Dataset — (ZIP) [file pone.0184704.s007.zip › S1_dataset/76库/52.pdf]

文章编号:1000-8020(2012)05-0860-01 ·调查报告·

山东省莱阳市小学生肥胖群体干预调查分析

刘剑超 金梅[1] 于美娥
烟台市莱阳中心医院,烟台 265200

关键词:儿童肥胖 营养干预 调查分析
中图分类号:R151.41 R589.2 文献标识码:A

儿童肥胖不但给肥胖儿童带来明显的身体和心理损害,而且也给家庭及社会带来沉重负担[1]。作者于2010年9月-2011年6月对莱阳市某小学1~5年级的学生进行群体性干预,随机将学生对象分为干预组和对照组,并对干预效果进行评价分析。

1 对象和方法

1.1 调查对象

选择莱阳某小学1~5年级的全体学生作为研究对象,按照随机和自愿的原则将5个级部20个班级的1640名学生分为干预组10个班级和对照组10个班级,对照组和干预组在同一学校,同一个活动场地,体育课设置,伙食供应等方面基本一致或相似。2010年9月到2011年6月对研究学校1~5年级的学生进行跟踪观察的结果,观察对象为1640名学生,干预组826名,对照组814名。

1.2 调查方法

1.2.1 调查内容(1)家长填写学生在家时间的问卷调查:学生吃饭速度、吃快餐(次/周)、喝甜饮料(次/天)、油炸食品(次/周)、看电视及玩电脑(小时/天)和晚餐后户外散步(次/周)及家长对儿童肥胖相关知识态度。(2)体格测量:身高和体重。由经过培训的校医采用标准测量方法进行测量,体重精确至0.1kg,身高精确至0.1cm。采用WHO身高体质指数BMI作为肥胖的判断标准:正常体重为18.5~22.9,超重为23~24.9,肥胖为大于25,受试者中未发现病理性肥胖患儿。

1.2.2 干预方案对学校老师和家长进行健康教育培训,使他们了解基本的健康知识一协助管理超重和肥胖儿童。对学生利用学校的健康教育课,用互动感和互助的方式对学生进行"科学饮食,合理控制体重"的健康教育,发放《关注儿童肥胖》健康教育手册。超重肥胖学生在干预期间记录《健康饮食管理日志》,每月由营养专业人员根据日志的记录进行一对一的指导,在健康教育手册中详细列出每个学生的标准食物摄入量供学生和家长参考。运动训练方案由体育老师在学校协助超重肥胖学生完成,每天训练30分钟。对照组除常规健康教育和体育课外无额外干预措施,仅完成相应的体格检查。

干预方案自2010年9月到2011年6月实施,干预组所有学生均为干预对象,进行相关知识讲座,采用以学校为基础的学校群体干预模式。

1.3 资料处理

全部数据输入计算机采用SPSS处理软件处理,计数资料的对比分析采用χ^2检验和危险度分析,计量资料采用t检验。

2 结果

2.1 干预前后两组儿童肥胖发病率的变化

干预前基线调查干预组和对照组肥胖发病率分别为15.6%和15.7%,两组间肥胖和超重发生率均无明显差异,经干预后干预组发病率降至12.1%,比干预前明显降低($P<0.01$),而对照组发病率上升为24.7%明显高于基线调查结果($P<0.01$)。

2.2 干预后两组儿童肥胖变化状况

干预组原非肥胖儿童中有3.5%变为肥胖。而对照组原非肥胖的儿童中有9%变为肥胖。干预后干预组非肥胖儿童成为肥胖的危险性明显小于对照组($P<0.01$),对照组肥胖儿童维持肥胖的可能性明显大于干预组($P<0.01$)。

2.3 干预后儿童饮食和运动行为的变化

从问卷调查结果看,家长的相关知识和行为,孩子在家静坐的时间都朝着有利方面发展,表现为干预组儿童进食速度快的比例明显降低,进食油炸食品,甜食,快餐及甜饮料的比例较干预前有明显的降低,而且晚饭后去户外活动的比

1山东省潍坊医学院附属医院营养科

?1994-2015 China Academic Journal Electronic Publishing House. All rights reserved. http://www.cnki.net

例明显增高。

3 讨论

学龄儿童是肥胖的高发人群,在学校实施肥胖干预方案十分必要,学校对学生的集体管理也使干预方案的实施易于取得成效,在本研究中,纵向观察结果显示干预组肥胖发生率从15.6%下降到12.1%,而对照组肥胖发病率从15.7%上升到24.7%。干预前两组儿童发病率基本相同,而干预后干预儿童发病率较对照组发病率低近两倍,由此可见,我们采用群体干预方案对降低儿童发病率的效果是有效的。

学校是儿童早期建立健康生活方式的良好场所,也为儿童和家长进行营养知识教育提供了便利条件。研究结果表明在学校进行群体干预队改变学生的饮食习惯和增加运动能达到良好的效果。

收稿日期:2011-11-11

文章编号:1000-8020(2012)05-0867-01 ·调查报告·

240例住院病人营养干预疗效观察

程振倩 金梅[1]
山东省千佛山医院,济南 250014

关键词:营养治疗 饮食结构 心血管病 脂代谢
中图分类号:R459.3 文献标识码:A

近10年来,在随着人民生活水平的提高,心血管的发病率呈上升型趋势,饮食结构与心血管病的发生有着密切的关系。不适当的饮食结构,摄入过多的脂肪、碳水化合物,引起机体脂代谢的紊乱,导致心血管病的发生[1,2]。作者于2010年2月-12月对本院心内科240例住院病人的饮食进行干预并做出院后一个月的追踪,监测饮食治疗前后总胆固醇TG、甘油三脂TC、低密度脂蛋白LDL-C、高密度脂蛋白HDL-C的变化。

1 对象和方法

1.1 对象

2010年2月—12月在本院心内科住院的240例心血管病患者,其中男性160例,女性80例,年龄28~82岁,平均年龄64岁。体质指数(BMI)以WHO1998年建议为标准,低于18.5者为消瘦,在18.5~25之间者为正常,高于25者为超重。BMI正常者为84例,超重者为156例。

1.2 方法

1.2.1 饮食习惯 采用问卷调查,有吸烟史的患者140人;有口味偏咸、喜食肥肉、暴饮暴食等不良饮食习惯者214人。

1.2.2 饮食治疗方法 热能根据患者体重情况供给,三大营养素热能比例分别按 碳水化合物55%~65%、蛋白质10%~15%、脂肪20%~30%计算配制食谱[1],且均在医院用餐,并进行营养咨询和宣教。出院1个月内继续随访病人进行饮食指导,对患者饮食治疗后的TG,TC,LDL-C,HDL-C进行监测。

1.3 统计学处理

对数据进行t检验统计学分析处理,计量数据以$\bar{x} \pm s$表示,$P<0.05$差异有显著性。

2 结果

2.1 食物选择和饮食习惯的改变情况

患者主食选用大米、燕麦、玉米、荞麦等含膳食纤维多的食物;蛋白质主要选择鱼、去皮禽肉和豆类;限制胆固醇的摄入,每日低于300mg类选择植物油和橄榄油;每日通过足够蔬菜补充维生素和矿物质,特别是要摄入深绿叶菜和红黄色蔬菜;限制油炸和糕点甜食;限盐每日低于6克。

2.2 治疗前后血生化指标变化情况

由表1可以看出饮食治疗后TG、TC、LDL-C、HDL-C均较治疗前明显下降,差异有显著性,$P<0.05$;HDL—C较治疗前升高,差异有显著性,$P<0.05$。

作者简介:程振倩,女,硕士,副主任医师
1 山东省潍坊医学院附属医院营养科

?1994-2015 China Academic Journal Electronic Publishing House. All rights reserved. http://www.cnki.net
