# Supplementary material for: Systematic review and meta-analysis of school-based obesity interventions in mainland China
Source: PLoS One. 2017 Sep 14;12(9):e0184704. doi: 10.1371/journal.pone.0184704 (PMC5598996; doi:10.1371/journal.pone.0184704)
Supplement: S1 Dataset — (ZIP) [file pone.0184704.s007.zip › S1_dataset/76库/53.pdf]

北京市小学生肥胖干预效果分析

石建辉，刘秀荣，田向阳，李玉青

【中图分类号】R151. 1 【文献标识码】A 【文章编号】1002－9982（2004）09－0782－04

【摘要】 目的 探讨可持续性控制儿童青少年肥胖的有效策略及方法。方法 在东城、海淀和顺义区每区选择干预校和对照校各一所，于2002年4月至2004年3月针对干预校三、四年级学生开展一系列的干预活动，同时设立自身对照和外部对照。结果 干预前后干预校的肥胖率分别为14.19%和15.93%，超重率分别为14.73%和16.33%。干预校的知识合格率由干预前的13.66%上升到干预后33.73%，态度合格率由83.40%上升到86.61%，行为合格率由61.18%上升到74.30%。结论 干预工作对改善儿童青少年肥胖控制相关的知识、态度、行为产生一定效果，但肥胖控制效果不理想。

【关键词】 小学生；肥胖；干预；效果

An Analysis of Intervention on Obese Students in Primary Schools in Beijing *SHI Jian-hui, LIU Xiu-rong, TIAN Xiang-yang, LI Yu-qing. Institute for Health Education, Beijing Center for Disease Control and Prevention, Beijing* 100013, China

【Abstract】 **Objective** To find the effective strategies and methods of controlling students' obesity. **Methods** Separately select one primary school as intervention group and another one as control group for each district in dongcheng, haidian and shunyi district, Beijing. Series of intervention activities were applied for 3～4 grade students in intervention group. **Results** In intervention group the obesity rate is 14.19%, 15.93% respectively before and after implementation of intervention activity, overweight rate is 14.73% and 16.33% respectively. The proportion of those who correctly answered the questions on obesity control knowledge, attitude, behavior respectively increased from 13.66%, 83.40%, 61.18% in baseline research to 33.73%, 86.61%, 74.30% after intervention. **Conclusion** School-based intervention activities worked effectively on students' knowledge, attitude and behavior change relating to obesity control, however with insignificant effect on obesity control.

【Key words】 Pupils; Obesity; Intervention; Effect

肥胖问题作为全球性的公共卫生问题，引起社会各界的关注，尤其是儿童青少年的肥胖问题更为突出。为探讨适合北京市特点的可持续控制儿童青少年肥胖的有效策略及方法，北京市疾病预防控制中心健康教育所于2002年4月至2004年3月在北京市部分小学中开展了肥胖干预效果研究工作，结果如下。

对象与方法

1. 对象 北京市东城、海淀、顺义区每区小学三四年级学生1502对。其中对照校755对，干预校747对。对照校男性占49.27%，女性50.73%，干预校男性占54.08%，女性45.92%；对照校年龄为9.11±0.71岁，干预校为9.15±0.73岁；对照校三年级学生占55.23%，干预校三年级学生占53.82%；干预校肥胖率、超重率分别为14.19%和14.73%，对照校肥胖率、超重率分别为11.79%和13.77%。

2. 方法 在北京市东城、海淀、顺义区分别选择一所小学作为干预校，同时每区选择一所在地域、规模大小、教学、经济等各方面基本相同的学校作为对照校；以三、四年级学生作为研究对象开展了为期两年的研究工作。为保证数据的准确和可靠性，设立

【作者单位】北京市疾病预防控制中心健康教育所，北京 100013
【作者简介】石建辉（1970－），女，河北保定人，大学本科，主管医师，主要从事健康教育与健康促进工作。

?1994-2015 China Academic Journal Electronic Publishing House. All rights reserved. http://www.cnki.

了外部对照和自身对照。外部对照校与干预校在年龄、性别、年级分布和超重/肥胖率上无显著性差异，均衡性较好，具有一定的可比性。此次只对其中 1540 对自身对照数据进行分析，剔除含有逻辑性错误的记录，获得有效数据 1502 对。

分析 2002 年 4 月基线调查数据发现超重和肥胖学生中所存在的最主要的行为问题是吃零食、吃洋快餐、缺少运动。针对以上主要问题，制定了具体干预对策。

（1）干预策略与方法。以控制肥胖增长趋势、提高学生健康水平为主要策略，根据在校学生和各校的特点，在干预校中开展如下干预活动：①成立项目领导小组，建立/健全组织机构；②出台与肥胖控制相关的健康政策和措施，如健康体检制度，常见病预防制度，肥胖、营养不良预防措施，增加体育活动课，开设健康教育课等；③教师讲课过程中结合各学科特点穿插与肥胖控制相关的知识；④举办讲座或培训，讲授肥胖危害和肥胖控制等相关知识。充分利用现有资源如广播、板报等宣传肥胖危害和防治方面的知识。发放宣传材料或小册子；⑤填写《学生个人管理手册》，及时了解肥胖和超重学生的能量摄入和消耗情况，要求肥胖和超重学生每月连续记录一周的食品摄入和运动情况，并且每周自测体重一次。由教师针对学生的记录情况提出个性化指导方案，逐步改善饮食结构，增加运动量；⑥改善物质环境，如改善体育设施和扩大运动场所等；⑦开展其他特色性工作，如鼓励并组织超重和肥胖学生参加集体活动，增加体育锻炼时间，并适当给予奖励；不歧视超重和肥胖学生等；在校内用餐的学生由教师监督进食量，在校外由家长进行监督；鼓励学生少吃零食和洋快餐等。

（2）监测与评价。使用中国疾病预防控制中心统一印制的《学生膳食与运动知识态度行为调查问卷》进行问卷调查。问卷主要包括一般情况、与肥胖控制相关的知识（13 道题）、态度（5 道题）和行为（15 道题）四部分。将知识、态度、行为状况分别分成合格和良好 2 个等级，其中，正确回答 8 道及以上知识题、4 道及以上态度题、9 道及以上行为题的分别为知识合格、态度合格和行为合格；正确回答 10 道及以上知识题、5 道态度题、12 道及以上行为题的分别为知识良好、态度良好和行为良好。问卷中的身高体重部分由教师统一填写。

（3）超重和肥胖的判定标准。根据中国学龄儿童青少年超重、肥胖筛查 BMI 分类标准[1]对被调查学生的营养状况进行判定。将干预前后学生营养状况转变情况分成 3 组，即营养状况不变组、营养状况转好组（指肥胖转为超重或正常；超重转为正常）、营养状况转差组（指正常体重转为超重或肥胖，或超重转为肥胖）。

3. 质量控制与数据处理分析　监测调查前由北京市疾病预防控制中心健康教育所对调查员进行统一培训，调查完毕收表前由调查员对调查表是否有漏项、误答等问题进行核查。

用 EPIDATA 2.0 建立数据库并录入数据，SPSS10.0 进行统计分析，计量资料比较采用 t 检验，计数资料比较采用 R×C 表的 χ^2 检验。

结　　果

1. 干预前后干预校、对照校学生身高、体重变化情况　干预后无论是干预校还是对照校学生身高、体重均有显著增长。干预前后干预校与对照校间无显著差异（见表 1）。

2. 干预前后干预校、对照校学生肥胖与超重率变化情况　干预前后干预校与对照校间的肥胖和超重率无显著性差异；干预校干预前后营养状况无显著性差异，对照校干预后超重率显著升高，体重正常率显著降低（见表 2）。

?1994-2015 China Academic Journal Electronic Publishing House. All rights reserved. http://www.cnki.

表 1 干预前后干预校、对照校学生身高、体重变化情况

指标	对照校（n=755）		干预校（n=747）	
	干预前	干预后	干预前	干预后
身高（cm）	137.54±6.56	150.38±7.53**	137.61±7.14	151.02±7.75**
体重（kg）	33.31±7.45	44.18±9.83**	34.16±8.30	44.62±11.20**

注：** $P<0.01$

表 2 干预前后干预校、对照校学生肥胖与超重率

营养状况	对照校（n=755）		干预校（n=747）	
	2002 年	2004 年	2002 年	2004 年
肥胖	11.79	12.45	14.19	15.93
超重	13.77	18.68**	14.73	16.33
正常	74.44	68.87*	71.08	67.74

注：干预前后比较：* $P<0.01$；** $P<0.01$

3. 干预前后干预校、对照校学生与肥胖控制相关的知识、态度、行为状况　干预校干预前后知识合格及良好、态度良好、行为合格及良好率显著升高。对照校知识合格及良好、行为合格率显著升高。与对照校相比，干预前干预校的知识合格、态度合格及良好率明显高于对照校，干预后干预校的知识合格、态度合格及良好、行为合格及良好率明显高于对照校（见表 3）。

4. 干预前后干预校、对照校学生营养状况转变情况　对照校的超重者中分别有 24.04%、25.96%转为正常体重和肥胖，肥胖者中分别有 14.61%、22.47%转为正常和超重；干预校的超重者中分别有 34.55%、21.82%转为正常或肥胖，肥胖者中分别有 4.72%、16.04%转为正常或超重。对照校中分别有 12.28%、1.96%的正常体重者转为超重和肥胖，干预校中分别有 10.73%、2.07%的正常体重者转为超重和肥胖。肥胖转变率干预校、对照校间存在显著性差异，干预校肥胖转正常率显著低于对照校（见表 4）。

表 3 干预前后干预校、对照校学生与肥胖控制相关的知识、态度、行为状况

知信行状况	对照校（n=755）		干预校（n=747）	
	2002 年	2004 年	2002 年	2004 年
知识合格	9.40	26.35**	13.66☆	33.73*☆☆
知识良好	0.66	3.44**	1.34	4.28**
态度合格	78.28	76.69	83.40☆	86.61☆☆
态度良好	54.57	57.48	59.97☆	71.49**☆☆
行为合格	61.46	68.21**	61.18	74.30**☆☆
行为良好	10.07	12.58	10.04	21.02**☆☆

注：干预前后比较：* $P<0.05$，** $P<0.01$；干预校与对照校比较：☆ $P<0.05$，☆☆ $P<0.01$

表 4 干预前后干预校、对照校学生营养状况转变情况

干预后体重水平	对照校（n=755）				干预校（n=747）			
	正常	超重	肥胖	合计	正常	超重	肥胖	合计
正常	85.77	24.04	14.61	74.44	87.19	34.55	4.72*	67.74
超重	12.28	50.00	22.47	18.68	10.73	43.63	16.04	16.33
肥胖	1.96	25.96	62.92	12.45	2.07	21.82	79.24*	15.93

注：干预校与对照校比较：* $P<0.05$

5. 干预前后营养状况转变的影响因素　干预活动后不同营养程度转变组的知识合格率均显著升高，营养程度不变组行为合格率显著升高。干预前后不同营养程度转变组间

?1994-2015 China Academic Journal Electronic Publishing House. All rights reserved.　http://www.cnki.

知识、态度、行为状况无显著差异。此外，通过分析还发现，男性营养状况转差率(16.36%)显著高于女性(9.92%)(见表5)。

表 5　干预前后不同营养状况转变组肥胖相关知识、态度、行为变化情况

营养程度变化	知识合格		态度合格		行为合格	
	2002 年	2004 年	2002 年	2004 年	2002 年	2004 年
营养状况不变	11.81	30.04**	79.58	81.52	61.27	71.48**
营养状况转差	10.55	30.15**	81.91	81.41	63.82	71.86
营养状况转好	10.17	29.66**	91.53	83.05	57.63	67.80

注：** 干预前后 $P<0.01$

讨　论

学校作为儿童青少年获得健康知识和养成正确行为的重要场所，对提高儿童的健康水平起着至关重要的作用。为及时了解肥胖相关知识、态度、行为状况，探索适合北京市小学校持续控制肥胖的有效策略和方法，北京市疾病预防控制中心健康教育所开展了此次研究工作，旨在利用学校的各种条件为学生创造一个健康的氛围和环境，提高学生的健康知识水平，培养健康的生活方式和行为，以达到控制肥胖增长趋势的目的，并切实提高学生的健康水平。

本次研究结果显示，干预校学生知识、态度和行为的正确持有率均有显著提高，并高于对照校；干预前后干预校学生超重率无显著变化，而对照校超重率显著升高，说明通过一系列的干预活动，在改善学生的知识、态度、行为方面已经取得了一定的效果。

由于儿童正处于生长发育的重要阶段，儿童肥胖的控制必须以不影响其正常的生长发育为前提[2]，而从本次研究结果看，干预校学生身高平均增长 13.41 厘米，体重平均增长 10.46 公斤，与对照校相比无显著性差异，说明干预活动对儿童的正常生长发育未造成不良影响。

另一方面，干预活动后干预校的知识良好率(4.28%)和行为良好率(21.02%)较低，肥胖率无明显改善，同时有 12.80%的正常体重者转为超重或肥胖，说明干预效果尚不理想。

研究结果还显示，干预校和对照校分别有 34.55%和 24.04%的超重者转为体重正常，说明该年龄段超重的可逆转性较好，应该作为肥胖干预的最佳阶段。

肥胖作为一种与生活方式密切相关的营养性疾病，不良的饮食行为和运动行为将通过多种方式影响儿童的能量摄入和能量消耗[3]，对营养状况转变的影响因素作进一步分析发现，各营养状况转变组的知识均有明显改善，而组间无显著性差异，说明知识水平的提高只是行为及营养状况改善的前提条件之一；另外，男生的营养状况转差率显著高于女生，与 7~12 岁城市男生肥胖检出率上升最快的报道[4]相符，提示我们今后的干预工作应该以超重人群为重点，并推广应用到以男生为主的全体学生。

此次干预工作在学生的知识、态度、行为改善方面取得一定的效果，而肥胖控制效果不理想。今后的干预工作应以超重人群为重点人群，辐射到全体学生。

【参考文献】

[1] 中国肥胖问题工作组. 中国学龄儿童青少年超重、肥胖筛查体重指数值分类标准 [J]. 中华流行病学杂志, 2004, 25(2): 97-102.

[2] 马洪亮, 周虹茹, 刘枫, 等. 创健康促进学校的实践与思考 [J]. 中国健康教育, 2000, 16(6): 668-670.

[3] 钱玲, 田本淳. 超重和肥胖学生膳食与运动的知·信·行分析 [J]. 中国学校卫生, 2004, 25(1): 32-33.

[4] 杨贵仁. 2000 年全国学生体质健康状况调研结果 [J]. 中国学校卫生, 2002, 23(1): 3-4.

[收稿日期] 2004-07-28　　[本文编辑] 王晓春

?1994-2015 China Academic Journal Electronic Publishing House. All rights reserved. http://www.cnki.
