# Supplementary material for: Systematic review and meta-analysis of school-based obesity interventions in mainland China
Source: PLoS One. 2017 Sep 14;12(9):e0184704. doi: 10.1371/journal.pone.0184704 (PMC5598996; doi:10.1371/journal.pone.0184704)
Supplement: S1 Dataset — (ZIP) [file pone.0184704.s007.zip › S1_dataset/76库/54.pdf]

文章编号: 1008-6579(2010)03-0206-04 【科研论著】

浦东新区部分小学生营养干预效果研究

郑晶泉[1], 邹淑蓉[2], 杜尉英[1], 王静[1], 陶晔璇[3]

(1 上海市浦东新区疾病预防控制中心健康促进部, 上海 200136; 2 上海市疾病预防控制中心, 上海 200336; 3 上海儿童医学中心临床营养中心, 上海 200127)

中图分类号: R179 文献标识码: A

摘 要: 【目的】 评价营养干预对浦东新区小学生的影响效果, 为探讨改善小学生营养状况的方法提供依据。【方法】 抽取浦东新区城区和乡镇地区小学各两所小学生 1 493 人, 按所在的地域分别分为干预组和对照组, 对干预组开展为期 1 年的营养干预, 并对干预效果进行评估。 【结果】 营养干预后, 城乡地区干预组小学生营养知识知晓率明显提高($P<0.05$); 营养态度有所提高; 营养行为较干预前提高, 城区干预组吃早餐和锻炼人数比例高于对照组; 乡镇干预组睡前不吃零食和锻炼人数比例高于对照组, 以甜饮料解渴和偏食人数比例低于对照组, 以上差异有统计学意义($P<0.05$)。城乡地区干预组小学生营养不良得到改善; 城区干预组超重和肥胖得到控制; 乡镇地区干预组超重和肥胖上升趋势较对照组有所减缓。 【结论】 营养干预能提高小学生营养知识知晓率, 改善营养行为, 控制学生超重及肥胖人数比例。

关键词: 营养干预; 知识; 态度; 行为; 营养状况; 小学生

Investigation on nutrition intervention effects of pupils in Pudong New Area. *ZHENG Jing-quan*[1], *ZOU Shu-rong*[2], *DU Wei-ying*[1], *WANG Jing*[1], *TAO Ye-xuan*[3]. (1 ***Pudong New Area Center for Disease Control and Prevention, Shanghai*** 200136, ***China***; 2 ***Shanghai Municipal Center for Disease Control and Prevention, Shanghai*** 200336, ***China***; 3 ***Shanghai Children's Medical Center, Shanghai*** 200127, ***China***)

Abstract: 【**Objective**】 To evaluate the nutrition intervention effects on pupils of elementary school in Pudong New Area to provide scientific evidence for nutrition status promotion. 【**Methods**】 About 1 493 pupils selected from 4 elementary schools in urban and suburban parts of Pudong New Area were divided into intervention group and control group. Nutrition interventions were conducted on intervention group for about a year. Nutrition status, knowledge, attitude and practices of both groups were surveyed before and after intervention to evaluate intervention effects. 【**Results**】 After nutrition intervention, the nutrition knowledge, attitude and practice of pupils from intervention group in both urban and suburban area were increased. The percentage of pupils from intervention group in urban area having breakfast and exercising were higher than control group($P<0.05$). The percentage of pupils of intervention group in suburban area eating nothing before sleeping and exercising were higher than control group($P<0.05$), while drinking beverage and food partialities were lower than control group($P<0.05$). After nutrition intervention, undernutrition of intervention groups in both urban and suburban area were declined. Overweight and obesity of intervention group in urban area were under control. The increasing speed of overweight and obesity of intervention group in suburban area slowed down compared with the control group. 【**Conclusion**】 Nutrition intervention could increase nutrition knowledge, enhance nutrition behavior and control overweight and obesity among pupils.

Key words: nutrition intervention; knowledge; attitude; practice; nutrition status; pupil

中国正处于营养快速转换时期, 儿童的营养状况有了一定的改善, 但是由于学生及家长营养知识的缺乏, 不能合理饮食, 儿童营养问题仍然存在[1-2]。浦东新区小学生 2004 年营养不良率为 15.38%, 肥胖率为 12.86%, 表现为营养不良和过剩并存[3]。为改善这一状况, 对浦东新区部分小学生的营养知识, 态度, 行为 (knowledge, attitude, practice, KAP) 状况进行基线调查, 然后开展为期 1 年的有针对性的营养干预活动, 为探索适合浦东新区特点的改善小学生营养状况的策略及方法提供依据。

1 对象和方法

1.1 对象 采用分层整群抽样原则, 将浦东新区体格营养状况监测点学校分为乡镇地区及城区学校, 再在其中各随机抽两所小学, 按所在的地域分别分为干预组学校和对照组学校。在抽中的学校 1～4 年级各随机抽两个班的学生作为研究对象。

1.2 内容及方法

1.2.1 KAP 调查 运用根据《中国居民膳食指南》

基金项目: 上海市浦东新区社会发展局科研项目资助(PW2005A-17)
作者简介: 郑晶泉(1973-), 女, 贵州人, 副主任医师, 硕士学位, 主要从事营养卫生工作。

?1994-2014 China Academic Journal Electronic Publishing House. All rights reserved. http://www.cnki.net

自行设计的 KAP 调查问卷，对研究对象进行基线和终期调查。

1.2.2　营养状况评价　营养不良、超重和肥胖的评价采用《1985 年全国 7 ~22 岁城乡学生身高标准体重值》。

1.2.3　质量控制　调查前对调查员统一培训，调查过程中质控员按 4% ~6 %的比例抽查，核实调查结果是否可靠。

1.3　干预措施　动员：取得学校领导重视，形成以卫生老师为中心，班主任配合，疾病预防控制中心和儿童医学中心技术指导的营养干预网络；宣传：卫生老师每周进行营养知识宣传广播；知识讲座：组织肥胖儿童家长及学生开展讲座，利用家长学校开展营养知识讲座，共 10 次；营养咨询活动：组织有营养问题的儿童的家长参加营养咨询活动共 4 次；发放宣传品：发放有针对性的营养宣传资料；管理肥胖超重学生：制定减肥计划，发放记步器，填写自我膳食及活动状况记录册，按月进行体重测量，宣教的同时，对行为给予指导。

1.4　统计学方法　采用 SPSS10.0 软件建立数据库，对指标变量进行频数描述、χ^2 检验等统计分析。

2　结　果

2.1　基本情况　共调查了 1 493 名小学生，男生 710 人，女生 783 人，最小年龄为 6 岁，最大年龄为 11 岁，其中城区干预组 317 人，对照组 306 人；乡镇地区干预组 427 人，对照组 443 人；城乡地区干预组及对照组学生在年龄、性别构成方面都差异没有统计学意义。

2.2　营养 KAP 变化

2.2.1　营养知识的变化　干预后，浦东新区城区干预组 10 道营养知识题的知晓率显著提高，其中 2 道题知晓率高于对照组且差异有统计学意义。乡镇干预组 14 个营养知识题的知晓率显著提高，有 9 道题知晓率高于对照组且差异有统计学意义。见表 1。

表 1　干预前后营养知识知晓率的变化情况（%）

Table 1　Changes of nutritional knowledge before and after intervention（%）

营养知识	城区干预前		城区干预后		乡镇干预前		乡镇干预后	
	干预组	对照组	干预组	对照组	干预组	对照组	干预组	对照组
主食应为粮谷类食物	70.3	80.1	80.6*	81.3	52.9	59.8	71.6*	66.9*
多样化食物优于高蛋白食物	55.8	69.0	83.3*	77.7*	21.8	40.4	39.9*△	31.4*
精白米面不利于营养	64.7	73.2	85.8*	79.9	42.9	50.8	61.7*△	50.5
水果不能代替蔬菜	86.1	87.9	90.9	91.7	73.1	77.7	87.6*△	81.3
每天喝牛奶有益于健康	94.6	93.5	96.4	97.1	83.6	85.1	88.3	88.9
每天吃豆及豆制品有益于健康	85.5	90.5	96.1*	92.8	63.9	72.7	81.5*△	72.5
多吃肉类对健康无益	58.7	52.0	63.0	58.3	51.8	55.8	70.9*△	56.6
吃盐过多会增加患高血压的危险性	66.6	68.3	81.5*	79.1*	50.4	57.8	54.7	50.7*
胖并非健康的表现	85.5	85.9	91.2*	87.8	77.8	74.5	86.4*△	75.3
三餐合理的能量分配	24.9	26.5	30.0	30.9	17.8	12.0	39.7*△	22.6*
钙的主要来源	61.9	61.8	84.2*△	66.5	27.2	48.8	45.5*	43.0
维生素 C 的主要来源	82.0	84.0	84.8	87.8	71.0	68.8	79.1*	73.8
维生素 A 的主要来源	39.4	38.2	53.9*△	40.6	20.8	27.1	50.0*△	24.7
铁的主要来源	17.7	26.8	33.6*	28.4	4.4	16.7	42.5*△	11.9
脂肪含量最高的肉类	73.1	80.4	77.0	81.8	64.2	66.1	72.8*	73.4*
变质食品即使高温加热也不能食用	88.6	92.8	94.8*	94.2	77.5	77.9	86.6*	84.3*

注：* 干预后与基线数据比较 $P<0.05$；△干预后干预组与对照组比较 $P<0.05$

2.2.2　营养态度的变化　干预后，浦东新区城乡地区干预组营养态度改变均表现为上升趋势，乡镇地区小学生部分营养态度干预前后变化差异有统计学意义。见表 2。

表 2　干预前后营养态度变化情况（%）

Table 2　Changes of nutritional attitudes before and after intervention（%）

营养状态	城区干预前		城区干预后		乡镇干预前		乡镇干预后	
	干预组	对照组	干预组	对照组	干预组	对照组	干预组	对照组
想提高健康水平	98.1	97.7	99.4	99.3	95.6	97.3	96.2	98.1
对营养知识感兴趣	89.9	93.5	93.3	96.0	86.7	96.6	88.7	83.9*
想使饮食更符合营养要求	96.2	97.1	98.8	97.5	92.0	99.1	93.9	95.0
愿改变不良饮食习惯	98.1	98.0	98.5	99.3	89.2	99.5	91.5	93.5*
体重超重或过轻时，愿通过饮食调整	93.7	94.1	96.7	92.8	81.0	98.0	91.3*	91.8*

注：* 干预后与基线数据比较 $P<0.05$。

2.2.3　营养行为的变化　干预后，城乡地区干预组各有 3 项健康行为人数比例增加，城区干预组常吃西式快餐人数比例下降，吃早餐和体育锻炼人数比例高于对照组；乡镇地区干预组睡前不吃零食和体

?1994-2014 China Academic Journal Electronic Publishing House. All rights reserved.　http://www.cnki.net

育锻炼人数比例高于对照组，以甜饮料解渴和偏食人数比例低于对照组，以上行为变化差异有统计学意义。见表 3。

表 3 干预前后营养行为的变化情况(%)

Table 3 Changes of nutritional practices before and after intervention(%)

营养行为	城区干预前		城区干预后		乡镇干预前		乡镇干预后	
	干预组	对照组	干预组	对照组	干预组	对照组	干预组	对照组
每天吃新鲜蔬菜	76.0	85.6	82.5 *	84.5	64.9	60.9	74.0 *	68.7 *
每天喝牛奶	71.3	72.2	72.1	76.3	64.2	64.1	68.3	66.9
每天吃早餐	86.4	91.5	97 *△	90.6	84.1	81.9	85.0	82.8
睡前 2 h 不吃零食	43.8	45.1	50.3	47.5	31.1	30.7	41.8 *△	34.4
常吃零食	38.2	41.5	38.8	41.4	50.1	46.7	38.0 *	42.2
以甜饮料解渴	30.3	29.1	28.8	32.7	23.0	27.8	20.2△	30.6
常吃西式快餐	8.8	7.2	4.5 *	7.5	16.6	15.6	13.1	12.2
口味偏咸	15.6	11.8	12.7	11.2	10.5	8.6	10.1	12.8
偏食	31.5	36.3	33.0	33.5	37.5	38.6	30.8 *△	38.4
经常体育锻炼	25.6	26.1	40.0 *△	25.2	39.1	37.5	43.7 *	35.6

注：* 干预后与基线数据比较 $P<0.05$；△干预后干预组与对照组比较 $P<0.05$。

2.2.4 超重及肥胖小学生营养行为的变化　对超重及肥胖小学生干预前后与超重及肥胖相关的营养行为变化进行分析表明，城区干预组吃蔬菜、早餐、睡前不吃零食及体育锻炼等健康行为干预后较干预前人数比例上升，其中体育锻炼人数比例上升差异有统计学意义($P<0.05$)；以甜饮料解渴人数比例上升，吃西式快餐人数比例下降。乡镇地区干预组干预前后行为变化差异无统计学意义。

2.3 营养状况的变化　干预后，城区干预组营养不良和肥胖人数比例低于干预前，超重人数比例高于干预前；超重及肥胖人数比例低于对照组。乡镇干预组营养不良人数比例低于干预前，超重和肥胖人数比例高于干预前；肥胖人数比例低于对照组差异有统计学意义。见表 4。

表 4 浦东新区小学生干预前后营养状况的变化(%)

Table 4 Changes of nutritional status before and after intervention(%)

营养状况	城区干预前		城区干预后		乡镇干预前		乡镇干预后	
	干预组	对照组	干预组	对照组	干预组	对照组	干预组	对照组
营养不良	21.3	16.7	19.7	16.2	25.1	18.9	23.2	18.7
超重	13.9	9.2	14.8	15.8 *	9.8	8.6	13.4	13.4 *
肥胖	10.1	11.4	9.4	13.7	10.1	10.6	10.6△	16.4 *

注：* 干预后与干预前比较 $P<0.05$；△干预后干预组与对照组比较 $P<0.05$。

3 讨论

3.1 营养 KAP 干预效果　和文献报道营养干预能有效提高小学生营养知识知晓率的结果相一致[4-8]，干预后，浦东新区城乡地区干预组小学生营养知识知晓率均较干预前及较对照组均有所提高。这和小学生求知欲强，对营养教育有浓厚的兴趣有关[7]。干预后，干预组小学生营养态度更为积极，这和文献报道的：营养干预活动的开展，使小学生对营养知识产生兴趣，从而营养态度更加积极一致[4,8]。

营养行为方面，干预组小学生营养行为改善明显，但常吃零食和偏食人数比例的变化不明显，这和对南京市小学生营养健康教育的结果一致[9]，可以推测这些行为是小学生比较难以改变的行为；而且文献显示，营养干预后，学生营养知识增加明显，但营养行为的改变要缓慢些[4]。也因此提示要改善学生营养行为需长期反复开展多形式的营养干预活动。本次研究乡镇小学生营养行为改善效果较好。这可能和乡镇小学生基线时营养知识知晓率低于城区小学生，营养行为也较城区小学生更不健康，经过营养干预后，营养知识知晓率提高，营养行为改善效果更好有关。

对超重及肥胖小学生行为干预前后变化情况分析可知，城区干预组营养行为的改变更为积极，这可能和干预组学校干预阶段正在进行学校合并搬迁，未能像城区干预组一样实行肥胖及超重儿童的管理，所以小学生营养行为虽有一定改善，但肥胖超重小学生行为未能和城区干预组一样改变积极，特别是对控制超重肥胖有重要作用的锻炼行为干预前后并未得到改善。

3.2 营养状况的变化　干预后，城区干预组肥胖人数比例低于干预前人数比例，但超重人数比例较干预前人数比例高，可以推测，部分肥胖小学生干预后由肥胖变成超重，导致超重人数比例有所增加；城区小学生营养状况变化提示，如果不开展营养干预活动，随着社会经济的发展，食物的丰富，营养不良可能逐渐得到改善，但与此同时超重和肥胖的人数比例则也在上升，可知营养干预活动的开展对城区小学生超重肥胖的控制起着重要的作用。乡镇干预组与干预前比较，超重、肥胖控制效果并不理想，这可能和乡镇干预组超重及肥胖学生营养行为未能得到积极改善有关。与文献报道的结果一致，儿童肥胖

?1994-2014 China Academic Journal Electronic Publishing House. All rights reserved. http://www.cnki.net

的控制是一项难道较大的工作[10]。也因此提示, 控制儿童超重、肥胖, 仅靠一些面上的宣传教育难以取得很好的效果, 要通过对肥胖超重儿童定期监测体重, 并及时修正饮食及运动行为才能取得理想的控制效果。

综上所述, 对小学生开展营养干预活动, 可以有效提高小学生的营养知识和态度, 改善饮食行为, 改善营养状况, 促进小学生健康。

[参 考 文 献]

[1] 徐辉, 张燕青. 天津市河西区 2001～2005 年中小学生营养状况[J]. 中国学校卫生, 2007, 28(1): 58-59.
[2] 王万陵, 李英明. 重庆市万州区 2001～2005 年中小学生营养状况[J]. 中国学校卫生, 2007, 28(1): 56-57.
[3] 杜尉英, 郑晶泉, 胡佳, 等. 上海市浦东新区中小学生 2001～2004 年营养状况[J]. 中国学校卫生, 2006, 27(3): 227-228.
[4] 彭云, 程茂金, 王红, 等. 开展“健康促进学校”项目对小学生营养 KAP 的影响效果评价[J]. 医学与社会, 2004, 17(3): 16-18.
[5] 丁虹. 营养教育对不同人群营养知识、态度、行为的影响[J]. 中国食物与营养, 2005, 11(1): 55-57.
[6] Chang Xiashi, Wei Zhangxin, Yang Xushui, et al. Creating health-promoting schools in China with a focus on nutrition[J]. Health Promotion International, 2004, 19(4): 409-418.
[7] 陈钢, 徐锦杭. 小学生三餐行为和营养知识教育效果评价[J]. 中国学校卫生, 2004, 25(3): 324-325.
[8] 夏庆华, 孙建平. 中学生及其家长合理营养综合干预效果评价[J]. 中国学校卫生, 2006, 27(6): 481-482.
[9] 王少康, 池红, 胡永桢, 等. 南京市中小学生营养知识健康教育效果评价[J]. 中国学校卫生, 2008, 29(7): 588-589.
[10] 石建辉, 刘秀荣, 田向阳, 等. 北京市小学生肥胖干预效果分析[J]. 中国健康教育, 2004, 20(9): 782-785.

收稿日期: 2009-07-14

(上接第 205 页) 差异有统计学意义($P<0.01$)。在营养不良患病率中, 低体重患病率最高, 且低体重在 1 岁以下年龄组患病率(2.83%)明显高于其他年龄组(1.01%和0.73%), 差异有统计学意义($P<0.01$)。发育迟缓在 1～2 岁组患病率(0.65%)高于其他年龄组(0.30%和0.54%), 各年龄组有显著统计学意义($P<0.05$), 消瘦各年龄组无统计学意义($P>0.05$)。见表 1。

3 讨 论

3.1 3 岁以下婴儿营养不良原因分析 营养不良是严重危害儿童身心健康的一种常见病[3]。家庭人均收入低、家庭不和睦、母亲不知道合理营养、经常患病为常见危险因素[4]。东风新村位于大庆市中心, 这里的家庭经济状况较好, 由于经济问题导致的营养不良很少发生。但仍存在母乳不足或者使用代乳品调配不当导致的营养不良。4～6 个月是添加辅食重要时期, 此阶段辅食添加晚, 辅食添加的质和量不足是营养不良的主要原因。6～12 个月呼吸道感染和腹泻是门诊常见病, 如果护理不当也会影响婴儿体重的增长。1 岁以下经定期到儿童保健科体检, 对合理喂养、辅食添加进行宣传指导, 营养不良患病率能得到一定改善, 所以本文论述低体重随着年龄的增加, 营养不良患病率下降。1～2 岁是断奶的关键阶段, 部分儿童在 1 岁以下营养不良未及时纠正, 断奶期没能很好的过渡而导致慢性营养不良是 1～2 岁发育迟缓高于其他年龄组的原因。

3.2 预防策略 1)母乳喂养能降低营养不良的发生率: 母乳营养丰富且含有多种免疫物质, 能减少疾病的发生, 降低婴幼儿营养不良患病率。所以, 通过新生儿访视、健康体检等方式进行母乳喂养知识的宣教可提高母乳喂养率; 2)及时合理的添加辅食可降低营养不良的发病率: 针对辅食添加知识上的缺乏, 加强对家长进行辅食添加的时间、顺序、品种及辅食制作等方面知识宣教, 帮助婴儿从流质到固体食物过渡; 3)坚持体格锻炼加强腹泻的综合管理可降低营养不良的发病率: 应做好婴幼儿的日常生活护理工作, 坚持体格锻炼, 预防上呼吸道感染, 加强对小儿腹泻的综合管理, 减少疾病对营养不良的影响; 4)儿童获得良好的保健可降低营养不良患病率: 通过孕期母亲学校、爱婴医院的宣教及儿童定期系统体检, 母亲及看护人早期获得母乳喂养知识、辅食添加知识及防病知识, 大大宣传科学育儿的方法, 早期预防营养不良的发生, 使儿童健康地成长。

[参 考 文 献]

[1] 何自力, 万爱琴, 龙冰颜. 遵义市 7 岁以下儿童营养不良流行学调查分析[J]. 中国儿童保健杂志, 2008, 10(5): 285-286.
[2] 荫士安. 中国婴幼儿的生长发育与辅食添加现状[J]. 中国儿童保健杂志, 2004, 12(6): 509-511.
[3] 何永贵, 钟纯兰. 中度营养不良婴幼儿 442 例分析[J]. 中国儿童保健杂志, 2005, 2(1): 82-83.
[4] 蒋汝刚. 151 例儿童营养不良的病例对照研究[J]. 中国儿童保健杂志, 2006, 2(1): 73-74.

收稿日期: 2009-11-19

?1994-2014 China Academic Journal Electronic Publishing House. All rights reserved.　http://www.cnki.net
