# Supplementary material for: Systematic review and meta-analysis of school-based obesity interventions in mainland China
Source: PLoS One. 2017 Sep 14;12(9):e0184704. doi: 10.1371/journal.pone.0184704 (PMC5598996; doi:10.1371/journal.pone.0184704)
Supplement: S1 Dataset — (ZIP) [file pone.0184704.s007.zip › S1_dataset/76库/55.pdf]

文章编号: 1008-6579(2002)06-0364-04 【科研论著】

学龄儿童单纯肥胖症的群体干预研究

蒋竞雄, 夏秀兰, 吴光驰, 谈藏文, 宋小芳, 王力, 郭素怡, 闫桂风 (首都儿科研究所, 北京 100020)

中图分类号: R153.2 文献标识码: A

摘 要: 【目的】 探索儿童单纯肥胖症的群体干预方法, 降低学龄儿童肥胖发病率。 【方法】 从北京市城区选择 5 所小学作为研究现场, 该小学所有学生均为研究对象, 其中 2 所学校为干预组(1 597 人), 3 所学校为对照组(2 118 人)。采用 WHO 身高标准体重值为肥胖判断标准。对干预组进行为期 3 年的群体干预, 干预措施包括健康教育、增加运动和饮食调整, 重点为调整晚餐进食顺序、增加蔬菜水果的摄入量、减少静坐时间、增加运动等。干预对象为超重儿童及其家长、肥胖儿童及其家长、非肥胖儿童家长。对照组除与干预组同期体检外不接受任何干预措施。 【结果】 经过 3 年干预, 干预组肥胖发病率从 16.9% 降至 12.1% ($P < 0.01$), 对照组肥胖发病率从 17.4% 上升为 23.2% ($P < 0.01$)。干预后两组肥胖发病率差别极显著。对干预组 757 名 1～3 年级儿童进行追踪观察, 3 年后原非肥胖儿童中 3.6% 成为肥胖儿童, 原肥胖儿童中 59.1% 仍维持肥胖状态。对照组 1 031 名 1～3 年级儿童中, 有 11.9% 的原非肥胖儿童三年后为肥胖儿童, 原肥胖儿童中 92.2% 仍维持肥胖状态。干预组非肥胖儿童成为肥胖的危险性明显小于对照组, 对照组肥胖儿童维持肥胖的可能性明显大于干预组。 【结论】 在学校进行以健康教育为主的肥胖群体干预方案切实可行, 能有效地降低学龄儿童的肥胖发病率。

关键词: 肥胖; 群体干预; 儿童

School-based Intervention for Obese Children. *JIANG Jing-xiong, XIA Xiu-lai, WU Guang-chi, TAN Zang-wen, SONG Xiao-fang, WANG Li, GUO Su-yi, YAN Gui-feng.* (***Capital Institute of Pediatrics, Beijing***, 100020, ***China***.)

Abstract: 【**Objective**】 To evaluate the impact of a school-based intervention program to childhood obesity and to reduce the prevalence of obesity among school children 【**Methods**】 Two intervention and three control primary schools were selected from Beijing urban district, randomly, to attend the field trial. all the children (3 715 students, grade 1 to 6) from these 5 schools were involved the study and there were 1 597 in intervention schools (interventiong group) and 2 118 in control schools (control group), respectively. The children from 2 intervention schools participated in a school-based intervention program over 3 school years (from 1997 to 2000). The program included health education, exercise, and dietary adjustment. The progam focused on decreasing consumption of high-caloric foods and incerasing fruit and vegetable intake in supper, and decreasing sedentary time. Obesity was defined by WHO standard weight for height. Outcomes were assessed using pre-intervention and follow-up measures. 【**Results**】 The prevalence of obesity among the children in intervention group was reduced from 16.9% to 12.1% over the 3 school years intervention periods. The obesity prevalence among the children in control group increased from 17.4% to 23.2% over the 3 school years. The prevalence of obesity in intervention group was significantly lower than control group after intervention. Among the children in intrevention group, 3.6% of no-obese children became to obese and 59.1% of obese children remained obese over the 3 school years intervention periods. During the same 3 years, 11.9% of no-obese children became to obese and 92.2% of obese children remained obese among the children in control group. The obesity incidence was significantly less for the children who were no-obese in intervention than control group (odds ratio, 0.276, 95% confidence interval, 0.127～0.443, $P < 0.01$). The percent of obesity maintenance was significantly greater for the children who were obese in control vs intervention group (OR, 0.122, 95% CI, 0.062～0.240, $P < 0.01$). 【**Conclusion**】 The school-based intervention program was suitable and effective in school, and reduced prevalence of obesity significantly among school children.

Key words: obesity; intervention school-based; children

单纯肥胖症是儿童期常见的营养性疾病, 不仅损害儿童身心健康, 而且与成人期心血管疾病的发生密切相关。近十年来单纯肥胖症在我国儿童中呈上升的趋势, 已成为城市儿童的一大健康问题。单纯采用针对肥胖患儿的临床个案治疗已难以控制迅猛增长的肥胖发病率, 必须采取针对较大人群范围的干预方案才能从根本上减少肥胖的发生。此文对一组学龄儿童实施为期三年的肥胖群体干预方案, 旨在探索儿童肥胖的群体控制方法, 降低儿童肥胖发病率。

1 对象和方法

1.1 对象 从北京市城区选择 5 所小学的全体学生作

基金项目: 中国优生科学协会和北京市科干局青年科学基金资助

作者简介: 蒋竞雄(1963-), 女, 副研究员, 硕士学位, 科主任, 主要从事儿童保健研究工作。

?1994-2014 China Academic Journal Electronic Publishing House. All rights reserved. http://www.cnki.net

为研究对象(共 3 715 人)。按照随机和自愿相结合的原则将 5 所小学分为干预组 2 所(1 597 人)和对照组 3 所(2 118 人),对照组和干预组在学校所处地区、活动场地、体育课设置、伙食供应等方面基本相似。

1.2 方法

1.2.1 体格测量 体格测量在两组受试者中同时进行,以除外季节的影响。测量内容为身高及体重,由专人采用标准测量方法进行测量[1],体重精确到 0.1kg,身高精确至 0.1cm,每次测量前校对测量器具。体格测量每年进行 2 次,对照组除体格测量外不接受任何干预措施。采用 WHO 身高标准体重值为肥胖判断标准,超过标准体重 10% ~19%为超重,超过 20% ~29%为轻度肥胖,超过 30% ~40%为中度肥胖,超过 50%以上为重度肥胖。受试者中未发现病理性肥胖儿。

1.2.2 干预内容 ①健康教育 采取讲课、讨论、抢答问题等方式进行教育,宣教内容包括肥胖判断标准、现状分析、发病原因、对健康的危害、治疗原则、儿童热卡生理需要量、食物热卡含量、摄入量的计算方法、饮食调整方法、运动锻炼方法等。②运动锻炼 由学校体育老师督促肥胖和超重儿童上体育课时增加运动,由班干部督促本班肥胖及超重儿童课间增加体育活动,由家长督促儿童闲暇时间减少静坐及增加运动。③饮食调整 重点为晚餐,晚餐前如有饥饿感除蔬菜水果外不摄入任何食物,晚餐进餐顺序为先吃蔬菜、水果、汤等低热卡食物,后吃主食及其它食物,减慢进餐速度,晚餐后至睡前不再进食。上述饮食调整方案均不限制儿童的饮食量。

1.2.3 实施步骤 每学期对全体超重和肥胖儿童进行 1~2 次健康教育,对肥胖及超重儿童家长进行 1 次健康教育,对全体家长进行 1 次有关肥胖的知识讲座,每学期末给全体肥胖及超重儿童家长发家长信和宣教材料,告知其子女目前的肥胖或超重状况,假期应采取的干预措施。

1.3 统计学处理 全部数据输入计算机后采用 Microsoft Excel 软件处理,计数资料的对比分析采用 χ^2 检验和危险度分析。

2 结 果

2.1 干预前后肥胖发病率及超重发生率的变化 干预前基线调查干预组和对照组肥胖发病率分别为 16.9% 和 17.4%,超重发生率分别为 13.7%和 12.1%,两组间肥胖发病率和超重发生率均无显著差异。经三年干预后干预组肥胖发病率降至 12.3%,比干预前明显降低($P<0.01$),而对照组肥胖发病率上升为 23.2%,明显高于基线调查结果($P<0.01$),干预后两组间肥胖发病率差异也有极显著性($P<0.01$)。干预后干预组超重发生率也较干预前明显下降,对照组干预前后超重发生率无明显变化。见表 1。

表 1 两组儿童干预前后肥胖及超重状况的比较 ($\bar{x}\pm s$)

项 目	干预组		对照组	
	干预前	干预后	干预前	干预后
样本量(例)	1 597	1 559	2 118	2 045
男	860	841	1 005	1 047
女	737	718	1 033	998
肥胖发病率(%)	16.9	12.3 *	17.4	23.2○
男	21.9	15.8 *	21.1	25.8○
女	11.7	8.2	13.7	20.4○
超重发生率(%)	13.7	9.8 *	12.1	11.5
男	12.7	9.9	12.7	13.4△
女	14.6	9.8 *	11.4	9.5

注: *:自身前后 χ^2 检验 $P<0.01$; △:两组间同期检测结果 χ^2 检验 $P<0.05$, ○:两组间同期检测结果 χ^2 检验 $P<0.01$。

2.2 两组间毕业班和入学新生中肥胖发病率的变化 干预前毕业班肥胖发病率及每年入学新生肥胖发病率在两组学校间基本相似,无统计学差异。见表 2。

表 2 两组儿童肥胖发病率在毕业班和入学新生中的比较

受检人群	干预组		对照组		P 值
	人数	肥胖发病率(%)	人数	肥胖发病率(%)	
1997 年毕业班	281	16.7	289	19.1	$P>0.05$
1997 年新生	256	10.0	289	11.5	$P>0.05$
1998 年新生	258	11.3	308	11.7	$P>0.05$
1999 年新生	220	10.5	281	12.1	$P>0.05$
2000 年新生	234	11.1	292	11.3	$P>0.05$

2.3 部分研究对象肥胖变化状况的追踪观察结果 见表 3。

表 3 两组儿童肥胖变化情况的追踪观察结果

项 目			干预组	对照组	OR	95%可信限	U 检验
观察人数	总		725	1 031			
	男		377	531			
	女		348	500			
肥胖发病率	总	干预前(%)	15.9	16.2	0.981		
		干预后(%)	12.4	24.9	0.498	0.382, 0.647	$P<0.01$
	男	干预前(%)	21.8	21.9	0.996		
		干预后(%)	17.0	32.9	0.517	0.374, 0.715	$P<0.01$
	女	干预前(%)	9.5	10.2	0.931		
		干预后(%)	7.5	16.4	0.457	0.287, 0.727	$P<0.01$
非肥胖儿童三年后变为肥胖儿童的比例(%)	总		3.6	11.9	0.276	0.172, 0.443	$P<0.01$
	男		4.4	16.1	0.239	0.129, 0.442	$P<0.01$
	女		2.9	8.0	0.337	0.160, 0.710	$P<0.01$
原肥胖儿童三年后仍肥胖的比例(%)	总		59.1	92.2	0.122	0.062, 0.240	$P<0.01$
	男		62.2	93.1	0.121	0.052, 0.282	$P<0.01$
	女		51.5	90.2	0.115	0.037, 0.362	$P<0.01$

在两组学校分别选择干预前基线调查时处于 1~3 年级的所有儿童进行追踪观察,分析其在三年干预中体格发育的变化情况。干预组基线调查时 1~3 年级共有儿童 757 人,三年中转学 32 人,资料完整者 725 人。对照组基线调查时 1~3 年级共有 1 077 人,三年中转学 46 人,资料完整者 1 031 人。上述儿童资料显示,经过干预后干预组儿童肥胖发病率从 15.9%降至 12.4%,原非肥胖的 610 名儿童中有 22 人(3.6%)变为肥胖;原肥胖的 115 名儿童中有 68 人(59.1%)干预后仍维持肥胖状态。对照组三年后肥胖发病率从 16.2%上升至 24.9%,原非肥胖的 864 名儿童中有 103(11.9%)变为肥胖;原肥胖的 167 名儿童中有 154 人(92.2%)仍维持

?1994-2014 China Academic Journal Electronic Publishing House. All rights reserved. http://www.cnki.net

肥胖状态。干预后干预组儿童肥胖发病率明显低于对照组儿童，干预组非肥胖儿童成为肥胖的危险性明显小于对照组（OR 值为 0.276，95％可信限为 0.127～0.443，$P<0.01$），对照组肥胖儿童维持肥胖的可能性明显大于干预组（OR 值为 0.122，95％可信限为 0.062～0.240，$P<0.01$）。

3　讨　论

肥胖由遗传和环境因素所致，环境因素中不良的生活方式，包括运动量过少、静坐时间较长、饮食摄入过多等，是导致肥胖的重要原因。引起肥胖的环境因素与经济状况和生活方式密切相关，近十年来，我国在经济腾飞的同时，肥胖人数在城市也迅速增长，儿童单纯肥胖症的发病已处于失控状况，从 80 年代中期的 2.7％增至 90 年代中期的 17.7％[2,3]。90 年代以来，我国学者在儿童肥胖的治疗方面已开展多项研究并取得一定成效[4,5]，但小范围的个案治疗难以解决人群中肥胖这一健康问题，无法控制快速增长的肥胖发病率。只有采取针对肥胖儿童和非肥胖儿童的群体干预措施，在人群中提倡健康的生活方式，才能从根本上改变肥胖高发病率的状况。[6]。

学龄儿童是肥胖的高发人群，在学校实施肥胖干预方案十分必要，学校对学生的集体管理也使干预方案的实施易于取得成效[7]。在本研究中，进行干预的学校肥胖发病率从 16.9％降至 12.3％，下降速度为 27.2％；而未经干预的对照学校肥胖发病率从 17.4％上升至 23.2％，增长速度达 33.3％。干预前两组学校肥胖发病率基本相同，而干预后干预学校肥胖发病率较对照学校低近两倍，由此可见，我们采用的群体干预方案对降低学龄儿童肥胖发病率效果是极其显著的，否则，可以预见，如果不采取任何干预措施，今后数年间，儿童肥胖的发病率仍将继续上升。

肥胖的群体干预方案与临床治疗最大的区别在于覆盖人群范围不同[8]。该研究在实施干预方案时不仅针对肥胖儿童，而且将非肥胖儿童，尤其是超重儿童纳入干预方案的实施对象之中，以减少肥胖新增人数。从研究结果看，接受干预方案的学校超重发生率从 13.7％降至 9.8％，经统计学检验差异有极显著性。在干预方案实施过程中，超重人数始终处于动态变化之中，由于经干预后一部分肥胖儿童转化为超重儿童，因而干预效果有时在超重发生率的变化上不易显现。该资料显示在干预后肥胖发病率明显下降的同时，超重发生率也明显降低，从另一方面反映了干预方案的有效性。

以往的调查资料表明，学龄儿童中肥胖的主要危险因素为活动量少和饮食量过多，晚餐大量进食及饭后无活动时间是学龄儿童的主要不良生活方式[3]。在进行健康教育时我们给家长讲述学龄儿童的正常生理需要量，比较儿童每日进食量与生理所需，解除了家长担心子女营养不足的顾虑。大多数超重及轻度肥胖儿童的家长认为自己的子女体重很正常，仍在鼓励其过度进食。本群体干预方案进行干预的重点是首先让所有超重和肥胖儿童及其家长明确儿童目前的体格发育状态，了解肥胖对儿童身心健康的危害，晚餐改变进餐顺序（先吃水果、蔬菜及汤等低热卡食物），放学后至晚餐前除水果外不吃其他食物，但不限制饮食量，体育课及闲暇时间增加活动时间及活动量。以改变生活方式为主的肥胖干预方案已有许多成功的案例[9,10]，我们采取的干预措施从健康入手，强调改变不良的饮食和生活习惯。由于不限制儿童的饮食量，且循序渐进，具有可持续性，易于被儿童接受。

单纯性肥胖是国家教育部规定的学校进行防治的疾病之一。虽然许多学校将该营养性疾病的防治列入学校工作的议事日程，但由于缺乏具体切实有效的措施，肥胖发病率仍无法控制，该研究中对照学校肥胖发病率的迅速上升便是明证。该干预措施与学校日常工作有机结合，在不明显增加学校老师工作量的基础上贯彻干预方案，收到了明显效果。在干预方案的实施过程中未发现任何学生因接受干预方案而出现不良反应。

两组学校每年均有毕业班离校及新生入学，如果干预初始时两组学校毕业班肥胖发病率相差较大，或每年新生肥胖发病率有明显差异，将影响干预后两组学校肥胖发病率的比较。本资料显示，无论基线调查时毕业班学生或每年新入学儿童，肥胖发病率在两组学校均无显著差异，可从另一方面摒除学生流动对干预效果的影响。

我们在两组学校各选择一组基线调查时处于 1～3 年级的所有儿童进行为期三年的追踪观察，观察期间转学者除外。追踪观察人数在干预组和对照组各有 725 人和 1 031 人。三年后，干预组原非肥胖儿童中有 3.6％变为肥胖，原肥胖儿童中有 59.1％仍处于肥胖状态。而对照组原非肥胖儿童中转为肥胖者达 11.9％，原肥胖儿童中 92.2％维持肥胖状况，两组儿童的肥胖发病率也由基线调查时的同一水平，发展为干预后对照儿童肥胖发病率明显高于干预组儿童。该结果说明经过群体干预，不仅肥胖儿童的肥胖状况得以改善，而且非肥胖儿童成为肥胖的危险性也大大降低。已有文献报道，在学校进行肥胖的群体干预可有效地解决肥胖这一健康问题[11,12]。此资料再次显示，除外学生人数变动因素，仍可看出干预方案对学龄儿童单纯肥胖症的良好控制效果。

和临床治疗方案比较，肥胖群体控制方案相对较为宽松，对运动量、饮食量均未作严格限制，因而对中重度肥胖儿童治疗作用不明显。我们在对学龄儿童进行群

?1994-2014 China Academic Journal Electronic Publishing House. All rights reserved.　http://www.cnki.net

体干预后, 中、重度肥胖的比例未见明显改善。如何将临床治疗方法与群体干预措施有效结合,使肥胖发病率降低的同时改善肥胖儿童的肥胖状况, 降低其肥胖度,乃是今后的研究方向。

[参 考 文 献]

[1] 叶广俊, 主编. 儿童少年卫生学[M]. 第 3 版. 北京: 人民卫生出版社, 1995, 170-171.

[2] 吴光驰, 郭素怡, 王乃坤, 等. 北京地区 468 名少儿肥胖及血压改变的八年随访观察[J]. 中华医学杂志, 1997, 77: 18-21.

[3] 蒋竞雄, 夏秀兰, 吴光驰, 等. 北京市朝阳区 2377 名小学生肥胖检出率及原因分析[J]. 中国儿童保健杂志, 1999, 7: 155-156.

[4] 丁宗一, 蒋竞雄, 许金华. 肥胖儿童的运动处方[J]. 中华医学杂志, 1992, 72: 131-134.

[5] 蒋竞雄, 吴光驰, 郭素怡, 等. 儿童单纯肥胖症门诊治疗效果观察[J]. 中国儿童保健杂志. 2001, 9: 14-16.

[6] Whitaker RC, Wright JA, Pepe MS, et al. Predicting obesity in young adulthood from childhood and parental obesity[J]. N Engl J Med 1997; 337: 869-873.

[7] Grossman DC, Neckerman HJ, Koepsell TD, et al. Effectiveness of a violence prevention curriculum among children in elementary school: a randomized controlled[J]. JAMA 1997; 277: 1605-1611.

[8] Gortmaker SL, Peterson K, Wiecha J, et al. Reducing obesity via a school-based interdisciplinary intervention among young[J]. Arch Paediatr Adolesc Med 1999; 153: 409-418.

[9] Epstein LH, Valodki AM, Smith JA, et al. Effects of decreasing sedentary behavior and increasing activity on weight change in obese children[J]. Health Psychol 1995; 14: 1-710.

[10] Braet C, Winckel MV, Leeuwen KV. Follow-up results of different treatment programs for obese children[J]. Acta paediatr 1997; 86: 397-402.

[11] Luepker RV, Perry CL, Mckinlay SM, et al. Outcomes of a field trial to improve children dietary patterns and physical activity: the child and adolescent trial for cardiovascular health (CATCH)[J]. JAMA 1996; 275: 768-776.

[12] Stone EJ, Baramowski T, Sallis JF, et al. Review of behavioral research for cardiopulmonary health: emphasis on youth gender and ethnicity[J]. J Health Educ 1995; 26(suppl): S9-S17

收稿日期: 2001-08-06

文章编号: 1008-6579(2002)06-0367-01

【基层儿保园地】

三胞胎合并症 5 例的治疗分析

汪勇芬, 王鲤珍 (福建医科大学附属第二医院儿科, 福建 泉州 362000)

中图分类号: R725.7 文献标识码: B
关键词: 多胞胎; 合并症; 治疗

多胎妊娠的发生率逐年增多。为了加强对多胞胎高危儿的治疗, 我们对 2000 年 2 月～2001 年 12 月我科共收治的三胎围产儿 5 例, 总结分析如下。

1 临床资料

1.1 *一般资料* 5 例均为剖宫产三胎儿, 胎龄 34～36 周, 体重 1 300～2 500g; 2 例新生儿出生时轻度窒息, 其余出生情况好; 合并缺氧缺血性脑病(HIE) 3 例, 硬肿症 2 例, 应激性溃疡 2 例。

1.2 *母亲情况* 所有产妇均于产前诊断为多胎妊娠, 并定期产科随访, 3 例分娩前合并妊高征, 2 例于怀孕前曾服用刺激排卵的药物。

1.3 *治疗及转归* 入院后予以保暖、吸氧、补液、预防感染、治疗合并症等处理, 生后第 1 日予以丙种球蛋白 500～750mg/kg 预防感染, 体重< 1 500g 的 4 个早产儿予以苯巴比妥预防颅内出血, 首次 15～20mg/kg, 24 小时后 5mg/(kg.d), 连用 3 天; 应激性溃疡出血 2 例予以胃管内注入思密达, 2～3 次/d, 出血很快得到控制; 合并 HIE 的按九五攻关项目协作组通过的治疗方案相应治疗; 加强营养支持, 奶量摄入不足者予以静脉高营养等治疗。转归: 均治愈出院, 出院后定期随访, 6 个月时智力及体格发育基本正常。

3 讨 论

近几年多胎妊娠逐年增多, 其因可能和母亲怀孕前服用刺激排卵的药物有关。随着多胎妊娠的增多, 多胎围产儿的监护和管理逐渐成为新生儿科医师的重要工作之一。多胎低体重儿及早产儿常见, 并发症较单胎儿多且重, 正确处理并发症是降低围产儿死亡率的关键; 预防早产, 积极治疗母亲妊高征, 促进胎儿肺成熟及促进宫内发育, 可提高多胎围产儿的成活率。5 例三胞胎均成活, 其因和分娩前的监护和处理有关。

由于保护性 IgG 抗体只有在妊娠末期才能大量经胎盘进入胎儿, 多胎早产儿未能获得足够抗体, 易于合并感染, 丙种球蛋白可提高 IgG 水平, 且可激活补体系统, 调节免疫缺陷。本组病例于生后第 1 日即应用丙种球蛋白, 所有病例均未发现明显感染存在; 消化道出血在新生儿多见, 其因大多为应激状态诱发胃应激性溃疡和胃粘膜糜烂, 思密达能覆盖消化道, 具有恢复上皮组织和止血作用, 我们对消化道出血患者胃管内注入止血, 取得良好疗效; HIE 在多胎儿中多见, 其病因和母亲妊高征致胎儿宫内缺氧有关, 治疗上我们注意维持良好通气及脏器血液灌注, 及时控制惊厥等治疗, 并加强随访, 3 例 HIE 患者智力及体格发育均正常。早产儿颅内出血的发生率高, 苯巴比妥对于降低颅内出血的预防作用已得到多数学者的肯定, 我们对体重< 1500g 的早产儿予以苯巴比妥, 所有病例未发现颅内出血发生; 此外在治疗中我们尚注意加强营养、保暖、护理、补液等综合措施, 密切监护患儿, 及时处理病情。

通过 5 例三胞胎的成功治疗, 我们体会到产前监护很重要, 应尽量延长怀孕周期, 出生后加强新生儿的监护, 及时处理合并症, 可提高存活率, 减少后遗症的发生。

收稿日期: 2002-06-01

?1994-2014 China Academic Journal Electronic Publishing House. All rights reserved. http://www.cnki.net
