# Supplementary material for: Systematic review and meta-analysis of school-based obesity interventions in mainland China
Source: PLoS One. 2017 Sep 14;12(9):e0184704. doi: 10.1371/journal.pone.0184704 (PMC5598996; doi:10.1371/journal.pone.0184704)
Supplement: S1 Dataset — (ZIP) [file pone.0184704.s007.zip › S1_dataset/76库/57.pdf]

综合行为疗法治疗青春期肥胖的研究

柏均青
(天津市六十一中学,300050)

摘　要　我校对32名12～15岁单纯性肥胖儿采用了学校、家庭、个人有机配合,实施多种措施,适当控制饮食,加强体育运动,建立良好生活习惯的综合行为疗法。经过两年的治疗,他们的肥胖程度显著下降($P<0.01$),而身高增长值和肥胖对照组、正常对照组基本一致。SBP、DBP、TC、TG、体脂百分含量(F%)也有一定程度下降。

关键词　单纯性肥胖　行为疗法　学生

进入80年代以来,青少年肥胖呈现增高的趋势。为使孩子们能够在体格方面协调发育、茁壮成长,减肥工作应该引起学校、家庭,乃至全社会的高度重视。近年我校对肥胖儿采用控制饮食,运动训练、建立良好生活习惯的综合行为疗法,取得了较为理想的效果,现将有关情况报道如下。

1　对象与方法

1.1　对象:选择我校12～15岁的肥胖学生和正常体重学生为受试者,体重超过标准体重20%为轻度肥胖,超过30%为中度肥胖,超过50%以上者为重度肥胖。对可疑病理性肥胖者进行内分泌系统的化验检查等,筛选出单纯性肥胖儿63例,根据自愿的原则,将此肥胖儿分为治疗组32例和对照组31例,同时在肥胖儿所在班级按同性别、同年龄的原则随机选择正常体重学生32名作为正常对照组。三组学生男女之比分别为23:9,20:11和23:9。肥胖学生肥胖度轻:中:重的比例为9:15:8和8:14:9。三组学生的年龄$\bar{x}\pm s$(岁)分别为14±1.3,14±1.5和14±1.3。

1.2 .方法:

1.2.1　检测项目:测量身高、体重、皮褶厚度,由专人负责按常规要求[1],使用国家体委科研所研制的体检器械进行检测。脉博、血压的测量,按照全国学生体质健康状况调研检测细则[2]进行。生化检验,清晨空腹取静脉血,用生化酶法测定血清总胆固醇(TC)、甘油三酯(TG)。肥胖度按超过身高标准体重的百分数计算,体脂百分含量通过所测皮脂厚度代入下列回归方程[3]进行计算。

	男生	女生
10～12岁	F% =9.0870+0.6616X	F% =11.2657+0.5311X
13～15岁	F% =4.8942+0.5496X	F% =10.8048+0.3614X
16～18岁	F% =3.6836+0.4097X	F% =8.4724+0.4249X

X:肱三头肌部及肩胛下角部皮褶厚度之和。

F%:体脂百分含量。

1.2.2　行为指导:通过病史询问、问卷调查、体格检查等方式摸清情况,在此基础上,帮助每位参加治疗的肥胖儿分析肥胖原因,设计减肥方案,确定减肥目标。这些要由校医、家长、肥胖儿共同商讨决定。并向孩子讲明,减肥是一个长期过程,要有信心、恒心和毅力,要增加减肥的自觉性。家长要对孩子饮食、运动和生活方式进行监督。学校定期检查执行情况,进行指导。

1.2.3　饮食疗法:开办肥胖儿和家长的减肥学习班,向他们介绍人体每日热卡生理需要量的计算方法,教会如何计算摄入食物的含热量,把日常食物的热卡量表印发给家长,介绍减肥食品、低热量食谱,要求在家庭中落实,定期反馈。

1.2.4　体育活动:根据参加减肥者的肥胖程度,本人可以耐受的减肥速度,要求每人每天进行30～60分钟的全身性有氧运动。运动方式为走路、长跑、各种球类、跳绳、健身操、游泳等均可。运动强度可为最大心率的65%,运动后即刻测试他们的脉搏应为110次/分左右。并把日常活动及各项运动热量消耗表介绍给他们,便于自行掌握。

2　结果

2.1　治疗组肥胖程度下降:肥胖治疗组经过两年的治疗,肥胖程度显著下降,肥胖对照组其肥胖度基本上没有什么变化,而三组学生的身高增长值接近(表1)。

2.2　血压、血脂等的变化:肥胖治疗组学生的血压、总胆固醇、甘油三酯、体脂率均有一定程度的下降,肥胖对照组学生上述指标无明显变化(表2)

2.3　行为矫正:肥胖治疗组学生不良饮食习惯、懒惰行为、自卑情绪均有所改变,体育成绩有一定程度的提高,而肥胖对照组以上各项基本无变化。

?1994-2015 China Academic Journal Electronic Publishing House. All rights reserved.　http://www.cnki.n

表 1　减肥治疗前后学生体格测量值的变化($\bar{x}\pm s$)

项　目		肥胖治疗组 (n=32)	肥胖对照组 (n=31)	正常对照组 (n=32)
身高(cm)	治疗前	162±7	163±8	162±8
	治疗后	170±8	171±9	170±9
体重(kg)	治疗前	71±9	71±10	50±6
	治疗后	74±8	82±9	61±8
肥胖度(%)	治疗前	38±11	39±11	-
	治疗后	26±10*	38±13	-

治疗前后比较差异均有显著性意义　* $P<0.01$

表 2 治疗前后肥胖学生血压、血脂和体脂含量的变化($\bar{x}\pm s$)

项　目		肥胖治疗组 n=32	肥胖对照组 n=31
收缩压(kpa)	治疗前	16.7±1.7	16.4±1.9
	治疗后	14.9±1.4	16.1±1.9
舒张压(kpa)	治疗前	10.9±1.4	11.1±1.5
	治疗后	9.4±1.3	11.2±1.6
总胆固醇	治疗前	4.8±0.7	4.6±0.8
(mmol/L)	治疗后	4.3±0.6	4.7±0.7
甘油三酯	治疗前	1.7±0.3	1.7±0.5
(mmol/L)	治疗后	1.5±0.4	1.8±0.3
体脂占体重	治疗前	35.6±8.7	36.2±8.9
比例　(%)	治疗后	26.2±7.9	35.9±9.8

3　讨论

经实践研究证明,学校、家庭、个人相结合的综合行为疗法治疗青春期肥胖安全、有效、可行。参加治疗的肥胖学生经过二年的治疗,肥胖程度明显下降,而身高的增长速度与二组对照组一致,体重保持较缓慢的增长状态,达到了治疗效果。同时,这些同学的血压、血脂也明显降低,体脂率下降,减少了心血管疾病的危险因素,符合国内外学者所倡导的治疗肥胖症的方向。

在行为治疗过程中,家长明白了减肥的重要性,懂得了减肥的道理,知道如何安排平衡而合理的减肥膳食,纠正了他们喜甜食、油炸、油腻食物,爱吃零食,饮食过量,进食速度过快,以及睡前进食等饮食行为上的习惯。督促孩子多参加户外活动,让孩子适当做一些力所能及的家务劳动。两年中,随着肥胖程度减轻,他们对体育运动逐渐产生了兴趣,体育测试成绩和运动水平也有所提高,作息、睡眠合理安排,良好的生活习惯逐渐养成,学习成绩也取得了进步。由此,他们的心理压力减轻了,自信心增强了,情绪也获得了很大的改善,综合行为治疗得到了满意的效果。

参 考 文 献

〔1〕 唐锡麟.儿童少年卫生学.第一版.北京:人民卫生出版社,1986:169.

〔2〕 中国学生体质与健康研究组.中国学生体质与健康研究.第一版.北京:人民教育出版社.1987:11~35,401~433.

〔3〕 元田恒,等.儿童少年体脂含量与肥胖评价标准的研究.学校卫生,1987,8(4):21.

中专学生对性教育的认识调查分析

李　玮

(湖北省武汉市城乡建设学校,430051)

当前,性教育是教育战线不可忽视的重要内容。为此,我们于 1994 年 5~6 月对本校 622 名中专学生进行了性教育认识状况调查,现将调查结果分析如下。

1　对象与方法

1.1　对象:武汉市城乡建设学校 1~4 年级的中专学生。一年级 163 人,二年级 165 人,三年级 140 人,四年级 154 人。共计男生 361 人,女生 261 人。年龄为 15~19 岁。

1.2　方法:采用自行设计的调查问卷,共有 13 个题目,每题有分式答案 3~4 个。为了提高真实性,采取无记名、短时间交卷,凡在答案中有更改或答题不全者均不作统计。发调查问卷 638 份,收回 635 份,无效资料 13 份,应答率 97.49%。

2　结果

2.1　中专生对性教育的态度:中专生认为性教育应当进行与积极进行者占调查总数的 94.37%;认为没有必要进行的占 5.63%($P>0.01$);对性教育内容,

?1994-2015 China Academic Journal Electronic Publishing House. All rights reserved.　http://www.cnki.n
