# Supplementary material for: Systematic review and meta-analysis of school-based obesity interventions in mainland China
Source: PLoS One. 2017 Sep 14;12(9):e0184704. doi: 10.1371/journal.pone.0184704 (PMC5598996; doi:10.1371/journal.pone.0184704)
Supplement: S1 Dataset — (ZIP) [file pone.0184704.s007.zip › S1_dataset/76库/58.pdf]

# 肥胖儿童运动疗法的效果观察

梁来强[1] ,陆阿明[1] ,范旭东[2]

(1. 苏州大学体育学院 ,江苏 苏州 215006 ;2. 哈尔滨体育学院 ,黑龙江 哈尔滨 150008)

**摘 要**:为探讨健身运动对肥胖儿童身体健康的影响 ,为肥胖儿童健身运动处方的制定与实施提供理论与实践依据。随机选择 20 名肥胖儿童进行为期 12 周的综合运动训练 ,测试运动前后的身体形态及机能的多项指标 ,并与随机选择的 20 名肥胖儿童(未接受运动处方)的相应指标进行比较 ,结果表明接受训练的肥胖儿童的各项身体指标变化与未接受训练的肥胖儿童的各指标变化的比较 ,具有显著或非常显著的差异 ,运动效果显著。运动能有效地降低体脂 ,增加瘦体重 ,改善儿童心肺功能和身体素质。

**关键词**:肥胖儿童 ;运动 ;效果

## Observation Effects of Movement Therapy for Fat Children

LIANG Lai - qiang[1] ,LU A - ming[1] ,FAN Xu - dong[2]

(1. Physical Education Institute of Soochow University ,Suzhou 215006 ,Jiangsu China ;
2. Harbin Institute of Physical Education ,Harbin 150008 ,Heilongjiang China)

**Abstract** : In order to probe into the impact of body building sports on fat children 's health , offer the theory and practice basis for formulation and implementation of fat body building prescriptions of children. We Choose 20 fat children to be trained the comprehensive sport which lasted for 12 weeks at random , then test health form and multiple indexes of the function before and after the sport , compareing with 20 fat corresponding index of children (not accepting the sport prescription ) which are choosen at random ,the comparison of all health index between the trained and no - trained fat children is remarkable that is the effect of sport is nemarkable. Sport can reduce body fat ,increase tight weight and improve childrens heart and lung function and physique effectively.

**Key words** : Fat children ; Sport ; Result

随着我国经济的飞速发展 ,人民生活水平的日益提高 ,儿童肥胖症在我国的发生率也越来越高 :1980 年 ,在北京抽样调查中 ,单纯性肥胖儿童的发生率为 3. 28 %;90 年代为 11. 25 %;1996 年全国 30 多个省、市、自治区 30 万中小学生调查的结果 ,男生中肥胖超重者由 10 年前的 3. 75 %上升到 8. 65 % ,女生中肥胖超重者由 3. 28 %上升到 7. 18 %;而且 ,由肥胖引起青少年高血压、冠心病、糖尿病等疾病的发病率也明显增加 ,儿童肥胖发展为成年肥胖的可能性较非肥胖儿童高 2. 33 倍[1] 。在第 22 届国际儿科学术会议上 ,专家指出 :儿童期单纯性肥胖症不仅是一个严重的健康问题 ,而且是一个潜在的社会问题。因此 ,在社会经济高速发展的今天 ,如何采取有效的措施去消除导致儿童肥胖的诸多因素 ,最终降低肥胖病的发生率 ,是提高我国国民健康素质和人口质量重要的医学和社会学问题。本文通过对 20 名肥胖小学生进行为期 12 周的综合性运动处方训练 ,旨在探讨对处于生长发育年龄阶段的小学生 ,以运动为主要减肥手段的确切疗效 ,为肥胖儿童的卫生保健提供一份安全有效、简便易行、适合儿童身心特点的肥胖儿童健身运动处方。

## 1 研究对象与方法

### 1. 1 研究对象

单纯性肥胖儿童 40 名 ,其中苏州市吴江实验小学 20 名肥胖小学生作为运动组 ;吴江市松陵小学 20 名肥胖小学生作为对照组 ,平均年龄为 10. 59 ±1. 27 岁。肥胖诊断依据[2] ,为男性学龄儿童 BMI〔体重指数 ,即体重 (kg) /身高 2 (m)〕> 18 ,女性学龄儿童 BMI > 17. 5。实验前两组肥胖儿童各项指标无明显差异(见表 1) 。

### 1. 2 研究方法

1. 2. 1 文献资料法 通过查阅有关资料 ,制定一套相应的运动处方 ,其原则为在保证运动量与运动强度的前提下 ,保证处方锻炼能长期进行、推广 ,处方内容的制定符合下列要求 :简单、安全、对场地器材的要求低。该处方的主要内容如下 :第 1～2 周 : (1) 步行 3～5min ,逐步加快速度 ,至心率达到 100～110 次/min ,时间相同 ,根据个人能力调节速度。(2) 广播体操和准备活动 5～7min ,使心率保持在 100～110 次/min ,根据个人能力调节运动力度。(3) 跳绳或踢毽 10min ,要求掌

**收稿日期**:2006 - 10 - 05 ;**修回日期**:2006 - 12 - 14

**第 1 作者简介**:梁来强 ,男 , (1981 - ) ,苏州大学体育学院在读硕士研究生 ,研究方向为健身运动的生理学原理与方法。

© 1994-2010 China Academic Journal Electronic Publishing House. All rights reserved. http://www.cnki.net

握技术和花样,心率仍保持在 100～110 次/min,通过间歇调整运动强度。(4)慢跑(游戏性)10～15min,在运动中心率上升至约 120～130 次/min。(5)斜式俯卧撑、立卧撑 10 个左右,做 2 组。(6)整理运动 5min 左右。注意:运动时间开始应短一些,控制运动量和运动强度,以运动时没有强烈的难受感觉和运动后反应不明显为标准,千万注意防止出现运动畏惧。第 3～4 周:巩固以上练习,逐步增加要求,重在培养学生的运动兴趣。因此,在运动中指导者应考虑用游戏等方式在各项运动间的过渡,灵活掌握。第 5～8 周:(1)广播体操和准备活动 5～10min,使心率上升至 100～110 次/min。(2)俯卧撑、立卧撑 10 个左右,做 3～4 组,控制间歇使心率保持在 100～110 次/min。(3)骑自行车、慢跑结合运动(游戏式)20～25min,运动时的心率控制在 120～140 次/min。(4)整理运动 5min 左右。注意:这一阶段运动总时间应保证在 45min 以上,对于女生,可做一些韵律操,而对于男生可采用踢球等方式,运动编排的目的是让学生提高运动的兴趣,养成运动锻炼的习惯,同时要注意个体差异,需要对部分不能坚持的学生做一些专门的辅导。第 9～12 周:基本内容与要求同上,可能的话女生以健身操、男生以踢球代替部分慢跑,同时注意发展一些柔韧素质和力量素质的练习。运动时间保证在 60min 左右,提高学生的运动能力和运动习惯。运动频率:3 次/周。

表 1　实验前两组肥胖儿童的各项身体和机能指标的比较

| | 对照组 | 运动组 | $p$ |
|---|---|---|---|
| 身高(cm) | 145.87 ±6.12 | 145.85 ±7.53 | >0.05 |
| 体重(kg) | 50.12 ±6.59 | 48.74 ±8.06 | >0.05 |
| 肺活量(ml) | 1876.9 ±465.77 | 1910.25 ±475.79 | >0.05 |
| 握力(kg) | 16.16 ±3.14 | 15.71 ±4.47 | >0.05 |
| 坐位体前屈(cm) | 3.88 ±5.98 | 4.55 ±6.95 | >0.05 |
| 仰卧起坐(次/min) | 19.23 ±9.50 | 17.8 ±8.14 | >0.05 |
| 立定跳远(cm) | 114.38 ±15.14 | 118.7 ±15.44 | >0.05 |
| 台阶实验指数 | 49.79 ±10.36 | 52.53 ±12.60 | >0.05 |
| 上臂部皮褶厚度(mm) | 25.76 ±4.61 | 26.55 ±6.40 | >0.05 |
| 肩胛下角皮褶厚度(mm) | 28.74 ±8.69 | 29.15 ±11.15 | >0.05 |
| 腹部皮褶厚度(mm) | 6.81 ±6.07 | 337.65 ±9.03 | >0.05 |

1.2.2　实验法　通过一套固定仪器和方法测试两组肥胖儿童的身体形态、心肺功能与身体素质的多项指标,具体如下:身高、体重、肺活量、握力、坐位体前屈、仰卧起坐、立定跳远、台阶实验指数、上臂部/肩胛下角/腹部皮褶厚度、体脂%(体脂%=(4.570/体密度-4.142)×100%,其中对于 9～11 岁少年儿童体密度可根据"推测体密度的回归方程式"推算出来,其公式如下:男子为 D=1.0879～0.00151X,女子为 D=1.0794～0.00142X(注意:X 为上臂部与肩胛下角两处皮褶厚度之和,D 为体密度[3])(以上测试由吴江市国民体质监测中心完成),测试方法按照国民体质测定各指标的要求进行,其中身体成分由韩国生产的 INBODY3.0 身体成分测定仪测定。

1.3　统计学分析

两组肥胖儿童的测试结果采用平均数差异 t 检验(实验后)。

## 2　研究结果

### 2.1　运动对肥胖儿童形态的影响

实验后两组肥胖儿童的体重变化不显著。实验后运动组各部分皮褶厚度和体脂%的下降较对照组显著,BMI 亦显著(见表 2)。

表 2　实验后两组肥胖儿童形态指标的比较

| | 对照组 | 运动组 | $p$ |
|---|---|---|---|
| 体重(kg) | 50.19 ±8.25 | 48.30 ±8.51 | >0.05 |
| 上臂部皮褶厚度(mm) | 26.18 ±7.19 | 24.45 ±6.16 | <0.05 |
| 肩胛下角皮褶厚度(mm) | 29.36 ±8.65 | 27.4 ±8.19 | <0.05 |
| 腹部皮褶厚度(mm) | 37.13 ±7.44 | 32.53 ±8.34 | <0.05 |
| 体脂% | 50.12 ±13.24 | 44.88 ±10.75 | <0.05 |
| BMI | 25.2 ±2.48 | 24.9 ±2.78 | <0.05 |

### 2.2　运动对肥胖儿童心肺功能的影响

实验后,运动组的肥胖儿童的肺活量和台阶实验指数较以前有所提高,且较对照组显著(见表 3)。台阶实验指数的增加,表明受试者心肺功能改善,心脏对运动的适应能力较为显著的增强。

表 3　实验后两组肥胖儿童心肺功能指标的比较

| | 对照组 | 运动组 | $p$ |
|---|---|---|---|
| 肺活量(ml) | 1774.45 ±456.23 | 1978.17 ±411.98 | <0.05 |
| 台阶实验指数 | 49.77 ±11.03 | 52.49 ±10.75 | <0.05 |

### 2.3　运动对肥胖儿童身体素质的影响

经过 12 周的运动锻炼,受试者握力、坐位体前屈、仰卧起坐、立定跳远的成绩大都有所提高,较对照组显著(见表 4)。

表 4　实验后两组肥胖儿童各项身体素质的比较

| | 对照组 | 运动组 | $p$ |
|---|---|---|---|
| 握力(kg) | 16.03 ±3.63 | 16.71 ±4.28 | >0.05 |
| 坐位体前屈(cm) | 0.57 ±7.58 | 7.07 ±7.26 | <0.05 |
| 仰卧起坐(次/min) | 20.38 ±8.33 | 25.46 ±7.26 | <0.05 |
| 立定跳远(cm) | 112.3 ±13.84 | 125.5 ±13.47 | <0.05 |

## 3　分析与讨论

大量的研究显示,运动作为减肥的最有效、最安全的方法之一,是因为:人体运动时主要能源来自糖和脂肪。有氧运动中,肌肉收缩活动初期能源为糖,当持续运动达 120min

© 1994-2010 China Academic Journal Electronic Publishing House. All rights reserved.　http://www.cnki.net

以上时,游离脂肪酸供能达 50 %～70 %之多。因此时肌肉对血中游离脂肪酸和葡萄糖的摄取和利用增多,导致脂肪细胞释放大量的游离脂肪酸,使脂肪细胞瘦小;同时使多余的脂肪被消耗而转化为血糖,结果体内脂肪减少,体重下降[4]。还有研究[5]认为,运动使交感神经兴奋性提高,儿茶酚胺活性增强,脂类氧化酶的数量增加、活性增强,血浆胰岛素水平下降,从而加强了脂类代谢,加速富含甘油三酯的乳糜和低密度脂蛋白的分解,最终加快游离脂肪酸的作用。另外,肥胖者进行适宜强度的运动训练后,常发生正常的食欲下降,摄食量减少,从而限制了热量的摄入,使机体能量代谢出现负平衡,引起体脂的减少。尽管实验前后受试者的体重变化不明显,但是瘦体重明显提高,体脂 %下降。此运动处方锻炼可有效减少学龄儿童的体脂,改善体脂成分,改善机体的形态特征。

肺活量是指最大吸气后,尽其所能呼出的最大气量,它反映了一次通气的最大能力,是最常用的测定肺通气机能指标之一。有研究表明,肥胖儿童肺通气功能明显低于正常儿童[6],因此在日常生活中,体力负荷稍一增加,肥胖儿童就会感到气喘和呼吸困难;另有研究表明,瘦体重与肺通气功能关系较为密切[7]。因此,肥胖儿童更应通过加强体育锻炼促进肌肉的发育,增加瘦体重,以提高肺的通气功能。在运动时,机体代谢旺盛,呼吸系统将发生一系列变化以适应机体代谢的需要。呼吸加深加快,肺通气量增加,科学的运动锻炼可使锻炼者呼吸肌发达、胸围增大、肺和胸廓弹性增强,肺活量加大。而台阶实验特别适应作为平时的健身活动或参考体育活动少的人群有氧能力的评定,而有氧运动的主要作用是提高机体的有氧能力和心肺功能,Geoorgia 对 74 名 7～9 岁的儿童进行了 4 个月的平均 5 天/周,心率在 157 ±7 次/min 的训练(每次消耗能量约为 925 ±201KJ),训练后总的心肺功能显著性提高,有氧能力也显著性增强[8],与本实验的结果相似。从本实验 2.2 结果可以看出运动能改善心肺功能,使机体很好的适应外界的环境。

运动能够有效地增粗肌纤维,增加其弹性,所以它能提高机体的各项身体素质。有研究表明肥胖儿童肌肉有氧代谢能力弱、效力低、运动能力低下[9]。因此,发展肥胖的身体素质也是进行健身运动处方锻炼目标之一。经过 12 周健身运动处方锻炼后,受试者各项素质均有所提高,结果提示本健身处方确实可有效发展肥胖儿童的身体素质。

## 4 小结与建议

(1)运动对肥胖儿童减去体重、降低皮褶厚度、改变身体组成成分,具有显著意义。

(2)运动对肥胖儿童的心肺功能产生良好的效果,使其更好地适应运动强度,提高他们的运动能力。

(3)运动对肥胖儿童的身体素质和机体整体活动能力均有显著的影响,肥胖儿童身体素质的提高有利于其建立良好的身体活动自信心,这对培养其运动习惯具有重要意义。

(4)建议在实施肥胖儿童运动处方时应执行综合的运动处方,在传统的减去体重、增加心肺功能的同时,要考虑到增强肥胖儿童的力量和整体活动能力,便于其在运动中建立自信心,养成坚持体育锻炼的良好习惯。

(5)若能将运动与饮食有机地结合起来,机体处于热量负平衡状态,势必增加脂库脂肪的动用,从而减少体脂含量。减肥效果更合理、更明显。

(6)建议教育部门和家长重视肥胖儿童的运动情况,并对其实施合理的、科学的综合运动处方,从而塑造一个健康的体质,增强他们的身体素质,更好的适应自然和社会。

## 参考文献:

[1] 杜熙茹. 健身运动对肥胖儿童健康的影响[J]. 广州体育学院学报,2003,23 (1):37 - 38.

[2] 焦东海. 儿童肥胖症防治[M]. 上海:上海文汇出版社:1998. 12 - 13.

[3] 邓树勋,等. 运动生理学[M]. 北京:高等教育出版社,1999. 418 - 419.

[4] 周鼓娟,等. 单纯性肥胖症儿童血脂变化及运动的影响[J]. 福建医药杂志,1999,21(4):17 - 18.

[5] 丁宗一. 中国儿童单纯性肥胖症现状、趋势及防治对策[J]. 天津体育学院学报,1999,1(1):12 - 14.

[6] 叶永延,等. 少年、儿童肺功能的初步研究[J]. 体育与科学,1994,(4):25 - 27.

[7] 王仁刚,等. 肥胖儿童肺通气功能的研究[J]. 广州体育学院学报,1997,17(3):38 - 42.

[8] Scott owens,etal. Effect of physical training on total and visceral fat in obese children[J],Med Sci Sports Exerc. 1999,31(1):164 - 170.

[9] 李珍妮,等. 对广州市 30 名肥胖小学生实施综合减肥法的疗效观察[J]. 广州体育学院学报. 1993,(4):11 - 15.

© 1994-2010 China Academic Journal Electronic Publishing House. All rights reserved. http://www.cnki.net
