# Supplementary material for: Systematic review and meta-analysis of school-based obesity interventions in mainland China
Source: PLoS One. 2017 Sep 14;12(9):e0184704. doi: 10.1371/journal.pone.0184704 (PMC5598996; doi:10.1371/journal.pone.0184704)
Supplement: S1 Dataset — (ZIP) [file pone.0184704.s007.zip › S1_dataset/76库/59.pdf]

动, 城乡以及家庭人均经济收入; 女性则分别为体力活动、年龄和职业。两性第一位的影响因素都是体力活动, 说明适当增加体力活动对防止向心性肥胖有积极作用。男性文化程度越高, 越易患向心性肥胖, 女性则相反, 这可能是因为男性文化程度高, 食物摄入量较多、体力活动相对较少等综合因素有关, 而女性可能文化程度越高, 越注意体形。吸烟对男性来说是向心性肥胖的保护因素, 但吸烟是一种严重影响健康的危险因素, 因此, 通过吸烟减肥不可取。

参考文献

1 中肥胖问题工作组数据汇总分析协作组. 我国成人体重指数和腰围对相关疾病危险因素异常的预测价值: 适宜体重指数和腰围切点的研究[J]. 中华流行病学杂志, 2003, 23(1): 5—10.

2 Report of a WHO Consultation. Obesity: Preventing and managing the global epidemic[R]. Technical Report Series, Geneva: WHO, 2000. 894.

3 Cameron AJ, Welborn TA, Zimmet PZ, et al. Overweight and obesity in Australia: the 1999—2000 Australian Diabetes Obesity and Lifestyle Study(AusDiab)[J]. Med J Aust, 2003, 178(9): 427—432.

收稿日期: 2004-07-26 (郭长胜编辑 孔繁学校对)

文章编号: 1001-0580(2005)03-0266-02 中图分类号: R179 文献标识码: A

【专题报道】

闵行区中小学学生单纯性肥胖干预效果评价*

孙 兰[1], 姚经建[1], 夏红[2], 屠月珍[1], 沈水仙[3], 罗飞宏[3]

摘 要: 目的 研究探索适合于上海市闵行区儿童青少年单纯性肥胖的干预方案, 为全社会开辟儿童表少年肥胖干预新途径提供参考依据。方法 组建学校卫生、心理、营养、临床多学科交叉研究小组, 从知识、行为、饮食及环境整治等多个环节, 对干预学校进行多管齐下的群体综合干预, 并对干预前后研究对象的肥胖超重度变化进行比较。结果 干预组超重学生经过 1 年干预后, 有 40.40%学生体重转为正常, 对照组为 21.845($P<0.01$); 干预组 11.25%学生体重转为肥胖, 而对照组达到 27.19%($P<0.01$)。在干预组中男女生体重下降程度差异无统计学意义。小学生与中学生比较干预效果差异无统计学意义。结论 以不校为基础的肥胖干预计划对控制闵行区儿童青少年肥胖有较显著的效果。长期坚持则需要学校领导、督导老师、家长及学生本人的配合, 小学生的肥胖干预更需要家长的支持与关心。

关键词: 肥胖; 干预; 效果评价

Appraisal of intervention effects on obesity students of primary and secondary schools in Minhang district *SUN Lan, YAO Jingjian, XIA Hong, et al. Center for Disease Control and Prevention of Minhang, Minhang Medical Health Board, Children' s Hospital of Fudan University (Shanghai 201100, China)*

Abstract: Objective To probe the effective intervention project on the pure obesity of the juvenile students in Minhang district and to provide reference to find the new intervention on the obese juvenile in China. **Methods** A multidisciplinary study group was organized including school health, psychology, nutrition and clinic. The comprehensive intervention on the school students was taken from the aspects of knowledge, practice, diet and environment improving. The degree of obesity and overweight was compared before intervention with that after intervention. **Results** After one year of intervention, there was 40.40% of the overweight students who lost weight to the normal level in the intervention group, and there was 21.84% in the control group($P<0.01$). There was 11.25% students who gained weight to the obese level, and there was27.19% in the control group($P<0.01$). There was no significant difference between the girls' weight and the boys'. The intervention effect of the primary school students was not significant and that of the secondary school students was significant. **Conclusion** The school-based intervention project for the obese juvenile of the Minhang district is effective. To carry out the project needs the cooperation of school leaders, supervisor teachers, parents and students themselves. The intervention on the primary students needs the support and regard of parents.

Key words: obesity; intervention; effect appraisal

成年人肥胖与小儿肥胖有着非常密切的关系。研究表明, 儿童期肥胖不仅有损于儿童的形态、机能及心理发育[1,2], 更可延续到成人期, 导致成人其肥胖, 而且使高血脂、高胰岛素血症、糖尿病等成年病在儿童期即提前出现[3]。本研究立足于群体预防和干预的角度, 开展以学校为基础的干预措施, 研究探索适合于上海闵行区儿童青少年肥胖儿童的有效方案, 并对其效果进行初步评价。

1 对象与方法

1.1 研究对象 整群抽取上海市闵行区经济、教学条件相似的 2 所小学一至五年级及 2 所中学预科班至初二、高一、高二的学生, 根据体检资料, 采用 WHO 推荐的身高标准体重法筛查出单纯性肥胖、超重学生, 排除因疾病等引起肥胖者, 共筛选出肥胖学生 620 名、超重学生 368 名。以其中一所中学和一所小学肥胖超重学生作为干预组, 肥胖 308 名, 超重 151 名; 另外一所中学和一所小学肥胖超重学生作为对照组, 肥胖 312 名, 超重 217 名。2 组学生年龄、性别分布差异均无统计学意

*基金项目: 上海市科技发展基金项目(区 2002—13)
作者单位: 1. 上海市闵行区疾病预防控制中心, 201100; 2. 上海市闵行区卫生局; 3. 复旦大学附属儿科医院
作者简介: 孙兰(1978—), 女, 上海人, 医师, 本科, 主要从事学校卫生工作。

?1994-2015 China Academic Journal Electronic Publishing House. All rights reserved. http://www.cnki.net

义。

1.2　入选标准　采用WHO推荐的身高标准体重法评估肥胖程度[4]。以肥胖度< 10%为正常, ≥10%为超重, ≥20%为肥胖。

1.3　干预方法　组建以学校卫生、心理、营养、临床多学科交叉研究小组, 在干预组学校开展"让肥胖远离你"为主题的以学校为基础的干预计划。干预措施包括: 根据流行病学调查结果, 具体分析每个对象的生活、饮食、行为、心理特点, 制定肥胖学生个体健康处方, 基于个体化实施干预; 对教师、厨师、学生开展课堂培训、小册子、板报、知识竞赛、义务咨询等综合知识宣传, 以达到更新知识、重新认识的目的; 制定肥胖超重学生运动方案, 建立《运动跟踪卡》, 项目包括跑步、跳绳等, 要求每次运动后心率达到120次/min, 由体育老师监督完成; 由营养师监测学生一周的饮食, 改进学生食物组成结构; 在学校内调整饮料供应结构, 以供应纯水、矿泉水为主。干预时间为1年。对照组除与干预组同期体检外不接受任何干预措施。

1.4　评价方法　采用标准方法和仪器准确测量身高和体重, 身高精确到0.1cm, 体重精确到0.1kg。早晨空腹时集体测量, 体重测量一次, 身高测量3次, 取平均值。指标测量由各学校卫生科医师专人负责。干预1年后采用同一测试方法和同一型号仪器、在同一地点和时间检测2组学生的身高和体重, 采用1995年上海市中小学生体质和健康评价标准以同一评价方法(即身高标准体重法)评估肥胖程度。对实验前后干预组和对照组学生的肥胖程度进行比较。

1.5　评价指标　干预前后肥胖学生肥胖度的差值可以反映该学生体重的变化。肥胖度增长值为负数, 说明体重减轻; 相反, 肥胖度增长值为正数, 说明体重增加。因此, 将肥胖度增长值作为考察干预效果的指标来进行分析。

1.6　统计分析　采用SPSS 11.0统计软件进行数据分析。

2　结果

2.1　干预组与对照组学生体重动态变化(表1)　由表1可见, 干预1年后干预组肥胖学生人数由308人减少为260人, 对照组则由312人增加到359人, 特别是肥胖学生转为超重干预组显著高于对照组, 超重学生转为正常者干预组显著高于对照组, 转为肥胖者干预组显著低于对照组。

表1　干预组与对照组学生体重动态变化

动态变化指标	干预组		对照组	
	肥胖 (n=308)	超重 (n=151)	肥胖 (n=312)	超重 (n=217)
干预1年后人数	260	73	359	85
转为正常	9(2.92)	61(40.40)**	4(1.28)	43(21.84)
转为超重	56(18.18)*	—	38(12.18)	—
转为肥胖	—	17(11.25)**	—	89(27.19)

注: 括号内为百分率; * P< 0.05, ** P< 0.01

2.2　干预组与对照组学生不同性别肥胖度负增长情况比较(表2)　干预组肥胖/超重肥胖度负增长男女生百分率均显著高于对照组, 但干预组男女生肥胖度负增长情况差异无统计学意义。

2.3　干预组与对照组学生不同年龄段肥胖度平均增长比较(表3)　由表3可见, 干预组肥胖/超重度负增长初中学生为75.68%和85.29%, 显著高于小学组及高中组。其中, 小学组学生肥胖/超重度负增长率与对照组比差异无统计学意义。

表2　不同性别干预组与对照组学生肥胖度负增长情况比较

性别	干预组		对照组	
	肥胖(%)	超重(%)	肥胖(%)	超重(%)
男	137(60.35)**	59(62.77)**	74(35.24)	33(27.73)
女	50(61.73)**	43(75.44)**	37(33.04)	34(34.69)

注: ** P< 0.01

表3　不同年龄干预组与对照组学生肥胖度负增长情况比较

年龄阶段	干预组		对照组	
	肥胖(%)	超重(%)	肥胖(%)	超重(%)
小学	33(41.25)	17(50.00)	49(47.57)	40(44.94)
初中	112(75.68)**	58(85.29)**	43(30.71)	41(56.16)
高中	42(52.50)	27(55.10)**	31(44.93)	8(14.55)

注: ** P< 0.01

3　讨论

从结果看, 本研究取得了一定的效果, 干预组肥胖超重学生的体重都有较显著的下降, 干预组肥胖/超重肥胖度负增长男女生百分比率均显著高于对照组, 但干预组男女生肥胖度负增长情况差异无统计学意义。国外研究表明[5], 开展以学校为基础的肥胖干预计划2学年后男生体重无变化, 女学生的肥胖有显著降低, 最主要的因素是看电视时间减少。这与本次研究结果不一致, 原因可能在于国内外教育方式的不同。由于学习负担的不断加重、活动场所设施的匮乏以及电脑的普及, 使得国内中小学生更倾向于静坐的生活方式。因此, 将本次以学校为基础的肥胖干预计划在全区推广, 防治重点放在超重学生, 将有效地控制肥胖学生的增长, 有助于全面降低闵行区中小学生肥胖患病率。

本研究结果还显示, 小学组学生肥胖/超重度负增长率较对照组差异无统计学意义, 小学生与中学生比较干预效果不显著。闵行区中小学生肥胖流行病学调查显示, 身高标准体重法评价的肥胖患病率在9岁前逐渐增加, 随后出现下降的趋势[6]。因此, 小学生应该是儿童肥胖防治工作的重点, 但由于学生家长的支持度不高, 缺少家庭的配合, 学生在学校接受肥胖干预后, 校外执行效果差, 因而影响到整个干预效果。提示, 在对父母依赖性较强的小学生进行肥胖干预时, 要更加注重学生家长的培训, 调动家长们的积极性, 取得他们的支持, 以共同完成好干预计划。

参考文献

1　丁宗一, 蒋竞雄, 许金华, 等. 肥胖儿童的有氧运动能力损伤[J]. 中华儿科杂志, 1993, 28(6): 341-343.
2　万同斌, 李重荣. 单纯性肥胖儿童自我意识水平. 社会适应能力与行为问题研究[J]. 中国心理卫生杂志, 1993, 7(1): 1-3.
3　Must A, Jacques PF, Dallal Ge, et al. Long term morbidity and mortality of overweight adolescents: A Follow-up of the 1922-35 Harvard Growth Study[J]. N Engl J Med, 1992, 327: 1350-1355.
4　上海市教育委员会, 上海市体育运动委员会, 上海市卫生局. 上海市中小学生体质和健康评价[R]. 1995, 118-123.
5　姚经建, 夏红, 屠月珍, 等. 上海市闵行区中小学生肥胖患病率调查[J]. 上海预防医学, 2004, 16(5)209-210.

收稿日期: 2004-08-23　(郭长胜编辑　赵淑艳校对)

?1994-2015 China Academic Journal Electronic Publishing House. All rights reserved.　http://www.cnki.net
