# Supplementary material for: Systematic review and meta-analysis of school-based obesity interventions in mainland China
Source: PLoS One. 2017 Sep 14;12(9):e0184704. doi: 10.1371/journal.pone.0184704 (PMC5598996; doi:10.1371/journal.pone.0184704)
Supplement: S1 Dataset — (ZIP) [file pone.0184704.s007.zip › S1_dataset/76库/60.pdf]

·临床研究·

超重、肥胖女学生有效减肥的运动及综合饮食处方 *

李 明

河南机电高等专科学校，河南省新乡市 453002
李 明，男，1962 年生，河南省开封市人，汉族，1985 年河南大学毕业，讲师，主要从事体育教育工作。

国家教育部全国教育科学"十五"规划教育部重点课题(DLA030208)*
中图分类号:R589.2 文献标识码:A 文章编号:1671-5926(2006)32-0044-03
收稿日期:2005-09-21 修回日期:2006-05-10 (05-50-9-7720/N·LL)

Anti-obesity effect of comprehensive diet and sports in girl students with simple obesity or overweight

Li Ming
Henan Mechanical and Electrical Engineering College, Xinxiang 453002, Henan Province, China
Li Ming, Lecturer, Henan Mechanical and Electrical Engineering College, Xinxiang 453002, Henan Province, China

Supported by: the Key Program of National Education Science of Ministry of Education during the Tenth Five-Year Plan, No. DLA030208*
Received: 2005-09-21 **Accepted:** 2006-05-10

Abstract
AIM: To investigate the effective anti-obesity therapy with comprehensive diet prescription and sports for girl students with simple obesity and overweight.
METHODS: The experiment was conducted between April 2004 and February 2005. Totally 120 girl students with simple obesity and overweight aged (15.5±3.7) years were selected from Xinxiang middle schools, and randomly divided into control group and test group. All the examinees or guardians were informed and agreed to join the test. Students in the test group were given the reducing weight therapy composed of aerobic exercise (body exercise, power, jump and flexible sports, body and mental healthy sports, relax sports), reasonable diet (protein: carbohydrate: fat=5:4:1), and mental modification for ten months; while the control group was just as usual. The body mass, body mass index (BMI), chest circumference, waistline, hip circumference, power of gripping, 800 meters running, standing long jump, sit-up, quiet heart rate, blood glucose, blood fat, cholesterol, insulin and leptin were measured before test (2004-03-28), during the test (2004-09-29) and after test (2005-03-01), respectively.
RESULTS: All the 120 students were involved in the result analysis. ①The mean body mass of the test group was increased 6 kg, the mean BMI was decreased from 26.74 kg/m^2 to 26.74 kg/m^2, the body mass, BMI, waist circumference and hip circumference and waistline of the test group during and after the test were obviously lower than those before the test; While those indexes of the control group had no obvious difference before, during and after the test, but were significantly higher than those of the test group at each time point ($P < 0.05$). ②The indexes of body diathesis in the test group during and after the test such as grip, 800 meters running, standing long jump, sit-up, sport ability had significant differences compared with those before the test ($P < 0.05$), so was that compared with t he control group ($P < 0.05$). ③The quiet heart rate, blood glucose, blood fat, cholesterol, insulin, leptin in the test group during and after the test were obviously lower than those before the test ($P < 0.05$); but the vital capacity were obviously higher than that before the test [(2 600±123), (2 650±218), (2 350±223) mL, $P < 0.05$]. ④Slight adverse reactions such as debility, dizzy, nausea appeared in some students during weight losing, but they did not affect normal learning and life.
CONCLUSION: The comprehensive therapy of sports combined with diet prescription for anti-obesity is rational, safe and valid; the appearance, functional, physiological and biochemical indexes of girl students with simple obesity and overweight are significantly improved, and the effect of weight losing is excellent.

Li M.Anti-obesity effect of comprehensive diet and sports in girl students with simple obesity or overweight.*Zhongguo Linchuang Kangfu* 2006;10(32):44-6(China)

李明.超重、肥胖女学生有效减肥的运动及综合饮食处方[J].中国临床康复，2006，10(32):44-6 [www.zglckf.com]

摘要
目的：探讨单纯性超重、肥胖女学生有效减肥的运动、饮食综合处方。
方法：实验于 2004-04/2005-02 进行。抽取来自新乡市普通学校单纯性肥胖女学生 120 名，年龄(15.5±3.7)岁，随机分成对照组和实验组，每组 60 名，受试者或监护人均对实验目的知情同意并配合实验。实验组采取有氧运动(包括形体运动，力量、跳跃、灵巧类运动，健身健心运动，反序运动，放松运动)、合理饮食(蛋白质:碳水化合物:脂肪为 5:4:1)、心理矫正等疗法进行为期 10 个月的减肥活动，对照组一切活动自由。在实验前 2004-03-28、实验中 2004-09-29、实验后 2005-03-01 分别检测体质量、体质量指数、胸围、腰围、臀围、握力、800 m 跑、立定跳远、仰卧起坐、安静心率、血糖、血脂、胆固醇、胰岛素和血瘦素。
结果：120 名学生均进入结果分析。①实验组实验后平均体质量较实验前减少 6 kg，平均体质量指数由原来的 26.74 kg/m^2 下降至 24.37 kg/m^2，实验中、后实验组体质量、体质量指数、胸围、腰围、臀围各指标与实验前比明显降低；而对照组上述指标在实验前、中、后比较无明显差异，且显著高于实验组相应时间点($P < 0.05$)。②实验中、后实验组身体素质指标如：握力、800 m 跑、立定跳远、仰卧起坐、运动功能能力各指标的实验中、后与实验前测试结果比较差异显著($P < 0.05$)，与对照组相应时间比较亦有显著性意义($P < 0.05$)。③实验组安静心率、血糖、血脂、胆固醇、胰岛素和血瘦素各指标的实验中、后与实验前测试结果比较明显降低($P < 0.05$)；而肺活量在实验中、后与实验前比较明显升高[(2 600±123)，(2 650±218)，(2 350±223) mL，$P < 0.05$]。④个别学生在减肥期间出现乏力、头晕、恶心等轻微不良反应，但不影响正常的学习和生活。
结论：运动、饮食综合减肥处方合理、安全、有效，超重、肥胖女学生的形态、机能、生理、生化等指标明显改善，获得了良好的减肥效果。
主题词：肥胖症；青少年；体重减轻

0 引言

超重与肥胖已成为危及人类健康的重要问题，选择一种合适的减肥方法不仅仅意味着体形的健美，也意味着生活质量的提高。因此，减肥健身方法倍受人们普遍关注。可令人担忧的是正处于青春发育期的青少年学生由于受西方文化和社会等因素的影响，一部分人为了过分追求身材美而盲目减肥，导致出现了一些不良后果[1,2]，如减肥期间出现乏力、记忆力不集中、贫血、失眠、焦虑和自卑等。为寻找一套安全可靠的减肥方法，本课题组采用有氧运动、合理营养、心理矫正和医务监督综合处方，观察青少年学生单纯性超重与肥胖减肥前后形态、功能、生理、生化指标、运动功能等变化，探讨行之有效的健康减肥措施。

1 对象和方法

设计：前后对照实验。

单位：新乡市省机电高等专科学校。

对象：实验于 2004-04/2005-02 进行。120 名受试者是来自新乡市普通学校的女中学生，均为采用体质量指数(BMI)法[3,4]筛选出的 BMI > 24 kg/m^2 的单纯性

?1994-2014 China Academic Journal Electronic Publishing House. All rights reserved. http://www.cnki.net

超重和肥胖者，并排除有内分泌与代谢疾病者。年龄（15.5±3.7）岁，经受试者或监护人同意将其随机分成两组，其中 60 名为对照组，60 名为实验组，两组年龄、体质量及 BMI 差异均无显著性意义（$P > 0.05$）。

设计、实施、评估者：设计、实施由作者和课题组其他成员共同完成，均经过培训，未采用盲法评估。

干预措施：对照组一切活动自由；实验组严格按照减肥处方执行，整个实验安排在第二课堂时间进行，共用 10 个月完成。

减肥处方的制定[5-7]：本课题组成员针对观察对象个人状况，共同讨论后制定出减肥处方。

有氧运动：活动时间为每天下午的课外活动，活动频率为每周 5~7 次，每次活动时间为 40~60 min，根据每个实验对象的个体特征，制定出不同的全面性的训练内容。①形体部分：拉伸和柔软操、垫上局部运动等。②力量、跳跃、灵巧类部分：垫上腹肌、背肌训练，哑铃坐姿上推，深蹲起、跳绳、蛇形跑等。③健身部分：快步走、慢长跑、健身操、健身舞、球类活动等。④健心部分：太极拳、体育游戏、交际舞、集体舞。⑤反序运动：倒走、倒跑、倒立。⑥放松部分：舒缓音乐中进行各种身心放松活动。

合理饮食：根据受试前每天的进食量，要求受试对象每天适当控制进食量，根据每天少摄取的热量和每天运动的最低耗热量，可以估计出每周减掉的体质量数。每天进食比例为 5:4:1，也就是蛋白质占食物净总量的 50%，碳水化合物占 40%，脂肪为 10%，并根据每个受试对象的个体特点，制定出一日三餐的饮食食谱。

行为矫正：行为矫正疗法要求改变肥胖者原来的饮食、运动以及生活习惯，使能量处于负平衡状态，并保持良好的心态，正确认识减肥问题，从而达到控制体质量的目的。

医务监测：在减肥过程中，要对学生实行严格的医务监测，及时观察她们身体对运动负荷和饮食处方的反应，定期测量她们的体质量、心率、血压等生理指标，及时调整减肥处方。

操作方法：实验组统一在下午下课后由学校或家长负责，严格按照处方要求进行运动处方的实验，合理饮食处方由家长配合完成，心理矫正等由学校和家长共同配合完成。

血样的采集和测定：清晨空腹取肘静脉血 5 mL，静置 30 min 后，以 3 000 r/min 离心 15 min，取出上层血清，-70 ℃保存。测定方法：血糖、血三酰甘油、血胆固醇的测定用全自动生化分析仪（日本 Olympus Au 400），酶法检测（单位为 mmol/L），血胰岛素和瘦素的测定用放射免疫法（单位为 IU/L 和 ng/L）。

在实验前 2004-03-28、实验中 2004-09-29、实验后 2005-03-01 分别检测各项指标，包括体质量、BMI、胸围、腰围、臀围、握力、800 m 跑、立定跳远、仰卧起坐、安静心率、血糖、血脂、胆固醇、胰岛素和血瘦素。

主要观察指标：120 名单纯肥胖女学生实验不同时间点身体形态、功能、生理和生化各指标。

统计学分析：数据由作者采用 SPSS 10.0 统计软件包进行处理，用 $\bar{x}\pm s$ 表示，计量资料差异比较采用 F 分析，t 检验。

2 结果

2.1 参与者数量分析 120 名单纯性肥胖女学生均进入结果分析，无脱落者。

2.2 实验前、中、后女学生形态指标的变化 见表 1。实验组 60 人中 10 个月后已有 9 人体质量恢复至正常，而对照组 60 人中无一人体质量恢复正常。

表 1 单纯性肥胖女中学生 120 名实验不同时间身体形态变化的比较
（$\bar{x}\pm s$，n=60）

项目	对照组		
	实验前	实验中	实验后
身高（cm）	159.46±3.57	160.46±2.12	160.34±2.32
体质量（kg）	70.37±2.24	71.04±2.38	71.46±2.11
体质量指数（kg/m^2）	26.31±1.45	26.68±2.78	26.61±3.02
胸围（cm ）	88.02±1.20	88.25±0.68	88.41±2.31
腰围（cm）	81.31±2.32	81.28±1.23	81.71±1.67
臀围（cm）	95.54±1.57	95.06±1.28	95.26±2.46

项目	实验组		
	实验前	实验中	实验后
身高（cm）	159.33±3.78	160.71±3.79	160.88±3.93
体质量（kg）	70.11±3.40	66.35±2.31^{ab}	64.14±2.31^{ab}
体质量指数（kg/m^2）	26.74±1.60	25.35±1.84^{ab}	24.37±2.21^{ab}
胸围（cm ）	88.54±1.06	85.66±0.31^{ab}	83.03±0.18^{ab}
腰围（cm）	81.47±2.32	78.04±1.34^{ab}	78.37±0.39^{ab}
臀围（cm）	95.45±1.17	92.14±1.46^{ab}	90.46±1.17^{ab}

与本组实验前比较，$^aP < 0.05$；与对照组同时间比较，$^bP < 0.05$

由表 1 可见，经过 10 个月的减肥干预实验，实验组在身体形态方面发生了很大的变化，其中平均体质量减少 6 kg，平均 BMI 由原来的 26.74 kg/m^2 下降至 24.37 kg/m^2，除身高外，体质量、BMI、胸围、腰围、臀围各指标的实验中、后与实验前测试结果比较差异有显著性意义（$P < 0.05$）；而对照组上述指标在实验前、中、后比较无明显差异，且实验组体质量、BMI、胸围、腰围、臀围各指标的实验中、后测试结果与对照组相应时间比较亦有显著性意义（$P < 0.05$）。

2.3 实验前、中、后女学生身体素质的变化 见表 2。

从表 2 可知，实验组在身体素质方面发生了很大的变化，其握力、800 m 跑、立定跳远、仰卧起坐、运动功能能力各指标的实验中、后与实验前测试结果比较差异有显著性意义（$P < 0.05$）；而对照组上述指标在实验前、中、后比较无明显差异，且实验组上述各指标的实验中、后测试结果与对照组相应时间比较亦有显著性意义（$P < 0.05$）。

2.4 实验前、中、后女学生生理、生化指标的变化 见表 3。

?1994-2014 China Academic Journal Electronic Publishing House. All rights reserved. http://www.cnki.net

表 2　单纯性肥胖女中学生 120 名实验不同时间身体素质变化的比较 ($\bar{x}\pm s$, n=60)

项目	对照组		
	实验前	实验中	实验后
握力 (kg)	32.63±3.41	33.41±3.32	33.41±2.21
800 m 跑(s)	244.39±16.32	243.54±15.63	243.64±14.33
立定跳远(cm)	161.32±14.31	161.88±12.35	162.54±13.21
仰卧起坐(个)	24.16±4.67	24.67±6.12	25.43±4.32
运动功能能力(F·c)	16.45±2.06	16.04±0.58	17.05±1.78

项目	实验组		
	实验前	实验中	实验后
握力 (kg)	33.36±3.31	35.67±2.32[ab]	36.67±2.53[ab]
800 m 跑(s)	244.45±17.03	232.12±13.53[ab]	230.47±15.64[ab]
立定跳远(cm)	161.44±13.43	171.32±15.44[ab]	175.37±13.22[ab]
仰卧起坐(个)	24.13±4.32	24.33±4.57[ab]	28.47±4.11[ab]
运动功能能力(F·c)	16.80±2.13	19.56±1.27[ab]	20.04±1.91[ab]

与本组实验前比较，$^aP < 0.05$；与对照组同时间比较，$^bP < 0.05$

表 3　单纯性肥胖女中学生 120 名实验不同时间生理、生化指标变化的比较 ($\bar{x}\pm s$, n=60)

项目	对照组		
	实验前	实验中	实验后
安静心率(次/min)	75.08±2.09	75.09±2.08	76.20±3.17
肺活量(mL)	2 350±225	2 350±257	2 400±220
血糖(mmol/L)	5.13±0.25	5.13±0.21	5.13±0.25
三酰甘油(mmol/L)	1.25±0.23	1.25±0.24	1.26±0.26
胆固醇(mmol/L)	4.48±0.31	4.48±0.37	4.49±0.38
胰岛素(IU/L)	20.13±2.89	21.5±1.36	21.01±3.14
瘦素(ng/L)	30.32±2.68	31.02±2.58	31.24±1.63

项目	实验组		
	实验前	实验中	实验后
安静心率(次/min)	75.05±2.23	73.30±2.41[ab]	72.20±2.25[ab]
肺活量(mL)	2 350±223	2 600±123[ab]	2 650±218[ab]
血糖(mmol/L)	5.06±0.30	4.65±0.23[ab]	4.60±0.12[ab]
三酰甘油(mmol/L)	1.20±0.28	0.81±0.26[ab]	0.78±0.32[ab]
胆固醇(mmol/L)	4.40±0.28	3.85±0.25[ab]	3.80±0.28[ab]
胰岛素(IU/L)	20.3±2.91	13.9±4.13[ab]	12.5±3.10[ab]
瘦素(ng/L)	31.54±9.53	21.26±3.49[ab]	18.68±2.94[ab]

与本组实验前比较，$^aP < 0.05$；与对照组同时间比较，$^bP < 0.05$

从表 3 可以看出，实验组的生理、生化指标在实验前、中、后发生了明显变化，其安静心率、血糖、血脂、胆固醇、胰岛素和血瘦素各指标的实验中、后与实验前测试结果比较明显降低($P < 0.05$)；而肺活量在实验中、后与实验前比较明显升高；对照组上述指标在实验前、中、后比较无明显差异，且实验组上述各指标的实验中、后测试结果与对照组相应时间比较亦有显著性意义($P < 0.05$)。

2.5　不良事件和副反应　个别学生在减肥期间出现乏力、头晕、恶心等轻微不良反应，但不影响正常的学习和生活。

3　讨论

单纯性肥胖症是多种因素混合作用的结果，单纯性肥胖的发病机制主要与以下因素有关[8,9]：①生活方式：包括膳食方面高热量、高脂肪饮食、进食次数及摄取激素、生长素的等，缺乏体力活动，工作和生活中越来越广泛地应用节省体力的设备。②社会因素：有城市化、移民心身问题、独生子女等问题。③某些药物，如抗精神病药、糖皮质激素等可使体质量增加。目前肥胖病已成为全球常见的一种慢性疾病，而中国青少年的超重和肥胖呈逐年上升趋势。众多研究表明：肥胖青少年约 80%将继续发展为肥胖成人，成人期发生的冠状动脉粥样硬化性心脏病、高血压、糖尿病等均与肥胖有密切关系。因此选择一种有效而不损害健康的减肥方法不仅仅意味着体形的健美，也意味着生活质量的提高。有关研究显示，从降低体质量结果看采用极低热量饮食只能维持 6~10 个月，加上心理矫正可持续一两年，再加上有氧运动可持续 1~6 年。因此课题组采用有氧运动、合理营养、心理矫正和医务监督综合处方。

运用不同有氧运动+合理饮食+心理矫正+医务监督综合减肥处方进行 6 个月的实验后，青少年女学生单纯性肥胖患者体质量明显降低，同时其形体发育指标、身体素质指标、生理、生化指标与减肥前比较有明显改善。10 个月后实验组已有 9 人体质量恢复正常，而对照组中无 1 人体质量恢复正常。同时增强了心血管系统功能，提高了身体素质，改变了体形和心理健康状况，有效降低单纯性超重与肥胖者的血糖、血脂、血胆固醇、胰岛素和瘦素等水平，减轻和消除导致动脉粥样硬化、高血压、冠状动脉粥样硬化性心脏病、2 型糖尿病等疾病的一些潜在于青少年中的危险因素，使他们能够更好地健康成长。这一数据及时地为扭转爱美心切的青少年女学生单纯性肥胖患者盲目减肥提供了客观有效的依据；同时为预防一些慢性疾病的发生、减少因肥胖而支出的医疗费用提供了保障；为今后正确引导其减肥行为、有针对性地开展营养教育及进行减肥健身方法指导提供了科学依据。

以往单一的减肥方法在减肥的过程中，出现的不良反应率较高，副作用大，许多学生坚持不下来，且影响他们的学习和生活，而本实验综合减肥首先使减肥者有一个正确的认识、正确对待，始终保持良好的心态，再置于医务监督之下，然后运用以有氧运动+合理营养为主的综合措施，只有这样减肥才会安全可靠、持久有效。因此，对单纯性超重与肥胖青少年学生及其全社会广大青少年开展健康教育，使他们积极从事体育锻炼，饮食上提倡合理营养与平衡膳食，正确认识和对待减肥这个漫长过程，有效地控制体质量增长，从而达到减肥健身、健美健心的目的。

4　参考文献

1　覃羽乔,徐永芳,梁桂民,等.超重和肥胖与高血压、糖尿病的关系[J].中国公共卫生,2004,20(6):656-7
2　王立勇. 简论减肥的几大误区与合理减肥[J]. 西华大学学报：哲学社会科学版,2004,23(S1):140-2
3　季成叶,孙军玲.中国学龄青少年体质量指数地域与人群分布差异的分析[J],中华儿科杂志,2004,42(5):328-32
4　中国肥胖问题工作组数据汇总分析协作组. 我国成人体质量指数和腰围对相关疾病危险因素异常的预测价值：适合体质量指数和腰围切点的研究[J].中华流行病学杂志,2002,23(1):5-10
5　彭莉.几种不同减肥方法及其效果评价[J].体育学报,2001,8(2):44-5
6　张佑琏.单纯性肥胖的运动处方[J].中国临床康复,2002,6(7):938-9
7　傅兰英,姬英涛,姬成茂.运动处方对女大学生减肥及健康状况影响研究[J].中国学校卫生,2004,10(25):539-40
8　黄小民. 肥胖的成因、危害及减肥手段[J]. 湖北体育科技,2000,4(4):57-62
9　李松,邹旭,邓铁涛.肥胖机制及中医药防治肥胖症的研究进展[J].中西医结合心脑血管病杂志,2004,2(11):657-9

?1994-2014 China Academic Journal Electronic Publishing House. All rights reserved.　http://www.cnki.net
