# Supplementary material for: Systematic review and meta-analysis of school-based obesity interventions in mainland China
Source: PLoS One. 2017 Sep 14;12(9):e0184704. doi: 10.1371/journal.pone.0184704 (PMC5598996; doi:10.1371/journal.pone.0184704)
Supplement: S1 Dataset — (ZIP) [file pone.0184704.s007.zip › S1_dataset/76库/62.pdf]

【健康教育】

平衡膳食与运动干预单纯性肥胖初中生效果评价

邢良美[1],林祥田[2],王文军[3],张超[4],孙成凯[1],冯伟军[1],赵洁[1],王晓青[1],朱丽[1]

1.江苏省连云港市海宁中学,222000;2.连云港市卫生监督所;3.连云港市第二人民医院;4.连云港市新海中学

【摘要】 目的 探讨平衡膳食与运动干预对单纯性肥胖初中生身体的影响,为制定有效干预措施提供依据。方法 选取连云港市 2 所中学肥胖初中生 48 名,随机均分为对照组、极限有氧运动组、平衡膳食组、平衡膳食加极限有氧运动组 4 组。测试体质量指数(BMI)、血液部分指标、皮脂厚度、围度。结果 干预后,平衡膳食极限有氧运动组与对照组在 BMI (27.82±2.52,30.68±2.56) kg/m^2、腰围(90.38±9.22,101.38±5.58) cm、上臂围(29.54±2.39,31.70±5.24) cm,差异均有统计学意义(P 值均<0.01),且优于其他两组;极限有氧运动组三酰甘油与对照组比较显著降低,且优于其他两组(P 值均<0.01)。结论 平衡膳食极限有氧运动对肥胖中学生 BMI、皮脂厚度、围度的影响具有协同效应。极限有氧运动可以显著降低三酰甘油含量。

【关键词】 饮食习惯;肥胖症;运动;干预性研究;学生

【中图分类号】 G 479 R 151.4 【文献标识码】 A 【文章编号】 1000-9817(2015)01-0051-03

Effects of balanced diet and exercise on simple obesity among middle school students/*XING Liang-mei**, *LIN Xiang-tian*, *WANG Wen-jun*, *et al*.* *Haining Middle School*, *Lianyungang*(222000), *Jiangsu Province*, *China*

【**Abstract**】 **Objective** To explore effects of balanced diet and exercise on simple obesity among middle school students in Lianyungang City. **Methods** A total of 48 obese boys and girls were randomly divided into four groups: control group, extreme aerobic exercise group, balanced diet group, balanced diet & extreme aerobic exercise group. At the end of intervention, BMI, blood variables, thickness, circumference were measured and calculated. **Results** After intervention, BMI, thickness, waist and upper arm´s circumference(27.82±2.52,90.38±9.22,29.54±2.39) improved significantly compared with control group(30.68±2.56, 101.38±5.58,31.70±5.24). Triglyceride(TG) decreased remarkably in extreme aerobic exercise group. **Conclusion** Combination of balanced diet and extreme aerobic exercise maximize the effects of obesity intervention among middle school students. Triglyceride could be improved through extreme aerobic exercise.

【**Key words**】 Food habits; Obesity; Exercise; Intervention studies; Students

目前,青少年肥胖不但在发达国家泛滥成灾,而且呈全球流行趋势[1]。本试验拟通过对连云港市区单纯性肥胖初中生日常生活方式的调整,在不影响学习、生长发育的基础上,使其健康减肥、快乐成长。

1 对象与方法

1.1 *对象* 选择连云港海宁中学和新海实验中学肥胖中学生 90 名,经过医学筛选,排除内分泌与代谢疾病,以体质量指数(BMI)≥28 kg/m^2 为肥胖筛选标准[2-3],筛选出 48 名单纯性肥胖者(男女生各 24 名)。在自愿参与试验的前提下,随机分成对照组、极限有氧运动组、平衡膳食组、平衡膳食极限有氧运动组,每组均为 12 名(男女生均各 6 名)。年龄在 13~14 岁之间。

1.2 *方法*

1.2.1 *干预方案* 对照组:不做任何干预。平衡膳食组:早餐 1 个鸡蛋,馒头或菜包不超过 2 个,稀饭 1 碗,蔬菜 1 份;中餐米饭 1 碗,鱼(除鱿鱼和带鱼)或虾不超过 100 g,高纤维的蔬菜 1 份(芹菜、荠菜、青菜等),不喝汤;晚餐馒头不超过 2 个,稀饭 1 碗,蔬菜 1 份;睡前 250 mL 牛奶。每天饮水不少于 2 000 mL,平时不喝含糖饮料、不吃甜点和油炸食品;女生每周中餐可有 1 次排骨,不超过 150 g。所有学生均在家用餐,家长根据既定食谱烹制食物并填写 1 周学生饮食表,每周一饮食表交与教师检查。极限有氧运动组:侧重于有氧运动,最大摄氧量 60%~70%,每周 3 次,由教师统一安排训练,每次 15 min 匀速跑,35 min 间歇跑;每天 3 组仰卧起坐和 3 组立卧撑,仰卧起坐每组 30 个,立卧撑每组 10 个(可以在家做),饮食不做干预。平衡膳食极限有氧运动组:在运动的同时给予饮食调整,方案同极限有氧运动组和平衡膳食组。

1.2.2 *干预时间安排* 第 1 次指标测试时间为 2011 年 11 月 4—5 日,第 2 次指标测试时间为 2012 年 5 月 25—26 日。膳食和运动干预从 2011 年 11 月 20 日至

【基金项目】 江苏省营养学会资助项目(JYX201109)。

【作者简介】 邢良美(1973-),女,江苏连云港人,硕士,教师,主要研究方向为运动人体科学。

?1994-2015 China Academic Journal Electronic Publishing House. All rights reserved. http://www.cnki.net

2012 年 5 月 20 日,共 6 个月。

1.2.3　指标测定　身高、体重、围度、皮脂厚度由经过训练的专业人员测试[4]。血样的采集和测定:采血前 1 天素食,清晨空腹取肘静脉血 5 mL,放置 37 ℃水浴箱静置 30 min 后,以 3 500 r/min 离心 10 min,取出上层血清;血胆固醇(TC)、血三酰甘油(TG)、高密度脂蛋白(HDL)、低密度脂蛋白(LDL)用全自动生化分析仪(DADE-RXL)测定。

1.3　统计分析　试验结果用平均值±标准差($\bar{x}\pm s$)表示,组间差异用 SPSS 13.0 进行方差检验,以 $P<0.05$ 为差异有统计学意义。

2　结果

2.1　不同干预方案对肥胖初中生 BMI 和围度的影响　各组干预前各项目均无差异(P 值均>0.05)。干预后,平衡膳食极限有氧运动组 BMI 为(27.82±2.52) kg/m^2、平衡膳食组(31.16±2.28) kg/m^2、极限有氧运动组(29.08±1.20) kg/m^2、对照组为(30.68±2.56) kg/m^2。平衡膳食极限有氧运动组 BMI 与对照组差异有统计学意义($P<0.01$);单纯膳食、运动组与对照组比较,差异均无统计学意义(P 值均>0.05)。平衡膳食极限有氧运动组、极限有氧运动组上臂围、腰围与对照组差异均有统计学意义($P<0.01$);平衡膳食组上臂围、腰围、大腿围与对照组差异均无统计学意义(P 值均>0.05)。平衡膳食极限有氧运动组与单纯膳食组比较,BMI、上臂围、腰围变化差异有统计学意义(P 值均<0.05)。见表 1。

表 1　不同组别肥胖中学生 BMI 和围度干预前后比较($\bar{x}\pm s$)

组别	干预前后	人数	BMI/($kg \cdot m^{-2}$)	上臂围/cm	腰围/cm	大腿围/cm
对照组	干预前	12	31.00±2.46	35.42±5.20	102.50±4.72	59.75±5.77
	干预后	12	30.68±2.56	31.70±5.24	101.38±5.58	59.00±5.94
平衡膳食极限	干预前	12	31.03±2.33	34.92±3.40	101.67±4.91	63.17±4.45
有氧运动	干预后	12	27.82±2.52**#	29.54±2.39**#	90.38±9.22**##	58.00±5.15
平衡膳食	干预前	12	31.24±2.21	34.04±2.24	103.83±6.45	60.00±4.79
	干预后	12	31.16±2.28	32.83±2.82	100.00±9.47	57.08±5.23
极限有氧运动	干预前	12	30.62±1.38	34.38±1.52	100.46±5.57	59.75±2.86
	干预后	12	29.08±1.20	30.33±1.07**	93.00±5.61*	59.50±2.39

注:干预后与对照组相比,* $P<0.05$,** $P<0.01$;与平衡膳食组相比,# $P<0.05$,## $P<0.01$。

表 2　不同组别肥胖初中生皮脂厚度干预前后比较($\bar{x}\pm s$,mm)

组别	干预前后	人数	背部	上臂	腹部
对照组	干预前	12	43.00±8.37	39.50±7.03	55.00±6.08
	干预后	12	43.08±8.33	39.50±7.33	50.33±6.87
平衡膳食极限	干预前	12	44.17±6.32	41.25±6.36	53.58±5.25
有氧运动	干预后	12	31.83±6.58**△△##	25.83±4.13**△△##	37.42±8.72**△△#
平衡膳食	干预前	12	40.33±4.01	38.42±6.01	54.50±5.84
	干预后	12	40.25±4.14	38.33±6.10	53.00±5.61
极限有氧运动	干预前	12	40.83±6.29	38.92±4.64	55.17±7.08
	干预后	12	45.33±6.23	37.58±3.73	46.67±8.24△

注:干预后与对照组相比,** $P<0.01$;与平衡膳食组相比,△ $P<0.05$,△△ $P<0.01$;与极限有氧运动组相比,# $P<0.05$,## $P<0.01$。

2.2　不同干预方案对肥胖初中生皮脂厚度的影响　由表 2 可见,干预后,平衡膳食极限有氧运动组与极限有氧运动组、平衡膳食组与对照组比较,背部、上臂、腹部皮脂厚度差异均有统计学意义(P 值<0.01)。

2.3　不同干预方案对肥胖中学生血液部分指标的影响　与对照组比较,平衡膳食极限有氧运动组三酰甘油含量差异有统计学意义($P<0.05$);平衡膳食组 4 个指标差异均无统计学意义;极限有氧运动组三酰甘油差异有统计学意义($P<0.05$),净下降率为 61.0%,总胆固醇、低密度脂蛋白、高密度脂蛋白差异均无统计学意义(P 值均>0.05)。见表 3。

表 3　不同组别肥胖初中生血液部分指标干预前后比较($\bar{x}\pm s$,$mmol \cdot L^{-1}$)

组别	干预前后	人数	总胆固醇	三酰甘油	高密度脂蛋白	低密度脂蛋白
对照组	干预前	12	4.08±0.86	1.30±0.48	1.23±0.31	2.69±0.58
	干预后	12	4.10±0.89	1.27±0.31	1.22±0.18	2.68±0.74
平衡膳食极限	干预前	12	4.05±0.86	1.30±0.41	1.17±0.16	2.75±0.95
有氧运动	干预后	12	3.66±0.84	0.82±0.48*	1.21±0.15	2.27±0.71
平衡膳食	干预前	12	4.06±0.76	1.28±0.33	1.18±0.22	2.72±0.59
	干预后	12	3.92±1.09	0.92±0.57	1.23±0.22	2.51±0.84
极限有氧运动	干预前	12	4.02±0.91	1.37±0.90	1.17±0.11	2.62±0.68
	干预后	12	3.63±0.83	0.76±0.38**	1.20±0.18	2.21±0.59

注:干预后与对照组相比,* $P<0.05$,** $P<0.01$。

?1994-2015 China Academic Journal Electronic Publishing House. All rights reserved.　http://www.cnki.net

3 讨论

肥胖在中国学生群体中已经广泛流行,且城市高于乡村[5-6]。BMI 是判断人体体型的常用指标,BMI 高低反映人体的胖瘦程度。本试验结果显示,平衡膳食极限有氧运动组试验后 BMI 与对照组比较差异有统计学意义,但其他干预组与对照组比较差异均无统计学意义。与 Frey-Hewitt 等[7]报道结果相似。可能是平衡膳食控制了多余能量的摄入,极限有氧运动既消耗了脂肪又改善了骨的营养,三者共同作用导致了 BMI 降低。提示平衡膳食极限有氧运动对 BMI 的影响优于单纯平衡膳食组和运动组。

本研究的结果显示,对腰围和上臂围的影响大小依次为平衡膳食极限有氧运动组、极限有氧运动组、平衡膳食组; 对大腿围的影响平衡膳食极限有氧运动组>平衡膳食组>极限有氧运动组。与 Frey-Hewitt 等[7]研究结果一致。即运动减肥的能量支出只有在摄入量不增加的情况下,才会累积能量损失,否则运动时摄入能量增加则减重作用不大。

平衡膳食极限有氧运动组肩胛下角、上臂、腹部皮脂厚度干预后分别下降 27.9%,37.4%,30.5%; 极限有氧运动组上臂、腹部皮脂厚度下降 3.4%,15.4%; 平衡膳食组的肩胛下角、上臂、腹部皮脂厚度净下降率分别为 0.2%,0.2%,2.8%; 极限有氧运动组肩胛下角皮脂厚度增加 11.0%。

皮脂为储存于皮下的脂肪组织,人体的脂肪大约有 2/3 储存在皮下。外国学者研究发现运动虽不能减少脂肪细胞数目,但可以抑制脂肪细胞的积累,减小脂肪细胞体积[8]。极限有氧运动一方面使机体减轻了胰岛素拮抗,减少了血糖进入脂肪细胞的量,抑制了脂肪的合成; 另一方面增加了能量消耗又降低了摄食效率,从而减少了体脂沉积,导致脂肪细胞体积缩小。另外,平衡膳食则从源头上控制了能量的摄入。三者共同作用于机体,导致皮脂厚度下降。单纯运动组的肩胛下角皮脂厚度略微增长是否与立卧撑有关,具体原因还有待探讨。

干预后平衡膳食极限有氧运动组和单纯运动组的三酰甘油水平较干预前都显著降低,分别下降了 36.9%,61.0%,单纯平衡膳食组三酰甘油的含量也下降了 28.1%,与付娟等[9]研究结果类似。说明运动干预和饮食调节对机体三酰甘油的含量都有影响,而极限有氧运动优于其他两组。

与对照组比较,平衡膳食极限有氧运动组、平衡膳食组、极限有氧运动组总胆固醇、高密度脂蛋白、低密度脂蛋白含量差异均无统计学意义。可能与试验时间长短、营养成分的搭配、运动强度等有关,具体原因还有待进一步探讨。

4 参考文献

[1] 季成叶.全球学龄儿童青少年超重与肥胖的流行现状和趋势[J].中国学校卫生,2006,27(8):648-650.
[2] 中国肥胖问题工作组.中国学龄儿童青少年超重、肥胖筛查体重指数值分类标准[J].中华流行病学杂志,2004,25(2):97-102.
[3] 季成叶.儿童肥胖筛查方法研究的最新进展[J].中国学校卫生,2006,27(4):279-280.
[4] 李洁,陈仁伟.人体运动能力检测与评定[M].北京:人民体育出版社,2005:4.
[5] 季成叶.我国中小学生超重肥胖流行现状及其社会经济差异[J].中国学校卫生,2008,29(2):106-108.
[6] 刘晓静.乌鲁木齐市汉族学龄儿童青少年超重肥胖现状[J].中国学校卫生,2012,33(2):210.
[7] FREY-HEWITT B.The effect of weight loss by dieting or exercise on resting metabolic rate in overweight men[J].Int J Obes,1990,14(4):327.
[8] HEDLEY AA,OGDEN CL,JOHNSON CL,et al.Prevalence of overweigt and obesity among US children,adolescents and adults,1999-2002[J].JAMA,2004,291(23):2847-2850.
[9] 付娟,肖明,乔静.健康管理对代谢综合症患者血液指标的影响[J].武警医学院学报,2010,19(11):859-860.

收稿日期:2014-05-07;修回日期:2014-07-07

(上接第 50 页)

[11] TANDON PS, ZHOU C, SALLIS JF, et al. Home environment relationships with children's physical activity, sedentary time, and screen time by socioeconomic status[J].Int J Behav Nutr Phys Act, 2012, 9:88.
[12] DUMITH SC, HALLAL PC, MENEZES AM, et al. Sedentary behavior in adolescents: The 11-year follow-up of the 1993 Pelotas (Brazil) birth cohort study[J].Cad Saude Publica, 2010, 26(10): 1928-1936.
[13] COOMBS N, SHELTON N, ROWLANDS A, et al. Children's and adolescents' sedentary behaviour in relation to socioeconomic position[J].J Epidemiol Comm Health, 2013, 67(10): 868-874.
[14] PULSFORD RM, GRIEW P, PAGE AS, et al. Socioeconomic position and childhood sedentary time: Evidence from the PEACH project[J].Int J Behav Nutr Phys Act, 2013,10: 105.
[15] GUEDES DP, SOUZA MV, FERREIRINHA JE, et al. Physical activity and determinants of sedentary behavior in Brazilian adolescents from an underdeveloped region[J].Percept Mot Skills, 2012, 114(2): 542-552.
[16] HALLAL PC, WELLS JC, REICHERT FF, et al. Early determinants of physical activity in adolescence: Prospective birth cohort study[J].BMJ, 2006, 332(7548): 1002-1007.
[17] PATRIARCA A, DI GIUSEPPE G, ALBANO L, et al. Use of television, videogames, and computer among children and adolescents in Italy[J].BMC Public Health, 2009, 9: 139.
[18] FAIRCLOUGH SJ, BODDY LM, HACKETT AF, et al. Associations between children's socioeconomic status, weight status, and sex, with screen-based sedentary behaviours and sport participation[J].Int J Pediatr Obes, 2009, 4(4): 299-305.

收稿日期:2014-06-11;修回日期:2014-07-12

?1994-2015 China Academic Journal Electronic Publishing House. All rights reserved. http://www.cnki.net
