# Supplementary material for: Systematic review and meta-analysis of school-based obesity interventions in mainland China
Source: PLoS One. 2017 Sep 14;12(9):e0184704. doi: 10.1371/journal.pone.0184704 (PMC5598996; doi:10.1371/journal.pone.0184704)
Supplement: S1 Dataset — (ZIP) [file pone.0184704.s007.zip › S1_dataset/76库/63.pdf]

开设运动处方，改善肥胖小学生体质的实证研究

厉利华 (浙江省杭州市外语实验小学，310014)

摘 要：以本校部分肥胖学生(BMI≥28)为实验对象，制定、实施运动干预措施，通过运动处方、自觉训练、监督指导的运动干预方式对肥胖学生进行16周的实验研究，以身体形态、生理机能和身体素质等为评价指标，探讨运动处方对肥胖学生体质的影响。结果表明：以有氧运动为主的运动处方干预可使肥胖学生的身体形态发生明显变化，身体素质、心肺功能得到明显提高。建议这些学生长期坚持以有氧为主的体育锻炼，达到增强体质的目的。

关键词：运动处方；肥胖；小学生；BMI；体质

本研究通过对本校部分BMI指数偏高学生以运动处方的形式进行运动干预，旨在揭示运动处方对改善肥胖学生体质的积极影响，为增强肥胖学生身体健康状况提供参考。

一、确定研究对象

对我校2012年体质测查数据进行分析，发现有67名肥胖学生（体重指数≥28）。经过调查了解，其中3名学生有先天性生理疾病，因此，本实验选择本校另外64名肥胖学生为调查对象，年龄7~12岁之间，排除心血管疾病等重大躯体疾病及其他慢性躯体疾病，随机分为对照组和实验组各32人(见表1)。

表1 受试学生的BMI指数范围(n=64)

BMI指数	28.0-29.0	30.1-32.0	≧32.1
实验组人数	14	7	11
对照组人数	13	7	12

BMI指数，简称体质指数，又称体重指数（body mass index,BMI[体重/身高2(kg/m2)]），是反映人体体重与身高关系，且与身体成分密切相关的、判断人体胖瘦程度和评价营养状况的常用指标。本试验中以中国肥胖问题工作组制定的中国学龄儿童少年超重肥胖BMI分类标准(简称WGOC标准）筛选各年龄组超重、肥胖，体重指数(BMI)≥28为肥胖。

二、运动处方干预方案的设计与实施

1.设计运动处方

按照易于开展、便于监督与指导的原则制定运动处方，处方中的项目是根据学生自己的兴趣、爱好选择，在运动量和运动强度得到保证的前提下进行（见表2）。

表2 运动干预对改善肥胖小学生体质实验的运动处方

类别	内 容
目的	减轻或控制体重，保持和增强身体运动能力
内容	选择以耐力运动或有氧运动为主的项目：<br>长跑、球类、游泳、跳短绳（长绳）、健身操等
时间	每周练习5~6次，每次40~60min
强度	适宜心率是最大心率的60%~80%。最大心率=220-年龄。<br>一般控制在120-140次/min
饮食	根据学生家庭实际情况进行搭配，运动干预期间，<br>以新鲜蔬菜、蛋类、牛奶等素食为主，同时控制食量

2.方案的实施

实验的目的：论证运用运动处方干预的方式改善肥胖小学生体质的有效方法，为解决肥胖小学生形体、生理机能和身体素质供有效的方法指导和依据。

周期与时间：实验周期约为1个学期，共4个月，实验组学生在校期间除每周参加学校安排的正常的体育课教学外，其余时间每天根据本实验给与的运动处方练习，时间不少于60分钟(周六、日由家长陪同练习并填写运动记录卡)。对照组学生按照学校常规的体育教学和大课间活动进行锻炼。

组织形式：根据自愿报名与教师推荐，32名学生分别加入到本校各个社团，依托本校足球队、体训队、健美操队和长绳队、篮球及羽毛球兴趣小组开展训练，由各社团负责的教师进行指导并监督，并填写训练记录卡，最后统一保存，以便于监督。布置一定的体育家庭作业，家庭作业以单跳绳、仰卧起坐、原地蹲跳起、踢毽子等易于开展的项目为主。训练的时间主要放在晨练、大课间、阳光体育锻炼时以及下午放学后，尽量与各社团活动时间统一。

家校联合：改善肥胖学生的体质状况是一个系统工程，小学生自我控制能力比较弱，因此需要学校和家庭相互配合，共同参与指导与监督，才能达到良好的效果。本实验借助校讯通、学生家校联系本、家长会等平台，加强与肥胖学生家长的沟通联系，取得其家长的支持与配合，让家长监督学生完成体育家庭作业，并及时记录、反馈，学生试验期间家长合理搭配饮食，控制食量。实验中按照尊重、保密和教育性原则进行，依据小学生生理、心理特点，注重训练的趣味性、实效性，让参加试验的学生享受到运动的愉悦和保持自觉训练的积极性。

三、实验后指标测试

测试指标主要有：身高、体重、安静心率、肺活量、400米跑、50米跑、立定跳远和仰卧起坐。实验前、后分别测试以上各类指标，并将测试数据建立数据库，对数据进行组内和组间对比分析。指标测试方法采用中小学生学生体质健康标准测试方法。

四、试验结果

1. 运动处方干预实验对肥胖学生身体形态方面的影响

根据实验的数据统计，实验组和对照组学生的体重和BMI指数有明显的区别(见表3)。

表3中显示，经过16周的运动干预试验，实验组的肥胖学生体重从51.92kg下降至47.26kg，BMI指数从31.12下降至28.92。实验组体重和BMIt值<0.05，说明有显著性差异；对照组体重和BMI指数t值>0.05，

?1994-2015 China Academic Journal Electronic Publishing House. All rights reserved. http://www.cnki.net

表3 实验前后体重和BMI指数比较（$\bar{x}\pm s$）

指数	体 重		BMI指数	
实验前	实验组：51.92±8.66		实验组：31.12±4.81	
	对照组：49.97±6.49		对照组：31.54±3.58	
时间	16周	t值	16周	t值
试验后	实验组47.26±11.52	<0.05	实验组28.92±4.5	<0.05
	对照组49.19±7.32	>0.05	对照组31.98±3.89	>0.05

没有显著性差异，说明没有科学训练的指导、方法和手段，对照组学生的体重控制和改善BMI指数的效果不理想。以上数据说明，运用运动处方干预的手段的对小学肥胖学生的体重和BMI指数进行干预，具有非常明显的效果，同时也是科学减脂的方法之一。

2.实验组肥胖学生生理机能指标的变化情况分析

肺活量能综合反映人体肺部和胸部的弹性以及呼吸肌的发达程度。肺活量大小与体育锻炼有密切联系，肺活量越大，说明人体呼吸系统的最大工作能力越强。有关资料表明，长期参加有氧锻炼可以使心肌纤维增粗，心肌收缩力增强，心输出量增加，提高供血能力。台阶试验是测量心血管系统机能水平的简单方法。它是在定量负荷下，按照持续运动的时间和运动后心率恢复速度的比例关系，来评价心血管系统对运动负荷的反应。由表4可以看出，对照组安静心率、肺活量、台阶试验指数实验前后无显著变化；实验组安静心率P<0.05，有显著差异；肺活量P<0.01、台阶试验指数P<0.01，说明实验前后有非常显著差异。实验组学生肺活量增大、安静心率下降、台阶指数显著提高，说明以有氧为主的体育锻炼对改善机体的生理机能确有积极的影响。因此，长期坚持以有氧为主的体育锻炼不仅能提高肥胖小学生的最大吸氧量，还能改善肥胖小学生机体运输、储备及利用氧的能力，有效提高机体有氧耐力。

表4 实验组和对照组实验前后生理机能指标比较（$\bar{x}\pm s$）

组别	实验前后	安静心率(次/min)	肺活量/ml	台阶试验指数
实验组	实验前	81.86±4.95	2228.1±560.99	45.08±12.07
	试验后	76.29±4.67 *	2437.9±615.2 **	53.79±11.56 **
对照组	实验前	80.79±3.86	2258.6±571.3	45.11±13.14
	试验后	81.02±5.24	2269.2±598.4	47.38±13.25

注：实验前后比较，t检验，*P<0.05，**P<0.01

3.肥胖学生身体素质各指标变化情况分析

表5显示，对照组在试验前后身体素质指标无显著变化，实验组实验前后以及实验组与对照组相比各指标均有显著性差异(P<0.05)。从实验结果还可看出，通过16周时间的运动干预，学生的耐力、速度、弹跳、力量素质提高比较明显。说明经过16周的运动处方干预，肥胖学生的各项身体素质均得到了增强。同时也提醒了我们在组织学校的晨间锻炼、大课间活动及阳光体育锻炼时要关注学生个体间的差异性，不同学生群体间的活动内容、运动量、运动强度要区别对待，使活动安排更科学合理，让每一个学生都得到相应的锻炼。

表5 实验组和对照组实验前后身体素质指标比较（x±s）

组别	实验前后	400米跑（s）	立定跳远（厘米）	仰卧起坐（个）	50米跑（s）
实验组	实验前	108.36 ±21.26	166.21 ±16.56	37.03 ±8.97	10.1 ±0.92
	试验后	97.13 ±20.06*	174.51 ±11.43*	41.86 ±7.17*	9.6 ±0.76*
对照组	实验前	109.44 ±22.15	161.9 ±11.42	34.28 ±8.74	10.25 ±0.90
	试验后	107.17 ±24.5	162.61 ±12.37	36.28 ±7.92	10.12 ±0.94

注：实验前后比较，t检验，*表示P<0.05

五、结论与建议

1.通过运动处方干预可使肥胖学生的身体形态发生明显变化，身体素质、心肺功能得到明显提高。

2.对肥胖学生实施运动处方干预的内容要以具有一定运动强度、频度和持续时间的有氧运动为主，也要考虑小学生的特点及对运动项目的兴趣、喜好，让学生乐于参与运动，保证锻炼效果。同时，由于小学生自控能力差，学校及家长要加强沟通，共同关注肥胖学生的体质健康，引导和督促肥胖学生坚持体育锻炼，养成良好的运动习惯和饮食习惯。

3.运动处方干预中应注意运动的时间、运动量和运动强度。运动干预的具体实施措施要科学、合理，符合小学生生理、心理发展的特点，锻炼方式要灵活丰富，趣味性强，这样才能达到较理想的效果。运动中将学生心率控制在合理范围内，适宜心率是最大心率的60%~80%，小学生一般控制在120~140次/min，运动时间每次不得少于40min。

4.运用运动处方干预的方式改善肥胖学生体质，要遵守循序渐进原则，对学生要求要由低到高，逐步增加运动量。尊重学生的个体差异，制定有针对性的目标，让学生在实现目标的过程中体验到成就感；教师要以鼓励、表扬为主，使学生真正了解自己的进步，激发学生内心的运动兴趣，树立他们持之以恒的锻炼观念。

5.运动干预的同时，配合适当的饮食控制，养成良好的生活习惯；运动后适当补充水分及无机盐，以弥补由于运动大量排汗引起的部分水分及无机盐的流失。

参考文献：

[1]董晓虹.大学男生BMI的分型与身体机能和素质的差异性比较研究[J].中国体育科技,2006,2.

[2]邓树勋,王健,乔德才.运动生理学[M].北京:高等教育出版社,1999:403–426.

[3]王维群,郭红,邓树勋等.有氧健身操运动处方对中年女科教@ABC[J].天津体育学院学报，2001,2:49– 52.

[4]国家体育总局学生体质健康标准研究课题组.学生体质健康标准（试行方案）[M].北京：人民教育出版社，2002:156.

[5]于素梅.肥胖与有氧运动减肥的生物学分析（综述)[J].北京体育大学学报,2001,1.

[6]严小波.大学新生不同BMI等级心脏储备能力的比较——以重庆大学为例[J].成都体育学院学报,2010,1.

[7]张成云.运动干预对改善高职院校肥胖学生BMI指数的实验与效果分析[J].广州体育学院学报,2009,1.

[8]中国肥胖问题工作组.中国学龄儿童超胖.中华流行病学杂志,2004,2.

?1994-2015 China Academic Journal Electronic Publishing House. All rights reserved. http://www.cnki.net
