# Supplementary material for: Systematic review and meta-analysis of school-based obesity interventions in mainland China
Source: PLoS One. 2017 Sep 14;12(9):e0184704. doi: 10.1371/journal.pone.0184704 (PMC5598996; doi:10.1371/journal.pone.0184704)
Supplement: S1 Dataset — (ZIP) [file pone.0184704.s007.zip › S1_dataset/76库/64.pdf]

【健康教育】

肥胖儿童生活环境控制和心理干预效果评价

陶佩生[1], 霍金芝[2], 徐济达[3], 冯向明[4], 钱晓勤[1], 张亮才[5], 蔡亮亮[6]

【摘要】 目的 探讨肥胖儿童生活环境控制和心理干预方法, 为及早控制肥胖儿童的体重提供科学依据。方法 随机抽取扬州市2所学校, 在1个学年期内, 建立可操作性的“学校—家庭—社区”、“三级预防”、“知—信—行”3个三位一体的健康教育实践模式, 实施生活环境控制和心理干预的综合措施。结果 干预后, 儿童、家长健康知识、态度和行为都有明显提高($P<0.01$); 肥胖儿童BMI和血压都降低($P<0.01$), 饮食行为有所改善, 社会心理功能、生理心理健康、生活环境、总体评价均高于干预前($P<0.01$), 生活质量呈上升趋势($P<0.01$)。结论 以家庭、学校和社区为平台, 通过对肥胖儿童生活环境控制和心理干预的综合措施, 可有效降低肥胖的发生。

【关键词】 生活方式; 精神卫生; 干预性研究; 结果评价(卫生保健); 儿童; 肥胖症

【中图分类号】 G 479 R 179 R 589.2 【文献标识码】 A 【文章编号】 1000-9817(2006)11-0983-03

全球儿童肥胖日渐增多并呈逐年增长的趋势。有研究表明, 各年龄组儿童体重均以0.2 kg/a的幅度增长[1]。儿童青少年的超重和肥胖不仅关系到他们当前的健康和心理发育, 而且影响其成年后的健康和社会功能及生活质量, 是一个关系到生命过程的公共卫生问题[2]。为此, 及早控制儿童期超重和肥胖是保护儿童身心健康和预防成年期疾病的重要措施之一。笔者在对扬州市中小学生肥胖流行情况及其影响因素的调查和分析的基础上, 进行了1学年的健康干预, 旨在探讨肥胖儿童生活环境控制和心理干预方法, 现将结果报道如下。

1 对象与方法

1.1 对象 整群抽取扬州市2所小学1 036名儿童及其家长为研究对象, 其中1所学校506名儿童及其家长为干预对象, 另1所学校530名儿童及其家长作为对照。

1.2 方法

1.2.1 体格测量 身高、体重和血压测量采用常规测量方法[3]。

1.2.2 肥胖、超重判定标准 按照国际生命科学学会中国肥胖工作组推荐的中国学生超重、肥胖BMI筛查标准进行评价[4]。

1.2.3 健康知识态度行为问卷调查 采用自行设计, 经专家审核的知识、态度、行为(KAP)调查问卷, 主要内容包括膳食与营养、生活方式与疾病、科学运动和自我监测。

1.2.4 儿童少年生活质量问卷 由华中科技大学同济医学院公共卫生学院儿少卫生教研室编制, 从自我满意度、师生关系、躯体感受、同伴关系、亲子关系、运动能力、学习能力及态度、自我概念、负性情绪、作业态度、活动机会性、生活便利性、其他共13个维度评定儿童的生理、心理、社会能力及生活环境等方面的生活状态。

1.2.5 健康干预 以家庭、学校和社区为平台, 在对群体儿童预防的基础上, 加强对肥胖儿童的指导, 在1个学年期内进行健康干预。学校开设健康教育课, 培养卫生骨干, 采用child-to-child方法传授卫生知识, 儿童通过调查、广播、办板报、发宣传资料、举办营养知识竞赛等形式, 在学校内开展儿童互动式宣传和干预。针对肥胖儿童不良生活习惯[5-6], 学校召开肥胖儿童及其家长会, 学校校长动员, 课题研究人员讲解“肥胖的危害及其干预知识”, 让儿童家长自身建立健康生活方式, 并督导儿童合理膳食、科学运动和保持心理健康, 同时鼓励肥胖儿童参与自身健康促进, 要求肥胖儿童多吃高纤维食物, 限制高能量食物的摄入, 减慢进食速度, 保证足够的蛋白质、维生素、矿物质和微量元素的摄入。除了上好体育课外, 每周至少3次, 每次0.5~1 h低到中强度的体力活动; 进行24 h膳食回顾和测身高、体重、血压等。通过认知过程, 纠正肥胖儿童不良饮食习惯和增加有氧运动。

在社区组织儿童家长开设“膳食营养”、“生活方式与健康”和“慢性病防治”等知识讲座, 为儿童及其家长提供“肥胖儿童膳食、心理、运动指导处方”、“中国居民膳食指南和膳食宝塔”和“慢性非传染性疾病防制”等健康教育资料。由电台、电视台、报纸等宣传媒体共同参与, 形成良好的生活环境和控制肥胖的氛围。对照组不进行任何健康干预。

1.2.6 统计方法 应用SPSS 10.0统计软件进行资料的处理和分析。

2 结果

2.1 干预前后儿童健康知识、态度、行为得分情况 干预前, 干预组与对照组儿童各项得分差异无统计学意义($P>0.05$)。干预后, 干预组各项得分均高于对照组($P<0.01$); 对照组各项得分差异无统计学意义($P>0.05$); 干预后干预组各项得分均高于干预前($P<0.01$)。见表1。

2.2 儿童与家长健康知识、态度、行为的改变情况 干预后儿童健康知识答对率由干预前的62.2%上升到96.0%; 干预后儿童家长健康知识答对率由干预前的42.0%上升到96.1%; 干预后儿童与家长健康知识都高于相应的对照组($P<0.01$)。儿童及家长回答正确率高于90%的有5项, 依次为健康的愿望与需求(儿童98.2%, 家长98.4%), 早餐的重要性和肥胖对健康的影响(儿童98.7%, 家长95.4%), 中国居民膳食指南内容(儿童知晓率97.83%, 家长知晓率97.0%), 蛋白质、铁、钙的食物来源(儿童与家长均为92.0%), 每天运动时间(儿童与家长分别为90.8%和92.1%)。

干预后儿童与干预前、对照组饮食行为相比, 每天吃杂粮、薯类、蔬菜、水果、豆制品的人数增多($P<0.01$), 吃甜食、油炸食物人数和进餐速度人数明显下降($P<0.01$)。见表2。

【基金项目】 江苏省预防医学科研项目基金资助(y2002023)

【作者简介】 陶佩生(1953—), 女, 江苏扬州人, 大学本科, 主任医师, 主要研究方向为儿童生长发育与慢性非传染性疾病控制。

【作者单位】 1 江苏省扬州市疾病预防控制中心, 225001; 2 苏州大学放射医学与公共卫生学院; 3 南京医科大学; 4 江苏省卫生监督所; 5 仪征市疾病预防控制中心; 6 扬州市梅岭小学。

?1994-2015 China Academic Journal Electronic Publishing House. All rights reserved. http://www.cnki.net

表1 干预组和对照组儿童干预前后健康知识、态度、行为得分比较($\bar{x}\pm s$)

组别	人数	时间	知识	态度	行为	总得分
干预组	506	干预前	61.55±12.60	20.57±4.61	56.51±9.41	138.65±17.41
		干预后	98.04±1.64*	25.92±2.16*	61.73±15.34*	185.69±15.77*
对照组	530	干预前	62.84±13.42	20.84±4.67	56.90±8.90	140.58±17.84
		干预后	63.31±14.39	20.75±4.42	56.69±11.16	140.75±18.80

注:*干预前后比较以及与对照组比较,$P<0.01$。

表2 干预组和对照组儿童干预前后饮食行为比较 n/%

组别	人数	时间	杂粮	薯类	蔬菜	水果	奶类	豆制品	动物性食物	甜食	油炸食品	进餐速度
干预组	506	干预前	2.4	3.2	82.2	71.5	82.2	70.4	96.0	58.9	44.7	77.5
		干预后	28.5**	27.7**	98.0**	96.0**	84.2	88.1**	98.0	25.3**	20.2**	39.9**
对照组	530	干预前	3.0	3.4	83.4	70.9	79.6	79.6	96.6	57.7	43.4	70.8
		1学年后	2.6	4.5	84.5	71.3	76.2	78.9	97.4	55.8	41.5	75.1**

注:**干预组前后比较以及与对照组比较,$P<0.01$。

2.3 干预前后肥胖率变化情况 通过1个学年的综合控制措施,干预组儿童的肥胖率由9.3%下降为6.9%($P<0.01$),而对照组则由9.1%上升为10.2%。

2.4 肥胖儿童干预前后体格指标、生活质量比较 从表3可见,肥胖儿童干预前BMI值、血压及胆固醇摄入量都高于对照组正常体重儿童,干预后BMI值、收缩压低于干预前($P<0.01$),胆固醇摄入量减少($P<0.01$)。干预前、后肥胖儿童社会心理功能、生理心理健康、生活环境、总体评价均低于对照组($P<0.01$),干预后肥胖儿童社会心理功能、生理心理健康、生活环境、总体评价均高于干预前($P<0.01$),干预后肥胖儿童生活质量呈上升趋势($P<0.01$)。

表3 肥胖儿童干预前后体格指标、胆固醇摄入量、生活质量比较($\bar{x}\pm s$)

组别	人数	时间	体重指数/($kg\cdot m^{-2}$)	收缩压/mm Hg	舒张压/mm Hg	胆固醇/mg
对照组	48	干预前	15.61±1.06	100.30±6.34	62.75±6.38	621.01±288.14
干预组	47	干预前	21.73±2.45	116.89±6.61	69.89±3.21	905.27±616.82
		干预后	20.82±2.22**	109.11±6.62**	65.64±4.50**	725.52±579.76**

组别	人数	时间	社会心理功能	生理心理健康	生活环境	总体评分
对照组	48	干预前	66.56±7.36	35.71±5.60	24.27±5.06	153.15±17.89
干预组	47	干预前	60.57±7.15	31.43±5.05	21.40±3.42	135.83±12.15
		干预后	64.72±7.45**	34.00±4.53**	23.09±3.51**	146.09±13.12**

注:干预组前后比较以及与对照组比较,**$P<0.01$。

3 讨论

3.1 儿童肥胖状况 随着社会经济的发展,生活水平的提高,人们膳食结构改变和体力活动减少,全球儿童肥胖以惊人的速度增长,美国1999—2000年学龄儿童和青少年超重率分别为15.3%和15.5%,较20世纪80年代初上升了30%~80%[2]。我国2000年肥胖检出率城男、城女、乡男、乡女分别达8.1%,4.1%,3.4%和2.3%;与10 a前相比,肥胖检出率分别增长2.8,3.2,5.8和4.5倍[5],正迅速接近发达国家水平(10%)。扬州市2000年儿童少年肥胖率为5.6%,2002年上升到9.4%[6]。肥胖不仅影响儿童当前身心健康,而且导致高血压、糖尿病、冠心病、脑卒中等成年期疾病的发生呈明显低龄化趋势。目前多数学者认为,肥胖发生率快速增长的主要原因是生活环境转变所致[7-9],因此改变肥胖儿童不良饮食行为,建立健康的生活环境和生活方式是预防肥胖的关键。

3.2 肥胖儿童生活环境控制 近年来,国内也进行了许多干预研究,多以肥胖儿童个体的治疗和指导为中心,或者是仅仅依赖学校宣传教育控制肥胖,并不能收到理想的效果。本课题以健康管理角度为切入点,系统地把3个三位一体有机地结合:"学校—家庭—社区"三位一体、"三级预防"三位一体、"知—信—行"三位一体。通过各种可利用社区资源,可操作性的方式和手段,达到环境控制、心理认知、行为培养与改变的有机结合。在整个过程中,疾病预防控制中心是使3个三位一体达到有效结合并运行的核心。由于肥胖发生原因的多重性,学校—家庭—社区防疫圈中的关系是松散的,任何一个环节都不能独立完成这项综合性的防治工作。所以,通过疾病预防控制中心进行计划管理与沟通,并充分应用卫生、临床和科研部门力量,使这一疾病控制模式形成并非常成功。

3.3 肥胖儿童干预结果 调查结果显示,肥胖儿童具有许多有别于正常儿童的心理行为特点,如低自律性、社会适应力差、交往不良、社会退缩、攻击性等,这与有关报道一致[7-9],干预前肥胖儿童社会心理功能、生理心理健康、生活环境、总体评价均低于对照组($P<0.01$)。这些心理行为对控制肥胖的发生发展有重要作用。通过健康教育,让肥胖儿童认识到肥胖的病因和危害性,认识到减肥的必要性,从而产生减肥的欲望和动力。98.72%的肥胖儿童愿意在获得有关知识的前提下作出健康的行为改变。干预前许多肥胖的儿童家长认为自己孩子体重正常并鼓励孩子吃多吃好。干预后,95.36%的家长认知发生变化,认识到儿童肥胖对健康的危害和经济负担,并愿意改变不良的生活方式。这为健康干预提供了有利条件。

肥胖儿童的控制需要全社会共同努力,尤其需要有社区和学校长期的支持环境。既要有家长作积极的表率和密切配合,也要有学校与社会的综合教育与管理。及时、及早地控制肥胖儿童,可避免像发达国家那样因肥胖流行而导致大量的资源浪费和巨大的健康代价。

(下转第986页)

?1994-2015 China Academic Journal Electronic Publishing House. All rights reserved. http://www.cnki.net

乡村中小学教师的个人教学效能感得分存在显著的性别差异,女教师高于男教师;城市中小学教师的个人教学效能感存在显著的教龄之间的差异,教龄为11~20 a的教师高于10 a以下和20 a以上组;乡村教师不同教龄组之间的一般教学效能感差异存在统计学意义,教龄20 a以上的教师低于10 a以下和11~20 a组。无论是城市还是乡村,个人教学效能感均存在职称之间差异,初级职称的教师低于中级职称和高级职称教师。

2.2 城乡中小学教师的抑郁状况分析 结果显示,肯定有抑郁症状的中小学教师占20.5%,其中小学教师占19.7%,中学教师占21.1%;城市教师占21.5%,乡村教师占19.7%。

由表1可见,抑郁得分城市和乡村教师差异无统计学意义。城市中小学教师的抑郁水平差异有统计学意义,小学教师高于中学教师;农村中小学教师的抑郁水平差异无统计学意义。城乡中小学教师各学历之间差异有统计学意义,大专以下学历的教师高于大专和本科学历的教师;城乡中小学教师抑郁得分职称间差异有统计学意义,中级职称的中小学教师高于初级和高级职称的中小学教师。乡村中小学教师抑郁得分性别差异有统计学意义,男教师高于女教师;城市中小学教师的抑郁得分不存在性别差异。城乡中小学教师抑郁得分各教龄组之间差异无统计学意义。

2.3 中小学教师的教学效能感和抑郁的相互关系 教学效能感和抑郁的相关分析表明,个人教学效能感、一般教学效能感和抑郁均存在显著负相关,相关系数分别为-0.266,-0.289,显著性水平均在0.01以上。提示中小学教师的教学效能感越低,抑郁程度越大。3 讨论

3.1 城乡中小学教师的教学效能感 城乡中小学教师一般教学效能感差异有统计学意义,乡村教师高于城市教师,这和吴国来等[4]的研究一致。原因可能是,农村父母受教育水平相对较低,用于教育子女的时间相对较少,对子女的期望相对较低,认为孩子的教育是学校的事,也很少主动配合教师的教育,这会让乡村教师感到学生的成绩、成长主要受学校教育和教师的影响;而城市教师更注重学生家庭环境和家庭教育的重要性,城市学生家长也更能主动配合教师的教育,加之城市教师对影响学生成绩因素的复杂性认识程度较高,导致城市教师的一般教学效能感相对较低。

个人教学效能感和一般教学效能感小学教师和中学教师差异有统计学意义,均为小学教师高于中学教师。小学生比较容易服从教师的管教;初中生由于成人感的产生,逆反心理较强,对教师的权威作用提出挑战,给中学教师的教育教学增加难度,从而导致中学教师的教育教学自信心的下降。

3.2 城乡中小学教师的抑郁状况 调查结果显示,中小学教师有抑郁症状者较多,占20.5%,高于李作佳等[5]的调查结果。知识的日新月异,基础教育改革的不断深入,聘岗、学生升学等同行竞争的不断加剧,都给中小学教师造成很大的压力。较大的压力会导致抑郁,从而使有抑郁症状的中小学教师比例升高。城乡中小学教师抑郁得分不同学历之间差异有统计学意义,学历越低,抑郁水平越高。学历低的教师在竞争中处于劣势,容易产生较多的心理紧张和抑郁。抑郁得分各职称之间差异显著,中级职称的中小学教师得分较高,高级职称的中学教师得分较低。已取得高级职称的初中教师不再承受评聘职称的压力,教学经验丰富,教学能力、业务水平相对较高,教学效能感较强,从而压力较轻,抑郁得分较低。中级职称的中小学教师在学校中往往承担着较重的教育教学任务,进取心更强,成就感更高,因而承受的压力更大,抑郁得分更高。

3.3 教学效能感和抑郁的相互关系 调查表明,中小学教师的教学效能感越低,抑郁程度越大;反之亦然。Chwalisz等[6]的研究发现,低教学效能感的教师比高教学效能感的教师拥有更高水平的职业倦怠。刘晓明[7]的研究表明,教师的教学效能感越低,情绪衰竭和人格解体的程度也将越严重。教师教学效能感作为教师在教学活动中完成教学工作、实现教学目标能力的知觉和信念,其教学效能感水平必然影响对教学活动的选择、教学行为成败的归因以及对情绪的调控。低教学效能感的教师在面对挫折时,比较倾向于采取逃避的策略,会因学生的不良纪律等而引起较大程度的焦虑,从而表现出更高程度的抑郁。

4 参考文献

[1] BANDURA A. Self-efficacy: Toward a unifying theory of behavioral change. Psychol Rev, 1977, 84(2): 191-215.

[2] 俞国良,辛涛,申继亮. 教师教学效能感:结构与影响因素的研究. 心理学报, 1995, 27(2): 159-166.

[3] 汪向东,王希林,马弘. 心理卫生评定量表手册: 中国心理卫生杂志增订版. 北京: 中国心理卫生杂志社, 1999: 200-202.

[4] 吴国来,白学军,沈德立. 中学教师教学效能感影响因素的研究. 天津师范大学学报: 基础教育版, 2003, 4(4): 16-20.

[5] 李作佳,周秋华,于振华. 中小学教师生活事件、应对方式与焦虑、抑郁的相关性研究. 中国临床心理学杂志, 2003, 11(4): 285-286.

[6] CHWALISZ K, ALTMAIER EM, RUSSELL DW. Causal attributions, self efficacy cognitions, and coping with stress. J Soc & Clin Psych, 1992, 11(4): 377-400.

[7] 刘晓明. 职业压力、教学效能感与中小学教师职业倦怠的关系. 心理发展与教育, 2004, 2: 56-61.

(收稿日期: 2005-12-29; 修回日期: 2006-03-10)

(上接第984页)

4 参考文献

[1] FREEMAN DS, SRINIRASAN SR, VALDEZ RA, et al. Secular increases in relative weight and adiposity among children over two decades: The Bogalusa Heart Study. Pediatrics, 1997, 99: 420-426.

[2] 陈春明. 对儿童肥胖问题的防治不可坐失良机. 中华流行病学, 2004, 25(2): 95-96.

[3] 季成叶,刘宝林,主编. 儿童少年卫生学. 5版. 北京: 人民卫生出版社, 2003: 201-202.

[4] 季成叶. 中国学生超重肥胖BMI筛查标准的应用. 中国学校卫生, 2004, 25(1): 125-128.

[5] 季成叶. 中国青少年生长与营养状况变化和改善策略. 北京大学学报: 医学版, 2002, 34(5): 525-529.

[6] 陶佩生,霍金芝. 扬州市青少年体重质量状况调查. 中国校医, 2005, 19(6).

[7] 霍金芝,陶佩生. 超重学生的行为和脑力工作能力特征. 中国校医, 2005, 19(1): 4-5.

[8] 陶佩生,霍金芝,陈柱之,等. 扬州市不同体重学生饮食行为和家庭相关因素分析. 中国校医, 2005, 19(5): 481-482.

[9] 孙扣红,霍金芝,赵晓宾. 单纯性肥胖与儿童心理行为的关系. 中国校医, 2003, 15(4): 318-319.

(收稿日期: 2006-08-18)

?1994-2015 China Academic Journal Electronic Publishing House. All rights reserved. http://www.cnki.net
