# Supplementary material for: Systematic review and meta-analysis of school-based obesity interventions in mainland China
Source: PLoS One. 2017 Sep 14;12(9):e0184704. doi: 10.1371/journal.pone.0184704 (PMC5598996; doi:10.1371/journal.pone.0184704)
Supplement: S1 Dataset — (ZIP) [file pone.0184704.s007.zip › S1_dataset/76库/65.pdf]

综合行为干预治疗学生单纯性肥胖的效果观察

李 平 李 刚

【摘要】 目的 探讨青春期单纯性肥胖生减肥的有效方法。方法 在 12～16岁的学生中筛选出单纯性肥胖生 80例,从健康教育行为指导、饮食控制、体育锻炼等方面着手,对实验组肥胖生采用学校、家长、个人有机配合的综合行为干预措施进行减肥。结果 经过两年干预,他们的肥胖程度显著下降($P<0.01$),身高增长值与正常对照组无差异仍符合生长发育一般规律。SBP、DBP、TC、TG体脂百分数 F%也有一定程度下降。结论 综合行为干预治疗学生单纯性肥胖具有一定效果。

【关键词】 单纯性肥胖 行为干预 青春期学生

Effects of comprehensive behavior intervention in curing the pure obesity of the juvenile students *Li Ping, Li Gang. Health and Antiepidemic Station Zaozhuang City, Zao Zhuang, Shandong Province* 277101

【**Abstract**】 **Objective** To probe the effective way to help the overweight juvenile students reduce. **Methods** Selecting 80 overweight students in the age of 12～16 as subjects and curing them of their obesity by the comprehensive intervention from active cooperation among the school, family and the students themselves from the following aspects: health education, guided behavior, diet control and physical exercises et al. **Results** After two years of such comprehensive intervention, there is an obvious reduction in their weight($P<0.01$) and the percentage of physical fat (F%), SBP, DBP, TC and TG in their body also decreased correspondinly. The rate of the growth in height has no difference compared with those students as control. **Conclusions** It is comparatively effective to use the comprehensive intervention of the pure obesity of juvenile students.

【**Key words**】 Pure obesity Behavior intervention Juvenile student

我们在参阅有关肥胖方面大量文献资料基础上[1～4],于 1993年试采取综合行为疗法对青春期单纯性肥胖者进行试验。经过两年的治疗取得一定疗效,现将有关情况报道如下。

对象与方法

一、对象 选择枣庄市市直中学三所共 4084名中学生作为调查对象,年龄跨度为 12～16岁。共检出肥胖生 122人,肥胖检出率为 2.99%。通过有关化验项目,剔除病理性肥胖生,最后共筛选取用单纯性肥胖生 80例。根据自愿原则,将肥胖生分为施以综合行为干预的肥胖干预组 38例及非干预的肥胖对照组 42例,并同时选择同一学校相应年龄段的同性别正常体重学生 40名作为非干预的正常对照组。三组学生男女之比分别为 27∶11、27∶15、29∶11。肥胖学生肥胖度轻、中、重比例为 11∶18∶9和 11∶19∶12,三组学生的年龄 $\bar{x}\pm s$(岁)分别为 14±1.5、14±1.3、14±1.5。将当年体检调查资料以“中国学生 7～22岁身高标准体重值”表为评价学生肥胖的参考标准进行筛选。肥胖程度分类依据为:标准体重 20%为轻度肥胖;>标准体重 30%为中度肥胖;>标准体重 50%为重度肥胖。

测试时间:于 1993年 9月进行第一次测试,经过两年多的治疗于 96年 5月进行了第二次测试。

二、方法

1.检测细则严格按专人专项原则测量身高、体重与皮褶厚度,建立 Dbase数据库,以 SPSS软件包进行统计分析。皮褶厚度采用国家体委科研所生产的皮褶厚度计进行测查。通过所测皮褶厚度值代入下列回归方程,计算各年龄段个体体脂百分含量。

	男 生	女 生
10～12岁	F% = 9.0870+0.6616X	F% = 11.2657+0.5311X
13～15岁	F% = 4.8942+0.5496X	F% = 10.8048+0.3614X
16～18岁	F% = 3.6836+0.4097X	F% = 8.4724+0.4249X

(X:肱三头肌及肩胛下角部皮褶厚度之和;F%:体脂百分含量)

脉搏、血压的测量按照全国学生体质健康状况调研检测细则进行。生化检验取清晨空腹静脉血,用生化酶法测定血清总胆固醇(TC)、甘油三酯(TG)。

2.行为指导 成立减肥领导小组,由市教委、卫生防疫站、校医、家长代表组成。通过体格检查、问卷调查、病史询问等方式摸清基础情况,取得家长的合作、信任与积极参与。建立肥胖生个人减肥档案,针对每个肥胖生分析其肥胖原因,制订减肥计划,设计减肥方案,确定减肥措施和目标,制定出家长、学生分别应遵守的规则。以市卫生防疫站为指导中心,行学校、家庭两级管理,对孩子饮食、运动和生活方式进行监督检查指导。建立定期反馈制度,召开激励会,释解减肥过程中出现的问题,尤其是心理问题。不断向孩子讲明减肥的长期性特点,帮助其培养信心、恒心、毅力、执著等个性品质,激发对体育锻炼的浓厚兴趣乃至渴求心理,变“要我做”为“我要做”,及时用已取得的效果巩固和进一步增强减肥的自觉

* 作者单位:277101 山东省枣庄市卫生防疫站

?1994-2015 China Academic Journal Electronic Publishing House. All rights reserved. http://www.cnki.net

性。

三、饮食疗法　配合定期反馈制度，开办肥胖生及家长的减肥学习班，主要向他们介绍健康的概念；儿童生长发育一般规律；饮食与肥胖的关系；致肥胖的危险因素；肥胖与疾病的关系；减肥的原理。重点讲述合理平衡膳食的重要意义：三大营养物质作用；人体每日热卡生理需要量及其计量方法，学会计算摄入食物的含热量，印发日常食物的热卡量表。介绍减肥食品、低热量食谱；养成良好家庭个人饮食习惯意义并结合行为指导促使其养成科学饮食习惯。健康教育无论在行为指导、饮食疗法抑或是体育锻炼上，帮助他们很好完成了从“所以然”到“之所以然”的理解过程，道理的通晓为工作顺利开展奠定了良好基础。具体工作由家长根据培训班上学到的内容安排孩子的一天饮食，填写食物种类、量，定期反馈。

四、体育锻炼　根据参加减肥生的肥胖程度、本人可以耐受的减肥速度，分阶段、循序渐进地提高运动量，要求每人每天至少进行 30~ 60分钟的全身性有氧运动。运动方式灵活多样，取决于肥胖生体育锻炼项目的偏好，可为散步、跳绳、长跑、游泳、健身操、太极拳、剑、武术及各种球类运动。运动强度要求为最大心率的 65% ，运动后即刻测试脉搏应为 110次 /分左右。针对干预组的每个肥胖生做好日常活动安排，一并将各项运动热量消耗表介绍给他们，要求自行掌握。凡参加干预组的肥胖生假期均参加市组织的减肥训练班进行集中锻炼。

结　果

一、干预组肥胖度下降。从测试结果看，肥胖干预组干预后较干预前下降了 25.33% ，差异显著。而肥胖对照组无明显变化（$P > 0.05$），同时干预组体重呈缓慢增长趋势。肥胖干预组、肥胖对照组和正常对照组三组身高增长值接近（$P > 0.05$，见附表）。

二、血压、血脂、体脂率改变。由表 1可见，肥胖干预组学生的血压、总胆固醇、甘油三酯、体脂率均有不同程度的下降，差异显著（$P < 0.01$）。肥胖对照组的学生上述各指标无明显变化（$P > 0.05$）。干预后两组值比较均有显著性差异。

附表　干预前后肥胖生血压、血脂和体脂含量的变化（$\bar{x} \pm s$）

项目	肥胖干预组 $n = 38$			肥胖对照组 $n = 42$			P^*
	第一次测试	第二次测试	P	干预前	干预后	P	
收缩压（Kpa）	16.4± 1.5	14.9± 1.4	<0.01	16.7± 1.8	16.3± 1.9	> 0.05	<0.01
舒张压（Kpa）	11.2± 1.4	9.6± 1.4	<0.01	11.1± 1.5	11.3± 1.6	> 0.05	<0.01
总胆固醇（mmol/l）	4.7± 0.6	4.1± 0.5	<0.01	4.6± 0.8	4.6± 0.7	> 0.05	<0.01
甘油三酯（mmol/l	1.8± 0.2	1.5± 0.3	<0.01	1.7± 0.3	1.8± 0.4	> 0.05	<0.01
体脂百分含量（%）	34.5± 8.6	26.7± 7.8	<0.01	36.8± 8.6	35.7± 9.7	> 0.05	<0.01

P为组内比较　　P^* 为干预后组间比较

三、行为矫正　干预组肥胖生有关肥胖所及知、信、行三方面均有了不同程度地提高。卫生保健知识增加，不良饮食习惯有了改善，改掉了懒惰行为，情绪心理状况良好，体育成绩也相应有了提高。而肥胖对照组以上各项变化均不显著，未有一定规律可循。

讨　论

实践证明，参加治疗的肥胖生在经过两年的综合行为干预后，肥胖程度明显下降，体重增长呈缓慢增长状态，显示该试验对进一步控制肥胖增加的效果显著，因而起到了明显的减肥作用。三组身高增长不存在差异，增长速度符合一般规律，减肥并未对肥胖生身高的生长产生不良影响。同时，血压、血脂等指标也明显降低，体脂率下降，减少了肥胖所致心血管疾病发生的危险因素。可以肯定地说，综合行为干预在治疗青春期单纯性肥胖方面不失为一种安全、有效、切实可行的方法。若在中学生中推广，可起到预防和治疗肥胖的双重作用。不仅如此，其重要意义还在于通过行为干预，可给学生带来多方面的教育。随健康知识的增加，卫生保健意识增强，学生变得有能力选择科学的生活方式，而摒弃诸如饮食过量、睡前进食、喜甜食、油炸油腻食物，爱吃零食等饮食行为上的不良习惯，改变懒惰行为，从喜静不动到喜欢户外活动、爱做家务、渐渐地爱上体育锻炼，不仅增强体质还有利于克服自卑心理，减轻心理压力，增强自信心，保持良好情绪状态，精力充沛等。在减肥过程中，能够为学生带来积极心态、希望和信念，使兴趣、毅力、坚强乐观等个性品质得到充分培养，从学会关心自己到别人，从自我意识增强到参与意识的浓厚，从理论知识到指导实践活动方法的掌握，这一切或许比单纯达到减肥目的更有意义。我们建议对于青春期单纯性肥胖的治疗不妨从最基本的健康教育、行为指导、饮食控制、体育锻炼等方面入手，因势利导、因地制宜地采取综合干预措施，就此所涉及的

?1994-2015 China Academic Journal Electronic Publishing House. All rights reserved.　http://www.cnki.net

许多有关问题有待于更深入的研究。

参考文献

1 张立芳、丁玉荣、杨冬梅.通县中小学生肥胖调查分析.中国学校卫生, 1997, 18(4): 246~ 248.
2 吕姿之.小学生单纯性肥胖原因初探(摘要).中华预防医学杂志, 1990, 24(6): 365~ 366.
3 徐剑峰.肥胖儿童的肥胖因素研究.中华预防医学杂志, 1990, 24(3): 146~ 148.
4 孙茂森.儿童肥胖对机体机能素质发育的影响.中国公共卫生, 1994, 10(2): 539~ 541.

(收稿日期: 1998- 07- 20 编辑、校对:崔立谦)

儿童抽动—秽语综合征神经心理测查报告

胡云清 杨晓玉 孟庆云

抽动—秽语综合征(TS)的病因及发病机理尚不清楚,为了从神经心理角度来探讨 TS的发病机理,我们对 21例 TS患儿进行心理测查,报告如下:

对象与方法

一、对象:测试组 21例 TS患儿,男 14例,女 7例,年龄 8~ 14岁;对照组 20例,为健康学龄期儿童,男 10例,女 10例,年龄 7~ 14岁。

二、方法:智力测验采用林传鼎等修订的韦氏儿童智力量表(WISC- CR);个性测验采用龚耀先等修订的儿童艾森克个性问卷(EPQ);行为测验采用上海精神卫生中心修订的 Achenbach儿童行为量表(CBCL)。结果采用 t检验进行统计学分析。

结 果

一、智力测查结果:对照组及 TS组将各分测验得分累加得粗分,将分测验粗分转换为量表分,进一步将量表分相加后查表可得总智商 FIQ、言语智商 VIQ和操作智商 PIQ及各分测验量表分,两组分别比较,FIQ、VIQ、PIQ差异无显著意义,但分测验中迷津、译码、背数分数显著低于对照组,见表 1。

表 1 TS组与对照组智力测查结果($\bar{x}\pm s$)

项目	TS组	对照组
类同	10.8± 0.7	11.2± 0.6
算术	7.9± 0.7	8.5± 0.5
理解	9.3± 0.3	9.5± 0.4
常识	8.9± 0.8	9.1± 0.2
背数	9.2± 0.4*	12.1± 0.6
填图	10.2± 0.3	9.9± 0.5
积木	10.4± 0.6	10.9± 0.8
译码	8.2± 0.4*	10.9± 0.5
迷津	9.1± 0.3*	11.9± 0.1
VIQ	94.2± 1.1	99.8± 1.9
PIQ	100.8± 2.2	103.4± 2.0
FIQ	92.9± 2.0	101.7± 1.5

* $P < 0.05$与对照组比较

二、个性测查结果:TS患儿精神质、神经质量表 T分显著高于对照组,而内外向性和掩饰性低于对照组。见表 2

三、行为测查结果:TS患儿 CBCL总分(38.7± 4.2)明显高于正常儿(20.3± 1.8),其中 5个行为因子得分显著高于对照组($P < 0.05$)。

表 2 TS组和对照组 EPQ个性测验结果

项目分数	TS组	对照组
精神质量表 T分	50.1± 1.6*	40.2± 1.5
神经质量表 T分	52.2± 1.9*	46.3± 1.7
内外向性量表 T分	45.5± 2.1*	56.6± 2.3
掩饰性量表 T分	46.2± 2.2*	55.1± 1.3

* $P < 0.05$与对照组比较

讨 论

国外有关 TS神经心理的研究认为 TS神经心理功能存在不同程度的缺陷[1]。我们的结果表明 TS患儿的智力水平正常,但分测验中迷津、译码、背数显著降低,提示 TS智力结构中的记忆不分心因子存在一定的缺陷,译码异常也提示 TS的非结构性视觉实践能力存在缺陷,这种非结构性视觉实践能力障碍被认为是原发性皮层损害或基底节神经功能失调的继发性结果[2]。背数可以反映注意能力,而 TS约半数左右伴有注意缺陷多动障碍(ADHD)[3],故推测 TS的背数异常可能与其伴随的 ADHD行为问题有一定的关系。译码异常提示 TS短时记忆存在缺陷。EPQ量表反映情绪稳定性,故 TS的神经质量表 T分增高说明患儿情绪不稳定,易激惹对外界各种刺激反应比较强烈。TS的精神质量表分增高说明患儿比较孤僻,适应外界环境能力差,喜欢攻击及不怕危险,TS的掩饰性 T分低说明患儿心理成熟延迟。本研究 TS的 CBCL总分明显高于正常,提示行为异常。

综上所述,TS患儿存在某些神经心理缺陷,故其发病是生物因素与心理因素共同结果,从中指导我们在药物治疗同时从心理上给予适当的干预。

参考文献

1 刘智胜,林庆,抽动—秽语综合征的神经心理研究进展.国外医学儿科学分册, 1994, 21(3): 116~ 120.
2 Bornstein RA, King G, Carroll A. Neuropyschological abnormalities in Gilles de la Tourette′s syndrone. J NervMent dis, 1983, 171: 497~ 498.
3 刘智胜,林庆. Tourette综合征.国外医学精神病分册, 1994, 21(2): 99~ 102.

(收稿日期: 1999- 01- 22 编辑、校对:林 立)

作者单位: 154002 黑龙江省佳木斯大学附属一院

?1994-2015 China Academic Journal Electronic Publishing House. All rights reserved. http://www.cnki.net
