# Supplementary material for: Systematic review and meta-analysis of school-based obesity interventions in mainland China
Source: PLoS One. 2017 Sep 14;12(9):e0184704. doi: 10.1371/journal.pone.0184704 (PMC5598996; doi:10.1371/journal.pone.0184704)
Supplement: S1 Dataset — (ZIP) [file pone.0184704.s007.zip › S1_dataset/76库/66.pdf]

· 儿童保健 ·

有氧运动综合干预措施对肥胖儿童血瘦素和血脂成分的影响①

许胜利　河南省新乡医学院　453003

中国图书分类号　R179　　文献标识码　B　　文章编号　1001-4411（2007）21-2935-03

【摘　要】　目的：观察有氧运动综合干预措施对单纯性肥胖儿童血瘦素及血脂成分的影响，为家长、教育系统建立有效可行的儿童肥胖干预措施提供科学依据。方法：单纯性肥胖儿童采取健康知识教育、有氧运动、合理饮食等疗法进行为期10个月的综合干预试验，测定干预前后身高、体重、腰围、臀围、血瘦素及血脂水平的变化。结果：经过10个月的综合干预活动，干预组肥胖儿童腰围、臀围、血瘦素、TG、LDL－C和$apoB_{100}$较干预前明显降低（$P<0.05$），HDL－C、apoA－Ⅰ较干预前明显升高（$P<0.05$）；而对照组上述指标在干预前后无明显变化（$P>0.05$）。结论：综合干预措施能有效降低体重，改善肥胖儿童体内瘦素和血脂成分，起到调节异常内分泌代谢的作用。

【关键词】　儿童　单纯性肥胖　干预措施　瘦素　有氧运动

Effect of aerobic exercise comprehensive intervention measures on leptin and blood fat composition in obese children

XU Sheng－Li. Xinxiang Medical College, Xinxiang 453003, *Henan, China*

〔**Abstract**〕　**Objective**: To investigate the effect of comprehensive intervention measures on leptin and blood fat composition in simple obese children, and give some advice to parents and education division. **Methods**: Simplex obese children accepted the comprehensive intervention measures composed of health eduction, aerobic exercise, reasonable diet, and medical supervision for ten month. Then the changes of length, weight, waist circumference, hip circumference, blood leptin and blood fat were examined pre － and post － intervention. **Results**: After ten month intervention, weight, waist circumference, hip circumference, the level of leptin, LDL－C, apoB 100 in intervention group were obviously lower compard with those pre－intervention group ($P<0.05$). HDL－C, apoAl increased significantly ($P<0.05$), but these parameters in control group were not obviously different compared with those in pre－intervention ($P>0.05$). **Conclusion**: Comprehensive intervention measures can significantly reduce the body weight and improve the level of leptin and blood fat composition in obese children, so it plays an important role in modifying the metabolism disorder.

〔**Key words**〕　Children; Simplex obesity; Intervention measures; Leptin; Aerobic exercise

随着人物质生活水平的不断提高，儿童单纯性肥胖的发生率呈日益增加的趋势，肥胖不仅影响外观，给生活带来不便，而且还影响儿童的生长发育、智力开发、生理机能、身心健康，更重要的是儿童肥胖可引起成年高血压、冠心病、糖尿病等多种并发症[1-3]，肥胖给儿童健康带来的近期及远期危害，使得肥胖的预防及其治疗显得极其重要。在探讨儿童科学、合理减肥的方法中，课题组研究了一套健康知识教育、有氧运动、合理膳食、行为矫正和医务监督的综合干预方法，为儿童肥胖及其并发症的预防和治疗提供一定的科学依据。

1　资料和方法

1.1　干预对象　本研究儿童采用WHO推荐的身高标准体重法，即≥身高标准体重120%为肥胖[4,5]。2005年10月选取单纯性肥胖儿童60例，来自新乡市普通中学，年龄8～14岁，平均（10.2±2.8）岁，其中男33例，女27例。经过医学筛选，排除内分泌疾病，随机分成干预组和对照组（各30例），两组儿童年龄、体重及肥胖情况均无差异。对照组一切活动自由；干预组严格按照干预处方执行，整个干预安排在第二课堂进行，共用10个月完成。

1.2　综合干预处方的制定[6-8]　①健康知识教育：采取观看教育录像、举办系列知识讲座、发放自编的宣传手册等方式，宣传肥胖防治知识，包括怎样控制体重、BMI如何计算与评价、食物的种类及其营养价值、合理膳食、科学锻炼、肥胖与慢性病关系等，并给其家长发放通知，教给家长一些科学的推算方法和常规干预措施；②有氧运动：活动时间为每天下午的课外活动，活动频率为每周5～7次，每次活动时间为20～30 min，根据每个干预对象的个体特征，制定出不同的全面性训练内容，以运动时脉搏达到145～150次/min为运动强度，选择跑步、踢毽、跳皮筋、爬楼梯、跳绳等运动方式，最初时间可以10 min左右，以孩子不感到过度疲劳，每日能坚持运动为原则，循序渐进；③合理饮食：一日三餐实施低热量平衡膳食，食物的选择要注意营养成份，低热量，具有

①基金项目：2003年全国教育科学“十五”规划教育部重点课题（批号：DLA030208）
2005年河南省教育厅资助项目<2005。ZX－405）
2006年河南省社科联调研课题（SKL－2006－950）

万方数据

饱腹感的食品；减少高油脂、高淀粉类食物，而适当增加蔬菜、水果、豆制品及纤维素的摄入；烹调方法多用煮、炖、凉拌、清蒸或生吃，改变进食快、嗜食油炸食品、偏食等不良饮食习惯，增加食物的咀嚼时间；在原有基础上先减少1/4，逐渐过渡到减少1/3～2/3。根据每个受试对象的个体特点，制定出一日三餐的饮食食谱并坚持膳食记录；④行为矫正：要求改变原来的饮食、运动以及生活习惯，使能量处于负平衡状态，父母应以积极的态度和行动参与到孩子的饮食及运动行为中去，鼓励和支持孩子；⑤医务监督：在减肥过程中，定期测量他们的体重、心率、血压等生理指标，及时观察他们身体对运动负荷和饮食处方的反应，及时调整减肥处方。

1.3　血样的采集和测定　清晨空腹抽取肘静脉血5 ml，静置30 min后，以3 000 r/min离心15 min，取上层血清，－70 ℃保存。血糖（GLU）、甘油三酯（TG）、胆固醇（TC）的测定用全自动生化分析仪（日本OlympusAu 400）；载脂蛋白B_{100}（$apoB_{100}$）、载脂蛋白A－I（apoA－I）的测定采用免疫透射比浊法；瘦素（leptin）的测定采用放射免疫法，试剂盒由美国Headquarters公司提供。

1.4　数据处理　采用SPSS 10.0统计软件包进行处理，用（$\bar{x}\pm s$）表示，采用方差分析、t检验，$P<0.05$为有统计学意义。

2　结果

2.1　干预前、后形态指标的变化　经过10个月的减肥干预试验，干预组单纯性肥胖儿童在身体形态方面发生了很大的变化。其中平均体重减少6.7 kg，BMI、臀围、腰围等各项指标干预后与干预前比较差异有显著性意义（$P<0.05$）；而对照组上述指标在干预前、后比较无显著性差异。

肥胖儿童干预前、后身体形态变化的比较详见表1。

表1　肥胖儿童干预前、后身体形态变化的比较（$\bar{x}\pm s$）

项目	对照组		干预组	
	干预前	干预后	干预前	干预后
身高(cm)	145.56±6.24	145.97±5.43	145.20±3.40	145.78±7.59
体重(kg)	56.54±3.21	57.17±4.87	56.87±3.51	50.14±3.42①
BMI	25.28±1.56	25.64±3.46	25.73±1.29	21.37±3.54①
腰围(cm)	80.31±4.56	80.79±2.57	80.47±4.26	69.37±3.57①
臀围(cm)	85.56±3.25	85.06±2.28	85.26±3.52	80.26±1.46①

与本组干预前比较，①$P<0.05$

2.2　干预前、后血瘦素和血脂成分的变化　干预组肥胖儿童瘦素（lep）、TG、LDL－C和$apoB_{100}$水平较干预前明显降低（$P<0.05$），HDL－C、apoA－I较干预前明显升高（$P<0.05$）；而对照组上述指标在干预前后无明显变化。

肥胖儿童干预前后血糖、瘦素和血脂变化的比较详见表2。

表2　肥胖儿童干预前后血糖、瘦素和血脂变化的比较（$\bar{x}\pm s$）

项目	对照组		干预组	
	干预前	干预后	干预前	干预后
GLU(mmol/L)	4.23±0.24	4.23±0.28	4.24±0.42	4.22±0.26
TG(mmol/L)	1.20±0.23	1.22±0.35	1.20±0.52	0.98±0.42①
TC(mmol/L)	4.23±1.06	4.24±0.88	4.24±0.86	4.19±0.97
HDL－C(mmol/L)	1.35±0.52	1.35±0.57	1.35±0.67	1.56±0.37①
LDL－C(mmol/L)	2.94±0.25	2.95±0.49	2.95±0.52	2.56±0.58①
lep(ng/L)	32.25±8.59	32.56±10.26	33.57±12.54	19.56±7.69①
$apoB_{100}$(g/L)	0.89±0.25	0.88±0.17	0.89±0.21	0.79±0.13①
apoA－I(g/L)	1.21±0.22	1.22±0.34	1.20±0.31	1.32±0.28①

与本组干预前比较，①$P<0.05$

3　讨论

在探讨儿童科学合理减肥的方法中，有研究显示：从降低体重结果看采用极低热量饮食只能维持6～10个月，加上心理矫正可持续1～2年，再加上有氧运动可持续1～6年。因此，本课题组采用健康知识教育、有氧运动、合理营养、行为矫正和医务监督综合干预措施。

饮食与运动干预是防治肥胖的两大基本方法，然而与成人减肥不同，儿童肥胖的控制必须以不影响其正常的生长发育为前提，这一点也是家长最关注的，同时也是影响家长支持子女控制体重的主要心理障碍。因此，笔者在设计儿童肥胖控制活动中特别强调了根据儿童的个性特点给每个儿童具体的、科学的指导，经与肥胖儿童的家长和肥胖儿童充分协商后，共同确定健康教育目标。而且本干预首次采用医务监督方法，即在干预过程中组成了由运动医学专家、专业健身教练、临床医师、营养学人士的医务监督队伍，及时观察他们身体对运动负荷和饮食处方的反应，定期测量他们的体重、心率等生理指标，并结合血糖、血脂等项目的生化检验指标，及时调整运动量、运动时间及营养处方等，以保证整个干预活动的正常顺利进行。

流行病学研究资料表明[9]，HDL－C水平与冠心病危险因素呈负相关，LDL－C水平与冠心病危险呈正相关。肥胖儿童体内脂质代谢紊乱表现为TG、TC、LDL－C的增高及HDL－C的降低。TG水平升高影响LDL颗粒结构，使小颗粒的LDL比例升高，易于沉积到动脉粥样硬化斑块上，而儿童TG水平升高常伴随有HDL的降低，并使血液处于高凝状态，故TG升高是动脉粥样硬化的危险因素，HDL－C通过将动脉壁中多余的胆固醇直接地或间接地转运给肝脏进行分解代谢，来发挥其抗动脉粥样硬化的作用，是冠心病的保护因子；LDL－C颗粒相对较小，能很快穿过动脉内膜层，是所有血清脂蛋白中首要的致动脉粥样硬化性脂蛋白，apoA－I是HDL的主要结构蛋白，主要功能是激活卵磷脂胆固醇酰基转移酶，对胆固醇逆向转运起重要作用，$apoB_{100}$是LDL、VLDL的结构蛋白，参与脂质转运，血清$apoB_{100}$及其LDL－C水平与动脉粥样硬化呈正相关，是动脉粥样硬化的危险因素。

瘦素是由脂肪组织分泌的一种肽类激素，具有广泛的生物学效应，一方面是增加能量消耗和减少摄食，参与能量平衡，从而使体重降低而减肥；另一方面瘦素还与神经内分泌

万方数据

之间组合成一条回路，影响胰岛素水平从而影响内分泌代谢过程。研究已表明[10]：肥胖儿童血瘦素水平均显著高于正常水平，即存在瘦素抵抗。在高瘦素存在下，致使体内糖类、脂类代谢异常，出现血甘油三脂、血糖、胆固醇、极低密度脂蛋白及游离脂肪酸增高，而高密度脂蛋白减少，极易发生动脉硬化、冠心病等。

我们的干预研究表明：采用综合干预措施进行为期10个月的干预活动后，肥胖儿童的体重、BMI、臀围、腰围均显著回降，说明此方法具有良好的减肥作用。同时干预组儿童lep、TG、LDL－C和$apoB_{100}$较干预前明显降低，HDL－C、apoA－I较干预前明显升高，说明此方法减肥后能降低肥胖者体内血瘦素水平和改变血脂成分，从而起到调节异常内分泌代谢的作用，减轻和消除了导致动脉粥样硬化、高血压、冠心病、2型糖尿病等一些潜在于单纯肥胖儿童体内的危险因素，使他们能够更好地健康成长。

4 参考文献

1 叶　军，韩连书，邱文娟 *et al*. 儿童、青少年肥胖者2型糖尿病和高危者筛查. 中华内分泌代谢杂志，2004，20（2）：132

2 王小引，陈艳霞，郭学鹏 *et al*. 综合减肥对超重和肥胖女学生性激素和生长激素的影响. 实用儿科临床杂志，2006，21（8）：467

3 李　炼，许　彤. 肥胖的流行现状、危害及防治对策研究进展. 实用预防医学，2003，10（5）：821

4 季成叶，孙军玲. 中国学龄青少年体重指数地域与人群分布差异的分析. 中华儿科杂志，2004，42（5）：328

5 季成叶. 中国学生超重肥胖BMI筛查标准的应用. 中国学校卫生，2004，25（1）：125

6 冯承芸，邓　冰，熊　敏. 儿童肥胖健康干预研究. 中国健康教育，2005，21（8）：620

7 傅兰英，王小引，王培勇 *et al*. 青少年学生单纯性超重与肥胖的综合干预方法研究. 体育科学技术，2006，42（2）：68

8 吕晓昌. 有氧锻炼负荷强度对儿童心理健康的影响. 体育学刊，2003，10（1）：43

9 许逢明，黄宇戈，温扬祥 *et al*. 单纯性肥胖儿童血脂水平与血管内皮功能的相关性研究. 中国妇幼保健，2005，20（11）：1361

10 陈瑞敏，杜敏联. 瘦素、肥胖与青春期发育. 国外医学·妇幼保健分册，2005，16（2）：95

（2006-07-03　收稿）

〔编校　邹庆红〕

双重病因对新生儿高胆红素血症发生率影响探讨

崔雪芳　李锡福　广东省中山市陈星海医院　528415

中国图书分类号　R722　文献标识码　B　文章编号　1001-4411（2007）21-2937-02

【摘　要】　目的：探讨存在葡萄糖6磷酸脱氢酶（G6PD）缺乏和新生儿溶血病（HDN）双重病因对新生儿高胆红素血症发生率的影响。方法：按检验操作规程对足月健康新生儿脐血进行G6PD和HDN血型血清学检验，对存在G6PD缺乏和HDN病因的新生儿调查其高胆红素血症发生率。结果：①G6PD缺乏组、HDN病因组、G6PD缺乏与HDN双重病因组的患儿高胆红素血症发生率均极显著高于正常对照组的新生儿（$P<0.01$）；②存在G6PD缺乏和HDN双重病因的患儿高胆红素血症发生率极显著高于G6PD缺乏组和HDN病因组的患儿（$P<0.01$）。结论：存在G6PD缺乏和HDN双重病因的患儿红细胞溶血破坏的几率增高，其发生高胆红素血症的可能性更大。

【关键词】　双重病因　高胆红素血症　葡萄糖6磷酸脱氢酶　新生儿溶血病

A study of the effect of dual causa morbi on the morbidity of hyperbilirubinemia among neonates

CUI Xue－Fang, LI Xi－Fu. Chenxinghai Hospital, Zhongshan 528415, *Guangdong, China*

〔Abstract〕　**Objective**: To study the effect of glucose－6 phosphate dehydrogenate (G6PD) deficiency and hemolytic disease of the newborn (HDN) on the morbidity of hyperbilirubinemia among neonates. **Methods**: According to clinic detection rules, G6PD and HDN with umbilical blood of the mature healthy neonates were determined to study the morbidity of hyperbilirubinemia of the neonates with dual G6PD deficiency and HDN. **Results**: ①Comparing to control group, the morbidity of hyperbilirubinemia was extremely higher in the G6PD deficiency, HDN group or dual G6PD deficiency and HDN group ($P<0.01$). ②Comparing to G6PD deficiency group or HDN group, the morbidity was extremely higher in dual G6PD deficiency and HDN group ($P<0.01$). **Conclusion**: In the dual G6PD deficiency and HDN patient－infants, the ratio of the wrecked erythrocyte is higher and the possibility of developing hyperbilirubinemia is much more.

〔Key words〕　Dual causa morbi; Hyperbilirubinemia; Glucose－6 phosphate dehydrogenate (G6PD); Hemolytic disease of the newborn (HDN)

高胆红素血症是新生儿期最常见的临床症状，可由多种病因所致。对新生儿脐血进行检验时，有时会遇到新生儿同

万方数据

有氧运动综合干预措施对肥胖儿童血瘦素和血脂成分的影响

作者： 许胜利， XU Sheng-Li
作者单位： 河南省新乡医学院,453003
刊名： 中国妇幼保健 ISTIC PKU
英文刊名： MATERNAL AND CHILD HEALTH CARE OF CHINA
年，卷(期)： 2007,22(21)
被引用次数： 5次

参考文献(10条)

1. 叶军;韩连书;邱文娟 儿童、青少年肥胖者2型糖尿病和高危者筛查[期刊论文]-中华内分泌代谢杂志 2004(02)

2. 王小引;陈艳霞;郭学鹏 综合减肥对超重和肥胖女学生性激素和生长激素的影响[期刊论文]-实用儿科临床杂志 2006(08)

3. 李炼;许彤 肥胖的流行现状、危害及防治对策研究进展[期刊论文]-实用预防医学 2003(05)

4. 季成叶;孙军玲 中国学龄青少年体重指数地域与人群分布差异的分析[期刊论文]-中华儿科杂志 2004(05)

5. 季成叶 中国学生超重肥胖BMI筛查标准的应用[期刊论文]-中国学校卫生 2004(01)

6. 冯承芸;邓冰;熊敏 儿童肥胖健康干预研究[期刊论文]-中国健康教育 2005(08)

7. 傅兰英;王小引;王培勇 青少年学生单纯性超重与肥胖的综合干预方法研究[期刊论文]-体育科技 2006(02)

8. 吕晓昌 有氧锻炼负荷强度对儿童心理健康的影响[期刊论文]-体育学刊 2003(01)

9. 许逢明;黄宇戈;温裼祥 单纯性肥胖儿童血脂水平与血管内皮功能的相关性研究[期刊论文]-中国妇幼保健 2005(11)

10. 陈瑞敏;杜敏联 瘦素、肥胖与青春期发育[期刊论文]-国外医学(妇幼保健分册) 2005(02)

本文读者也读过(10条)

1. 何飞 丰富多彩的健身走[期刊论文]-少林与太极2010(6)
2. 何惠丽.许浩.邵慧秋.HE Hui-li.XU Hao.SHAO Hui-qiu 步行锻炼对江苏省公务员体质的影响[期刊论文]-南京体育学院学报（自然科学版）2008,7(2)
3. 王福彦.李昌茂 城乡小学生单纯性肥胖现状调查[期刊论文]-中国妇幼保健2007,22(12)
4. 谢培益 您不知道的血脂成分[期刊论文]-糖尿病天地2007(8)
5. 郭志强.GUO Zhi-qiang 有氧运动健身的生理学分析[期刊论文]-哈尔滨体育学院学报2009,27(3)
6. 廖雪玲.马娟.楼滨.吴满平 血浆高密度脂蛋白的组成成分在LPS诱导的急性相反应中的改变[期刊论文]-复旦学报(医学版)2004,31(5)
7. 张德甫.胡巧云.陈姜.郗园林.耿敏学.ZHANG De-fu.HU Qiao-yun.CHEN Jiang.XI Yuan-lin.GENG Min-xue 肥胖儿童血清瘦素水平与体格发育的关系[期刊论文]-实用儿科临床杂志2005,20(6)
8. 刘军.刘芳.陈影.徐东丽.姚庆姑 2型糖尿病患者心血管危险因素集簇现象[期刊论文]-复旦学报(医学版)2004,31(2)
9. 柴军土.CHAI Jun-tu 2型糖尿病患者大血管病变与脂蛋白(a)的关系[期刊论文]-河北医科大学学报2010,31(6)
10. 王慧.王晓芹.WANG Hui.WANG Xiao-qi 糖尿病合并急性脑梗死老年患者的血脂分析[期刊论文]-实用医药杂志2011,28(3)

引证文献(5条)

1. 戈越红 护理干预在儿童单纯性肥胖症患者中的疗效观察[期刊论文]-中国卫生产业 2013(34)

2. 张薇 学龄前儿童单纯性肥胖的减肥策略和调查分析[期刊论文]-中外健康文摘 2012(3)

3.曾援.钟日英.郑欣 儿童肥胖的预防及干预[期刊论文]-中外医学研究 2012(15)

4.胡瑞.李晓春 儿童单纯性肥胖干预措施研究进展[期刊论文]-护理学报 2009(14)

5.黄亚茹.纪环.葛小川.胡扬.毛小云 4周运动配合饮食控制对肥胖青少年体成分、血脂的影响及相关调控机理[期刊论文]-中国体育科技 2013(1)

引用本文格式：许胜利.XU Sheng-Li 有氧运动综合干预措施对肥胖儿童血瘦素和血脂成分的影响[期刊论文]-中国妇幼保健 2007(21)
