# Supplementary material for: Systematic review and meta-analysis of school-based obesity interventions in mainland China
Source: PLoS One. 2017 Sep 14;12(9):e0184704. doi: 10.1371/journal.pone.0184704 (PMC5598996; doi:10.1371/journal.pone.0184704)
Supplement: S1 Dataset — (ZIP) [file pone.0184704.s007.zip › S1_dataset/76库/67.pdf]

【学生营养】

儿童单纯性肥胖的社区群体干预效果评价

黄水平, 邵继红, 姜海霞, 卓朗, 陈虎

【摘要】 目的 研究学龄儿童单纯性肥胖的干预对策, 为预防儿童单纯性肥胖提供依据。方法 采用分层整群抽样法, 抽取徐州市8所小学7～11岁肥胖学生405名为研究对象, 其中4所学校的肥胖学生278人为干预组, 另4所学校的127名肥胖学生为对照组。采用以社区为基础的学校群体干预方法, 对干预学校的学生进行2 a的干预和追踪研究, 并在干预前、后分别进行问卷调查和体格测量。结果 干预学校的学生和家长关于肥胖和合理营养的知识、态度和行为的正确率比对照学校高, 肥胖儿童的BMI有下降趋势。结论 以社区为基础的学校群体干预方法, 对肥胖儿童有一定预防和控制作用, 值得在社区推广。

【关键词】 肥胖症; 干预性研究; 社区活动; 结果评价(卫生保健); 儿童

【中图分类号】 R 151.1 G 479 R 153.2 【文献标识码】 A 【文章编号】 1000-9817(2005)11-0897-02

Effects of Community Group Intervention on Childhood Simple Obesity/ *HUANG Shui-ping, SHAO Ji-hong, JIANG Hai-xia, et al. Department of General Medicine, Xuzhou Medical College, Jiangsu(221002), China*

【**Abstract**】 **Objective** To study the intervention program of preventing against simple obesity among school-aged children, and to provide evidence for preventing childhood simple obesity. **Methods** By using stratified cluster sampling method, 405 obesity students (aged 7—11) were chosen from 8 primary schools. Two hundred and seventy-eight obese children from 4 schools were taken as intervention group, 127 ones from another 4 schools were taken as control. With the method of school-based community intervention, 2 years intervention and follow-up study were conducted among the students. Data of questionnaire, weight and height were collected before and after the intervention performed. **Results** Compared with the control schools, the correct rates of knowledge, attitude and practice of obesity and adequate diatery among students and their parents were higher in the intervention schools, and the BMI appeared a decreasing trend. **Conclusion** School-based community group intervention is efficient to obese children, and it is worthy of being popularized in community.

【**Key words**】 Obesity; Intervention studies; Community action; Outcome assessment(health care); Child

学龄儿童是肥胖的高发人群, 儿童时期严重的肥胖会使儿童的社会适应能力、活动能力、社交能力下降, 产生各种行为问题[1]。徐州市儿童肥胖的形势非常严峻, 且儿童肥胖与营养过度以及饮食行为偏差和缺乏必要的营养知识等有关[2]。为探索有效防治儿童肥胖的群体措施, 制订防治对策, 进行肥胖的一级预防, 笔者于2002年9月～2004年9月以社区为基础, 对肥胖儿童、家长和学校教师开展了有针对性的学校群体干预, 取得了一定的效果。现报道如下。

1 对象与方法

1.1 对象 采用分层整群抽样法, 于2002年6月在徐州市抽取8所学校7～11岁的肥胖学生425名为研究对象, 有效登记人数为405名。其中4所学校的肥胖学生278人为干预组, 另外4所学校的127人为对照组, 所有调查对象均无心肺及内分泌疾病。

1.2 干预方法 根据因素分析结果, 与学校、家长合作, 采用以社区为基础的学校群体干预方法, 对干预学校的肥胖学生进行2 a(2002年9月～2004年9月)的干预和追踪研究, 干预措施包括对家长和学生集中授课、专家咨询、制作健康教育电教材料、在学校出专题黑板报、个别联系辅导答疑、学校督促、家长会以及奖励等多种形式, 提出合理膳食、运动和生活习惯改进建议。同时, 一旦发现问题, 再次进行健康教育。对照组不采取干预措施, 仅在学校正常上卫生保健课。

1.3 质量控制 严格按照抽样方法取样, 调查人员经培训后按统一要求进行问卷调查, 统一仪器进行体检, 并注意校验测量仪器; 专人负责督察, 专人录入资料。

1.4 体格检查 分别于2003年10月和2004年10月进行2次体检, 指标为身高、体重等。

1.5 儿童超重与肥胖的判断 采用体质指数(BMI)法, BMI=体重(kg)/身高(m)^2, 以2003年11月全国儿童肥胖学术会议公布的不同年龄、性别BMI标准为依据。

1.6 统计方法 原始资料经复核后, 用Epi Date 3.0双份录入计算机。经核对、逻辑纠错后建立数据库, 用SPSS 11.5, State 7.0软件进行数据处理。

2 结果

2.1 干预前后干预组与对照组BMI变化情况 干预前后干预组与对照组BMI变化差别有统计学意义($\chi^2=8.796$, $P=0.012$), 见表1。

表1 干预组与对照组学生干预前后BMI变化情况

组别	人数	BMI减少	BMI不变	BMI增加
对照组	127	62(48.8)	0	65(51.2)
干预组	278	133(47.8)	18(6.5)	127(45.7)

注: ()内数字为构成比/%。

2.2 干预前后干预组与对照组对肥胖危害和营养知识的知晓情况 采用以社区为基础的学校群体干预儿童肥胖措施后, 以问卷调查方法了解学生和家长有关儿童肥胖危害、合理营养等相关知识知晓率、行为改善和态度(KAP)等情况, 结果见表2。

经过以社区为基础的学校预防和控制肥胖的健康教育, 干预组儿童对肥胖危害的认识和对营养知识的掌握都有了一定程度的提高, 且对肥胖的防治有一定的促进作用, 其效果好于对照组。

【基金项目】 江苏省教育厅自然科学基金项目(02KJD330002)。

【作者简介】 黄水平(1963～), 女, 江苏启东人, 研究生, 教授, 主要从事流行病与卫生统计工作。

【作者单位】 徐州医学院全科医学系, 江苏 221002。

?1994-2015 China Academic Journal Electronic Publishing House. All rights reserved. http://www.cnki.net

表2 干预组与对照组干预前后健康教育效果比较(P 值)

指标	对照组	干预组
对控制孩子的饮食并进行适当运动的态度	0.000	0.839
家长认为自己孩子的体型是否肥胖	0.051	0.044
家长对控制孩子肥胖的态度	0.137	0.000
对合理控制孩子肥胖方式的认识	0.678	0.009
对儿童肥胖原因的认识	0.601	0.309
是否认为主食量太多	0.902	0.717
是否认为动物类食品吃得太多	0.197	0.514
是否认为油炸食品吃得太多	0.110	0.156
是否认为晚餐吃得太多	0.264	0.931
是否认为零食吃得太多	0.861	0.578
是否认为肥胖可能引起消化系统疾病	0.193	0.382
是否认为肥胖可能引起心血管疾病	0.795	0.501
是否认为肥胖可能引起心理障碍	0.291	0.408
控制肥胖是否需要良好的行为生活方式	0.952	0.018
控制肥胖是否需要合理营养	0.440	0.163
控制肥胖是否需要掌握防治知识	0.767	0.257
是否了解《中国居民平衡膳食宝塔》	0.648	0.000
是否了解《中国居民膳食指南》	0.892	0.000
是否了解《特定人群膳食指南》	0.557	0.001
是否了解食物分类	0.000	0.000
是否了解营养素种类	0.000	0.000
是否了解高血压与食盐量有关	0.000	0.008
是否了解早餐对健康有影响	0.433	0.961
经常不吃早餐对健康是否有影响	0.149	0.169
对早餐食物种类的认识	0.051	0.000
饮食行为对将来身体健康的影响	0.533	0.174
营养知识对健康是否很重要	0.350	0.888
对改变孩子不良饮食行为的认识	0.520	0.471
对营养知识的兴趣	0.533	0.894
对开展营养健康教育课的态度	0.652	0.108

3 讨论

学校是儿童成长的重要场所。研究认为,各种危险因素在不同年龄时期对肥胖的作用也是不同的[3]。学龄时期正是儿童逐渐独立、体重增加较快的危险时期,被看作是纠正影响儿童肥胖的生活方式和饮食习惯的有效时期。但儿童对膳食和生活方式的自控能力低,更多的是依靠父母、家庭和社会环境以及学校的引导教育;对干预的力度和方法的要求更高,对学校和家庭的参与要求也更高。另外,我国城市儿童多为独生子女,受家庭呵护溺爱;同时随着家庭收入的增加,吃零食和在外就餐现象普遍增加,儿童不良饮食习惯和生活方式的改变难度也增大[4]。

笔者采用以社区为基础的学校健康教育,在取得社区、教育单位和家长配合同时,通过社区、学校、家庭联合行动,对肥胖儿童进行群体干预,收到了一定的效果。肥胖儿童健康相关知识知晓率得以提高,合理饮食行为和生活方式有了一定的改善;干预学校学生的肥胖和超重上升趋势在一定程度上得到缓解。但这种防治效果还是有限的,特别需要提高和巩固,而且也不能指望一段时间的干预就能持续改变肥胖孩子的所有不良饮食习惯和行为生活方式,还需要在社区、学校、家庭形成预防儿童肥胖的合力和社会环境。

目前,在持续预防和控制儿童肥胖方面,仍存在一些困难,如家长合理营养和肥胖控制的意识还有待进一步提高;学校虽然初步建立了一些防治对策,对儿童肥胖危害已有一定认识,但对儿童肥胖预防的重视程度还不够。此外,社区和家庭运动空间与设施有限,限制了孩子有足够的运动量,加之学生的课业负担较重,家长、学校和社会重视的是孩子的学习成绩,而忽略了保障孩子有足够的运动时间和运动量。这些影响因素是持续预防和控制的难点,也使得扩大和巩固防治效果的难度加大。

4 参考文献

[1] 蔡美琴,王少墨,张晓敏,等. 上海市中小学生肥胖流行情况及影响因素的相关分析. 上海第二医科大学学报, 2002, 22(1): 74-77

[2] 邵继红,黄水平,陈虎,等. 徐州市儿童单纯性肥胖调查及不同诊断标准的比较分析. 徐州医学院学报, 2004, 24(3): 244-247

[3] David S, Laura K, William H, et al. Relationship of childhood obesity to coronary heart disease risk factors in adulthood: The Bogalusa heart study. Pediatrics, 2001, 108(3): 712-718

[4] Patrick K, Norman GJ, Calfas KJ, et al. Diet physical activity and sedentary behaviors as risk factors for overweight in adolescence. Arch Pediatr Adolesc Med, 2004, 158(4): 385-390

(收稿日期: 2005-04-08)

(上接第896 页)

陶芳标等[6]曾在中专学生中研究发现,随着生活事件数量的增加, SCL-90 评定的心理卫生问题检出率增加,呈明显的剂量-反应关系。本文研究结果也反映出这样一种趋势,由于女性在处理事件时特定的态度、推论方式以及沉思的反应方式,比男生表现出更多的消极归因;随着负性生活事件的不断累积,她们惯用的消极应对方式使其情绪的表达也趋于内在化,从而表现出较多的抑郁、焦虑倾向。这种负性情绪增加到一定程度时,就会产生明显的抑郁、焦虑症状。反过来,本身就有明显抑郁、焦虑症状的人,情绪起伏较大,社会适应和应激能力差,在面对各种紧张性刺激时,更容易依靠别人,而不是积极依靠自己或寻求解决的方式,这种思维模式也使有抑郁、焦虑的女生消极应对的倾向增加。生活事件作为一种应激源,联结了抑郁、焦虑症状与应对方式的相互作用。

本研究对开展专科学校心理卫生工作的启示是,学校和社会在努力提高办学质量的同时,应注重培养学生积极乐观的人生态度,发展社交技能,改善家庭环境。尤其是对更加敏感、细腻并且已有明显情绪症状的女生,应针对负性情绪不同的成因进行干预,通过减少环境中的不利因素减少焦虑、抑郁症状的发生。

4 参考文献

[1] 汪向东,王希林,马弘,编. 心理卫生评定量表手册(增订版). 北京: 中国心理卫生杂志, 1999. 106-238

[2] 李作佳,周秋华,于振华. 中小学教师生活事件、应对方式与焦虑、抑郁的相关性研究. 中国临床心理学杂志, 2003, 11(4): 285-286

[3] 苏虹,程慧,张秀军,等. 919 名幼师女生抑郁、焦虑情绪的流行学特征及相关因素. 中国心理卫生杂志, 2003, 17(7): 465-467

[4] Bebbington PE. Sex and depression. Psychol Med, 1998, 28(1): 1-8

[5] Davidson RJ, Jackson DC, Kalin N. Emotion, plasticity, context and regulation: perspective from affective neuoscience. Psychol Bull, 2000, 126(6): 890-909

[6] 陶芳标,汪宏珊,曾广玉,等. 中专学生负性生活事件及其对心理健康影响. 中国校医, 1998, 12(1): 10-14

(收稿日期: 2005-03-16)

?1994-2015 China Academic Journal Electronic Publishing House. All rights reserved. http://www.cnki.net
