# Supplementary material for: Systematic review and meta-analysis of school-based obesity interventions in mainland China
Source: PLoS One. 2017 Sep 14;12(9):e0184704. doi: 10.1371/journal.pone.0184704 (PMC5598996; doi:10.1371/journal.pone.0184704)
Supplement: S1 Dataset — (ZIP) [file pone.0184704.s007.zip › S1_dataset/76库/68.pdf]

[文章编号] 1001- 7062(2005)02- 0115- 03　[中图分类号] R 153.2　[文献标识码] B

[论著]

儿童单纯肥胖症群体综合干预研究

刘素芹[1], 张清华[1], 岳亿玲[2], 李玉芹[2]

[摘　要] 目的　探讨儿童单纯肥胖症的群体综合干预方法,降低儿童肥胖发生率。　方法　从淮北市城区选择 8 所初级中学作为研究现场,其所有在校学生为研究对象,其中 4 所学校为干预组(2 346 人),4 所学校为对照组(2 834 人)。采用 WHO 身高标准值为肥胖判断标准。对干预组进行为期 3 年的群体干预,干预措施包括膳食调整、运动处方、健康教育、行为矫正、耳穴按压等。干预对象为超重儿童及其家长、肥胖儿童及其家长及非肥胖儿童家长。对照组除与干预组同期体检外不施加任何干预措施。　结果　①经过 3 年干预,干预组肥胖发生率从 9.8% 降至 7.0% ($P < 0.01$),对照组肥胖发生率从 9.8% 上升为 12.5% ($P < 0.01$)。干预后两组肥胖发生率差异有显著意义。②干预后肥胖度干预组从 45.9% ±11.3% 降至 34.2% ±11.83%,差异有显著意义($P < 0.05$),对照组从 46.2% ±12.1% 上升至 48.9% ±13.7%,差异无显著意义($P > 0.05$)。干预组与对照组儿童身高增长幅度基本| 致。③干预后血 TCH、TG、APOB 及 SBP 降低,对照组肥胖儿童无明显变化。　结论　儿童单纯肥胖症群体综合干预可有效降低儿童肥胖发生率和肥胖度。

[关键词] 肥胖; 干预研究; 儿童保健服务

School-based intervention for obese children *LIU Su-qin, ZHANG Qing-hua, YUE Yi-ling, LI Yu-qin, et al. Shunde District Maternity and Infant Health Institute of Foshan, Foshan 528300, Guangdong, China*

[Abstract] **Objective** To evaluate the impact of a school-based intervention program to childhood obesity. **Methods** Eight middle schools (4 intervention and 4 control) were selected randomly from Huaiber urban district to attend the field trial. All the children (5 180 students, Grade 1 to 3) from these 8 schools were involved in the study and there were 2 346 cases in intervention schools (intervention group) and 2 834 cases in control schools (control group), respectively. Obesity was defined by WHO standard weight for height. The intervention group participated in a school-based intervention program over 3 school years (from 2000 to 2003). The program included dietary adjustment, sport prescription, health education, behavior corrects and stimulating acupuncture points in ears. **Results** 1. The prevalence of obesity among the children in the intervention group reduced from 9.8% to 7.0% over the 3 school years intervention periods, and it increased from 9.8% to 12.5% in the control group. 2. The degree of obesity among the children in the intervention group reduced from (45.9±11.83)% to (34.2±11.83)% ($P < 0.05$), while the degree of obesity among the children in the control group changed from (46.2±12.1)% to (48.9±13.7)% ($P > 0.05$). 3. The index signs such as TCH, TG, APOB and SBP all dropped after the intervention. **Conclusions** The school-based intervention for obese children can reduce prevalence of obesity and the degree of obesity significantly.

[Kew words] Obesity; Intervention Studies; Child Health Services

儿童单纯肥胖症是儿童期常见的营养问题,其对儿童心理行为和身体发育的影响已引起人们广泛关注,它不仅损害儿童的身心健康,而且还与成人期心血管疾病的发生密切相关。儿童单纯肥胖的发生率在不同国家、地区和民族有较大的差异。1999 年淮北市中小学生营养状况调查显示,儿童肥胖发生率为 9.8%,其中男生为 12.5%,高于全国城市男生平均值(12.03%)[1]。为探讨儿童单纯肥胖症的群体综合干预方法,减少肥胖的发生,同年,课题组在部分中学试行群体综合干预方案,结果有效地降低了儿童单纯肥胖症的发生率。本文旨在探讨儿童单纯肥胖症的群体干预方法。

[作者单位] 1. 佛山市顺德区妇幼保健院(广东　佛山　528300); 2. 安徽省淮北市人民医院

[收稿日期] 2004- 12- 28

1　对象和方法

1.1　对象　以淮北市初中学生为对象,根据调查致胖因素、学校情况等制定群体综合干预方案,随机抽取 4 所学校(2 346 人)作为干预组,另 4 所学校(2 834人)作为对照组,两组所处的城市位置、学校设施、生源等基本情况相同。

1.2　方法

1.2.1　体格测量　由统一培训的专职医务人员进行。采用《全国学生常见病综合防治方案技术规范》所规定的身高体质量测量方法和测量器具进行测量,体质量精确到 0.1 kg,身高精确到 0.1 cm。按 WHO 推荐的学生身高别体质量标准,结合我国学生 7~ 22 岁《营养评价参考标准(身高标准体重)》进行评价。将前后两次测查结果进行比较分析。按儿童

© 1994-2011 China Academic Journal Electronic Publishing House. All rights reserved.　http://www.cnki.net

肥胖诊断标准, 以体质量超过身高标准体质量的 20% ~ 29% 为轻度肥胖, 超过 30% ~ 49% 为中度肥胖, 超过 50% 以上为重度肥胖。

1.2.2. 方案制定 使用自行设计的《青少年健康与行为调查表》、《学生营养知识、态度与行为调查表》, 经预试验后对在校学生进行问卷调查, 将肥胖儿日常生活行为与正常体质量儿对比、分析, 寻找导致肥胖的不良因素, 制定并不断完善综合干预方案。

1.2.3 干预实施 群体干预由校医、体育老师督导, 利用宣传单、健康教育课堂、黑板报等宣传合理营养的重要性; 开设营养咨询门诊; 开设家长课堂每学期 2 次, 对全体家长进行营养知识讲座。学校、家庭、医生、宣传媒体共同参与, 营造控制肥胖氛围。干预组肥胖学生写减肥日记, 家长参与督导, 营养门诊随访, 每月一次, 如测量体质量、调整饮食、心理教育等, 营养门诊的健身房免费向干预组开放。

1.2.4 干预内容 ①膳食调整: 针对肥胖儿摄入过多, 采取分阶段过渡到正常摄入量。注意引导食粗纤维食物。中重度肥胖者限制进食量, 但青春期日摄入量不低于 6 270kJ, 必须保证充分的蛋白质、维生素、矿物质和微量元素的摄入; ②运动处方: 以学校体育教学大纲要求为基础, 具体内容: 运动强度一般为最大氧耗量的 50% (约为最大心率的 60% ~ 65%) , 每周 3~ 5 次, 时间为 1~ 2 h。③行为矫正: 通过与肥胖儿、家长、教师交谈及观察分析, 寻找致肥的主要危险因素, 确定需纠正的靶行为。要注意家长、教师、小伙伴对肥胖儿治疗过程的强化作用, 创造有利于肥胖儿坚持训练的环境。④健康的危害。⑪耳穴按压: 一般一周贴压一次, 5 周为一疗程。

1.3 统计学处理 所有数据采用 Microsoft Excel 软件处理。

2 结果

2.1 *群体综合干预前后肥胖发生率及超重发生率情况* 我市中学生两次调查结果表明, 肥胖、超重率均呈自然增长趋势, 干预前肥胖率、超重率干预组和对照组间差异均无显著意义。实施干预后, 干预组肥胖率从 9.8% 降至 7.0% ($P < 0.01$) , 而对照组肥胖发生率从 9.8% 上升为 12.5% ($P < 0.01$) , 干预后两组间肥胖率差异有极显著意义($P < 0.01$) , 干预后干预组及对照组超重率均有下降趋势, 差异无显著意义($P > 0.05$, 表 1) 。

表 1 两组儿童干预前后肥胖及超重状况比较

项目	干预组			对照组		
	干预前	干预后	P 值	干预前	干预后	P 值
样本量(例)	2 346	2 328		2 834	2 820	
男	1 199	1 192		1 522	1 516	
女	1 147	1 136		1 312	1 304	
肥胖发生率(%)	9.8	7.0	< 0.01	9.8	12.5	< 0.01
男	12.6	9.4	< 0.05	12.6	16.0	< 0.01
女	6.6	4.5	< 0.05	7.1	7.4	> 0.05
超重发生率(%)	10.2	9.8	> 0.05	9.9	10.8	> 0.05
男	9.6	9.3	> 0.05	10.3	10.5	> 0.05
女	10.7	10.6	> 0.05	9.5	11.1	> 0.05

2.2 *肥胖儿集体综合干预前后肥胖度情况* 综合干预前两组肥胖儿的肥胖度分别为 45.9% ± 11.83%, 46.2% ± 12.1%, 差别无显著意义($P > 0.05$) , 实施干预后肥胖度分别为 34.2% ±11.83%, 48.9% ±13.7% 差异有显著意义($P < 0.05$) , 干预组干预前后差异有显著意义($P < 0.05$) , 而对照组干预前后肥胖度有上升但无显著变化($P > 0.05$) , 肥胖干预组与对照组儿童身高增长幅度基本一致。

2.3 *部分肥胖儿血糖、血脂、血压变化情况* 干预前肥胖儿童 66.7% 有不同程度的脂肪肝, 28.6% 血 TG 超过成人最高限(1.71 mmol/ L) , 24.5% TCH 高于国际上公认小儿最高值(5.17 mmol/ L) , 其中 7.9% 超过成人高限(6.1 mmol/ L) ; 肥胖儿空腹 FBS、APOB 均值高于正常儿, 16.7% 的肥胖儿 SBP 增高(正常值参照年龄血压百分位值。干预后肥胖干预组儿童脂肪肝的好转不明显, 但血 TCH、TG、APOB 及 SBP 降低, 而对照组肥胖儿童无明显变化, 集体干预后心脑血管疾病的危险因素降低(表 2) 。

表 2 干预前后两组肥胖儿童血醣、血脂及血压变化

项目	肥胖干预组(n= 63)				肥胖对照组(n= 63)			
	干预前	干预后	u 值	P 值	干预前	干预后	u 值	P 值
FBS(mmol/ L)	4.56±0.54	4.50±0.52	0.236 6	> 0.05	4.57±0.53	4.57±0.62		
TCH(mmol/ L)	4.46±0.93	4.15±0.92	1.911 2	> 0.05	4.46±0.91	4.46±0.98		
HDL(mmol/ L)	1.32±0.18	1.34±0.16	0.251 3	> 0.05	1.32±0.19	1.32±0.21		
TG(mmol/ L)	1.47±0.39	1.36±0.43	1.879 8	< 0.05	1.47±0.38	1.46±0.41		
APOAI(g/ L)	1.32±0.08	1.32±0.08			1.32±0.12	1.32±0.09		
APOB(g/ L)	0.80±0.17	0.73±0.11	3.401 0	< 0.01	0.81±0.16	0.81±0.18		
SBP(kPa)	15.44±1.65	14.94±1.63	2.235 5	< 0.01	15.53±1.54	15.39±1.51	0.096 5	> 0.05
DBP(kPa)	9.38±1.93	9.24±1.92	0.298 9	> 0.05	9.33±1.70	9.32±1.77	0.227 5	> 0.05

© 1994-2011 China Academic Journal Electronic Publishing House. All rights reserved. http://www.cnki.net

3　讨论

随着我国经济的快速增长及人们生活水平的不断提高, 儿童单纯肥胖症的发生率愈来愈高, 目前已处于几近失控状态[2]。我国学者在儿童肥胖的个体干预方面进行了许多有益的探索[3], 然而小范围的个体干预无法满足快速增长的肥胖人群的需求, 寻找切实可行的群体干预方法, 是当前医务工作者努力的方向。研究表明, 肥胖是由遗传、行为及环境相互作用的多因素所致。淮北市两次营养状况调查表明, 三年来, 肥胖发病率从 9.8% 上升到 12.5%, 且男生增长速率较女生为快[4]。调查结果显示, 不适当的膳食与生活方式、活动量不足及遗传因素是导致儿童肥胖的主要原因。

儿童单纯肥胖症的治疗, 作为世界性医学难题, 至今尚无突破性进展, 包含饮食调整、运动锻炼、行为矫正、健康教育的综合干预方法目前仍是干预儿童期肥胖症的主流方法。本文采用自行设计群体综合干预方案, 对淮北市区三所初中学生(2 346 人) 进行为期三年的综合干预, 结果表明, 干预组肥胖发病率从 9.8% 降至 7.0%, 下降速度为 29.6%, 而对照组肥胖发生率从 9.8% 上升为 12.5%, 增长速度为 24.2%, 干预后肥胖度明显下降, 血 TCH、TG、APOB 及 SBP 降低。

作者曾采用个体化方法对儿童单纯肥胖症进行干预, 虽取得较好效果[5], 但个体化的干预对人力物力的要求太高, 比较而言, 群体干预覆盖面广、经济实惠, 更适合我国国情, 此外, 学校对学生的集体管理使干预方案更易于实施。本干预方案重点放在加强学生营养知识教育、改变其不良生活方式上, 将超重、正常体质量学生纳入干预对象, 这样有利于减少新增肥胖人数, 更重要的是良好的生活方式将可能使孩子受益终生。

在干预过程中作者体会, 在综合干预的几个方面: ①行为干预尤其重要[6], 它是形成一生健康生活模式的重要环节, 国内外已有运用认知行为模式(CBT) 对肥胖群体干预并取得较好短期、长期效果的报道, 过分注重运动处方(尤其运动强度) 的作用有失偏颇。②干预方法注意生活化, 家庭化。由于肥胖儿童运动能力差异较大, 集体干预时统一运动强度有一定难度。因此, 生活化、家庭化的干预方案并定期强化训练, 效果较好。具体操作时, 应灵活掌握, 如根据儿童的不同情况调整运动强度和运动时间的关系等。③强调家长参与。在干预中, 家长参与也相当重要, 只有家长参与其中, 干预才能持久。④注意加强学生营养知识教育。营养知识教育应纳入学校健康教育课堂, 培养良好的生活方式与饮食习惯是减少肥胖形成的重要手段, 这需要家庭、学校、社会的密切配合。

[参考文献]

[1] 岳亿玲, 刘素芹, 张清华, 等. 淮北市中小学生营养状况调查[J]. 中国学校卫生, 2001, 22(2): 141- 142.

[2] 梁国新, 卫巧贤, 刘丽, 等. 短期治疗在儿童单纯性肥胖症治疗中的作用分析[J]. 中国儿童保健杂志, 2003, 11(2): 125- 126.

[3] 冯帮胜, 张慧敏, 钟群英, 等. 健康教育对低龄儿童单纯性肥胖的早期干预研究[J]. 中国妇幼保健杂志, 2004, 19(10) 上半月: 23 - 25.

[4] 岳亿玲, 刘素芹, 张清华, 等. 肥胖儿童心理行为特征配对调查[J]. 中国学校卫生, 2002, 23(8): 341.

[5] 岳亿玲, 刘素芹, 张清华, 等. 47 例单纯性肥胖儿童综合干预成效研究[J]. 安徽预防医学杂志, 2002, 8(4): 223- 225.

[6] 蒋竟雄, 夏秀兰, 吴光驰, 等. 学龄儿童单纯肥胖症的群体干预研究[J]. 中国儿童保健杂志, 2002, 10(6): 364- 368.

[编辑]　樊继忠

© 1994-2011 China Academic Journal Electronic Publishing House. All rights reserved.　http://www.cnki.net
