# Supplementary material for: Systematic review and meta-analysis of school-based obesity interventions in mainland China
Source: PLoS One. 2017 Sep 14;12(9):e0184704. doi: 10.1371/journal.pone.0184704 (PMC5598996; doi:10.1371/journal.pone.0184704)
Supplement: S1 Dataset — (ZIP) [file pone.0184704.s007.zip › S1_dataset/76库/69.pdf]

(1) 盆腔清扫破坏了下段输尿管血供，使管壁血供不足，蠕动功能不良；(2) 输尿管与术区组织粘连，使管壁僵硬，蠕动差；(3) 癌肿局部复发、侵犯及转移；(4) 放射治疗后输尿管周围结缔组织增生反应，进而引起输尿管及周围组织水肿及纤维化；(5) 感染增加输尿管周围组织对放疗的敏感性；(6) 放化疗对输尿管的直接放射性损伤。

上尿路梗阻治疗的关键是解除梗阻，恢复肾功能，缓解临床症状，那么如何在早期解除梗阻成为关键。输尿管内双-J管置入术是一种微创操作，而且尿液的内引流不影响患者的生活质量，是一种值得推荐的方法[3]。本组 16 例患者均首选输尿管内双-J管置入术作为解除尿路梗阻的方法，效果满意，所以本研究认为对于输尿管梗阻的病人，输尿管内双-J管置入术是一种快速有效的解救方法之一，能够迅速缓解梗阻症状；但宫颈癌术后放化疗的病人，特别是有腹膜后纤维化者，输尿管内“双-J”管置入术常失败[4]，目前认为宫颈癌术后放化疗的患者出现“双-J”管无法置入的原因多由于手术后输尿管异位，放化疗后致腹膜后纤维化所致；而对于此类病人最好是能早期行手术治疗，对于肾功能的恢复及病人的生活质量能起到积极的作用，但手术的方式存在着一定的争议，有的学者[5]认为宫颈癌术后放化疗的患者均为晚期肿瘤患者，可能无法耐受大的手术治疗，经皮肾造瘘应为首选；而有的学者[6]认为情况危急时可行经皮肾造瘘治疗，但因宫颈癌术后放化疗而引起的输尿管狭窄多为良性狭窄，所以应以切除狭窄行膀胱输尿管再吻合术或尿流改道术为最佳方案。本组行输尿管内"双-J"管置入术失败的病人首选给予了狭窄段切除及尿流改道治疗，对于 1 例患者全身情况较差及 1 例就诊时肾功能较差的患者给予了经皮肾穿刺造瘘术治疗，效果满意。

4 小结

宫颈癌术后放化疗导致上尿路梗阻多为良性梗阻，解除梗阻后患者肾功能多数可以恢复，但因其发病具有隐蔽性，所以应定期复查泌尿系 B 超，做到早诊断，早期解除梗阻就十分必要。解除梗阻的首选方法为输尿管内“双-J”管置入术，如失败则应行尿流改道术，病人情况危重时可先行经皮肾穿刺造瘘术。正确及时的处理宫颈癌术后放化疗导致上尿路梗阻，可以改善和提高患者的生活质量及生存时间，对于患者日后的进一步放化疗治疗有着积极的意义。

参考文献

1 王素梅，张立华. 晚期宫颈癌伴上尿路梗阻 19 例分析〔J〕. 中国妇幼保健，2006，21 (6)：743-744.

2 Park TK, Kim SN, Kim SW, et al. Concurrent chemotherapy and radiotherapy in invasive cervical cancer patients with high risk factors〔J〕. Korean Med Sci, 2000, 15 (4): 436-441.

3 孙友红，崔林，刘建军，等. 宫颈癌、卵巢癌致上尿路梗阻 23 例分析〔J〕. 交通医学，2007，21 (4)：444-445.

4 徐建平，程明忠，姜在兴. 双 J 管在宫颈癌输尿管梗阻中的应用〔J〕. 临沂医学专科学校学报，2005，27 (6)：409-410.

5 Feng MI, Bellman GC, Shapiro CE. Management of ureteral obstruction secondary to pelvic malignancies〔J〕. Endourol, 1999, 13 (7): 521-524.

6 方银忠，叶定伟，陈鸣之，等. 宫颈癌相关的上尿路梗阻的处理〔J〕. 中国肿瘤临床，2004，31 (12)：690-692.

(本文编辑：王溪婷)

护理干预对超重与肥胖青少年减肥的影响

陈维婵 方苗 黄海滨 林少芬 李爱军

【摘 要】 **目的** 根据儿童青少年的生长发育特点，从护理干预角度，建立超重与肥胖青少年合理饮食和良好生活方式，控制体重，从而有效控制儿童青少年超重及肥胖的发展。**方法** 对汕头市 8 所学校 27 196 名学生采取抽样法、询问法、问卷调查法；并从中抽取 1192 名超重及肥胖青少年做饮食结构及生活方式评估，根据评估结果进行健康教育、有氧运动、合理饮食、心理行为改善、医务监督等综合性干预为主要内容。**结果** 护理干预 2 年后超重及肥胖青少年行为、形态差异有显著性意义($P<0.05$)。**结论** 护理干预对改善超重与肥胖青少年饮食结构和生活方式，对控制体重是至关重要的。

【关键词】 护理干预； 儿童肥胖病； 超重与肥胖； 青少年学生

中图分类号：R473.5 文献标识码：B

我国有关调查资料表明，中小学生肥胖率上升速度很快，大城市尤为突出，其他国家也呈类似的上升趋势[1]。本文于 2008 年 1 月至 2009 年 12 月对汕头市 8 所学校展开了调查超重及肥胖进行护理干预效果的研究。现报告如下。

DOI：10.3760/cma.j.issn.1673-4351.2010.10.025

作者单位：515031 广东汕头，中山大学附属汕头医院汕头市中心医院（陈维婵，方苗，黄海滨，李爱军）；广东汕头第三中学（林少芬）

1 对象与方法

1.1 对象

汕头市 8 所学校 27 196 名学生，采取抽样法、询问法、问卷调查法；并从中抽取 1 192 名超重及肥胖青少年为对象，男女各半，年龄 12~18 岁，按对照匹配原则分为干预组和对照组各 596 名。

1.2 方法

万方数据

1.2.1　登记建档，为每个学生建立一本干预效果观察手册，包括学生的班级、姓名、性别、年龄。

1.2.2　干预方法

采用护理综合干预措施为主要内容，以体重指数 BMI≥24 kg/m² 界为超重，≥28 kg/m² 为肥胖[2,3]，儿童采用 WHO 推荐的身高标准体重法，即≥身高标准体重 120% 为肥胖[4]，经过医学筛选排除内分泌疾病。干预内容：（1）健康教育，依靠学校校医室、团委会、学生会、学校广播室等部门宣传合理膳食控制体重，科学锻炼，预防慢性病等知识，提高青少年对肥胖的认识，观看健康保健录象，开通校园网络咨询，宣传《中国居民膳食指南》和肥胖防治知识。（2）有氧运动（如慢跑、球类运动等项目），每次训练 40～60 min，运动频率为每周 5～7 次，运动强度达到本人最高心率 70～80%。（儿童青少年均最大心率以 200 次/min）。（3）合理饮食，评估每位肥胖者饮食习惯及结构，找出各自的危险因子，制订相应的干预措施，指导肥胖者改变晚餐丰盛和夜宵的习惯，减少进食动物脂肪及高热量油炸食物，保证每日摄人新鲜蔬菜和水果，少饮含糖水多的饮料。（4）心理行为和生活方式改善，从肥胖者体重指数，运动方式及运动时间，心理因数等方面进行评估，发放抑郁自量表 SDS[5] 进行测评，评价肥胖者心理失衡状态及评定肥胖者焦虑主观感受，帮助肥胖者执行减肥，健身计划，探索失衡肥胖者的心理状况，根据思想情绪，性格特点等心理要求，帮助他们解除心理负担，培养有利减肥的良好心态和饮食习惯。（5）医务监督，定期测量体重、BMI、腰围、胸围等变化，同时应注意整个过程观察。

1.3　统计学分析

所有数据均采用 SPSS 10.0 统计软件包进行处理，采用配对 t 检验和方差分析。

2　结果

2.1　根据体重身高及体重指数，符合儿童肥胖病诊断标准为 2 212 名。按年龄、性别分组并进行统计学处理见表 1。从表中可看出儿童肥胖病发病率随着年龄增加而上升，6～11 岁的儿童为 7.28%，12～18 岁时上升 8.3%，在这些 12～18 岁儿童青少年中，肥胖在男性中更为常见，（男性和女性分别为 13.5% 和 6.2%）。

表 1　8 所学校学生儿童肥胖病患病率

年龄(岁)	性别	人数	儿童肥胖病	患病率(%)
6～11	男	8 520	620	7.28
	女	6 706	402	6
12～18	男	6 150	830	13.5
	女	5 820	360	6.2

2.2　干预前后超重与肥胖青少年学生不合理饮食及不良生活方式的变化比较见表 2。

2.3　干预前后超重及肥胖青少年身体形态变化比较

经过 2 年的干预后超重与肥胖青少年学生在体重、BMI、腰围、胸围均有显著性差异（$P<0.05$）。而对照组体重、BMI、腰围、胸围等指标呈增长趋势见表 3。

表 2　护理干预前后超重及肥胖青少年不合理饮食及不良生活方式的变化比较〔n（%）〕

项目	例数	干预前	干预后
不合理饮食	1 192	954(80.0)	238(20.0)
缺乏运动(<30 min/d)	1 192	810(67.9)	36(3.0)
心理失衡	1 192	739(62.0)	173(14.5)
下课继续学习做功课	1 192	516(43.3)	436(36.6)

表 3　干预前后超重与肥胖青少年学生身体形态变化的比较（$\bar{x}\pm s$）

项目	对照组（n=596）		干预组(n=596)	
	干预前	干预后	干预前	干预后
身高(cm)	159.44±3.46	160.33±2.34	159.32±3.76	160.87±3.94①
体重(kg)	70.36±2.23	72.07±2.12	70.12±3.41	64.13±2.31①
BMI	26.32±1.46	27.62±3.03	26.73±1.61	24.36±2.2①
腰围(cm)	81.31±2.32	81.71±1.67	81.47±2.32	76.37±0.39①
胸围(cm)	88.02±1.02	88.63±2.31	88.54±1.06	84.03±0.18①

注：①干预组与对照组对应时间的比较 $P<0.05$

3　讨论

3.1　合理饮食和有氧运动是控制和预防肥胖的重要手段

表 2 显示，不合理饮食行为，体力活动减少和遗传是肥胖的主要原因，因此从饮食结构改善着手，积极限制高脂饮食的摄人，降低脂肪热能比，提高饮食纤维和营养素的摄人量，以饮食指南为准，指导肥胖者制定健康食谱。

坚持有氧运动的持续性是控制和预防肥胖发生至关重要因素。对肥胖者而言有氧运动是客观生活中最活跃，也是最重要因素之一[6]。干预前后发现，肥胖者都喜欢上体育课和参加锻炼，完全不想参加很少，但因学习任务重，下课后继续看书做作业仍没明显改善，因此学校和家庭的教育应重视这一问题，科学安排学生的学习和作息时间，积极推广宣传科学锻炼身体方法，促进学生每天运动持久性，超重及肥胖高发年龄段都在 12～18 岁之间见表 1，而此时正是青少年体格发育期，及早地预防是极其重要。

3.2　加强医务监督教育，培养个人良好生活方式

肥胖与个人的行为密不可分，但个人行为的改变离不开所在学校家庭的影响和环境的支持，加强医务监督，重视对肥胖青少年人群的管理，把健康食谱与运动处方的制度列人学校卫生工作的重要内容，根据青少年的心理情况，还可以将基本情况相似的肥胖者编成一组，制定与相适应的食谱和运动方案，以年级、班级为单位，起到同伴相互监督，相互激励作用，达到共同改变不良生活习惯的目的。

3.3　心理干预对肥胖者引起心理行为障碍的影响

肥胖者心理造成的损害，国内外已有不少文献报道，主要表现为肥胖儿童缺乏自信心，受歧视，伙伴关系不良，自我感觉差、害羞、畏惧、性情暴躁等。在心理干预过程中我们采取问卷调查法了解肥胖者的心理问题及原因，及时给予

万方数据

鼓励，消除儿童的心理障碍，使其心理状态恢复正常。

本文超重与肥胖青少年体重、BMI、胸围、腰围等指标都发生很大变化，说明正在接受干预的青少年控制体重的意识明显增强，对他们造成的严重心理损害，已引起社会、学校及家长的重视，同时把心理治疗也纳入在其中。总之，护理干预对改善青少年超重及肥胖至关重要。

参考文献

1 谭琪，徐勇．中国儿童青少年超重发展趋势分析〔J〕．中国学校卫生，2003，24（12）：609－610.
2 季成叶，孙军玲．中国学龄青少年体重指数地域与人群分布差异的分析〔J〕．中华儿科杂志，2004，42（5）：328－332.
3 傅兰英，盛伟，王小引，等．青少年女学生综合减肥方法研究〔J〕．中国学校卫生，2005，26（5）：353－355
4 国际生命科学学会中国肥胖工作组．中国学生超重、肥胖BMI筛选标准〔G〕．北京：中国儿童青少年肥胖问题研讨会，2003：1－263.
5 张宁．医学心理学〔M〕．南京：东南大学出版社，2001：104－106.
6 傅兰英，姬英涛，姬成茂，等．运动处方对女大学生减肥及健康状况影响研究〔J〕．中国学校卫生，2004，25（5）：329－540.

（本文编辑：王溪婷）

食管癌术后并发吸入性肺炎的治疗和护理

邓攀　刘胜中　高虹　丛伟

【摘　要】 目的　总结食管癌术后并发吸入性肺炎的治疗和护理经验。方法　对我科12例食管癌术后并发吸入性肺炎患者的临床治疗进行回顾性分析。结果　本组治愈7例其中包括2例发生ARDS的患者；死亡5例，其中ARDS所致的严重缺氧1例，并发吻合口瘘导致的严重感染及MODS 4例。结论　治疗中要注重气道管理与呼吸支持，科学地使用呼吸机支持呼吸；合理、及时、有效地使用敏感抗生素；维护重要脏器的功能，尤其是肺脏功能的保护。护理中要避免在机械通气治疗过程中增加误吸的发生，警惕吻合口瘘的发生；重视生命体征观察，并注重与患者的交流与沟通。

【关键词】 吸入性肺炎；　食管癌；　并发症；　护理
中图分类号：R473.6　　文献标识码：B

食管癌术后并发吸入性肺炎并不少见，严重者常演变成急性呼吸窘迫综合征（ARDS）[1]。其原因是多方面的，该病病情复杂，预后差，死亡率高。我科自2000年1月至2009年12月共有12例，现就其治疗和护理经验报告如下。

1　资料与方法

1.1　一般资料

本组12例，男9例，女3例；年龄45～77岁，平均65.2岁；上段食管癌5例，中段4例，下段3例。均行食管癌根治术，其中经左胸一切口行弓下机械吻合术4例，弓后胸顶机械吻合术5例，经左胸及颈部二切口行颈部吻合术2例，经右胸、颈部及腹部三切口行颈部吻合术1例。合并急性胃扩张者1例，吻合口瘘者2例，胸腔积液2例。术后所有患者均有明确的呕吐及误吸史。确诊时间为术后3～12 d，其中7例发生ARDS。

1.2　治疗方法

1.2.1　一般疗法　麻醉过程中应定期吸痰，拔管前彻底吸净口咽部分泌物及潴留液；在无痛状态下鼓励、指导病人尽力咳痰，以防蓄积；变换体位，保持30～60°斜卧位或侧斜卧位、半卧位，利于痰液引流和防止、减少胃返流液的吸入；合理饮食，少食多餐，进无刺激性固态食物间隔2～3 h，饭后1～2 h内尽量保持坐卧位或站立位。

1.2.2　气道管理与呼吸支持　保证呼吸道通畅，科学地使用呼吸机支持呼吸，对病情的控制和顺利恢复至关重要。气管插管气囊应定时放气减压，得以改善黏膜血运，放气前吸净囊上口、咽、喉部的滞留液；气管插管留置时间超过5～7 d者，应行气管切开插管，便于吸痰和咽喉部休息、功能恢复，用粗细适中的乳胶管吸痰，宜轻柔、快捷，尽可能达到最大深度；吸痰管应多方向的变换位置，更好地吸出左、右支气管内分泌物。无自主呼吸、呼吸能力差、呼吸肌疲劳的病人，应及时使用呼吸机辅助呼吸，呼吸模式为APC或Simv，给予适当的PEEP4～15 cm H_2O，多主张高频、小潮气量通气，可以减轻肺损伤；各种呼吸参数的变更，须根据病人状态随时调整，原则上宜小幅度、缓慢进行，不要过快、大范围地变化；使用呼吸机时应定时复查血气、电解质等。

1.2.3　合理应用抗生素　及时、有效地使用有效抗生素是治疗的关键。根据痰菌培养＋药敏试验来选择敏感抗生素，是控制感染的基本原则。

1.2.4　肺脏的保护　除尽量避免其他因素对肺脏的损伤外，采取必要的措施对呼吸道黏膜、肺泡细胞进行保护。定期给予雾化吸人，每次5～15 min，每2～6 h 1次，其作用基本同湿化液，但更均匀、广泛接触呼吸道黏膜及肺泡，效果更佳；静脉用药沐舒坦，成人最大量可达1 000 mg/d，分次给人；口服富露施等药物，对保护肺细胞，改善其周围环境及稳定

DOI：10.3760/cma.j.issn.1673－4351.2010.10.026
作者单位：610072 四川成都，四川省医学科学院·四川省人民医院医胸外科

万方数据

护理干预对超重与肥胖青少年减肥的影响

作者：　陈维婵，　方苗，　黄海滨，　林少芬，　李爱军
作者单位：　陈维婵,方苗,黄海滨,李爱军(中山大学附属汕头医院汕头市中心医院,广东汕头,515031)，林少芬(广东汕头第三中学)
刊名：　国际护理学杂志 ISTIC
英文刊名：　INTERNATIONAL JOURNAL OF NURSING
年，卷(期)：　2010,29(10)
被引用次数：　2次

参考文献(6条)

1. 谭琪;徐勇 中国儿童青少年超重发展趋势分析[期刊论文]-中国学校卫生 2003(12)

2. 季成叶;孙军玲 中国学龄青少年体重指数地域与人群分布差异的分析[期刊论文]-中华儿科杂志 2004(05)

3. 傅兰英;盛伟;王小引 青少年女学生综合减肥方法研究[期刊论文]-中国学校卫生 2005(05)

4. 国际生命科学学会中国肥胖工作组 中国学生超重、肥胖BMI筛选标准 2003

5. 张宁 医学心理学 2001

6. 傅兰英;姬英涛;姬成茂 运动处方对女大学生减肥及健康状况影响研究[期刊论文]-中国学校卫生 2004(05)

本文读者也读过(9条)

1. 孙璞.夏淑敏.SUN Pu.XIA Shu-min 对北京市肥胖青少年的现状调查与分析[期刊论文]-中国体育科技 2001,37(3)

2. 郭晓英 儿童单纯肥胖症治疗方法[期刊论文]-中外健康文摘2011,08(4)

3. 唐君 强化心理与饮食干预对肥胖型糖尿病患者的影响[期刊论文]-中国现代药物应用2010,4(9)

4. Echo 减肥从心理操开始[期刊论文]-中国保健营养2010(3)

5. 吴瑛 营养运动心理综合治疗重度肥胖症1例[期刊论文]-河北医学2010,16(12)

6. 蔡德培 儿童肥胖症[会议论文]-2003

7. 魏艳军.李亚平 中青年肥胖相关性高血压患者的心理问题及护理措施[期刊论文]-中国医药导刊2010,12(6)

8. 黄卡 心理-饮食-运动干预治疗儿童单纯性肥胖症的效果观察[期刊论文]-中国基层医药2011,18(2)

9. 黄萍.龙朝杰 儿童肥胖症的综合护理干预[期刊论文]-当代护士（专科版）2010(9)

引证文献(2条)

1. 李品群.张芬芳.杜马玲.张可渴 护理干预对股骨头缺血性坏死介入治疗后功能康复的影响[期刊论文]-国际护理学杂志 2011(10)

2. 张华.庞礼娟.宋学勤 心理行为干预在降低抗精神病药源性肥胖中的作用[期刊论文]-医学与哲学 2012(4)

本文链接：http://d.wanfangdata.com.cn/Periodical_gwyx-hlxfc201010025.aspx
