# Supplementary material for: Systematic review and meta-analysis of school-based obesity interventions in mainland China
Source: PLoS One. 2017 Sep 14;12(9):e0184704. doi: 10.1371/journal.pone.0184704 (PMC5598996; doi:10.1371/journal.pone.0184704)
Supplement: S1 Dataset — (ZIP) [file pone.0184704.s007.zip › S1_dataset/76库/70.pdf]

营养教育对学生饮食行为及营养状况的影响①

赵伟明　李吴萍　陶秀娟　杨静芳②　刘贺荣　金　维
宁夏医科大学公共卫生学院（宁夏　银川）　750004

中国图书分类号　R173　文献标识码　C　文章编号　1001-4411（2011）12-1780-03

【摘　要】　目的：评价营养教育对中小学生饮食行为、营养状况的影响，为有效开展营养教育提供科学依据。方法：将学生分为干预组和对照组，对干预组学生采用多种形式进行为期1年的营养教育。结果：干预组学生营养教育后营养知识知晓率明显提高，平均知晓率由52.2%提高到75.3%，差异有统计学意义（$P<0.01$）；每天吃早点的人数增加，从干预前的51.0%提高到干预后的71.6%，早点的质量有所改变，经常喝牛奶、吃蛋类的人数明显增多，且显著高于干预前（$P<0.01$）；超重、肥胖率干预前分别为5.2%和3.2%，干预后为5.9%和1.6%，肥胖率有所下降，差异有统计学意义（$P<0.05$）；贫血患病率由干预前的6.9%降到6.0%，但差异无统计学意义。结论：营养教育对提高营养知识、促进学生饮食行为的改变切实有效。

【关键词】　营养教育　中小学生　体质指数　贫血

Effect of nutritional education on dietary behaviors and nutritional state of students

ZHAO Wei－Ming，LI Wu－Ping，TAO Xiu－Juan et al. School of Public Health，Ningxia Medical University，Yinchuan 750004，Ningxia，China

〔**Abstract**〕　**Objective:** To evaluate the effect of nutritional education on dietary behaviors and nutritional state of students，provide a scientific basis for carrying out nutritional education effectively. **Methods:** The students were divided into intervention group and control group，the students in intervention group received many types of nutritional education for one year. **Results:** After nutritional education，the awareness rate of nutritional knowledge in intervention group increased significantly，the average awareness rate of nutritional knowledge increased from 52.2% to 75.3%，there was significant difference before and after nutritional education（$P<0.01$）；the number of students having breakfast every day increased from 51.0%（before nutritional education）to 71.6%（after nutritional education），the quality of breakfast changed，the number of children drinking milk and eating eggs frequently after nutritional education increased significantly，which was higher than that before nutritional education（$P<0.01$）；the rates of overweight and obesity before nutritional education were 5.2% and 3.2%，respectively，the rates of overweight and obesity after nutritional education were 5.9% and 1.6%，respectively；the rate of obesity decreased significantly after nutritional education（$P<0.05$）. **Conclusion:** Nutritional education is an effective method to increase nutritional knowledge and promote the changes of dietary behaviors of students.

〔**Key words**〕　Nutritional education；Young students；Body mass index；Anemia

中小学生处于生长发育的重要阶段，合理的营养膳食不仅能促进其健康成长，也是预防成人期营养相关慢性疾病的有效手段。营养教育是改善中小学生营养状况的一种有效、经济的干预措施[1]。2007年11月～2009年3月对宁夏吴忠地区1 718名中小学生进行了为期1年多的营养干预研究，现将结果报告如下。

1　资料与方法

1.1　干预对象　在吴忠地区共选8所学校，其中中学4所（初二、初三）抽取部分班级、小学四所（四、五、六年级）抽取部分班级共1 718人，以学校为单位将学生分为干预组和对照组。自行设计调查表，调查内容包括营养知识、早餐食用情况和饮食行为。调查表由班主任在上课时下发并当场回收。

1.2　干预方法　只对干预组学生进行教育，主要采取的方法是发放宣传手册、年画、专题讲座、黑板报或手抄小报等。于干预前及干预1年后（2009年3月）对学生再次进行问卷调查、身高体重测量和采血分析。体格检查按照《全国学生体质健康状况研究工作手册》要求测定身高、体重，根据体质指数判断儿童的营养状况[2]。血红蛋白测定一律取左手无名指末梢血10 μl，用氰化高铁法测定血红蛋白含量，按WHO制定的贫血诊断标准进行评价：7～14岁低于120 g/L、>14岁男低于130 g/L、女低于120 g/L为贫血。

1.3　资料统计方法　剔除无效问卷，在有效答卷中，部分被试者有部分题目未答全，在进行与该项目有关的统计分析时作缺失值。用SPSS 11.5统计软件进行统计分析，频数之间的比较采用χ^2检验。

2　结果

2.1　干预前后营养知识知晓率比较　干预后学生的营养知识知晓率明显提高，特别是有关钙、铁、维生素A、膳食宝塔

①2007年宁夏科技厅科技攻关项目〔072164024〕
②宁夏吴忠市卫生局

?1994-2014 China Academic Journal Electronic Publishing House. All rights reserved.　http://www.cnki.net

等方面的知识提高比较明显，平均知晓率由 52.2% 提高到 75.3%，差异有统计学意义（$P<0.01$）。见表 1。

2.2　干预前后饮食行为比较　干预后学生每天吃早点的人数增加，从干预前的 51.0% 提高到干预后的 71.6%，早点的质量有所改变，经常喝牛奶、吃蛋类的人数明显增多，且显著高于干预前（$P<0.01$）。见表 2、表 3。

表 1　干预前后学生营养知识知晓率〔n（%）〕

内容	干预前（n=1 718）		干预后（n=1 585）	
	干预组（n=887）答对人数	对照组（n=831）答对人数	干预组（n=831）答对人数	对照组（n=754）答对人数
缺钙导致疾病	477(53.8)	444(53.4)	653(78.6)	415(55.0)
含钙丰富的食物	567(63.9)	559(67.3)	737(88.7)	532(70.6)
含维生素 A 的食物	177(20.0)	181(21.8)	450(54.2)	199(26.4)
含维生素 C 的食物	805(90.8)	759(91.3)	798(96.0)	696(92.3)
富含蛋白质的食物	727(82.0)	683(82.2)	768(92.4)	614(81.4)
贫血有关因素	404(45.5)	345(41.5)	572(68.8)	328(43.5)
哪种食物预防贫血	388(43.7)	398(47.9)	544(65.5)	370(49.1)
吃糖对身体的影响	823(92.8)	755(90.9)	773(93.0)	683(90.6)
晒太阳预防疾病	354(39.9)	336(40.4)	472(56.8)	312(41.4)
缺碘导致疾病	656(74.0)	618(74.4)	693(83.4)	541(71.8)
富含碘的食物	599(67.6)	611(73.5)	660(79.4)	537(71.2)
米面富含的营养素	106(12.0)	76(9.1)	345(41.5)	114(15.1)
人体热能的来源	87(9.8)	52(6.3)	506(60.9)	75(9.9)
听说过膳食宝塔	310(35.0)	245(29.5)	791(95.2)	382(50.7)
平均知晓率	463(52.2)	433(52.1)	626(75.3)①②	414(54.9)

注：①与干预前比 $P<0.01$；②与对照组相比 $P<0.01$。

表 2　干预前后学生早餐食用情况

内容	干预前				干预后			
	干预组		对照组		干预组		对照组	
	n	%	n	%	n	%	n	%
每天吃早餐	452	51.0	468	56.3	595	71.6①②	415	55.1
有时候吃	404	45.5	327	39.4	217	26.1	309	40.9
不吃	31	3.5	36	4.3	19	2.3	30	4.0

注：①与干预前比较 $P<0.01$；②与对照组比较 $P<0.01$。

表 3　干预前后学生早餐经常食用的食物频率

内容	干预前				干预后			
	干预组		对照组		干预组		对照组	
	n	%	n	%	n	%	n	%
谷类	646	72.8	593	71.4	597	71.8	564	74.8
牛奶	373	42.1	342	41.1	451	54.3①	291	38.6
肉类	34	3.8	31	3.7	27	3.2	31	4.1
鸡蛋	248	28.0	143	17.2	308	37.1②③	146	19.4
豆浆	139	15.7	131	15.8	120	14.4	129	17.1

注：①与干预前比较 $P<0.01$；②与干预前比较 $P<0.01$；③与对照组比较 $P<0.01$。

2.3　干预前后超重、肥胖率比较　超重、肥胖率干预前分别为 5.2% 和 3.2%，干预后为 5.9% 和 1.6%，肥胖有所下降，差异有统计学意义。对照组实验前为 6.6% 和 2.0%，实验后为 7.6% 和 2.1%，差异无统计学意义。见表 4。

2.4　干预前后贫血患病率比较　干预后学生贫血患病率稍有下降，由干预前的 6.9% 降到 6.0%，但差异无统计学意义。见表 5。

表 4　干预前后学生超重、肥胖情况〔n（%）〕

类别	干预前		干预后	
	干预组（n=887）	对照组（n=831）	干预组（n=831）	对照组（n=754）
超重	46(5.2)	55(6.6)	49(5.9)	57(7.6)
肥胖	28(3.2)	17(2.0)	13(1.6)①	16(2.1)
合计	74(8.3)	72(8.7)	62(7.5)	73(9.7)

注：①与干预前比较 $P<0.05$。

表 5　干预前后学生贫血患病率

类别	干预组			对照组		
	调查人数	贫血人数	%	调查人数	贫血人数	%
干预前	449	31	6.9	431	55	12.8
干预后	415	25	6.0	380	31	8.2

3　讨论

营养教育作为一种大众营养改善与促进手段，在世界范

?1994-2014 China Academic Journal Electronic Publishing House. All rights reserved.　http://www.cnki.net

围内日益受到营养学界的重视和推崇，也是指导人们科学合理地选择平衡膳食及建立健康生活方式的重要途径。健康饮食行为的转变过程是由知识→态度→行为的转变，只有具备一定的知识和良好的接受教育态度，才有可能实现这个转变。中小学生正处于身体发育和生活习惯、健康行为形成的重要时期，学生营养知识的知晓，能够使他们将来养成健康的生活方式[3]。本次研究结果显示，中小学生对中国居民膳食指南的认识和营养知识水平都有了很大的提高，与相关研究结果[4~6]一致，说明营养教育对提高营养知识切实有效。

早餐是一天能量和营养素的重要来源，每天吃早餐是世界卫生组织倡导的一种促进健康的行为[3]。然而，早餐也是最容易被忽视的一餐，经常不吃早餐会引起能量和营养素摄入不足，乃至营养缺乏，长期营养不足还会影响生长发育[7]。营养早餐应该包括谷类、肉蛋类、奶类、蔬菜等四大类食物，营养早餐可以促进小学生的生长发育，提高大脑作业能力。本次实验干预结果表明通过营养教育使中小学生传统的早餐理念得到了转变，每天吃早餐的人数明显增多，早餐的质量有所改进，牛奶和蛋类在早餐中的食用频率明显提高。

良好的饮食习惯和健康的生活方式是预防青少年营养失调发生的有效措施。虽然研究结果显示，学生超重、肥胖和贫血的患病率并无明显变化，由此也说明要获得更为满意的效果，最终实现知识获得、态度转变到行为实践的飞跃，还需要长期的、广泛的开展营养教育工作。营养教育必须长期坚持，而且应该学校、家庭、个人都行动起来，共同努力才有可能收到预期的效果。

4 参考文献

1 李斯特．国外营养教育与管理〔J〕．中国食物与营养，2004，5（1）：4

2 国际生命科学学会中国肥胖工作组．中国学生超重、肥胖 BMI 筛查标准〔M〕．北京：中国儿童青少年肥胖问题研讨会，2003：1~26

3 赵文君．小学生营养知识和早餐行为干预效果评价〔A〕．中国健康教育，2006，22（7）：551

4 张艳丽，王守英，谷玉梅．营养教育对中学生健康知识态度行为的影响效果评价〔J〕．中国学校卫生，2007，28（8）：689

5 赵丽云，李 丹，郝 宏 *et al.* 广东、吉林、四川、湖北四省 0~6 岁儿童有关营养教育前后知信行的比较〔J〕．中国健康教育，2003，19（1）：4

6 虞 华，余 昭，陈 川 *et al.* “以营养教育为重点的学校健康促进”效果评价〔J〕．中国健康教育，2003，19（6）：434

7 高树军，马冠生，翟凤英 *et al.* 我国中小学生早餐食物种类研究〔J〕．中国学校卫生，2001，22（3）：196

（2011-01-04 收稿）
〔编校 徐 强〕

健商培训在产前教育中的应用

丁郭平[①] 常德职业技术学院护理系（湖南 常德） 415000

中国图书分类号 R193 文献标识码 A 文章编号 1001-4411（2011）12-1782-03

【摘 要】 目的：研究健商理念在产前健康教育中的应用效果，为产前教育的临床实践提供相关数据，为有针对性地采取干预措施提供科学依据。方法：采用平行随机对照临床试验研究，收集 120 例孕妇的社会人口学资料，随机分配为接受健商培训的试验组和未接受健商培训的对照组。结果：两组孕妇基线资料一致，具有可比性。健商培训后，试验组孕妇的健康知识、自然生产自我效能得分明显高于对照组，且明显高于培训前，对照组无改变。试验组孕妇焦虑水平明显低于对照组。两组产妇实际分娩方式比较，试验组的剖宫产率显著低于对照组。结论：产前教育中加入系统的、有计划的、有针对性的健商培训，增强了孕妇的产前认知、分娩认知，促进了孕妇健康行为改变，提高了自然分娩率，具有一定的临床应用价值。

【关键词】 孕产妇 健商 产前教育 剖宫产

妊娠分娩是一种生理现象，但分娩对于产妇确实是一种持久而强烈的应激原[1]。在我国，现在至少 50% 以上的产妇放弃自然分娩方式而选择剖宫产[2]，中国有些地区的剖宫产率已达 50%~80%，无医疗指征但孕妇及家属要求的剖宫产增长迅速[3]。造成这一现象的原因是多样的，其中妇女的主观意愿起到了重要的作用[4]。

1 资料与方法

1.1 研究设计 该试验设计方法为平行随机对照临床试验研究。两组孕妇的年龄、孕周、孕次、文化程度、职业构成、经济状况、婚姻状况、医疗费用支付方式、希望分娩方式等比较差异无统计学意义（$P>0.05$），资料具有可比性。健商培训前对试验组和对照组进行健商水平、健康知识、自然生产自我效能、焦虑等情况的调查。试验组分别于孕 28、30、32、34、36、37、38 周接受 7 次系统加入健商培训的产前教育。38 周时进行健康知识、自然生产自我效能、焦虑等情况的调查。分娩后，进行实际分娩情况的调查。

1.2 资料收集工具 包括孕妇基本情况问卷、谢华真健康知识得分问卷、产妇自然生产自我效能量表、状态－特质焦虑问卷、实际分娩情况表。

①中南大学护理学院硕士研究生

?1994-2014 China Academic Journal Electronic Publishing House. All rights reserved. http://www.cnki.net
