# Supplementary material for: Systematic review and meta-analysis of school-based obesity interventions in mainland China
Source: PLoS One. 2017 Sep 14;12(9):e0184704. doi: 10.1371/journal.pone.0184704 (PMC5598996; doi:10.1371/journal.pone.0184704)
Supplement: S1 Dataset — (ZIP) [file pone.0184704.s007.zip › S1_dataset/76库/71.pdf]

DOI:10.16038/j.1000-6710.2008.03.025
中国运动医学杂志2008年5月第27卷第3期 Chin J Sports Med, May 2008, Vol.27, No.3

基于学校的有组织的运动干预对肥胖青少年BMI和糖、脂代谢的影响

刘文[1] 常翠青[1] 赵小倩[2] 姜雪[1] 陈志民[1] 谢岚[1] 于长隆[1]
1 北京大学第三医院运动医学研究所(北京 100083) 2 北京市八一中学

摘要 目的:探讨基于学校的有组织的运动干预对青春期早期肥胖青少年体重指数(BMI)和糖脂代谢的影响及其量效关系。方法:12~14岁肥胖青少年49名,按照BMI和个人意愿分为运动组(25名)和对照组(24名),运动组进行有组织的9个月、运动量两阶段(0~3个月,大运动量;4~9个月,小运动量)递减的运动干预。分别于运动前、3个月末和9个月末进行体格测试和空腹血脂、血糖、胰岛素水平和胰岛素抵抗指数检测。结果:(1)运动组男生和女生BMI在干预期末比干预前分别下降了0.3和0.5,对照组男生和女生BMI分别上升0.2和0.3。(2)运动组血清甘油三酯(TG)水平在第3个月末和9个月末与运动前相比均显著下降($P<0.05$),对照组血清TG水平显著上升($P<0.05$);运动组血清高密度脂蛋白胆固醇(HDL-C)在整个干预期间无显著性变化,而对照组血清HDL-C水平显著下降($P<0.01$),且低于运动组水平($P<0.01$)。(3)运动组血糖($P<0.01$)、血清胰岛素水平($P<0.05$)、胰岛素抵抗指数(HOMA-IR)($P<0.05$)在运动干预后均显著下降;对照组血糖显著升高($P<0.05$),血清胰岛素水平和HOMA-IR无显著性变化。(4)运动组高甘油三酯血症检出率和空腹血糖受损率在3个月末分别下降了66.7%和100%,并持续维持到9个月运动干预末;对照组则分别上升了2.25倍和1.98倍。结论:基于学校的有组织的运动干预可以有效地预防和延缓肥胖青少年肥胖的进一步发展,改善糖脂代谢,降低高甘油三酯血症和空腹血糖受损发生率。

关键词 运动;肥胖青少年;血脂;血糖;体能;减体重

Effect of School-Based Supervised Exercise Intervention on Body Mass Index and Glucolipid Metabolism in Chinese Obese Adolescents

Liu Wen[1], Chang Cuiqing[1], Zhao Xiaoqian[2], Jiang Xue[1], Chen Zhimin[1], Xie Lan[1], Yu Changlong[1]
1 Institute of Sports Medicine, Third Hospital, Peking University, Beijing, China 100083
2 Ba Yi Middle School, Beijing, China

Abstract ***Objective*** To study the effect of school based supervised exercise on the body mass index (BMI) and glucolipid metabolism in Chinese obese adolescents. ***Methods*** Forty nine obese adolescents (aged 12yrs to 14yrs) were divided into exercise group and control group. There was no difference in BMI between the two groups. The exercise group underwent a 9-month exercise intervention supervised by a PE teacher. The exercise volume in the first 3 months was higher than that in the last 6 months. The control group exercised freely without supervision. Anthropometry and fasting serum lipids, glucose, insulin and HOMA-IR were measured at month 0, 3, 9 of the intervention. ***Results*** (1)The BMI of boys and girls decreased by 0.3 and 0.5 respectively in exercise group as compared with the pre-intervention, whereas the BMI increased by 0.2 and 0.3 respectively in control group. (2)The serum TG level significantly decreased ($P<0.05$) in exercise group at the end of the 3rd and 9th month, while increased significantly in control group as compared with the pre-intervention ($P<0.05$). The serum HDL-C level remained unchanged in exercise group during the whole intervention, while decreased significantly in control group ($P<0.01$), and remained lower than in exercise group even after the intervention ($P<0.01$). (3)As compared with the pre-intervention, the fasting serum glucose level of post-intervention decreased in exercise group ($P<0.01$), but increased in control group ($P<0.05$). The insulin level and HOMA-IR decreased significantly and constantly in exercise group during the intervention ($P<0.05$), while there was no significant change in control group. (4)The hypertriglyceridemia rate and impaired fasting serum glucose rate in exercise group decreased by 66.7% and 100% respectively by the end of the 3rd month and kept the same level until the 9th month, and those in control group increased by 2.25 and 1.98 times respectively after the intervention. ***Conclusion*** School-based supervised

基金项目:北京大学医学部"985工程"二期建设项目(项目编号:985-2-028-24)资助
通讯作者:常翠青, Email: cuiqingchang@yahoo.com.cn

?1994-2015 China Academic Journal Electronic Publishing House. All rights reserved. http://www.cnki.net

exercise intervention could effectively prevent and delay the deterioration of obesity among the over-weighted adolescents and improve their glucolipid metabolism.

Key words　exercise, obese adolescents, serum lipids, serum glucose, fitness, weight loss

青少年肥胖常常伴随着胰岛素抵抗[1]，血压和血脂异常，将来发生心血管疾病和2型糖尿病的危险性大大增加[2]。引起儿童青少年肥胖的主要原因是体力活动不足和能量摄入过多。对于预防和治疗青少年肥胖，目前公认的科学有效的方法是合理膳食营养，适量运动和纠正不良行为。研究表明，饮食控制和大运动量（每天运动2.5小时以上）运动干预可以改善儿童青少年肥胖和降低血糖、血脂，改善胰岛素抵抗等代谢综合征症候群[3]。但较小运动量对肥胖儿童青少年健康的影响还需要进一步研究。儿童青少年一天中大部分时间在学校度过，学校除了授课外，也应当是一个培养学生良好生活习惯的场所，而且学校的组织和纪律性强的统一管理模式和统一规律的集体生活使运动干预活动更容易开展，也为以学校为基础的运动干预活动提供了保障。故本研究从运动的有效性、可行性和可持续性发展方面考虑，采用基于学校的运动量阶段式递减的干预方式，探讨有组织的运动对肥胖儿童青少年健康的影响及其量效关系，旨在帮助肥胖儿童青少年逐步形成主动运动习惯，培养受益终生的健康生活方式。

1　对象与方法

1.1　研究对象

通过体检筛选出12～14岁汉族肥胖青少年65名（男生47名，女生18名），根据BMI和个人意愿分为两组：运动组33名（男生24名，女生9名），对照组32名（男生23名，女生9名），两组BMI水平无显著性差异。最后坚持完成实验的运动组25名（男18，女7），对照组24名（男18，女6）。实验前运动组和对照组年龄、身高、体重和BMI均无显著性差异（表1）。

表1　受试者一般情况

Table 1　Characteristics of subjects

组别 groups	n	年龄（yrs） age	身高（cm） height	体重（kg） weight	BMI
运动组 exercise					
男 M	18	12.5±0.6	165.8±9.5	76.4±15.1	27.5±3.4
女 F	7	12.8±1.2	161.7±3.2	69.1±6.7	26.5±3.4
总计 M+F	25	12.6±0.8	164.7±8.4	74.3±13.5	27.2±3.4
对照组 control					
男 M	18	12.3±0.8	162.0±6.9	72.1±13.8	27.3±3.5
女 F	6	12.9±0.4	159.3±3.3	69.1±6.4	27.1±3.1
总计 M+F	24	12.2±0.1	161.3±6.2	71.3±12.3	27.2±3.3

肥胖的判断标准采用中国学龄儿童青少年超重、肥胖筛查体重指数值分类标准[4]。本研究得到北京大学医学部伦理委员会批准，所有受试者及其家长均签署了知情同意书。

1.2　方法

实验为期9个月。采用健康教育和开放式有组织强化运动方式。运动组进行健康教育（内容包括平衡膳食，合理营养，纠正不良膳食行为和适量运动，每3个月1次）和有组织的运动训练。对照组进行单纯健康教育（每3个月1次）。健康教育由运动营养专家实施，有组织的运动训练由专职体育老师指导和管理。

1.3　运动方案

采用运动量阶段式递减的干预方式，以中等强度有氧运动为主，辅以力量和素质训练。有氧运动包括慢跑、篮球、跆拳道、爬山、游泳、骑自行车等。力量和素质训练包括仰卧起坐、俯卧撑、立定跳远、实心球、立位体前屈等。采用心率监测运动强度。运动方案为：前3个月（共12周）为第一阶段，即大运动量阶段，每周运动5天，运动强度为3～7MET，其中有氧运动每天60～90分钟，每周5天；力量和素质训练每天60分钟，每周2天。第4～9个月（共24周）为第二阶段，即小运动量阶段，每周4天，运动强度为3～7MET，其中有氧运动每天30～40分钟，每周4天，力量和素质训练每天20分钟，每周2天。

1.4　测试指标及方法

运动组分别于干预0、3、9个月时进行体格检查和血液生化指标测定；对照组各项指标分别在运动前后各测1次。身体测量和血液样本采集均在清晨空腹状态下进行。

1.4.1　身体测量

身高：采用金属立柱式身高坐高仪测定，受试者赤脚，立正姿势，躯干自然挺直，头部正直，足跟、骶骨部及两肩胛间三点与立柱相接触，由同一人员进行测量，测量人员两眼与水平板呈水平位进行读数，测量数据精确到0.1cm。

体重和体脂百分含量：采用TBF-300专业体成分测定仪（Tanita公司）测定。测量时受试者空腹、脱鞋、只穿轻薄的衣物，体重精确到0.1kg。

体重指数（BMI）＝体重/身高^2（kg/m^2）。

腰围：按照世界卫生组织（WHO）推荐的方法测量：被测者站立，双脚分开25～30cm，使体重均匀分布。沿髂前上嵴和第12肋下缘中点的水平连线测量腰围，皮尺紧贴软组织，但不压迫。

1.4.2　血液生化检查

血糖和血脂（TG、TC、HDL-C）：常规酶法。试剂盒购自北京中生北控生物科技股份有限公司。低密度脂蛋白胆固醇（LDL-C）采用Friedewald公式[5]计算：LDL-C（mmol/L）＝TC－HDL－TG/2.2。

空腹血清胰岛素检测采用放射免疫法[6]，试剂盒购自北京原子能研究所。

胰岛素抵抗指数（Homeostasis Model Assessment Insulin Resistance，HOMA-IR）[7]计算公式为HOMA-IR＝空腹血糖（mmol/L）×空腹胰岛素（μU/ml）/22.5。

胰岛素敏感指数（Insulin Sensitivity Index，ISI）[8]计算公式为ISI＝－ln（空腹血糖×空腹胰岛素）。

1.4.3　高甘油三酯（TG）血症和空腹血糖受损判断标准

?1994-2015 China Academic Journal Electronic Publishing House. All rights reserved.　http://www.cnki.net

采用美国 Sara 等针对儿童青少年提出的血清 TC 升高和空腹血糖受损的标准[9]：TG≥1.1mmol/L(100mg/dl)，血糖≥6.1mmol/L(110mg/dl)。

1.5 统计学处理

统计学处理采用 SPSS14.0 统计软件包进行。符合正态分布的，以均数±标准差进行统计描述；不符合正态分布的，以中位数(最小值~最大值)进行统计描述。符合参数检验条件的，组内采用配对 t 检验进行比较，组间采用独立样本 t 检验；不符合参数检验条件的，组内采用配对秩和检验，组间采用独立样本秩和检验。计数资料采用卡方检验。所有统计检验均采用双侧检验，P 值小于 0.05 被认为所检验指标的差异有统计意义。

2 结果

2.1 身体测量结果

在整个干预期间，两组受试者男、女生身高均连续增长(男生 $P<0.01$，女生 $P<0.05$。图 1A)；两组受试者男生体重连续显著增长($P<0.01$)，女生体重无显著性变化(图 1B)；运动组男生和女生 BMI 在干预期末与干预前相比分别下降 0.3 和 0.5，对照组男生和女生 BMI 分别上升 0.2 和 0.3，但变化无显著性(图 1C)。运动组女生体脂百分比在 9 个月末显著下降($P<0.01$)，对照组男、女生体脂百分比均无显著性变化(图 1E)；运动组男、女生在整个干预期间腰围均无显著性变化，而对照组男、女生腰围均显著上升($P<0.01$)(图 1D)。

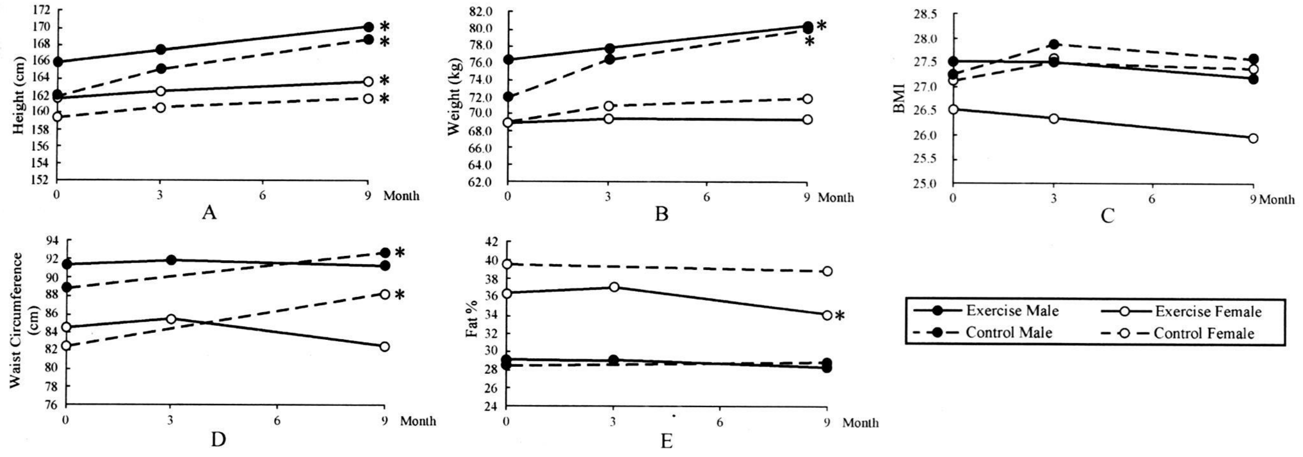

图 1 实验期间两组肥胖青少年身高、体重、BMI、腰围和体脂的变化(运动组 $n=25$，对照组 $n=24$)

Figure 1 The changing tendency of height, weight, BMI, waist circumference and body fat percentage of the obese adolescents during the exercise intervention (exercise group $n=25$, control group $n=24$).

A：身高；B：体重；C：BMI；D：腰围；E：体脂百分含量。*$P<0.05$，与运动前比较。

A: Height, B: Weight, C: Body mass index (BMI), D: Waist circumference, E: Body fat percentage (%). *$P<0.05$, compared with the pre-exercise intervention.

2.2 血脂、血糖、胰岛素、HOMA-IR 和 ISI 结果

表 2 实验期间两组肥胖青少年血脂、血糖、血清胰岛素、HOMA-IR 和 ISI 的变化

Table 2 The levels of fasting serum lipids level, glucose level, insulin level, HOMA-IR and ISI in obese adolescents

测试指标 parameters	组别 groups	n	运动干预前 pre-intervention	3 个月末 3rd month	9 个月末 9th month
TG(mmol/L)	运动组 exercise	24	1.31±0.67^{a}	1.02±0.60^{b}	0.87±0.29^{b}
	对照组 control	13	1.09±0.53^{a}		1.30±0.50^{b}
TC(mmol/L)	运动组 exercise	24	4.05±0.64	4.19±0.62	3.89±0.53
	对照组 control	13	4.48±0.81		4.83±0.80
HDL-C(mmol/L)	运动组 exercise	24	1.31±0.17	1.29±0.16	1.31±0.18
	对照组 control	13	1.35±0.36^{a}		0.95±0.27^{b}**
LDL-C(mmol/L)	运动组 exercise	24	2.13±0.68^{a}	2.44±0.45^{b}	2.21±0.47^{a}
	对照组 control	13	2.65±0.69		3.29±0.87
Serum glucose(mmol/L)	运动组 exercise	24	5.22±1.04^{a}	5.01±0.28^{a}	3.97±0.31^{b}
	对照组 control	13	4.64±0.80^{a}		5.23±0.69^{b}
Insulin(mU/L)	运动组 exercise	24	29.2(8.9~59.1)^{a}	23.7(7.2~54.1)^{b}	18.5(7.2~56.4)^{c}
	对照组 control	13	37.6(24.1~92.5)		43.8(12.6~99.9)
HOMA-IR	运动组 exercise	24	6.81(1.82~15.61)^{a}	5.16(1 57~12.45)^{b}	3.48(1.21~9.60)^{c}
	对照组 control	13	7.50(4.19~21.37)		10.84(3.20~27.44)
ISI	运动组 exercise	24	−5.05±0.44^{a}	−4.73±0.50^{b}	−4.35±0.53^{c}
	对照组 control	13	−5.32±0.56		−5.59±0.62

注：同行不同字母表示组内比较，$P<0.05$；**表示组间比较，$P<0.01$。

Note: The data for each parameter without a common superscript letter indicate significantly different ($P<0.05$), comparing within the same group. **$P<0.01$, compared with exercise group.

?1994-2015 China Academic Journal Electronic Publishing House. All rights reserved. http://www.cnki.net

表2显示，与运动干预前比较，运动组血清TG水平在3个月末和9个月末均显著下降($P<0.05$)，TC和HDL-C无显著性变化，LDL-C在3个月末上升($P<0.01$)，9个月末下降至干预前水平。对照组TG显著上升($P<0.05$)，HDL-C显著下降($P<0.01$)，且低于运动组水平($P<0.01$)，TC无显著性变化，LDL-C有上升趋势($P=0.058$)。

运动组空腹血糖在3个月末无显著性变化，9个月末显著下降($P<0.01$)，低于运动干预前；血清胰岛素水平、HOMA-IR在实验的9个月内均持续下降($P<0.05$)，ISI持续上升($P<0.05$)。而对照组血糖显著升高($P<0.05$)，血清胰岛素、HOMA-IR和ISI无显著变化。

2.3　高甘油三酯血症和空腹血糖受损检出率

如图2所示，运动组高甘油三酯血症比例在3个月末由运动前的60.0%(15/25)下降到20.0%(5/25)($P<0.05$)，9个月末仍为20.0%(5/25)($P<0.05$)，下降了66.6%，而对照组由运动前的22.2%(4/18)上升到72.2%(13/18)($P<0.05$)，上升了2.25倍；运动组空腹血糖受损比例在3个月末由运动前的24.0%(6/25)下降到0(0/25)($P<0.05$)，9个月末仍为0(0/25)，下降了100%，而对照组由6.3%(1/16)上升到18.8%(3/16)($P<0.05$)，上升了1.98倍。

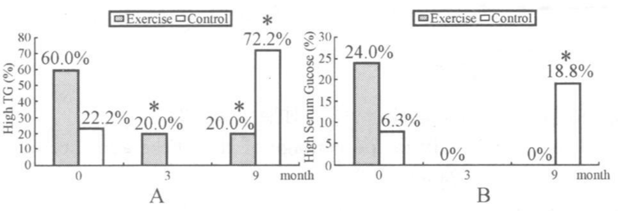

图2　实验期间两组肥胖青少年高甘油三酯血症(A)和空腹血糖受损(B)发生率的变化

Figure 2　Detection rates of hypertriglyceridemia and impaired fasting serum glucose

A：高甘油三酯血症(运动组 $n=25$，对照组 $n=18$)；B：空腹血糖受损(运动组 $n=25$，对照组 $n=16$)。

A：hypertriglyceridemia(exercise group $n=25$, control group $n=18$); B: impaired fasting serum glucose(exercise group $n=25$, control group $n=16$).

* $P<0.05$，与运动前比较，as compared with pre-exercise.

3　讨论

目前，对于肥胖儿童青少年是否需要进行干预减体重有两种不同的观点：一种观点是儿童青少年正处于生长发育期，随着身高的快速长高，肥胖会自动消失，因此不需要进行干预；另一种观点是为了肥胖儿童青少年的身心健康，防止肥胖儿童青少年发生代谢综合征和一些心理问题，以及成年后发生心血管疾病等慢性病，必须进行干预。本研究为运动干预不仅可以减体重，而且可以使肥胖青少年获得更多的健康效益提供了一定证据。

3.1　有组织的运动干预对肥胖的影响

儿童青少年正处于生长发育的重要阶段，其肥胖的控制应以不影响其正常的生长发育为前提。本研究所有受试者(包括运动组和对照组)在整个干预期间自由饮食，按照中国居民营养学会推荐的中国居民膳食营养素参考摄入量(Chinese DRIs)标准[10]，建议每日能量摄入为2200～2400kcal(9205～10042kJ)，适当调整膳食结构，减少脂肪摄入，多吃蔬菜和水果。膳食调查结果显示，运动组受试者每天平均能量摄入量为2063.3±576.5kcal，对照组为2005.9±592.5kcal，两组之间无显著差异，表明所有受试者在运动干预期间的膳食能量摄入量可以满足机体的需要，不会影响他们的正常生长发育。

在两组受试者膳食能量摄入量无显著差异且均正常的情况下，本研究结果显示，9个月的运动干预后，运动组身高与体重平均增长速度与对照组相比无显著差异，表明本研究的干预活动对青少年的正常生长发育速度无不良影响。对于生长发育期的儿童青少年，BMI水平随着年龄的增长而增长，因此，对肥胖儿童青少年的干预只要维持BMI水平不变，或者减少BMI增长的速度，便可以达到减肥目的。在本研究9个月、运动量两阶段递减的运动干预后，运动组男、女生BMI分别下降0.3和0.5，而对照组分别升高0.2和0.3，表明即使是持续小运动量运动也利于延缓肥胖的进程，预防肥胖的进一步发展。

研究证明，中心性肥胖是心脑血管病的独立危险因素[11]。青春期是体内脂肪重新聚集的一个时期，减肥时，减少体脂比减少体重更重要。目前认为腰围是反映中心性肥胖的较好指标。本研究结果显示，干预前后运动组肥胖男、女青少年的腰围无明显变化，女生体脂明显下降，而实验前后对照组男、女生腰围均显著上升，提示运动有助于防止肥胖青少年尤其是女生体脂在体内的过度聚集，抑制中心性肥胖的进一步发展，从而可能有利于降低心血管病发生的危险。

3.2　有组织的运动干预对糖、脂代谢的影响

本研究结果显示，运动组肥胖青少年血清TG、空腹血糖、胰岛素水平和HOMA-IR在经过3个月的较大运动量干预和随后6个月的小运动量干预后均显著降低，ISI持续升高，高甘油三酯血症和空腹血糖受损检出率明显下降，表明在较大运动量干预后坚持小运动量可以改善肥胖青少年糖脂代谢，提高胰岛素敏感性，减少高甘油三酯血症和空腹血糖受损发生率。其原因除了与运动本身动员脂肪消耗外，可能还与运动提高胰岛素敏感性，加强脂代谢有关[12]。胰岛素抵抗被认为是导致代谢综合征的危险因素，受内脏和肌肉脂肪积聚情况的调节，脂肪积聚的减少有助于改善胰岛素抵抗[13-15]。此外，运动可以加强胰岛素受体的自身磷酸化作用，增加葡萄糖转运蛋白4的表达，有利于葡萄糖运输，从而增加胰岛素敏感性[16-18]。

本研究结果显示，经过9个月的运动干预，运动组高密度脂蛋白胆固醇水平无明显变化，这与以前的研究结果一致[3,19,20]。但对照组肥胖青少年在干预期末高密度脂蛋白胆固醇显著下降，且低于运动组($P<0.05$)，表明长期运动可以有效预防肥胖青少年高密度脂蛋白胆固醇水平下降。

此外，本研究结果亦显示，有组织的运动量阶段式递减运动对肥胖青少年血清总胆固醇和低密度脂蛋白胆固醇无显著影响，可能是因为运动量较小，不足以使血清总

?1994-2015 China Academic Journal Electronic Publishing House. All rights reserved.　http://www.cnki.net

胆固醇和低密度脂蛋白胆固醇降低。研究表明,要降低血总胆固醇和低密度脂蛋白胆固醇水平可能需要更大的运动量(每天运动至少2.5~4小时)[3,19]和严格的饮食控制(平均每天能量摄入为1412kcal)[3],或者需要两者的共同作用。本研究考虑运动的可行性和持续性,希望以较少时间的运动达到有效的健康效益,故采用的运动量较小,即使在前3个月的较大运动量,每天的运动时间最多也只有2~2.5小时,后6个月运动时间只有0.8~1小时。采用的饮食方式是自由饮食,没有严格控制能量摄入(每天能量摄入为2063kcal)。

较正常孩子而言,肥胖孩子参加身体活动较少,并且在学校体育课中成绩较差。本研究在运动前后对受试者体能的测试结果显示,运动组运动后扔实心球的距离和立定跳远的距离明显增加,800米(女)/1000米(男)跑的速度显著提高,一分钟仰卧起坐的次数也显著增多,运动能力分别提高了17.9%、12.3%、22.3%、20.4%,反映速度的50米跑成绩也有上升的趋势。表明运动干预后,肥胖青少年上、下肢力量、耐力、柔韧性都有显著提高,速度素质也有改善的趋势,这不仅仅与减肥有关,可能更与运动本身提高体能的作用有关。

3.3 学校是对肥胖青少年进行运动干预的有效场所

在实行义务教育或将大多数儿童青少年纳入到教育体系的国家中,学校是开展运动干预的重要场所,这也是WHO提出"健康促进学校"计划的目标。以学校为基础的运动干预除了具有可行性和有效性外,还有良好的持续性和覆盖率,因为学生在校时间很长,能够通过不同的有组织的活动进行运动干预,如通过每天的课间操、体育课和课外活动等形式使运动持续下去,并覆盖大部分甚至所有在校学生。本研究结果也说明基于学校的有组织的开放式的运动干预对预防和防治肥胖是有效的、可行的。但由于某些家长和学生本人对肥胖的危害和运动干预认识不足,加之课业负担重,导致部分受试者中途退出。

4 总结

基于学校的有组织的运动量阶段式递减运动干预可以有效地预防和延缓肥胖青少年肥胖的进一步发展,改善糖脂代谢,提高胰岛素敏感性,降低高甘油三酯血症和空腹血糖受损发生率,预防高密度脂蛋白胆固醇的下降,增强体能素质。

5 参考文献

[1] Sung RY, Tong PC, Yu CW, et al. High prevalence of insulin resistance and metabolic syndrome in overweight/obese preadolescent Hong Kong Chinese children aged 9-12 years. Diabetes Care, 2003, 26(1): 250-251.

[2] Khaodhiar L, McCowen KC, Blackburn GL. Obesity and its comorbid conditions. Clin Cornerstone, 1999, 2(3): 17-31.

[3] Chen AK, Roberts CK, Barnard RJ. Effect of a short-term diet and exercise intervention on metabolic syndrome in overweight children. Metab Clin Exp, 2006, 55(7): 871-878.

[4] 中国肥胖问题工作组. 中国学龄儿童青少年超重、肥胖筛查体重指数值分类标准. 中华流行病学杂志, 2004, 25(2): 97-102.

[5] Friedewald WT, Levy RI, Fredrickson DS. Estimation of the concentration of low-density lipoprotein cholesterol in plasma, without use of the preparative ultracentrifuge. Clin Chem, 1972, 18(6): 499-502.

[6] Herbert V, Lau KS, Gottlieb CW, et al. Coated charcoal immunoassay of insulin. J Clin Endocr Metab, 1965, 25(10): 1375-1384.

[7] Haffner SM, Gonzalez C, Miettinen H, et al. A prospective analysis of the HOMA model. The Mecico City diabetes study. Diabetes Care, 1996, 19(10): 1138-1141.

[8] 李光伟, 潘孝仁. 检测人群胰岛素敏感性的一项新指数. 中华内科杂志, 1993, 32(10): 656.

[9] Sarah D, Kimberlee G, David S, et al. Prevalence of the metabolic syndrome in American adolescents. findings from the third national health and nutrition examination survey. Circulation, 2004, 110(16): 2494-2497.

[10] 中国营养学会. 中国居民膳食营养素参考摄入量(简要本). 第1版. 北京: 中国轻工业出版社, 2001.13.

[11] 周北凡, 武阳丰, 赵连成, 等. 我国中年人群向心性肥胖和心血管病危险因素及其聚集性. 中华心血管病杂志, 2001, 29(5): 70-73.

[12] Houmard JA, Tanner CJ, Slentz CA, et al. Effect of the volume and intensity of exercise training on insulin sensitivity. J Appl Physiol, 2004, 96(1): 101-106.

[13] Weiss R, Dufour S, Taksali SE, et al. Prediabetes in obese youth: a syndrome of impaired glucose tolerance, severe insulin resistance, andaltered myocellular and abdominal fat partitioning. Lancet, 2003, 362(9388): 951-957.

[14] Krssak M, Petersen KF, Dresner A, et al. Intramyocellular lipid concentrations are correlated with insulin sensitivity in humans: a 1HNMR spectroscopy study. Diabetologia, 1999, 42(10): 113-6.

[15] Montague C, O'Rahilly S. The perils of portliness: causes and consequences of visceral adiposity. Diabetes, 2000, 49(6): 883-8.

[16] Oungren JF, Keen S, Kulp JL, et al. Enhanced muscle insulin receptor autophosphorylation with short-term aerobic exercise training. Am J Physiol Endocrinol Metab, 2001, 280(3): E528-33.

[17] Vukovich MD, Arciero PJ, Kohrt WM, et al. Changes in insulin action and GLUT-4 with 6 days of inactivity in endurance runners. J Appl Physiol, 1996, 80(1): 240-4.

[18] Perseghin G, Price TB, Petersen KF, et al. Increased glucose transport-phosphorylation and muscle glycogen synthesis after exercise training in insulin-resistant subjects. N Engl J Med, 1996, 335(18): 1357-62.

[19] 李筱雯, 艾华, 张宝慧, 等. 有氧锻炼结合饮食控制减体重对肥胖者血抵抗素及心血管疾病危险因素的影响. 中国运动医学杂志, 2006, 25(4): 399-403.

[20] Esposito K, Pontillo A, Di-Palo C, et al. Effect of weight loss and lifestyle changes on vascular inflammatory markers in obese women: a randomized trial. JAMA, 2003, 289(14): 779-804.

(2007.06.22收稿; 2008.02.03修回)

?1994-2015 China Academic Journal Electronic Publishing House. All rights reserved. http://www.cnki.net
