# Supplementary material for: Systematic review and meta-analysis of school-based obesity interventions in mainland China
Source: PLoS One. 2017 Sep 14;12(9):e0184704. doi: 10.1371/journal.pone.0184704 (PMC5598996; doi:10.1371/journal.pone.0184704)
Supplement: S1 Dataset — (ZIP) [file pone.0184704.s007.zip › S1_dataset/76库/72.pdf]

·基础研究·

运动干预青少年单纯性肥胖效果的研究 *

荣湘江[1] 朱稼霈[2] 张世伟[3] 马 军[4]

摘要 目的:根据儿童青少年的生长发育特点和运动爱好特点,从运动干预角度以运动处方的形式对超重肥胖儿童青少年进行干预,以尝试找出更为合理且细化的运动处方,为学校、家庭及临床治疗提供客观的、可操作性的运动实施方案。方法:本次研究以北京十一学校初一年级 12、13 岁超重肥胖学生共 108 人为研究对象,其中实验组 54 人、对照组 54 人。测量并比较实验组受试者实验前后身体质量指数、肥胖度等反映肥胖状况的相关指标及与肥胖相关的生化指标的变化情况。结果与结论:3 个月的运动干预使受试对象体重减轻、BMI 值降低、身体脂肪含量减少,与国内外相近领域的其他研究报道相比是呈现相同性的,可以认定这些指标的变化与运动干预是有密切关系的。

关键词 肥胖;运动干预;运动处方;有氧运动

中图分类号:R493 **文献标识码**:A **文章编号**: 1001-1242(2007)-08-0702-04

A study on effects of exercise for simple obesity in young students/RONG Xiangjiang, ZHU Jiapei, ZHANG Shiwei, et al.//Chinese Journal of Rehabilitation Medicine, 2007, 22(8):702—705

Abstract Objective: To lose weight in overweight youth by behavior control according to their characteristics, namely growth, development and sports hobby. Such a more reasonable and particular prescription can provide, an objective and feasible measure for school, family and clinic. Method: The object of the research was 108 students with overweight in the age 12—13 among them 54 from experiment class and 54 from comparing class. The index of the body, fatness as well as the relevant condition were measured and compared. Result & Conclusion: Through 3-month-term measures the students in the experimental lost group weight, reduced BMI and fattiness, representing similarity with other researches home and abroad. The change of these indexes are closely relevant with the behavior intervention.

Author's address The Studies' Section of Health Care and Rehabilitation of Sports of Capital Institute of P.E. Beijing, 100088

Key words fattiness; behavior control; exercise prescription; aerobics

肥胖不仅是一个独立的慢性病, 也是多个慢性疾病的重要危险因素。我国有关调查资料表明:中小学生肥胖率上升速度很快,大城市尤为突出,其他城市也呈类似的上升趋势[1]。儿童期是生活方式和饮食习惯建立的关键阶段,此期实施正确的心理、行为改善、平衡饮食、运动处方、临床治疗和追踪监测的综合性干预是治疗肥胖最有效的途径和方法, 也是最具成本效益的干预方案。本文为提高学生身体素质,促进肥胖儿童的全面健康发展, 建立并实施儿童成年期疾病早期预防综合防治方案提供科学依据和技术支撑。

1 资料与方法

方便抽样

1.1 研究对象

选取北京市海淀区十一学校作为试点学校,根据海淀区 2006 年中小学体质健康监测数据结果并结合《中国儿童青少年超重、肥胖筛查 BMI 分类标准》对该校初中一年级 12、13 岁超重肥胖青少年进行筛选(12 岁男生 BMI 值在 21 以上,女生 BMI 值在 21.9 以上;13 岁男生 BMI 值在 21.9 以上, 女生 BMI 值在 22.6 以上), 对所筛选出的超重肥胖青少年进行正规体检,剔除因内分泌疾病、药物副作用等引起的肥胖儿童,最后确定 108 人作为干预对象,其中 54 人作为实验组,54 人作为对照组。实验对象均为单纯性超重肥胖学生。

1.2 研究内容

1.2.1 基本情况调查:主要内容包括父母、祖父母、外祖父母及其他亲属有无高血压、糖尿病、高脂血症、冠心病、肥胖、肿瘤等疾病,第二性征是否出现等方面。

1.2.2 测试指标:测量身高、体重、胸围、血压、血糖、

* 基金项目:国家体育总局 2006 年群体科研资助项目

1 首都体育学院保健康复教研室, 北京市北三环西路 11 号, 100088
2 首都铁路卫校
3 北京大学医学部研究生院
4 北京大学儿少卫生研究所
作者简介: 荣湘江,男,副教授

收稿日期:2007-05-10

© 1994-2010 China Academic Journal Electronic Publishing House. All rights reserved. http://www.cnki.net

甘油三酯、胆固醇、高密度脂蛋白、低密度脂蛋白等指标。测量肥胖率、身体脂肪量、脂肪百分比和去脂肪体重。

在 3 个月的干预实验结束后, 使用同样的仪器、方法和标准, 由相同的测试人员对受试对象进行以上各项的测试。

1.3 研究方案

1.3.1 运动干预: 干预期为 3 个月。采用运动处方的形式进行, 定期(每月)更换运动处方。运动强度: 以最大心率 (儿童青少年平均最大心率以 200 次/min 为准) 的 70%—80%为适宜。 运动时间: 每天下午 4 点左右, 40—50min; 运动频率: 每周 7 次, 每周一、三、五由学校体育教师监督指导, 每周二、四、六、日由家长监督指导。

运动项目: 根据男女学生的生理特点和运动兴趣爱好的不同选择相应的运动处方, 以慢跑、爬楼梯、跳绳、登山、划船等有氧运动为主。

1.3.2 饮食和行为控制: 由于所有受试对象有住校和走读的区别, 且绝大部分为走读生, 故无法进行集中的饮食和行为控制。要求实验组和对照组所有学生保持原有的饮食和行为习惯。

1.4 统计学分析

采用 SPSS11.0 统计软件对数据结果进行统计分析。两组实验前后肥胖程度构成的比较使用 χ^2 检验。对于数值型变量, 数据结果均以均数±标准差方式表示, 采用配对样本 t 检验比较自身前后变化。

2 结果

2.1 干预前后超重肥胖学生肥胖程度分布情况

见表 1—2。实验组和对照组干预前后肥胖程度构成比可以看出, 实验组 5 人体重恢复正常, 超重人数所占比例上升; 而对照组无体重恢复正常者, 超重和肥胖人数各自所占比例差异无显著性意义。

2.2 干预前后形态学、体成分测试、生理、生化指标的变化

见表 3。实验组和对照组实验前后身高均有非常显著性差异 (P<0.01), 实验组体重减少了 1.21kg, 存在显著性差异 (P<0.05); 实验组受试对象干预实验前后胸围值变动不大 (P>0.05), 差异无显著性意义; 对照组学生胸围实验前后变化有非常显著性意义 (P<0.01)。

实验组 BMI、肥胖率、身体脂肪量和脂肪百分比各值均较实验前降低, 呈现非常显著性差异 (P<0.01), 去脂肪体重一项无差异 (P>0.05)。而对照组中身体脂肪量和脂肪百分比的值实验后较实验前增加, 有非常显著性差异 (P<0.01); 其他几项指标差异无显著性意义 (P>0.05)。

超重肥胖学生干预实验前后生理、生化指标的变化见表 4。干预实验后实验组受试对象的收缩压和舒张压均较实验前降低, 收缩压一项有显著性差异 (P<0.05); 舒张压一项无显著性差异 (P>0.05)。血液中的血糖、甘油三酯、胆固醇和低密度脂蛋白的含量均比以前降低, 高密度脂蛋白含量比实验前升高, 差异有非常显著性意义 (P<0.01)。对照组这 7 项指标实验前后差异均无显著性意义 (P>0.05)。

表 1 实验组超重肥胖学生干预前后肥胖程度构成比

营养评价	BMI 参考值				干预前		干预后	
	男		女		人数	构成比(%)	人数	构成比(%)
	12 岁	13 岁	12 岁	13 岁				
正常	<21.0	<21.9	<21.9	<22.6	0	0.00	5	9.26
超重	21.0-24.7	21.9-25.7	21.9-24.5	22.6-25.6	22	40.74	28	51.85
肥胖	>24.7	>25.7	>24.5	>25.6	32	59.26	21	38.89

表 2 对照组超重肥胖学生干预前后肥胖程度构成比

营养评价	干预前		干预后	
	人数	构成比(%)	人数	构成比(%)
正常	0	0.00	0	0.00
超重	31	57.41	31	57.41
肥胖	23	42.59	23	42.59
合计	54	100.00	54	100.00

3 讨论

3.1 实验组运动干预的显形效果

3.1.1 运动干预对实验组超重、肥胖学生形态学指标的影响: 有研究表明, 运动时, 机体内血浆胰岛素水平下降, 胰高血糖素、促肾上腺皮质激素、促甲状腺素的浓度上升。这些激素的升高都可能激活脂肪细胞膜上的腺苷酸环化酶, 使细胞内环一磷酸腺苷水平提高, 由此又导致激素敏感性脂肪酶激活, 从而促进脂肪组织中脂肪水解成甘油和脂肪酸而进入血流。这样, 加速了体内脂肪组织中脂肪动员, 减少了体内贮脂[5]。另有实验证明, 脂肪氧化供能中游离脂肪酸是提供能量的主要物质。运动时肌肉对血液内游离脂肪酸和葡萄糖的摄取和利用增加, 它一方面使脂肪细胞释放大量游离脂肪酸, 使脂肪细胞逐渐缩小变瘦; 另一方面使多余的血糖被消耗而不能转化为脂肪。结果使体内脂肪量减少, 体重下降[6]。

© 1994-2010 China Academic Journal Electronic Publishing House. All rights reserved. http://www.cnki.net

表3 受试对象实验前后形态学及体成分测试指标的变化 ($\bar{x}\pm s$)

指标	实验组(n=54)		对照组(n=54)	
	实验前	实验后	实验前	实验后
身高(cm)	159.82±0.94④	160.99±0.93①④	159.63±1.00	160.81±1.00①
体重(kg)	66.13±1.51④	64.92±1.57②④	64.31±1.37	65.36±1.37①
胸围(cm)	85.70±0.95④	85.50±1.03④③	85.65±0.92	89.49±0.86①
BMI	25.78±0.43④	24.95±0.47①④	25.15±0.38	25.21±0.39③
肥胖率(%)	20.07±2.09④	16.12±2.20①④	16.56±1.86	16.79±1.87③
身体脂肪量(kg)	19.09±0.80④	17.33±0.78①④	18.79±0.70	19.74±0.72①
脂肪百分比(%)	28.41±0.66④	26.22±0.69①④	28.94±0.60	29.92±0.60①
去脂肪体重(kg)	47.04±0.87④	47.64±0.97④③	45.51±0.84	45.63±0.83③

组内实验前后比较: ①P<0.01有非常显著性差异, ②P<0.05, ③P>0.05; 组间实验前后比较: ④P>0.05

表4 受试对象干预实验前后生理、生化指标的变化($\bar{x}\pm s$)

指标	实验组(n=54)		对照组(n=54)	
	实验前	实验后	实验前	实验后
收缩压(mmHg)	113.41±1.86③	110.19±1.53②③	112.00±1.62	111.22±1.52⑤
舒张压(mmHg)	69.30±1.37③	68.33±1.24③⑤	65.37±0.87	66.33±1.28⑤
血糖(mmol/L)	5.44±0.62③	5.28±0.56①③	5.46±0.56	5.46±0.57⑤
甘油三酯(mmol/L)	1.05±0.04③	0.87±0.43①③	1.07±0.58	1.08±0.58⑤
胆固醇(mmol/L)	4.00±0.86③	3.73±0.78①③	4.00±0.11	4.02±0.11⑤
高密度脂蛋白(mmol/L)	1.42±0.37③	1.56±0.36①③	1.43±0.36	1.44±0.36⑤
低密度脂蛋白(mmol/L)	2.30±0.76③	1.97±0.57①④	2.22±0.92	2.22±0.91⑤

组内实验前后比较: ①P<0.01, ②P<0.05; ⑤P>0.05; 组间实验前后比较: ③P>0.05; ④P<0.05

Scottowns[7]对81例7—11岁的肥胖儿童进行运动训练后发现体脂百分比显著降低; 而去脂体重增加,有氧能力也显著性增加。谭晖等[8]对76例7—11岁单纯性肥胖儿童进行为期2.5年的中等强度体育锻炼,结果显示,总皮褶厚度、体脂率、肥胖度、BMI等4项指标均有所下降。说明健身运动锻炼可有效减少学龄期儿童的体脂、改善体成分,有效控制肥胖儿童体重。

表3示, 运动干预实验后受试对象的身高、体重、BMI指数、肥胖率、身体脂肪量和脂肪百分比都较实验后产生了一定的变化, 这几项指标的变化差异具有显著性。本研究结果实验组身体脂肪量减少,差异有显著性意义, 可以说在降低受试者身体脂肪含量方面运动干预是有效果的。

3.1.2 运动干预对实验组超重、肥胖学生生理、生化指标的影响: 空腹血糖、胆固醇、甘油三酯等都是与肥胖有关的危险因子[9]。有研究表明, 正常情况下, 血中胆固醇和甘油三酯的浓度受进食、运动、疾病等多种因素的影响, 短时期外源性的高胆固醇和高饱和脂肪酸的摄入对血胆固醇和甘油三酯并没有明显的影响, 但长期的饮食结构和成分是影响血胆固醇和甘油三酯水平的重要因素[10—11]。血脂中, 尤其是胆固醇的质和量是影响身体健康的直接因素[12]。体育运动, 尤其是有氧运动可改善脂质代谢,运动时肾上腺素、去甲肾上腺素分泌的增加,可提高脂蛋白酯酶的活性, 加速富含甘油三酯的乳糜和极低密度脂蛋白的分解。因此,可以降低血脂而使高密度脂蛋白胆固醇量升高[13]。

王小引等[14]实验结果的生化指标当中, 血糖、血脂、总胆固醇、低密度脂蛋白等与干预前测试结果比较明显降低, 而对身体有益的高密度脂蛋白比干预前明显升高。李蓓[15]在其研究结果中所显示的血压、胆固醇、甘油三酯等指标变化与以上两个研究成果亦相近。本研究通过对单纯性超重肥胖儿童青少年3个月的运动干预实验, 受试对象的收缩压、舒张压、血糖、甘油三酯、胆固醇、高密度脂蛋白和低密度脂蛋白均较干预前有所变化。其中高密度脂蛋白值比干预实验前增高, 其他指标均较干预实验前降低。这些生化指标均向有益于身体方向发展。可以认为运动干预对超重肥胖儿童青少年身体各项与肥胖有关的指标的改变有一定的效果。

3.2 实验组运动干预的非显形效果

3.2.1 实验组运动干预对超重肥胖学生胸围的影响及与对照组的对比: 对于运动干预对胸围的影响, 各实验结果不尽相同。李明[16]实验结果中实验组学生的胸围由实验前的88.54cm经过10个月的干预降低到83.03cm, 有非常显著性差异。傅兰英[17]的实验结果中胸围也是有非常显著性差异的。而左燕等[18]的研究结果显示胸围没有显著性差异。本实验在进行运动干预后, 实验组学生胸围平均值仅降低了0.2cm, 无显著性差异。分析可能的原因如下: ①12、13岁的儿童青少年正处于发育阶段, 他们自身各形态学指标都要发育, 胸廓也不例外。②由于所取的胸围指标是男女生的均值, 再综合第一方面的原因, 可以看出3个月的运动干预对实验组学生胸围的减少是产生了一定的作用的。③干预时间较短, 还不足以使胸围下降很明显。

3.2.2 运动干预对超重肥胖学生去脂肪体重的影响: 研究表明,长期的运动干预可以有效地降低脂肪含量、使去脂肪体重增加。本实验研究结果当中, 实验组去脂肪体重由47kg增加到47.6kg, 增加幅度很小。分析原因可能是由于干预时间较短, 儿童青少年学生去脂肪体重成分不至于增长很快, 各种运动方式还不足以对学生的瘦体重的增长形成明显的促进作用。

© 1994-2010 China Academic Journal Electronic Publishing House. All rights reserved. http://www.cnki.net

3.3 实验组与对照组在运动干预前后两组间的效果对比

表 3 示形态学及体成分指标实验组和对照组对比结果中，无论实验前还是实验后两组比较 P 值均>0.05。在表 4 中，生理、生化指标干预实验前后实验组和对照组对比结果，在实验前只有舒张压差异有显著性意义(P<0.05)，其他各项均差异无显著性意义；实验后对比结果中除低密度脂蛋白一项有差异以外，其他各项差异均无显著性。以上结果，实验前比较无差异说明所选受试对象的各指标在同一水平上。对于身高一项，在实验前后两组差异均无显著性意义，说明本实验运用的运动干预措施未对学生的生长发育造成影响，这是我们所希望的。对于其他形态学和体成分指标两组间干预前后比差异无显著性意义的，分析原因可能是由于 3 个月的干预时间较短，各项指标的变化程度相对较小，还不足以显示出明显的差异性。

3.4 用运动处方形式进行运动干预的益处

运动处方是在身体测评的基础上，根据锻炼者身体的需要，按照科学健身的原则，为锻炼者提供的量化指导方案。它以生理学为理论依据，以身体练习为基本手段，以增强体质、促进身体全面发展、提高生存质量为根本目的。具有科学性、灵活性、针对性强，便于自我控制和自我评价等特点。运动处方的最大益处在于安全性好、针对性强。在本次研究中根据受试对象的性别和生理特点来制订不同的运动处方，并且定期根据效果更换，事实证明这种运动干预的形式容易被受试者接受，并且效果也较好。

4 结论

在接受 3 个月的干预实验后，实验组受试对象的体重、BMI 指数、肥胖率、身体脂肪量、脂肪百分比均出现了有意义的变化，而对照组则显示不出与实验组相同的变化和差异性。另外实验组受试对象生理、生化指标中除舒张压差异无显著性外，其他各项均较实验前有显著性变化，而对照组无一项有差异性变化。可以认为，超重、肥胖青少年的生理生化指标的改变是与运动干预有密切关系的。胸围一项均值实验后较实验前有所下降，虽然其均值变化不是很明显，但从青少年身体正常发育来看，若不是对其实施运动干预胸围应为上升值。本结果提示，运动干预对胸围的影响也是有一定效果的。而去脂肪体重一项实验后较实验前有所上升，但差异无显著性意义，这与青少年生长发育规律是一致的。

参考文献

[1] 谭琪，徐勇.中国儿童青少年超重发展趋势分析[J].中国学校卫生，2003，12：609—610.

[2] 孟昭恒.对儿童青少年肥胖判定方法的评价[J].中华预防医学杂志，1998.32(3)：185—186.

[3] 中国肥胖问题工作组.中国学龄儿童青少年超重、肥胖筛查体重指数值分类标准[J].中华流行病学杂志，2004，25(2)：97—102.

[4] 刘纪清，李国兰.实用运动处方[M].哈尔滨：黑龙江科技出版社，1993.

[5] 李香华.单纯性肥胖的主要原因与运动减肥的机理[J].岳阳师范学院学报，2001，14(3)：83—86.

[6] 李红，周小平.试论有氧运动对防止肥胖症的作用[J].湛江师范学院学报，1999，20(1)：96—98.

[7] Scottowns, Bernard Gutin, Jerry Allison, et al. Effect of physical training on total and viseral fat in obese children[J]. Med Sci Sports Exer,1999,31:143—148.

[8] 谭晖，王震维，安爱华.单纯性肥胖儿童体育锻炼的效果评价[J].实用预防医学，2002，9(1)：78.

[9] De Fronzo RA, Ferranini E, Insulin resistance.A Multifaceted syndrome responsible for NIDDM,obesity,hypertension, dyslipidemia, and atheroscl- erosis cardiovascular disease [J]. Diabetes Care,1991,14(3):173—194.

[10] 戴俊，姜振，张兵.运动与营养疗法干预单纯性肥胖儿童的效果[J].中国临床康复，2006，10(25)：20—22.

[11] 于素梅.肥胖有氧运动减肥的生物学分析[J].北京体育大学学报，2001，24(1)：63—64.

[12] 华建军，张国强，苏国英，等.运动减肥的机制及运动处方[J].内蒙古电大学刊，2005，5：107—108.

[13] 王小引，傅兰英，姬成茂.有氧运动及综合减肥对超重和肥胖女学生影响[J].中国公共卫生，2006，22(8)：904—905.

[14] 李蓓. 儿童单纯性肥胖症的综合干预治疗 [J]. 中国临床康复，2002，6(13)：1952—1953.

[15] 李明.超重、肥胖女学生有效减肥的运动及综合饮食处方[J].中国临床康复，2006，10(32)：44—46.

[16] 华人运动生理与体适能学者学会.运动生理学传入中国——中国第一本《运动生理》研究报告专论 [J]. 中国运动医学杂志，2006，25(5)：604—607.

[17] 左燕，张旭，宋加华. 肥胖学生减肥健身初探 [J]. 体育学刊，1999，4：57—59.

© 1994-2010 China Academic Journal Electronic Publishing House. All rights reserved.　http://www.cnki.net
