# Supplementary material for: Systematic review and meta-analysis of school-based obesity interventions in mainland China
Source: PLoS One. 2017 Sep 14;12(9):e0184704. doi: 10.1371/journal.pone.0184704 (PMC5598996; doi:10.1371/journal.pone.0184704)
Supplement: S1 Dataset — (ZIP) [file pone.0184704.s007.zip › S1_dataset/76库/73.pdf]

体医结合方案对单纯性肥胖儿童血清瘦素和血脂水平的影响

杨 晓 林
（新乡医学院 体育教学部，河南 新乡 453003）

Effect of Combination of Medicine and Sport on Serum Leptin and Lipid Composition of Obese Children

YANG Xiao - lin

摘要：目的 研究体医结合方案对单纯性肥胖儿童血清瘦素及血脂水平的影响，为建立行之有效的体医结合方案提供科学依据。**方法** 采用整群抽样法，筛选出44例单纯性肥胖儿童，随机分为干预组和对照组。干预组采用体医结合方案进行为期8个月的干预，对照组不予任何干预。检测干预前后身体形态、血清瘦素及血脂水平的变化。**结果** 干预后，干预组腰围、臀围、血清瘦素、TG、LDL-C和apoB100较干预前明显降低（$P_a < 0.05$），HDL-C、apoAI较干预前明显升高（$P < 0.05, 0.01$）；而对照组干预前后比较差异均无统计学意义（$P_a > 0.05$）。**结论** 体医结合方案能有效降低体质量，改善单纯性肥胖儿童瘦素和血脂水平，起到调节异常内分泌代谢的作用。

实用儿科临床杂志，2010，25（18）：1447－1448

关键词： 体医结合方案；单纯性肥胖；瘦素；儿童
中图分类号： R725.8 **文献标识码：** B **文章编号：** 1003－515X(2010)18－1447－02

随着人们物质生活水平的不断提高，单纯性肥胖（肥胖）儿童日渐增多，肥胖不仅给生活带来不便，而且还影响正常的生长发育，甚至可引起成年高血压、冠状动脉粥样硬化性心脏病（冠心病）、糖尿病等多种并发症[1-3]。本研究采用体医结合方案对肥胖儿童进行干预研究，观察干预前后血清瘦素及血脂水平的变化，为肥胖儿童及其并发症的预防和治疗提供依据。

1 资料与方法

1.1 一般资料 2008年8月在新乡市3所普通高校的附属中、小学整群抽取肥胖[4]儿童44例，男22例，女22例；年龄8～14（11.2±2.4）岁。随机分为干预组和对照组，每组各22例。2组儿童年龄、体质量及肥胖情况均无统计学差异。干预组按照体医结合方案执行，对照组自行安排。

1.2 体医结合方案的制定[5-6]

1.2.1 体育干预 根据干预对象的个体情况，选择跑步、踢球、踢毽子、跳皮筋、爬楼梯、跳绳、游泳等体育运动，以脉搏达到130～150次·min^{-1}为宜，每天下午课后，周末不间断，每周5～7次，每次时间为30～60 min，以孩子不感到过度疲劳为宜。干预时间8个月。

1.2.2 医务监督 组织患儿和患儿家长，采取观看录像、讲座和发放自编宣传手册等方式，宣传肥胖防治知识（包括任何控制体质量、BMI的计算与评价、食物的种类及合理搭配、科学锻炼、肥胖与慢性病关系等）；在减肥干预过程中，定期测量身体形态、血脂等指标。根据每个孩子的特点，制定出一日三餐的饮食食谱，并做膳食记录。一日三餐实施低热量平衡膳食，食物的选择要注意营养成分，选取低热量、具有饱腹感的食品，减少高油脂、高淀粉类食物，适当增加蔬菜、水果、豆制品及纤维素摄入；饮食疗法要有家长的参与。通过教育使肥胖儿童认识到肥胖的潜在危害，并自我矫治不良饮食习惯。制订自我监测内容，包括每天、每餐的进食量，定期测量体质量等，有成绩及时给予鼓励，以增强减肥的信心。最终让孩子自觉执行减肥方案，达到健康减肥的目的。

1.3 血清瘦素及血脂检测 清晨空腹取肘静脉血5 mL。血糖（GLU）、三酰甘油（TG）、胆固醇（TC）的测定应用全自动生化分析仪（日本Olympus Au 400）采用酶法检测；载脂蛋白B100（apoB100）、载脂蛋白AI（apoAI）测定采用免疫透射比浊法；瘦素测定采用放射免疫法，试剂盒由美国Headquarters公司提供。

1.4 统计学处理 采用SPSS 15.0软件，数据用$\bar{x} \pm s$表示，采用t检验，$P < 0.05$为差异有统计学意义。

2 结果

2.1 干预组及对照组干预前后身高、体质量、BMI、腰围及臀围的变化 结果见表1。

2.2 干预组及对照组干预前后GLU、血清瘦素和血脂成分的变化 结果见表2。

3 讨论

体医结合是指运动医学、保健体育、康复医学、医学营养、健康评估、运动处方等众多知识体系于一身的一门学科，知识面几乎涵盖了所有与运动有关的医学内容。儿童肥胖的治疗有别于成人肥胖，因为儿童具有生长发育的特点，加之儿童不像成人能较好地配合，给干预方案的实施带来困难，家长的合作就显得尤为重要。因此，本研究采用体医结合方案、邀请了儿童和儿童家长共同参与。

研究显示采用极低热量饮食仅维持6～10个月，加上心理矫正可持续1～2 a，加上体育活动可持续1～6 a[5-6]。

基金项目： 2009年全国教育科学"十一·五"规划重点课题（DIA090307）；2010年河南省政府决策招标课题（E336）；2007年河南省教育厅教科所课题（2007－JKGHAG－349）
作者简介： 杨晓林，男，副教授，学士学位，电子信箱 yangxiaolin@xxmu.edu.cn。

万方数据

实用儿科临床杂志第25卷第18期2010年9月 J Appl Clin Pediatr, Vol. 25 No. 18, Sep. 2010

表1 对照组及干预组肥胖儿童干预前后体检变化 ($\bar{x}\pm s$)

指标	对照组		t	P	干预组		t	P
	干预前	干预后			干预前	干预后		
身高/cm	146.58±6.22	146.90 ±5.40	0.84	>0.05	146.24±3.42	146.76±7.56	0.69	>0.05
体质量/kg	56.70±3.26	57.13±4.65	1.59	>0.05	57.24±3.46	51.10±3.40	2.24	<0.05
BMI	25.24±1.57	25.66±3.42	0.98	>0.05	25.54±1.26	21.35±3.52	2.09	<0.05
腰围/cm	80.30±4.56	80.68±2.55	0.87	>0.05	80.45±4.27	69.35±3.54	3.58	<0.01
臀围/cm	84.88±3.34	85.06±2.20	1.23	>0.05	85.04±3.48	80.30±1.36	2.12	<0.05

表2 对照组及干预组肥胖儿童干预前后血清 GLU、瘦素和血脂变化的比较 ($\bar{x}\pm s$)

指标	对照组		t	P	干预组		t	P
	干预前	干预后			干预前	干预后		
GLU/mmol·L^{-1}	4.22±0.23	4.24±0.26	0.87	>0.05	4.24±0.40	4.22±0.24	1.67	>0.05
TG/mmol·L^{-1}	1.20±0.22	1.21±0.35	1.20	>0.05	1.20±0.50	0.96±0.41	2.68	<0.01
TC/mmol·L^{-1}	4.22±1.07	4.23±0.86	1.32	>0.05	4.23±0.80	4.18±0.96	2.03	<0.05
HDL-C/mmol·L^{-1}	1.35±0.50	1.35±0.55	0.57	>0.05	1.35±0.62	1.56±0.36	2.25	<0.05
LDL-C/mmol·L^{-1}	2.95±0.26	2.95±0.47	0.67	>0.05	2.95±0.50	2.56±0.56	3.01	<0.01
瘦素/ng·L^{-1}	32.27±8.56	32.50±10.27	0.73	>0.05	33.58±12.52	19.58±7.67	4.65	<0.01
apoB100/g·L^{-1}	0.89±0.25	0.88±0.17	1.14	>0.05	0.89±0.20	0.79±0.13	2.24	<0.05
apoAI/g·L^{-1}	1.21±0.23	1.22±0.34	0.97	>0.05	1.20±0.30	1.32±0.27	2.42	<0.01

然而，与成人减肥不同，儿童肥胖的控制必须以不影响其正常的生长发育为前提，因此，本研究与每个肥胖儿童及其家长共同确定健康减肥的教育目标。而且组成了由运动医学专家、专业健身教练、临床医师、营养学人士的医务监督队伍，及时观察儿童身体对运动负荷和饮食的反应，定期测量他们的各项指标，并结合 GLU、血脂等项指标，及时调整运动量及饮食疗法。

流行病学研究表明，HDL-C 水平与冠心病发病危险性呈负相关，LDL-C 水平与和患冠心病危险性呈正相关[7]。肥胖儿童脂质代谢紊乱表现为 TG、TC、LDL-C 增高及 HDL-C 降低。研究表明，肥胖儿童血瘦素水平均显著高于正常水平，即存在瘦素抵抗[8,9]。在高瘦素存在条件下，致使体内糖类、脂类代谢异常，出现血 TG、GLU、TC、极低密度脂蛋白及游离脂肪酸增高，而高密度脂蛋白减少，极易发生动脉硬化、冠心病等。

本组采用体医结合方案进行 8 个月的干预，肥胖儿童的体质量、BMI、臀围、腰围均显著下降，同时瘦素、TG、LDL-C 和 apoB100 较干预前明显降低，HDL-C、apoAI 较干预前明显升高，说明该方案具有良好的减肥作用。

参考文献：

[1] 李田华，张春香. 单纯性肥胖对青春期前男童性发育的影响[J]. 实用儿科临床杂志，2009，24(20)：1574－1575.
[2] 全国儿童期单纯性肥胖症研究协作组. 全国 0－6 岁儿童期单纯性肥胖流行病学研究[J]. 中华儿科杂志，2008，46(3)：179－184.
[3] 凌文杰. 不同有氧运动处方对单纯性超重和肥胖青少年学生减肥效果观察[J]. 新乡医学院学报，2007，24(3)：232－234.
[4] 杨锡强，易著文. 儿科学[M]. 6 版. 北京：人民卫生出版社，2004：86.
[5] 傅兰英，刘小学，王小引，等. 超重与肥胖青少年减肥现状及干预效果研究[J]. 中国康复医学杂志，2007，22(1)：65－66.
[6] 陈华佳，胡 婷. 单纯性肥胖儿童胰岛素抵抗与游离脂肪酸的关系[J]. 新乡医学院学报，2008，25(3)：250－252.
[7] 杨万龄，罗盈怡，王晓明. 儿童青少年肥胖超重状况及影响因素分析[J]. 中国公共卫生，2009，25(8)：958.
[8] 倪世宁，刘倩琦，朱子阳，等. 儿童单纯性肥胖与胰岛素抵抗综合征危险因素的关系[J]. 实用儿科临床杂志，2009，24(7)：503－504.
[9] 王小引，盛 伟，傅兰英. 试验减肥对单纯性肥胖青少年女学生血瘦素和胆固醇的影响[J]. 新乡医学院学报，2005，22(2)：83－84.

(收稿日期：2010－07－20)
(本文编辑：周二强)

《中国中西医结合肾病杂志》2011 年征订启事

《中国中西医结合肾病杂志》是中国中西医结合学会主办、中国科学技术协会主管、国内外公开发行的国家级学术期刊。本刊执行“中西医并重、促进中西医结合”方针，旨在报道我国中西医结合在肾病临床、科研、预防等方面的经验，介绍国内外有关肾病专业的进展，为提高中西医结合水平，继承和发扬我国传统的医药学，为促进我国医学科学现代化服务。本刊已进入《中国科技论文统计源期刊》、《中国科技核心期刊》、《中国学术期刊综合评价数据库统计源期刊》、《中国科学技术引文数据库来源期刊》、《中国学术期刊(光盘版)》。本刊辟有著名专家论坛、名医经验荟萃、实验论著、临床论著、短篇论著、病例报告、讲座与综述、专病专方、体会与交流、学术动态等栏目。杂志现发行面已覆盖全国各省、市、自治区，全国各医疗卫生单位内科、肾内科、儿科、妇产科、中医科、泌尿科等医务人员及相关的科研、教学人员均可参阅。本刊为月刊，每期订价 12.00 元，全年 144.00 元，国际标准刊号：ISSN 1009－587X，国内统一刊号：CN 14－1277/R，邮发代号：22－26，全国各地邮局均可预定，也可直接向编辑部办理邮购。编辑部地址：山西省太原市 06079 信箱，邮政编码：030006；电话(传真)：0351－7965258，8500946；电子信箱：sx7965258@126.com。

万方数据

万方数据 WANFANG DATA 文献链接

体医结合方案对单纯性肥胖儿童血清瘦素和血脂水平的影响

作者： 杨晓林， YANG Xiao-lin
作者单位： 新乡医学院体育教学部,河南,新乡,453003
刊名： 实用儿科临床杂志 ISTIC PKU
英文刊名： JOURNAL OF APPLIED CLINICAL PEDIATRICS
年，卷(期)： 2010,25(18)

参考文献(9条)

1. 李田华;张存香 单纯性肥胖对青春期前男童性发育的影响[期刊论文]-实用儿科临床杂志 2009(20)

2. 全国儿童期单纯性肥胖症研究协作组 全国0-6岁儿童期单纯性肥胖流行病学研究[期刊论文]-中华儿科杂志 2008(03)

3. 凌文杰 不同有氧运动处方对单纯性超重和肥胖青少年学生减肥效果观察[期刊论文]-新乡医学院学报 2007(03)

4. 杨锡强;易著文 儿利学 2004

5. 傅兰英;刘小学;王小引 超重与肥胖青少年减肥现状及干预效果研究[期刊论文]-中国康复医学杂志 2007(01)

6. 陈华佳;胡婷 单纯性肥胖儿童胰岛素抵抗与游离脂肪酸的关系[期刊论文]-新乡医学院学报 2008(03)

7. 杨万龄;罗盈怡;王晓明 儿童i少年肥胖超重状况及影响因素分析[期刊论文]-中国公共卫生 2009(08)

8. 倪世宁;刘倩琦;朱子阳 儿童单纯性肥胖与胰岛素抵抗综合征危险因素的关系[期刊论文]-实用儿科临床杂志 2009(07)

9. 王小小;盛伟;傅兰英 试验减肥对单纯性肥胖青少年女学生血瘦素和胆固醇的影响[期刊论文]-新乡医学院学报 2005(02)

引用本文格式：杨晓林.YANG Xiao-lin 体医结合方案对单纯性肥胖儿童血清瘦素和血脂水平的影响[期刊论文]-实用儿科临床杂志 2010(18)
