# Supplementary material for: Systematic review and meta-analysis of school-based obesity interventions in mainland China
Source: PLoS One. 2017 Sep 14;12(9):e0184704. doi: 10.1371/journal.pone.0184704 (PMC5598996; doi:10.1371/journal.pone.0184704)
Supplement: S1 Dataset — (ZIP) [file pone.0184704.s007.zip › S1_dataset/76库/75.pdf]

·论著·

社会生态模式在儿童青少年肥胖防控中对体格指标的干预效果

崔馨月[1,2], 陈天娇[2△], 马 军[2]
(1.北京大学公共卫生学院,北京 100191;2.北京大学儿童青少年卫生研究所,北京 100191)

[摘 要] 目的:探索利用社会生态模型,从“学生-学校-家庭”三个层面对学生肥胖进行干预是否有效。方法:利用分层整群抽样方法选择北京市房山区城市小学、乡村小学、城市中学和乡村中学各4所,共16所学校7~18岁学生共得到有效样本3 175人,每层4所学校各选取2所学校共1 661人,另2所学校作对照组,利用社会生态模型从“学生-学校-家庭”三个层面进行为期3个月的肥胖干预。干预方式包括健康教育、环境改善等,干预内容涉及肥胖相关知识、健康饮食与运动等。检测干预前后身体形态指标。结果:干预可以整体改善超重肥胖(OR=1.12,P<0.05),降低腰围、腰臀比(waist-hip ratio,WHR)等中心性肥胖指标(adjusted difference=0.63、0.02,P<0.05);女生腰围和WHR降低(adjusted difference=0.52、0.02,P<0.05)并改善了超重肥胖(OR=1.18,P<0.05),男生腰围和WHR降低(adjusted difference=0.73、0.01,P<0.05),女生效果优于男生;城市地区WHR降低(adjusted difference=0.01,P<0.05),乡村地区腰围和WHR降低(adjusted difference=1.05、0.02,P<0.05)并改善了超重肥胖(OR=1.43,P<0.05),乡村效果优于城市;小学WHR降低(adjusted difference=0.02,P<0.05)并改善了超重肥胖(OR=1.21,P<0.05),中学WHR降低(adjusted difference=0.02,P<0.05),小学效果优于中学;干预措施有效降低超重肥胖学生的超重肥胖率(χ^2=11.01,P<0.01)。结论:“学生-学校-家庭”社会生态模式干预方法短期内可改善腰围、WHR等中心性肥胖指标,可以作为一种有效干预手段推广使用。

[关键词] 肥胖,腹型;早期干预(教育);学生;腰围;腰臀比
[中图分类号] R179 [文献标志码] A [文章编号] 1671-167X(2015)03-0400-06
doi:10.3969/j.issn.1671-167X.2015.03.007

Effect of obesity intervention with socio-ecological model on anthropometric measurements of children and adolescents

CUI Xin-yue[1,2], CHEN Tian-jiao[2△], MA Jun[2]
(1. Peking University School of Public Health, Beijing 100191, China; 2. Institute of Child and Adolescent Health, Peking University, Beijing 100191, China)

ABSTRACT **Objective:** To study whether the socio-ecological model based on “student-school-family” three-level strategy is effective in obesity prevention. **Methods:** A total of 3 175 students aged 7 to 18 from 16 schools (4 urban primary schools, 4 rural primary schools, 4 urban secondary schools and 4 rural secondary schools, of which 2 intervention schools were selected, respectively) were recruited by stratified cluster sampling method. A three-month intervention using “student-school-family” socio-ecological model was conducted through health education and environment improvement. The intervention contents included knowledge on obesity, healthy diet and physical activities. Their anthropometric indexes were recorded. **Results:** The intervention prevented obesity (OR = 1.12, P < 0.05), and was effective in waist circumference (WC) and waist-hip ratio (WHR) (adjusted difference = 0.63, 0.02, P < 0.05). WC and WHR were reduced in girls (adjusted difference = 0.52 & 0.02, P < 0.05), and obesity was prevented in girls (OR = 1.18, P < 0.05). WC and WHR were reduced in boys (adjusted difference = 0.73, 0.01, P < 0.05). WHR were reduced in urban areas (adjusted difference = 0.01, P < 0.05). WC and WHR were reduced (adjusted difference = 1.05, 0.02, P < 0.05) and obesity was prevented (OR = 1.18, P < 0.05) in rural areas. WHR were reduced (adjusted difference = 0.01, P < 0.05) and obesity was prevented (OR = 1.21, P < 0.05) in primary schools. WHR were reduced in secondary schools (adjusted difference = 0.02, P < 0.05). The intervention effect was better in girls than in boys, in rural areas than in urban areas, and in primary schools than in secondary schools. The overweight and obesity prevalence went down after the intervention (χ^2 = 11.01, P < 0.01). **Conclusion:** Intervention strategy is effective in central obesity indexes such as WC and WHR, and it can be used widely.

基金项目:卫生公益性行业科研专项(201202010)资助 Supported by the Specific Research Project of Health Pro Bono Secters, Ministry of Health, China (201202010)
△ Corresponding author's e-mail, tianjiaochen@163.com
网络出版时间:2015-5-15 13:14:16 网络出版地址:http://www.cnki.net/kcms/detail/11.4691.R.20150515.1314.003.html

?1994-2015 China Academic Journal Electronic Publishing House. All rights reserved. http://www.cnki.net

KEY WORDS　Obesity，abdominal; Early intervention (education); Students; Waist circumference; Waist-hip ratio

中国儿童青少年超重肥胖问题已经是一个公认的严重的健康问题，近年来，儿童的超重肥胖率迅速上涨，沿海大城市男生的超重肥胖率已达到32.6%[1]。我国学者用营养干预、运动干预、综合干预等干预方式进行肥胖防控，取得了一定的效果。1988年Mcleroy等[2]指出外部环境对人的健康促进干预影响可以通过社会生态模型的五个水平进行(个体水平、人际水平、机构水平、社区水平和公共政策)。国外很多学者利用此模型对肥胖进行干预，并建议在今后的肥胖干预中设计对应各个水平的干预措施[3-4]，此模型在中国多应用于艾滋病、运动相关健康教育与行为促进[5-6]，而在肥胖防控中的作用尚未见报道。为探索更加有效的学生肥胖防控方法，本研究利用社会生态模型理论的前三个水平，从"学生-学校-家庭"对学生肥胖进行干预，观察和评估干预效果。

1　资料与方法

1.1　研究对象

采用分层整群抽样的方式抽取调查对象，将北京市房山区学校分为城市小学、乡村小学、城市中学、乡村中学各4所，各选取2所学校7～18岁的学生为干预对象进行3个月的干预，剩余2所学校学生为对照，要求所有学校干预前均未进行类似肥胖干预项目。干预前共体检3 414名学生，干预结束后再次体检共3 296名学生，两次体检均参加的有效样本共3 175人，平均年龄(11.31±2.19)岁，其中干预对象共1 661人。本研究开始前经北京大学生物医学伦理委员会批准审查批准(IRB00001052-12048)，所有参加者及其监护人均签署知情同意书。

1.2　干预方法

本研究利用社会生态模型理论，从"学生-学校-家庭"三个方面对干预组学生进行3个月的肥胖干预，对照组在这3个月间不进行任何干预。干预措施分别是：(1)学生层面：利用健康教育课、肥胖指导手册、主题班会向学生进行健康教育，利用饮食运动行为记录表的[illegible]式督促行为；(2)学校层面：确保和利用大课间等提高学生体育活动时间，举办"健康饮食快乐运动"活动月，组织各种体育竞赛活动、征文征画活动、张贴海报、校园拒绝零食与含糖饮料、组建可爱嘟嘟训练营等；(3)家庭层面：利用家长讲座、分发宣传手册、倡议书等方法进行健康教育，并鼓励给孩子情感、环境和物质支持。

1.3　体格测量

身体形态指标包括身高、体重、腰围、臀围、腰臀比。身高、体重、臀围按2010年全国学生体质健康调研体检细则操作[7]，腰围以脐为测量点绕脐水平一周测量。体质指数(body mass index，BMI)的计算公式为体重(kg)/身高(m)^2，腰臀比(waist-hip ratio，WHR)的计算公式为腰围/臀围。根据中国肥胖工作组制定的标准对BMI进行分类，分为非超重肥胖、超重肥胖2种营养状况组[8]。

1.4　数据分析

利用SPSS 20.0软件进行数据分析，配对t检验分析干预前后研究对象身体形态指标、超重肥胖率的变化；t检验分析干预组与对照组的各指标前后差值是否有差异；卡方检验分析不同人群特征学生干预前后营养状况是否有变化；将干预与测量时间作为自变量，性别、年龄、地区作为协变量，是否超重肥胖这一二分类变量作为因变量，进行广义估计方程(generalized estimating equations，GEE)分析，若$OR>1$，则提示可以改善超重肥胖；将干预与测量时间作为自变量，性别、年龄、地区作为协变量，数值型变量作为因变量进行线性混合模型(linear mixed models，LMMs)分析，调整后差值差>0，说明干预组比对照组降低更多或上升更少，干预方法对这一指标有效。

2　结果

2.1　干预前后总体效果

本研究有效样本3 175人，其中干预组1 661人，对照组1 514人。干预后干预组与对照组的体重、BMI均上升，超重肥胖率干预前分别为35.1%、35.2%，干预后为33.9%、36.5%，与对照组相比干预组超重肥胖率有下降趋势，但差异无统计学意义($P>0.05$)。干预组干预后腰围、WHR下降，差异均有统计学意义。干预组与对照组比较腰围、WHR下降更多，对照组臀围下降，差异有统计学意义(表1)。GEE分析发现，干预可以改善超重肥胖($OR=1.12$，$P<0.05$)，LMMs分析发现干预组腰围、WHR调整后差值差下降更多，干预对这些指标有效(调整后的差值差=0.63、0.02，$P<0.05$，表2)。

2.2　性别差异

研究对象中共有男生1 651人，干预组与对照

?1994-2015 China Academic Journal Electronic Publishing House. All rights reserved.　http://www.cnki.net

组的超重肥胖率干预前分别为 40.0%、40.0%，干预后为 40.2%、41.9%，前后差异均无统计学意义；干预组 859 人，干预后腰围、WHR 下降，其余指标上升，差异有统计学意义；干预组与对照组比较腰围、WHR 下降更多（t = 3.00、6.29，P < 0.05）。女生 1 524人，干预组与对照组的超重肥胖率干预前分别为 29.8%、29.9%，干预后为 27.2%、30.6%，干预组干预后有下降趋势，但差异无统计学意义；干预组 802 人，干预后腰围、WHR 下降，体重与 BMI 上升，差异有统计学意义；干预组与对照组比较腰围、WHR 下降更多（t = 2.07、10.20，P < 0.05，表 2）。与男生相比，干预可以改善女生的超重肥胖（OR = 1.18，P < 0.05），男女生的腰围、WHR 调整后的差值差均下降更多（表 3）。

表 1 干预组与对照组干预前后各形态学指标的变化（$\bar{x} \pm s$）

Table 1 Changes of anthropometric indexes in intervention group and control group before and after intervention（$\bar{x} \pm s$）

Measure	Intervention group（n = 1 661）			Control group（n = 1 514）		
	Baseline	Follow-up	Difference	Baseline	Follow-up	Difference
Weight/kg	43.51 ± 16.34	45.51 ± 13.35*	2.00 ± 3.31	45.56 ± 15.57	47.05 ± 15.00*	1.49 ± 3.37#
BMI/（kg/m^2）	19.33 ± 4.55	19.85 ± 4.53*	0.52 ± 1.64	19.82 ± 4.45	20.27 ± 4.17*	0.45 ± 1.52
WC/cm	68.22 ± 13.25	67.79 ± 12.67*	−0.43 ± 3.85	68.58 ± 12.38	68.65 ± 12.17	0.07 ± 3.81#
HC/cm	81.15 ± 11.83	81.33 ± 11.81*	0.29 ± 3.81	82.63 ± 11.06	81.96 ± 11.37	−0.70 ± 3.20#
WHR	0.84 ± 0.06	0.83 ± 0.06*	−0.01 ± 0.04	0.83 ± 0.06	0.84 ± 0.06*	0.01 ± 0.04#

* P < 0.05, compare differences between baseline and follow-up. # P < 0.05, compare differences of baseline and follow-up between intervention group and control group. WC, waist circumference; BMI, body mass index; HC, hip circumference; WHR, waist-hip ratio.

2.3 城乡差异

研究对象中共有 1 559 名城市地区学生，干预组与对照组的超重肥胖率干预前分别为 35.9%、39.7%，干预后为 39.2%、40.5%，前后差异均无统计学意义；干预组 831 人，干预后腰围、WHR 下降，体重、BMI 上升，差异有统计学意义；干预组与对照组比较 WHR 下降更多（t = 4.71，P < 0.05）。乡村地区学生 1 616 人，干预组的超重肥胖率干预前分别为 34.3%、31.0%，干预后为 28.6%、32.8%，与对照组相比干预组干预后超重肥胖率降低，差异有统计学差异（χ^2 = 6.44，P < 0.05）；干预组 830 人，干预后腰围、WHR 下降，其余指标上升，差异有统计学意义；干预组与对照组比较腰围、WHR 下降更多（表 2）。与城市学生相比，干预可以改善乡村学生的超重肥胖（OR = 1.43，P < 0.05），乡村学生腰围、WHR 调整后差值差下降更多（表 3、表 4）。

2.4 非超重肥胖与超重肥胖学生体重差异

在干预前，超重肥胖学生 1116 人，干预组 583 人，干预后 88（15.1%）人 BMI 恢复正常，对照组 46 人（8.6%）恢复正常，差异有统计学意义（χ^2 = 11.01，P < 0.05）；干预组干预后腰围、WHR 下降，差异均有统计学意义；干预组与对照组比较 WHR 下降更多，差异有统计学意义。干预前非超重肥胖体重学生共 2 059 人，其中干预组 1 078 人，干预前后营养状况变化差异无统计学意义；干预组干预后 WHR 下降，体重、BMI、臀围上升；干预组与对照组比较腰围、WHR 下降更多（表 2）。

2.5 年级差异

小学生共 1 562 人，平均年龄（9.35 ± 1.28）岁，干预组与对照组的超重肥胖率干预前分别为 30.7%、36.7%，干预后为 30.4%、40.9%，与对照组相比干预组干预后超重肥胖率上升的少，但差异无统计学意义；干预组 875 人，干预后 WHR 下降，体重、BMI 上升，差异有统计学意义；干预组与对照组比较 WHR 下降更多，体重、BMI 上升更少，差异均有统计学意义。中学生 1 613 人，平均年龄（13.21 ± 0.75）岁，干预组与对照组的超重肥胖率干预前分别为 39.9%、34.0%，干预后为 37.8%、32.9%，前后差异均无统计学意义；干预组 786 人，干预后腰围、WHR 下降，其余指标均上升，差异有统计学意义。干预组与对照组比较腰围、WHR 下降更多（表 2）。干预可以改善小学生的超重肥胖（OR = 1.21，P < 0.05），中小学生 WHR 调整后差值差均下降更多（表 3、表 4）。

3 讨论

从 2007 年起美国亚利桑那州在美国疾病预防与控制中心社区改变项目的基础上做了对城市及农村地区儿童保健环境干预改善的研究[9]，从健康教育、改善儿童的饮食运动行为等方面入手，与本研究相似。荣湘江等[10]为期 3 个月的干预期中超重肥胖率及体脂率下降，Danielsen 等[11]在为期 3 个月的以家庭为基础的干预中对于体重的效果较为明显，说明 3 个月的干预期对肥胖干预来说可以取得一定的效果。

?1994-2015 China Academic Journal Electronic Publishing House. All rights reserved. http://www.cnki.net

表 2　不同性别、地区、年级间干预组与对照组干预前后各形态学指标的变化($\bar{x}\pm s$)

Table 2　Changes of anthropometric indexes in different Gender, region and grade of intervention group and control group before and after intervention ($\bar{x}\pm s$)

Characteristic		Weight/kg		BMI/(kg/m^2)		WC/cm		HC/cm		WHR	
		Control group	Intervention group	Control group	Intervention group	Control group	Intervention group	Control group	Intervention group	Control group	Intervention group
Gender											
Boy (n = 1 651)	Baseline	45.60 ± 17.75	47.15 ± 16.58	19.80 ± 4.79	20.20 ± 4.65	69.78 ± 13.85	69.92 ± 13.15	81.83 ± 12.11	82.71 ± 11.18	0.85 ± 0.06	0.84 ± 0.06
	Follow-up	47.81 ± 8.30*	48.72 ± 15.88*	20.34 ± 4.75*	20.65 ± 4.31*	69.25 ± 3.46*	69.97 ± 12.87	82.18 ± 12.30*	82.93 ± 11.59*	0.84 ± 0.06*	0.84 ± 0.06
	Difference	2.21 ± 3.67	1.57 ± 3.56#	0.53 ± 1.82	0.44 ± 1.60	−0.54 ± 4.33	0.06 ± 3.58#	0.63 ± 4.37	0.21 ± 3.00#	−0.01 ± 0.04	0.00 ± 0.04#
Girl (n = 1 524)	Baseline	41.27 ± 14.36	43.82 ± 14.19	18.82 ± 4.21	19.40 ± 4.19	66.54 ± 11.73	67.13 ± 11.26	80.43 ± 11.49	82.54 ± 10.93	0.83 ± 0.06	0.81 ± 0.05
	Follow-up	43.06 ± 14.56*	45.22 ± 13.77*	19.33 ± 4.22*	19.86 ± 3.96*	66.23 ± 11.52*	67.21 ± 11.01	80.41 ± 11.20	80.91 ± 11.03*	0.82 ± 0.06*	0.83 ± 0.06*
	Difference	1.79 ± 2.86	1.39 ± 3.13#	0.50 ± 1.42	0.46 ± 1.43	−0.31 ± 3.26	0.08 ± 3.78#	−0.07 ± 3.06	−1.67 ± 3.13#	−0.01 ± 0.04	0.02 ± . 0.05#
Region											
Urban (n = 1 559)	Baseline	44.86 ± 16.84	46.91 ± 15.95	19.66 ± 4.67	20.19 ± 4.60	67.29 ± 2.92	70.05 ± 12.49	82.43 ± 12.20	84.20 ± 10.97	0.84 ± 0.07	0.83 ± 0.06
	Follow-up	47.34 ± 17.31*	48.40 ± 15.63*	20.33 ± 4.63*	20.58 ± 4.32*	68.73 ± 12.86*	69.70 ± 12.46*	82.35 ± 11.81	83.78 ± 10.98*	0.83 ± 0.06*	0.83 ± 0.06*
	Difference	2.47 ± 3.16	1.48 ± 2.75#	0.67 ± 1.57	0.38 ± 1.26#	−0.41 ± 4.40	−0.34 ± 3.92	0.21 ± 4.53	−0.45 ± 3.27#	−0.01 ± 0.04	0.002 ± 0.04#
Rural (n = 1 616)	Baseline	42.16 ± 15.73	44.31 ± 15.12	19.00 ± 0.79	19.47 ± 4.29	67.29 ± 2.92	67.21 ± 12.13	79.88 ± 11.32	81.17 ± 10.95	0.84 ± 0.06	0.83 ± 0.06
	Follow-up	43.69 ± 16.00*	45.80 ± 14.31*	19.36 ± 0.75*	19.98 ± 4.00*	66.85 ± 12.41*	67.66 ± 11.81*	80.31 ± 11.73*	80.27 ± 11.46*	0.83 ± 0.07*	0.84 ± 0.06*
	Difference	1.54 ± 3.40	1.49 ± 3.85	0.36 ± 1.70	0.51 ± 1.73	−0.44 ± 3.22	0.45 ± 3.40#	0.37 ± 2.92	−0.92 ± 3.13#	−0.01 ± 0.04	0.02 ± 0.04#
Grade											
Primary school (n = 1 562)	Baseline	33.74 ± 9.78	35.63 ± 10.42	17.75 ± 3.64	18.34 ± 3.82	62.32 ± 10.40	63.27 ± 11.07	74.18 ± 8.65	75.78 ± 9.01	0.84 ± 0.06	0.83 ± 0.06
	Follow-up	35.09 ± 9.88*	37.61 ± 10.25*	18.07 ± 3.58*	19.10 ± 3.65*	62.14 ± 10.15	63.13 ± 11.59	74.21 ± 8.62	74.68 ± 9.25*	0.83 ± 0.06*	0.84 ± 0.06*
	Difference	1.35 ± 3.24	1.98 ± 2.61#	0.32 ± 1.82	0.76 ± 1.42#	−0.18 ± 4.28	−0.14 ± 3.12	0.29 ± 4.36	−1.13 ± 2.88#	−0.01 ± 0.04	0.01 ± 0.04#
Secondary school(n = 1 613)	Baseline	54.39 ± 15.28	53.82 ± 14.27	21.09 ± 4.81	21.05 ± 4.57	74.82 ± 12.54	72.97 ± 11.64	88.89 ± 9.93	88.31 ± 9.23	0.84 ± 0.06	0.82 ± 0.06
	Follow-up	57.12 ± 15.16*	54.89 ± 13.77*	21.83 ± 4.65*	21.24 ± 4.32*	74.11 ± 12.20*	73.22 ± 11.33	89.24 ± 9.65*	88.01 ± 9.22*	0.83 ± 0.06*	0.83 ± 0.06*
	Difference	2.73 ± 3.24	1.07 ± 3.84#	0.74 ± 1.38	0.19 ± 1.55#	−0.71 ± 3.30	0.24 ± 4.08#	0.29 ± 3.09	−0.34 ± 3.41	−0.01 ± 0.04	0.01 ± 0.04#
Nutrition status											
Normal (n = 2 059)	Baseline	36.62 ± 10.60	39.03 ± 10.40	16.85 ± 2.16	17.32 ± 2.19	61.51 ± 8.01	62.12 ± 7.77	76.02 ± 8.80	77.95 ± 8.54	0.81 ± 0.05	0.80 ± 0.04
	Follow-up	38.79 ± 11.41*	40.94 ± 10.15*	17.53 ± 2.40*	18.01 ± 2.09*	61.44 ± 7.50	62.66 ± 8.54*	76.29 ± 9.02*	77.32 ± 8.89*	0.807 ± 0.05*	0.81 ± 0.05*
	Difference	2.17 ± 2.95	1.91 ± 3.06	0.68 ± 1.42	0.68 ± 1.41	−0.07 ± 3.58	0.54 ± 3.73#	0.38 ± 3.72	−0.65 ± 3.55#	−0.01 ± 0.05	0.01 ± 0.05#
Overweight/ obesity (n = 1 116)	Baseline	56.26 ± 17.42	57.95 ± 16.33	23.91 ± 4.23	24.42 ± 3.86	80.66 ± 12.00	80.48 ± 10.68	90.65 ± 10.82	91.24 ± 9.95	0.89 ± 0.05	0.88 ± 0.05
	Follow-up	57.69 ± 17.94*	58.29 ± 15.98*	24.14 ± 4.41*	24.43 ± 3.81	79.57 ± 11.84*	79.67 ± 11.05*	90.66 ± 10.64	90.51 ± 10.44*	0.88 ± 0.06*	0.88 ± 0.06
	Difference	1.71 ± 3.88	0.70 ± 3.75#	2.23 ± 1.95	0.02 ± 1.62	−1.08 ± 4.25	−0.81 ± 3.81	0.12 ± 3.97	−0.78 ± 3.28#	−0.01 ± 0.04	0.001 ± 0.05#

* $P<0.05$, compare differences between baseline and follow-up. # $P<0.05$, compare differences of baseline and follow-up between intervention group and control group. WC, waist circumference; BMI, body mass index; HC, hip circumference; WHR, waist-hip ratio.

?1994-2015 China Academic Journal Electronic Publishing House. All rights reserved.　http://www.cnki.net

表 3 干预对不同性别学生是否有效(GEE 与 LMMs 分析)

Table 3 Intervention effect in different genderstudents (GEE analysis and LMMs analysis)

Measure	Urban			Rural			Total
	Primary school	Secondary school	Total	Primary school	Secondary school	Total	
Boy							
Weight adjusted difference/kg	0.24(−0.18 − 0.65)	−2.13(−2.86 − −1.41)*	−0.9(−1.33 − −0.47)*	1.86(1.01 − 2.74)*	−1.63(−2.30 − −0.96)*	0.14(−0.41 − 0.69)	−0.38(−0.73 − −0.03)*
BMI, adjusted difference/(kg/m^2)	0.13(−0.14 − 0.40)	−0.72(−1.02 − −0.42)*	−0.29(−0.50 − −0.09)*	1.00(0.54 − 1.45)*	−0.54(−0.80 − −0.27)*	0.18(−0.08 − 0.44)	−0.05(−0.22 − 0.11)
WC adjusted difference/cm	−0.59(−1.63 − 0.45)	2.21(1.42 − 3.00)*	0.83(0.17 − 1.49)*	0.07(−0.51 − 0.65)	0.90(0.30 − 1.51)*	0.63(0.21 − 1.05)*	0.73(0.34 − 1.11)*
HC adjusted difference/cm	−0.43(−2.82 − 1.96)	1.15(−1.63 − 3.92)	0.40(−1.44 − 2.24)	−0.29(−0.29 − 1.98)	−0.89(−3.50 − 1.71)	−0.34(−2.09 − 1.41)	0.03(−1.24 − 1.31)
WHR adjusted difference	0.01(−0.00 − 0.03)	0.01(−0.01 − 0.03)	0.01(0.00 − 0.02)*	0.01(−0.01 − 0.02)	0.02(0.00 − 0.04)*	0.01(−0.00 − 0.02)	0.01(0.00 − 0.02)*
Nutrition status, *OR*	1.04(0.83 − 1.30)	0.77(0.62 − 0.95)#	0.89(0.77 − 1.04)	1.38(1.05 − 1.81)#	1.31(1.01 − 1.70)#	1.32(1.10 − 1.59)#	1.08(0.96 − 1.22)
Girl							
Weight adjusted difference/kg	−0.29(−0.81 − 0.23)	−1.14(−1.76 − −0.51)*	−0.64(−1.05 − −0.24)*	1.31(0.87 − 1.75)*	−0.91(−1.65 − −0.16)*	0.27(−0.17 − 0.71)	−0.18(−0.48 − 0.12)
BMI adjusted difference/(kg/m^2)	−0.05(−0.34 − 0.25)	−0.43(−0.68 − −0.17)*	−0.21(−0.41 − −0.02)*	0.76(0.52 − 1.01)*	−0.39(−0.71 − −0.06)*	0.19(−0.02 − 0.39)	−0.01(−0.15 − 0.13)
WC adjusted difference/cm	0.5(−0.30 − 1.30)	−1.68(−2.36 − −1.00)*	−0.54(−1.07 − −0.01)*	0.62(0.01 − 1.22)*	2.24(1.46 − 3.02)*	1.48(0.98 − 1.97)*	0.52(0.15 − 0.89)*
HC adjusted difference/cm	−0.88(−2.96 − 1.19)	−1.04(−3.52 − 1.45)	−1.10(−2.74 − 0.53)	−2.46(−4.71 − −0.21)*	−2.02(−4.36 − 0.32)	−1.96(−3.61 − −0.32)	−1.54(−2.70 − 0.34)*
WHR adjusted difference	0.02(0.00 − 0.04)*	−0.01(−0.03 − 0.00)	0.01(−0.01 − 0.02)	0.03(0.02 − 0.05)*	0.05(0.03 − 0.06)*	0.04(0.03 − 0.05)*	0.02(0.02 − 0.03)*
Nutrition status, *OR*	0.94(0.81 − 1.39)	0.73(0.58 − 0.93)#	0.89(0.74 − 1.06)	1.48(1.11 − 1.96)#	1.68(1.24 − 2.26)#	1.57(1.28 − 1.92)#	1.18(1.03 − 1.35)#

* $P<0.05$, linear mixed models (LMMs) analysis, adjusted difference ≠ 0. #$P<0.05$, generalized estimating equations (GEE) analysis, OR ≠ 1. CI, confidence interval; WC, waist circumference; BMI, body mass index; HC, hip circumference; WHR, waist-hip ratio; *OR*, odds ratio.

经过 3 个月的干预，干预组的腰围、WHR 较对照组下降，这个结果也出现在不同性别间、不同地区间、不同学校间，说明这些中心性肥胖指标可能对干预较敏感。干预组与对照组的体重、BMI 均上升，原因是儿童正处于生长发育时期，体重增加是一个自然的过程，干预后测量时期处于冬季，冬季人体体重也会自然增加，且初期减重因为肌肉的增加体重不会过多减轻，因此在儿童肥胖干预效果评价中，不能将体重、BMI 作为最重要的指标，而腰围、WHR 等中心性肥胖指标相对更敏感[12]。同样，在女生分类中出现了干预组与对照组相比臀围有上升的情况，原因也可能是干预组运动增加所致。

男、女生干预效果存在差异。与男生相比，干预对女生的超重肥胖有保护作用，干预对女生的作用大于男生，与王红等[13]以健康促进的学校干预方式进行干预 10 个月的研究结果相符。可能是女生更愿意参加并完成干预[14]，也可能是男生对体形认识有误，对自身体重控制意识不如女生强烈，家人及社会对他们的体形容忍度更大有关[15]。在下一步的肥胖防控工作中应更加重视男生的防控工作。但是城市女中学生的腰围上升，与其余人群不同，原因仍需在以后研究中进一步探索。

城市和乡村学生干预效果存在差异。城市干预组 WHR 一项指标下降比对照组多，差异有统计学意义。而乡村学校干预组腰围、WHR 指标均较对照组有所改善，GEE 结果显示干预对乡村学生超重肥胖降低是有效的。本干预方法对乡村学校更有效，可能原因是城市经济水平高于乡村[16]，城市学生零食消费率高于乡村学生[17]，提示可能与城市学生在家中吃零食更多有关[18]，具体原因仍需进一步研究探讨，也提示在以后的干预中应设法加强城市学生的干预。

中小学生中干预组的 WHR 指标较对照组有所改善，干预是小学生是否超重肥胖的保护因素。从这一结果中可以看出干预对小学生的效果较中学生更好，原因可能是小学生的依从性较好[19]，中学生由于课业压力较重[20]，进行体育锻炼及参与干预的时间较少所致。如何提高中学生在繁重课业中增加体育锻炼时间提高干预效果是今后工作中应该注意的问题，同时也提示肥胖干预应“从小抓起”。

国内学者肥胖干预大多只针对已经超重肥胖的学生，而本研究对象还包括非超重肥胖学生，用以研究干预在全学生人群中的作用，为今后在学校范围内推荐该干预措施提供依据。本研究显示非超重肥

?1994-2015 China Academic Journal Electronic Publishing House. All rights reserved. http://www.cnki.net

胖学生的腰围、WHR 均有所改善，超重肥胖学生营养状况改善为正常的比例干预组(15.1%)大于对照组(8.6%)，说明干预不仅对非超重肥胖学生有效，对超重肥胖的学生更有效。因此，在对全体学生进行干预的基础上对超重肥胖学生进行加强干预的方法是有效的，这为适合全学校学生参与的肥胖干预措施的实施提供了依据。同时，本研究对干预对象的人群特征进行了分析，为今后的预防工作提供了依据。此外，本研究采用 GEE 与 LMMs 判断干预方法对学生肥胖防控是否有效，在进行重复测量资料分析时更加适用并可减少偏倚[21-22]。

综上所述，"学生-学校-家庭"社会生态模型对学生的腰围、WHR 等中心性肥胖指标干预有效，干预效果女生优于男生，乡村优于城市，小学优于中学。在今后的儿童青少年肥胖干预工作中应该重视学生、学校、家庭综合干预方式的应用。

表 4　干预对不同地区、年级学生是否有效(GEE 与 LMMs 分析)

Table 4　Intervention effect in different region and gradestudents (GEE analysis and LMMs analysis)

Index	Urban	Rural	Primary school	Secondary school
Weight adjusted difference/kg	−0.77 (−2.50 −0.96)	0.20 (−1.41 −1.82)	0.78 (−0.50 −2.05)	−1.51 (−3.46 −0.43)
BMI adjusted difference/(kg/m^2)	−0.25 (−0.85 −0.34)	0.18 (−0.37 −0.74)	0.46 (−0.05 −0.97)	−0.53 (−1.16 −0.10)
WC adjusted difference/cm	0.45 (−1.14 −2.03)	1.05 (0.47 −2.51)*	0.40 (−1.01 −1.81)	0.95 (−0.69 −2.58)
HC adjusted difference/cm	−0.30 (−1.54 −0.94)	−1.13 (−2.34 −0.08)	−1.00 (−2.12 −0.14)	−0.67 (−1.96 −0.61)
WHR adjusted difference	0.01 (0.00 −0.02)*	0.02 (0.016 −0.03)*	0.02 (0.01 −0.03)*	0.02 (0.01 −0.03)*
Nutrition status, *OR*	0.89 (0.79 −1.01)	1.43 (1.24 −1.63)#	1.21 (1.07 −1.38)#	1.04 (0.92 −1.18)

* $P<0.05$, LMMs analysis, adjusted difference≠0. # $P<0.05$, GEE analysis, *OR*≠1. WC, waist circumference; BMI, body mass index; HC, hip circumference; WHR, waist-hip ratio; *OR*, odds ratio.

参考文献

[1] Ji CY, Chen TJ. Empirical changes in the prevalence of overweight and obesity among Chinese students from 1985 to 2010 and corresponding preventive strategies [J]. Biomed Environ Sci, 2013, 26(1): 1-12.

[2] Mcleroy KR, Bibeau D, Steckler A, et al. An ecological perspective on health promotion programs [J]. Health Educ Q, 1988, 15(4): 351-377.

[3] Yin Z, Moore JB, Johnson MH, et al. Micro- and macro-level correlates of adiposity in children [J]. J Public Health Manag Pract, 2012, 18(5): 445-452.

[4] Kellou N, Sandalinas F, Copin N, et al. Prevention of unhealthy weight in children by promoting physical activity using a socio-ecological approach: What can we learn from intervention studies [J]. Diabetes Metab, 2014, 40(4): 258-271.

[5] 彭佳林. 艾滋病健康教育和行为干预效果评价指标体系的基本框架探讨 [J]. 医学与社会, 2007, 20(9): 1-2.

[6] 曾永忠, 赵苏喆. 促进身体锻炼行为的社会生态理论模型研究述评 [J]. 福建体育科技, 2008, 27(1): 20-22.

[7] 中国学生体质与健康研究组. 2010 年中国学生体质与健康调研报告 [M]. 北京: 高等教育出版社, 2012: 32-47.

[8] 中国肥胖问题工作组. 中国学龄儿童青少年超重、肥胖筛查体重指数值分类标准 [J]. 中华流行病学杂志, 2004, 25(2): 10-15.

[9] Drummond RL, Staten LK, Sanford MR, et al. A pebble in the pond: the ripple effect of an obesity prevention intervention targeting the child care environment [J]. Health Promot Pract, 2009, 10(Suppl 2): S156-S167.

[10] 荣湘江, 朱稼霈, 张世伟, 等. 运动干预青少年单纯性肥胖效果的研究 [J]. 中国康复医学杂志, 2007, 22(8): 702-705.

[11] Danielsen YS, Nordhus IH, Juliusson PB, et al. Effect of a family-based cognitive behavioural intervention on body mass index, self-esteem and symptoms of depression in children with obesity (aged 7-13): a randomised waiting list controlled trial [J]. Obes Res Clin Pract, 2013, 7(2): e116-e128.

[12] Tipton KD, Wolfe RR. Exercise, protein metabolism, and muscle growth [J]. Int J Sport Nutr Exerc Metab, 2001, 11(1): 109-132.

[13] 王红, 程茂金, 周铌, 等. 健康促进学校对小学生肥胖的群体干预效果评价 [J]. 中国学校卫生, 2008, 29(11): 1029-1030.

[14] Fagg J, Cole T J, Cummins S, et al. After the RCT: who comes to a family-based intervention for childhood overweight or obesity when it is implemented at scale in the community [J]. J Epidemiol Community Health, 2015, 69(2): 142-148.

[15] 崔馨月, 陈天娇, 王海俊, 等. 北京市房山区中小学生自我体形评价分析 [J]. 中国学校卫生, 2014, 35(9): 1355-1358.

[16] 宋艳伟. 北京城乡经济统筹发展水平评价研究 [D]. 北京: 首都经济贸易大学, 2010.

[17] 于冬梅, 张兵, 赵丽云, 等. 中国 3~17 岁儿童青少年零食消费状况 [J]. 卫生研究, 2008, 37(6): 710-713.

[18] 刘爱玲, 李艳平, 郝利楠, 等. 我国 7 城市中小学生零食消费行为分析 [J]. 中国健康教育, 2009, 25(9): 650-653.

[19] 梁碧绿, 郭赐贶, 蔡健生, 等. 低龄小学生家庭视力健康促进效果研究 [J]. 中国学校卫生, 2010, 31(9): 1068-1070.

[20] 陈传锋, 陈文辉, 董国军, 等. 中学生课业负担过重: 程度、原因与对策: 基于全国中学生学习状况与课业负担的调查 [J]. 中国教育学刊, 2011(7): 11-16.

[21] 陈丹萍, 赵耐青, 林燧恒. 分层整群随机抽样数据的不同分析方法及结果比较 [J]. 中国卫生统计, 2010, 27(2): 122-124.

[22] 安胜利, 张燕虹, 陈征. 应用 SPSS 软件实现二分类重复测量资料的 GEE 及 GLMMs 分析 [J]. 南方医科大学学报, 2012, 32(12): 1777-1780.

(2015-01-10 收稿)
(本文编辑: 王　蕾)

?1994-2015 China Academic Journal Electronic Publishing House. All rights reserved.　http://www.cnki.net
