# Supplementary material for: Systematic review and meta-analysis of school-based obesity interventions in mainland China
Source: PLoS One. 2017 Sep 14;12(9):e0184704. doi: 10.1371/journal.pone.0184704 (PMC5598996; doi:10.1371/journal.pone.0184704)
Supplement: S1 Dataset — (ZIP) [file pone.0184704.s007.zip › S1_dataset/76库/76.pdf]

文章编号：1003-8507(2010)12-2225-03 中图分类号：R723.14 文献标识码：A

【儿少卫生与妇幼保健】

徐州市儿童肥胖干预研究

吴秀娟[1]，王健[2]，董华[2]，耿跃春[2]，张训保[1]，邵继红[1]，孙桂香[1]

摘要：［目的］了解儿童肥胖现状，探讨控制儿童肥胖的有效措施。［方法］采用多级整群分层抽样，对 8~13 岁 15 119 名学生进行问卷调查、体格检查等，并从中抽取单纯性超重与肥胖学生 208 名进行干预研究，采用健康教育+有氧运动+合理营养综合干预措施为主要内容，对减肥前、后的行为、形态指标进行干预研究。［结果］干预后的超重与肥胖儿童行为、形态变化较干预前与对照组相比差异有统计学意义（$P<0.05$）。［结论］综合干预措施对防治儿童超重与肥胖是安全有效的。

关键词：儿童；肥胖；干预研究

CHILDHOOD OBESITY INTERVENTION STUDY IN XUZHOU *WU Xiu-juan，WANG Jian，DONG Hua，et al.（Xuzhou Medical College，Xuzhou 221002，China）*

Abstract：［Objective］To understand the current situation of children' s obesity，and explore the effective action of controlling children's obesity.［Methods］Using the multistage group stratified sampling，we carried out questionnaire investigation and medical examination among 15 119 students at the age of 8 to 13 year old，and extracted 208 students with retinitis simplex overload and obesity to conduct intervention research. The health education + oxygen movement + reasonable nutrition synthesis intervention measure were used as the primary measurement，and the behavior and shape indexes before and after losing weight were studied.［Results］The behavior and shape indexes of children with overweight and the obese after intervention had significant difference with the control group（$P<0.05$）.［Conclusion］The synthesis intervention measure is safe and effective in preventing and controlling the child overweight and obesity.

Key words：Children；Obesity；Intervention study

肥胖问题作为全球性的公共卫生问题，已引起社会各界的关注，尤其是儿童青少年的肥胖问题更为突出，预防和控制儿童肥胖已成为当务之急[1]。为进一步探讨徐州市超重与肥胖儿童减肥现状，我们课题组采用健康教育+有氧运动+合理营养综合减肥模式为主要内容，于 2006 年 9 月~2007 年 7 月对徐州市部分中、小学展开了调查和超重与肥胖实验效果的研究。

1 对象与方法

1.1 研究对象

采用多级整群分层抽样的方法。在市区相当规模的中、小学中随机抽取 10 所学校 8~13 岁 15 119 名学生进行问卷调查、体格检查等，对所筛选出的超重肥胖儿童进行正规体检，剔除因内分泌疾病、药物副作用等引起的肥胖儿童，最后确定 208 人为研究对象，其中 110 人作为实验组，98 人作为对照组。实验对象均为单纯性超重肥胖学生。

1.2 干预方法

采用综合干预措施为主要内容。干预内容：① 健康知识教育：与学校、家长合作，对家长和学生集中授课、专家咨询、设立专题宣传栏、发放自编的宣传手册等方式，宣传《中国居民膳食指南》和肥胖防治知识。② 有氧运动：每次体育课或课外活动 15~20 min，运动频率为每周 3~5 次，运动强度达到本人最高心率的 70%~80%。③ 合理营养：指导超重肥胖儿童家长调整饮食结构，各营养素按比例摄入；合理分配 3 餐，控制总热量。教育学生改变不良生活方式：如少吃油煎、油炸食品，少吃甜食及饮料，避免暴饮暴食、多食少动、睡前进餐等不良习惯。

由研究者和经过培训的医学生定期测量体重、心率、血压、生理、生化等指标，同时对整个干预试验进行全面医学观察和监控。实验组严格按照计划执行，干预时间为 5 个月。对照组不接受干预。

1.3 诊断标准

采用体重指数（BMI）法，BMI=体重(kg)/身高(m)^2，以 2003 年 11 月全国儿童肥胖学术会议公布的按年龄、性别的 BMI 标准为依据判定正常、超重和肥胖。

1.4 统计学分析

用 EPIDATE3.0 双份录入计算机，经核对、逻辑纠错后建立数据库，用 SPSS13.0 软件进行数据处理及统计分析。

2 结果

2.1 儿童超重与肥胖现状调查

调查结果显示，男生 8~13 岁儿童超重的检出率为 16.50%（1 117/8 096），肥胖检出率为 13.80%；女生 8~13 岁超重的检出率为 9.81%（689/7 023），肥胖检出率为 9.31%，男生高于

基金项目：江苏省科技厅社发项目基金科研课题（BS2006508）

作者简介：吴秀娟（1960-），女，学士，副教授，研究方向：流行病学科研和教学

通讯作者：王健，E-mail：kbt5600532@e165. com

作者单位：1.徐州医学院公共卫生学院，徐州，221002；2.徐州医学科学研究所

?1994-2015 China Academic Journal Electronic Publishing House. All rights reserved. http://www.cnki.net

女生。通过比较各年龄组肥胖检出率，男生肥胖检出率最高年龄组在 12~13 岁，女性肥胖检出率最高年龄组在 8~9 岁。见表 1。

表 1　青少年学生超重与肥胖的检出率（$\times10^{-2}$）

年龄（岁）	男				女			
	调查人数	超重人数	肥胖人数	肥胖检出率	调查人数	超重人数	肥胖人数	肥胖检出率
8～9	3168	495	463	14.61	3267	328	342	10.47
10～11	3059	446	372	12.16	2143	203	185	8.63
12～13	1869	395	282	15.09	1613	158	127	7.87
合计	8096	1336	1117	13.80	7023	689	654	9.31

2.2　干预前、后超重与肥胖儿童的饮食、运动行为的变化比较

干预组干预后吃饭速度、睡觉前进食、经常吃甜食、油炸食品等饮食行为的改变较干预前及对照组相比差异有统计学意义（干预组干预前后比较 χ^2 = 19.84~39.97，P < 0.01；干预组干预后与对照组比较 χ^2 = 5.01~39.09，P < 0.05）。干预组与对照组学生大多喜欢上体育课，干预组干预前后比较 χ^2 = 9.50，P < 0.01。见表 2。

表 2　干预前、后超重与肥胖儿童的饮食、运动行为的变化比较（$\times10^{-2}$）

调查项目	干预组（n = 110）				对照组（n = 98）			
	干预前		干预后		干预前		干预后	
	人数	率	人数	率	人数	率	人数	率
吃早餐习惯	56	50.9	61	55.5	45	45.9	48	49.0
吃饭速度快	96	87.3	52	47.3[ab]	76	77.6	64	65.3
睡觉前进食	69	62.7	36	32.7[ac]	50	51.0	47	48.0
常吃甜食	92	83.6	56	50.9[ab]	71	72.4	82	83.7
常吃油炸食品	102	92.7	71	64.5[ab]	90	91.8	88	89.8
喜欢上体育课	89	80.9	104	94.5	83	84.7	90	91.8

注：与干预前比较，[a]P< 0.01；与对照组比较，[b]P< 0.01；[c]P< 0.05

2.3　干预前、后超重与肥胖儿童身体形态变化比较

经过 5 个月的干预实验，超重与肥胖儿童在体重、腰围、臀围、BMI 发生了很大变化，干预组干预前后比较差异有统计学意义（t = 7.87~22.34，P < 0.01），干预后干预组与对照组比较这些指标差异亦有统计学意义（t = 10.01~22.46，P < 0.01），而对照组在干预前后这些指标无统计学意义（P > 0.05），见表 3。

表 3　2 组干预前后超重与肥胖儿童身体形态变化的比较（$\bar{x} \pm s$）

指标	干预组（n = 110）		对照组（n = 98）	
	干预前	干预后	干预前	干预后
体重（kg）	67.36 ± 8.53	58.42 ± 8.31[ab]	70.31 ± 7.65 ..	69.61 ± 7.75
腰围（cm）	85.07 ± 3.67	74.18 ± 3.56[ab]	85.47 ± 3.86	84.68 ± 3.62
臀围（cm）	93.08 ± 1.34	89.56 ± 1.25[ab]	93.86 ± 1.67	93.64 ± 1.37
BMI（kg/m^2）	29.14 ± 2.50	24.75 ± 2.36[ab]	29.08 ± 2.55	28.52 ± 2.53

注：与干预前比较，[a]P< 0.01；与对照组比较，[b]P< 0.01

3　讨论

调查结果显示，徐州市儿童的超重与肥胖现象较为严重，超重与肥胖的检出率分别为男生 16.50%、13.80%；女生 9.81%、9.31%，已接近我国发达地区 2000 年城市儿童超重与肥胖的检出率[2]。男生肥胖检出率最高年龄组在 12~13 岁，女性肥胖检出率最高年龄组在 8~9 岁，该年龄段正处于童年期向青春前期的过渡阶段，青春前期的儿童是预防超重和肥胖的关键时期，且男生的干预任务更加艰巨。儿童肥胖重在多因素综合防治，本研究通过改变儿童的饮食结构和习惯、坚持有氧运动锻炼、改善儿童不良习惯等方式入手，对儿童进行综合干预收到了良好的效果。

首先对肥胖儿童进行饮食方面的干预。干预组干预前有吃饭速度快、经常吃甜食、睡觉前进食、常吃油炸食品等饮食习惯，这些不良的饮食习惯导致总热量、脂肪摄入过高，而膳食纤维和微量营养素却明显不足。因此，从饮食结构的改善着手，积极限制总热量、高脂饮食的摄入，降低脂肪热能比，提高饮食纤维和营养素的摄入量，以饮食指南为标准，为肥胖儿童制定健康食谱。通过我们采取干预措施后，干预组干预后饮食结构，吃饭速度、睡觉前进食、经常吃甜食、油炸食品等饮食行为的改变较干预前及对照组相比差异有统计学意义，说明干预是有效的。

坚持有氧运动是控制和预防肥胖发生至关重要的措施。傅兰英等认为[3]对肥胖者而言有氧运动是客观生活中最活跃，也

（下转第 2230 页）

?1994-2015 China Academic Journal Electronic Publishing House. All rights reserved. http://www.cnki.net

敏等情况，如有异常及时报告医生进行处理。孕妇如有躁动，注意协助固定，防止子宫穿孔。用吸痰管及纱布及时擦拭孕妇口鼻分泌物或呕吐物，保持呼吸道通畅。（3）术后护理：术后帮助孕妇擦净血迹和消毒液，在受压处给予适当的按摩。术后 10 min 左右孕妇一般会完全清醒，密切观察 30 min，有无头晕、恶心、呕吐、乏力等症状。帮助孕妇穿戴好衣服，到观察室进行充分休息，注意保暖。同时，做好术后宣教，耐心详细的教导孕妇洗浴、清洗外阴、抗生素使用、避孕方法等注意事项，预防感染的发生。术后 1 个月进行复诊。

1.2.3 观察指标及判定标准 （1）镇痛效果：根据病人的自我感觉和表现将疼痛程度分为 4 级[4]：Ⅰ级（无痛）、Ⅱ级（轻度）、Ⅲ级（中度）、Ⅳ级（重度）。将Ⅰ、Ⅱ级判定为镇痛有效，Ⅲ、Ⅳ级判定为镇痛无效。（2）人工流产综合征（PAAS）判定标准[5]：病人出现心动过缓（≤60 次/min）、心律失常、血压下降（ > 20 mmHg）、脉搏变慢（ > 20 bpm）、面色苍白、头晕、胸闷等症状。（3）宫颈扩张程度判定标准：以 6 号扩宫器无阻力一次性顺利通过为有效。（4）出血判定标准：用 30 ml 量杯测量。

2 结果

在异丙酚静脉麻醉行无痛人工流产术中通过精心、舒适护理，孕妇镇痛效果有效率 100%，无人工流产综合征发生，平均手术时间（3.8 ± 0.9）min，平均出血量为（19.4 ± 4.2）ml，无其他不良反应及手术并发症发生，孕妇在生理和心理方面均得到很大改善。

3 讨论

人工流产作为避孕失败的补救措施，早已被人们广泛接受，已成为现今意外怀孕后的首要选择。传统的人工流产手术会给病人带来身体和心理的双重痛苦，越来越多的病人选择无痛人工流产术。静脉麻醉下行无痛人工流产术，病人安静、无痛，而且手术时间短、出血量低，大大减少了手术的风险和人工流产综合征的发生。

异丙酚作为快速短效的麻醉药，具有操作方便、起效快、镇痛作用强、术后苏醒迅速、副作用小等优点，可以缩短手术时间，减少出血量，避免人工流产综合征，术后不抑制子宫收缩，同时也大大减轻了护士的工作强度，特别适用于人工流产，是人工流产较为理想的麻醉方法[6]。但是异丙酚在手术中会引起血压、脉搏下降、短暂性呼吸暂停等情况，有关研究表明不同心理状态异丙酚的麻醉效果有很大差异[7]，因此对孕妇应加强术前心理护理，消除恐惧紧张情绪，异丙酚麻醉应由专业麻醉师操作并监测，在手术时必须严密观察生命体征予以精心护理，确保手术顺利进行。

总之，异丙酚静脉麻醉下行无痛人工流产术是一种无痛、安全、有效的人流方法。做好充分的术前准备和心理护理，术中严密观察、精心护理，术后人性化抚慰、宣讲是保障无痛人工流产术成功的关键。

参考文献：

[1] 崔长虹. 268 例无痛人工流产术患者的护理［J］. 山东医药，2008，48（33）：89.

[2] 余红. 无痛人工流产术的护理体会［J］. 现代中西医结合杂志，2008，17（35）：5535.

[3] 李颖，杜俊英，宋俊兰. 异丙酚复合芬太尼静脉麻醉行无痛术的护理［J］. 中国误诊学杂志，2008，8（32）：7970-7971.

[4] Tramer M. Propofol anaesthesia and post operative nausea and vomiting：quantitative systematize review of randomized controlled studies［J］. Can J Anaesth，1997，44：225-247.

[5] 薄雪梅，魏恩江. 异丙酚复合芬太尼在妇科门诊人工流产术中的应用［J］. 现代中西医结合杂志，2007，16（36）：5 493

[6] 袁春红，唐爱萍，茅志娟. 丙泊芬静脉麻醉用于无痛人流的护理［J］. 中国误诊学杂志，2007，7（14）：3305-3306.

[7] N Kumarasinghe，R Harpin，AW Stewart. Blood loss during suction termination of pregnancy with two different anaesthetic techniques［J］. Anaethesia and intensive care，1997，25（1）：48-50.

（收稿日期：2010-01-20）

（上接第 2226 页）

是最重要因素之一。本项目干预措施的重点在于通过对肥胖、超重儿童进行膳食结构调节和饮食控制基础上，加上适当强度的有氧运动，使摄入和消耗的能量平衡或呈负平衡，从而控制体重[4]。干预前后发现，肥胖者都喜欢上体育课和参加锻炼，但因运动空间与设施有限，限制了儿童有足够的运动量，加之学习负担较重，锻炼的时间不足。因此，学校和家长应重视这一问题，合理安排学生的学习和作息时间，使学生有一定的运动时间，达到控制体重的目的。

生活行为方式的干预是防治肥胖的基础。肥胖与个人的行为密不可分，但个人行为的改变离不开所在学校及家庭的影响，应重视对肥胖儿童人群的管理，把健康知识教育与运动处方的制定列入学校卫生工作之中。本次通过对 208 名超重与肥胖儿童的研究结果显示：通过干预，超重与肥胖儿童在体重、腰围、臀围、BMI 发生了很大变化，这些指标与干预前及对照组比较差异具有统计学意义（$P < 0.01$）。说明干预后超重与肥胖儿童对肥胖认识、控制体重的意识明显增强。

总之，综合干预措施对改善儿童超重与肥胖是安全有效的。但儿童青少年肥胖预防和干预是一项长期的系统工程，需要政府、社会、学校、家长的共同努力。只有共同付诸于行动，探索出合适的、长期的、持久的综合干预策略，才能有效的解决儿童青少年肥胖问题[5]。

参考文献：

[1] 石建辉，刘秀荣，田向阳，等. 北京市小学生肥胖干预效果分析［J］. 中国健康教育，2004，20（9）：782-785.

[2] 中国学生体质与健康研究组. 2000 年中国学生体质与健康调研报告［R］. 北京：高等教育出版社，2002. 142-159.

[3] 傅兰英，姬英涛，姬成茂，等. 运动处方对女大学生减肥及健康状况影响研究［J］. 中国学校卫生，2004，25（5）：539-540.

[4] 薛惠娟，戚萍芳. 托幼机构肥胖儿童干预措施的探讨［J］. 中国妇幼保健，2005，20（24）：3201-3202.

[5] 姚兴家. 儿童青少年肥胖判定及干预策略［J］. 中国学校卫生，2006，27（3）：185-188.

（收稿日期：2009-02-19）

?1994-2015 China Academic Journal Electronic Publishing House. All rights reserved. http://www.cnki.net
