# Supplementary material for: Systematic review and meta-analysis of school-based obesity interventions in mainland China
Source: PLoS One. 2017 Sep 14;12(9):e0184704. doi: 10.1371/journal.pone.0184704 (PMC5598996; doi:10.1371/journal.pone.0184704)
Supplement: S1 Dataset — (ZIP) [file pone.0184704.s007.zip › S1_dataset/76库/77.pdf]

缺氧亦可导致一定程度的新生儿脑功能受损，因其远期预后有待观察探讨，需引起儿科临床及保健工作者的重视。因此，对围产期急、慢性缺氧的新生儿早期积极观察、治疗的同时，均应进行早期干预，康复训练，减少神经系统发育合并症，促进儿童神经精神健康发育。

本研究通过对出生时轻、重度窒息及母亲患重度PIH等新生儿出生后24 h内检测血清尿酸水平，结合其血尿素氮、肌酐水平及EEG检查结果，发现围产期急慢性缺氧新生儿血清尿酸水平均显著升高，且与缺氧严重程度呈正相关。血清尿酸水平在一定程度上可反映缺氧后脑、肾功能损伤程度。在血清尿酸水平与远期预后关系方面尚需进一步探讨，但对于具有严重围产期缺氧并血尿酸水平较高的新生儿应积极跟踪随访观察。

4　参考文献

1　Becker BF. Towards the physiological function of uric acid. *Free Radic Biol Med* 1993; 14: 615-31
2　朱明德. 现代临床生物化学[M]. 上海: 上海医科大学出版社, 1996: 227
3　Baldree LA, Stapleton FB. Uric acid metabolism in children. *Pediatr Clin North Am* 1990; 37(2): 391-400
4　Ruilope LM, Garcia-puig J. Hyperuricemia and renal function. *Curr Hypertens Rep* 2001; 3(3): 197-202
5　陈虹, 王倩, 黄瑞兰. 新生儿缺氧缺血性脑病55例的脑电图监测与预后评估[J]. 中国临床康复, 2003, 7(1): 101

中国临床康复　*第9卷第11期*　2005-03-21出版
Chinese Journal of Clinical Rehabilitation, March 21 2005 Vol. 9 No. 11

·基础研究·

行为矫正对儿童肥胖症的控制效果

张秀莲

张秀莲, 商丘市第一人民医院儿科, 河南省商丘市　476100
张秀莲, 女, 1953年生, 河南省商丘市人, 汉族, 1976年河南医学院毕业, 学士, 副主任医师, 主要从事儿科疾病的诊断与治疗。
zhangxiulian@126.com
电话: +86-370-3255210

中图分类号: R725.8　文献标识码: A　文章编号: 1671-5926(2005)11-0166-02
收稿日期: 2005-01-07　修回日期: 2005-01-27　(05/NZY/YL)

Effect of behavior modification in controlling children's obesity

Zhang Xiu-lian
Zhang Xiu-lian, Department of Pediatrics, First People's Hospital of Shangqiu City, Shangqiu 476100, Henan Province, China
Correspondence to: Zhang Xiu-lian, Associate chief physician, Department of Pediatrics, First People's Hospital of Shangqiu City, Shangqiu 476100, Henan Province, China　zhangxiulian@126.com
Received: 2005-01-07　**Accepted**: 2005-01-27

Abstract

AIM: The interactions of heredity, behavior and environment can influence obesity in children. This paper aims to investigate the effect of behavior modification with the participation of medical service, school, parents and students in controlling the incidence rate of simple obesity in children.
METHODS: Pupils of Grade 6 in an elementary school were taken as the subjects during March 2002 and March 2003. Fifty-six subjects, who were in accordance with the diagnostic criteria of children's obesity screened by physical examination, were randomized into observation group ($n=29$) and control group ($n=27$). Subjects in the observation group received behavior modifications in diet, exercise and psychological cognition, while activities in the control group were as usual. Children in both groups were involved in this study with the permission of their parents.
RESULTS: After follow-up for one semester, the incidence rate of moderate and severe obesity in the observation group was significantly reduced from 79% to 41% ($\chi^2=4.1$, $P<0.01$), while that in the control group was increased from 85% to 96%. There were highly significant differences in the incidence rate of moderate and severe obesity between the two groups in the two detections ($\chi^2=17.81$, $P<0.01$).
CONCLUSION: The behavior modification in cooperation of medical service, school, parents and students can effectively reduce the incidence rate of obesity in children.

Zhang XL. Effect of behavior modification in controlling children's obesity. *Zhongguo Linchuang Kangfu* 2005; 9(11): 166-7(China)
张秀莲. 行为矫正对儿童肥胖症的控制效果[J]. 中国临床康复, 2005, 9(11): 166-7　[www.zglckf.com]

摘要

目的: 遗传、行为及环境等因素相互作用均可对儿童肥胖构成影响，本组旨在探讨由医疗机构、学校、家长、学生共同参与的行为矫正方法对儿童单纯肥胖症发生率的控制效果。
方法: 2002-03/2003-03以商丘市某小学6年级学生为研究对象，经体检筛选符合儿童肥胖诊断标准56例，随机将其分为观察组29例，对照组27例。观察组进行饮食、运动、心理认知等行为矫正。对照组与观察组同期体检外一切活动如常。
结果: 随访1个学期，观察组中、重度肥胖率从79%降至41%，两组患儿及家长均知情同意，差异有显著性意义($\chi^2=4.1$, $P<0.01$)；而对照组中、重度肥胖率从85%上升为96%。两组两次检测间中、重度肥胖率比较差异有非常显著性意义($\chi^2=17.81$, $P<0.01$)。
结论: 医疗机构、学校、家长、学生四位一体的行为矫正方法可有效降低儿童肥胖发生率。

主题词: 儿童; 肥胖症; 行为疗法

0　引言

儿童单纯肥胖症是一种营养障碍，体内常常伴有铁、钙等微量元素的不足，易引起缺铁性贫血、软骨病等多种营养缺乏性疾患，同时对儿童心血管、呼吸功亦能产生慢性损伤[1-3]。2002-03/2003-03随机抽取符合儿童肥胖诊断标准56例进行分组对照研究，探索儿童单纯肥胖症的行为干预的效果。

1　对象和方法

设计: 以小学生为研究对象的同期对照研究。

单位: 一所市级医院的儿科。

对象: 商丘市某小学6年级512名学生，本项研究提出的纳入标准: ①性别不限，年龄在11～13岁；②患者及家长知情同意。③经体检筛选符合儿童肥胖诊断标准[1,2]，纳入56例，男41例，女15例。随机将其分为观察组29例(男21例，女8例)，对照组27例(男20例，女7例)。观察组进行饮食、运动、心理认知等行为矫正。对照组与观察组同期体检外一切活动如常。

设计、实施、评估者: 资料收集者、实施干预为作者本人。评估者由本院经过专业训练医生进行。采用盲法。

方法: 体检: 由本人及校医共同完成。按WHO推荐的学生身高与体质量标准，结合中国学生7～22岁《营养评价参考标准(身高标准体质量)》[2,4]进行评价。行为矫

?1994-2015 China Academic Journal Electronic Publishing House. All rights reserved.　http://www.cnki.net

正：①饮食认知调整：针对肥胖儿童喜欢偏食、甜食、肉食的饮食习惯及摄入过多，开展由医务人员、学生、学生家长参与的健康教育课堂，讲解内容为：分析肉、鱼蛋奶、蔬菜、水果的营养结构及构成比，摄食后人体的生化代谢过程及偏食、多食所带来的营养不均衡等。中重度肥胖者限制摄食量，并采取分阶段过渡的方式。注意引导食粗纤维食物。饮食合理成分为：碳水化合物（非精制、富含纤维素，以其燕麦和麸皮面包为最好）占总热量的 50%～60%，脂肪占 25%～30%，蛋白质占 10%～20%，多吃瓜果蔬菜。②运动理念的培养：让学生了解有氧运动的方式、运动时间的掌握、运动速率、运动时的心率控制等。让学生明白有效性和持续性的运动对减肥效果的影响，同时，请老师、家长参与督导。运动强度一般为最大氧耗量的 50%（约为最大心率的 60%～65%）[1]，每周 3～5 次，时间为 1～2 h。

主要观察指标：行为矫正前后肥胖发病率及超重发生率。

统计学分析：所有数据由第一作者采用 SPSS 10.0 软件处理，计数资料的对比分析采用 χ^2 检验。

2 结果

2.1 描述性统计　参与者数量分析：参与体检 512 例，纳入 56 例，最终进入结果分析 56 例，观察组 29 例，对照组 27 例。

2.2 统计推断

2.2.1 儿童肥胖观察指标　执行儿童肥胖诊断标准[1]。轻度肥胖：体质量超过身高标准体质量的 20%～29%；中度肥胖：体重超过身高标准体质量的 30%～49%；重度肥胖：体质量超过身高标准体质量的 50% 以上。

2.2.2 行为矫正前后肥胖发病率及超重发生率情况　行为矫正、随访时间为 1 个学期。行为矫正后观察组中、重度肥胖率从 79% 降至 41%，差异有显著性意义，（$\chi^2=4.10$，$P<0.05$）而对照组中、重度肥胖率从 85% 上升为 96%。两组两次检测间中、重度肥胖率比较呈差异有非常显著性意义（$\chi^2=17.81$，$P<0.01$）。见表 1。

表 1　两组儿童干预前后肥胖及超重状况比较　（n/%）

项目	观察组（n=29）		对照组（n=27）	
	干预前	干预后	第一次测量	第二次测量
重度肥胖率	3/10	0/0	4/15	5/18
中度肥胖率	20/69	12/41	19/70	21/81
轻度肥胖率	6/21	11/38	4/15	1/4
正常	0/0	6/21	0/0	0/0

3 讨论

儿童单纯肥胖症多由遗传、行为及环境等多因素相互作用所致。有关研究资料[5－7]表明，儿童肥胖易导致多种血管疾患。儿童肥胖者易诱发高血压及糖尿病的发生，易引起运动系统疾患。对发育期的儿童来讲，肥胖导致过度增加的体质量，对骨骼和关节等运动系统，特别是对脊椎和下肢是一种额外的负担[2]。骨骼、关节等组织长期支撑过重的体质量，易发生关节炎、肌肉劳损或脊神经根压迫，严重影响肢体活动，影响儿童的生长发育。因此，预防儿童期肥胖是当前的一个很重要的课题。本研究重点在于培养孩子良好的饮食认知，开展由医务人员、学生、学生家长参与的健康教育课堂，针对肥胖儿童喜欢偏食、甜食、肉食的饮食习惯及摄入过多，讲解分析肉、鱼蛋奶、蔬菜、水果的营养结构及构成比，摄食后人体的生化代谢过程及偏食、多食所带来的营养不均衡等。让学生明白预防肥胖的重点是在满足生长发育的需要的前提下维持能量摄入和输出的"平衡"。将能量摄入控制在合理水平，通过额外的体力活动增加能量的消耗，既在控制摄能的同时，增加耗能。

培养儿童运动理念，让学生多参加户外运动。体育运动不仅可消耗多余的热能，还可促进肥胖儿童心、肺及运动系统功能发育。父母亲患有肥胖，其子女更要注意锻炼和合理饮食，以减轻遗传因素的影响。体育锻炼具有调节体重作用，它可以使主要产热营养脂肪和糖类消耗增加，有助于减少皮下脂肪的过多蓄积，使儿童少年得以协调匀称的发育。

结论：行为矫正对儿童单纯肥胖症的治疗及预防是可行的，可有效降低儿童肥胖发生率和肥胖度。

4 参考文献

1　卫生部卫生监督司．全国学生常见病综合防治方案技术规范[S]. 1993
2　孟昭恒，李加梅．儿童少年肥胖评价标准的探讨[J]．营养学报，1987，9(3)：248－5
3　王德芬，主编．现代儿科内分泌学－基础与临床[M]．上海：科学技术文献出版社，2001：4，400－13
4　陈学存．中国儿童营养研究的进展[J]．中华预防医学杂志，1999，33(3)：134
5　蒋竞雄，夏秀兰，吴光施，等．学龄儿童单纯肥胖症的群体干预研究[J]．中国儿童保健杂志，2002，10(6)：364－7
6　彭惠，杨书荣，李静，等．肥胖儿童血脂水平与个性特点分析[J]．中国临床康复，2004，8(36)：8275－7
7　朱永光．徐州市儿童肥胖的相关因素分析[J]．中国临床康复，2004，8(36)：8282－3

2005 年国家级继续医学教育项目“疾病诊治临床思维”学习班招生

如何才能作出正确的诊断呢？误诊和漏诊的原因是什么？作出诊断以后还应当怎么评估病情？合理的治疗决策是怎么产生的？怎么监测疗效和不良反应？治疗无效的原因是什么？如何选择实验室检查和辅助检查的项目？如何分析和应用检查的结果和报告？这些都是临床医师在工作中每天都会遇到的问题？也就是临床思维、临床判断和临床决策的问题。在长期开展临床思维研究的基础上，邀请我院内科、外科、诊断学、影像学、药剂科和检验科的有关专家作讲座，介绍临床思维研究和应用的经验及体会。并组织多种形式临床思维训练，如临床思维讨论会、临床问题辩论和提供系列病例当堂问答等。

时间：2005 年 6 月 3 日报到。

学习时间：2005 年 6 月 4 日～6 月 8 日报到地点：上海市中山医院。

学费：700 元/人，包括讲义费（食宿费、交通费自理）。

学分：国家级 I 类 10 学分（编号：2005－15－01 034）

报名方法：

电话：+86-21-64041990-2218 郑玉英、李哉萍、白浩鸣

传真：+86-21-64041990－2524

Email: jxjy@ zshospital. net

上海市医学院路 136 号中山医院继续教育科

邮编：200032 报名于 5 月 25 日前，收到报名单后发正式报名通知报到。

?1994-2015 China Academic Journal Electronic Publishing House. All rights reserved.　http://www.cnki.net
