# Supplementary material for: Systematic review and meta-analysis of school-based obesity interventions in mainland China
Source: PLoS One. 2017 Sep 14;12(9):e0184704. doi: 10.1371/journal.pone.0184704 (PMC5598996; doi:10.1371/journal.pone.0184704)
Supplement: S1 Dataset — (ZIP) [file pone.0184704.s007.zip › S1_dataset/76库/78.pdf]

【政策、管理与服务】

上海市杨浦区小学生营养干预模式研究

韩雪[1]，凌培敏[2]，陈远芬[1]，傅华[2]，李洋[2]

摘要：[目的]探讨适合杨浦区区情的学生营养餐干预模式。[方法]采用分层随机抽样方法，在杨浦区按照东西南北中每一块抽取2所学校，共抽取10所公立小学校，再随机抽取5所学校作为干预组，5所学校作为对照组，对5所干预学校运用健康促进手段进行干预，三年后进行干预后评价。[结果]过三年的营养综合干预，学生及家长的合理营养知信行水平提高，学生的体质情况有所改善，但肥胖率与超重率还有所上升，贫血率显著下降，营养午餐的三大营养素的摄入达标。[结论]进一步深化以健康促进为主要手段的学生营养餐综合干预模式。

关键词：小学生；营养干预；模式

Study on the Comprehensive Intervention Method of Nutritious Diet Which Fits for Students in Yangpu District, Shanghai *HAN Xue, LIN Pei-min, CHEN Yuan-fen, FU Hua, LI Yang* (*1.Yang-pu District Center for Diseases Prevention and Control of Shanghai, Shanghai* 201100, *China*; *2.School of Public Health, Fudan University, Shanghai* 200032, *China*)

Key Words: student; intervention; method of nutritions

慢性非传染性疾病的死亡率增长成为导致人群死亡率增长最快的原因，此类疾病已成为疾病发生和死亡的首要原因。造成这种趋势的主要原因是一些不健康的生活方式，包括对健康饮食认识不足及不良的饮食习惯。

虽然慢性疾病的临床表现出现较晚，但有研究表明主要行为危险因素在儿童时代就已形成。这些危险因素和一定的生活方式相关联，例如吸烟、长时间静坐、饱和脂肪的高消耗、以及普遍的食物过度消费，这些行为方式通常在儿童和青少年时期学会，而到成人阶段被养成。流行病学研究提示了心脑血管疾病危险因素在儿童和成人之间的高度相关性[1]。国内外经验表明，健康促进手段是改变持续增长的慢性疾病的发病率和死亡率的有效手段[2]。

为了改变这种情况，自2003年起，一个为期三年的综合性健康促进项目开始在杨浦区实施。干预项目的整体目标是促进儿童时期的健康饮食、生活习惯，并最终达到在成人时期的慢性病危险因素最小化的目的。

通过三年的综合干预，父母和孩子的健康意识增加，学生的饮食习惯有所改善，体质指数提高，学生营养午餐逐步合理，现将3年综合干预模式及干预初步结果报告如下。

1 研究对象和方法

1.1 研究对象

在杨浦区内按照东西南北中每一块所抽取2所学校，共十所学校中，再随机抽取5所学校作为干预组，5所学校作为对照组，对干预组进行综合干预，再对饮食摄入等变量进行3年干预后评价。对教育局下属的配餐中心供应的营养午餐进行营养评价。

1.2 调查方法

1.2.1 学生营养认知情况的调查

在5所干预学校和5所对照学校中，每所学校简单随机抽取1~4年级共280名学生（每个年级抽取70名学生），抽取的学生作为调查对象。在3年干预期的前后，利用自制调查表来评估学生的健康知识水平。调查表内容集中于饮食习惯、食品。调查表在研究小组成员面前完成。调查表重复实验的可靠性是通过在2周间隔时间内二次抽取的35名一年级和35名四年级学生样本中发放调查表来评估的。通过比对实验，第一次评估和重复评估之间无显著性差异（$P=0.073$，$P=0.23$）。

1.2.2 学生家长的营养认知情况

上述被抽中的学生家长作为调查对象，在3年干预期的前后，利用自制调查表来评估学生家长的健康知识水平。调查表内容集中于饮食习惯、食品。调查表在研究小组成员面前完成。调查问卷共设计10个有关营养、健康知识的问题，以答对6个题目作为认知合格标准。

1.2.3 配餐中心营养午餐的营养评估

在3年干预期的前后，由营养师随机抽取一个月的菜谱，利用营养分析软件对膳食营养摄入情况进行评价。以“中国居民膳食营养素参考摄入量”作为营养素评价标准。

1.3 数据收集方法

数据收集发生在2003年4~6月和2006年4~6月，由区疾控中心项目负责人对调查人员进行统一培训。

1.4 质量控制

由课题主持者对区疾病预防控制中心调查员进行统一培训；资料收集后，随机抽取5%的调查对象资料进行电话回访；

作者简介：韩雪（1974-），女，主管医师，硕士，主要从事疾病预防工作

作者单位：1.上海市杨浦区疾病预防控制中心，上海 201100；2.复旦大学公共卫生学院，上海 200032

?1994-2015 China Academic Journal Electronic Publishing House. All rights reserved.　http://www.cnki.net

数据录入采用双输入，对两次输入不一致的数据对照原始数据进行核查；通过计算机逻辑检错进行数据库资料整理，进一步更正录入错误和核查原始资料。

1.5 干预方法

1.5.1 应用 PRECEDE-PROCEED 模式[3]制订干预策略

PRECEDE-PROCEED模式提供了一个应用理论的组织框架，通过这个组织框架找出最合适的学生营养餐干预策略并指导实施。

通过社区诊断，结果如下：①杨浦区小学生对早餐的重要性的认知程度较高，也都养成了吃早餐的好习惯，但早餐营养保证情况不理想；②杨浦区小学生总体对膳食金字塔知识很缺乏，但具有对营养知识的兴趣，普遍具有挑食偏食习惯；③学生家长们的营养知识还很缺乏，对孩子们养成良好的饮食习惯还不利；④杨浦区小学生的体质出现“小胖墩”与“豆芽菜”两极分化的现象，一边是肥胖、超重率较高，一边是营养不良率较高，且贫血率较高；⑤杨浦区学生家庭的膳食结构正向高能量、高脂肪、高糖、低纤维“三高一低”的方向不断发展。⑥配餐中心（教育局三产下属，为试点学校配售午餐生菜）无营养师，缺乏对学生营养午餐的监督监测机制；无明确对各有关人员进行相关知识培训的规定；营养师资缺乏等等。

进一步的分析如图1显示，对影响这些行为和环境因素的倾向、促成、强化因素进行分析，因此我们认为应优先考虑的关键问题是：午餐的营养保证及各种人群相关知识和技能的提高。

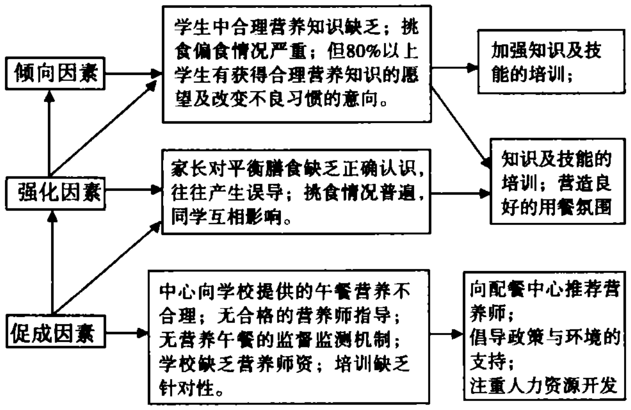

图1 应用PRECEDE-PROCEED模式设计学生营养干预项目示意图

1.5.2 应用生态学模型进行干预

（1）政策支持：区教育局出台《关于加强学生营养午餐工作管理的实施意见》发放至全区各学校；干预组学校制定营养午餐管理制度；配餐中心制订配餐中心管理制度（包括营养师的配备要求及职责，内部的监督机制等）及制定学生营养午餐基本要求。（2）环境支持：教育局、学校共同出资改建了干预组食堂；配餐公司配备了高素质的营养师进行监督指导；编印学生营养小报，免费发给学生、教师，并在小报上开辟“朱医生信箱”（朱医生为配餐中心营养师）；营养师发挥了对学校、家庭、社区营养指导及沟通作用。（3）人力资源开发：由区CDC对学校卫生老师、配餐中心工作人员等有关人员进行营养师资培训，然后由学校、配餐中心进行层层培训。

2 结果

2.1 营养综合干预后学生的营养认知情况评估

2.1.1 营养综合干预三年前后学生的一般情况

调查的十所小学校均位于区内中心地段，属公立学校，每所学校设立一到五年级。调查随机抽取十所小学校一至四年级的2 800名学生进行问卷调查，干预组1 400名学生，干预前实际调查到1 333名学生，应答率95.2%；干预后实际调查到1 328名学生，应答率94.9%。对照组1 400名，干预前实际调查到1 345名学生，应答率96.1%，干预后实际调查到1 342名学生，应答率95.9%。

2.1.2 干预前后调查学生的年龄性别构成

干预组学生中干预前男生708人，女生625人，共计1 333人；干预后男生704人，女生624人，共计1 328人；对照组学生中干预前男生706人，女生639人，共计1345人。干预后男生707人，女生635人，共计1 342人。调查对象中干预组对照组在干预前后性别年龄比相似。（因篇幅原因，具体表格不再列出）

2.1.3 营养综合干预三年前后干预组与对照组学生的营养认知情况比较

干预前干预组与对照组的营养认知情况无差异（$P>0.05$）。在综合干预三年后，干预组与对照组对营养知识的认知情况差异皆有显著的统计学意义（$P<0.05$）（见表1）。

表1 营养综合干预三年前后干预组与对照组的营养认知情况比较

认知情况	干预组人（%）	对照组人（%）	检验统计量(χ^2)	P值
干预前认为早餐很重要	1 296(97.22)	1 321(98.21)	2.95	>0.05
干预后认为早餐很重要	1 327(99.92)	1 310(97.62)	20.56	0.000***
干预前有吃早餐的习惯	1285 (96.40)	1 297(96.43)	0.00	>0.05
干预后有吃早餐的习惯	1 324(99.69)	1 286(95.82)	37.25	0.000***
干预前能保证早餐营养	645(48.39)	622(46.25)	1.23	>0.05
干预后能保证早餐营养	1 089(82.00)	637(47.47)	344.34	0.000***
干预前自诉对营养知识感兴趣	1173(87.90)	1 113(82.75)	18.82	0.000***
干预后自诉对营养知识感兴趣	1 317(99.17)	1 098(81.82)	222.62	0.000***
干预前了解膳食金字塔的内容	254(19.05)	242(17.99)	0.50	>0.05
干预后了解膳食金字塔的内容	1 189(89.53)	271(20.19)	1 286.8	0.000**
干预前自诉无挑食、偏食的习惯	559(41.94)	569(42.30)	0.04	>0.05
干预后自诉无挑食、偏食的习惯	897(67.55)	524(39.05)	215.70	0.000**

[注]**：$P<0.001$；*：$P<0.01$（以下各表含义相同）

表2 干预组在干预三年前后的营养知识认知情况比较

认知	干预前人（%）	干预后人（%）	检验统计量(χ^2)	P值
认为早餐很重要	1 296(97.22)	1 327(99.92)	34.45	0.00**
有吃早餐的习惯	1 285(96.40)	1 324(99.69)	37.79	0.00**
能保证早餐营养	645(48.39)	1 089(82.00)	331.03	0.00**
自诉对营养知识感兴趣	1 173(87.90)	1 317(99.17)	138.10	0.00**
了解膳食金字塔的内容	254(19.05)	1 189(89.53)	1 330.79	0.00**
自诉无挑食、偏食的习惯	559(41.94)	897(67.55)	176.02	0.00**

[注]*：$P=0.00$，**：$P<0.05$

?1994-2015 China Academic Journal Electronic Publishing House. All rights reserved. http://www.cnki.net

2.1.3.1 干预组在干预三年前后的营养知识认知情况比较

干预组在干预后的营养知识认知情况与干预前相比皆有统计学差异(见表2)。

2.1.3.2 对照组在干预三年前后的营养知识认知情况比较

对照组在三年前后的营养认知情况没有统计学差异(因篇幅原因,具体表格不再列出)。

2.2 营养综合干预三年前后干预组及对照组学生体质指数情况比较

2.2.1 营养综合干预三年前后干预组与对照组的体质指数情况比较

干预前干预组与对照组的体质指数及贫血率差异无统计学意义,在综合干预三年后,干预组较对照组贫血人数减少,有显著的统计学意义,肥胖人数与超重人数的改变无显著的统计意义(见表3)。

表3 营养综合干预三年前后干预组与对照组的体质指数情况比较

各类人群		干预组人(%)	对照组人(%)	检验统计量(χ^2)	P值
肥胖	干预前	187(14.03)	175(13.01)	0.59	>0.05
	干预后	209(15.74)	212(15.80)	0.00	>0.05
超重	干预前	362(27.13)	379(28.18)	2.29	>0.05
	干预后	422(31.78)	421(31.37)	0.10	>0.05
贫血	干预前	152(11.40)	164(12.19)	0.40	>0.05
	干预后	77(5.80)	159(11.85)	30.43	0.00**

2.2.2 干预组在干预三年前后学生体质情况比较

干预组在干预后的学生体质情况与干预前相比,贫血人数下降,有统计学差异。超重肥胖人数的改变没有统计学意义(见表4)。

表4 干预组在干预三年前后学生体质情况比较

各类人群	干预前人(%)	干预后人(%)	检验统计量(χ^2)	P值
肥胖	187(14.03)	209(15.74)	1.53	>0.05
超重	362(27.13)	422(31.78)	3.45	>0.05
贫血	152(11.40)	77(5.80)	26.56	0.00**

2.2.3 对照组在三年前后学生体质指数情况比较

对照组在三年后,学生的超重人数、肥胖人数、贫血人数与干预前相比皆无统计学差异(因篇幅原因,具体表格不再列出)。

2.3 营养综合干预前后学生家长营养认知情况

2.3.1 营养综合干预前后学生家长一般情况

本次共发放调查问卷2 800份,干预组与对照组各1 400份,干预组回收有效问卷1368份,回收率97.71%;对照组回收有效问卷1 324份,回收率94.57%。干预组与对照组人群在年龄、性别、职业构成上无明显差异(因篇幅原因,具体表格不再列出)。

2.3.2 营养综合干预前后学生家长营养认知情况评价

根据卷面评分情况,干预前干预人群中有20.70%的家长达到了合理营养认知合格标准,干预后干预人群中91.47%的家长达到了合理营养认知合格标准,干预前干预人群与对照人群营养认知水平没有差异,干预后干预人群较对照人群有明显改变。干预后较干预前的合理营养认知水平有明显增加。干预前对照人群中有16.99%的家长达到了合理营养认知合格标准,干预后对照人群中有23.48%的家长达到了合理营养认知合格标准,对照人群的营养认知水平也有增加。(见表5)

表5 杨浦区家长合理营养认知合格标准情况

合格标准情况	干预人群	对照人群	检验统计量(χ^2)	P值
干预前合格	283(20.70)	255(18.73)	0.87	>0.05
干预后合格	1 245(91.47)	312(23.48)	1 292.44	0.000***
检验统计量(χ^2)	1 372.5	5.41	-	-
P值	0.000***	0.0*	-	-

2.3.3 营养午餐的膳食摄入评价

经过三年营养干预,配膳中心学生营养午餐优质蛋白质的比例从2003年的35.1%上升到2006年的52.7%。能量的营养素来源中,来自碳水化合物的能量下降,由2003年的63.0%下降到2006年的56.3%,符合55%~65%的合理范围[4]。来自蛋白质和脂肪的能量上升,来自蛋白质的能量由2003年的15.3%上升到2006年的18.1%,来自脂肪的能量由2003年的21.7%上升到2006年的26.6%,符合中国居民膳食营养素参考摄入量和世界卫生组织推荐的30%的上限之内的标准[5]。

3 讨论

经过三年的营养综合干预,学生及家长的合理营养知信行水平提高,学生的体质情况有所改善,但肥胖率与超重率还有所上升,贫血率显著下降,说明营养干预对贫血率的干预还是有成效的,但对超重和肥胖成效不明显,说明营养干预还需进一步深化,如何进一步加强对超重和肥胖儿童的营养干预将是我们进一步探讨的问题。

3.1 此项目干预的主要特点

3.1.1 加强培训,并通过教师来对学生进行教育

在教育局及卫生局的支持下,对5个干预组学校的教师举行研讨班。举办研讨班的目的是使教师熟悉项目对象和他们的职责。同时研讨班也重点强调了干预项目中将健康、营养、适应性等内容整合的重要性和意义。最后,在研讨班上将预备好的教学和课堂用材料提供给教师。研究组的成员定期观察干预学校的老师,以次来监督和评估传授项目内容的教师。

内容丰富的教学资料将干预学校之间可能的教学差异性降到最低。教师培训班在提供标准化的素材方面也起了很大作用,而课题小组也对教师及时提供帮助。课题小组对教学定期的监督,显示教学时间安排和材料使用方面与项目设计的完好一致性。

3.1.2 加强父母的参与意识

父母在改变小孩,尤其是幼儿饮食和运动习惯方面所扮演的重要作用已经得到广泛认同。课题组召开家长会,会上提供给父母由课题组设计的,能提供营养指导和体育运动知识的手册。并由营养师对父母们进行了与儿童饮食和运动习惯主题相关的知识讲座。此外,父母被鼓励改变自身的饮食习惯,也被

?1994-2015 China Academic Journal Electronic Publishing House. All rights reserved. http://www.cnki.net

建议鼓励子孙进行体育活动而不是惯于久坐。为了促进父母的参与，这些会议通常在每个学校一年举办一次。这些会议使得父母有机会就他们子女健康问题发表看法和提出疑虑。对照组父母不参加任何这种教育会议。

自从广泛推荐家庭在改变儿童饮食和运动习惯方面的扮演重要角色以来，父母的参与已经成为绝大多数学校健康干预项目的组成部分。学校教师要求每个家长必须参加会议，如果因事不能到的话，将对缺席家长进行补课。经过综合干预，项目实施3年以来父母健康知识水平的增长也表明了项目对父母产生影响。然而，目前研究中运用的父母培训班和学校干预都没有显示任何显著性的饮食方面的改变。这也许是因为改善饮食习惯干预难度较大。

3.1.3 干预组学生健康知识的显著性改变主要归因于课堂健康教育课

干预组学校健康教育课的时间总数超过了40 h。干预组的学校教师对项目的热情和自身的能力是学校学生健康知识改变的主要因素之一。

总之，相对于对照组来说，在干预组中观察到的显著性变化归因于项目中父母的高参与性，以及精心设计和不断扩大的健康促进课程。这种干预为小学营养教育项目提供了一个很好的模式，而且不需要相当的学校时间和新的资源。

3.2 杨浦区学生营养餐综合干预模式的形成

在综合干预过程中，我们采取了场所、部门、策略、措施的综合干预模式。干预组学校制定营养午餐管理制度；食堂硬件符合营养卫生要求；承担对学生、教师、家长、炊事员的营养知识教育。并编印《学生合理营养指南》下发至学生手中。配餐中心制订配餐中心管理制度（包括营养师的配备要求及职责，内部的监督机制等）；制定学生营养午餐基本要求；为教育局修订学生营养午餐实施细则提供参考意见；负责对学校食堂管理员、配餐中心职工、进行营养知识教育；负责进行学生对午餐满意度以及随意丢弃饭菜比例等的监测；负责编印学生营养小报，免费发给学生、教师，并在小报上开辟“朱医生信箱”（朱医生为配餐中心营养师）发挥对学校、家庭、社区营养指导及沟通作用。街道办事处通过“校弄结合” 配合学校做好“校门清”；通过画廊、黑板报、广播、资料入户及家庭主妇烹调培训等形式对居民进行营养教育；与学校配合通过“亲子教育”传播营养知识。社区卫生服务中心：对辖区内学校、配餐中心进行营养指导及监督监测；对街道办事处乃至居民进行营养指导。

在干预过程中，完善了一系列的监督措施：

①学校接受学生、教师、家长的监督：学校每周一在校门口公布一周午餐食谱，并每天展出当天午餐实物，供监督。不定期进行对营养午餐满意度测定。②配餐中心接受学校的监督：每天送货时，对照食谱进行质与量的验收；学校每天记录学生倒饭情况，并向配餐中心反馈；配餐中心每周一次，召集各学校食堂管理员听取意见及建议。每季一次听取校领导意见及建议。③教育局及区卫生局的监督：区教育局会同区卫生局不定期到学校随机抽取当天午餐，请营养师盲样分析。

经过三年的运转，干预组学生体质增强，午餐营养大大改善，在干预学校及配餐中心已形成惯性运转；作为可持续发展考虑，经验已由五所干预学校扩大至全区81个小学进行推广。

3.3 尚需进一步改进的地方

①有些学校教师在进行营养健康教育课时，讲解内容不够专业、精炼、贴切，需加强培训，以不断提高健康教育课的质量和效果。②学校食堂的硬件建设普遍不能满足实际需要，因此，某些合理营养的要求尚难达到。③因经费和成本核算所限，故在营养午餐的品种上显得有些单调，难以符合学生口味和营养需求，有关方面应适当协调，在不增加学生负担的前提下，酌情改善营养午餐的品种和质量。

参考文献：

[1]Yannis Manios, Evaluation of a Health and Nutrition Education Program in Primary School Children of Crete over a Three-Year Period[J]. Preventive Medicine 28, 149-159(1999).

[2]Perry C L, Luepker R V, Murray D M, et al. Parent involvement with children's health promotion: The Minnesota Home Team[J]. Am J Publ Health, 1988, 78:1150-1160.

[3]傅华，李枫.现代健康促进理论与实践.上海：复旦大学出版社.

[4]中国营养学会. 中国居民膳食营养素参考摄入量(Chinese DR Is)[M].北京：中国轻工业出版社，2000：102，127.

[5]世界卫生组织和粮农组织联合专家磋商会报告. 膳食、营养和慢性疾病预防[R]. 世界卫生组织技术报告丛书(916号)，2003：13-29.

?1994-2015 China Academic Journal Electronic Publishing House. All rights reserved. http://www.cnki.net
