# Supplementary material for: Systematic review and meta-analysis of school-based obesity interventions in mainland China
Source: PLoS One. 2017 Sep 14;12(9):e0184704. doi: 10.1371/journal.pone.0184704 (PMC5598996; doi:10.1371/journal.pone.0184704)
Supplement: S1 Dataset — (ZIP) [file pone.0184704.s007.zip › S1_dataset/76库/79.pdf]

【运动人体科学】

DOI:10.13293/j.cnki.tylt.002577

大课间体育活动干预对超重和肥胖儿童身体形态的影响研究

唐艳平[1] 郭旭霞[2]
(1.廊坊师范学院体育学院, 河北 廊坊 065000 2.广西大学体育学院, 广西 南宁 530004)

摘 要:对廊坊市第一实验小学三、四年级学生的 60 名超重肥胖儿童,分成对照组和实验组,每组各 30 人,对照组进行传统的大课间活动,实验组进行一个学期的运动干预,对两组的身体形态指标进行比较分析。结果:男生和女生腰臀比没有明显变化($P>0.05$),BMI 都有显著的下降($P<0.05$),男生的身高和体重有显著的增高($P<0.05$),女生体重有明显下降趋势($P<0.01$)。结论:科学合理的大课间运动干预能够降低超重、肥胖小学生的体重和 BMI 值;身体形态得到一定的改善。

关键词:大课间 超重 肥胖 小学生 运动干预 身体形态

中图分类号:G804 **文献标识码**:A **文章编号**:1004—5643(2015)11—0071—03

1 大课间体育活动概述

体育专家将“大课间”定义为是集中安排一段时间,在学校统一组织安排下,以促进学生身心健康发展为目的的全校性的集体课间活动[1]。大课间体育活动属于体育课程的下位概念,是活动类体育课程[2]。活动的主要目标是提高学生的健康素质,发展学生的健全人格。与传统的课间操相比,大课间活动时间长、互动范围大、内容丰富、组织形式多样、活动场地大、强度适宜、整体效益大、对于学生身心和谐发展有着重要的意义。现代的大课间体育活动将“快乐教育”、“养成教育”、“健康教育”、“艺术教育”等现代教育理念贯穿于活动之中[3],使之更加喜欢参与其中,兴趣浓厚,在增强体质的同时,使学生形成积极向上的人生观,健康的心态。因此,大课间体育活动可以根据学校具体情况,安排特色运动内容,在班主任参与管理下有组织、有计划地开展。一般大课间体育活动项目分为“一个核心,两个基本点”、“一个核心”即以实施素质教育为核心,促进学生综合素质的提高与发展,融育智、育德、育美、娱乐于健体之中。“两个基本点”指:一是发挥学生的主体作用,激发学生体育锻炼的兴趣,培养少年儿童良好的体育锻炼习惯,为健康身体打下牢固的基础;二是在使学生接受更多的运动项目后,对其喜爱和适合的项目进行进一步的教学和训练. 从而遵循小学生学习范围广泛和运动技能形成的过程性这两个要点[4,5]。

2 实验对象与方法

2.1 实验对象及分组

根据国家体质健康标准,测试廊坊市第一实验小学三、四年级 300 名学生身高、体重,计算出 BMI 指数,根据 2003 年确定的“中国学龄儿童青少年超重、肥胖 BMI 筛查分类标准,筛选出超重和肥胖学生,使其了解并自愿参加本次实验。然后由调查者复测,排除因疾病等引起的肥胖者,将满足超重及肥胖标准者纳入研究,共筛选出 60 人,并分成对照组和实验组,每组各 30 人,其中男生 18 人,女生 12 人。学生年龄为 8-9 岁。(年龄的计算方法:测定时已过生日者年龄(周岁)= 测定年 - 出生年)。

表 1 中国学龄儿童青少年超重、肥胖 BMI 筛查分类标准

年龄(岁)	男超重	男肥胖	女超重	女肥胖
7~	17.4	19.2	17.2	18.9
8~	18.1	20.3	18.1	19.9
9~	18.9	21.4	19.0	21.0
10~	19.6	22.5	20.0	22.1
11~	20.2	23.6	21.1	23.3
12~	21.0	24.7	21.9	24.5

表 2 实验对象具体分组情况

组别	男	女	合计
对照超重组	9	6	15
对照肥胖组	9	6	15
实验超重组	9	6	15
实验肥胖组	9	6	15
合计	36	24	60

作者简介:1.唐艳平(1974~),女,副教授。研究方向:运动人体科学。

?1994-2016 China Academic Journal Electronic Publishing House. All rights reserved. http://www.cnki.net

表 3　实验对象分组后身高、体重及 BMI 情况

组别	性别	身高（cm）	体重（kg）	BMI(kg/m²)
对照超重组	男	144.31±5.54	39.33±4.47	18.92±7.53
	女	146.20±6.03	41.23±5.48	19.35±7.41
对照肥胖组	男	148.47±5.59	53.34±12.14	24.25±1.50
	女	146.39±4.32	54.20±12.86	25.43±2.34
实验超重组	男	145.34±8.04	40.80±12.14	19.43±6.24
	女	146.31±6.62	42.90±4.52	20.13±8.04
实验肥胖组	男	147.62±9.03	54.85±10.49	25.51±3.53
	女	147.09±7.65	51.02±14.45	23.43±1.53

从表 3 可知，对照组和实验组受试者在身高、体重、BMI、在运动干预之前均无显著性差异（P>0.05）。

2.2　实验方法

2.2.1 指标的选择

身体形态指标：身高、体重、体指数（BMI）、腰臀比。

身高、体重：采用金属立柱式身高坐高仪测定。受试者赤脚立正姿势，躯干自然挺直，头部正直，足跟、骶骨部及两肩胛间三点与立柱相接触，由同一人员进行测量。测量人员两眼与水平版呈水平位进行读数，测量数据精确到 0.1cm。

体指数（BMI）= 体重（kg）/ 身高（m2）

腰围：按照世界卫生组织推荐的方法测量。被测者站立，双脚分开 25-30cm，使体重均匀分布。腰围量取水平站立时脐上 0.5cm 处的水平周长。

臀围：臀围量取臀大肌最突起处的水平周长。

腰臀比 = 腰围（cm）/ 臀围（cm）

2.2.2 实验设计

对廊坊市第一实验小学三、四年级学生进行身高体重等三方面指标的测定，算出 BMI 值分出实验组和对照组：对照组和全校学生一起做传统课间活动，包括眼保健操（5 分钟），第九套广播体操（16 分钟）和学校自编操（5 分钟）。实验组进行 19 周的大课间运动干预，每周运动 5 次，每次 26 分钟，具体见表 4，实验结束后，对对照组和实验组的 60 位学生进行指标测试。

表 4　实验方案

组别	星期一	星期二	星期三	星期四	星期五
对照组	眼保健操＋广播体操＋学校自编操	眼保健操＋广播体操＋学校自编操	眼保健操＋广播体操＋学校自编操	眼保健操＋广播体操＋学校自编操	眼保健操＋广播体操＋学校自编操
实验组	热身 +1 分钟跳绳 *3+ 捕鱼	热身 +1 分钟仰卧起坐 *3+"贴膏药"	热身＋俯卧撑 *3+ 喊数抱团成组	热身 +1 分钟象限跳 *3+ 老鹰捉小鸡	热身 +1 分钟后振躯干 *3+ 丛林大冒险

3　结果与分析

3.1　19 周后身体形态指标的变化

表 5　19 周后身体形态的变化

组别	性别	运动干预	身高(cm)	体重(kg)	BMI(kg/m²)	腰臀比(%)
对照组	男	干预前	146.39±5.67	46.33±8.99	21.50±4.46	0.91±0.053
		干预后	147.09±8.09	48.01±10.05	21.28±0.78	0.93±0.005
	女	干预前	146.34±5.75	47.71±7.25	22.56±6.78	0.92±0.104
		干预后	147.06±9.05	49.21±9.86	22.93±9.54	0.93±0.408
实验组	男	干预前	147.08±8.08	46.32±11.08	22.47±4.56	0.89±0.054
		干预后	148.99±9.03■	44.71±7.25■	20.94±9.42■	0.88±0.092
	女	干预前	146.72±11.02	46.96±9.45	21.96±0.38	0.90±0.015
		干预后	147.06±15.04	43.85±12.09■■	19.84±6.36■	0.89±0.085

注：干预前与干预后比较：■示对比显著（P<0.05），■■示非常显著（P<0.01）；

由表 5 可知，对照组干预前后男生和女生身高、体重、BMI 和腰臀比都没有明显变化（P＞0.05），体重反而还有增重的趋势，说明对照组进行传统的大课间活动，效果并不明显。实验组干预前后，男生的身高有显著的增高（P<0.05），男生和女生腰臀比没有明显变化（P＞0.05），男生体重有显著下降趋势（P<0.05），女生体重有明显下降趋势（P<0.01），BMI 都有显著的变化（P<0.05）。实验组的身高与体重平均增长速度与对照组相比无显著差异（P＞0.05），这表明本研究的干预活动对儿童青少年的正常生长发育速度无不良影响，也说明本次大课间活动内容的整体设计比较合理，出现了明显的效果。分析其原因可能是学生正处于生长发育的敏感期，刺激强度合理。

身高是判断青少年儿童发展的重要指标之一，而且能反应学生的发育状况。通过研究表明肥胖学生的生长速度较正常青少年儿童比较慢。在本次研究中，对照组超重肥胖男生的身高变化不明显，实验组的身高变化明显，这与大多数的研究结论是一致的，也就是说，经常参加体育锻炼对儿童少年的减脂和生长起到一定的促进作用，生长速度也较快。体重也是判断青少年儿童发展的重要指标，实验组的男生体重有明显的下降，女生体重有非常明显的下降，这主要是因为本次的运动干预中游戏属于有氧运动，可以更好地消耗脂肪，使之减少体脂含量，同等强度下，女生的体脂含量下降更明显。BMI 是判断一个胖瘦和评价一个人的营养状况的常用指标。对于生长发育期的儿童青少年，BMI 水平随着年龄的增长而增长，对肥胖儿童青少年的运动干预只要维持 BMI 水平不变，或者减少 BMI 增长的速度，便可以达到减肥目的。刘文等人对 49 名 12- 14 岁肥胖青少年儿童进行了运动干预之后男生 BMI 比干预前下降了 0. 3 和女生下降了 0. 5，而不运动组 BMI 则有所上升[6]。王筝对 8 岁至 12 岁肥胖儿童进行运动干预，运动干预后试验组 BMI 明显低于对照组[7]。通过各种资料调查显示，经过学校组织的长期运动干预的学生，尤其是进行中强度干预的学生，有效预防和延缓肥胖青少年肥胖的进一步发展，改善糖脂代谢，对体重的降低可以得到有效的控制。在本次对肥胖生运动干预也说明了这一点，在本实验中男生的 BMI 平均下降了 1.5，女生下降了 2.1，有效达到了减肥的目的。

腰臀比是人的腰围和臀围之比，是反映腹腔及腹部皮下深

?1994-2016 China Academic Journal Electronic Publishing House. All rights reserved.　http://www.cnki.net

层脂肪厚薄以及腹肌体积，也是反映腹部脂肪堆积程度的较好指标，同时也是早其中鉴别肥胖指标的一种有效方法。沈雪寒等对 23 名重度肥胖女性儿童青少年运动减肥前后身体形态指标进行研究，经过 1 周运动减肥后，腰臀比减小了 5. 61%；腰围 / 身高比下降了 13. 65%；臀围 / 身高比下降了 8. 53%，运动减肥可改善腰臀比 [8]。本研究干预后发现实验组的腰臀比虽有所下降，但效果并不明显，对照组的腰臀比有所上升，说明本次大课间干预对内脏脂肪有一定的消耗，但强度还不够大，导致效果不太明显。

4　结论

19 周的大课间体育活动干预，可使超重、肥胖儿童的体重得到有效的控制，BMI 明显下降，但腰臀比不明显，身体形态有一定的改善。

参考文献：

[1]葛敏娜．“大课间活动”——教育人性化的觉醒[J]．东方青年·教师，2013，5（92）．

[2]周凤施．对大课间体育活动的理论研究[J]．湖北体育科技，2007（6）：713-716．

[3]喻修文．“大课间体育活动”研究[J]．新课程研究 教师教育，2012（2）：43-45．

[4]许震磊．“阳光体育”背景下开展小学生大课间分项目活动之初探[J]．考试周刊，2013（91）：116-117．

[5]祁生仓．开展大课间体育活动丰富校园文化生活[J]．青海教育，2005（04）．

[6]刘 文．基于学校的有组织的运动干预对肥胖青少年 BMI 和糖、脂代谢的影响[J]．中国运动医学杂志，2008（3）：329-331．

[7]王 筝．运动对儿童肥胖的影响及其可能机制的研究[D]．浙江大学硕士学位论文，2013．

[8]沈雪寒，等．运动减肥对重度肥胖女儿童青少年腰围、臀围以及腰臀比的影响[J]．体育科研，2010（3）：96-98．

Effect of large class break activity intervention on the body shape of overweight and obese children

Tang Yanping[1]　Guo Xuxia[2]

(1.College of physical education, Lang Fang Teacher's College, Hebei Langfang 065000；2.P.E. School of Guangxi University,Xining Guangxi 530004)

Abstract：60 overweight and obese children in the First Experimental Primary School of Langfang city were divided into control group and experimental group, with 30 persons in each group. The control group was performed with traditional large class activities, and the experimental group was conducted a term of exercise intervention, and the body shape index of two groups were compared. Results: the waist hip ratio of boys and girls was not significantly changed ($P > 0.05$); both BMI had a significant decrease ($P<0.05$), the height and weight of male students were significantly increased ($P<0.05$), and the weight of female students was significantly decreased ($P<0.01$). Conclusion: scientific and reasonable exercise intervention can reduce the overweight and obese students' body weight and BMI value; the body shape can be improved.

Key words：large class break　overweight　obesity　primary school students　exercise intervention　body shape

---

（上接第 67 页）control of peripheral physiological systems during exercise in humans[J]．Br J Sports Med，2005（1）：52-62．

Speed Rhythm Feature Analysis of Men's Full Stage of 20km Race Walking

Sun Xiaoxue

(Shandong Normal University，Ji'nan Shandong 250014)

Abstract：Through using the grey correlation analysis method and on the basis of multiple game athletes relative speed variation characteristics, this paper sums up the winning rule of men's 20 km race walking pace. The results show that the men's entire 20 km race walking's winning pace of four stages are as follows: the first phase begins with a slow start; the second phase positive acceleration, for better positions; the third phase adjusts rhythm to keep good position; the fourth stage chooses appropriate spurt timing.

Key words：Men's 20km Race Walking　Division of stages　Speed rhythm

?1994-2016 China Academic Journal Electronic Publishing House. All rights reserved.　http://www.cnki.net
