# Supplementary material for: Systematic review and meta-analysis of school-based obesity interventions in mainland China
Source: PLoS One. 2017 Sep 14;12(9):e0184704. doi: 10.1371/journal.pone.0184704 (PMC5598996; doi:10.1371/journal.pone.0184704)
Supplement: S1 Dataset — (ZIP) [file pone.0184704.s007.zip › S1_dataset/76库/80.pdf]

厦门市某小学儿童肥胖影响因素分析

陈友兰[1]，杜玉开[2]，杨晓剑[1]，管纪惠[3]，余红[4]，徐月英[5]

【中图分类号】R193，R151.1 【文献标识码】A 【文章编号】1002-9982（2010）10-0756-05

【摘要】 目的 探索儿童肥胖的影响因素，评价学校对肥胖儿童进行群体性干预的效果。方法 选择条件基本相同的两所小学，1所为干预学校，1所为对照学校。干预学校开展“中国/WHO以肥胖控制为切入点发展健康促进学校”活动，对照学校不开展相关活动。2004年4月完成基线调查，然后对两所学校各820名学生进行持续4年的跟踪监测，并于每年的4月分别对两所学校全体学生、学生家长和全体教职工进行知信行问卷调查、身高测量和体重测量。结果 厦门市学龄期儿童单纯性肥胖的影响因素主要为营养过度和饮食行为偏差，采取针对性干预措施后，干预校学生的肥胖率和超重率显著下降，分别从原来的28.3%和17.4%下降到9.9%和15.0%，差异具有统计学意义（$\chi^2=150.322$，$P<0.01$）；知识、态度、行为的正确率显著提高，分别从原来的51.5%、82.7%和59.4%提高到95.3%、99.1%和97.2%，差异具有统计学意义，P值均小于0.01。结论 健康促进学校活动对肥胖儿童进行群体性干预的效果显著，为儿童肥胖干预提供了一个非常好的平台。

【关键词】 儿童；肥胖；影响因素；分析

Analysis on influencing factors of childhood obesity at one primary school in Xiamen City

CHEN You-lan, DU Yu-kai, YANG Xiao-jian, GUAN Ji-hui, YU Hong, XU Yue-ying. Xiamen Municipal Center for Disease Control and Prevention, Xiamen 361021, China

【Abstract】 **Objective** To explore the influencing factors of childhood obesity at one primary school in Xiamen City, and to evaluate the effectiveness of school-based group intervention for obese children. **Methods** Two primary schools with basically same conditions were selected, one as the intervention group, and another as the control group. In the intervention school, the " focus on obesity control to create health-promoting school" activities were carried out, while in the control school, the associated activities were not implemented. The baseline survey was completed in April 2004, and a four-year follow-up surveillance was conducted. In April of each following years, approximately 820 participants including students, teachers, parents, and administrative staff at each school were investigated with KPA questionnaire along with weight and height measurements. **Results** The leading influencing factors for childhood obesity in Xiamen City were over-nutrition and eating disorder. After the intervention, the obesity rates was changed from 28.3% to 9.9%, and the overweight rates from 17.4% to 15.0% ($\chi^2=150.322$, $P<0.01$). The rates of knowledge awareness, attitude holding and behavior forming was increased from 51.5%, 82.7%, and 59.4% to 95.3%, 99.1%, and 97.2%, respectively ($P<0.05$). **Conclusion** Health-promoting school is a good platform for the childhood obesity intervention, and the effectiveness of the school-based group intervention is remarkable.

【Key words】 Childhood; Obesity; Influencing factor; Analysis

国际肥胖特别工作组（TOTF）指出：“肥胖将会成为21世纪威胁人类健康和生活满意度的最大敌人”[1]。近年来，随着我国人民生活水平的提高，儿童肥胖的发生率呈日益增加的趋势，7~22岁城市学生超重、肥胖检出率男生由1995年的10.15%上升到2005年的24.64%，女生由8.03%上升到13.74%[2]，因此，从儿童期开始预防肥胖是十分必要，2004年4月厦门市疾病预防控制中心健康教育所通过开展中国与世界卫生组织合作的以肥胖控制为切入点发展健康促进学校项目，以厦门市外国语附属小学作为试点学校开展儿童肥胖干预活动，历时近5年，于2008年12月进行了末次调查，现将调查结果报告如下。

1 对象与方法

1.1 对象 厦门市外国语附属小学学生。

【作者单位】1 厦门市疾病预防控制中心健康教育所，福建 厦门 361021；
2 华中科技大学同济医学院，福建 厦门 361003；
3 福建省疾病预防控制中心，福建 福州 350001；
4 厦门市中小学保健所，福建 厦门 361003；
5 厦门市外国语学校附属小学，福建 厦门 361004
【作者简介】陈友兰（1979-），女，福建厦门人，大学本科，主管医师，主要从事健康教育与行为干预工作。

© 1994-2010 China Academic Journal Electronic Publishing House. All rights reserved. http://www.cnki.net

1.2 方法

1.2.1 检测方法　以厦门市外国语附属小学作为干预学校，选择各方面条件基本相同的厦门市大同小学作为对照学校。在干预学校开展"中国/WHO以肥胖控制为切入点发展健康促进学校"项目活动，在对照学校不开展相关活动。重点跟踪监测 820位三、四年级学生的身高和体重，学生一旦被纳为跟踪监测对象，无特殊原因不得退出监测，跟踪时间为 2004年 4月至 2007年 9月，每月测量一次，直至四年级学生升学毕业后，再继续随访 1年（2007年）。同时，对两校全体学生进行每年一次的体格检查和问卷调查，体格检查和问卷调查基本上同时进行，时间为 2004年 4月、2005年 4月、2006年 4月、2007年 11月和 2008年 12月。

1.2.2 测量仪器　使用同一身高体重仪测量项目学校和对照学校学生，并将测量结果录入问卷相应栏目中。

1.2.3 评价标准　以体质指数（BMI）为评价指标，以国际生命科学学会中国肥胖工作组（WGOC）提出的《中国青少儿超重、肥胖分类标准推荐建议》为评价标准[3]。

1.2.4 问卷调查　①学生问卷测量：使用的是中国健康教育中心统一印制的《学生膳食与运动、知识、态度行为调查问卷》，每年调查一次，与体格检查同时进行，问卷中的身高、体重资料由调查人员统一测量后填写，其余内容在经过统一培训的调查员进行必要的说明后，由学生当场填写；②家长问卷测量：调查家长的问卷有两种，一种问卷是基线调查问卷，在项目开始实施的第一次调查中使用，该问卷是参照相关研究[4,5]自行设计，并经过专家讨论和预实验后确定，问卷内容包括：一般情况（儿童、父母）、儿童出生成长情况、儿童饮食习惯（吃饭速度、饱食程度和饮食偏好等）和生活方式（睡眠时间、体育活动）等，目的是探索儿童肥胖的可能成因；另一种问卷是监测问卷，每年调查一次，使用的是中国疾病预防控制中心统一印制的《家长膳饮食与运动、知识、态度行为调查问卷》，问卷中的身高、体重资料由家长自行测量后填写。

1.2.5 干预策略　根据基线调查结果，在干预学校开展以下有针对性的干预措施，对照校不采取以下措施。

综合性策略：①成立学生肥胖控制领导小组；②出台与肥胖控制相关健康政策和措施，如每月身高体重监测制度，增加体育活动时间，增设膳食营养健康教育课；③把学生肥胖控制工作纳入班级和教师的年终考核；④举办预防超重、肥胖讲座与培训，开办儿童肥胖控制家长学校，利用广播、板报、宣传材料视频教学等宣传肥胖危害防治方面的知识；⑤学生按时填写《学生个人管理手册》，及时反馈能量摄入和消耗情况；⑥教师根据学生的反馈记录，开展有针对性的个性化指导；⑦改善体育设施，扩大运动场，增加体育锻炼时间；⑧督促家长引导儿童有节制地看电视、用电脑和玩电子游戏，减少不必要的正餐外高热量摄入等。

针对性策略：①学校营养午餐专门为超重和肥胖学生设计减肥食谱，在校内用餐的学生由教师监督食量，在校外由家长进行监督，纠正不良的饮食习惯；②肥胖和超重学生每月连续记录一周的食品摄入和运动情况，每周自测体重一次，学校每月监测身高、体重一次；③教师针对超重、肥胖学生开展膳食、运动、睡眠等指导；④肥胖超重学生由体育教师组织进行跳绳、踢毽球、打球等集体体育活动，逐步改善饮食结构，增加运动量；⑤心理介入治疗，鼓励肥胖儿童树立信心，减少心理负担，给肥胖儿童营造宽松环境。

1.2.6 数据处理　采用 EpiData软件编制的数据库进行数据录入，使用 SPSS 13.0软件进行数据处理及统计分析。基线调查将问卷的选项根据专业理论知识设置为有序变量，以预期危害因素的大小，从小到大排列，然后以 0、1、2、3对应由弱到强的影响关系，将有序变量变为数值变量，采用单因素卡方检验分析和多因素 Logistic回归分析。

2 结果

2.1 学生肥胖原因分析　调查对象是被纳入跟踪监测的学生的家长，为与学生共同生活，负责喂养学生的家长，一般指父母两人。在项目开始时共发出调查问卷 1640份，回收有效问卷 1614份，有效应答率为 98.4%。根据问卷调查内容，采用单因素卡方检验分析，筛选出 22个影响因素（$P<0.05$）为：性别、出生体重、周岁体重、父亲肥胖、母亲肥胖、父母至少一方肥胖、主食摄取量、进食速度、不吃早餐、饮食不规律、暴饮暴食、喜

© 1994-2010 China Academic Journal Electronic Publishing House. All rights reserved.　http://www.cnki.net

食肉类、经常零食、喜食蔬菜、喜食水果、喜食碳酸饮料、喜食油炸食品、喜食罐头食品、喜食糕点、饮食口味、喜食宵夜和体育锻炼。

进一步将单因素分析差异具有统计学意义的变量采用多因素 Logistic逐步回归分析（拟和优度检验，$x^2 = 94.42$，$P < 0.01$；进入标准 0.05，剔除标准 0.10），观察研究因素对回归模型的影响和交互作用（表 1）。有 15项影响因素差异具有统计学意义（$P < 0.05$），其中危险因素 14项，根据 *OR* 值大小判断其对肥胖影响力大小依次为：主食量大、母亲肥胖、喜食罐头食品、喜食碳酸饮料、缺乏体育锻炼、饮食口味重、喜食糕点、进食速度快、父亲肥胖、暴饮暴食、经常零食、饮食不规律和喜食肉类；保护因素 1项为喜食蔬菜。

表 1　学生超重和肥胖的影响因素多因素 Logistic回归分析

因素	*B*	*SE*	*Wald*	*Sig.*	*Exp*（*B*）	95% *CI*
性别	0.159	0.138	1.338	0.247	1.173	0.895–1.536
出生体重	0.215	0.200	1.150	0.284	1.240	0.837–1.835
周岁体重	0.104	0.184	0.321	0.159	1.110	0.774–1.593
父亲肥胖	0.592	0.286	4.282	0.039	1.808	1.032–3.170
母亲肥胖	1.050	0.295	12.654	0.000	2.856	1.602–5.093
父母至少一方肥胖	0.536	0.340	2.485	0.115	1.708	0.878–3.324
主食摄入量大	1.080	0.204	27.979	0.000	2.946	1.974–4.396
进食速度快	0.603	0.183	10.847	0.001	1.828	1.277–2.618
不吃早餐	0.415	0.178	5.404	0.020	1.514	1.067–2.147
饮食不规律	0.430	0.186	5.336	0.021	1.538	1.067–2.216
暴饮暴食	0.584	0.189	9.572	0.002	1.793	1.239–2.596
喜食肉类	0.403	0.189	4.538	0.033	1.496	1.033–2.167
经常零食	0.558	0.183	9.331	0.002	1.748	1.221–2.500
喜食蔬菜	–0.361	0.138	6.858	0.009	0.697	0.532–0.913
喜食水果	–0.214	0.139	2.360	0.125	0.807	0.615–1.061
喜食碳酸饮料	0.699	0.188	13.874	0.000	2.011	1.393–2.905
喜食油炸食品	0.298	0.202	2.177	0.140	1.347	0.907–2.001
喜食罐头食品	0.940	0.202	21.598	0.000	2.559	1.722–3.804
喜食糕点	0.605	0.173	12.298	0.000	1.831	1.306–2.568
饮食口味	0.640	0.169	14.341	0.000	1.896	1.361–2.640
喜食宵夜	0.249	0.207	1.443	0.230	1.282	0.855–1.924
缺乏体育锻炼	0.674	0.163	17.066	0.000	1.961	1.425–2.700
常数	–2.195	0.186	138.706	0.000	0.111	

2.2　干预前后两校 **820**名学生体重控制效果分析　2004年干预前，干预校和对照校学生 BMI指数分别为 19.634±3.561和 19.610±3.716，$P = 0.896$，差异没有统计学意义。

2007年干预后，干预学校与对照学校学生 BMI分别为 17.568±3.126和 20.457±3.469，进行干预后干预组与对照组的配对 *t*检验，$t = -17.715$，$P < 0.01$，差异具有统计学意义。

2.3　干预前后两校全体学生体质变化及控制胖肥相关知信行改变情况　2004～2008年对两校全体学生分别进行 5次身高体重监测和问卷调查，调查得到两校学生体质变化情况及控制肥胖知信行变化情况显示，干预校学生的肥胖率和超重率显著下降，分别从原来的 28.3% 和 17.4% 下降到目前的 9.9%和 15.0%，干预 5年效果显著，差别具有统计学意义（$x^2 = 150.322$，$P < 0.01$），干预校与对照校比较差异亦有显著意义（$x^2 = 154.943$，$P < 0.01$，表 2）。

干预校学生健康知识掌握水平由 2004年的 51.5%提高到 2008年的 95.3%，差异具有统计学意义（$x^2 = 611.096$，$P < 0.01$），实施肥胖控制项目 5年来，学生已经掌握了较多合理膳食、肥胖危害等方面的知识，并且掌握了控制体重和减肥的正确方法，干预校与对照校比较差异具有统计学意义（$x^2 = 307.571$，$P < 0.01$）；两校学生的正确态度持有率都在提高，但干预校学生的正确态度持有率变化显著，差异具有统计学意义（$x^2 = 202.652$，$P < 0.01$），表示干预校学生更愿意学习有关营养方面知识，参加运动锻炼和坚持每天运动，改变不健康的饮食习惯；干预校学生的健康行为形成率由 59.4%

© 1994-2010 China Academic Journal Electronic Publishing House. All rights reserved. http://www.cnki.net

提高到 97.2%, 变化显著, 差异具有统计学意义 ($\chi^2 = 520.466$, $P < 0.01$), 干预校与对照校比较, 差异也具有统计学意义 ($\chi^2 = 198.773$, $P < 0.01$), 干预学校学生饮食习惯得到了明显改变, 学习、休息和运动时间趋向合理且充足, 并且更自觉地参加运动锻炼和每天坚持运动。

表 2 两校学生体质变化情况及控制肥胖知信行变化情况 (%)

调查内容	干预校					对照校				
	2004年 ($n=1230$)	2005年 ($n=1246$)	2006年 ($n=1250$)	2007年 ($n=1248$)	2008年 ($n=1243$)	2004年 ($n=1228$)	2005年 ($n=1238$)	2006年 ($n=1245$)	2007年 ($n=1247$)	2008年 ($n=1251$)
BMI 正常率	54.3	63.3	70.5	72.1	74.7	58.0	53.5	56.9	55.2	54.2
超重率	17.4	17.8	17.1	17.0	15.3	14.5	17.7	15.5	16.6	17.2
肥胖率	28.3	18.9	12.4	10.9	10.0	27.5	28.8	27.6	28.2	28.6
健康知识掌握率	51.5	83.1	91.2	92.7	95.3	47.6	57.5	68.6	67.8	68.2
正确态度持有率	82.7	98.0	98.6	98.9	99.1	82.2	83.3	90.8	88.6	89.5
健康行为形成率	59.4	80.1	93.4	95.2	97.2	59.4	61.2	73.8	70.8	78.8

3 讨论

3.1 厦门市学龄期儿童单纯性肥胖主要归因为营养过度和饮食行为偏差 肥胖的发生是遗传和环境双重因素共同作用的结果[6], 亲代肥胖会使子女肥胖的风险增加, 特别是母亲的肥胖对儿童肥胖有显著的正性影响。有研究显示, 父母双方肥胖, 其子女大约有 70% ~ 80% 发生肥胖; 父或母其中一人肥胖, 子女发生肥胖的几率大约为 40% ~ 50%; 这也可能因为儿童的活动量、饮食习惯可能受父母和家庭的影响。排除遗传因素和疾病因素后, 肥胖的根本原因可以归结为摄食超量和能量消耗减少, 主食摄入过量是肥胖的物质基础, 肥胖与饮食特征 (如频繁进食[7]、食欲亢进、喜食肉类油腻、蛋类和甜食等) 密切相关[8]。久坐的生活方式是肥胖发生的重要危险因素, 缺乏体育锻炼是肥胖发生的重要危险因素[9]。

基线调查结果显示, 厦门市学龄期儿童单纯性肥胖的影响因素以营养过度和饮食行为偏差为主。因此, 控制食量、调整饮食结构、合理营养, 改变不良饮食习惯, 使能量的摄入与能量的消耗平衡, 对防止儿童肥胖的发生至关重要。由于学龄儿童的生活、运动和部分饮食形式主要由学校决定, 以学校为基础的行为干预模式, 被公认为预防儿童肥胖的重要干预措施。肥胖虽然有遗传倾向, 但营养过度, 运动不足, 行为偏差对肥胖的贡献更大, 所以防止学生肥胖应以运动为基本处方, 行为矫正为关键技术, 饮食调整和健康教育贯穿始终, 制定由医生、家长、学校、儿童共同参与的综合干预方案, 营造良好的体重控制氛围, 促进儿童健康成长[5]。

3.2 低年级学龄期儿童是肥胖预防控制的关键时期 学龄期儿童正处于生长发育阶段, 体重变化迅速, 是肥胖干预工作的重点阶段。同时学生肥胖控制工作必须从低年级抓起。2005年下半年, 项目组及时调整工作思路和方法, 将项目由二、三、四年级扩展到全校, 把健康行为习惯的培养作为低年级学生控制肥胖工作的重点, 开展内容丰富的实践活动, 让学生从实践中掌握健康知识, 从观念上养成良好习惯。执行上述干预措施后, 干预校学生的肥胖率和超重率显著下降, 说明积极的肥胖干预工作措施是正确的。研究结果也显示: 干预校学生健康知识掌握水平正确态度持有率健康行为形成率 2008年与 2004年相比差异具有统计学意义。说明通过一系列的干预活动, 干预校学生的健康知识、态度、行为得到强化, 逐渐改变了不健康的饮食习惯, 增加了参加运动锻炼和每天坚持运动的时间[10]。

3.3 儿童肥胖控制需要社会共同参与, 家庭干预尤为重要 儿童处在生长发育时期, 控制肥胖不宜提倡严格限食[2], 对膳食的调整应合理、适度。儿童对膳食和生活方式的自控能力低, 家庭和学校必须密切配合, 不能放松监测和干预。控制儿童肥胖需要社会环境的支持, 需要长期的、多方面的、可持续性的干预活动, 尤其是针对家庭开展干预[12]。有研究表明[11], 母亲教育水平对儿童膳食的影响, 主要表现在儿童的动物性食物摄入上, 说明若在能满足儿童生长发育和保持合理体重的基础上, 父母能做到合理搭配小儿膳食, 控制小儿发胖是促进儿童健康发育的有效措施。

(下转第 766页)

© 1994-2010 China Academic Journal Electronic Publishing House. All rights reserved. http://www.cnki.net

在特殊环境下的预防措施也未尝不可。

处于性活跃期的劳务人员在国外期间不可避免地面临如何缓解性压力的问题，对他们偶尔发生自慰行为应持宽容态度，某种程度上有利于避免发生性冒险行为。劳务公司应积极协调多开展一些文化娱乐活动，最好能写进劳务合同附加条款中，这样既能丰富业余生活，也能分散他们对性需求的注意力，对顺利完成境外劳务大有裨益。

各阶段干预后，一些劳务人员对 STD/AIDS仍持歧视和反对态度，他们担心会因日常生活中与 STD/AIDS感染者接触而染病。加强宣传消除恐惧和歧视心理是健康教育的重要内容，如果 STD/AIDS相关知识掌握得多了，人们会对艾滋病的恐惧或歧视心理有所弱化[5]。

劳务人员本质上还是农民工，是特殊的流动人群，健康教育已被许多国家证明是针对流动人口等社会弱势群体进行性病艾滋病干预的有效措施[6]，本次研究也得到满意效果[7,8]。劳务人员如从高流行区归国，应作为高危人群，加强后续管理，加大宣传力度，取得劳务公司配合，注意同劳务人员家人加强亲情联系，鼓励性伴发挥同伴教育作用，说服他们入境后主动咨询或志愿检测 STD/AIDS。

很多以行为改变为主要策略的疾病/健康干预已经表明[9]，只有持久的行为干预才能产生广泛的影响，进而使行为发生明显、稳定、持久的改变[10]。考虑到劳务人员态度改变是复杂、渐进、连续的曲折过程，需要持之以恒地教育才能最终改变态度。本研究选择“专业干预 + 同伴教育”相结合的模式，在 2年相对较长的时间内，保证了劳务人员 STD/AIDS态度干预总效果，对其今后一段时期预防 STD/AIDS也能起到积极的作用。

参考文献

[1] 陈岳坤. 安庆市建筑民工艾滋病相关知识健康教育效果评价[J]. 中国农村卫生事业管理, 2008, 28(3): 210.

[2] 马骁. 健康教育学[M]. 北京: 人民卫生出版社, 1996. 1.

[3] Johson AM. Condoms and HIV transmission[J]. N Engl J Med, 1994, 11: 391-392.

[4] 王艳梅. 黑龙江口岸赴俄罗斯劳务人员艾滋病健康教育效果的评估[J]. 中国国境卫生检疫杂志, 2008, 31(4): 252.

[5] 石健. 艾滋病知识、态度、行为的相关性研究[J]. 广西预防医学, 2005, 11(2): 77-81.

[6] 明中强. 艾滋病预防与控制的成功经验[J]. 国外医学流行病学传染病学分册, 1999, 26(5): 193-197.

[7] 钟海波. 流动人口艾滋病健康教育效果评价[J]. 实用预防医学, 2006, 13(1): 108-109.

[8] 张巧利, 刘金祥, 陈柏芬, 等. 不同职业流动人口艾滋病知识、态度和行为调查[J]. 中国健康教育, 2008, 24(2): 105-107.

[9] 季奎. 医学生吸烟行为健康教育干预效果的研究[J]. 预防医学情报杂志, 2001, 17(3): 186-187.

[10] 张开宁. 应对艾滋危机的公共管理与公共服务[M]. 北京: 中国人口出版社, 2005. 1.

[收稿日期] 2010-07-30　[本文编辑] 王晓春　庞宇

(上接第 759页)

鉴于此，要有效地控制学生肥胖，必须力求达到学校、家庭、社区合力引导学生行为的改变，而创建健康促进学校为肥胖干预控制提供了一个非常好的氛围和平台，学校应该成为儿童肥胖控制的主要场所之一。

参考文献

[1] 徐勇, 谭琪. 我国儿童青少年肥胖的现状及发展趋势研究[J]. 中国卫生事业管理, 2003, 19(3): 166-168.

[2] 马洪亮, 周虹茹, 刘枫, 等. 创建健康促进学校的实践与思考[J]. 中国健康教育, 2000, 16(6): 668-670.

[3] 国际生命科学学会中国肥胖工作组. 中国学生超重、肥胖 BMI 筛查标准[M]. 北京: 中国儿童青少年肥胖问题研讨会, 2003. 1-26.

[4] 马面生, 胡小琪. 影响我国四城市儿童青少年肥胖的环境和行为因素[J]. 中国慢性病预防与控制, 2002, 10(3): 114-116.

[5] 王小燕, 贾宝山. 我国儿童肥胖相关因素的系统评价[J]. 中国儿童保健杂志, 2008, 16(6): 708-710.

[6] Chaudry G, Navarro OM, Levine Ds, et al. Abdominal manifestations cystic fibrosis in children[J]. Pediatr Radio, 2006, 36(3): 233-240.

[7] Toschke AM, Kochenhoff H, Koletzko B, et al. Meal free-quince and childhood obesity[J]. Obesity Research, 2005, 13(11): 1932-1938.

[8] Styne DM. Obesity in child hood: what's activity got to dowith it[J]. American Journal of clinical Nutrition, 2005. 81(2): 337-338.

[9] Stachar I Duet in the management of weight loss[J]. MAJ, 2006, 174(1): 56-63.

[10] 陈友兰, 杨晓剑. 厦门市以肥胖为切入点发展健康促进学校项目中期效果评价[J]. 现代预防医学杂志, 2007, 34(21): 4001-4003.

[11] 蒋竞雄, 夏秀兰, 惠京红. 学校肥胖控制干预的研究[J]. 中国学校卫生, 2007, 28(1): 234.

[12] 李东海, 喻素彬, 徐耀初, 等. 儿童肥胖发病机制研究进展[J]. 预防医学情报杂志, 2003, 19(1): 24-26.

[收稿日期] 2010-03-31　[本文编辑] 王晓春

© 1994-2010 China Academic Journal Electronic Publishing House. All rights reserved.　http://www.cnki.net
