# Supplementary material for: Systematic review and meta-analysis of school-based obesity interventions in mainland China
Source: PLoS One. 2017 Sep 14;12(9):e0184704. doi: 10.1371/journal.pone.0184704 (PMC5598996; doi:10.1371/journal.pone.0184704)
Supplement: S1 Dataset — (ZIP) [file pone.0184704.s007.zip › S1_dataset/76库/81.pdf]

儿童超重、肥胖相关因素及干预措施分析

陆兆美　广西壮族自治区贵港市人民医院儿内一区　537100

中国图书分类号　R179　文献标识码　A　文章编号　1001-4411(2015)09-1393-02; doi:10.7620/zgfybj.j.issn.1001-4411.2015.09.29

【摘　要】　目的：分析儿童超重、肥胖的相关因素，调查实施干预措施后对儿童超重、肥胖的影响效果。方法：随机抽取本地区儿童 356 名（均为小学生），调查分析其超重、肥胖原因，并将其随机分为对照组（156 名）和观察组（200 名）。对照组不实施任何干预措施，观察组实施相应的干预措施，统计分析 1 年后两组儿童的超重、肥胖情况。结果：实施干预措施前，两组儿童肥胖、超重情况相近，差异无统计学意义（$P>0.05$）；实施干预措施后，观察组儿童超重率（5.5%）和肥胖率（3.5%）明显低于对照组儿童超重率（11.5%）和肥胖率（7.7%），两组比较差异有统计学意义（$P<0.05$）。结论：遗传因素、出生体重、饮食习惯、睡眠时间及运动时间等都是影响儿童超重、肥胖的原因，实施相应的干预措施能够有效改善儿童超重、肥胖现状，值得各地区推广实施。

【关键词】　儿童　超重　肥胖　影响因素　干预措施　效果

近年来，我国青少年超重率已达到 4.5%，肥胖率已达到 2.1%[1]，且呈持续上升的趋势。本研究选取本地区儿童 356 名，分析儿童超重、肥胖的相关因素，并针对其影响因素提出相应的干预措施，取得了一定效果。

1　资料与方法

1.1　资料来源　随机抽取本地区儿童 356 名（均为小学生），调查分析其超重、肥胖原因，并将其随机分为对照组（156 名）和观察组（200 名）。对照组中男生 85 名、女生 71 名，年龄 7～11 岁；观察组中男生 115 名、女生 85 名，年龄 7～11 岁。两组小学生性别比例、年龄、超重及肥胖等基本资料比较差异均无统计学意义（$P>0.05$），具有可比性。

1.2　研究方法　找出可能影响儿童肥胖的相关因素，并根据相关因素制定问卷调查表，对两组儿童及其家长进行问卷调查，分析两组儿童肥胖和超重原因，针对最具代表性的两种影响因素制定干预措施。对照组不实施任何干预措施，观察组实施相应的干预措施，统计分析 1 年后两组小学生的超重、肥胖情况。

1.3　影响因素分析

1.3.1　遗传因素　有研究[2]结果表明：肥胖受遗传因素影响，即肥胖父母所生子女发生超重和肥胖的概率要高于非肥胖父母所生子女，肥胖具有遗传性。

1.3.2　出生体重　母亲在怀孕期间为保证营养充分，通常会补充大量营养，导致营养超标，胎儿体型过大。有研究[3]结果显示：胎儿的出生体重越高，其发生超重和肥胖的概率越大，出生体重与儿童超重、肥胖呈正相关。

1.3.3　饮食习惯　儿童对饮食无理智克制能力，喜食高油脂的油炸食品和甜品饮料，常出现暴饮暴食现象，导致脂肪过度囤积从而形成肥胖[4]。进食速度也是影响儿童肥胖的重要因素[5]，由于进食速度过快，机体未及时发出饱腹感信号，导致儿童不自觉地过量摄入食物，从而引起肥胖。

1.3.4　体育活动　机体储存的能量将运用于身体的正常活动，适当的参加户外活动能够帮助机体消耗多余的脂肪和能量。有研究[6]结果表明：不参加机体活动的男孩发生超重、肥胖的危险性要高于常参加机体活动的男孩，说明运动对于预防儿童肥胖具有重要意义。

1.3.5　睡眠时间　人体每日应保证睡眠 8 h 才能够完全恢复精力，儿童由于沉迷于网络游戏、电子书或漫画等而导致睡眠时间不足，由于学习压力导致睡眠质量不高，利用周末或假日进行补眠等行为会增加青少年发生超重的风险[7]。

根据以上 5 种常见因素制定问卷调查表，调查发现 76 名肥胖、超重小学生中有 23 名（30.26%）饮食习惯不良，29 名（38.16%）运动量不足，11 名（14.47%）存在睡眠问题，5 名（6.58%）父母都超重，8 名（10.53%）存在其他相关问题。

1.4　干预措施分析　不良饮食习惯及运动量不足是影响青少年儿童肥胖的主要原因，针对这两种因素制定干预措施，具体如下。

1.4.1　科学饮食管理[8]　儿童饮食应注重营养，少食多餐，避免过饥或过饱，在校期间由学校统一安排饮食，按照早、中、晚定时定量供应食物，合理搭配营养，同时尽量少吃或不吃西式油炸类快餐和含糖分较高的饮料及零食，培养科学合理的饮食习惯，保证营养的同时杜绝营养过剩。宣传教育进食要细嚼慢咽，避免进食过快、暴饮暴食。

1.4.2　适当组织课外活动　单一的身体活动对于控制儿童体重的效果不明显，存在易反弹的风险，而通过学校干预实施规律性体育活动锻炼来控制儿童肥胖效果较好。通过学校来制定学生的体育活动计划，增加儿童身体活动量，如每天清晨组织跑步晨练、课间十分钟组织学生做有氧操或游戏等都能够有效增加儿童的机体活动量，从而消除多余脂肪。有研究结果表

?1994-2015 China Academic Journal Electronic Publishing House. All rights reserved.　http://www.cnki.net

明：学校组织体育活动，儿童肥胖率得到了明显改善[9]。

1.5　判断标准　根据中国肥胖问题工作组（IGOC）2003 年发布的关于中国儿童超重、肥胖分类标准进行超重、肥胖鉴定。

1.6　统计学方法　采用统计学软件 SPSS 19.0 对数据进行统计学分析，计量资料采用 t 检验，计数资料采用 χ^2 检验，$P<0.05$ 为差异有统计学意义。

2　结果

经过 1 年相关措施干预，观察组儿童超重率（5.5%）和肥胖率（3.5%）明显低于干预前水平（13.0% 和 8.5%），且明显低于对照组超重率（11.5%）及肥胖率（7.7%），两组比较差异均有统计学意义（$P<0.05$）。见表 1。

表 1　两组小学生超重、肥胖情况对比表〔名（%）〕

组别	超重		肥胖	
	1 年前	1 年后	1 年前	1 年后
对照组	20(12.82)	18（11.54）	13（8.33）	12（7.69）
观察组	26（13.00）	11（5.50）	17（8.50）	7（3.50）
P 值	0.025	0.038	0.032	0.050

3　讨论

影响儿童超重、肥胖的因素主要有遗传因素、出生体重、饮食习惯、体育活动及睡眠时间等。本文针对调查分析影响最明显的两项因素提出了相应的干预措施，主要以家庭和学校为干预措施实施地点，其中学校环境下的干预措施影响范围大、易操作、干预措施持久、有规律，获得的儿童体重管理效果更加明显，通过对饮食习惯、生活作息规律及活动量的干预来改善儿童肥胖、超重现象。

此外，预防儿童超重、肥胖需要家庭和学校的配合，以学校为基础单位，制作和发放宣传资料，由教师向儿童宣传肥胖的危害，教育儿童养成科学健康的饮食习惯。同时举办营养知识讲座，向家长讲解肥胖形成的原因、危害以及改善儿童肥胖的措施和方法，科学合理地管理儿童肥胖。家庭则应该注重培养儿童的良好生活习惯，为儿童建立健康的生活作息时间表，每日早睡早起，监督儿童不准熬夜看电视、玩游戏或看漫画书等，睡前给其饮一杯热牛奶帮助睡眠，利用儿童生物钟影响，使儿童一到晚间就快速进入睡眠状态。督促儿童早起，父母起带头作用，带儿童晨起锻炼或读书，改掉睡懒觉的坏习惯，锻炼注意连续性，避免一次性活动，即使节假日也不能间断，逐渐养成科学的作息规律。

随着宣传教育工作的不断开展、人们健康知识素质的不断提高及对生活质量要求的不断提高，儿童的肥胖问题越来越受到重视，从孕期开始就注意胎儿的营养状况，按照科学理念管理儿童的饮食及环境，培养儿童科学的作息习惯及良好的生活习惯，从而避免儿童肥胖问题的产生。未来儿童肥胖问题会在家庭、学校、医学界和社会的共同努力下而逐渐得到改善。

本次研究中实施干预措施 1 年后，观察组小学生的超重率（5.5%）及肥胖率（3.5%）明显低于对照组（11.5%，7.7%），两组比较差异有统计学意义（$P<0.05$），表明分析影响儿童超重、肥胖的影响因素，并针对影响因素实施相应的干预措施来控制儿童的超重、肥胖状况具有一定效果。有效地控制儿童体重增长需要家庭和学校的协作配合[10]，从儿童的生活和学习大环境着手，有条不紊又安全有效地改善中国儿童肥胖问题。

4　参考文献

1　林蓉，杜琳，刘伟佳，等. 广州市城区儿童超重肥胖影响因素分析〔J〕. 中国儿童保健杂志，2011，19（5）：409－411.
2　李友，许丽丽，陈艳兰，等. 大理市小学生超重肥胖状况及影响因素多水平模型分析〔J〕. 中国学校卫生，2013，34（1）：85－87.
3　任静朝，武俊青，李玉艳，等. 母亲妊娠期及围生期因素与儿童超重肥胖的关系〔J〕. 中国儿童保健杂志，2012，20（11）：991－994.
4　杨凡，王文娴，郭红卫，等. 上海市某区小学生超重肥胖的干预效果〔J〕. 环境与职业医学，2013，30（5）：333－337.
5　张茜，谭晓艳，于连龙，等. 山东省城市学龄儿童超重、肥胖现状及影响因素研究〔J〕. 中国儿童保健杂志，2013，21（5）：528－531.
6　王跃娜，栾振昌，马志君. 儿童肥胖运动干预的实践研究〔J〕. 当代体育科技，2014，4（2）：195－196.
7　张申尧，郭红卫. 上海市某社区学龄前儿童超重肥胖状况调查及健康教育〔J〕. 环境与职业医学，2014，31（2）：116－118.
8　高曦，孟派，廖中强，等. 上海市中小学生超重、肥胖相关的膳食模式分析〔J〕. 环境与职业医学，2014，31（1）：12－17.
9　李思杰，周琦，彭焱. 重庆某地区小学儿童超重和肥胖的危险因素研究〔J〕. 中国健康教育，2014，30（3）：235－237.
10　徐瑞芳，李丹华，丁玎，等. 小学生肥胖的学校－家庭－社区综合干预模式研究〔J〕. 环境与职业医学，2010，27（6）：342－345.

（2014-10-23　收稿）
〔编校　薛丽萍〕

?1994-2015 China Academic Journal Electronic Publishing House. All rights reserved.　http://www.cnki.net
