# Supplementary material for: Systematic review and meta-analysis of school-based obesity interventions in mainland China
Source: PLoS One. 2017 Sep 14;12(9):e0184704. doi: 10.1371/journal.pone.0184704 (PMC5598996; doi:10.1371/journal.pone.0184704)
Supplement: S1 Dataset — (ZIP) [file pone.0184704.s007.zip › S1_dataset/76库/82.pdf]

实验与调查
doi:10.3969/j.issn.1674-151x.2011.12.030

体育家庭作业对肥胖中学生BMI指数影响的实验研究

黄兆媛
（长春师范学院体育学院，吉林 长春 130032）

摘 要：本文采用实验法对体育家庭作业对肥胖中学生BMI指数影响的进行了实验研究。通过5个月（一个学期）的实验，结果显示，实验组学生的身体质量指数（BMI）下降，其中男生BMI指数差异具有显著性意义（P<0.05）、女生BMI指数差异具有高度显著性意义（P<0.01）。对照组学生的身体质量指数（BMI）无显著性意义（P＞0.05）。说明在实际体育教学过程中，应实行体育家庭作业制，加强学生对体育锻炼的认识，帮助其树立正确的体育观和良好的健身习惯，改善青少年的体质。

关键词：体育家庭作业；肥胖中学生；BMI指数；实验
中图分类号：G804.2 文献标识码：A 文章编号：1674-151X（2011）12-064-03

1 前 言

《中国青年报》于2010年3月30日刊发的报道《国民体质监测显示我国青少年体能连续10年整体下降》，引起了公众广泛关注和讨论。根据国民体质监测结果，我国青少年学生体能素质整体下降。2005年与1995年相比，学生的柔韧性、爆发力、肌力、耐力、肺活量均呈下降趋势。

这些问题如不切实加以解决，将严重影响青少年的健康成长。要解决这些问题，单靠每周几节体育课和体育活动课，其余时间不去参与体育活动，学生的整体健康水平是难以得到提高的。因此，给学生布置体育家庭作业，树立“科学运动、合理营养”的健康观念，促进学生利用校外的体育资源积极主动地参与体育锻炼是提高学生健康水平的有效途径。

2 研究对象与方法

2.1 研究对象和分组

被试对象为东北师范大学附属中学、长春市72中学、长春市103中学、长春市108中学、长春市汽车厂第10中学等5个中学，初二学生150人（男女生个一半）。参照《中国学龄儿童青少年超重、肥胖筛查体重指数（BMI指数）分类标准》在上述五所学校各筛选被试30人。被试要求：身体健康、无重大疾病、无遗传病史。150名被试分为实验组和对照组（控制组），每组各75人。

2.2 研究方法

2.2.1 文献资料法 在研究本课题的过程中，根据与本研究相关的青少年体质、健康促进等内容的需要，查阅中国电子期刊网及图书馆有关体质与健康、运动训练学、运动生理学、体育科研方法等方面的资料。

2.2.2 访谈法 通过访谈确定被试。即能否积极配合本实验、是否有健身习惯、有无家族病史、饮食习惯、生活方式、健身锻炼基本情况等内容。

2.2.3 实验法

（1）实验仪器。由韩国NEOMYTH公司研制的VIVENTE GOLD可以精确测试人体的身体质量指数，即BMI指数。

（2）实验测试指标。在两组实验前、后，分别对被试的身体质量指数（BMI指数）等数据进行采集，分析实验前后被试身体质量指数（BMI指数）的变化。

（3）实验周期。本研究采用实验法实验周期为5个月。

（4）实验刺激。实验组的实验刺激为体育家庭作业。在实验过程中要求学生按照规定完成家庭作业并填好体育家庭作业卡片，有家长和班主任监督，体育教师定期检查。体育家庭作业主要以身体素质练习为主：俯卧撑、30米折返跑、仰卧起坐、跳绳、原地高抬腿组等，男、女生的运动量要求不同，男生运动量稍大。要求：被试锻每天都要完成其中的两项，每次在10分钟之内完成，认真填好体育家庭作业卡片。对照组不施加任何实验刺激。

指导家长监督学生完成体育家庭作业的方法，并对家长提出制定合理膳食的科学的、合理的建议配合本实验的完成。

2.2.4 数理统计法 运用SPSS13.0软件对所获得的数据进行统计分析。

3 分析与讨论

3.1 相关概念界定

3.1.1 体育家庭作业 体育家庭作业是学校体育教学的延伸，是指体育教师指导学生进行自觉锻炼的有效形式，是培养学生对体育活动自觉参与的重要途径。主要是利用假期和课余时间通过体育课的学习，让学生自己课下查阅相关资料了解体育锻炼健身方法；指导学生根据个人的兴趣爱好，选择时间、场地、器材等设计适合自身特点的家庭作业卡片，并按照其执行健身（练习）计划。教师则要定期批阅、个别指导、定期检验练习效果。同时还邀请家长共同监督学生练习情况和练习效果。

3.1.2 BMI指数 BMI指数即身体质量

投稿日期：2011-09-08
作者简介：黄兆媛（1968~），副教授，硕士。研究方向：体育人文社会学。

© 1994-2012 China Academic Journal Electronic Publishing House. All rights reserved. http://www.cnki.net

表1　中国学龄儿童青少年超重、肥胖筛查体重指数（BMI指数）分类标准

年龄	超重		肥胖	
	男性	女性	男性	女性
7	17.4	17.2	19.2	18.9
8	18.1	18.1	20.3	19.9
9	18.9	19.0	21.4	21.0
10	19.6	20.0	22.5	22.1
11	20.3	21.1	236	23.3
12	12.0	21.9	24.7	24.5
13	21.9	22.6	25.7	25.6
14	22.6	23.0	26.4	26.3
15	23.1	23.4	26.9	26.9
16	23.5	23.7	27.4	27.4
17	23.8	23.8	27.8	27.7
18	24.0	24.0	28.0	28.0

表2　实验前两组被试BMI指数对比

	身体质量指数（BMI指数）$\bar{x}\pm S$	
实验组（150人）	男生（30.13±2.15）	女生（30.81±2.79）
对照组（150人）	男生（30.37±1.99）	女生（30.18±2.12）
T	1.756	1.692
P	＞0.05	＞0.05

表3　实验前、后被试BMI指数自身对照表

	实验组（150人）$\bar{x}\pm S$		对照组（150人）$\bar{x}\pm S$	
	男生	女生	男生	女生
前测	30.13±2.15	30.81±2.79	30.37±1.99	30.18±2.12
后测	28.25±3.93	27.61±3.31	29.87±2.95	30.16±3.31
T	−2.573	−3.48	1.587	1.479
P	＜0.05	＜0.01	＞0.05	＞0.05

指数，是与体内脂肪总量密切相关的指标，是检测全身性超重和肥胖的一个重要指标。由于青少年的生长变增，因此本实验采用《中国学龄儿童青少年超重、肥胖筛查体重指数（BMI指数）分类标准》。（表1）

3.2　实验结果

3.2.1　实验前测　表2数据显示，实验组和对照组的身体成分各项指标虽然略有差异，但均无差异无统计学意义（$p>0.05$）。也就是说实验前，实验组和对照组的被试身体成分这两项指标都基本接近。因此，这两个组被试的身体质量指数（BMI）这个参数具有可比性，可以进行对照试验。

表3显示了两组对比结果，实验组身体质量指数参数（BMI指数）发生了明显的变化，而对照组身体质量指数参数（BMI指数）没有明显的变化。实验组男生BMI参数差异具有显著性意义。特别是女生BMI参数差异具有高度显著性意义，而对照组身体质量指数（BMI指数）虽有下降，但差异无显著性意义。

3.2.2　实验结果分析　中学阶段是学生身体发育的最佳时期，本实验通过体育家庭作业弥补学校体育教学的有限性，让学生积极地参加身体锻炼和体育运动，有效地改善学生的体质和身体素质，对学生的身体、心理起到良好的促进作用。学生在体育老师的指导下，在家长的协助和班主任的督促下，利用业余时间完成的。它可以使学校学校、家庭、社会，全方位、多层次、多渠道的关心重视学生的健康成长。体育课家庭作业能够科学有效地促进学生自我锻炼，使学生有更多的机会、更多的场所、更多的时间参与更多的体育活动，培养学生的体育习惯。

实验结果表明（表3），通过5个月（一个学期）的实验，实验组的学生的身体质量指数参数（BMI指数）明显下降，男生BMI参数差异具有显著性意义；女生BMI参数差异具有高度显著性意义。对照组学生的身体质量指数参数（BMI指数）无明显变化。

男生的BMI参数下降没有女生BMI参数下降的明显，主要原因是：一是部分男中学生的自我管理能力较差，完成体育家庭作业的质量较差；二是通过完成体育家庭作业，身体成分发生明显变化，脂肪的重量减少，肌肉的重量相应的增加，体重下降不明显，导致BMI指数下降不明显；三是女生的自我管理能力较强，或者进入青春期的女生比较注重自己的身材，注意合理膳食，并能保质保量的完成体育家庭作业，BMI指数下降明显。

4　结　语

通过体育家庭作业，肥胖学生的BMI指数明显下降，有效地控制了肥胖中学生的体重。通过体育家庭作业的实施，学生合理利用课余时间，把教、学、练有机地结合起来，有利于实现课堂内外的一体化，肥胖学生建立了良好的体育健身习惯，不但提高了肥胖学生身体素质和健康水平，还调动了家长对于学校体育的积极性，为学生的健康成长创造了良好的家庭和社会氛围。

参考文献：

[1] 高嫌，张建华，毛振明．对我国基破教育与健康课程改革问题的反思[J]．北京体育大学学报，2007，(1) 75-78.

（下转第46页）

© 1994-2012 China Academic Journal Electronic Publishing House. All rights reserved.　http://www.cnki.net

况下进行每次 90min，每周两次，为期一年的有氧运动，同时还进行每周 3 次的家庭基本训练。其结果显示，TG 较训练前显著下降（P 小于 0.05）[7]。

本实验考虑到场地因素及运动强度的控制，采用快步走的运动方式，在实际康复中，可选用散步、慢跑、游泳、骑自行车、太极拳等运动。而长期锻炼对血脂影响的报道较多。据国内外报道，长期运动可使 TC、TG 以及 ApoB 水平降低，而 HDL-C 水平升高，运动后 TG 的变化，除少量研究无显著变化外，大部分均有明显下降 [7]。

3.2.3 运动强度对血脂水平的影响

根据实验结果，70% 最大心率的运动强度可以对各项血脂指标的良性变化产生显著效果。运动强度的变化主要对脂肪供能的比例产生影响，有研究表明，以 25%VO_2max 运动 30 分钟脂肪氧化供能占总供能的 70%~80%[4]，另一些文献表明，不需要较长时间的训练，低强度有氧运动就能对血清 TC 产生影响 [14]。故以中低强度的有氧运动对血脂的影响最大。而运动强度对脂蛋白的影响，目前仍存在争议。有研究认为，低强度的运动就可以有效提高 HDL 水平，大强度，大运动量的有氧运动和无氧运动均均不能使 HDL-C 产生良性改变 [15]。而另一些研究认为，只有较高强度的运动才能改善脂蛋白水平。现在较普遍认为 60%~80% 最大心率的有氧运动可明显改善脂代谢状况。

运动强度的控制还需考虑受试者自身的身体机能情况，本实验通过脉率的监控来调整实验强度。有研究表明，根据运动后脉率的恢复及主观感觉来判断，可有效控制运动强度。一般运动后休息 5min 脉率恢复到运动前的水平，说明运动量偏小；如超过 10min 还不能恢复， 则说明运动量过大 [7]。

3.3 对于中年高血脂症患者运动康复的建议

中年高血脂症患者可选用中等强度的有氧运动来实现高血脂症的康复，每次运动时间应在半个小时左右，若运动强度过大或时间过长会对机体造成损伤，强度过小或时间过短则运动康复效果不明显。还有中年人特别是中年高血脂症患者应经常进行体育锻炼，养成良好的运动习惯，有规律地参加体育运动对于高血脂症患者的康复有着重要的意义。

4 结 论

4.1 70% 最大心率强度有氧运动对受试者并未出现较大不良反应，且完成率较高，是中年高血脂人群锻炼的适宜运动强度。

4.2 为期 5 周，强度为 70% 最大心率的快步走，降低血清 TC、TG、LDL-C，升高血清 HDL-C，对血脂产生良好影响。

参考文献：

[1] 李克拉，尹瑞兴 . 我国人群血脂流行病学的研究进展 [J]. 中华医学实践杂志，2010，9（5）：28.
[2] 陆彩凤，戴海滨，沈云生 . 运动和饮食干预对男性高血脂公务员血脂及体质影响的实验研究 [J]. 吉林体育学院学报，2008，24（5）：83.
[3] 杨静宜，徐峻华 . 运动处方 [M]. 北京：高等教育出版社，2006：135.
[4] 张蕴琨，丁树哲 . 运动生物化学 [M]. 北京：高等教育出版社，2006：91.
[5] 金志清，关会涛 . 引发高血脂症的因素及防治 [J]. 齐齐哈尔医学院学报，2009，30（2）：191.
[6] 邓永明 . 有氧健身运动对高血脂症的调控作用 [J]. 河北体育学院学报，2004，18（3）：38.
[7] 关宇光 . 有氧运动对大鼠动脉粥样硬化发生发展过程中血浆及心肌血管紧张素 II 的影响 [J]. 体育科学，2005，25（7）：33.
[8] 中国肥胖问题工作组数据汇总分析协作组 . 我国成人体重指数和腰围对相关疾病危险因素异常的预测价值：适宜体重指数和腰围切点的研究 [J]. 中华流行病学杂志，2002，23（1）：6.
[9] 关宇光 . 有氧运动对大鼠动脉粥样硬化发生发展过程中血浆及心肌血管紧张素 II 的影响 [J]. 体育科学，2005，25（7）：33.
[10] 关乐林 . 中老年人高血脂对心血管病的影响 [J]. 亚太传统医药，2008，8（4）：18.
[11] 王薇 . 中国 11 省市 35-64 岁人群血清甘油三酯分布特点及与其他心血管病危险因素关系的研究 [J]. 中华流行病学杂志，2001（22）：25.
[12] 宋红梅，魏珉 . 儿童肾病综合征高脂血症的危害及其预防 [J]. 实用儿科临床杂志，2004，19（9）：729-731.
[13] 张奕秉，黄胡萍，孙娟，林志辉 . 血清甘油三酯和白蛋白水平与急性胰腺炎严重程度的相关性研究 [J]. 中国医学创新，2010，7（5）：13.
[14] 朱命祺，王建设 . 有氧运动对中老年高血脂症患者血脂水平的影响 [J]. 河南师范大学学报，2010，28（4）：172.
[15] 祁斌，张愉 . 运动对中老年男性血脂代谢水平的影响 [J]. 现代检验医学杂志，2010，25（4）：142.
[16] 史亚丽，辛晓林 . 有氧游泳运动对老年高血脂大鼠脂代谢影响的研究 [J]. 北京体育大学学报，2004，27（8）：1053-1054.

（上接第 65 页）

[2] 祁国鹰 . 体育统计应用案例 [M]. 北京：北京体育大学出版社，2005.
[3] 中国肥胖问题工作组 . 中国学龄儿童青少年超重、肥胖筛查体重指数值分类标准 [J]. 中华流行病学杂志，2004（2）：97-102.
[4] 王斌 . 影响中学生余暇体育锻炼行为的因素及策略 [J]. 上海体育学院学报，2005（2）：90-93.
[5] 江芸涵 . 体育也有“家庭作业”[N]. 四川日报，2007，6（12）.
[6] 裘松杰 . 合理布置小学体育家庭作业 [N]. 中国体育报，2007，7（11）.
[7] 马北北 . 国民体质监测显示我国青少年体能连续 10 年整体下降 [N]. 中国体育报，2010，3（30）.
[8] 马军 . 儿童青少年肥胖的运动干预 [J]. 中国学校卫生，2009（3）：198-200.

© 1994-2012 China Academic Journal Electronic Publishing House. All rights reserved. http://www.cnki.net
