# Supplementary material for: Systematic review and meta-analysis of school-based obesity interventions in mainland China
Source: PLoS One. 2017 Sep 14;12(9):e0184704. doi: 10.1371/journal.pone.0184704 (PMC5598996; doi:10.1371/journal.pone.0184704)
Supplement: S1 Dataset — (ZIP) [file pone.0184704.s007.zip › S1_dataset/76库/83.pdf]

· 调查研究 ·

综合群体干预对小学生肥胖预防的研究

王志秦，冯颖
（天津市滨海新区汉沽妇女儿童保健中心，300480）

摘要 : 目的　采用“ 三位一体 ”的综合群体干预措施，探索小学生肥胖防控的有效方法，为小学生肥胖群体防治工作提供依据。方法　选取 2010 年 4 月 - 2012 年 12 月滨海新区 6 所小学 6~11 岁 5434 名小学生进行体格监测，其中 2 所小学 2498 人为干预组，其他 4 所小学为对照组。结果　①干预后肥胖发生率降低，与对照组比较差异有统计学意义；②群体干预提高了家长对肥胖知识的知晓率。结论　实施“ 三位一体 ”的群体干预方法在干预期内可减少肥胖的新增人数，对儿童肥胖预防有较好疗效。

关键词 : 肥胖；饮食行为；群体干预

中图分类号 :R723.14　　文献标识码 :B

随着我国经济的发展和人民生活水平的提高，近年来小学生肥胖率急剧增高 [1]，这已成为公共卫生问题。超重与肥胖的发生在不同程度上影响儿童的身心健康，增加其成年后糖尿病、高血压、心脑血管等疾病的发生几率。本研究通过纠正儿童不良饮食习惯等措施采用群体干预法来探讨儿童肥胖的发生率，以期为儿童肥胖预防提供依据。

1　资料与方法

1.1 一般资料

采用整群抽样的方法对天津市滨海新区汉沽 6 所小学 6~11 岁学生进行体格监测 (内容包括身高、体重、血压、肺活量等)，监测时间为 2010 年 4 月 -2012 年 12 月。共计 5434 人，其中男 2973 人，女 2461 人。随机确定两所小学 2498 人 (其中男 1316 人，女 1182 人) 为干预组，实施群体干预措施，其他 4 所小学为对照组。两组小学生在城乡分布、性别、年龄等方面差异无统计学意义。

1.2 方法

1.2.1 诊断标准　根据中国学龄儿童青少年超重肥胖筛查 BMI 标准 [2]，统计肥胖发生情况，研究中不包括病理性肥胖患儿。

1.2.2 应用工具　干预组应用自制《儿童体重控制家庭指导手册》和《儿童肥胖防治多功能指导盘》(专利号 :201220138456.0) 进行指导，其内容包括儿童所需热卡、生理需要量、食物热卡计算、运动锻炼方法、营养健康自我监测方法等应用知识。

1.2.3 测量工具　现场采用符合国家标准的杠杆体重计、身高坐高计、血压计、肺活量计，并在每次测量前统一校正。

1.2.4 测量人员　由经过统一培训的儿童保健专业人员进行身高、体重、血压、肺活量的测量和内科检查工作。

1.3 “ 三位一体 ” 综合干预内容

1.3.1 医生干预指导　儿保医生通过讲座、图片、幻灯片等通俗易懂的形式对儿童家长讲解肥胖及超重相关知识，并发放《儿童体重控制家庭指导手册》和《儿童肥胖防治多功能指导盘》，教其使用，每学期 2~3 次。

1.3.2 家庭干预配合　家长利用《儿童体重控制家庭指导手册》，并按时记录孩子的饮食及运动量的平衡状况，随时应用《儿童肥胖防治多功能指导盘》，及时纠正孩子不良行为，儿保医生定期对家长进行电话随访，以增加家长与学生的依从性。

1.3.3 学校干预监测　学校保健医配合组织，由儿童保健医生具体实施，每季对干预组进行身高、体重、肺活量和血压的监测。

1.4 统计学方法

采用 SPSS 13.0 软件进行分析，组间比较采用 χ^2 检验方法，以 P<0.05 为差异有统计学意义。

2　结果

2.1 干预前后肥胖的发生情况

干预前，干预组肥胖发生率高于对照组，差异有统计学意义 (χ^2=4.929，P=0.026)。干预后，干预组肥胖发生率降低，并且小于对照组的发生率，差异有统计学意义 (χ^2=22.531，P<0.000 1)，见表 1。

表 1　干预前后肥胖的发生情况 (n,%)

组别	干预前		干预后	
	肥胖	非肥胖	肥胖	非肥胖
干预组	719(28.8)	1 779(71.2)	485(19.4)	2 013(80.6)
对照组	766(26.1)	2 170(73.9)	728(24.8)	2 208(75.2)

2.2 健康教育情况

群体干预采用讲课、看幻灯片和发放宣传材料等形式多样的健康教育宣传形式，群体健康教育覆盖率达 95% 以上。干预前，干预组知晓率与对照组知晓率比较，差异无统计学意义 (χ^2=3.267，P=0.071)。干预后干预组家长整体对儿童《饮食行为与运动相关知识》知晓率达 95.8%，高于对照组 82.0%，差异有统计学意义 (χ^2=249.92，P<0.001)。

表 2　两组儿童家长《饮食行为与运动相关知识》知晓率情况 (n,%)

组别	干预前		干预后	
	知晓	非知晓	知晓	非知晓
干预组	1 953(78.2)	545(21.8)	2 393(95.8)	105(4.2)
对照组	2 354(80.2)	582(19.8)	2 407(82.0)	529(18.0)

3　讨论

3.1 不良饮食习惯矫正，家长责任重大

单纯性肥胖症的发生环境因素起着重要作用，而环境因素中生活方式和个人行为模式是主要危险因素 [3]。儿童饮食行为的形成取决于家庭环境和家长的生活方式，本研究中，在《饮食行为与运动相关知识》调查时，肥胖儿童中狼吞虎咽的饮食方式、上网、看电视时间长、喜食油炸油腻食品、户外活动时间短等不良习惯较明显。研究认为要使干预对象饮食习惯发生良好转变，家长以身作则是很重要的，改变自身饮食习惯和

(下转第 58 页)

收稿日期 :2013-08-13

?1994-2015 China Academic Journal Electronic Publishing House. All rights reserved.　http://www.cnki.net

3　讨论

孕产妇保健管理是从确定妊娠开始到产后 42 d 之内，以母子共同为监护对象，按照各期所规定的检查项目进行系统检查、监护和保健指导，及时发现高危并及时转诊治疗，确保母婴安全 [4]。 开展农村孕产妇保健管理，通过孕期保健做到预防为主，达到减少甚至消除对母婴的有害因素，是降低农村孕产妇和围产儿死亡的有利措施。

2009 年农村孕产妇住院分娩补助项目和国家基本公共卫生项目在马龙县实施以来，在全县 66 个村委会和社区进行了孕产妇规范管理，几年的管理情况证明，必须按照管理规范要求认真完成，把好质量关，才能有效降低孕产妇死亡率和婴儿死亡率，确保母婴安全。在各级政府和业务主管部门的支持、指导下，马龙县妇幼卫生队伍逐年壮大，人员业务素质有所提高、基层网络逐步健全、孕产妇保健服务全覆盖，住院分娩率由 2009 年的 89.06% 上升到 2012 年的 99.31%，产前检查、产后访视人均次数逐年增加。国家政策支持、政府经费投入，使妇幼卫生工作得到进一步发展，妇幼卫生各项任务指标从数量到质量都有较大提高。

马龙县地处边陲，受地理环境、自然条件、经济 、人文等方面影响，妇幼卫生工作处于初级阶段。从几年数据来看，孕产妇保健管理工作发展缓慢，极不平衡，从整体水平来看管理率普遍提高，但在边远山区，文化素质偏低、经济极不发达、技术力量薄弱、设施设备简陋、人才紧缺等条件下要达到孕产妇系统管理要求，尚有很大差距；早孕检查率偏低，检查项目不全，产前检查、产后访视达不到管理要求，质量低；由于村医文化素质偏低，普遍存在保健手册填写不认真、不准确、不规范，错、漏项多；卫生院、卫生所对高危孕产妇的筛查、管理和监护工作不到位；再者由于缺乏所需的设备和适宜技术，导致抢救措施不力，处理不规范，转诊不及时，制约了妇幼卫生工作的正常开展。保健管理质量得不到保障。

妇幼卫生工作是一项社会性的系统工程，需要国家政策扶持和全社会的关注，更需要各部门的密切配合。近年来，妇幼卫生相关项目的实施，落实了边远贫困地区孕产妇、危急孕产妇的救助。完善县、乡、村三级保健网，着实解决县、乡、村保健人员的待遇，增强责任心，切实提高保健服务质量，把孕期保健服务工作落到实处；加强高危孕产妇的筛查和管理工作，认真落实“ 云南省孕产妇系统管理实施办法 ” 实行分级管理，明确各级职责，结合马龙县实际，根据不同人员层次和工作任务，强化培训；加强妇幼保健知识宣传，注重边远山区的教育，认真落实计划和流动人口的管理，使他们享有同样的保健服务，防止漏管和脱管。

综上所述，提高管理质量，把农村孕产妇保健服务真正落到实处，需要国家政策支持、地方政府高度重视、卫生行政部门制定强有力的综合性监管措施，通过稳定基层人员、提高业务素质、掌握适宜技术、增加硬件设施、改善服务条件等措施，才能使新形势下农村妇幼卫生工作更上一个台阶。

参 考 文 献

[1] David A Nagey.The content of prenatal care[J].obstetrics &cynecology,1989,74:516-526.
[2] Hollander D.Prenatal benefits improve birth outcomes among working Mexican women[J]. Intfam plan prospect,1997,23(4):94.
[3] 乐杰 . 妇产科学 [M].7 版 . 北京 : 人民卫生出版社 ,2010:44.
[4] 云南省卫生厅 . 云南省农村孕产妇系统管理实施办法 (试行)[S].2010.

(上接第 56 页)

对食物的偏好，在家庭中实施平衡膳食，安排适合于儿童的食谱，从根本上减少儿童单纯性肥胖症的发生与蔓延。

3.2 儿童保健医生的指导应社区化、长期化

与以往对单纯性肥胖症的群体实施干预的研究报道 [4] 和实行综合干预方式的研究 [5] 的不同之处在于，本研究采用自制的《儿童体重控制家庭指导手册》和《儿童肥胖防治多功能指导盘》，使控制肥胖儿童体重、纠正不良饮食和开展监测更加直观、便利。针对肥胖儿童，儿童保健医生联合学校保健医，将如何纠正不良饮食行为习惯、如何合理安排安全运动等内容设为学生的 “ 健康课堂 ” 、“ 必修课程 ” ，使儿童保健医生走进学校。此外，不仅针对肥胖儿童，本研究将正常人群也列入培养良好饮食习惯之列，使其发展为肥胖的几率大大下降。本次研究不同性别、年龄、文化程度和经济状况的家长对《儿童饮食行为与运动相关知识》的知晓率不同，提示儿童保健医生针对家长的健康知识宣传时间应提早，范围应社区化。

3.3 学校是开展宣传的良好阵地

儿童肥胖多属单纯性肥胖，学校与家长应对儿童从小加强健康理念教育，建立 “ 健康第一 ” 的理念，使儿童从小树立良好的饮食行为观念，认识到肥胖的危害，认识到自身存在的危险行为，形成想要改变的意识 [6]。本研究将继续应用，并希望将儿童肥胖防治管理纳入儿童保健工作内容，使儿童肥胖防治工作更加系统，效果更加持久。

参 考 文 献

[1] 覃世龙 , 贾翠平 . 孙雯 , 国内外儿童伤害流行病学特征比较 [J]. 中国社会医学杂志 ,2006,23(4):93-96.
[2] 中国肥胖问题工作组 . 中国学龄儿童青少年超重、肥胖筛查体重指数值分类标准 [J]. 中华流行病学杂志 ,2004,25(2):97-102.
[3] 王敏 , 李燕 , 刘锦桃 .3~6 岁儿童肥胖和营养不良与血压的关系 [J]. 中国妇幼保健 ,2008,23(1):183-184.
[4] 丁宗一 . 中国儿童单纯性肥胖症研究历史与现状及展望 [C]. 全国肥胖防治专题学术会议论文选集 ,2007,1.
[5] 张小君 , 李寿融 . 对儿童肥胖采取综合干预措施的研究 [J]. 中国实用医药 ,2012,7(34):256-257.
[6] 李松艳 , 洪倩 , 王德斌 . 行为改变理论在儿童单纯性肥胖症干预中的应用 [J]. 中国健康教育 ,2010,26(5):396-397.

?1994-2015 China Academic Journal Electronic Publishing House. All rights reserved. http://www.cnki.net
