# Supplementary material for: Systematic review and meta-analysis of school-based obesity interventions in mainland China
Source: PLoS One. 2017 Sep 14;12(9):e0184704. doi: 10.1371/journal.pone.0184704 (PMC5598996; doi:10.1371/journal.pone.0184704)
Supplement: S1 Dataset — (ZIP) [file pone.0184704.s007.zip › S1_dataset/76库/84.pdf]

北京体育大学

硕士学位论文

运动对肥胖少年体成分、体脂分布及其身体素质的影响

姓名：王蓓蓓

申请学位级别：硕士

专业：人体运动科学

指导教师：王正珍

20050501

中文摘要

随着人类物质生活水平的不断提高，单纯性肥胖症成为 21 世纪严重的健康问题。目前，发展中国家尤其是亚洲和一些太平洋岛国，肥胖发病率也在迅速上升，肥胖少年的增长比例更大，成为成年人肥胖的强大后备军，对整个国民素质构成潜在的威胁。肥胖问题的研究已逐渐引起人们的关注，但是对单纯性肥胖症的研究多集中在中青年人和老年人，对刚进入或即将进入快速生长期青少年的单纯性肥胖症系统研究不多，本文就实施运动处方后，肥胖少年体脂含量、脂肪分布、身体素质等指标的变化做一探讨，并与没有参加锻炼的肥胖少年作比较分析，以期对单纯性肥胖少年运动干预提供科学依据。

本试验选取单纯性肥胖少年共 45 名，以自愿的形式分为肥胖运动组 27 名，肥胖对照组共 18 名。通过递增负荷实验测得个体最大摄氧量，根据每个受试者不同的最大摄氧量,制定个性化运动处方，运动过程中用心率遥测仪控制运动强度。 运动组实施 12 周的运动处方，对照组除日常体育课之外，不进行其他锻炼。

结果发现：

12 周的有氧运动使男女肥胖少年身体成分发生良好改变，主要表现为瘦体重增加，体脂百分比减少，BMI 降低，肥胖度减少。男肥胖少年更着重于瘦体重的增长。

12 周的有氧运动使男女肥胖少年上臂、背部、腹部皮褶厚度减少。12 周的有氧运动能显著减小女性肥胖少年身体围度和腰臀比。但对男性肥胖少年围度影响效果不明显。

12 周的有氧运动增强了肥胖少年的身体素质，使运动协调能力和体育成绩显著提高。男生运动能力提高幅度比女生大。

关键词： 肥胖少年 体成分 皮褶厚度 身体围度 身体素质 运动能力

Abstract

As the continuous improvement of human living standard, obesity has become the most serious health problem in the 21st century . Recently , the growth of obese youngster increase rapidly, Becoming the offspring of the adult obese. It is a potential threaten . The researches about obese problem gradually attract people ’s attention. But the researches mostly focus on the adolescent and the elderly. There are not too much systemic researches about the obesity of the rapid growing adolescent. After implementing of a sport prescription to the obese adolescent, this paper is going to discuss about the indexes change of body component 、fat distribution 、function capacity etc. Expecting to offer some scientific evidences to sport intervention of obese adolescent.

This experiment select 45 obese adolescent totally, dividing into two groups voluntarily. 27 adolescent in sport group and 18 adolescent in compared group. Through experiment of increasing the load gradually to get each adolescent ’s VO2max, according to different VO2max of each one to control sport intensity by heart rate . Sport group implement a 12-weeks long sport prescription and compared group without any exercise except daily physical education [PE).

The results show:

12-weeks aerobic sport exercise let body component changed properly. It mainly shows in the increase of lean body mass, and simultaneously in the decrease of body fat percentage. The degree of obesity declined and the rate of basic metabolism increased. Male obese adolescent emphasize on the increase of lean body mass.

12-weeks aerobic sport exercise makes different degree of decrease of the male and female obese adolescent ’s skin-drape thickness.

12-weeks aerobic sport exercise makes the decrease of waistline、waist-to-hip ratio of the female obese adolescent. But the exercise does not have much influence on the body surround of the male obese adolescent.

12-weeks aerobic sport exercise strengthen the function capacity of the obese adolescent. Mainly showed in the improvement of strength in male obese adolescent. 12-weeks aerobic exercise also makes an obvious improvement of the obese adolescent ’s body coordination and sport ability, especially in male obese adolescent.

Key words: aerobic exercise obese adolescent body component
Skin fold function capacity Sport ability.

1 前言（文献综述与选题依据）

随着人类物质生活水平的不断提高，单纯性肥胖症成为 21 世纪严重的健康问题。肥胖不仅在欧美等发达国家的发病率很高，而且在发展中国家也开始迅速上升。肥胖不仅影响人的外形和心理，为工作和生活带来不便，而且与某些慢性疾病的发生密切相关[1]，还会缩短人的寿命[2]，已成为当今世界所面临的严峻公共健康问题。因此，防肥和减肥成为人们关注的焦点。

肥胖是一个全球公共健康问题[3]，至今仍没有一个满意的治疗方法。据推测，目前全球肥胖者至少有 2-5 亿，而且正以每 5 年增加 1 倍的速度增多。欧美等发达国家发病率很高，每年因肥胖造成的直接与间接死亡达 30 万人[4]，发展中国家尤其是亚洲和一些太平洋岛国，肥胖发病率也在迅速上升，与肥胖相关的疾病发病率也呈上升趋势。我国的肥胖症患病率近年来也呈上升趋势[17]。1998 年，中国在 14-64 岁的人群中，超重人数已逾 1 亿，预计 2002 年将达到总人口的 25%。其中，北京市人口肥胖率高达 40%，位居全国之首。

肥胖少年的增长比例更大，成为成年人肥胖的强大后备军，对整个国民素质构成潜在的威胁[52]。北京市国民体质监测中心的调查显示，1996 年，全国中小学生肥胖的检出率为 3%－5%，北京的这一数字略高于全国，为 7%－9%，到了 2001 年，全国中小学生的肥胖率约为 5 %－7%，而北京竟达到了 15%－ 17%，部分地区更是高达 25%－30% 。1979 年至 1995 年对我国 16 个省会城市汉族学生身体形态机能和素质的动态分析发现，我国 7 一 17 岁城乡男女学生的发育速度处于快速增长阶段，但身体机能和运动能力全面下降，具体表现为体重增加的幅度与身高增长不协调，体脂重量和体脂百分率显著增加，肺活量、肌肉力量和耐力水平等出现明显的负增长。

肥胖问题的研究已逐渐引起人们的关注，但是对单纯性肥胖症的研究多集中在中青年人和老年人，对刚进入或即将进入快速生长期青少年的单纯性肥胖症系统研究不多，尤其是关于肥胖少年体脂含量、脂肪分布以及实施运动处方后的干预情况的研究较少，本文就上述问题做一探讨，并且通过对肥胖少年运动干预前后体成分、身体素质的变化分析对单纯性肥胖少年运动干预的有效手段。

1.1 肥胖程度的评定

肥胖是体内脂肪积累过多而导致的一种状态，被认为是一种易被发现的、明显的、复杂的代谢失调症，是一种可影响整个机体正常功能的生理过程。

判定肥胖最常用的指标是 BMI、体脂百分比(Fat%)及肥胖度。近年来国际流行的标准体重测量方法，是 WHO 推荐的国际统一使用的肥胖分型标准参数。计算公式如下:体重指数（BMI）＝实际体重(Kg)/身高(m^2)。利用体重指数衡量人体肥胖程度，其特点是受身高的影响较小，该方法的局限性在于不能反映局部体脂的分布情况。1998 年世界卫生组织将 BMI ≥ 25kg/m^2 和≥30 kg/m^2，分别定为超重和肥胖。与欧洲人相比，亚洲人在较低的 BMI 时便出现代谢性疾病[13-14]。国际生命科学学会中国办事处中国肥胖问题工作组针对亚洲人制定标准 BMI ≥24kg/m^2 为超重，≥28 kg/m^2 为肥胖。

估算的理想体重(kg) :身高(cm)－105 。或者(男性)理想体重(kg):[身高(cm) －100]×0.9；(女性)理想体重(kg):(身高(cm)－100) ×0.85。实际体重超过理想体重的百分数即为肥胖度，即肥胖度: [（实测体重－标准体重）/标准体重]×100%。 正常为士 10%, 大于等于 10%为超重，大于等于 20%为肥胖。理想体重与肥胖度的计算已广泛应用，但有一定的局限性，精确度不高。用 Fat%判定肥胖一般以>20%作为诊断标准[15],一般以超出标准体重的 20-30%为轻度肥胖，超出 30-50%为中度肥胖，超出 50%为重度肥胖[16]。

最近国际生命科学学会中国办事处中国肥胖问题工作组根据对我国人群大规模测量数据，汇总分析了体重指数与相关疾病患病率的关系，提出对中国成人判断超重和肥胖程度的界限值，及结合腰围来判断相关疾病的危险度，其建议[26－27]如下：

表 1　中国成人超重和肥胖的体重指数和腰围界限值与相关疾病危险的关系

分类	体重指数 (kg/m2)	腰围（cm）		
		男：< 85<br>女：< 80	男：85-95<br>女：80-90	男：≥95<br>女：≥90
体重过低	<18.5	—	—	—
体重正常	18.5 - 23.9	—	增加	高
超重	24.0 - 27.9	增加	高	极高
肥胖	≥28	高	极高	极高

这项建议是根据 1990 年代以来我国 13 项大规模流行病学调查，总计约 24 万成人的数据汇总分析得出的。结果表明： BMI ≥ 24 kg/m^2 者患高血压的危险是体重正常（BMI]18.5-23.9 kg/m^2）者的 3-4 倍，患糖尿病的危险是体重正常者的 2-3 倍，具有 2 项

及 2 项以上危险因素[即危险因素聚集，主要的 5 个危险因素包括血压高、血糖高、血清总胆固醇高、血清甘油三酯高和血清高密度脂蛋白胆固醇降低]的危险是体重正常者的 3-4 倍。BMI≥28 kg/m^2 的肥胖者中 90%以上患有上述疾病或有危险因素聚集。

男性腰围达到或超过 85 厘米，女性腰围达到或超过 80 厘米者患高血压的危险约为腰围低于此界限者的 3.5 倍，其患糖尿病的危险约为 2.5 倍；其中有 2 项及 2 项以上危险因素聚集者的危险约为正常体重者的 4 倍以上[28]。在 10 个地区对 24900 名 35-59 岁人群的调查中，冠心病事件、脑卒中和缺血性脑卒中事件对超重和肥胖的归因危险度分别为 32.0%、30.6%和 53.5%，即这些疾病的发病由超重和肥胖引起的可能性很大[29]。这些证据表明，上述对体重指数划分界值的建议，对中国成年人群的肥胖防治一般是适用的。 值得注意的是在青年期体重指数即超标者，以后患相关疾病的危险度可能比中老年后才肥胖者更高[30—32]。

腰臀比(Waist-to-Hip Ratio, WHR)是腰围和臀围的比值，WHR 是区分脂肪分布类型的指标，WHR 偏高者为中心性肥胖，低则为周围性肥胖。WHO 推荐的测量腰围和臀围方法:腰围是受试者取站立位，双足分开 25-30cm 以使体重均匀分布，在肋骨最下缘和髂骨上缘之间的中点水平，在平稳呼吸时测量。臀围在臀部[骨盆]最突出部测量周径最突出部测量周径。男性 WHR > 0.90 为中心性肥胖，女性 WHR >0.85 为中心性肥胖。优点是能很好地反映腹内脂肪的变化，但测量者经验、手法等会影响结果[24]。

1.2 肥胖的测量方法

1.2.1 水下称重法

水下称重法[24]是一种经典的、基本的、可靠的方法，是测定体脂百分含量的“ 金指标 ”。它主要根据阿基米德的浮力原理，把人体大致分为脂肪重量(fat mass)和无脂肪重量(fat-free mass)两部分，依据公式求出人的体积和密度，进而得出体脂百分含量。

这一方法的优点是结果较准确，误差小。缺点是耗时多，所用仪器携带不方便，并需要被测对象的配合，在幼童、老年人和病人中的应用非常困难甚至不可行，另外不能测定局部体脂含量。

1.2.2 同位素释法

由于脂肪组织几乎无水分，所以通过测定体内水分的含量，可以计算出人体除脂肪以外身体的重量，并由此可知体内脂肪的重量。方法是将氘[氢的同位素]标记的定量重水注射入人体，经过 2 小时[重水均匀分布在体内除脂肪以外的各部位]后测定体液中氘的浓度，可以计算出体内总水量，进一步得出去脂体重和体脂百分含量。

此方法的优点是测定值的变异系数小，误差为 1%左右。缺点是价格昂贵、技术难度大、同位素的不良影响以及不能测量局部体脂。

1.2.3 生物电阻抗法

脂肪组织和其它含水量大的组织电阻抗不同。人体内含脂肪百分比越大，其电阻抗就越大，导电能力越小，故可从身体导电性和电阻程度间接计算人体脂肪组织百分比。具体方法是：用 50kHz 的单频或变频交流电，将一对电极置于受试者的上肢和下肢测量阻抗，根据公式计算人体水分含量和体脂含量。

此方法的优点是价格相对低廉、快速简便、重复性好，可以在床边检查，测定结果和水下称重法十分接近，适用于流行病学调查,但是此法不能测量局部体脂。

1.2.4 双能 X 线吸收法

用两束能量不同的微弱 X 线穿过人体，通过 X 线衰减程度的差异间接计算出体内非脂肪组织、脂肪组织和骨矿物质的含量。 这种方法的优点是安全、方便、精确度高。缺点是价格昂贵，检查对象有体重的限制，适用于体重小于等于 150kg 的个体，亦不能测量局部体脂。

1.2.5 其他

超声检测(Ultra-sound Technology)：发射脉冲超声进入人体，不同的组织有不同的回声强度和声衰减，含水多的组织声衰减小，脂肪组织含水量少，其声速比其他组织低，与相邻的皮肤肌肉组织的回声特性有明显的差异，可从声像图上分辨出脂肪组织的边界，并测量其厚度。

计算机断层(Computed Tomorgraphy, CT)：以 X 线辐射受试者，得到一系列准确性高的图像。根据扫描层面或节段的脂肪组织面积及体积来评估总体脂含量和局部体脂。一般采用

脐孔或第 4-5 腰椎间水平扫描，计算腹腔内脏脂肪面积，是诊断腹部型肥胖最精确的方法之一。

磁共振显像：这是 80 年代发展起来的一种全新的影像检查技术，对人体无放射性损失。根据扫描层面脂肪组织的面积、体积推算总体脂肪含量和局部体脂。

整体电传导：利用脂肪和水分对电磁场反应的不同，估测人体脂肪含量

中子激活法：此法可在原子水平测定脂肪含量。原理:用己知能量的快速中子轰击受试者，激活体内的化学元素，通过发射的 Y 射线识别被激活的化学元素。同位素的半衰期长短不一，可在两个不同时相进行扫描，准确测定不同的同位素，计算出体脂含量

红外线感应法(Near Infra-radinteractance)：此法利用红外光对皮肤有良好的穿透性以及其背向散射与脂肪厚度呈线性关系的原理，实现了人体皮下脂肪厚度的红外无损检测。

体钾测定法：因体内脂肪组织含钾量极微，对人体所有器官存在的钾进行放射性追踪，即通过计算放射性同位素在体内的分布来估测体钾含量，并可计算出人体脂肪含量。

综上所述，目前尚无一种既简单又精确的测定体脂的理想方法。故可根据不同的目的选用不同的测定方法。传统的水下称重法己趋于被生物电阻抗法及双能 X 线吸收法取代。整体电传导、中子激活法、红外线感应法、体钾测定法等因需特殊设备、价格昂贵、方法繁杂、操作难度大等原因而不适用于常规的检查。实际操作中上应推荐使用简单、准确和价廉的测定方法。如评估总体脂，可选用上述的体重指数、肥胖度等参数，或通过生物电阻抗法和双能线吸收法测定。如评估局部体脂或腹型肥胖，可测量腰围、腰臀比，尚可根实际条件选用超声、CT 或 MRI 等检查来测定总体脂肪和局部体脂。

1.3 肥胖对健康的危害

肥胖症患者往往有高血压、高血脂和葡萄糖耐量异常，肥胖是影响冠心病发病和死亡的一个独立危险因素[18]。肥胖症患者多在餐后较长时间内血脂持续在较高水平，富含甘油三酯的低密度脂蛋白(LDL)中的较小而致密的颗粒有直接致动脉粥样硬化的作用。大量研究表明[5—8]，肥胖与心血管疾病、糖尿病、胆囊疾病、骨关节病、肾病及恶性肿瘤等的发生密切相关，甚至会缩短人的寿命，体重超出理想体重 5 磅左右即可使寿命缩短一年[9]。另外肥胖者患其它非致命性疾病的机率也升高，如背痛、关节炎、不育、免疫功能低下及睡眠呼吸异常

等症[10]，还会因体态臃肿，行动不便而引发心理障碍[11－12]。

防治超重和肥胖症的目的不仅在于控制体重本身，更重要的是肥胖与许多慢性病有关，控制肥胖症是减少慢性病发病率和病死率的一个关键因素。根据世界卫生组织的报告，与肥胖相关疾病的相对危险度见下表。

表 2　肥胖者发生肥胖相关疾病或症状的相对危险度*

危险性显著增高<br>危险性显著增高(相对危险度大于 3)	危险性中等增高<br>(相对危险度 2-3)	危险性稍增高<br>(相对危险度 1-2)
2 型糖尿病	冠心病	女性绝经后乳腺癌，子宫内膜癌
胆囊疾病	高血压	男性前列腺癌，结肠直肠癌
血脂异常	骨关节病	男性前列腺癌，结肠直肠癌
胰岛素抵抗	高尿酸血症和痛风	多囊卵巢综合征
气喘	脂肪肝	生育功能受损
睡眠中阻塞性呼吸暂停	背下部疼痛	麻醉并发症

注 :相对危险度是指肥胖者发生上述肥胖相关疾病的患病率是正常体重者对该病患病率的倍数

1.3.1 高血压

肥胖者的肥胖持续时间越长,高血压患病率高，尤其是女性，发生高血压的危险性越大。而控制饮食和增加运动使体重降低时，使血容量、心排血量和交感神经活动下降，血压也随之降低[19]。

对我国 24 万人群的汇总分析显示，BMI≥24 者的高血压患病率是 BMI 在 24 以下者的 2.5 倍,BMI≥28 者的高血压患病率是 BMI 在 24 以下者的 3.3 倍。男性腰围达到或超过 85cm，女性腰围达到或超过 80cm，其高血压患病率是腰围正常者的 2.3 倍。一些减轻体重的实验表明，经减重治疗后，收缩压和舒张压也随平均体重的下降而降低。超重和肥胖引发高血压的机制可能与胰岛素抵抗代谢综合征有关。

1.3.2 型糖尿病

体重超重、肥胖和腹部脂肪蓄积是 2 型糖尿病发病的重要危险因素。我国 24 万人群数据的汇总分析显示，如以空腹血糖≥126 毫克/100 毫升或餐后 2 小时血糖仍≥200 毫克/100 毫升者诊断为 2 型糖尿病患者,BMI≥24 者的 2 型糖尿病的患病率为 BMI 在 24 以下者的 2.0

倍，BMI≥28 者的 2 型糖尿病患病率为 BMI 在 24 以下 者的 3.0 倍。男性和女性腰围分别为≥85cm 和≥80cm 时，糖尿病的患病率分别为腰围正常者的 2-2.5 倍。

1.3.3 血脂异常

我国 24 万人群数据的汇总分析显示，BMI≥24 者的血脂异常(甘油三酯≥200 毫克/100 毫升)检出率为 BMI 在 24 以下者的 2.5 倍,BMI≥28 者的血脂异常检出率为 BMI 在 24 以下 者的 3.0 倍，腰围超标者高甘油三酯血症的检出率为腰围正常者的 2.5 倍。BMI≥24 和≥28 者的高密度脂蛋白胆固醇降低(<35 毫克/100 毫升)的检出率分别为 BMI 在 24 以下者的 1.8 倍和 2.1 倍。腰围超标者高密度脂脂蛋白胆固醇降低的检出率为腰围正常者的 1.8 倍。

1.3.4 冠心病和其它动脉粥样硬化性疾病

我国 10 个人群的前瞻性研究显示，体重指数增高是冠心病发病的独立危险因素，冠心病事件(指急性心肌梗塞，冠心病猝死和其他冠心病死亡)的发病率随体重指数的上升而增高。前述的高血压，糖尿病和血脂异常都是冠心病和其他动脉粥样硬化性疾病的重要危险因素，而超重和肥胖导致这些危险因素聚集，大大促进了动脉粥样硬化的形成。BMI≥24 和 BMI≥28 的个体，有 2 个及以上危险因素聚集者动脉粥样硬化的患病率分别为 BMI 在 24 以下者的 2.2 和 2.8 倍。腰围超标危险因素聚集者的患病率为腰围正常者的 2.1 倍。表明超重肥胖是促进动脉粥样硬化的重要因素之一。

1.3.5 睡眠呼吸暂停症

肥胖引起睡眠中呼吸暂停，是由于在脖颈、胸部、腹部和横膈部位的脂肪堆积过多，使胸壁的运动受阻，在躺下时上呼吸道变窄和气流不通畅引起呼吸困难。因血液二氧化碳浓度过高和血氧低可抑制呼吸中枢，出现暂时窒息现象。如伴有严重呼吸道疾病，则容易产生肺动脉高压、心脏扩大和心力衰竭等。

1.3.6 内分泌及代谢紊乱

脂肪细胞不仅仅储存脂肪，还具有内分泌功能，同时也是许多激素作用的靶器官。肥胖者血浆中胰岛素明显高于正常水平，并经常存在胰岛素抵抗，中心性肥胖患者的激素水平改变更大。肥胖者血循环中的性激素平衡被破坏，尤其是腹部脂肪过多的女性常有排卵异常、雄激素过多，往往伴有生殖功能障碍。有的中度肥胖妇女发生多囊性卵巢综合征。体力活动常常能通过减轻体重而提高机体对胰岛素的敏感性。

1.3.7 超重和肥胖导致的社会和心理问题

由于文化背景、种族等的差异，人们对肥胖的态度不同，例如在经济不发达时期，我国曾把肥胖称为"发福"并作为富裕的象征。在发达国家和迅速发展的国家中，肥胖者必须与来自社会和环境的偏见和歧视作斗争。肥胖者也往往受社会观点、新闻媒介宣传的影响，对自身的体形不满，总认为在社交中会受到排斥，尤其在受到中、高等教育的年轻女性中易受这种心理屈使，把减肥作为时尚；往往出现体重处于正常范围的人还在奋力减重的现象，有人甚至因此导致厌食症。从小就发胖的儿童容易产生自卑感，对各种社交活动产生畏惧而不愿积极参与，造成心理问题。

暴饮暴食是肥胖患者中常见的一种心理病态行为。其主要特点是常常出现无法控制的食欲亢进，大多发生于傍晚或夜间，在夜里醒来后想吃东西。越来越多的观察发现，饮食习惯不良有时与肥胖患者的节食行为有关，如在上顿少吃或不吃后下顿大量进食的现象，严重影响治疗效果。还有人为了怕发胖，在大量进食美餐后自行引吐，这些与肥胖相伴的心理变化都有害于身心健康。

1.4 运动与减肥的生物学分析[43]

1.4.1 运动对脂肪体积的影响

运动以通过增可加能量消耗减少体内脂肪的积蓄。能量的消耗不足和能量代谢缺陷可能是某些肥胖发生和持续肥胖的基础。近年来，Applegate 等人对动物的实验研究发现，在以有氧运动减体脂过程中，虽不能减少脂肪细胞数目，但可以抑制脂肪细胞的积累，减小脂肪细胞体积。同时，有氧运动通过增加能量消耗减低了摄食效率，也减少了体脂沉积。

运动降低体脂含量，增加能量消耗，可通过调节内分泌代谢来实现，其中以对胰岛素作用的影响最为显著。首先，运动可改善肥胖者胰岛素受体结合力的降低。肥胖时首先出现骨骼肌胰岛素受体结合力下降，发生胰岛素抵抗，使能量储存转向脂肪组织，大量的葡萄糖进入脂肪组织，能量以脂肪形式储存。而运动可以逆转肌细胞膜胰岛素受体结合力的下降。其次，运动还可改善肥胖者胰岛素的敏感性.一般认为,运动通过增加能量消耗，造成体内能量负平衡，而达到减体脂的目的。一些学者通过运动对胰岛素作用影响的研究观察到，运动可增加胰岛素的敏感性。从而有效地调节体成分，达到减体脂的目的。

运动能增加热能消耗是毫无疑问的。一般情况下，即使轻微的体力活动也能使机体多消

耗10%-20%的能量。机体运动时，交感神经兴奋和血浆中抗胰岛素如儿茶芬胺、胰高血糖素、生长素、糖皮质激素等的浓度升高，抑制了胰岛素分泌。所以随着运动强度增大及运动时间延长，血浆胰岛素浓度趋于下降。运动引起儿茶芬胺和肾上腺皮质激素分泌增加，胰岛素分泌减少，促使脂肪水解过程的限速酶、甘油三a酶、细胞色素C氧化酶及柠檬酸合成酶活性增加，这些酶与脂肪的摄取、活化和动用有关，酶活性的提高会加速脂肪的水解。因此，运动能促进脂肪分解。

1.4.2 运动与糖和蛋白质

肌肉运动能增加人体对糖和蛋白质的利用.防止多余的糖和蛋白质转化为脂肪，减少脂肪的形成.糖是人体最便捷最有效的能源物质，运动能消耗摄入的糖和储备糖，阻止多余的糖向脂肪的转化.肌肉运动还能增强肌肉组织中蛋白质的新陈代谢，使肌细胞的代谢能力增强，增加肌纤维，减少脂肪储存.

1.4.3 运动与酶调节

运动能调节酶的活性，使一些酶的活性升高.如有氧耐力训练可提高骨胳肌中线粒体酶的活性，参与三梭酸循环的酶及呼吸链的氧化酶类的活性提高，从而保证长时间运动，保持良好的有氧代谢能力，促进糖特别是脂肪等物质的有氧氧化过程.

1.4.4 运动与瘦素

运动能影响体内血瘦素的水平.长期运动能调低人体瘦素水平，瘦素水平的变化与体脂的变化成正比例.运动还能影响脂肪细胞0b基因的表达,减少脂肪细胞0b基因表达leptin，改善肥胖患者的瘦素的抵抗. Kohrt研究表明，60-72岁健康老年女性受试者先进行2个月柔韧性训练，接着9个月行走、慢跑、爬楼梯等有氧训练，结果血Leptin水平下降，且与体脂量减少呈密切的关系(R为0.55,P<0.01)。

1.5 青少年单纯性肥胖定义和引起肥胖的因素

青少年单纯性肥胖症(Children Simple Obesity, CSO)已经成为一个世界性的问题，不但发达国家儿童、青少年肥胖现象普遍存在，发展中国家的发病率也在逐年增加。从近十年来的发病率来看，美国CSO的发病率为5-10%，我国大中城市CSO的发病率已接近发达国家水平。单纯性肥胖将是21世纪儿童、青少年期的一个重要健康问题。青少年单纯性肥胖症已被公认为是一种疾病，统计资料显示，在许多国家，近10年来小儿肥胖症的发病率升高了50%以上。现已证实，小儿肥胖，尤其是青春期肥胖者中有80%的人数将延续成为成人肥胖，而成人肥胖的治疗非常因难[25]。

1.5.1 青少年单纯性肥胖的定义和诊断标准

年龄在 0-18 岁(包括儿童、青少年和少年)，在研究的青少年肥胖中均排除了有染色体异常、内分泌紊乱、脑外伤、及肾脏疾病等既往病史的继发性肥胖少年，研究对象均属于青少年单纯性肥胖[33]

儿童、青少年单纯性肥胖诊断方法很多，彼此相关，但结果并不一致。例如:按年龄制定的标准体重，并没有充分考虑到同年龄身高影响。一些方法考虑到体重和身高两个因素的影响，但应用起来很不方便。特别是诊断标准极不统一。相关研究结果不便于比较。The International Obesity TaskForce (IOTF)2000 年儿童、青少年少年肥胖标准，根据年龄段划分成不同的标准。(见附件 1)，以体重指数 BMI 做为人群调查评价儿童、青少年肥胖的方法，其公式为 BMI]体重[kg]/身高[m]^2。消除了身高对体重的影响。应用起来比较方便 因此，认为 BMI 可以较合理地评价儿童、青少年青少年的体格发育和肥胖程度。2004 年，中国肥胖问题工作组根据对中国汉族 7—18 岁中小学生 244200 人的调查分析，得出中国学龄儿童、青少年超重、肥胖新标准，见表 3。考虑近年来中国学龄儿童、青少年生长加速趋势，缩短了和国际标准，又具有中国特点，体现东亚人群种族特征。

表 3中国学龄儿童、青少年青少年超重、肥胖 BMI 分类标准

年龄（岁）	男超重	男肥胖	女超重	女肥胖
7—	17.4	19.2	17.2	18.9
8—	18.1	20.3	18.1	19.9
9—	18.9	21.4	19.0	21.0
10—	19.6	22.5	20.0	22.1
11—	20.3	23.6	21.1	23.3
12—	21.0	24.7	21.9	24.5
13—	21.9	25.7	22.6	25.6
14—	22.6	26.4	23.0	26.3
15—	23.1	26.9	23.4	26.9
16—	23.5	27.4	23.7	27.4
17—	23.8	27.8	23.8	27.7
18	24	28.0	24.0	28.0

1.5.2 儿童、青少年青少年致肥胖因素的研究

(一)与饮食习惯的关系

肥胖的发生是复杂的，即有环境因素[外因型]又有遗传因素[基因内分泌型]的作用。研究表明，肥胖起源于能量摄取和能量消耗之间的不平衡，摄取能量超过消耗能量，剩余能量则以中性脂肪的形式主要储存在脂肪组织内。

肥胖儿童、青少年一般在饮食方面表现为自控性低，行为调节能力差的特点。自律性是指自我约束自己、控制自己、调节自己行为的能力。肥胖儿童、青少年有着特有的一种饮食方式，进食速度快，晚上进食，喜好油炸、糖类食品，同时过饮过食，对摄人没有节制性，这种过食行为，特别是摄入脂肪含量高的食品及碳水化合物，能引起丘脑下部的腹内侧核饱食中枢饱食感的阂值增高，而且过多的脂肪摄人更倾向在体内蓄积。

(二)与体育运动不足有关

现在孩子大部分是独生子女，家长溺爱，造成孩子依赖性强，自理能力差，活动量下降。他们由于体型的缘故，运动不甚灵活，从而比较懒惰，越懒越胖，越胖越懒，恶性循环。肥胖儿童、青少年喜睡，在体力、耐力、运动、灵敏度方面均低于正常儿童、青少年，使体能消耗少，摄取量与消耗量不平衡，营养物质转化成脂肪蓄积在体内。

(三)与社会因素的关系

由于近年来经济迅速发展，家庭生活富裕，不少孕妇从怀孕起即过度增加营养，造成新生儿肥胖，过渡至婴幼儿期肥胖;还由于缺乏正确的科学喂养知识及多年来社会上的旧观念认为孩子愈胖愈健康，唯恐孩子营养不足，强制或半强制地造成摄人过多，导致热量过剩[33]关于经济状态与肥胖关系，目前结论不致。美国许多肥胖来源于社会低薪阶层[34]。在新加坡资料表明，大多数肥胖来源于社会中上阶层[35]。

(四)与遗传因素有关

肥胖与遗传因素关系密切，肥胖儿童、青少年常有家族倾向性。据有关资料报道，双亲均为肥胖者，子女有 70%一 80%为肥胖者;双亲之一[特别是母亲]为肥胖者，子女有 40%为肥胖者;如果双亲均为非肥胖者，他们的子女只有 10%-14%为肥胖者。

Boron 用双生子法研究儿童、青少年肥胖的病因，其肥胖的遗传度为 0.88(P<0.01)[36]。20 世纪 60 年代人类发现了肥胖基因。Zhang 等[1994]用原位克隆分离出小鼠的肥胖基因(obese gene ob)。进而发现了 ob 编码的 mRNA 表达产物肥胖蛋白(obese protein op), Jeffrey Friedman 研究组成功克隆了肥胖基因，即 Leptin 的编码基因.其编码产物名为肥胖蛋白(Leptin)，也称 ” 瘦素 ”。它主要是通过其受体的直接结合来调节体内能量平衡，脂

肪蓄积及某些内分泌功能。人类肥胖基因普遍存在瘦素抵抗，例如:大多数肥胖者血中具有较高的瘦素水平，肥胖儿童、青少年较非肥胖儿童、青少年，女性较男性的瘦素水平都高37%。此外，近年还发现了其它与肥胖有关的基因及生物因子，如肿瘤坏死因子、白细胞介素、胰岛素样生长因子等。

1.5.3 肥胖对青少年的影响

(一)对身体素质的影响

肥胖少年由于体重负荷过重,心肺功能受限,在一定程度上影响了正常体质潜在的发挥。Schlicker 对美国儿童、青少年身体素质研究发现,肥胖少年的身体素质较一般青少年差[37]。季成叶等对我国 9 个省市区 11664 名 12 岁的小学生调查表明，肥胖学生的 50m 跑、立定跳远、斜身引体向上、仰卧起坐及往返跑等的成绩差生检出率明显高于非肥胖少年。另一研究提示，背肌力、握力等静力量可能未受损[38]。其原因可能是肥胖少年大脑接受外界信息与支配肢体反应的速度较慢，过多的脂肪使肌肉收缩时摩擦力增加，影响肌肉收缩的速度及爆发力;过重的体重影响身体运动的协调性和灵活性;肥胖少年的心肺功能较差，有氧能力下降，耐力水平必然受到影响[40]。有文章报道，肥胖对青少年身体危害主要包括 Pickwickian 综合征、心肌病、胰腺炎、外科异常(如变形性关节炎、膝内翻、髓内翻、扁平足、股骨头无菌性坏死)、呼吸系统异常(如上呼吸道阻塞、胸壁运动受限、睡眠中呼吸暂停综合征) [39]。

(二)肥胖对儿童、青少年行为的影响

Israeal 使用儿童青少年行为量表研究发现，肥胖儿童、青少年有更多的行为问题。在国内应用同一研究工具发现，肥胖儿童、青少年的社会适应能力、活动、社交及学校能力降低，在行为上常表现为内向、抑郁、焦虑及分裂样行为增多[41]。导致肥胖儿童、青少年心理行为改变的原因很复杂，由于肥胖儿童、青少年的体型臃肿，动作笨拙，运动能力较差，在集体活动中引人注目，常成为取笑的对象。

(三)肥胖对儿童、青少年智力与学业的影响

肥胖儿童、青少年的智力是否受到影响，报道结果不一。有研究发现，肥胖小学生的韦氏语言智商，操作智商及总智商均显著低于对照组，且 8 门课程中有 6 门课程成绩显著低于对照组[41]。另有研究，肥胖儿童、青少年不仅学习成绩差，且对自我学习状况，师生关系及同伴关系的满意度均较非肥胖儿童、青少年低[42]，提示肥胖儿童、青少年的学习能力较差。有学者认为，肥胖对儿童、青少年的智力发育的影响并不是由于肥胖导致的某种器质性疾病

所引起，而是由于肥胖儿童、青少年的精神负担加重，心理压力加大，不能专心于学业;同时，在学校中与同伴关系及师生关系不良，影响了情绪，造成学习情景不良，学习动机受损，影响其智力潜力的充分发挥而致。但也有报道肥胖儿童、青少年的智商与体重正常儿童、青少年差异无显著性。由于智力的内涵较广，智商和学习成绩只反映智力的某些方面，要对肥胖儿童、青少年智力作出正确评价，仍有待于进一步进行全面的研究。

1.6 肥胖儿童、青少年的运动处方

1.6.1 运动方式

对于肥胖儿童、青少年来说，运动方式的选择还应同时兼顾安全、经济和趣味性，使肥胖儿童、青少年愿意执行并能长期坚持。国外近期报导并认为在水中进行运动效果较好:水有浮力,使关节负担减轻;水中的静水压力作用于体表可使中心血容量增加:人在水中运动时体热容易消除;并且在水中的运动除游泳外，还可以在水中进行行走、跳跃、水中球类游戏等多种运动，趣味性较好[45]。但应注意安全和保护措施。

1.6.2 运动强度

一般以心率或氧消耗来衡量运动强度。 运动强度是运动处方定量化与科学性的核心问题，同时也是锻炼效果与安全性的关键，所以运动强度是设计运动处方中最困难的部分，也是争议最多的地方，因此各家学者意见并不完全一致[44, 46－48]。日本的池上晴夫教授认为，如果把有氧运动能力的增加作为运动效果指标，为了取得明显效果，必须用 Vo_2max 的 50%以上运动量;并认为 70%Vo2max 是最合适的界限，也是无氧性训练的阑值，这个界限以上的运动是危险的[47]。Wenger 等研究表明，当运动强度大于 90%Vo_2max 后，效果会因为疲劳而下降。但大多学者认为，低、中度有氧运动对减肥最好[44.46]。如以心率为指标则运动时应达到个人最大心率的 60% -85%[49]，如用氧消耗为指标，一般应取个人最大氧消耗的 50%-70%作为有氧运动的强度(50)一些研究提出单纯性肥胖儿童、青少年能承受较大强度的运动(最大 VO_2 的 60%一 80%),剧烈运动对儿童、青少年减肥起作用。Georgia 对 74 个 7- 9 岁的儿童、青少年进行了 4 个月的平均 5d/week, 40 min/次，157±7 beat/min(每次消耗能量约为 925 士 201 kJ)，训练后总的身体成分和心肺功能显著性提高，去脂体重显著增加。

由于心率可直接精确的反映心肌的工作强度，且心率容易测定和控制，有人指出用靶心率(THR)来控制运动强度较好[46]，THR 的测定可根据 Karvonen 公式: THR= (MHR－RHR) ×

50-85 %+RHR。(其中 MHR 为最高心率，RHR 为安静时心率。）但关于 THR 是否适用于儿童、青少年及我国各年龄儿童、青少年最高心率平均值的研究目前还较少。

1.6.3 运动频率

日本体育科学中心的一些专家认为,最标准的运动处方的理想频率是每周进行 3 次训练[48]。池上晴夫教授指出每周训练 1-2 次不仅不能取得良好的效果，而且由于身体不适应，外伤也多;而每天进行强烈运动也会有疲劳蓄积所致的坏影响[47]。大多学者认为锻炼者可根据需要、兴趣和功能状态选择适合自己情况的运动频率，但每周最低不能少于 2 次，以每周 3－5 次为适宜[45]。

1.6.4 运动持续时间和运动进度

关于每次运动持续的时间，大多学者认为应与运动强度联系起来一起考虑:即当运动强度较大时，持续时间可短一些，而当运动强度较小时，持续时间应长一些，以保证足够的运动量。对于不同的人群也应不同对待,Carl 等指出对心肺疾病患者持续时间可限定在 20 min,而对于肥胖者则持续时间最少应为 30 分钟或更长[46]。众多学者认为除准备活动和整理活动外，每次进行 20-60 分钟的有氧运动是比较适宜的。此外，由于机体的生物节律周期，参加同样的运动，下午与晚上比上午多消耗 20%的能量[45]。随着运动强度和时间的增加，运动效应逐渐出现，大部分参加者的运动效应是在运动的第 6-8 周时出[44]。医生、治疗师和健康指导者应根据参加者的特点(个体的最大功能、健康状况、年龄和目标)、新的运动实验和训练课中的运动能力，不断调整运动处方。

对于持续时间，一般认为一年或更长的时间要比短期有效，反弹的程度也较小。此外，10 年的跟综研究发现，体育活动对 6 岁的肥胖儿童、青少年比对 12 岁的肥胖儿童、青少年效果好。尽管这些研究有很大的差异性，但总起来说就是要求儿童、青少年多从事活动，少处于静止状态。运动干预对儿童、青少年肥胖的治疗研究结果的差异性是不是由于个体对运动的敏感性差异造成的，也就是说不同基因型的个体需要不同的运动负荷，这一方面的研究目前不明确。

由于肥胖儿童、青少年在肥胖程度、运动能力等方面个体差异较大，制定运动处方时应有针对性的调节运动强度和时间，并注意树立肥胖儿童、青少年完成运动的信心，培养他们逐渐形成良好的运动习惯，以达到减肥的效果，同时提高他们的身心健康水平。

1.7 运动对肥胖儿童、青少年体成分和身体素质的影响

1.7.1 运动对体脂的影响

有氧运动可以通过增加能量消耗减少体内脂肪的积蓄[53]。能量的消耗不足和能量代谢缺陷可能是某些肥胖发生和持续肥胖的基础。近年来，Applegate 等人对动物的实验研究发现，在以有氧运动减体脂过程中，虽不能减少脂肪细胞数目，但可以抑制脂肪细胞的积累，减小脂肪细胞体积。同时，有氧运动通过增加能量消耗减低了摄食效率，也减少了体脂沉积。

颜宜芭等[54]、Harsha Dwetat[55]的研究报道，有氧运动能使肥胖个体体重降低，肥胖指数下降。刘善云的研究表明[56]，长期有氧运动能提高机体对脂肪的利用率。于青梅[57]报道有氧运动能加速脂肪的动员速度，增加脂肪供能效率。王从容等[58]研究证实运动不仅能增加能量总消耗，还可增加安静代谢能量消耗[RMR]，一次运动后耗氧量可持续增高 48h。

王正珍等[52]研究发现以 60%-65%最大摄氧量作为运动强度经过 5 周的健身锻炼，少年的身体成分发生了明显变化，男、女生体重、脂肪体重和体重指数均有显著性变化。

Treuth 等人通过双标记水的方法研究提示，体脂成分的差异可能与体育活动消耗的能量有关[59]。体育活动与体脂的相关系数为:r=0.49，低水平体育活动的儿童、青少年三头肌皮下脂肪是积极参加体育活动的 3 倍多。还有研究指出，低的体育活动与肥胖的相对危险度为 2. 6，每天增加 1h 的看电视时间，青少年单纯性肥胖的危险度增加 12%，而每天增加 1 h 的体育活动，肥胖的危险度则降低 10%。

Georgia 对 74 个 7-9 岁的儿童、青少年进行了 4 个月的平均 5 d/week, 40 m in/次，157 士 7 beat/m in[每次消耗能量约为 925±201 kJ]，训练后总的身体成分和心肺功能显著性提高，与对照组相比，T FM, BF%, SAAT 都显著性降低，去脂体重显著增加，VAT 在训练组增加的比对照组显著少。Scottow ns 对 81 个 7- 11 岁的肥胖儿童、青少年进行同样的运动训练后也发现 BF%, TFM, VAT，都显著性降低，FFM 增加，有氧能力也显著性增加[63]。Gutin 的研究还证实了骨密度也有提高。而另一些研究却指出，在最大 VO2 的 40%最为合适，Ladda 的研究也支持这种观点, 并且他指出运动对男童体脂的改变高于女童。

1.7.2 运动与腰臀比

腰臀比（WHR）反映了肥胖的类型，它与很多疾病的关系非常密切。有研究发现[60]WHR 与血糖水平呈正相关关系，提随着 WHR 的增加 TGT、MD 患病率呈上升趋势。BMI, WHR 与血

压、血糖水平呈正相关关系。大量研究证实肥胖易引起胰岛素抵抗、高胰岛素血症，从而导致高血压、高血脂、高血糖等“X综合症”。

张新华等研究[61]得出腰臀比[WHR]越大，其运动能力越差，当腰臀比>0.73时，腰臀比与运动能力呈负相关，由此可以看出身体较胖的人在减肥时不愿选择运动方式，总想以药物代替其它方式的减肥，可能与他们本身的运动能力有一定关系，由于腰围的增粗，给下肢工作带来了不便，实验中观察到，对腰臀比不正常的人，运动中心电图没有发生任何改变，但已感到胸闷气短，不能坚持运动。实验结果锻炼可改善女学生腰臀比，减少腰腹部的脂肪堆积，增强心功能，增强运动能力。

杜熙茹对学龄儿童、青少年实验干预发现[62]，锻炼中基本部分心率在 140—160beat/min，每次运动时间为45 min，每周3次安排在下午课后进行，持续10周。结果表明:实验前后男女受试者的体重变化不明显。男女受试者实验后腰围、臀围值、腰臀比和体脂百分比的下降非常显著，BMI亦显著下降，瘦体重增加。

1.7.3 运动对肥胖儿童、青少年身体素质的影响

有氧运动除了可以减轻体重，还可以提高肥胖儿童、青少年身体谐调性，灵敏性，耐力等身体素质。龚贞观等研究[51]发现，初中儿童、青少年进行运动强度为最大心率的 50－60%锻炼，每10天为一周期，周期之间安排一次调整共三个周期后，实验结果7人平均体重减少1.75千克，最多的减少5千克。身体素质也得到了提高。1000米跑平均提高了37秒，立定跳远平均提高9.8厘米，仰卧起坐平均提高4.7个。通过实验得知:强化训练、兴趣培养和习惯养成是运动减肥不可缺少的三个部分。只有三方面有机结合才能取得良好的效果。

王正珍等[52]研究中选取超重和肥胖为对象，以60%一65%最大摄氧量作为运动强度，用相对应的心率作为靶心率来控制运动强度 经过5周每天2小时的健身锻炼，大多数超重肥胖少年的身体素质有明显提高，仰卧起坐、立卧撑、立定跳远、800米疾走、50米跑、女子10×4往返跑、女子握力成绩均有非常显著性提高(P<0.01)，男子10 × 4往返跑、男子50米跑成绩有显著性提高(P<0.05)。

综上所述,肥胖问题在现代社会逐渐受到重视和关注,肥胖严重危害了人们的身体健康.随着社会的发展,青少年单纯性肥胖症的发病率越来越高,而其中大多数将延续成为成人肥胖，本文就少年时期如何减肥问题做一探讨，并且通过对肥胖少年运动干预前后体成分、脂肪分布、身体素质、运动能力的变化，分析对单纯性肥胖少年运动干预的合理有效手段。

2 研究目的

随着我国经济的发展，人民生活水平的日益提高，青少年肥胖症在我国的发生率也越来越高，但目前对肥胖少年体成分运动干预情况的研究不多，本文拟通过有目的健身锻炼研究有氧锻炼对肥胖少年体成分、身体素质的影响。研究目的为:①观察分析肥胖少年体成分、脂肪分布以及实施运动处方后的干预情况②通过对肥胖少年运动干预前后身体素质的变化，分析对单纯性肥胖少年运动干预的有效手段。③促进生长发育,特别是线性发育，减少脂肪体重，增重速率在正常范围内增强体质，提高体育成绩合格。远期目标是培养科学合理的生活锻炼方式。

3 研究对象和研究方法

3.1 研究对象和分组

受试者为北京市海淀区某中学学生，年龄 12-14 岁，目测法初步筛选研究对象，然后对其身高和体重进行测试，以体重指数 BMI 为依据，根据 the International Obesity Task Force(IOTF)2000 年的标准（见附件 2）选出肥胖少年，然后进行调查以了解其生活方式和个人行为模式，从而排除某些内分泌、代谢、遗传、中枢神经系统疾病引起的继发性肥胖或因使用药物所诱发的肥胖，最终确定单纯性肥胖少年共 45 名，以自愿报名的形式分为肥胖运动组（obesity exercise，OE)。肥胖对照组（obesity control，OC)，基本情况见表 4。

表 4　实验组和对照组受试者基本情况

	男生		女生	
	运动组（n＝17）	对照组（n＝9）	运动组（n＝10）	对照组（n＝9）
年龄（year）	13.45±0.39	13.56±0.73	13.58±0.32	13.78±0.44
身高（cm）	167.07±8.28	168.23±10.25	162.10±7.71	164.13±4.36
体重（kg）	80.19±11.15	89.16±7.83	70.03±11.52	74.94±10.12
BMI（kg/m^2）	28.75±3.81	31.54±3.00	26.49±2.68	27.80±3.01
体脂（%）	34.71±4.79	35.44±7.45	35.14±2.76	36.61±2.11

3.2 研究方法

3.2.1 文献资料法

查阅国内外与本研究有关肥胖少年体育锻炼研究的文献资料，收集关于健身理论最新成果。

3.2.2 实验法

3.2.2.1 准备工作

在学生家长中发放问卷，收集有关生活方式和体育锻炼的基本信息 ，召开家长会,发放告家长信，以取得家长的支持和协助。

3.2.2.2 测试

运动处方实施前后,在相同实验条件下（相同测试人员，相同测试仪器，相同测试时间）对受试者进行两次测定。

锻炼前第一次测试 → 锻炼 12 周后 → 锻炼后第二次测试

①形态指标测试：

身高、体重、体成分、胸围、腰围、臀围、皮褶厚度。

体成分：使用韩国产 Inbody 3.0 电阻抗体成分仪 ，测空腹时体成分。

腰围：带尺经脐上 0.5-1 厘米处（肥胖者可选在腰部最粗处）。

臀围：受臀大肌最粗处将带尺沿水平位经后方绕至前方读数。

胸围：带尺上缘经背部肩胛下角下缘围向胸前乳头上缘，对已发育的女性，带尺经第四胸肋关节处

皮褶厚度：共测试三次，取中间值或两次相同的值。测试部位：上臂部皮褶厚度、肩胛下角皮褶厚度、腹部皮褶厚度。

②身体素质测试:

采用国家体育总局规定的体质监测器材，根据 2000 年国民体质监测工作要求， 测试指标：握力、背力、纵跳、坐位体前屈。

③心肺功能指标:

最大摄氧量（通过递增运动负荷实验测量)、肺活量、心率、血压。

④运动能力测试：

横跨（在平坦的地面上画 3 条平行直线，每条间隔 1 米。测试开始前，受试者两脚分立于中线两侧，微屈膝，听到开始信号后，向左或向右跨步，使两脚分跨在端线两边，然后再回到中线；再以同样动作，向另一端线跨步，再回到中线原来位置时为完成一次动作)。

50 米跑、立卧撑

3.3 运动处方制订与实施

运动组实施 12 周的运动处方。对照组不参加体育锻炼。通过递增负荷实验,测试每个受试者不同的最大摄氧量,计算出每个受试对象在规定运动强度时的心率，从而制定个性化运动处方。

① 锻炼时间:

总共 1 个小时，每次运动分 3 个阶段，即准备活动 5-10 分钟、训练 40-50 分钟、整理

活动 5-10 分钟。

② 运动方式:

根据少年的特点，运动方式多样化，包括耐力训练、力量训练、柔韧训练、灵敏谐调性训练、体育游戏等等。每进行 15-30 分钟练习，休息 2-3 分钟。

③ 运动强度:

运动强度取 60%-65%VO2max，通过 PACCER RS-232 跑台和美国产 PHYSIO-DYNEmax-II 自动气体分析仪直接测定每个受试者的最大摄氧量（VO_2max），用选取的运动强度相对应的心率作为靶心率控制每个受试者锻炼时的运动强度。注意个体差异性。在运动处方实施过程中定期对每个受试者进行运动强度监测(通过心率遥测仪对受试者运动中的心率进行测定)，已随时调整运动量从而保证每个受试者在以既定的运动强度运动。

④运动频率:

每周运动 4～5 次。肥胖运动组除去体育课外，放学后集中在学校操场上或体育馆内进行体育锻炼。肥胖对照组除去体育课并不进行运动训练。每周末组织肥胖实验组一次登山，游泳，定向等户外体育锻炼，以保证同学们的运动锻炼次数以及提高运动积极性。

⑤注意事项

锻炼过程中加强医务监督，观察受试者的主客观反应，出现异常时及时停止运动。

饮食方面：给学生家长发放饮食注意事项通知，锻炼后注意平衡膳食，减少含糖饮料，油炸食品的摄入。

3.4 数据处理

各指标测定值以“平均数±标准差”（$\overline{X}\pm SD$）表示。所有统计学处 Microsoft Excel 2000 统计软件上完成。显著性水平取 0.05，非常显著性水平取 0.01。

4 研究结果

4.1 运动处方实施前后皮褶厚度和围度的变化

表 5　运动组男生皮褶厚度变化

测试指标	锻炼前	锻炼后
上臂皮褶厚度（mm）	26.90±5.52	24.07±5.87*
背部皮褶厚度（mm）	30.80±8.89	27.41±7.97**
腹部皮褶厚度（mm）	43.03±9.30	41.80±8.83

注　**：锻炼前与锻炼后比较，P＜0.01

*：锻炼前与锻炼后比较，P＜0.05

如图所示，肥胖运动组男生经过 12 周锻炼后上臂皮褶厚度减少，从 26.90±5.52 减少到 24.07±5.87，减少了 10.5%,具有显著性差异(P＜0.05)， 背部皮褶厚度也减少，从 30.80±8.89 减少到 27.41±7.97，减少了 11%，具有非常显著性差异(P＜0.01)，腹部皮褶厚度 12 周前后变化无显著性差异(P＞0.05)。

表 6　对照组男生身体皮褶厚度变化

测试指标	实验前	实验后
上臂皮褶厚度（mm）	29.02±7.20	33.33±8.50*
背部皮褶厚度（mm）	37.06±9.43	38.17±10.14
腹部皮褶厚度（mm）	40.83±3.39	46.06±3.97**

注　**：锻炼前与锻炼后比较， P＜0.01 *：锻炼前与锻炼后比较， P＜0.05

如图所示，肥胖对照组男生没有参加锻炼，12 周后上臂皮褶厚度增加，从 29.02±7.20 减少到 33.33±8.50，增加了 14.9%,具有显著性差异(P＜0.05)， 腹部皮褶厚度也增加，从 40.83±3.39 增加到 56.06±3.97，增加了 37.3%，具有非常显著性差异(P＜0.01)，背部皮褶厚度 12 周前后无显著性差异(P＞0.05)。

表 7　运动组男生和对照组男生 12 周前后皮褶变化比较

测试指标	12 周前		12 周后	
	运动组	对照组	运动组	对照组
上臂皮褶（mm）	26.90±5.52	29.02±7.20	24.07±5.87	33.33±8.50*
背部皮褶（mm）	30.80±8.89	37.06±9.43	27.41±7.97	38.17±10.14*
腹部皮褶（mm）	43.03±9.30	40.83±3.39	41.80±8.83	46.06±3.97*

注 ** 运动组与对照组比较，P＜0.01

*：运动组与对照组比较， P＜0.05

如图所示，12 周前运动组和对照组男生皮褶厚度没有显著性差异，12 周后，运动组和对照组上臂、背部、腹部的皮褶出现显著性差异（P＜0.05）。

表8　运动组女生皮褶厚度变化

测试指标	锻炼前	锻炼后
上臂皮褶厚度（mm）	27.56±5.15	24.80±4.66*
背部皮褶厚度（mm）	30.11±7.55	27.62±6.02
腹部皮褶厚度（mm）	37.29±7.16	33.90±5.49*

注　**：锻炼前与锻炼后比较， P＜0.01

*：锻炼前与锻炼后比较， P＜0.05

如图所示，肥胖运动组女生经过 12 周锻炼后上臂皮褶厚度减少，从 27.56±5.15 减少到 24.80±4.66，减少了 10%,具有显著性差异(P＜0.05)， 腹部皮褶厚度从 37.29±7.16 减少到 33.90±5.49，减少了 9.1%，具有显著性差异(P＜0.05),背部皮褶厚度 12 周前后变化无显著性差异(P＞0.05)。

表 9　对照组女生皮褶厚度变化

测试指标	实验前	实验后
上臂皮褶厚度（mm）	27.00±2.78	30.44±3.36**
背部皮褶厚度（mm）	26.78±3.46	31.56±3.43**
腹部皮褶厚度（mm）	34.61±6.21	41.67±5.72**

注　**：锻炼前与锻炼后比较， P＜0.01

*：锻炼前与锻炼后比较，　P＜0.05

如图所示，肥胖对照组女生没有参加锻炼，12 周后上臂皮褶厚度增加，从 27.00±2.78 减少到 30.44±3.36，增加了 12.7%,具有显著性差异(P<0.01)。 背部皮褶厚度也增加，从 26.78±3.46 增加到 31.56±3.43，增加了 17.8%，具有非常显著性差异(P<0.01)。腹部皮褶厚度也增加，从 34.61±6.21 增加到 41.67±5.72，增加了 20.4%，具有非常显著性差异(P<0.01)。

表10　运动组女生和对照组女生12周前后皮褶变化比较

测试指标	12周前		12周后	
	运动组	对照组	运动组	对照组
上臂皮褶（mm）	27.56±5.15	27.00±2.78	24.80±4.66	30.44±3.36*
背部皮褶（mm）	30.11±7.55	26.78±3.46	27.62±6.02	31.56±3.43*
腹部皮褶（mm）	37.29±7.16	34.61±6.21	33.90±5.49	41.67±5.72**

注 ** 运动组与对照组比较 ， P＜0.01

*：运动组与对照组比较 ， P＜0.05

如图所示，12周前运动组和对照组女生皮褶厚度没有显著性差异，12周后运动组和对照组上臂、背部皮褶出现显著性差异（P＜0.05），腹部皮褶厚度两组之间出现非常显著性差异（P＜0.01）。

表11　运动组男生围度变化

测试指标	锻炼前	锻炼后
胸围（cm）	98.83±5.44	99.17±5.31
腰围（cm）	93.83±7.02	94.90±7.40
臀围（cm）	100.43±6.86	102.63±6.17 **
腰臀比	0.94±0.05	0.93±0.06

注 ** ：锻炼前与锻炼后比较， P＜0.01

*：锻炼前与锻炼后比较， P＜0.05

如图所示，肥胖运动组男生经过三个锻炼月后，随着身体的发育臀围增加，从100.43±6.86增加到102.63±6.17 ， 胸围、腰围、腰臀比在实验前后变化无显著性差异(P＞0.05)。

表 12　对照组男生围度变化

测试指标	实验前	实验后
胸围（cm）	104.89±4.34	106.61±4.29
腰围（cm）	101.90±6.26	103.67±7.14
臀围（cm）	109.91±4.83	112.17±4.86**
腰臀比	0.93±0.05	0.92±0.05

注 ** ：锻炼前与锻炼后比较， P＜0.01

*：锻炼前与锻炼后比较， P＜0.05

如图所示，肥胖对照组男生没有参加锻炼，12 周后臀围增加，从 109.91±4.83 增加到 112.17±4.86,具有非常显著性差异(P＜0.01), 胸围、腰围、腰臀比在实验前后变化无显著性差异(P＞0.05)。

表 13　运动组男生和对照组男生 12 周前后围度变化比较

测试指标	12 周前		12 周后	
	运动组	对照组	运动组	对照组
胸围（cm）	98.83±5.44	104.89±4.34	99.17±5.31	106.61±4.29
腰围（cm）	93.83±7.02	101.90±6.26	94.90±7.40	103.67±7.14
臀围（cm）	100.43±6.86	109.91±4.83	102.63±6.17	112.17±4.86
腰臀比	0.94±0.05	0.93±0.05	0.93±0.06	0.92±0.05

注 ** 运动组与对照组比较， P＜0.01；*：运动组与对照组比较， P＜0.05

如图所示，12 周前后，男生运动组和对照组的围度变化都没有没有显著性差异。

表 14　运动组女生围度变化

测试指标	锻炼前	锻炼后
胸围（cm）	95.30±7.75	95.85±6.86
腰围（cm）	84.05±4.30	81.45±6.49 *
臀围（cm）	100.12±7.48	99.85±7.03
腰臀比（cm）	0.84±0.05	0.81±0.03 *

注　**：锻炼前与锻炼后比较， P＜0.01；*：锻炼前与锻炼后比较， P＜0.05

如图所示,肥胖运动组女生经过三个锻炼月后腰围减小,从 84.05±4.30 到 81.45±6.49,减小了 3.1%,具有显著性差异(P＜0.05)。腰臀比减小,从 0.84±0.05 到 0.81±0.03,减小了 3.6%,具有显著性差异(P＜0.05)。安静胸围、臀围在实验前后变化无显著性差异(P＞0.05)。

表 15　肥胖对照组女生围度变化

测试指标	实验前	实验后
胸围（cm）	94.61±7.14	99.00±8.20**
腰围（cm）	85.61±8.78	86.52±7.15
臀围（cm）	102.97±6.21	105.00±7.66*
腰臀比（cm）	0.83±0.06	0.83±0.05

注　**：锻炼前与锻炼后比较， P＜0.01；*：锻炼前与锻炼后比较， P＜0.05

如图所示，肥胖对照组女生没有参加锻炼，12 周后安静胸围增加，从 94.61±7.14 增加到 99.00±8.20，增加了 4.6％，具有非常显著性差异(P＜0.01)。臀围增加，从 102.97±6.21 到 105.00±7.66，具增加了 2%，有显著性差异(P<0.05)。腰围、腰臀比在实验前后变化无显著性差异(P>0.05)。

表 16　运动组女生和对照组女生 12 周前后围度变化比较

测试指标	12 周前		12 周后	
	运动组	对照组	运动组	对照组
胸围（cm）	95.30±7.75	94.61±7.14	95.85±6.86	99.00±8.20*
腰围（cm）	84.05±4.30	85.61±8.78	81.45±6.49	86.52±7.15*
臀围（cm）	100.12±7.48	102.97±6.21	99.85±7.03	105.00±7.66*
腰臀比	0.84±0.05	0.83±0.06	0.81±0.03	0.83±0.05 *

注 ** 运动组与对照组比较， P＜0.01；*：运动组与对照组比较， P＜0.05

如图所示， 12 周前女生运动组和对照组身体围度没有显著性差异，12 周后运动组和对照组胸围、腰围、臀围、腰臀比出现显著性差异（P＜0.05)。

4.2 运动处方实施前后身体成分的变化

表 17　肥胖运动组男生身体成分变化

测试指标	锻炼前	锻炼后
体重（kg）	80.19±11.15	80.64±11.26
脂肪重量（kg）	27.92±6.09	25.91±7.15**
体脂百分比（%）	34.71±4.79	31.86±5.93**
肌肉重量（kg）	48.90±7.09	51.24±6.98**
肌肉重/体重	0.61±0.05	0.64±0.06**
蛋白总量（kg）	10.42±1.50	10.89±1.50**
蛋白总量/体重	0.13±0.01	0.14±0.01**
无机盐（kg）	3.38±0.40	3.51±0.39**
无机盐/体重	0.0423±0.0029	0.0438±0.0036**
肥胖度（%）	135.07±19.48	131.20±18.73**
BMI（kg/㎡]	28.75±3.81	28.20±3.74*
BMR（kcal]	1912.81±223.29	1981.91±221.09**

注 **：锻炼前与锻炼后比较， P＜0.01；*：锻炼前与锻炼后比较， P＜0.05

如图所示，肥胖运动组男生经过 12 周锻炼后，肌肉重量、蛋白总量、无机盐、BMR 都增加，具有非常显著性差异(P<0.01)。脂肪重量、体脂百分比、肥胖度实验后比实验前减少，具有非常显著性差异(P<0.01)，BMI 比实验前减少了 1.7%，具有显著性差异(P<0.01)。体重在实验前后变化无显著性差异(P>0.05)。

表 18　肥胖对照组男生身体成分变化

测试指标	实验前	实验后
体重（kg）	89.16±7.83	92.68±9.01**
脂肪重量（kg）	31.32±6.05	33.98±6.89 **
体脂百分比（%）	35.44±7.45	36.89±7.68 *
肌肉重量（kg）	54.56 ±9.62	55.41±9.99
肌肉重/体重	0.61±0.07	0.60±0.07*
蛋白总量（kg）	13.76 ±2.18	13.98±2.22
蛋白总量/体重	0.154±0.016	0.151±0.017*
无机盐（kg）	3.24±0.66	3.29±0.69
无机盐/体重	0.036±0.006	0.035±0.005**
肥胖度（%）	147.44 ±18.49	153.22±19.18**
BMI（kg/㎡）	31.54±3.00	32.78±3.26**
BMR（kcal）	2061.92±285.42	2074.68±292.81

注　**：锻炼前与锻炼后比较，　P<0.01 *：锻炼前与锻炼后比较，　P<0.05

如图所示，肥胖对照组男生没有参加锻炼，12 周后体重、脂肪重量、肥胖度、BMI 都增加，具有非常显著性差异(P<0.01=。同时，体脂百分比也增加，具有显著性差异(P<0.05=。肌肉重/体重、蛋白总量/体重、无机盐/体重实验后比实验前减少，具有显著性差异(P<0.05=。肌肉重量、蛋白总量、无机盐、BMR 在实验前后变化无显著性差异(P>0.05)。

表 19　　肥胖运动组女生身体成分变化

测试指标	锻炼前	锻炼后
体重（kg）	70.03±11.52	68.79±11.24**
脂肪重量（kg）	24.74±5.36	22.88±5.77**
体脂百分比（%）	35.14±2.76	32.94±3.69**
肌肉重量（kg）	42.28±6.37	42.90±6.03*
肌肉重/体重	0.61±0.03	0.63±0.03**
蛋白总量（kg）	9.01±1.33	9.12±1.25*
蛋白总量/体重	0.1290±0.01	0.1332±0.01**
无机盐（kg）	3.00±0.36	3.04±0.34*
无机盐/体重	0.0431±0.0024	0.0445±0.0030**
肥胖度（%）	126.60±11.27	123.00±12.03**
BMI（kg/㎡）	26.49±2.68	25.82±2.79**
BMR（kcal）	1697.49±202.37	1716.17±191.60*

注　**：锻炼前与锻炼后比较，　P＜0.01

　　*：锻炼前与锻炼后比较，　P＜0.05

如图所示，肥胖运动组女生经过 12 周锻炼后，肌肉重量、蛋白总量、无机盐、BMR 增加，具有显著性差异(P＜0.05)。脂肪重量、体脂百分比、肥胖度、BMI 实验后比实验前减少，具有非常显著性差异(P＜0.01)。

表 20　　肥胖对照组女生身体成分变化

测试指标	实验前	实验后
体重（kg）	74.94±10.12	75.91±12.17
脂肪重量（kg）	27.47±4.30	27.50±5.87
体脂百分比（%）	36.61±2.11	35.98±2.57
肌肉重量（kg）	44.60 ±6.26	45.51±6.64
肌肉重/体重	0.59±0.02	0.60±0.02
蛋白总量（kg）	10.71±2.69	10.91±2.80
蛋白总量/体重	0.141±0.02	0.142±0.017
无机盐（kg）	2.88±0.17	2.93±0.19
无机盐/体重	0.0389±0.0041	0.0392±0.0050
肥胖度（%）	131.78±13.44	132.89±17.62
BMI（kg/㎡）	27.80±3.01	28.07±3.84
BMR（kcal）	1762.37±209.29	1765.64±224.69

注 ** ：锻炼前与锻炼后比较 ， P＜0.01

*：锻炼前与锻炼后比较 ， P＜0.05

如图所示，肥胖对照组女生没有参加锻炼，12 周后各项体成分指标在实验前后变化无显著性差异(P＞0.05)。

表 21 运动组男生和对照组男生 12 周前后体成分变化比较

测试指标	12 周前		12 周后	
	运动组	对照组	运动组	对照组
体脂含量（%）	34.71±4.79	35.44±7.45	31.86±5.93	36.89±7.68 *
肌肉重/体重	0.61±0.05	0.61±0.07	0.64±0.06	0.60±0.07 **
无机盐/体重	0.042±0.0029	0.036±0.006	0.044±0.0036	0.036±0.005 *
肥胖度（%）	135.07±19.48	147.44 ±18.49	131.20±18.73	153.22±19.18
BMI（kg/m²）	28.75±3.81	31.54±3.00	28.20±3.74	32.78±3.26*

注 ** ：运动组与肥胖对照组比较 ， P＜0.01

*：运动组与对照组比较， P＜0.05

如图所示，12 周前运动组男生和对照组男生身体成分各项指标没有显著性差异。12 周后两组比较，肌肉/体重有显著性差异（P＜0.01），体脂含量、无机盐/体重、肥胖度、BMI 有显著性差异（P＜0.05）。

表 22运动组女生和对照组女生 12 周前后体成分变化比较

测试指标	12 周前		12 周后	
	运动组	对照组	运动组	对照组
体脂含量（%）	35.14±2.76	36.61±2.11	32.94±3.69	35.98±2.57 *
肌肉重/体重	0.61±0.03	0.59±0.02	0.63±0.03	0.60±0.02
无机盐/体重	0.042±0.0024	0.039±0.0041	0.045±0.003	0.039±0.005 *
肥胖度（%）	126.60±11.27	131.78±13.44	123.00±12.03	132.89±17.62*
BMI（kg/m²）	26.49±2.68	27.80±3.01	25.82±2.79	28.07±3.84*

注 ** ：运动组与对照组比较 ， P＜0.01

*：运动组与对照组比较， P＜0.05

如图所示，12 周前运动组女生和对照组女生身体成分各项指标没有显著性差异。12 周后两组比较，体脂含量、无机盐/体重、肥胖度、BMI 有显著性差异（P＜0.05）。肌肉

重/体重没有显著性差异（P＞0.05）。

4.3 运动处方实施前后身体素质的变化

表 23 肥胖运动组男生身体素质变化

测试指标	锻炼前	锻炼后
握力（kg）	29.03±8.62	32.15±8.38 *
背力（kg）	97.82±22.11	111.18±23.38**
纵跳（cm）	25.68±6.53	28.81±6.09 **
坐位体前屈（cm）	3.67±6.59	4.93±7.39*

注 ** ：锻炼前与锻炼后比较， P＜0.01； *：锻炼前与锻炼后比较， P＜0.05

如图所示，肥胖运动组男生经过 12 周锻炼后握力变大，从 29.03±8.62 增加到 32.15±8.38，具有显著性差异(P＜0.05)。柔韧程度改善，坐位体前屈从 3.67±6.59 增加到 4.93±7.39，具有显著性差异(P＜0.05)。背力增大，纵跳能力提高，具有非常显著性差异(P＜0.01)。

表 24 肥胖对照组男生身体素质变化

测试指标	实验前	实验后
握力（kg）	30.89±7.65	31.61±8.19*
背力（kg）	95.11±33.01	112.89±27.14*
纵跳（cm）	22.47±5.67	26.14±6.93*
坐位体前屈（cm）	4.34±9.05	7.26±9.90

注 ** ：锻炼前与锻炼后比较， P＜0.01 ；*：锻炼前与锻炼后比较， P＜0.05

如图所示，肥胖对照组男生没有参加锻炼，12 周后随着身体的发育，握力变大，从 30.89±7.65 增加到 36.61±8.19，具有显著性差异(P＜0.05＝。背力增大，从 95.11±33.01 增加到 112.89±27.14。，具有显著性差异(P＜0.05＝。纵跳能力提高，从 22.47±5.67 提高到 26.14±6.93，具有显著性差异(P＜0.05＝。坐位体前屈在实验前后变化无显著性差异(P＞0.05)。

表25　运动组男生和对照组男生12周前后身体素质变化比较

测试指标	12周前		12周后	
	运动组	对照组	运动组	对照组
握力（kg）	29.03±8.62	30.89±7.65	32.15±8.38	31.61±8.19*
背力（kg）	97.82±22.11	95.11±33.01	111.18±23.38	112.89±27.14*
纵跳（cm）	25.68±6.53	22.47±5.67	28.81±6.09	26.14±6.93*
坐位体前屈（cm）	3.67±6.59	4.34±9.05	4.93±7.39	7.26±9.90

注 ** 运动组与对照组比较， P＜0.01； *：运动组与对照组比较， P＜0.05

如图所示，12周前男生运动组和对照组身体素质各项指标没有显著性差异，12周后运动组和对照组握力、背力、纵跳出现显著性差异（P＜0.05)，坐位体前屈指标没有显著性差异(p＞0.05)。

表26　肥胖运动组女生身体素质变化

测试指标	锻炼前	锻炼后
握力（kg）	22.09±4.10	24.84±3.45*
背力（kg）	67.80±17.99	88.20±16.42**
纵跳（cm）	20.60±3.42	23.90±2.48**
坐位体前屈（cm）	7.53±9.51	14.54±4.89*

注　**：锻炼前与锻炼后比较， P＜0.01

*：锻炼前与锻炼后比较， P＜0.05

如图所示，肥胖运动组女生经过12周锻炼后握力变大，从22.09±4.10增加到24.84±3.45，具有显著性差异(P＜0.05)。柔韧程度改善，坐位体前屈从7.53±9.51增加到14.54±4.89，具有显著性差异(P＜0.05)。背力增大，纵跳能力提高，变化具有非常显著性差异(P＜0.01)。

表27　肥胖对照组女生身体素质变化

测试指标	实验前	实验后
握力（kg）	22.80±4.96	26.81±5.76 *
背力（kg）	73.44±16.90	69.89±23.49
纵跳（cm）	22.06±5.40	23.82±5.19
坐位体前屈（cm）	11.91±7.17	11.03±8.03

注　**：锻炼前与锻炼后比较， P＜0.01

*：锻炼前与锻炼后比较， P＜0.05

如图所示，肥胖对照组女生没有参加锻炼，12 周后随着身体的发育，握力变大，从 22.80±4.96 增加到 26.81±5.76，具有显著性差异(P＜0.05)。背力、纵跳、坐位体前屈在实验前后变化无显著性差异(P＞0.05)。

表 28　女生运动组和女生对照组 12 周前后身体素质变化比较

测试指标	12 周前		12 周后	
	运动组	对照组	运动组	对照组
握力（kg）	22.09±4.10	22.80±4.96	24.84±3.45	26.81±5.76
背力（kg）	67.80±17.99	73.44±16.90*	88.20±16.42	69.89±23.49*
纵跳（cm）	20.60±3.42	22.06±5.40	23.90±2.48	23.82±5.19
坐位体前屈（cm）	7.53±9.51	11.91±7.17	14.54±4.89	11.03±8.03*

注 ** 运动组与对照组比较 ， P＜0.01

*：运动组与对照组比较 ， P＜0.05

如图所示，12 周前运动组女生和对照组女生背力有显著性差异（P＜0.05），对照组女生背力偏大，其他身体素质各项指标没有显著性差异，12 周后运动组女生背力增加到 88.20±16.42，而对照组女生只有 69.89±23.49，12 周后两组背力、坐位体前屈有显著性差异（P＜0.05），握力、纵跳没有显著性差异(p＞0.05)。

4.4 运动处方实施前后运动能力的变化

表 29　肥胖运动组男生运动能力变化

测试指标	锻炼前	锻炼后
横跨（个/分）	29.31±2.70	32.00±2.92**
立卧撑（个/20 秒）	8.13 ±2.36	10.81±2.20**
50 米跑（秒）	9.13±1.27	8.67±1.23**

注 *：锻炼前与锻炼后比较， P＜0.05

** ：锻炼前与锻炼后比较， P＜0.01

如图所示，肥胖运动组男生经过 12 周锻炼后运动能力明显改善，各项测试指标都提高，并且实验前后变化具有非常显著性差异(P＜0.01)。

表 30　肥胖运动组女生运动能力变化

测试指标	锻炼前	锻炼后
横跨（个/分）	28.10±3.48	31.10±3.70*
立卧撑（个/20 秒）	8.70±2.06	9.10±1.29
50 米跑（分）	9.89±1.11	9.36±0.78*

注　**：锻炼前与锻炼后比较， P＜0.01；*　：锻炼前与锻炼后比较 ， P＜0.05

如图所示，肥胖运动组女生经过 12 周锻炼，50 米跑的速度加快，从原来的 9.89±1.11 提高到 9.36±0.78。身体灵敏谐调能力增强，12 周后，横跨从 28.10±3.48 增加到 31.10±3.70，变化具有显著性差异(P<0.05)。立卧撑变化具有没有显著性差异(P>0.05)。

5 分析讨论

5.1 运动对肥胖少年身体围度和皮褶厚度的变化

皮褶厚度的测量，是了解人体体成分（即体脂肪量、体脂百分比和瘦体重等）的一种简易方法[64]。人体过胖或过瘦，会给人的健康带来很大影响。现代社会的许多文明病，如高血压、心血管疾病、肥胖症和营养不良症等，都与人体内脂肪的含量和分布状态有密切的关系。

本实验结果，肥胖运动组男生经过12周锻炼后上臂皮褶厚度减少，从26.90±5.52减少到24.07±5.87，减少了10.5%,具有显著性差异（P<0.05）， 背部皮褶厚度也减少，从30.80±8.89 减少到 27.41±7.97，减少了 11%，具有非常显著性差异（P<0.01)，腹部皮褶厚度 12 周前后变化无显著性差异（P>0.05)。肥胖对照组男生没有参加锻炼，12 周后上臂皮褶厚度增加，从 29.02±7.20 增加到 33.33±8.50，增加了 14.9%,具有显著性差异（P<0.05 ）， 腹部皮褶厚度也增加，从 40.83±3.39 增加到 56.06±3.97，增加了 37.3%，具有非常显著性差异（P<0.01)，背部皮褶厚度 12 周前后无显著性差异（P>0.05)。 运动组男生和肥胖组男生之间进行比较分析，12 周前运动组和对照组男生皮褶厚度没有显著性差异，12 周后，运动组和对照组上臂、背部、腹部的皮褶出现显著性差异（P<0.05)。

肥胖运动组女生经过 12 周锻炼后上臂皮褶厚度减少，从 27.56±5.15 减少到24.80±4.66，减少了10%,具有显著性差异（P<0.05）， 腹部皮褶厚度从37.29±7.16减少到33.90±5.49，减少了 9.1%，具有显著性差异（P<0.05）,背部皮褶厚度12周前后变化无显著性差异（P＞0.05)。肥胖对照组女生没有参加锻炼，12 周后上臂皮褶厚度增加，从27.00±2.78增加到30.44±3.36，增加了12.7%,具有显著性差异（P<0.01)。 背部皮褶厚度也增加，从26.78±3.46增加到31.56±3.43，增加了17.8%，具有非常显著性差异（P<0.01)。腹部皮褶厚度也增加，从34.61±6.21增加到41.67±5.72，增加了20.4%，具有非常显著性差异(P<0.01)。运动组女生和肥胖组女生之间进行比较分析，12 周前运动组女生和对照组女生皮褶厚度没有显著性差异，12周后运动组上臂、背部、腹部皮褶减少，而对照组增加，分析得到运动组和对照组上臂、背部皮褶出现显著性差异（P<0.05)，腹部皮褶厚度两组之间出现非常显著性差异（P<0.01)。

综合上述分析，我们可以看出12周的有氧运动干预后，肥胖运动组的男生上臂、背部臂皮褶厚度减少，腹部皮褶厚度变化不明显，而肥胖对照组男生上臂、腹部皮褶厚度增加，背部皮褶厚度变化不大。通过两组之间的对比，结果表明12周后运动组和肥胖组之间，各

部位的皮褶厚度存在显著性差别（P<0.05)。肥胖运动组的女生上臂、腹部皮褶减少，背臂皮褶厚度厚度变化不明显。肥胖对照组女生上臂、背部皮褶厚度、腹部皮褶厚度都增加。运动组女生和肥胖组女生之间进行比较分析， 12 周前运动组和对照组女生皮褶厚度没有显著性差异，12 周后运动组和对照组上臂、背部皮褶出现显著性差异（P<0.05)，腹部皮褶厚度两组之间出现非常显著性差异（P<0.01)。

由此可见，12 周的运动处方致使男女肥胖少年皮褶厚度都有不同程度的减少， 女肥胖少年更着重于腹部皮褶脂肪的减少。

胸围是胸廓的最大围度，可以表示胸廓大小和肌肉发育状况，是人体宽度和厚度最有代表性的指标，在一定程度上反映身体形态和呼吸器官的发育状况，同时也是评价生长发育水平的重要指标。腰围在一定程度上反映腹部皮下脂肪厚度和营养状态，是间接反映人体脂肪状态的简易指标。同时，腰围的大小，不仅可以反映出体型特点，而且，保持腰围与臀围的适当比例关系，对体质和健康及其寿命有着重要意义。腰臀比，是腰围和臀围的比值，与身体健康和某些疾病的发生关系密切。腰臀比（WHR）反映了肥胖的类型，它与很多疾病的关系非常密切。有研究发现(60) WHR 与血糖水平呈正相关关系，提随着 WHR 的增加 TGT、MD 患病率呈上升趋势。BMI, WHR 与血压、血糖水平呈正相关关系。大量研究证实肥胖易引起胰岛素抵抗、高胰岛素血症，从而导致高血压、高血脂、高血糖等“ X 综合症 ”。男性腰围达到或超过 85 厘米，女性腰围达到或超过 80 厘米者患高血压的危险约为腰围低于此界限者的 3.5 倍，其患糖尿病的危险约为 2.5 倍；其中有 2 项及 2 项以上危险因素聚集者的危险约为正常体重者的 4 倍以上[28]。

本文实验结果表明肥胖运动组男生经过三个锻炼月后胸围、腰围、腰臀比在实验前后变化不大，臀围增加了 2.2%，具有非常显著性差异（P<0.01)。 肥胖对照组男生 12 周后胸围、腰围、腰臀实验前后无明显变化，而臀围也增加，也具有非常显著性差异（P<0.01)。运动组男生和对照组男生进行对比分析，发现 l2 周前后两组的围度变化都没有没有显著性差异(P>0.05)。肥胖运动组女生经过 12 周锻炼后，腰围减小，从 84.05±4.30 到 81.45±6.49，减小了 3.1%，具有显著性差异(P<0.05)。腰臀比减小，从 0.84±0.05 到 0.81±0.03，减小了 3.6%，具有显著性差异(P<0.05)。张新华等研究[61]结果显示锻炼可改善女学生腰臀比，减少腰腹部的脂肪堆积，与本试验结果吻合。肥胖对照组女生没有参加锻炼，12 周后胸围增加，从 94.61±7.14 增加到 99.00±8.20，增加了 4.6%，具有非常显著性差异(P<0.01)。臀围增加，从 102.97±6.21 到 105.00±7.66，具增加了 2%，有显著性差异(P<0.05)。腰围、腰臀比在实验前后变化无显著性差异(P>0.05)。运动组女生生和对照组女生进行对比分析发

现， 12 周前运动组女生和对照组女生身体围度没有显著性差异，而 12 周后两组胸围、腰围、臀围、腰臀比均出现显著性差异（P<0.05)。

可见，本实验通过 12 周的运动处方，肥胖运动组男生和肥胖对照组男生相比，胸围、腰围、腰臀比变化没有大的差异 ，两组研究对象由于身体青春期发育造成臀围都增加。而女生围度变化比较明显。12 周的有氧运动能显著减小女性肥胖少年腰围、腰臀比，但对男性肥胖少年围度影响不大。通过分析发现，男性肥胖少年通过 12 周的锻炼，上臂、背部、腹部皮褶厚度都显著减少（P<0.05)，但是身体的围度却没有显著性变化（P>0.05)，原因可能是处于青春突增期男性肥胖少年随着青春期身体的发育，骨骼、肌肉全身各器官都生长发育，但是由于 12 周的锻炼后男性肥胖少年皮褶显著减少，所以身体围度没有表现显著差异。

5.2 运动对肥胖少年身体成分的变化

组成人体各组织、器官的总成分，称身体成分，其总重量为体重。根据生理功效的不同，常把体重分为:脂肪重(体脂)和去脂体重(瘦体重)。在正常的情况下(成年人)，瘦体重是相对的恒定，而脂肪重是体重变化的主要因素，身体成分是判断肥胖的标准之一[65]。但对于正处在青春发育期的青少年来说，因为正处于发育阶段，所以盲目的进行节食减肥，不仅减肥后会反弹,而且会严重影响青少年正常的生理发育,正确的减肥方法不光要减去多余的脂肪，而且要促进肥胖少年的肌肉、骨骼的的发育，提高整体素质和机能。

在本实验里，肥胖运动组男生经过 12 周锻炼后，肌肉重量、蛋白总量、无机盐、BMR 都增加,具有非常显著性差异(P＜0.01)。脂肪重量、体脂百分比、肥胖度实验后比实验前减少，具有非常显著性差异(P＜0.01)，BMI 比实验前减少了 1.7%，具有显著性差异（ P＜0.05)。体重在实验前后变化无显著性差异(P＞0.05)。肥胖对照组男生 12 周后体重、脂肪重量、肥胖度、BMI 都增加,具有非常显著性差异(P＜0.01)。同时，体脂百分比也增加，具有显著性差异(P＜0.05)。肌肉重/体重、无机盐/体重实验后比实验前减少，具有显著性差异(P＜0.05)。肌肉重量、无机盐、BMR 在实验前后变化无显著性差异(P＞0.05)。

肥胖运动组女生经过 12 周锻炼后，肌肉重量、无机盐、BMR 增加,具有显著性差异(P＜0.05)。体重、脂肪重量、体脂百分比、肥胖度、BMI 实验后比实验前减少，具有非常显著性差异(P＜0.01)。 肥胖对照组女生没有参加锻炼，12 周后身高增加,具有显著性差异(P＜0.05)。其他各项体成分指标在实验前后变化无显著性差异(P＞0.05)。

通过数据分析得知肥胖实验组和肥胖对照组在实验前,身体成分各项指标没有显著性差异。通过 12 周的训练，肥胖运动组男生和女生肌肉重、无机盐，基础代谢率都增加，脂肪

重量、体脂百分比、BMI、肥胖度实验后都比实验前减少。锻炼后肥胖组运动与肥胖对照组差别较大，对照组男生没有参加锻炼，12 周后脂肪重量、体脂百分比、BMI、肥胖度实验后都比实验前增加。对照组女生指标变化不明显。12 周后男生运动组和对照组相比，肌肉/体重有非常显著性差异（P<0.01），体脂含量、无机盐/体重有显著性差异（P<0.05）， 12 周后女生运动组和对照组相比体脂含量、无机盐/体重有显著性差异（P<0.05）。肌肉重/体重没有显著性变化。 男女差别是：肥胖运动组男生更侧重于肌肉重量、蛋白质、无机盐的增长。而女性肥胖少年更侧重于脂肪的减少,肌肉增长不明显。

Arsha Dwetat[67]的研究报道，有氧运动能使肥胖个体体重降低，肥胖指数下降。刘善云的研究表明[68]，长期有氧运动能提高机体对脂肪的利用率。于青梅[69]报道有氧运动能加速脂肪的动员速度，增加脂肪供能效率。Gllbert 的研究显示，有氧运动能提高脂肪动员的限速酶的活性。Sep 等人报道长期进行有氧运动可使 LPL 活性提高 35%。本实验过程中，采用多种形式的运动，运动时全身各大小肌群都参与活动，而运动肌对各种供能物质的利用比率主要取决于运动强度及运动持续时间。持续运动时间越长，依靠脂肪氧化供能占人体总能量代谢的百分率也越高。而本研究的有氧锻炼中，运动强度是 60%－65%最高心率，每次持续运动 60 min 的有氧运动，体内脂肪不断通过水解和氧化以供骨骼肌能量，导致体内脂肪组织减少。

在本研究实施运动处方过程中，规律性的增加了力量 、耐力和柔韧性锻炼，在保证减少脂肪体重的同时，保持或适当增加肌肉体重，以保证良好的身体机能和健康水平。通过对照组和实验组的对比分析表明男生肌肉重量显著增加,这些反映出了健身运动对身体成分的良好影响,如果依靠节食减轻体重将造成肌肉重量明显下降。本实验结果男性肥胖少年在减少脂肪的同时，肌肉、无机盐增幅比女性肥胖少年较大，原因可能是本研究对象所选年龄段的男性肥胖少年正处在青春发育期,这个阶段是男性力量发展的高峰期。另外在实施运动处方的过程中，增加了力量和耐力训练的内容，所以在减少脂肪体重的同时促进了肌肉、骨骼的生长发育。

5.3 运动对肥胖少年身体素质的变化

本实验所选身体素质测试指标是全国学生体质健康标准中部分指标,反映了受试者力量、柔韧、爆发力等素质，握力主要测试前臂以及手部肌肉的力量。背力测试腰背部肌肉的最大伸展力。纵跳通过测试受试者的纵跳高度，反映下肢的弹跳力。坐位体前屈主要测量下肢的柔韧性。

通过数据分析得知肥胖实验组和肥胖对照组在实验前，身体素质各项指标除女性肥胖少年对照组的背力比女性肥胖少年运动组高，其他指标没有显著性差异。本实验结果发现肥胖运动组男生经过 12 周锻炼后握力增加，从 29.03 ±8.62 增加到 32.15±8.38，具有显著性差异(P<0.05)。柔韧程度改善，坐位体前屈从 3.67±6.59 增加到 4.93±7.39，具有显著性差异(P<0.05)。背力增大，纵跳能力提高，具有非常显著性差异(P<0.01)。肥胖对照组男生没有参加锻炼，12 周后随着身体的发育，握力相应增加，从 30.89±7.65 增加到 36.61±8.19，具有显著性差异(P<0.05)。背力增大，从 95.11±33.01 增加到 112.89±27.14。，具有显著性差异(P<0.05)。纵跳能力提高，从 22.47±5.67 提高到 26.14±6.93，具有显著性差异(P<0.05)。坐位体前屈在实验前后变化无显著性差异(P>0.05)。

肥胖运动组女生经过 12 周锻炼后握力增加，从 22.09±4.10 增加到 24.84±3.45，具有显著性差异(P<0.05)。柔韧程度改善，坐位体前屈从 7.53±9.51 增加到 11.03±8.03，具有显著性差异(P<0.05)。背力增大，纵跳能力提高，变化具有非常显著性差异(P<0.01)。肥胖对照组女生没有参加锻炼，12 周后随着身体的发育，握力增加，从 22.80±4.96 增加到 26.81±5.76，具有显著性差异(P<0.05)。背力、纵跳、坐位体前屈在实验前后变化无显著性差异(P>0.05)。

进行两组之间的对比分析发现，12 周前第一次测试，男生运动组和对照组身体素质各项指标没有显著性差异，运动组经过 12 周的锻炼后和对照组对比，握力、背力、纵跳出现显著性差异 (P<0.05)，坐位体前屈指标没有显著性差异(p>0.05)。12 周前运动组女生和对照组女生除背力之外其他身体素质各项指标没有显著性差异，12 周锻炼后运动组女生背力增加到 88.20±16.42，而对照组女生只有 69.89±23.49，12 周后两组背力、坐位体前屈有显著性差异 (P<0.05)，而握力、纵跳没有显著性差异(p>0.05)。

以上可以看出，12 周的运动干预后，肥胖运动组男生握力、纵跳、背力增大，坐位体前屈变化不显著，男性肥胖少年力量素质增长显著。肥胖运动组女生经过 12 周锻炼后背力、坐位体前屈增加，握力、纵跳无显著变化。由于本实验对象为处在青春发育期的肥胖少年，所以在运动组和对照组进行对比分析时发现，对照组因为身体的生长发育，12 周后部分指标也在增加，但是和运动组 12 周后的结果对比，身体素质的指标变化仍然存在显著性差异。

肥胖少年在参加健身锻炼之前体力活动少、身体协调性、灵活性和柔韧性较差[52]。本实验表明实施 12 周的运动处方后，男女肥胖少年身体素质均有提高。本实验中肥胖对照组女生 12 周前后背力、纵跳，体前屈都没有明显变化，但是肥胖运动组女生各项指标都显著改

善，本实验表明当肥胖对照组女生身体素质不再提高的情况下，肥胖运动组女生进行锻炼，能明显提高身体各项素质。

5.4 运动对肥胖少年运动能力的变化

运动能力是体现全国学生体质健康的重要组成部分。横跨反映的是受试者的谐调性和灵敏性。50 米反映的是速度素质。立卧撑间接反映受试者肌肉持续工作的能力。

本实验中，肥胖运动组男生经过 12 周锻炼后运动能力明显改善，各项测试指标都提高，并且实验前后变化具有非常显著性差异(P<0.01)。肥胖运动组女生经过 12 周锻炼，50 米跑的速度加快，从原来的 9.89±1.11（秒）提高到 9.36±0.78（秒），身体灵敏谐调能力增强，12 周后，横跨从 28.10±3.48（个/分）增加到 31.10±3.70（个/分），变化具有显著性差异(P<0.05)。立卧撑锻炼前后变化没有显著性差异(P>0.05)。

以上可知，运动除了可以减轻体重，可以提高肥胖少年身体各项运动能力.经过 12 周的锻炼，肥胖组的男女生运动能力都有所提高。其中男生提高幅度较大。可能和青少年生长发育特点以及男女青少年不同时段的生长发育高峰出现有关。

本实验采用中等运动强度和多种运动方式。在本方案中，一方面中等运动强度能够较多地利用脂肪供能，有助于减少脂肪体重[66]。同时中等强度的训练在运动技能形成过程中主要作用是提高锻炼者的动作技能熟练程度，常常表现出完成动作时身体的协调性、灵活性、等综合能力的提高。另一方面，在锻炼中采用多种运动方式对提高锻炼者身体有多重作用。在本次实验中，可以看到肥胖少年运动能力、协调能力普遍显著提高。

6 结论

6.1　实施 12 周的运动处方后，男女肥胖少年身体成分发生良好改变，主要表现为瘦体重增加，体脂百分比减少，BMI 降低，肥胖度减少，并阻止了肥胖的进一步加重。男肥胖少年更着重于瘦体重的增长。

6.2　实施 12 周的运动处方后，男女肥胖少年上臂、背部、腹部皮褶厚度减少，女性肥胖少年身体围度和腰臀比减少。

6.3　实施 12 周的运动处方后，提高了肥胖少年的身体素质，使运动能力、协调能力显著提高。男生运动能力提高幅度比女生大。

7 建议

建议肥胖少年不要盲目的节食减肥或者不科学运动，锻炼时采用心率法控制运动强度，并养成规律运动的习惯,减少脂肪重量的同时，增加瘦体重，提高身体素质和运动谐调能力，从而收益终生。

致谢

本文是在导师王正珍教授的悉心指导下本人独立完成的，其中倾注了导师大量的心血。谨此向尊敬的导师表示衷心的感谢！本文的撰写，从选题、开题、收集资料、实验到论文初稿后的批阅、修改，每一个环节都凝结着导师的心血。大到论文结构框架的调整，小到用词、标点符号，导师都一一审阅。导师严谨的治学态度，宽以待人的处世原则，一丝不苟的敬业精神都对我产生了很大的影响。从导师身上，我学到的不仅是知识和做学问的方法，更重要的是做人的道理。三年来，导师对我严格要求，关怀备至，在我人格和学业上起到醍醐灌顶的作用，促使我不断前进。

在实验过程中得到了北京八一中学体育教研室赵小倩老师的热情帮助和支持，另外，还得到了清华大学研究生院刘俊玲、张舒同学，北京体育大学赵慧娟、蔡利敏、洪锦峰同学的无私帮助。在此表示诚挚的谢意。最后借此机会，借此一角，向所有关心我，帮助我的各位老师和同学们表示感谢！

文献资料

1 .Schonfeld Warden N, Warden CH. Pediatric obesity: An overview of etiology and treatment(J).Pediatr Clin North Am. Apr 44(2):339-361.1997.
2.刘纪清，李国兰.编著《适用运动处方》 黑龙江科学技术出版社，1993.
3. Caterson ID, Management Strategies for Weight Control, Eating, Exercise and Behavior(J),Drugs.39 suppl 3:20-32., 1990.
4.向红丁.肥胖与代谢综合症一中国之现状。现代康复.5(7):12-14,2001.
5. Colditz GA, Willett WC，Rotnitsky A, et al. Weight gain as a risk factor for clinical diabetes in women(J).Arch Int Med.122:481-486,1995.
6. Lean MEJ,Hans TS,Seidell JC.Impairment of health and quality life in people with large waist circumference (J).Lancet. 351:853一856,1998.
7.Kennel WB,D Agostino RI3,Cobb JL. Effect of weight on cardiovascular disease(J).Am J Clin Nutr.63 (suppl 4):19s-22s.1996.
8.Kopelman.Causes and consequences of obesity(J).Med Int.22:385-388,1994.
9.Blair SN. Evidence for success of exercise in weight loss and control. Ann Intern Med.:702-706,1993.
10.Peter G. Obesity as a medical problem. Nature (J).404 (6):635-642, 2000.
11.李红政，雷美英综述，姚树桥审校.社会心理因素与单一纯性肥胖(J)国外医学社会医学分册 2001,18(1):1-3.
12.董砚虎，孙黎明，李利. 肥胖的新定义及亚太地区肥胖的重新评估与探讨， 辽宁实用糖尿病杂志.9(2):3-6,2001.
13 国际生命科学学会中国办事处中国肥胖问题工作组：中国成人体质指数分类的推荐意见简介 中华预防医学杂志 2001；35(5)：349-350
14.中国肥胖问题工作组数据汇总分析协作组：我国成人体重指数和腰围对相关疾病危险因素异常的预测价值，适宜体重指数和腰围切点的研究。中华流行病学杂志，2002；23(1)：5-10。
15.陈吉棣，曹国华. 运动在减肥综合措施中的地位 (J).中国运动医学志.8 (3):167-172,1989.
16. 孟昭恒.对儿童、青少年少年肥胖判定方法的评定((J).中华预防医学杂志.32(3):185-186,1998.
17. 陈春明，肥胖防治刻不容缓，中华预防医学杂志，2001,35(5)
18. 《中国成人超重和肥胖症预防与控制指南》编写组，预防肥胖指南
19. 史轶繁等：肥胖症临床诊治手册 上海科学技术出版社；2001年
20. Dorien PC ;van Aggel-Lei. jssen,Wim HM Saris, Gabby B HuLet al. Short-term effect of weight loss with or without low intensity exercise training on fat metabolism in obese men .Arn J Nutr73:523-531, 2001.
21,沈稚舟.单纯性肥胖的饮食和运动疗法(J).上海预防医学杂志.9（10）11—12,1997.
22.叶超群.饮食控制，运动对肥胖者免疫功能影响的研究进展(J).中国运动医学杂志.18(10):51-53,1999.
23.Luc Tappy, Jean P.Fellber,et al. Energy and substrate metabolism in obesity and nosmhese statefll Dinhetes Care_14 (12)- 1 IRO- 11 RR 1991
24.邱斌. 生物电阻抗技术(BIA) 测定体脂百分含量在肥胖及相关疾病研究中的意义 2002年硕士学位论文
25 ..Denen ME, Hennessey JV, Markert RJ. Outpatient evaluation of obesity in adults and children: A review of the performance of internal medicine/pediatrics residents.

J Genet Intern Med,1993:8:268
26.国际生命科学学会中国办事处中国肥胖问题工作组：中国成人体质指数分类的推荐意见简介 中华预防医学杂志 2001；35(5)：349-350
27.中国肥胖问题工作组数据汇总分析协作组：我国成人体重指数和腰围对相关疾病危险因素异常的预测价值：适宜体重指数和腰围切点的研究。中华流行病学杂志，2002；23(1)：5-10。
28.赵连成，武阳丰，周北凡等：体质指数与冠心病、脑卒中发病的前瞻性研究 2002，中华心血管病杂志 30(7)：430-433
29.WHO reassesses appropriate body-mass index for Asian population. The Lancet, 2002, 360:9328 7-20
30. National Institutes of Health, National Heart Lung, and Blood Institute in cooperation with the National Institute of Diabetes and Digestive and Kidney Diseases: Clinical guidelines on the identification, evaluation, and treatment of overweight and obesity in adults. The evidence report. NIH Publication, no. 98-4083, September 1998
31. National Health and Medical Research Council: Acting on Australia's weight: a strategic plan for the prevention of overweight and obesity. 1998
32. Scottish Intercollegiate Guidelines Network: Obesity in Scotland. integrating prevention with weight management. A national clinical guideline recommended for use in Scotland. Pilot edition, November 1996.
33.王金道，主编.临床疾病心理学.北京:北京师范大学出版社，1994,333
34. Gerald LB，Anderson A，Johnson GD，et al. Social class，social support and obesity risk in children. Child: Care，Health Der 1994:20:145
35.Ho TF，Yip WC，Tay JS，et al. Social class distribution of obese Chinese children. J Singapore ped soc，1991:133:55
36.Borjeson M. The Aetiology of obesity in children — A study of 101 Twin Pairs Acta Peadiat Scand. 1976,65(3):279.
37. Schlicker SA，Borra ST，Regan C. The Weight and fitness status of United states children. Nutr Rev 1994 , 52:11
38.夏庆华，汪玲，王文英，等.肥胖儿童、青少年身体素质的配对研究.中国公共卫生，1998，14(7):404
39.Jhonston FE. Health implication of childhood obesity. Ann Inter Med，1985，101 .1069 — 1072(5):473 — 474
40.夏庆华〔综述〕.肥胖对儿童、青少年身心健康的影响.中国学校卫生.2001,22
41.X Li. A study of intelligence and personality in children with simpleobesity. lnt J Obes，1995，19(1):1 — 3
42.夏庆华，汪玲，王文英，等.单纯性肥胖儿童、青少年智能状况的配对研究.上海预防医学杂志，1998,10(5) :210 — 212
43.仰庆惠.关于肥胖的治疗(J).中国运动医学杂志，1994 ,13 (2) :106 .
44.刘纪清，李国兰.实用运动处方.哈尔滨:黑龙江科学技术出版社，1993
45.蒋竞雄，丁宗一儿童、青少年期单纯肥胖症的综合治疗方案.中华医学杂志. 1991. 71 (8): 473-475
46. Carl NK，ark DS. Exercise testing and prescrption. Sports Med. 1996, 21 (5):326^-336
47.池上晴夫.健康人运动处力.国外医学，物理医学与康复学分册，1988 (1): 31-32
48.日本体育科学中心编，吕帆译.日本健身运动处方.北京:人民体育出版社，1980

49. Jun SK, et al. A long-term aerobic exercise program decrease the obesity index and increase the high density lipopratein cholosterol concertration in obese children. Int Obesity, 1987, 1:330
50. S M Fox III, et al. Physical activity and the prevention of coronary heart disease. Annals of Clin Res. 1971, 3:404
51.龚贞观等. 对肥胖少年进行运动减肥实验， 中国学校教育 99-4： 55
52.王正珍等 健身运动对超重和肥胖少年身体素质的影响 .沈阳体育学院学报 Vol. 23 No. 3, Jun.2004
53.于素梅 .肥胖与有氧运动减肥的生物学分析 Vol. 24 No. l Mar. 2001
54.颜宜芭等.有氧运动对饮食诱导单纯性肥胖大鼠脂蛋白代谢的影响「J).中国运动医学杂志，1998 ,(7).
55.Harsh Detach . composition and childhood obesity. Endocrinal metab chin N . Am .1996.
56.刘善云.有氧运动对小鼠到脂血症及脂蛋白代谢的影响(J).中国教育科学，1998,(4) .
57.于素梅.肥胖与有氧运动减肥的生物学分析「J」.北京体育大学学报，2001, (1).
58.王从容.运动减体脂和药物减体脂「J」.天津体育学院学报，1999,(1).
59.Treuth MS, Figueroa-Colon R, Hunter GR, et al. Energy expenditure and physical fitness in overweight versus non-overweight pre-pubertal girls(J).Int J Obesity. 1998, 22: 440-447
60.彭绩 体质指数、腰臀围比与高血压、糖尿病患病关系研究 数理医药学杂志 2000 年
61.张新华 ,健美操对大学女生心脏功能及腰臀围之比的影响 哈尔滨师范大学自然科学学报 Vol .19 , No.6 2003
62.杜熙茹 健身运动对肥胖儿童、青少年健康的影响.广州体育学院学报 Vol. 23 No. l ,2003
63..Scott Owens, Bernard Gutin, Jerry Allison, et al. Effect of physical training on total and viseral fat in obese children(J). Med Sci Sports Exercise . 1999, 31: 143一148
64. 国家体育总局群体司 ，2000 年国民体质检测报告
65. 黄晓丽:有氧健身操对肥胖型女大学生身体成分的影响，哈尔滨学院学报，Vol.24 No.12 .Dec .2003
66.Exercise testing and prescription fox special cases . Theoretical Basis andclinicapplication，James，1994
67. Harsh Detach .bocomposition and childhood obesity.Endocrinal metab chin N . Am .1996.
68. 刘善云.有氧运动对小鼠到脂血症及脂蛋白代谢的影响(J).中国教育科学，1998,(4) .
69. 于素梅.肥胖与有氧运动减肥的生物学分析[J) .北京体育大学学报，2001, (1).
70. 王从容，等.运动减体脂药物减体脂[J].天津体育学院学报，1999,(1).

附件 1

给家长的一封信

尊敬的____________家长同志：

您好！

据调查现代社会中肥胖患者越来越多，单纯性肥胖不仅是冠心病，糖尿病，高血压脑血管意外，癌症等发病的危险因素，还会降低人的自然寿命。特别是少年儿童的单纯性肥胖则更为突出，少年单纯性肥胖症的发生率正逐年提高，并由此伴随的少年体质普遍下降，也越来越引起人们的重视，所以肥胖的防治已成为少年健康的一个重大课题。

北京体育大学运动人体科学学院和八一中学共同合作，挑选八一中学部分超重和肥胖少年通过一系列详细的测试，针对每一个参加体能锻炼学生的心肺功能及其它指标，制定出运动处方，并正在进行科学的体育锻炼。

为了不影响孩子的学习，锻炼时间安排在每周一至周五的下午放学后，由老师带领同学运动，锻炼时间为 60 分钟（包括准备活动，跳绳，踢毽，沙包，体育游戏，定向越野，健美操，柔韧训练，女生的形体锻炼，力量训练，球类等运动），每天会有签到和离开的时间登记。通常周六下午会安排一次户外运动，根据同学的自身情况选择参加。体能小组选择从三月到六月初这段气候适宜的季节开展训练，希望体能小组的同学不光能减轻身体的重量，而且提高自信心，身体各方面机能都得到改善，从而增强体质，提高中考体育成绩但是在进行体育锻炼的同时，如果饮食不当，或者没有进行有规律的进行锻炼将会减弱应有的效果，因此希望各位家长平时能注意孩子的饮食，督促孩子按时有规律的锻炼，希望每周六次运动能练习四次以上，以配合体育锻炼取得预期的效果。另外您有什么问题可及时和体能小组的老师联系，根据签到离开时间了解您孩子的离校时间，避免孩子贪玩影响学习。

先将您的孩子近期的出勤情况反馈如下：

从_____到____,   出勤_____次   户外活动参加_____次

最后希望通过我们的共同努力，达到最好的效果！

家长签字

北京体育大学和北京八一中学联合体能小组 2004.4.2

附件 2

the International Obesity Task Force(IOTF) 2000 年的标准

Measurement and definition

Table 1.1. International body mass index cut-offs for overweight and obesity by sex between 2 and 18 years, defined to pass through body mass index 25 and 30 at age 18 years

Age (years)	Body mass index 25		Body mass index 30	
	Boys	Girls	Boys	Girls
2	18.4	18.0	20.1	19.8
2.5	18.1	17.8	19.8	19.5
3	17.9	17.6	19.6	19.4
3.5	17.7	17.4	19.4	19.2
4	17.6	17.3	19.3	19.1
4.5	17.5	17.2	19.3	19.1
5	17.4	17.1	19.3	19.2
5.5	17.5	17.2	19.5	19.3
6	17.6	17.3	19.8	19.7
6.5	17.7	17.5	20.2	20.1
7	17.9	17.8	20.6	20.5
7.5	18.2	18.0	21.1	21.0
8	18.4	18.3	21.6	21.6
8.5	18.8	18.7	22.2	22.2
9	19.1	19.1	22.8	22.8
9.5	19.5	19.5	23.4	23.5
10	19.8	19.9	24.0	24.1
10.5	20.2	20.3	24.6	24.8
11	20.6	20.7	25.1	25.4
11.5	20.9	21.2	25.6	26.1
12	21.2	21.7	26.0	26.7
12.5	21.6	22.1	26.4	27.2
13	21.9	22.6	26.8	27.8
13.5	22.3	23.0	27.2	28.2
14	22.6	23.3	27.6	28.6
14.5	23.0	23.7	28.0	28.9
15	23.3	23.9	28.3	29.1
15.5	23.6	24.2	28.6	29.3
16	23.9	24.4	28.9	29.4
16.5	24.2	24.5	29.1	29.6
17	24.5	24.7	29.4	29.7
17.5	24.7	24.8	29.7	29.8
18	25	25	30	30

万方数据 WANFANG DATA 文献链接

运动对肥胖少年体成分、体脂分布及其身体素质的影响

作者： 王蓓蓓
学位授予单位： 北京体育大学
被引用次数： 1次

本文读者也读过(3条)

1. 刘身强 耐力锻炼结合力量练习对高一学生身体成分和VO<,2>max影响的实验研究[学位论文]2008
2. 欧阳凤秀.王文英.王震维 儿童肥胖度、体脂分布与贫血的关系研究[期刊论文]-中国公共卫生2001,17(7)
3. 金利新.朱钦 山东潍坊市汉族城乡学生身体体成分分析[期刊论文]-解剖学杂志2004,27(6)

引证文献(1条)

1.杨梦利.娄晓民.彭玉林.王瑞娟.李岚.郭蔚蔚 大学生BMI与身体素质指标的相关性[期刊论文]-中国学校卫生 2013(9)

引用本文格式：王蓓蓓 运动对肥胖少年体成分、体脂分布及其身体素质的影响[学位论文]硕士 2005
