# Supplementary material for: Systematic review and meta-analysis of school-based obesity interventions in mainland China
Source: PLoS One. 2017 Sep 14;12(9):e0184704. doi: 10.1371/journal.pone.0184704 (PMC5598996; doi:10.1371/journal.pone.0184704)
Supplement: S1 Dataset — (ZIP) [file pone.0184704.s007.zip › S1_dataset/76库/86.pdf]

独 创 声 明

本人郑重声明：所呈交的学位论文是本人在导师指导下进行的研究工作及取得的研究成果，学位论文的知识产权属于山西师范大学。除了文中特别加以标注的地方外，论文中不包含其他人已经发表或撰写过的研究成果，也不包含为获得山西师范大学或其他教育机构的学位或证书使用过的材料。本声明的法律后果将完全由本人承担。

作者签名：解达之　　　　签字日期：2013. 5. 29

学位论文版权使用授权书

本学位论文作者完全了解山西师范大学有关保留、使用学位论文的规定，有权保留并向国家有关部门或机构送交论文的复印件和电子版，允许论文被查阅和借阅。本人授权山西师范大学可以将学位论文的全部或部分内容编入有关数据库进行网络出版，可以采用影印、缩印或扫描等复制手段保存、汇编学位论文。（保密的学位论文在解密后适用本授权书）。

作者签名：解达之　　　　签字日期：213. 5. 29

导师签字：[illegible]　　　　签字日期：2013. 5. 29

Y2307329

论文题目： 运动与健康教育联合干预对山区儿童体质健康的影响

专　　业： 体育教育训练学

硕 士 生： 解达立　　　　签名：解达立

指导教师： 乔玉成　　　　签名：乔玉成

摘　要

研究目的：调查了解山区儿童体质状况及其影响因素，观察运动和健康教育联合干预对山区体质健康指标的影响，探讨增强山区儿童体质的方法，为提高山区儿童体质与健康水平提供理论依据。

研究方法：1.查阅文献资料，了解我国不同地域儿童体质健康现状及其影响因素。2.通过问卷调查，了解山区儿童的饮食、生活习惯及参加体育锻炼的情况；采用体质测量的方法明确山区儿童的体质健康状况。3.选取万荣县万泉小学 92 名学生为研究对象,随机分为对照组 32 人，运动干预组 30 人，运动和健康教育联合干预组 30 人，分别对运动干预组、运动和健康教育组进行为期 12 周的运动与运动和健康教育联合干预。选用身高、体重、胸围、腰围、臀围、肱三头肌皮褶厚度、腹部皮褶厚度、握力、背力、跳远、50 米跑、50 米×8 跑、肺活量、血压等指标进行测量，计算出 BMI、WHR。将所得数据输入计算机进行分析处理，对实验前后的结果进行单因素方差分析和组间、组内比较。

研究结果：1.山区学生在日常生活习惯方面干预前后变化不大，但参加体育锻炼行为、性格、胆量方面要好于干预前。2 身体形态指标干预前后变化不大。3 身体机能指标肺活量干预后要明显高于干预前，且差异显著（$p<0.01$），机能指标血压干预前后变化不大（$p>0.05$）。4 身体素质方面，表现力量素质的背力，柔韧素质的坐位体前屈；爆发力的跳远；耐力素质的 50 米×8；速度素质的 50 米显著于好干预前（$p<0.05$），但反映力量素质的握力指标变化不大（$p>0.05$）。

结论：运动与健康教育联合干预对山区儿童体质健康状况具有显著作用，是提高山区儿童体质水平的有效手段。

【关键字】体质　干预　运动与健康教育　山区儿童

【论文类型】应用研究

Title: The effects of Sports and health education Joint intervention on children's physical health in mountain areas
Major: Sports pedagogy and Training

Name: Xie Dali **Signature:** Xie Dali

Supervisor: Qiao Yucheng **Signature:** Qiao Yucheng

Abstract

Research objectives: to offer theoretical basis on improving the body and health condition of children in mountainous area, the paper investigates the condition of children body in mountainous areas and its influencing factors, observes the influence of joint intervention of sports and health education on body health indicator and discusses the methods of improving children body in mountainous area.

Research methods: 1. reading documents and literatures, knowing children body health condition and its influencing factors in different areas. 2. Through the questionnaire survey, understanding mountain children's diets, living habits and the situation about participating in the physical training the situation, determining the research object. 3. This study selected 92 students from Wan Quan primary school of Wan Rong Town as the research objects. And there are 32 students in control group, 30 students in sports group and 30 students in sports-health education group. It takes 12 weeks of sport and sports - health education intervention to the sports group, sports -health education group respectively. Before and after the experiment according to the same equipment, the same test method acquisition subjects body mass index of the original data, the selection of height,

body weight, chest circumference, waist, hip circumference, triceps skinfold, abdominal skinfold, grip strength, back force, long jump, 50 m running, 50mx 8 run, vital capacity, blood pressure indicator for measuring, calculate BMI, WHR. Input the data into the computer to carry on the analysis, and the experimental results before and after the single factor analysis of variance between the group and the comparison of the in the group.

Research results: 1. there are little change of mountain students in daily life habits before and after the intervention in physical exercise. While the character and courage is better than before in intervention. 2. physical aspects in general, students' physique in mountainous area after intervention is better than before the intervention. 3. aspects of body form without big changes before and after the intervention. 4 .to body function aspects in lung capacity after intervention was significantly better than that of before intervention and significant difference. Blood pressure without big changes before and after the intervention. 5.aspects of physical strength quality back force; The quality of the soft body bending on seats; Bursts of long jump; Endurance quality 50 m ×8; The quality of the speed 50 m significant better than the before intervention, and in power quality grip strength aspect does not play a significant role.

Conclusion: the method of joint intervention of sports and health education on body of mountainous area children is an effective way and has a significant role in improving their body conditions.

【key words】 physical intervention sports and health education mountainous area children

【The paper type】 application research

目 录

1 引言 1
1.1 选题依据 1
1.2 研究目的 1
2 文献综述 3
2.1 相关概念的界定 3
2.2 影响体质的因素 3
2.2.1 遗传因素 3
2.2.2 环境因素 3
2.2.3 营养因素 4
2.2.4 体育锻炼因素 4
2.3 国内外有关青少年体质健康的研究动态 5
2.3.1 国外的研究动态 5
2.3.2 国内的研究动态 7
2.4 山区体育教育现状 7
3 研究假设 9
4 研究对象与方法 11
4.1 研究对象 11
4.2 研究方法 11
4.2.1 文献资料法 11
4.2.2 问卷调查法 11
4.2.3 实验法 11
4.2.4 数据的统计与处理 14
5 结果与分析 15
5.1 实验对象所在地的基本情况 15
5.2 运动和健康教育干预对山区小学生日常饮食习惯的影响 15
5.3 运动和健康教育联合干预对山区小学生参加体育锻炼活动的影响 17

5.4 运动和健康教育联合干预对山区小学生心理健康状况的影响 18
5.5 运动和健康教育联合干预对山区儿童身体形态结构的影响 19
5.5.1 运动和健康教育联合干预对山区儿童身高、体重和 BMI 指数的影响 19
5.5.2 运动和健康教育联合干预对山区儿童胸围、腰围、臀围、WHR 的影响 21
5.5.3 运动和健康教育联合干预对山区儿童肱三头肌和腹部皮褶厚度的影响 23
5.6 运动和健康教育联合干预对山区儿童生理机能的影响 24
5.6.1 运动和健康教育联合干预对山区儿童安静状态下血压的影响 24
5.6.2 运动和健康教育联合干预对山区儿童肺活量的影响 25
5.7 运动和健康教育联合干预对山区儿童身体素质的影响 26
5.7.1 运动和健康教育联合干预对山区儿童力量素质的影响 26
5.7.2 运动和健康教育联合干预对山区儿童速度素质的影响 28
5.7.3 运动和健康教育联合干预对山区儿童柔韧素质的影响 28
5.7.4 运动和健康教育联合干预对山区儿童耐力素质的影响 29
6 讨论 31
6.1 运动和健康教育对山区儿童日常行为习惯和心理健康的影响 31
6.2 运动和健康教育联合干预对山区儿童身体形态的影响 31
6.3 运动和健康教育联合干预对山区儿童生理机能的影响 33
6.4 运动和健康教育联合干预对山区儿童身体素质的影响 33
7 结论与建议 39
7.1 结论 39
7.2 建议 39
致谢 41
参考文献 43
附录 45

1 引言

1.1 选题依据

一个国家的兴衰，和该国少年儿童的体质健康分不开的，少年儿童是“初升的太阳”，是共和国未来的建设者和接班人。当今少年儿童的体质状况如何，能否能成为未来合格建设者和接班人，是全社会共同关注的焦点。

自 1985 年始，五年一次的中国学生体质与健康调研结果显示，我国中小学学生身体形态及生长发育水平有较大幅度的提高，如身高、体重、胸围等发育指标持续增长，然身体机能和素质增长不明显，甚至还出现了下降的趋势，明显落后于身体形态生长水平的提高，主要表现为关节灵活性差、肌肉力量差、动作不协调。同时，儿童心理状况也不容乐观，主要表现为情绪调节能力较差，抗挫折能力低下，心理承受能力较弱等。此外，儿童近视率持续增加，糖尿病和高血压等成年性疾病开始呈现低龄化趋势，并有由城市向农村蔓延的趋势。儿童体质和健康水平关系到国民素质的大事，是衡量国力的重要指标。近年来，各级政府部门对中小学生健康状况高度重视，并采取了一系列措施，取得了明显成效。

长期以来，我国学者对中小学生的体质进行过很多研究，积累了丰富的经验，找出了影响我国小学生体质健康水平下降的主要原因，并提出了一些改进措施和方法。[1]但大多数研究都针对城市儿童为研究对象进行的，对农村儿童尤其是山区儿童很少涉及。山区儿童的生存环境、生活方式、饮食习惯、体育锻炼活动、家庭经济状况、接受教育方式、生长发育特点及规律等与城市和平川农村有着较大的差别。为此，本研究将选择山区儿童为研究对象，通过现场调查和测量山区儿童体质状况，并采取运动与健康教育联合干预措施进行干预，观察其对山区农村儿童体质健康的影响，为制定相关干预策略，促进山区儿童健康成长提供依据。

1.2 研究目的

近几年来，随着社会的发展，生活水平不断的提高，人们参加体育锻炼的方式也多种多样，对体质也越来越重视，体质研究也就成为十分重要的研究领域。在我国开展全面、系统的体质研究已有 20 多年的历史，有关体质的概念、内容、测量与评价、比较研究、干预研究等研究内容涉及到多方面，但是过去研究大都针对城市儿童，对农村儿童尤其是山区儿童的研究几乎成为空白。本研究通过现场调查以及对山区儿童进行运动+健康教育干预的实验研究，以期达到如下目的：

（1）了解山区儿童的日常生活行为习惯和体质健康现状。

（2）探讨运动和健康教育联合干预对提高山区儿童体质健康水平的效果。

（3）找出适合提高山区儿童体质健康水平的健康促进措施以及体育锻炼方案并进行推广。

[1] 夏磊.运动干预对西安市小学生体质健康影响的实验研究[D].西安体育学院硕士论文 2012,5

2 文献综述

2.1 相关概念的界定

运动干预：是指测试者按照一定的运动方案和目的，采取主动施加运动的措施，通过适量的运动负荷、运动强度和运动频率，在一定时间段内对被试者进行持续且循序渐进的干预，通过这种科学合理的方式，改善因为缺少运动或运动方式不当而引起的诸如心肺功能不全、抵抗力差等不良后果，从而达到干预的预期目的。[1]

健康教育干预：按照一定的教育方案，用口头或书面的形式，有组织、有计划的对被试者进行一段时间的干预，从而达到预防疾病、促进健康、提高生活质量的目的。

体质：关于体质的定义较多，目前，使用较多的有：（1）中国学生体质与健康研究组的体质定义：人体的健康状况和对外界的适应能力，具体包括：身体形态发育水平、生理生化功能水平、身体素质、运动能力、心里状态、适应能力[2]。（2）中国体育科学学会体质研究分会于 1982 年对其下的定义:体质是指人体的质量，一般认为它是在先天遗传和后天获得的基础上形成的、表现为人体形态结构、生理机能、心理因素和功能活动均相对稳定的特征。[3]

山区：就其地理意义而言，通常来讲包括山地和丘陵分布地区，也包含比较崎岖的高原，就其人文意义而言，指包含一定海拔、坡度、地貌以及人口、资源等在内的综合生活区。在本文研究中，山区是相对于城市而言的，它专指远离城市的、经济相对欠发达地区，在该地区，基础设施建设较为薄弱、人民物质生活水平较为低下，精神生活也相对匮乏。

2.2 影响体质的因素

2.2.1 遗传因素

遗传对体质的好坏有重要的影响，是人体发育的先决条件，大量的研究证实人的体质与遗传密切相关。如，Bouchard 等学者认为，在青春发育期遗传对体质的影响较为明显，研究发现体脂的遗传度是 0.25，肌肉能力的遗传度是 0.2-0.4，心血管能力的遗传度是 0.1-0.25。[4]张占平的研究表明，在体型指征相关指标中，BMI 和 WHR 均受遗传因素影响较大。其中，女性的 BMI 和 WHR 主要受遗传因素的影响,而男性 WHR 与遗传因素关系不大,主要受环境因素的影响。[5]程颖曾对沈阳市内五个区 15 所中小学 16037 名学生的身高进行过调查研究，检出身高低于同年龄、同性别的人数有 72 例，发育配对分析表明致矮的因素主要为遗传因素。[6]

2.2.2 环境因素

遗传决定了生长发育的可能性，而环境影响遗传基因的表达。在对文献进行研究梳理时，我

[1] 王增峰.以运动干预为手段改善北京市大学生心理健康状况的可行性探析[D].北京体育大学硕士论文 2007 5.
[2] 中国学生体质与健康研究组.2000 年中国学生体质与健康调研报告[M].北京:高等教育出版社,2002.
[3] 于道中.体质健康概念与我国学生体质健康状况[J].山东体育学院学报,1994,10(2):7-14.
[4] 刘路,影响体质的相关因素分析[J].大众体育,2011,6(22):151-153.
[5] 张占平.对高职高专学生体质健康现状及若干影响因素的研究[D].苏州大学硕士论文,2005,10.
[6] 程颖.中小学生矮身材发育状况及致矮因素研究[J].中国校医,1995,9(1)5-9.

们发现影响体质健康的外界因素主要包括自然地理环境和社会环境。

地理环境又称自然环境或自然地理环境。影响不同地区、不同生活习惯人的体质方面的差异与所在地理位置有关，所在地的气候特征、水土等都是影响体质的因素。胡卓生[1]在对甘肃省城市和农村的学生进行研究时，选取反映形态发育水平的身高、体重、胸围，反映身体机能水平的肺活量、脉搏、血压和反映身体素质的 50 米跑、立定跳远、握力、背力及坐位体前屈等指标进行统计分析。结果显示不同地区的学生在身体形态、身体机能和身体素质之间存在明显差异。杨文英[2]在对国家级贫困县岢岚县的小学生体质进行研究时发现，该地区学生身体发育方面不如城里学生，但在反应身体素质的速度素质和爆发力方面要好于城市的学生。

社会因素对人体质的的影响方面往往是最容易被忽视，但又是很重要的一个因素。社会因素主要包括经济、文化、教育、生活方式等。世界卫生组织研究结果认为，人们的生活方式及所在地区的经济水平对体质影响也比较大。[3]2005 年、2010 年全国学生体质调研结果显示，多数在校生的体质都有逐年下降的趋势，身体处于亚健康的状态，这与学生平时的不良生活习惯有关。姜文凯对少年儿童进行研究时发现，城市女孩在身体发育的敏感期发育比男孩快，一方面与遗传有关，另一方面与所在家庭的经济条件有关。[4]

2.2.3 营养因素

决定体质好坏的另一个物质条件是营养水平。长期的营养不良或营养过剩都不利于体质的发展。我国贫困地区由于长期营养低下，导致青少年身体发育迟缓、智力低下，受教育程度低下身体的机能和素质状况以及心理发育受到严重影响。对处于发育敏感期的儿童少年来说，营养对其体质健康有很重要的作用，营养情况不仅决定儿童少年基本的健康问题，同时也决定以后的发育和学习情况。2000 年学生体质调研结果与 1995 年相比，7-18 岁中小学学生的低体重及营养不良率分别下降了 1.1%和 7.6%，19-22 岁的大学生的低体重及营养不良率分别下降 10.5%和 31%。[5]沈勇伟研究显示，体院女大学生的 BMI、肺活量、均低于全国平均水平，分析原因可能与女大学生营养不良有关，营养调查发现她们平时的膳食中缺少大量的蛋白质、钙、维生素 E。[6]胡艳龙等对北京市某一寄宿制学校营养缺乏的学生进行 6 周的运动营养干预后，男女生的肌肉力量都有所增长，男生的肺活量和体重指数有所增长。[7]

2.2.4 体育锻炼因素

体育锻炼是人们增强体质最有效的手段之一，对体质的影响主要表现在生理和心理上。经常参加体育锻炼，可以使人的呼吸系统、运动系统、心血管系统得到改善。如赵强等的研究显示，从实施阳光体育运动以来，在校大学生的体型得到明显改善，超重、肥胖比例下降，男生的肌肉力量、

[1] 胡卓生.2000 年甘肃省城乡中小学生形态发育与体质健康状况[J].中国学校卫生,2004,25(6)730-732.
[2] 杨文英.对国家级贫困县岢岚县小学生体质现状的研究[D].太原理工大学,2008,05.
[3] 姚武,姚兴.学生体质下降的社会因素分析[J].体育科研,2008,29(3)87-90.
[4] 姜文凯,江苏省国民和学生体质监测某些跨系统共有指标的特征及其影响因素[J].体育与科学,2002,23(3) 40-45.
[5] 杨则宜.中国大陆学生体质状况及其运动和营养干预[J].体育科研,2006,6(27):65-68.
[6] 沈伟勇.女大学生营养与体质关系的比较研究[J].搏击武术科学,2009,6(11):87-89.
[7] 胡艳龙,李铁柱等.运动营养干预改善初中寄宿制学生体质健康效果的研究[J].2012,34(6):45-48.

下肢爆发力，女生的柔韧素质都有大幅的提高。[1]原黎君的研究显示，经常参加体育锻炼，可以使骨骼变粗、变厚，增强关节的牢固性，提高关节的灵活性，增加耐力素质、力量素质。[2]张翠琴的研究显示，经常参加体育锻炼的人，可以使心血管系统里面的心脏和体重的比值加大，心容积相对增大，使呼吸系统的肺脏弹性增大，呼吸肌力量增大，肺活量增大。[3]由此可知，体育锻炼对增强体质具有重要的作用。

2.3 国内外有关青少年体质健康的研究动态

2.3.1 国外的研究动态

（1）美国学生体质健康的研究

美国是世界上最早重视体质研究的国家之一，对体质的研究有着悠久的历史。对体质研究的有关文献进行整理后发现，美国的体质研究主要体现在测定和评价两大方面。

在19世纪80年代后期，美国许多学校就对本校的学生进行了体能测验其结果并非令人满意，但这些测试并没有引起美国有关部门的重视。直到 1954 年他们采用（Kraus-We-ber）测试法对美国儿童的腰背肌肉力量和柔韧性进行测试，并与欧洲儿童进行比较，才发现，美国儿童在这两方面的能力远不如欧洲儿童，这引起了美国当局的重视。[4]在之后的 1958 年，美国的各个研究机构共同研究，并设计了包括 50 米跑、600 米跑、垒球掷远、立定跳远、引体向上、往返跑、仰卧起坐在内的 7 个指标，对美国青少年的体质状况进行测试，与此同时这 7 个测试指标在全国范围内也开始试用。1985 年在有关部门的协助下，美国对全国的青少年学生进行了第二次的体质测试，这次测试项目包括 600 米跑、垒球掷远、引体向上、往返跑、仰卧起坐在内的 5 个指标，将立定跳远和 50 米跑两个项目删除。并规定在以后每隔 10 年对都全国青少年学生进行一次全方位的体质测试。

1988 年，美国开始制定体质健康发展目标，并开发出适合青少年身心发展的《最佳健身计划》指导青少年健身并采用新的指标进行体能测试，测试指标主要包括皮褶厚度、坐位体前屈和引体向上，这些指标主要用来评价青少年的身体成分、柔韧水平、肌肉耐力。如果这三个指标处于良好状态说明青少年具备良好的体质水平。

体质评价是体质研究中一个非常重要的环节。上世纪 80 年代至 90 年代，美国对学生体质评价主要采用两种评价方法，即常模标准和健康标准，常模标准是通过对大样本人群进行体质测验，以百分位数法统计出分布规律，通常以第 50 位作为标准，当对个体测试完成后，以其结果与这个常模标准进行比较。1983 年北卡罗来那州通过测试学生的 9 分跑、仰卧起坐、坐位体前屈、皮褶厚度（肱三头肌处、腹部）建立了健康标准，并以此标准把测试群体分为合格和不合格两大类，然后采取相应措施，促使青少年体质发展。

---

[1] 赵强,薛玉行.体育锻炼增强大学生的实证研究[J].体育文化导刊,2011,(4):94-97.
[2] 原黎君.再论体育锻炼对增强体质的作用[J].科技信息,2007,(26):493.
[3] 张翠琴.增强体质的手段—体育锻炼[J].山西师范大学体育学院学报,1996,11(2):53-56.
[4] 江崇民,张一民.中国体质研究进程与发展趋势[J].体育科学,2008,28(9):27-28.

（2）欧洲学生体质健康的研究

上世纪七十年代前，欧洲各国都有自己一套测试体质的方法。1978 年欧洲各国为了比较各国身体教育制度的效果，弄清影响体质状况的因素，统一了体质测定标准并签订了协议，开始相关的工作。[1]

1986 年整个研究工作结束后，欧洲联盟出版了统一的体质测试指南，并成立了名为“尤罗菲特”的委员会，他的主要任务是协调各国学生体质的测试工作，检查和比较他们的评定结果，并对他们的测试工作和评定结果提出建议。[2]具体的体质测试的内容主要有：身高、体重、速度素质、耐力素质、柔韧、平衡。瑞典在 1987 年和 2001 年对学生进行了两次抽样测试，结果显示，男生在呼吸系统、循环系统、肌肉素质都出现了下降的趋势，女生只在力量素质方面出现了下降的趋势。研究者认为，导致这种变化的原因之一是因为体育活动的数量和性质发生了变化，从而影响了学生体质的发展继而影响了国民体质的发展。[3]

（3）日本学生体质健康的研究

日本也是世界上对青少年体质比较重视的国家之一，同时也是学生体质调研资料最全的国家，积累了有关青少年学生体质发展的很多资料。早在 1879 年，日本就对本国部分青少年学生的体质进行了调查,测试了包括身高、体重、胸围、腰围、肺活量、握力在内的 6 项指标，1939 年日本为了霸占殖民地，进行了历史上规模最大的国民体质测定。第二次世界大战失败后，日本为了恢复国民健康，增强国家的实力，对本国的青少年进行了新一轮的体质测试，测试的指标主要是反映柔韧、灵敏、力量、耐力等方面的情况。[4]

随着科学技术的不断发展，经济水平的不断提高，世界各国都朝着信息化、多样化和老龄化的方向发展，各国的国民体质都会受到一定程度的影响，同样日本也不例外。于是日本在 1963 年和 1964 年分别对全国中小学以及大学颁布了《体质测定实施要案》，《要案》中规定全国的青少年必须进行体质测定和运动能力测定。1967 年，日本对本国的青壮年进行了一次体质测定，并规定在每年的 6 月份，都根据各自的《要案》进行统一的体质测定，并由各省对测定结果进行公布，1999 年日本对《要案》进行了修改，从此试行了新的测试标准。[5]

到了 20 世纪 80 年代后期，日本大多数学生及本国的公民都认识到体质的重要性，譬如在日本学生每个人手上都有一本有关体质各个项目的图形，以便和自己的身体状况进行比较，学生可以根据这样的标准有针对性的进行体育锻炼。日本的每个学生及其公民可以根据自身的年龄、性别、身体条件在任何时候、任何地方选择自己喜欢的运动方式进行体育锻炼。

[1] 杨文英.对国家级贫困县岢岚县小学生体质现状的研究[D].太原理工大学硕士论文,2008.
[2] 马思远.我国中小学体质下降及其社会成因研究[D].北京体育大学博士论文,2012.
[3] 何仲恺.体质与健康关系的理论于实证研究[D].北京体育大学博士论文,2001.
[4] 于可红,母顺碧.中国、美国、日本体质研究比较[J].体育科学,2004,24(7):51-53.
[5] 马思远.我国中小学体质下降及其社会成因研究[D].北京体育大学博士论文,2012.

2.3.2 国内的研究动态

建国以来，我国对国民体质的研究也非常重视。于 70 年代末 80 年代初开始了学生体质的研究，取得了丰硕的成果。

1979-1980 年，包括国家体委、教育部、卫生部在内的几部门对我国各个省市的青少年儿童进行了体质调查。这次调查结果，使我国初步了解了该阶段青少年儿童的身体形态、身体机能、身体素质的现状，并制定了相关的评价标准。[1]1985 年，我国又对全国青少年学生进行了大规模体质测试，测试的内容主要包括身体形态、身体素质、身体机能在内的 20 多个指标，而且这次测试还覆盖了少数民族地区，填补了我国在这方面的空白。在随后的 1991 年、1995 年、2000 年、2005 年、2010 年，又分别对全国的青少年学生进行大规模的体质测试，并且不断的完善体质测试系统，学生体质的测试经过这几年不断的实践和改进，逐步的走向规范化。[2]国家体育总局和教育部，根据我国青少年的体质状况，于 2001 年又制定出了《学生体质健康标准》，并决定 2002 年的第一学期，在全国各中小学实施。这个标准的实施，表明我国在青少年学生的体质研究方面更加系统，更加成熟。

近二十年，随着社会的发展，生活方式的改变，应试教育的影响，多数青少年对体育锻炼的不重视，致使我国青少年学生的体质状况不断下降为扼制这种状况的进一步发展。2007 年，教育部和共青团联合下发了《关于开展亿万学生阳光体育运动的决定》，让阳光体育运动走进每一所学校。[3]其目的是改善青少年体质现状。

与此同时，我国的科研工作者也在青少年体质健康方面做了很多研究工作，并对引起青少年体质下降的原因进行了分析，取得了一批有价值的研究成果。如：徐元玉等的研究认为：当代大学生在身体形态方面的超重和肥胖，主要与营养过剩有关。身体机能的下降，主要与不经常参加体育锻炼有关。[4]廖文科的研究显示，反映学生身体形态的身高，在过去一段时间存在城乡差异，乡村学生的身高增长幅度大于城市，反映身体机能的肺活量在过去也存在城乡和性别差异，女生的下降幅度大于男生，乡村学生的下降幅度大于城市。[5]尹小俭对 1985、1991、1995、2000、2005 和 2010 年全国学生体质健康调查数据进行研究，结果显示：男女大学生超重及肥胖的比例逐年上涨，主要原因是大学生不良的生活方式和不经常参加体育锻炼造成的。[6]

2.4 山区体育教育现状

大量的研究结果表明，目前我国农村体育发展明显滞后于城镇，而山区学校的情况更加显著。主要表现在以下几方面：(1)师资力量薄弱，专职体育教师人数稀缺，距离农村体育教育的需求

[1] 何仲恺.体质与健康关系的理论与实证研究[D].北京体育大学博士论文.2001,04.
[2] 李玉强.青少年学生生活习惯与体质健康的调查及实验研究[D].华东师范大学硕士论文.2010,05.
[3] 史儒林.青海高原地区大学生生活方式和体质健康状况的调查研究[D].北京体育大学硕士学位论文.2005,07.
[4] 徐元玉,翟芳,周烈.当代大学生健康状况的调查与分析[J].安徽体育科技.2006,27(2):68-71.
[5] 廖文科,中国 7-18 岁汉族学生体质与健康动态变化和综合评价研究[D].中南大学博士论文.2009,05.
[6] 尹小俭,季成叶,王树明.我国大学生肥胖流行现状及体质变化趋势[J].成都体育学院学报.2009,35(1):65-68.

仍有较大的差距；(2)学校体育的经费和仪器投入不足，导致很多和学校体育有关的运动项目无法进行；(3)有关体育教学的课程设置不合理，达不到国家规定的素质教育的要求；(4)体育课时不能满足《课程标准》的要求，且教学形式比较单一，学生的课外体育活动时间得不到保证；

造成山区体育教育落后的原因有，体育课在学校不被领导和老师重视，责任不明确；学生在校期间没有专职老师组织学生进行课外体育活动；山区学校的专职体育教师数量严重短缺；和体育有关的场地器材配备不足等等。学校体育课开展的好坏直接影响学生体质健康状况，王德平在对甘肃省中小学的体育现状进行抽样调查和分析中发现，农村学校的体育师资、体育经费和体育器材都很缺乏。[1]钟全宏在对某贫困地区的中小学体育教师队伍调查发现，贫困山区体育教师的年龄参差不齐教学能力和待遇都比较低。[2]

综上可以看出，我国山区学校体育发展水平总体低下，产生此种原因的因素是经济水平低下，体育师资力量薄弱，学校体育观念落后。本研究认为，农村学校体育发展水平的总体低下与体育教师有着密不可分的关系，面对总总不如意的生活和环境，大多体育老师没有积极的面对，这就使体育在学校尤其是基层学校的地位越来越低，体育教师的各方面能力也无法提高。因此，作为山区学校体育教师，应该能动地发挥自身的才能，把自己在学校所学习的知识有声有色的交给学生和老师，用自己的行动来影响周边的教师和校领导，只有这样才能真正地促进学校体育发展，才有可能得到学校领导的重视，从而改善学校体育的开展，同时也为国家教育大计献上微薄之力。

[1] 王德平等.甘肃省学校体育教学现状与发展对策[J].兰州大学学报 2000(12).
[2] 钟全宏.西北贫困民族聚居区学校体育师资队伍现状的调查分析[J].首都体育学院学报.2009(01).

3 研究假设

地理位置的特殊，经济条件的限制，不注重营养搭配，缺少体育锻炼等都是影响山区儿童体质的因素，通过健康教育干预、加强体育锻炼、养成良好的生活习惯对改善山区儿童体质健康状况会起到一定的作用。

4 研究对象与方法

4.1 研究对象

以山区小学生为调查和实验对象，对山区儿童体质健康状况和运动与健康教育联合干预的效果等相关问题进行研究。

4.2 研究方法

4.2.1 文献资料法

充分搜集、整理和研究国内外文献资料，熟悉了解本研究的研究现状和前沿动态，理清本研究的研究方向和研究线路。

4.2.2 问卷调查法

根据研究需要，自行设计调查问卷，了解山区儿童的生活、行为方式，体育锻炼情况以及对健康知识的知晓程度。

问卷由本人进行现场发放和现场回收，调查内容主要包括个人基本信息(性别、出生年月、年级、是否独生子女)、患病史、生活习惯、家庭经济状况，参加家务劳动、营养与饮食和体育锻炼、心理等方面的内容（详见附件)。

4.2.3 实验法

(1)实验对象及其分组

在学生、家长知情、同意、自愿的情况下，在万荣县万泉小学，筛选出无遗传性或营养性疾病的儿童 92 名，年龄 9-12 岁（小学四、五年级)。按照性别、年龄分层法将 92 名儿童随机分为三组：对照组、运动锻炼干预组、运动和健康教育联合干预组。其中对照组 32 人、运动干预组 30 人、运动+健康教育联合干预组 30 人。各组在年龄、性别、身高、体重、健康状况方面无显著差异（表 4-1)。

表 4-1 实验对象的基本情况及分组 *(M±SD)*

	n	年龄（岁）	年级	身高（cm）	体重（kg）	健康状况
对照组	32	10.91±0.86	四、五年级	139.63±4.25	34.05±3.91	良好
运动组	30	10.93±0.74	四、五年级	139.71±4.42	32.34±4.36	良好
运动和健康教育组	30	11.07±0.91	四、五年级	140.20±4.29	34.06±5.70	良好

（2）干预措施

①运动干预

根据研究对象的实际情况，为运动干预组和运动+健康教育联合干预组制定出相应的运动处方。

运动项目：根据实验对象所处年龄阶段特点、兴趣爱好及所在学校场地和器材等实际情况，

本研究所选择的运动项目主要有跳绳、篮球游戏、跑步及拉伸活动。

运动强度：每次运动时脉搏保持在 120-140 次/分钟。

运动持续时间：30-40 分钟/次。

运动频度：每周 3 次。运动干预组和运动和健康教育联合干预组除去体育课外，放学后集中在学校操场上进行体育锻炼。锻炼的时候多加入学生们感兴趣的体育锻炼项目，这样可以保证学生参加活动的积极性。对照组除上体育课外，不额外安排其他体育活动。

运动中应当注意的问题：锻炼过程中加强医务监督和保护帮助，观察受试者的反应，发现异常时立刻停止运动。出现异常时及时停止运动。

②健康教育干预

a 开设健康教育课，向运动和健康教育联合干预组学生传授相关知识。

b 饮食营养监督。

c 不良生活方式纠正。

d 帮助学生养成良好的生活行为习惯。

（3）效应指标

①形态测试：

指标包括：身高、体重、胸围、腰围、臀围、皮褶厚度。

腰围：反映腹腔内脂肪堆积程度，测试时受试者站立，双臂适当张开下垂，双脚合并，使体重均匀分担于双脚，露出腹部皮肤；测量时平缓呼吸，不收复或屏气，测量在肚脐上缘水平面进行；尺刻度下缘距肚脐上缘 1cm，水平环绕一周；皮尺贴近皮肤，但避免紧压而使皮尺陷入皮内。读数以 cm 为单位，精确到小数点后一位。

臀围：指臀部的最大围度，测试时受试者身穿单薄长裤，自然站立，两臂下垂并适度张开，双脚并拢，臀部放松，将卷尺置于臀部向后最突出部位，水平绕臀一周；皮尺紧贴皮肤，勿压软组织；在受试者平静呼气时读数。读数以 cm 为单位，精确到小数点后一位。

胸围：测试时受试者安静站立，两臂下垂，均匀呼吸。用卷尺围绕胸廓，背部置于肩胛骨下角下缘，前面经乳头下缘，若受试者为乳房开始发育少女，则放置胸前锁骨中线第四肋骨处，测试平静状态下的胸围，读数以 cm 为单位，精确到小数点后一位。

皮褶厚度：指局部的皮下脂肪厚，测试时受试者自然站立；测量者右手持皮褶厚度计，张开钳臂；左手拇、食间保持约 3cm 距离，将该处皮肤（含皮下组织如图：4-1）捏紧提起，将皮褶钳在该捏起处嵌入约 1cm；放开活动把柄，按指针数读数，单位 mm，共测试三次，取中间值或两次相同的值。测试部位：肱三头肌皮褶厚度、腹部皮褶厚度。

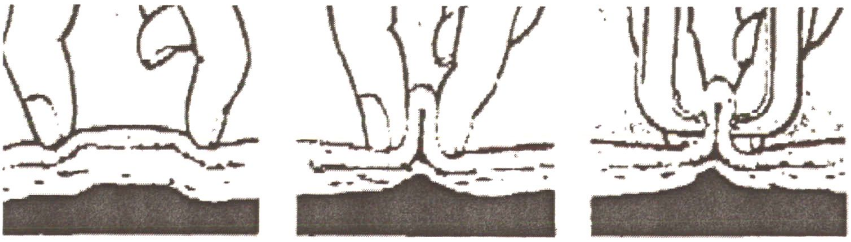

图 4-1 皮褶法[1]

②身体素质测试：

采用国家体育总局规定的体质监测器材，根据 2010 年国民体质监测工作要求， 测试指标：握力、背力、坐位体前屈、50 米 跑、立定跳远和 50 米×8 往返跑等五项指标，分别作为衡量儿童力量素质、柔韧素质、速度素质、耐力素质好坏的依据。

测试仪器主要包括：坐位体前屈测试仪、握力计、皮尺以及秒表等。具体测试方法如下：

（1）坐位体前屈测试。测试方法为：受试者两脚分开 5-10cm，与平台前言横线平齐；两脚跟并拢，双腿伸直；上体尽量前屈，双臂及手指伸直；两手并拢，用中指指尖轻推游标下滑（不得有突然下振动作），直到不能继续下伸为止。读取小数点后两位。

（2）握力测试 。测试方法为：受试者两脚自然分开成直立姿势，两臂自然下垂，计量盘面朝外一手持握力计全力紧握，手臂不能来回摆动，不能靠腰或其他物体借力。右手（或利手）连测三次（适当间隔休息），取最大值读数。

（3）背肌力测试 测试方法：测试时受试者先做充分准备活动（重点是腰部）；站在踏盘上，双足稍分开，躯干略向前倾；测试者调节链条长短，使其握柄和受试者的双膝等高；双臂、双腿伸直，尽最大力量向上牵拉，连续测三次取最大值。

（4）50 米直线跑 50 米直线跑目的是测试学生速度、灵敏素质及神经系统灵活性的发展水平。测试方法受试者两人一组，站立起跑，受试者听到“跑”的口令后开始起跑，不得抢跑。成绩以秒为单位，取小数后一位。

（5）立定跳远 立定跳远目的是测试学生下肢爆发力及身体协调能力的发展水平。测试方法：要求受试者两脚自然分开站立，站在起跳线后，脚尖不得踩线。两脚原地同时起跳，不得有垫步或连跳动作。每人试跳三次，记录其中成绩最好一次，以 cm 为单位不计小数。

（6）50 米×8 往返跑 测试方法：采用 50 米直线跑道，起点线前 0.5 米和 49.5 米处各立一标杆，杆高 1 米，立于跑道正中。要求受试者 2 人一组进行测试。用站立式起跑，当听到“跑”口令后开始起跑，往返 4 次，往返跑时就逆时针方向绕过标杆，不得碰扶标杆，不得串道，当受试者躯干部到达终点线的垂直面时停表。先按分秒登记成绩，再将分换算成秒不计小数。

③心肺功能指标：

肺活量 测试方法：受试者直立，一手握通气管；头略后仰，尽力深吸气（至再不能吸为止）后憋住，将嘴对准吹嘴做一次性尽力深呼气（至不能呼为止），测量两次，取最大值，单位 ml，

[1] 体育测量与评价编写组. 体育测量与评价[M].北京体育学院出版社.1986,1:123.

不计小数。

血压 测试方法：测试时受试者肘部、血压计、心脏放在同一水平；

所有指标均采用实验前测与实验后测

实验前测试 → 干预 12 周 → 实验后测试

4.2.4 数据的统计与处理

使用 Excel 工作表把所测数据初步统计并绘制图表，所获数据均以平均值±标准差*(M±SD)*表示，所有数据均用 Excel2003 和 spss17.0 统计学软件进行处理。实验各组干预前后以及干预后的各组之间的变化采用单因素方差分析，显著水平取 $p<0.05$。

5 结果与分析

5.1 实验对象所在地的基本情况

本实验样本来源于山西省万荣县万泉小学。万泉乡位于运城市万荣县城正南 15 公里，地处孤峰山之荫，总面积 44.15 平方公里，海拔约 1000 米，全乡人口 15693 人，2011 年的人均收入约为 2000 元。境内山势起伏，沟壑纵横，地势南高北低，耕地多呈梯形，总耕地面积 42517 亩，以盛产大葱、酥梨、苹果、蔬菜、小麦为主。万泉小学地处于万泉乡万泉村，全校共设六个年级，七个班（六年级两个班），共有学生 350 人，教师 21 人（没有专职体育老师）。该校为寄宿制学校，学校在体育场地和器材方面比较缺乏。学生上体育课的场地为万泉村的广场，体育器材只有篮球架、水泥乒乓球台、跳绳。

5.2 运动和健康教育干预对山区小学生日常饮食习惯的影响

表 5-1 运动和健康教育联合干预前后学生饮食习惯的变化

题项	选项	对照组		运动干预组		运动和健康教育干预组	
		实验前	实验后	实验前	实验后	实验前	实验后
是否有吃早餐的习惯	是	32（100）	32（100）	30（100）	30（100）	30（100）	30（100）
	否	0（0）	0（0）	0（0）	0（0）	0（0）	0（0）
有吃零食的习惯吗	是	19（59）	19（59）	18（60）	18（60）	17（57）	16（53）
	否	13（41）	13（41）	12（40）	12（40）	13（43）	14（46）
一日吃几餐	两餐	0（0）	0（0）	0（0）	0（0）	0（0）	0（0）
	三餐	23（72）	23（72）	24（80）	24（80）	25（83）	27（90）
	三餐以上	9（28）	9（28）	6（20）	6（20）	5（17）	3（10）
早餐以什么为主	鸡蛋	8（25）	8（25）	8（27）	8（27）	6（20）	6（20）
	牛奶	6（19）	6（19）	6（20）	6（20）	6（20）	6（20）
	豆浆	0（0）	0（0）	0（0）	0（0）	0（0）	0（0）
	馒头和稀饭	18（56）	18（56）	16（53）	16（53）	18（60）	18（60）
喜欢吃哪些零食	糖果类	7（22）	7（22）	6（20）	6（20）	7（23）	8（27）
	麻辣片	18（56）	18（56）	17（57）	17（57）	19（63）	15（50）
	干果类	4（13）	4（13）	4（13）	4（13）	3（10）	4（13）
	甜点类	3（9）	3（9）	3（10）	3（10）	1（4）	3（10）
午餐以什么为主	米饭	5（15）	5（15）	4（13）	4（13）	3（10）	3（10）
	面食	6（19）	6（19）	5（17）	5（17）	8（27）	8（27）
	馒头	21（66）	21（66）	21（70）	21（70）	19（63）	19（63）
晚餐以什么为主	米饭	6（19）	6（19）	5（17）	5（17）	4（14）	4（14）
	面食	5（15）	5（15）	4（13）	4（13）	7（23）	7（23）
	馒头	21（66）	21（66）	21（70）	21（70）	19（63）	19（63）

注：（）中的数据为百分比（%）

表 5-1 可以看出通过对山区儿童的调查得知，三组被试对象干预前分别有 72%、80%、80%的学生一天三餐，对照组有 28%的学生有晚上加餐的习惯，运动干预组与运动和健康教育联合干预组有 20%的学生有晚上加餐的习惯，早餐方面三组学生大部分都已馒头和菜为主，这主要与饮食的区域性有关，由于被试学生籍贯都是我国北方地区，当地大部分人口都是以馒头为主食，加上受试学生均为寄宿制，在同一食堂用餐，食堂饭样较少，从而影响了学生的主食选择性。在零食的选择方面三组学生大部分喜欢吃麻辣片类的零食，这主要是由于山区经济条件的限制，麻辣片相对于其他零食要便宜，但麻辣片里含有大量对身体不健康的食品添加剂，营养价值不大并且长期食用会对肠胃造成极大的伤害，另外学生由于选择余地较少，加上大部分学生对零食的利弊没有清晰的认识，故出现这样的结果。由于被试学生所在的学校是全封闭寄宿制学校，中午的正餐和晚餐都是在学校食堂吃的，同时因为食堂饭菜的局限性，所以导致学生饮食的选择范围也是有限的。

饮食习惯是人类在长期的生活中形成的，受多种客观因素的影响如经济条件、区域特点等，所以干预前后对照组和运动干预组在饮食习惯和饮食种类方面都没有变化，运动和健康教育联合干预组在零食方面有细微的变化主要表现为喜欢吃麻辣片的学生由干预前的 57%降到 50%，这可能与干预的过程中给学生讲解麻辣片对身体的危害有关。

5.3 运动和健康教育联合干预对山区小学生参加体育锻炼活动的影响

表 5-2 运动和健康教育联合干预前后学生参加体育锻炼活动的变化

题项	选项	对照组		运动干预组		运动和健康教育干预组	
		实验前	实验后	实验前	实验后	实验前	实验后
喜欢上体育课吗	喜欢	25（78）	24（75）	25（83）	27（84）	23（77）	28（93）
	不喜欢	7（22）	8（25）	5（17）	3（16）	7（23）	2（7）
是否经常参加课外体育活动	是	15（47）	15（47）	13（47）	18（62）	15（50）	22（73）
	否	17（53）	17（53）	17（53）	12（38）	15（50）	8（27）
是否知道科学的体育锻炼方法	是	0（0）	0（0）	0（0）	15（50）	0（0）	23（77）
	否	32（100）	32（100）	30(100）	15（50）	30（100）	7（23）
你觉得体育锻炼对你的生活学习有好处吗	有	12（38）	12（38）	11（37）	16（53）	12（40）	16（53）
	没有	5（15）	5（15）	5（16）	3（10）	5（17）	3（10）
	不清楚	15（47）	15（47）	14（47）	11（37）	13（43）	11（17）
每周参加体育活动的次数	不参加	5（16）	5（16）	5（16）	3（10）	5（17）	1（3）
	一次	19（59）	19（59）	17（57）	15（50）	17（57）	14（47）
	两次	8（25）	8（25）	8（27）	12（40）	8（26）	15（50）
	两次以上	0（0）	0（0）	0（0）	0（0）	0（0）	0（0）
每次参加体育活动的时间	30 分钟以下	21（66）	21（66）	19（63）	14（47）	20（70）	14（47）
	30-60 分钟	11（34）	11（34）	11（37）	16（53）	10（30）	16（53）
	60 分钟以上	0（0）	0（0）	0（0）	0（0）	0（0）	0（0）
你见过城里的小朋友上体育课吗	见过	5（16）	5（16）	4（13）	7（23）	5（17）	9（30）
	没见过	27（84）	27（84）	26（87）	23（77）	25（83）	21（70）

注：( ) 中的数据为百分比（%）

由表 5-2 可以看出，干预前对照组、运动干预组、运动和健康教育联合干预组对体育课的喜欢程度分别为 78%、83%、77%，无显著性差异（$p>0.05$），说明山区儿童对体育课有极大的兴趣，大部分都愿意积极参与到体育课中去。12 周的运动和健康教育联合干预后，运动干预组、运动和健康联合干预组与干预前相比有所增加，对照组在干预前后没有多大变化，这主要与在干预过程中给学生增加的体育游戏有关。三组学生分别有 47%、47%、50%的学生能够做到经常参加体育活动，仅有 37%、38%、40%的学生知道体育锻炼对生活和学习有极大的帮助，说明只有部分学生了解体育锻炼在日常生活中的重要性。12 周干预后，运动干预组与运动和健康教育联合干预组经常参加体育活动和了解体育锻炼对日常生活重要性的人数都有大幅增加，与干预过程中给他们讲解体育锻炼对人体的重要性有关。此外，实验前三组学生没有一个人清楚科学的体育锻炼方式，说明山区学生体育知识的缺乏，与学校在体育基本知识的宣传方面做不够有关。12 周干

预后，运动干预组、运动和健康教育联合干预组学生对科学的体育锻炼有了很大程度的了解，一方面与干预过程中实施的科学体育锻炼实践有关，另一方面与给他们讲解科学体育锻炼的方法有关。

5.4 运动和健康教育联合干预对山区小学生心理健康状况的影响

表 5-3 运动和健康教育联合干预前后学生心理健康状况的变化

题项	选项	对照组		运动干预组		运动+健康教育干预组	
		实验前	实验后	实验前	实验后	实验前	实验后
见了认识的长辈	主动热情打招呼	13（41）	13（41）	12（40）	13(43)	13（43）	15（50）
	不好意思	12（40）	11（34）	13（43）	11(37)	13（43）	9（30）
	躲起来	7（19）	8（25）	5（17）	6（20）	4（14）	6（20）
和新同学在一起玩	主动邀请一起玩	12（38）	11（34）	12（40）	11(37)	14（47）	18（60）
	不好意思接近	5（15）	7（22）	5（17）	7（23）	5（16）	4（13）
	只顾自己玩	15（47）	14（44）	13（43）	12(40)	11（37）	8（27）
上课不敢发言，即使知道答案	偶尔	11（34）	11（34）	12（40）	12(40)	12（40）	13（43）
	没有	5（16）	5（16）	3（10）	3（10）	3（10）	6（20）
	经常	16（50）	16（50）	15（50）	15(50)	15（50）	11（37）
和同学在一起时，你感到	快乐	20（63）	18（56）	21（70）	22(73)	21（70）	23（77）
	拘谨	8（25）	9（28）	6（20）	5（17）	6（20）	5（17）
	不合群	2（6）	2（6）	2（7）	2（7）	2（7）	1（3）
	孤独	2（6）	3（10）	1（3）	1（3）	1（3）	1（3）
期末考试时	心情紧张	15（47）	15（47）	15（50）	15(50)	15（50）	11（37）
	有点紧张	8（25）	9（28）	8（27）	8（27）	7（23）	8（27）
	无所谓	4（13）	5（16）	4（13）	4（13）	4（13）	4（13）
	有信心，不紧张	5（15）	3（9）	3（10）	3（10）	4（14）	7（23）

注：（）中的数据为百分比（%）

从表 5-3 可以看出，山区学生在性格、自信心、胆量方面不及城里的学生。心理健康情况调查结果显示，当和新同学在一起时，对照组、运动干预组、运动和健康教育联合干预组三组的学生分别仅有 38%、40%、47%的学生主动邀请新同学一起玩；见了认识的长辈后，三组学生主动上前打招呼的不到一半，这些数据说明大部分山区学生性格偏于内向，见到新同学和长辈时会比较拘谨、放不开及不敢表达自己，甚至有个别学生会出现排斥现象，只有和认识的同学在一起时才比较放松自在；12 周的运动+健康教育干预后，对照组和运动干预组干预前性格方面没有多大变化，而运动和健康教育联合干预组愿与同学主动交往的人数由干预前的 47%增加到 60%，这与干预过程中给同学们灌输如何与人进行交际方面的知识有关。上课回答问题方面，三组学生在干预前有将近一半的人知道答案不敢回答，期末考试方面，三组学生有一半心情紧张。这从侧面

反映了大部分山区学生对自己没有足够的自信心和勇气，在考试临近时容易出现焦虑现象，同时揭示出教师在这方面的引导和教育是不足的，不能帮助和引导学生建立自信心和勇气以及及时排解忧虑情绪；经过 12 周的运动和健康教育联合干预后，对照组和运动干预组干预前后没有多大变化，运动和健康教育联合干预组的学生在这两方面有好的表现，如见到认识的长辈能够主动打招呼的人数由干预前的 43%上升到 50%，期末考试紧张的人数由干预前的 50%降到 37%，这与在健康教育的过程中向学生灌输如何提高勇气和自信心的知识有关。

5.5 运动和健康教育联合干预对山区儿童身体形态结构的影响

5.5.1 运动和健康教育联合干预对山区儿童身高、体重和 BMI 指数的影响

表 5-4 运动和健康教育联合干预对山区儿童 BMI 指数的影响（*M±SD*）

	n	身　高　(cm)		体　重　(kg)		BMI(kg/m²)	
		实验前	实验后	实验前	实验后	实验前	实验后
对照组	32	139.63±4.25	140.03±4.28	34.05±3.91	34.19±3.95	17.42±1.70	17.42±1.74
运动组	30	139.71±4.42	140.38±4.82	32.34±4.36	32.14±4.32▲	17.45±1.68	16.39±1.29*▲
运动和健康教育组	30	140.20±4.29	140.86±4.17	34.06±5.70	33.42±5.44▲	17.71±2.73	16.35±2.22*▲

注：　各组实验前后比较：*P<0.05；**P<0.01。

实验后不同组别之间的比较。与对照组比较：▲P<0.05；▲▲P<0.01；

与运动组比较：●P<0.05；●●P<0.01

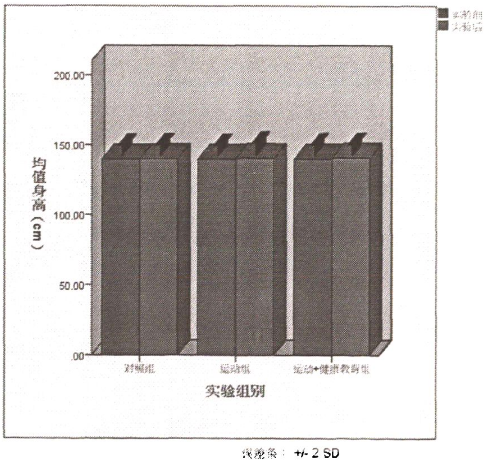

图 5-1 实验前后各组身高的变化

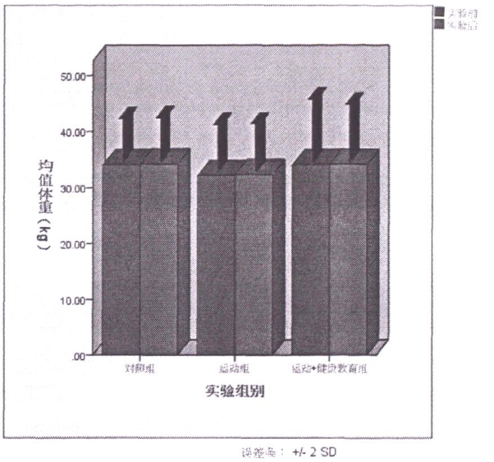

图 5-2 实验前后各组体重的变化

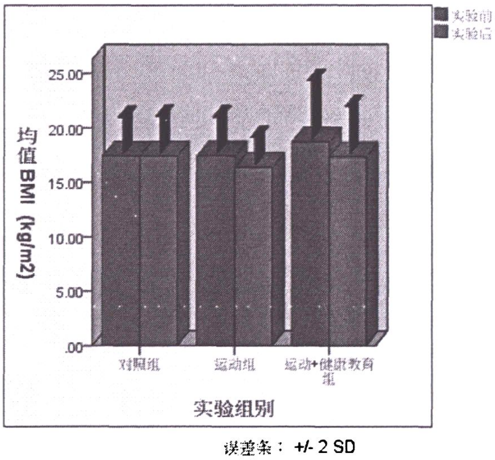

图 5-3 实验前后各组 BMI 的变化

如表 5-4、图 5-1、图 5-2、图 5-3 所示，实验前后对照组、运动干预组、运动和健康教育联合干预组学生身高均有一定程度的增高，但三组之间相比无显著差异（p＞0.05），这与儿童正处于生长发育期有关。从体重指标看，三组实验前后变化均不大，均在正常范围内，但运动干预组与运动和健康教育联合干预组与对照组相比，体重的增加较为缓慢，其中运动干预组、运动和健康教育联合干预组与对照组相比有显著差异（p＜0.05），这可能与运动干预组与运动和健康教育联合干预组运动过程中热量消耗较大有关，提示运动、运动和健康教育联合干预在一定程度上对预防儿童体重过快增长具有一定的作用。从 BMI 指数指标看，对照组、运动干预组、运动和健康教育联合干预组三组学生实验前后均在正常范围之内，虽然运动干预组、运动和健康教育联合干预组与对照组相比有一定程度的下降，并出现显著差异（p＜0.05），但对正处于生长发育时期的儿童来说意义不大。

5.5.2 运动和健康教育联合干预对山区儿童胸围、腰围、臀围、WHR 的影响

表 5-5 运动和健康教育联合干预对山区儿童围度的影响（*M*±*SD*）

	n	胸围（cm）		腰围（cm）	
		实验前	实验后	实验前	实验后
对照组	32	65.88±4.06	66.06±4.12	60.89±3.97	60.56±4.16
运动组	30	65.18±4.01	65.18±3.99	60.27±4.00	59.88±4.21
运动和健康教育组	30	66.58±4.07	67.05±4.14	61.93±4.08	61.33±4.11

表 5-6 运动和健康教育联合干预对山区儿童围度的影响（*M*±*SD*）

	n	臀围（cm）		WHR	
		实验前	实验后	实验前	实验后
对照组	32	72.94±3.58	73.08±3.52	0.78±0.04	0.78±0.05
运动组	30	73.33±4.20	73.52±4.11	0.81±0.05	0.80±0.06
运动和健康教育组	30	74.60±4.97	74.97±4.89	0.80±0.05	0.79±0.06

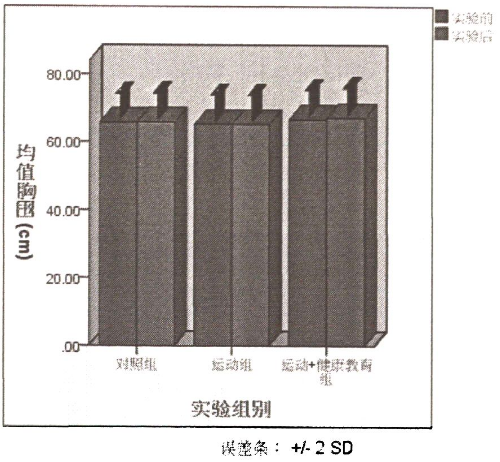

图 5-4 实验前后各组胸围的变化

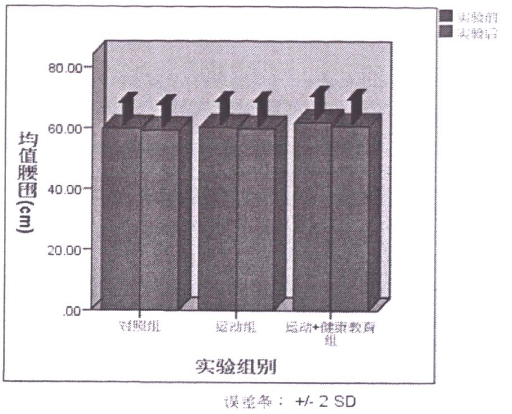

图 5-5 实验前后各组腰围的变化

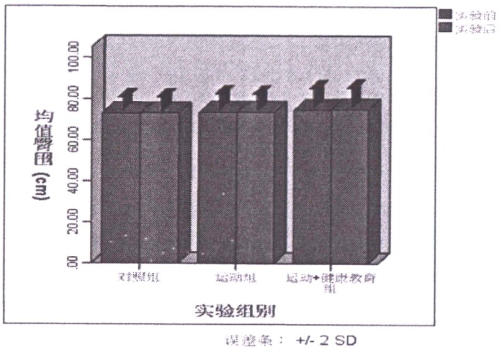

图 5-6 实验前后各组臀围的变化

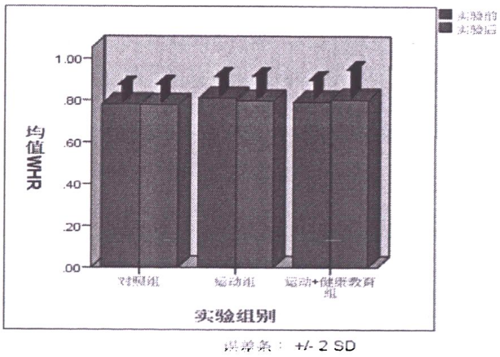

图 5-7 实验前后各组 WHR 的变化

如表 5-5、5-6，图 5-4、图 5-5、图 5-6 和图 5-7 所示，实验前后对照组、运动干预组、运动和健康教育联合干预组的胸围都有一定程度的增加，但三组之间相比无显著性差异（p＞0.05），这与儿童正处于生长发育期有关，胸围的增长除了胸廓的生长发育以外，还与脂肪的堆积有关。从腰围指标看，三组实验前后变化均不大，均在正常范围内，三组之间相比无显著性差异（p＞0.05），运动干预组、运动和健康教育联合干预组与对照组相比出现一定程度的下降，这可能与运动干预组、运动和健康教育联合干预组运动过程中消耗的脂肪较多有关。从臀围指标看，对照组、运动干预组、运动和健康教育联合干预组的臀围实验后都有一定程度的增加，但三组之间相

比无显著性差异（p>0.05），这与儿童正处于生长发育期有关。从 WHR 指标来看，对照组、运动干预组、运动和健康教育联合干预组三组学生实验前后均在正常范围内，运动干预组、运动和健康教育联合干预组与对照组相比有一定程度的下降，但未出现显著性差异（p>0.05）。提示运动锻炼、有明显的塑体健身作用。

5.5.3 运动和健康教育联合干预对山区儿童肱三头肌和腹部皮褶厚度的影响

表 5-7 运动和健康教育联合干预对山区儿童皮褶厚度的影响（M±SD）

	n	肱三头肌皮褶厚度（cm）		腹部皮褶厚度（cm）	
		实验前	实验后	实验前	实验后
对照组	32	2.16±0.27	2.14±0.32	2.42±0.38	2.44±0.35
运动组	30	2.12±0.36	2.08±0.32	2.42±0.42	2.37±0.51
运动和健康教育组	30	2.12±0.41	2.02±0.38	2.22±0.49	2.17±0.44

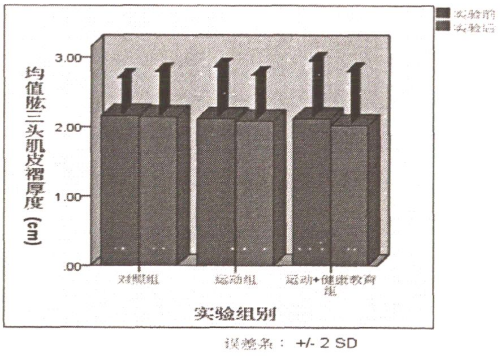

图 5-8 实验前后肱三头肌皮褶厚度的变化

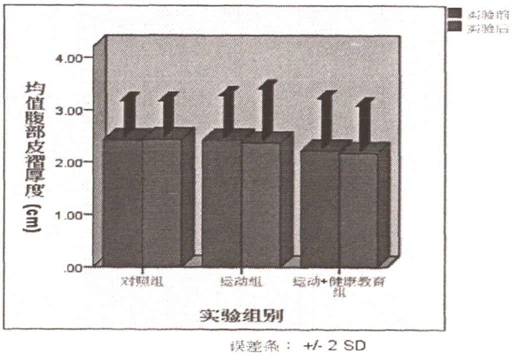

图 5-9 实验前后皮褶厚度的变化

如表 5-7、图 5-8、图 5-9 所示，实验前后对照组、运动干预组、运动和健康教育联合干预组的肱三头肌皮褶厚度都有一定程度的下降，三组之间相比没有显著性差异（p>0.05），这与儿童

正处于生长发育期有关，运动干预组、运动和健康教育联合干预组下降的程度大于对照组，这可能与运动干预组、运动和健康教育联合干预组运动时消耗掉多余的脂肪有关。从腹部皮褶厚度指标来看，实验后对照组的腹部皮褶厚度指标有一定程度的增加，可能与儿童处于生长发育期有关。运动干预组、运动和健康教育联合干预组的皮褶厚度在实验后与对照组相比出现一定程度的下降，但三组之间相比没有显著性差异（p＞0.05），这可能与山区儿童皮褶厚度本来就较薄有关。

5.6 运动和健康教育联合干预对山区儿童生理机能的影响

5.6.1 运动和健康教育联合干预对山区儿童安静状态下血压的影响

表 5-8 运动和健康教育联合干预对山区儿童血压的影响 *（M±SD）*

	n	收缩压（mmHg）		舒张压（mmHg）	
		实验前	实验后	实验前	实验后
对照组	32	103.13±6.69	100.88±6.23	68.75±6.35	65.22±5.94
运动组	30	103.77±6.13	101.17±5.83	67.50±5.53	66.43±4.14
运动和健康教育组	30	103.67±6.29	102.50±7.51	67.50±5.04	67.00±4.47

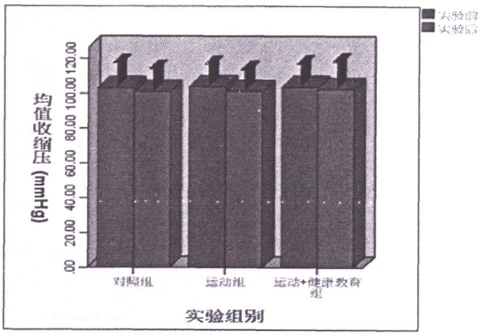

误差条： +/- 2 SD

图 5-10 实验前后收缩压的变化

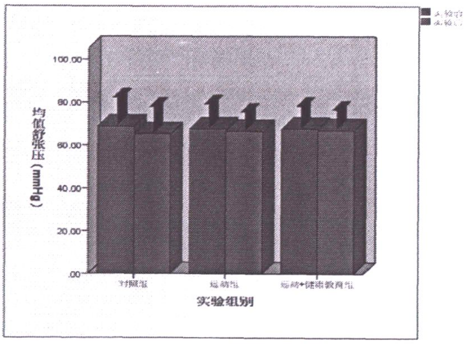

误差条： +/- 2 SD

图 5-11 实验前后舒张压的变化

如表 5-8、图 5-10、图 5-11 所示，实验前后对照组、运动干预组、运动和健康教育联合干预组的收缩压和舒张压变化幅度不大，三组之间相比没有显著性差异（p＞0.05）。这主要与三组儿

童血压值均在正常范围之内，运动、健康教育干预均不会引发血压出现较大的波动。

5.6.2 运动和健康教育联合干预对山区儿童肺活量的影响

表 5-9 运动和健康教育联合干预对山区儿童肺活量的影响（*M±SD*）

	n	肺活量（ml） 实验前	实验后
对照组	32	1992.81±346.99	2098.75±390.14 *
运动组	30	2159.67±316.30	2331.67±326.35 ** ▲▲
运动和健康教育组	30	2117.67±424.20	2395.33±460.03 ** ▲▲

注： 各组实验前后比较：*P＜0.05；** P＜0.01。

实验后不同组别之间的比较。与对照组比较：▲P<0.05；▲▲P<0.01；

与运动组比较：●P<0.05；●●P<0.01

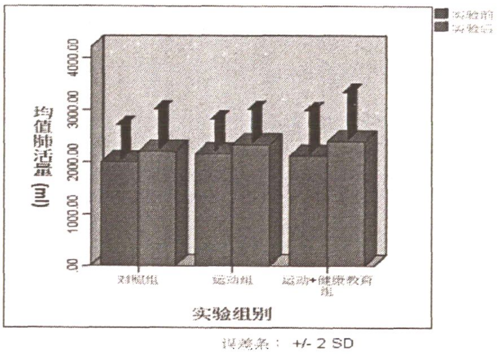

图 5-12 实验前后肺活量的变化

如表 5-9、图 5-12 所示，实验前后对照组、运动干预组、运动和健康教育联合干预组的肺活量都有一定程度的增加，其中运动干预组、运动和健康教育联合干预组与对照组相比差异性显著（p＜0.05），说明无论是单纯运动干预、还是运动和健康教育联合干预对提高儿童肺活量均能获得较好的效果。

5.7 运动和健康教育联合干预对山区儿童身体素质的影响

5.7.1 运动和健康教育联合干预对山区儿童力量素质的影响

表 5-10 运动和健康教育联合干预对山区儿童力量素质的影响（*M*±*SD*）

	n	握力（kg）		背力（kg）	
		实验前	实验后	实验前	实验后
对照组	32	16.05±2.38	16.04±2.43	43.34±5.85	44.25±5.37
运动组	30	16.08±2.89	17.60±2.97▲	44.30±9.61	48.33±7.44**▲▲
运动和健康教育组	30	16.22±3.19	18.03±3.68*▲	43.40±7.79	49.67±10.08**▲▲

注：　各组实验前后比较：*P＜0.05；** P＜0.01。

实验后不同组别之间的比较。与对照组比较：▲P<0.05；▲▲P<0.01；

与运动组比较：●P<0.05；●●P<0.01

表 5-11 运动和健康教育联合干预对山区儿童力量素质的影响（*M*±*SD*）

	n	跳远(cm)	
		实验前	实验后
对照组	32	131.06±12.50	133.22±14.53
运动组	30	132.23±14.77	139.77±17.74**▲▲
运动和健康教育组	30	132.70±16.80	143.67±15.23**▲▲•

注：　各组实验前后比较：*P＜0.05；** P＜0.01。

实验后不同组别之间的比较。与对照组比较：▲P<0.05；▲▲P<0.01；

与运动组比较：●P<0.05；●●P<0.01

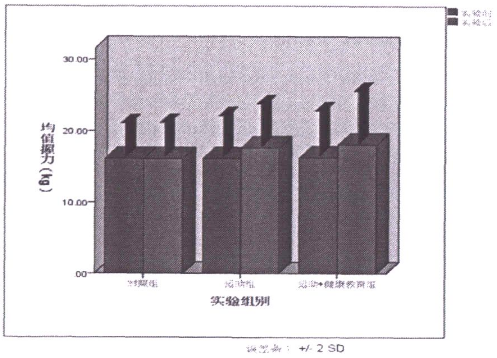

图 5-13 实验前后握力的变化

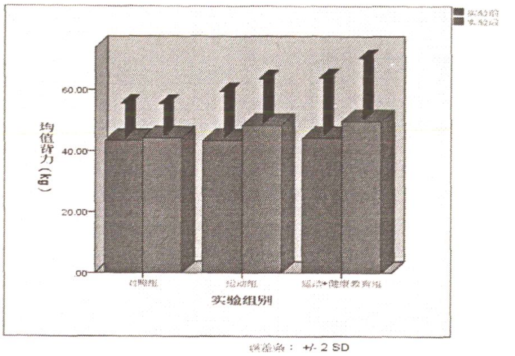

图 5-14 实验前后背力的变化

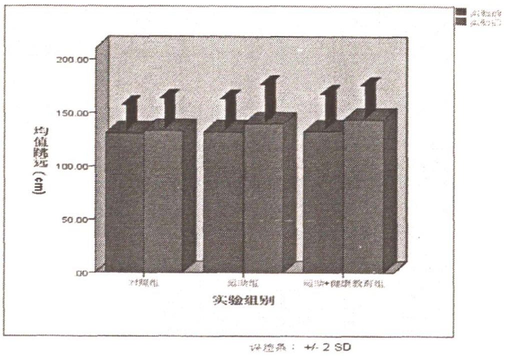

图 5-15 实验前后跳远成绩的变化

如表 5-10、5-11，图 5-13、图 5-14、图 5-15 所示，实验结束后运动干预组、运动和健康教育联合干预组的握力都有一定程度的增加，对照组则变化不大，运动干预组、运动和健康教育联合干预组与对照组相比差异性显著（$p<0.05$），运动和健康教育联合干预组与实验前相比差异显著（$p<0.05$），说明运动和健康教育增加前臂肌肉力量有显著作用。从背力指标来看，实验前后对照组、运动干预组、运动和健康教育联合干预组都有一定程度的增加，三组之间相比差异极其显著（$p<0.01$），但运动干预组、运动和健康教育联合干预组增加更为显著（$p<0.01$），说明运动和健康教育联合干预对促进儿童背力的增加有较好的效果，而对照组背力的增加可能与儿童自然生长发育有关。从反映下肢爆发力的指标跳远成绩来看，对照组实验前后跳远成绩变化不大，而运动干预组、运动和健康教育联合干预组提高幅度较大（$p<0.01$），且运动和健康教育联合干预组成绩优于单纯运动干预组，说明运动和健康教育联合干预对提高下肢爆发力效果更好。

5.7.2 运动和健康教育联合干预对山区儿童速度素质的影响

表 5-12 运动和健康教育联合干预对山区儿童速度素质的影响（*M±SD*）

	n	50 米跑（s）	
		实验前	实验后
对照组	32	9.21±0.61	9.17±0.65
运动组	30	9.21±0.60	8.89±0.67** ▲
运动和健康教育组	30	9.24±0.68	8.87±0.67** ▲

注： 各组实验前后比较：*P＜0.05； ** P＜0.01。

实验后不同组别之间的比较。与对照组比较：▲P<0.05;▲▲P<0.01;

与运动组比较：●P<0.05;●●P<0.01

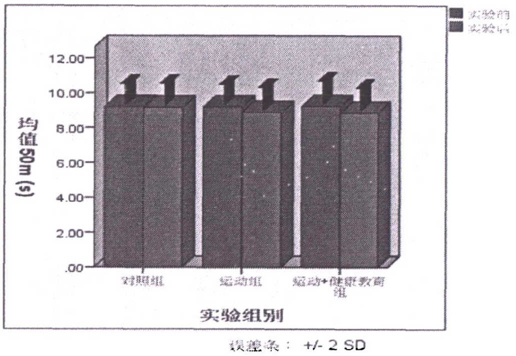

图 5-16 实验前后 50m 的变化

如表 5-12、图 5-16 所示，实验前后对照组 50m 跑成绩变化不大，运动干预组、运动和健康教育联合干预组显著提高（P＜0.01），且运动和健康教育联合干预组成绩好于单纯运动干预组，说明运动以及运动和健康教育联合干预对速度素质的发展具有显著效果。

5.7.3 运动和健康教育联合干预对山区儿童柔韧素质的影响

表 5-13 运动和健康教育联合干预对山区儿童柔韧素质的影响（*M±SD*）

	n	坐 位 体 前 屈（cm）	
		实验前	实验后
对照组	32	5.73±3.41	6.54±3.44
运动组	30	5.73±3.59	7.35±3.96** ▲
运动和健康教育组	30	5.51±3.75	6.95±3.64**

注： 各组实验前后比较：*P＜0.05； ** P＜0.01。

实验后不同组别之间的比较。与对照组比较：▲P<0.05;▲▲P<0.01;

与运动组比较：●P<0.05;●●P<0.01

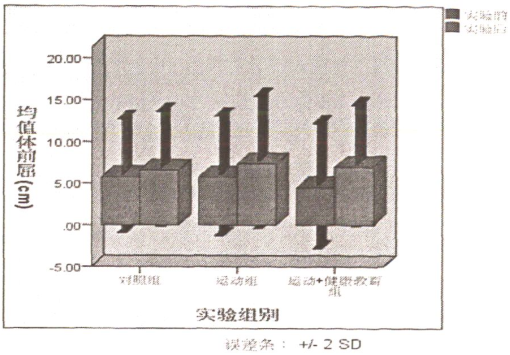

图 5-17 实验前后坐位体前屈的变化

如表 5-13、图 5-17 所示，实验前后对照组坐位体前屈成绩变化不大，而运动干预组、运动和健康教育联合干预组均有一定程度的提高（p<0.01），但运动干预组与运动和健康教育联合干预组相比无差异显著（p>0.05）。说明对发展柔韧素质而言，起主要作用的是运动干预，与运动锻炼促进关节的活动幅度和肌肉的力量增加有关。

5.7.4 运动和健康教育联合干预对山区儿童耐力素质的影响

表 5-14 运动和健康教育联合干预对山区儿童耐力素质的影响 *（M±SD）*

	n	50 米×8（s）	
		实验前	实验后
对照组	32	123.77±4.11	123.79±4.11
运动组	30	123.94±4.11	121.59±4.05** ▲▲
运动和健康教育组	30	124.24±4.02	121.11±3.96** ▲▲

注： 各组实验前后比较：*P<0.05；** P<0.01。

实验后不同组别之间的比较。与对照组比较：▲P<0.05；▲▲P<0.01；

与运动组比较：●P<0.05；●●P<0.01

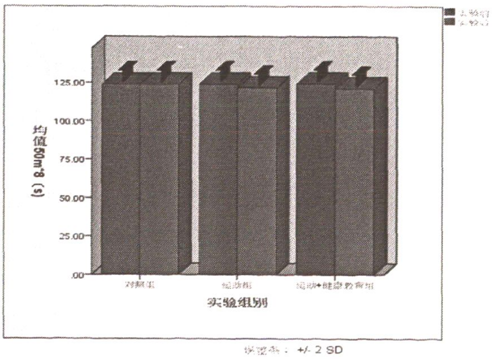

图 5-18 实验前后 50m×8 的变化

如表 5-14、图 5-18 所示，实验前后对照组 50m×8 成绩变化不大，运动干预组、运动和健康教育联合干预组的提高幅度较大（$p<0.01$），但运动干预组、运动和健康教育联合干预组之间差异不明显，说明在提高儿童有氧耐力素质方面，起作用的主要是运动干预。

6 讨论

6.1 运动和健康教育对山区儿童日常行为习惯和心理健康的影响

本研究中日常行为习惯主要包括饮食、生活习惯、参加课外体育活动的情况，本研究经过12周的运动+健康教育联合干预后，运动干预组、运动和健康教育联合干预组在饮食方面没有多大变化，这与他们所在区域的经济以及所在的学校性质（该校为寄宿制学校）有关。运动干预组、运动和健康教育联合干预组学生参加课外体育活动的情况要好于干预前，这是由于系统的体育锻炼不仅对学生体质发展有良好的作用，而且参加体育锻炼使他们心情舒畅，对他们的学习也有很好的帮助。运动和健康教育联合干预组的学生在生活习惯方面也好于干预前，这与健康教育的过程中给他们灌输有关健康生活行为习惯方面的知识，给他们讲解不良生活习惯对正处在生长发育阶段儿童的危害有关。

体育锻炼可以增强人的体质，对人的身体具有良好的促进作用，同时在参加体育锻炼的时候人的心理也会产生变化。进入21世纪，越来越多的研究者开始运用心理学的理论与方法，来揭示身体健康、体育锻炼和心理健康之间的相互关系。[1]有关体育锻炼与心理健康方面的已有大量的研究，如有研究证实体育锻炼对人的情绪有积极的作用，主要表现为焦虑水平和压力都有明显的降低。[2]Mclnman 等的研究表明 30 分钟的体育锻炼，可以使参与者的焦虑水平、紧张程度和心理紊乱的程度得到明显的改善，同时还可以使人心情愉快，精力充沛。[3]

本研究通过对受试对象进行为期12周的运动和健康教育联合干预后，对照组和运动干预组干预前后没有多大变化，运动和健康教育联合干预组学生在性格方面越来越开朗，主要表现为见认识的长辈主动上前 打招呼和主动邀请新同学一起玩的人越来越多，和同学在一起玩的过程中那种不合群、感到孤独的人越来越少、胆量和焦虑方面与干预前相比也有很大的提高，主要表现为考试之前紧张的人数变少了，有信心的人数增加了。一方面体育锻炼可以产生积极的情绪效应，降低焦虑水平。另一方面与健康教育干预过程中给他们讲解有关如何提高心理健康方面的知识有关。

6.2 运动和健康教育联合干预对山区儿童身体形态的影响

身体形态是指人体外部与内部的形态特征，通常用高度（身高、坐高、足弓高等），长度（身高、腿长、臂长手长、头长、颈长、足长），围度（胸围、臂围、腿围、腰围、臀围），宽度（头宽、肩宽、髋宽）和充实度（体重、皮脂厚度等）等来反映，可反映出人的身体发育状况在某些方面本质特征。

---

[1] 季浏，罗伯特.科克比.身体锻炼心理学的研究现状和未来方向[J].天津体育学院学报，1997，12(3):8-11.
[2] Weinberg, Jackson, Kolodny. The relationship of massage and exercise to mood enhancement[J].The Sport Psychologist, 1988, 2:202-221.
[3] Mclnman A D.Berger B G.Self-Concept and Mood Changes Associate with AerobicDance[J].AuJPsych, 1993, (45):134-140.

关于运动干预儿童少年身体形态方面的研究国内外学者已进行过多方面的研究。陈铮等[1]的研究证实：身体素质的提高与瘦体重增加和肌肉的发达程度有关；而皮下脂肪的增加,体重不仅会增加，肌肉的力量和速度也都会变小，从而影响身体的灵活性和协调性。邢文华[2](1984)曾在 14-15 岁青少年学生身体成分的研究报告中说,瘦体重以及瘦体重与身高的比值都与身体运动能力之间有密切的关系。郑晶[3]在运动干预对武汉市学龄儿童体质影响的研究中，主要选取身高、体重、皮褶厚度（上臂、腹部）等指标来对受试者身体形态的变化进行评价。12 周的运动干预后实验组受试者的体重、上臂的皮褶厚度与对照组相比变化显著（$p<0.05$），说明系统的体育锻炼可以消耗多余的脂肪从而使机体的重量和皮褶厚度降低。夏磊[4]的研究也证实系统的体育锻炼可以使机体的体重和皮褶厚度降低。王国志在对儿童少年体质影响的研究中，主要选取围度（胸围、腰围、臀围）、宽度（肩宽、骨盆宽）、长度也就是身高等指标对受试者的身体形态进行评价，他的研究证实长期的体育运动会对机体的围度和宽度起到良好的作用。[5]以上研究都是针对城市儿童，对山区儿童的研究比较少。

本研究主要是针对山区儿童，通过对被试对象的调查发现在饮食方面山区学生主要以馒头、菜、米汤为主，这与该地区处在中国的北方有关，北方地区的主食主要是馒头和面食为主。学校给学生菜的量和种类很少,菜主要以咸菜和豆芽菜为主,学校一星期让学生吃一次包子或者油饼。造成以上原因主要与山区经济条件的限制，学校的经费不足加上管理混乱等原因有关。本研究的受试对象所在学校为寄宿制学校，参加体育锻炼情况方面，学生每周两节体育课，由于没有真正的体育老师学生上课的时候就是自己玩，或者体育课被其它主课老师占据，除体育课外学生平时也没有时间参加体育活动。把表 5-4、5-5、5-6、5-7 的数据和 2010 年全国学生体质健康调研数据进行比较就可以看出,本研究的受试对象身体形态指标大部分指标都低于全国平均水平。虽然，教育部、国家体育总局、共青团中央联合发出声明，在全国各大、中、小学开展阳光体育运动，但是在很多地方，尤其是山区，大多数学生及家长、教师和校领导因受到传统观念的影响，对有关体育锻炼可以促进体质健康认识不足；还由于好多山区学校没有操场和专门的体育器械，也没有专业的体育教师，再加上小学生的认知水平和能力的限制，使课外体育锻炼活动在山区几乎成了空白。

经过 12 周的运动和健康教育联合干预后，从表 5-4、5-5、5-6、5-7 可以看出受试对象身体形态的各个指标都有了明显的改善。身高方面，对照组、运动干预组、运动+健康教育联合干预组的身高与实验前相比都有所增长，这主要与儿童正处于生长发育阶段有关，运动干预组和运动和健康教育联合干预组身高的增长比对照组的幅度大，这是由于儿童生长发育的同时，与运动干预组和运动和健康教育联合干预组学生锻炼过程中骨骺受到刺激有关。体重方面，经过 12 周的

[1] 陈铮,姚兴家,王金行,等.小学生身体成分与身体素质的研究[J].中国学校卫生,1994,15(1):5-6.
[2] 邢文华.14-15 岁男女生身体成份、机能和运动能力的研究[J].体育科学,1984.
[3] 郑晶.运动干预对武汉市学龄儿童体质健康的影响研究[D].华中师范大学,2012,5.
[4] 夏磊.运动干预对西安市小学生体质健康影响的实验研究[D].西安体育学院,2012,5
[5] 王国志.武术运动对少年儿童体质影响的实验研究[D].苏州大学,2001,05.

干预后，对照组与实验前相比没有多大变化，运动干预组、运动和健康教育联合干预组的体重与实验前相比有一定程度的下降，可能与运动过程中热量的消耗与身体肌肉增多有关。体重指数方面，经过 12 周的运动以及运动和健康教育联合干预后，实验前后对照组没有多大变化，运动干预组和运动和健康教育联合干预组与实验前相比有一定程度的降低，但这对正处于生长发育阶段的儿童来说意义不大。身体围度方面，经过 12 周的运动以及运动和健康教育联合干预后，胸围和臀围对照组与实验前相比没有多大变化，运动干预组、运动和健康教育联合干预组与实验前相比有一定程度的增加，这一方面儿童少年与正处于生长发育阶段有关，另一方面与运动过程中肌肉的增加以及热量的消耗有关。腰围方面，经过 12 周的运动和健康教育联合干预后，对照组的腰围在实验前后没有多大变化，运动干预组和运动和健康教育联合干预组的腰围有一定程度的降低，这与运动过程中热量以及脂肪的消耗有关。皮褶厚度方面，经过 12 周的及运动和健康教育联合干预后，对照组在干预前后没有变化，运动干预组、运动和健康教育联合干预组的皮褶厚度有一定程度的降低，这与运动过程中脂肪的消耗有关，差异不明显与山区学生皮下脂肪本来就薄有关。

6.3 运动和健康教育联合干预对山区儿童生理机能的影响

心肺机能是用来评价人体有氧能力的重要指标。良好的心肺机能可以增强人体的运动能力，也可以提高平时学习和工作的效率。[1]本实验主要选取肺活量和血压两项指标对心肺机能进行评价。

本研究测试之前对学生的调查得知，在山区，由于专业体育教师的缺乏学校没有健康教育这方面的老师，都是其他主课带上导致学生在健康教育这方面的知识面很窄，对基本的健康常识如肺活量、血压、脉搏这些生理机能一无所知。由表 5-9 可以看出，干预前学生肺活量远远低于 2010 全国学生体质健康调研平均水平。

经过 12 周的运动和健康教育联合干预后，运动干预组与运动和健康教育联合干预组学生肺活量不仅要远高于实验前的成绩，也大大高于对照组的数据，而对照组实验前后变化不大。一方面主要由于体育锻炼的效果加之山区学生平时的生活习惯，他们平时放假去地里帮家长干一些力所能及的农活，也喜欢结伴去爬山。另一方面，在运动和健康教育联合干预的过程中，让学生掌握呼吸的方法，让他们学会深呼吸的方法尤其是运动过程中“极点”的时候更要有意识的进行深呼吸，这可以减轻“极点”的反应，推迟“极点”的出现，促使第二次呼吸的提早到来。这些对肺活量的提高都有一定的作用。从表 5-8、图 5-10、5-11 可以看出，12 周的运动和健康教育干预后，对照组、运动干预组、运动和健康教育联合干预组学生血压的变化不大均处在正常范围内，这与他们处于生长发育阶段有关系。

6.4 运动和健康教育联合干预对山区儿童身体素质的影响

身体素质是反映人体运动能力好坏的重要指标，他好坏不仅影响人体的运动能力，还对身体

[1] 夏磊.运动干预对西安市小学生体质健康影响的实验研究[D].西安体育学院,2012,5.

形态和身体机能产生直接的影响。[1]观察运动干预对受试者身体素质的影响及作用的大小，是此次实验的主要内容之一。本研究受试者的年龄均处于身体素质的增长发育阶段，研究证实运动和健康教育联合干预对身体素质的提高，起到了极大促进作用。

目前国内外已有很多关于运动干预促进速度素质发展的研究，如钟春盛[2]研究证实，学生的兴趣和注意力的集中是运动干预提高速度的重要因素。 刘洪迅[3]从生理学的角度对速度素质进行分析，得出速度的快慢主要是由大脑皮层神经系统活动所决定的，肌肉的协调与放松是由于中枢神经系统兴奋与抑制的交替而产生的，中枢神经系统兴奋和抑制交替得越快，肌肉的收缩与放松也就交替的越快，同时表现的速度就越高。

本研究用 50 米跑的指标来对学生的速度素质进行评价，干预前通过对学生体育课的观察，在体育课上服装各式各样，学生跑的姿势五花八门等等。由于本研究所选取的样本来自山区小学，学校师资力量缺乏，上体育课没有专业体育教师，也没有人给他们讲上体育课的基本要求以及正确的跑的姿势。经过 12 周的运动和健康教育联合干预后，学生在着装有了很大的改观，跑的姿势也比以前好多了，50 米跑成绩也比干预前提高了。由表 5-12、图 5-16 得知干预后运动干预组、运动和健康教育联合干预组 50 米的成绩要好于干预前，也好于 2010 年全国学生体质健康调研数据该年龄段城市儿童的平均成绩（9.85s），而对照组干预前后没有多大变化。分析原因我认为主要是在系统的运动干预过程中教会他们在跑之前要进行热身活动，使身体各个器官机能处于兴奋状态，并教会了他们掌握正确的技术动作，如正确的摆臂姿势、如何增加步幅增快步频等，加之他们正处于速度素质的敏感时期（9-12 岁），这些对于速度素质的提高会起到很好的作用。

耐力素质是主要反映人体长时间运动的能力，也是用来评价一个人体质健康与否的重要指标。一般情况我们把耐力素质分为一般耐力素质和专项耐力素质。“全国学生体质健康调研”规定小学生采用 50 米×8 往返跑的测试方式，通过成绩的评定来反映学生的一般有氧耐力素质。

本研究也是通过 50 米×8 的指标来对山区学生的耐力素质进行评价的，通过对山区学校以及学生生活习惯的调查得知，山区学校由于所在地理位置的特殊性、经济条件的限制及校领导的不重视等原因，导致学校不能给学生提供很好的场地器材以及较大的师资力量来对发展学生的耐力素质，但也正是由于地理位置的特殊性决定了山区儿童的生活习惯与城市儿童的不同，城市儿童由于家长的溺爱，假期生活基本上都在家里看电视、玩电脑等，而山区儿童放假的时候喜欢结伴进行登山活动，所以干预前山区儿童的耐力素质要好于同年龄段的城市儿童。但是由于他们对长跑方面的知识了解很少，比如长跑在“极点”到来的时候，他们只是停下来歇歇，而不是想办法克服“极点”或推迟“极点”的到来。

从表 5-14、图 5-18 可以看出，经过 12 周的运动和健康教育联合干预后，运动干预组以及运动和健康教育联合组 50 米×8 的成绩不仅好于干预前，也好于对照组，主要由于对正处于生长发

[1] 陈志华.短跑运动员体能训练的内容及原则[J].商情,2010,26:15.
[2] 钟春盛.如何提高小学生的短跑成绩[J].田径,2009, 10:46-48.
[3] 刘洪迅,龙斌.速度素质在现代竞技运动中的作用、影响因素及对策[J].武汉体育学院学报,2002,5(36):61-63.

育的他们进行系统的运动干预。而干预后运动和健康教育联合干预组的成绩比运动干预组的成绩更好，分析原因我认为主要由于在健康教育的过程中给他们讲解了有关长跑方面的知识，让他们对耐力有关知识有了初步的了解，知道了长跑时出现了“极点”、“第二次呼吸”这些专业术语，也知道了在长跑过程中当“极点”到来的时候该怎么处理，这些对耐力素质的提高均有很好的作用，这一点也得到了有关研究的支持。如姚卫[1]从生理学的角度对小学生的有氧耐力进行研究，他的研究发现采用最大心率的 60%-80%的强度进行递增负荷训练，每个强度持续 2 周可以有效的提高学生的心肺功能和有氧耐力素质。夏磊[2]的研究证实，长期参加有规律的、系统的体育锻炼，能够提高有氧耐力，促进血液循环。

身体的柔韧性是由身体一个关节或多个关节的联合活动范围来表现的，是评价一个人身体素质的又一个重要指标。柔韧性的水平高低直接决定相应关节运动幅度大小。研究发现，四肢的柔韧性与身体运动能力的关系很小，而躯干和髋部的柔韧性与运动能力的关系密切。坐位体前屈是评价学生柔韧性水平最常用的指标之一，其功能主要是测试人体在静止状态下身体的躯干部位关节的灵活性以及躯干周围肌肉和韧带的弹性和伸展性。宋启灿等在儿童少年柔韧素质发展规律研究中证实，儿童、少年的韧柔素质，随年龄的增大而减退，在整个发展过程中出现两次韧柔发展的敏感期，男子 8-10 岁、13-14 岁，女子是 8-10 岁、13-15 岁。在他的研究中还发现，儿童少年柔韧素质较差主要与不参加体育锻炼有关。[3]郑晶[4]通过对武汉市某一小学的学龄儿童的研究中证实，柔韧的好坏与关节、肌肉的力量和伸展性有关。每次实施运动干预训练前，组织受试学生进行全身的拉伸练习，同时在运动干预中也安排了一些拉伸韧带和肌肉的技术动作，训练结束后组织学生进行拉伸部位的放松练习，这些对于提高学生的柔韧素质都有很好的效果。

本研究通过用坐位体前屈指标对山区儿童的柔韧素质进行评价。干预前对山区儿童上体育课的基本情况调查得知，山区学生在上体育课不管进行任何练习都没有进行热身活动的习惯，也不知道如何做准备活动，在体育课结束的时候也没有进行放松活动的习惯，这与他们缺乏专业体育老师的指导有关。本实验经过 12 周的运动和健康教育联合干预后，对照组在实验前后没有多大变化，运动干预组、运动和健康教育联合干预组的成绩要好于实验前，一方面是由于在运动干预的过程中增加很多活动关节拉伸、韧带和肌肉的技术动作并多次练习，另一方面在健康教育的过程中，给他们讲解做准备活动的重要性以及如何进行准备活动，教他们一些基本的活动关节和拉伸肌肉、韧带的动作。

力量素质评价人体身体素质的又一个重要指标，直接影响人体的运动能力，生活中的很多运动都离不开力量素质，如最基本的跑、跳、投以及平时的体力活动。力量素质的好坏还直接影响我们的日常工作和学习。本研究通过对握力、背力、跳远等指标来对山区学生的力量素质进行评价。

[1] 姚卫. 运用最大心率百分比控制小学生有氧耐力训练强度[J].上海体育科学研究,2003,24(5):76-77.
[2] 夏磊.运动干预对西安市小学生体质健康影响的实验研究[D].西安体育学院,2012,5.
[3] 宋启灿,董平,张万芝.儿童少年柔韧素质发展规律初探[J].西北师范学院学报,2005,4(6):74-78.
[4] 郑晶.运动干预对武汉市学龄儿童体质健康的影响研究[D].华中师范大学,2012,5.

握力主要是用来测试前臂肌肉的力量大小。目前有关握力的研究国内外以进行过多方面的研究，如常燕[1]在儿童少年体成分与力量素质的相关的研究中证实，男生握力的大小与瘦体重有关，可以通过减少多余的脂肪增加瘦体重，来增加握力的大小。女生握力的大小与瘦体重关系不大，则要有计划的进行上臂以及前臂小肌肉群的练习来增加握力。陈严等[2]的研究发现发现握力与肘关节屈肌力量相关性高，而与伸肌力量的相关性较低。尹松[3]在对重庆市南开中学的学生进行网球运动干预的研究证实，握力的提高与大量的身体练习有关，主要是肘部和腕部以及前臂的练习。在对受试者进行网球运动干预时，肘部和腕部的肌肉保持高度的紧张性和灵活性。受试者经过15周的干预后肘部和腕部的肌肉力量和关节的灵活性都会增加，从而提高受试者的握力。

本研究通过对受试者进行12周的运动和健康教育联合干预后，运动干预组、运动和健康教育联合干预组握力成绩的提高大于对照组且差异显著。一方面由于本研究对象所处的地方为山区，平时身体活动时间较城市儿童要多，他们在放假的时候也会帮家里干一点力所能及的农活，久而久之力量素质发展较好。另一方面在运动干预的时候加入一些与增加前臂肌肉力量的动作，比如引体向上、提重物、单杠悬垂等。这些对握力的提高都有很好的效果。

立定跳远是用来测试学生的下肢爆发力与弹跳力等身体素质的一项指标。Blake 等[4]首先对肢体的摆动幅度进行研究，设计了严格控制组与不摆动组，发现摆动组跳远远度超过不摆动组的21.2%，进一步拓宽了立定跳远技术研究的方向。代丹[5]建议练习立定跳远时，多做增加大腿股四头肌和小腿三头肌肌肉力量和爆发力的练习，从而提高立定跳远的成绩。 Seyfarth 等[6]从动力学机制方面对跳远进行了研究，指出在完成跳远动作时支撑腿的负荷和平衡的控制的研究是未来研究的重点。王宏达[7]从跳远时，大小腿夹角对成绩的影响进行研究的，在研究中发现起跳时站位的角度对跳远的成绩影响很大，起跳时站位角度 *90°*时的成绩最好。

本研究通过对受试对象进行12周的运动和健康教育联合干预后，运动干预组、运动和健康教育联合干预组跳远的成绩要好于对照组且差异显著。一方面是由于对受试者进行运动干预，干预的内容主要是增加大腿股四头肌和小腿三头肌下肢肌肉力量和爆发力有关的动作，爆发力的好坏与肌肉力量增加的程度有关。另一方面，对受试者进行运动健康教育干预，在干预的过程中给他们讲解跳远的动作及注意事项，给他们示范跳远的完整动作。由于本研究对象为山区学生，经济条件、地理位置的限制，加上山区没有真正的体育老师，学生也不清楚跳远的规范动作，他们的跳远都是根据自己平时的感觉来跳，这些都是影响跳远成绩提高的因素。

背力主要是用来测试受试者背部肌肉的力量，目前对背力的研究相对较少。常燕[8]在体成分

---

[1] 常燕,郑师超.儿童少年体成分与力量素质相关的研究[J].荆州师范专科学校,2004,3(39):273-274.
[2] 陈严,郝卫亚,温煦,庄洁.简易力量测试法和等速肌力测试相关性研究[J].体育科学，2006,26(9):93-97.
[3] 尹松.网球运动对中学生体质健康影响的研究[D].西南大学,2010,5.
[4] Bblake M.Ashby,Jean Heegaard.Role of arm motion in the standing long jump[J].Journal of Biomechanics ,2002,35:1631-1637.
[5] 代丹.对立定跳远相关肌肉训练的探讨[J].辽宁体育科技, 2003,5(6):7-8.
[6] A. Seyfarth,A. Friedrichs,V. Wank, R. Blickhan.Dynamics of the long jump[J]. Journal of Biomechanics,1999(32) :1259-1267.
[7] 王宏达.试论起跳时大小腿夹角对立定跳远成绩的影响[J].辽宁师专学报,2003, 1(6):96-99.
[8] 常燕,郑师超.儿童少年体成分与力量素质相关的研究[J].荆州师范专科学校,2004,3(39):273-274.

与力量素质相关的研究中证实，9-13 岁的儿童少年背力的增加是随着体重的增长而增加。本研究中受试者经过 12 周的运动和健康教育联合干预后，运动干预组、运动和健康教育联合干预组背力成绩提高要好于对照组且差异显著。背力的增加主要是肌肉力量的增加，他们正处于生长发育的关键时候，肌肉力量的增加会随着年龄体重的增加而增加。

7 结论与建议

7.1 结论

（1）12 周的运动和健康教育联合干预对山区儿童日常行为习惯、体育锻炼行为、心理健康方面可起到良好的促进作用，但由于受家庭经济条件、学校条件的限制，在饮食方面的作用不大。

（2）12 周的运动和健康教育联合干预对山区儿童身体形态的发育可起到良好的促进作用，具体表现为：增高、塑身促进儿童身体各部位协调发展。

（3）12 周的运动和健康教育联合干预有利于山区儿童心肺功能的发展，但对血压的影响不大。

（4）12 周的运动和健康教育联合干预有利于促进学生身体素质的协调发展。在发展学生的力量素质（背力、跳远）、柔韧素质、速度素质、耐力素质方面的作用尤为明显，但由于干预时间及学生自身特点、教学条件的局限，在发展学生力量素质的握力作用不明显。

7.2 建议

（1）各级政府部门教育部门要重视山区体育的发展，有关山区体育的经费投入要得到保证，场地器材、师资力量及教学条件要得到改善。

（2）体育课的课程设置严格按照国家体育课程标准执行，学校有关校领导和教师要认真落实国家规定的每天一小时的课外体育活动。

（3）上级部门要认真落实国家对贫困山区的“营养补贴”，加强营养知识的普及。

致谢

三年的研究生学习生活转瞬即逝，诸多不舍、诸多留恋、诸多伤感。但，这不是终点，只是一个新起点。三年，这片土地积淀了我的性格，让我稳重、踏实、勤劳；三年，这所校园养成了我的态度，让我积极、务实、求真。我将背着这载满知识和精神的行囊继续我的人生之路。回顾这大半年来的毕业论文完成过程，有过手足无措，有过停滞迷茫，但是在许多老师、同学和朋友的帮助下，我历经艰辛、克服困难终于战胜了自己，完成了论文。所以，在此我要向他们表达最诚挚的谢意。

首先，我要感谢我的导师乔玉成教授。乔老师不仅知识渊博、态度严谨而且为人谦和、平易近人。从论文开始的选题，到中间的具体操作，再到最后的写作阶段，乔老师倾注大量的心血为我引导、纠错、。在我陷入迷茫，停滞不前时，乔老师又给予我莫大的关心和鼓励，让我走出困境，坚持向前。在此，我谨向乔老师致以深深的谢意。

其次，我还要感谢陈乐琴老师，邹远强同学、刘博同学、许鹏同学正是他们的无私帮助，我才能汲取到更多的专业知识，顺利的完成论文。最后，还要特意感谢我的父母，他们一如既往的关心照顾，让我勇敢的步步向前进。

参考文献

[1] 夏磊.运动干预对西安市小学生体质健康影响的实验研究[D].西安体育学院硕士论文,2012,05.
[2] 王增峰.以运动干预为手段改善北京市大学生心理健康状况的可行性探析[D].北京体育大学硕士论文,2007,05.
[3] 中国学生体质与健康研究组.2000 年中国学生体质与健康调研报告[M].北京:高等教育出版社,2002.
[4] 于道中.体质健康概念与我国学生体质健康状况[J].山东体育学院学报,1994,10(2):7-14.
[5] 丁锡祉,郑远昌.再论山地学[J].山地研究,1996,14(2):83-88.
[6] 张占平.对高职高专学生体质健康现状及若干影响因素的研究[D].苏州大学硕士论文,2005,10.
[7] 季成叶.中国高才青少年的地域分布特点[J].体育科学,2000,20(1):89-92.
[8] 程颖.中学生矮身材发育状况及致矮因素研究[J].中国校医,1995,9(1):5-9.
[9] 胡卓生.2000 年甘肃城乡中小学生形态发育与体质健康状况[J].中国学校卫生,2004,25(6):730.
[10] 张钊,林彦.河北省不同经济地域国民体质机能指标研究[J].保定学院学报,2008,21(4):95-97.
[11] 姜文凯,江苏省国民和学生体质监测某些跨系统共有指标的特征及其影响因素[J].体育与科学,2002,23(3):40-45.
[12] 仇建生.中体育测量发展比较研究[J].北京体育大学学报,2003,26(6):778-780.
[13] 张建华.美国体适能教育计划对我国体育课程改革的启示[J]体育与科学,2001,22(l):68-70.
[14] 麦克吉.叶国雄译.体育实用测量学[D].湖南:湖南科学技术出版社,1993,07.
[15] 杨贵仁.学生体质健康泛教育论[D].福州大学博士师范论文,2005.
[16] 何仲恺.体质与健康关系的理论与实证研究[D].北京体育大学博士论文,2001.
[17] 史儒林.青海高原地区大学生生活方式和体质健康状况的调查研究[D].北京体育大学硕士学位论文,2005,07.
[18] 徐元玉.当代大学生健康状况的调查与分析[J].安徽体育科技,2006,27(2):68-71.
[19] 吕建中.常州市市区小学生形态生长发育状况研究[J].现代预防医学,2008,22:4411-4413.
[20] 赵德才.2004 年七个省汉族学生的运动素质发育状况[J].中华预防医学杂志,2005,6:385-387.
[21] 尹小俭.我国大学生肥胖流行现状及体质变化趋势[J].成都体育学院学报,2009,35(1):65-68.
[22] 陈铮,姚兴家,王金行.小学生身体成分与身体素质的研究[J].中国学校卫生,1994,15(1):5-6.
[23] 邢文华.14-15 岁男女生身体成份、机能和运动能力的研究[J].体育科学,1984,17(2):17-19.
[24] 郑晶.运动干预对武汉市学龄儿童体质健康的影响研究[D].华中师范大学,2012,5.
[25] 陈志华.短跑运动员体能训练的内容及原则[J].商情,2010,26:15.
[26] 钟春盛.如何提高小学生的短跑成绩[J].田径,2009,10:46-48.
[27] 刘洪迅,龙斌.速度素质在现代竞技运动中的作用、影响因素及对策[J].武汉体育学院学报,2002,5(36):61-63.

[28] 运用最大心率百分比控制小学生有氧耐力训练强度[J].上海体育科学研究,2003,24(5):76-77.

[29] 宋启灿,董平,张万芝.儿童少年柔韧素质发展规律初探[J].西北师范学院学报,2005,4(6):74-78.

[30] 郑晶.运动干预对武汉市学龄儿童体质健康的影响研究[D].华中师范大学,2012,05.

[31] 常燕.儿童少年体成分与力量素质相关研究[J].荆州师范专科学校,2004,3(39):273-274.

[32] 陈严,温煦,庄洁.简易力量测试法和等速肌力测试相关性研究[J].体育科学,2006,26(9):93-97.

[33] 尹松.网球运动对中学生体质健康影响的研究[D].西南大学,2010,05.

[34] Bblake M.Ashby,Jean Heegaard.Role of arm motion in the standing long jump[J].Journal of Biomechanics ,2002,35:1631-1637.

[35] 代丹.对立定跳远相关肌肉训练的探讨[J].辽宁体育科技,2003,5(6):7-8.

[36] A. Seyfarth,A. Friedrichs,V. Wank, R. Blickhan.Dynamics of the long jump[J]. Journal of Biomechanics,1999(32):1259-1267.

[37] 王宏达.试论起跳时大小腿夹角对立定跳远成绩的影响[J].辽宁师专学报,2003, 1(6):96-99.

[38] 季浏,科克比.身体锻炼心理学的研究现状和未来方向[J].天津体育学院学报,1997,12(3):8-11.

[39] Weinberg,Jackson,Kolodny.the lation ship of massage and exercise to mood enhancement[J].The Sport Psychologist,1988,2:202-221.

[40] 吴德亮.阳光体育对农村小学生体质健康影响的实验研究[D].海南师范大学,2011,05.

附录

山西省运城市某山区儿童饮食和体育锻炼情况的调查问卷

亲爱的小朋友们：

你们好！根据你对自己的看法，判断下面每个句子所描述的内容是否符合自己的实际情况。所有题目没有对错，请你快速回答，看谁做的最快。你们都是最棒的！

山西师范大学 2010 级研究生：解达立

导　师：乔玉成

填表说明：

（1）按要求请在符合您情况的“()”内划“√”

（2）凡遇到“_____”时，请把答案写到上面。

调查对象的基本情况

1. 性别：　（1）男　（2）女
2. 年龄：　_____ 周岁
3. 年级：　_____
4. 是否是独生子女：（1）是　（2）否
5. 你的健康状况：　(1)良好　（2）一般　(3)差

关于饮食的基本情况

6. 一日吃几餐？

（1）一餐　（2）两餐　（3）三餐　(4)三餐以上

7. 是否有吃早餐的习惯：

（1）是　（2）否

8. 早餐以什么为主？（可多选）

（1）鸡蛋　（2）豆浆　（3）牛奶　（4）粥或稀饭　(5)馒头　（6）水果　（7）其他

9. 有吃零食的习惯吗？

（1）有　（2）没有

10. 把哪些食物作为零食？（可多选）

（1）糖果类（巧克力 、糖块）（2）麻辣片类　（3）甜点类（饼干、蛋糕）　（5）干果（瓜子、花生）　（6）其他

11. 午饭一般以什么为主？

（1）大米　（2）　面食　（3）馒头　（4）其他

12. 晚饭一般以什么为主？

（1）大米　（2）面食　（3）馒头　（4）稀饭　（5）其他

心理健康方面的基本情况

13. 见了认识的长辈（　　）

（1）主动热情招呼 （2）不好意思 （3）躲起来

14. 和同学在一起玩，你感到（　　）

（1）快乐 （2） 一般（3）不合群 （4）孤独

15. 与新朋友在一起（　　）

（1）主动邀请一起玩（2）不好意思接近 （3）只顾自己玩

16. 课堂上老师提问（　　）

（1）马上回答（2）想一会再回答（3）等别人答（4）老师提问才答，一般不回答

17. 我上课不敢发言，即使我知道答案.

（1）偶尔 （2）没有 （3）经常 说不清楚

18. 老师布置的作业（　　）

（1）认真完成（2）完成一些（3）不完成 （4）边做边玩

19. 期末考试时（　　）

（1）心情紧张（2）有点紧张（3）无所谓（4）有信心，不紧张

20. 假期一般做什么？

（1）看电视 （2）找同学玩 （3） 一个人呆在家里 （4） 帮家长干活

体育锻炼的基本情况

21. 喜欢上体育课吗？

（1）喜欢 （2） 不喜欢

22. 是否经常参加课外体育活动？

（1）是 （2）否

23. 每周参加体育活动的次数（体育课除外）：

（1）一次 （2）两次 （3）两次以上

24. 每次参加体育活动的时间大约是：

（1）30分钟以下 （2）30-60分钟 (3)60-90分钟

25. 是否知道科学的体育锻炼？

（1） 是 （2） 否

26. 你觉得体育锻炼对你的学习生活有好处吗？

（1）有 （2） 没有 （3）不清楚

万方数据 WANFANG DATA 文献链接

运动和健康教育联合干预对山区儿童体质健康的影响

作者： 解达立
学位授予单位： 山西师范大学

引用本文格式：解达立 运动和健康教育联合干预对山区儿童体质健康的影响[学位论文]硕士 2013
