# Supplementary material for: Systematic review and meta-analysis of school-based obesity interventions in mainland China
Source: PLoS One. 2017 Sep 14;12(9):e0184704. doi: 10.1371/journal.pone.0184704 (PMC5598996; doi:10.1371/journal.pone.0184704)
Supplement: S1 Dataset — (ZIP) [file pone.0184704.s007.zip › S1_dataset/76库/88.pdf]

上海师范大学

硕士学位论文

三个月耐力锻炼对小学四年级学生有氧能力为主的体质指标的影响

姓名：金海娜

申请学位级别：硕士

专业：体育教育训练学

指导教师：卢昌亚

20090301

三个月耐力锻炼对小学四年级学生有氧能力为主的体质指标的影响

（金海娜 上海师范大学 200234） 指导教师：卢昌亚 教授

摘要：

最大吸氧量是人体最大有氧运动能力的主要指标之一。最大吸氧量的测定对评价小学生的生长发育水平、心肺功能潜力和运动素质具有重要的意义。本文通过对实验组小学生进行为期三个月的有氧耐力练习并将结果与对照组、锻炼前实验组进行比较分析，试探讨三个月的时间对有氧工作能力以及身体形态和运动素质方面的影响。

研究对象为温州市瑞安安阳实验小学四年级学生,平均年龄 10.3 岁，共 122 人，男生 69 人，女生 53 人，在规定实验组人数的前提下随机分为实验组和对照组。其中对照组 93 人，（男 54 人，女 39 人）；实验组 29 人，（男 15 人，女 14 人）。锻炼后实验组有效人数为男生 15 人，女生 14 人。事先了解学生的身体状况，排除有身体外伤以及有慢性疾病的学生。

本论文研究方法包括文献资料法、实验测量法、数理统计法和比较分析法。所有直接测量项目参照《学生体质健康标准》所要求的进行测量，了解小学生的体质健康状况。间接推算最大吸氧量绝对值：采用卡尔普曼公式,先求出 PWC170 台阶实验的功率，然后由功率推算最大吸氧量值。探讨最大吸氧量与肺活量、胸围的关系，并建立回归方程。

实验结果表明：(1) 测试学生与全国学生的各项指标的差异性分析得出，除了身高，各项都存在差异，部分存在非常显著性差异。(2) 卡尔普曼公式推算儿童的最大吸氧量是可行的，测试对象的最大吸氧量水平中等偏下，以最大吸氧来量相对值计算比绝对值的成绩好。(3) 对照组在三个月时间里男女的身高体重都有一定的增长，男生的身高体重存在显著性差异，其他各项指标不存在统计学差异。(4) 实验组与对照组锻炼前后，男女身高体重都不存在统计学差异，但是实验组的增长比例比对照组大，男女在立定跳远、肺活量、PWC170 上存在显著性差异，男生在最大吸氧量绝对值上存在显著性差异，女生在最大吸氧量相对值上存在显著性差异。说明，锻炼达到了提高人体有氧工作能力的效果。(5)，实验

组锻炼前后，男女生在 PWC170 和最大吸氧量绝对值上都存在显著性差异，在身体形态和运动素质指标方面，除了女生的体重存在显著性差异，其他各项均没有统计学差异。但是在各项的增长比例上均大于锻炼后的对照组的数值，说明在一定程度上，锻炼还是对身体形态和运动素质起到积极的作用。（6）最大吸氧量绝对值与肺活量的回归方程与相关性系书 R，男生 Y=0.7585x+628.44，R=0.677，高度正相关，女生，Y=0.4017x+1151.2，R=0.583，中度正相关；最大吸氧量相对值与胸围之间的回归方程和相关系数 R，男生，Y=0.4676X+37.547，R=0.67 高度正相关，女生，Y=-0.5309X+91.708 ，R=0.554，中度负相关，女生方面出现的负相关可能与女生的身体发育有关。(7)三个月可以作为一种阶段性的措施，在一定程度上提高了小学生的有氧工作能力，对于身体形态和运动素质也有一定的正面影响。

关键词：小学生，耐力锻炼，有氧工作能力，最大吸氧量，身体形态，运动素质

Abstract:

The maximum oxygen uptake are human maximal aerobic exercise capacity, one of the main indicators. Determination of maximum oxygen uptake for the evaluation of growth and development of primary level, the potential of cardiopulmonary function and quality of exercise is of great significance. Based on the experimental group pupils for a three-month aerobic endurance exercise and the results with the control group, the training before the experimental group carried out a comparative analysis of three-month trial period to explore the ability to work on the aerobic and physical shape and quality of sports impact.

Research subjects for Anyang Ruian Wenzhou City Experimental Primary School fourth grade students, average age 10.3 years old, a total of 122 people, 69 boys and girls 53 people, at the number provided for the experimental group, under the premise were randomly divided into experimental group and control group. One of the control group of 93 people, (male 54, female 39 people); the experimental group of 29 people, (male 15, female 14 people). Effective after training the experimental group the number of boys and 15 girls 14 people. Know in advance the physical condition of students, has ruled out physical injury as well as students who have chronic diseases.

This dissertation research methods including literature, experimental and mathematical statistics and comparative study of law. Direct measurement of all items with reference to "student's health standards" required to carry out measurement, pupils know the physical health status. Indirectly, the largest projected absolute oxygen uptake: The Karepuman formula, first obtained experimental stage PWC170 power, then by the maximum oxygen uptake values of power projection. Explore the maximum oxygen uptake and lung capacity, the relationship between the chest and set up the regression equation.

The experimental results show that: (1) to test students with the various indicators of national student diversity analysis, in addition to height, the differences are partly exist very significant difference. (2) formula Karepuman oxygen uptake child's best projections are feasible, the maximum oxygen uptake test the level of lower-middle, with the greatest amount of oxygen to the relative value than the absolute value of a

good grade. (3) the control group at three-month period for men and women must have the height and weight growth, height and weight of boys, there was a significant difference, other indicators do not exist significant difference. (4) the experimental group and control group before and after training, male and female height and weight do not exist significant difference, but the growth of the proportion of the experimental group than the control group, and men and women in standing long jump, vital capacity, PWC170 on significant differences exist, the boys in the maximum inspiratory oxygen exist on the absolute value of significant difference between the girls at the largest on the relative value of oxygen uptake, there was a significant difference. Shows that training has reached the body improve the effectiveness of aerobic work capacity. (5), the experimental group before and after training, for boys and girls at PWC170 and maximum oxygen uptake in absolute terms on all exist significant differences in body shape and exercise quality indicators, apart from the weight of females, there was a significant difference, the other did not statistical difference. However, the growth in the proportion of training than the control group values in a certain extent, training or exercise on the body shape and play a positive role in the quality. (6) the largest absolute value of oxygen uptake and lung capacity of the regression equation with the relevant book of the Department of R, male Y = 0.7585x +628.44, R = 0.677, a high positive correlation, girls, Y = 0.4017x +1151.2, R = 0.583, moderate positive correlation; maximum oxygen uptake Maximum oxygen uptake and the relative value of the regression equation between the chest and the correlation coefficient R, for boys, Y = 0.4676X +37.547, R = 0.67 a high positive correlation, girls, Y =- 0.5309X +91.708, R = 0.554, moderate negative correlation girls there a negative correlation may be related to the physical development of girls concerned. (7) for three months can be used as a stage measures, to a certain extent improve the primary job of aerobic capacity, body shape and for the quality of sport must also have positive effects.

Key words: Primary, endurance training, aerobic work capacity, maximum oxygen uptake, body shape, the quality of sports

论文独创性声明

本论文是我个人在导师指导下进行的研究工作及取得的研究成果。论文中除了特别加以标注和致谢的地方外，不包含其他人或机构已经发表或撰写过的研究成果。其他同志对本研究的启发和所做的贡献均已在论文中做了明确的声明并表示了谢意。

作者签名：[illegible] 日期：2009.6

论文使用授权声明

本人完全了解上海师范大学有关保留、使用学位论文的规定，即：学校有权保留送交论文的复印件，允许论文被查阅和借阅；学校可以公布论文的全部或部分内容，可以采用影印、缩印或其它手段保存论文。保密的论文在解密后遵守此规定。

作者签名：[illegible] 导师签名：[illegible] 日期：2009.6

第一部分　文献综述

1. 有氧工作能力

1. 1 有氧工作能力的概念

有氧工作能力（Aerobic working capacity），是指反映本人有氧供能的能力。这种能力包括最大摄氧量、维持最大和次最大摄氧量的能力。

1. 2 最大吸氧量

在进行有大量肌肉参加的长时间激烈运动中，心肺功能和肌肉利用氧的能力达到本人极限水平时，单位时间内所能摄取的氧量称为最大吸氧量（Maximal oxygen consumption）。人体进行有氧工作时，最大吸氧量反映了机体呼吸和循环系统氧的运输工作能力。

1. 3 最大吸氧量概念产生的历史背景

早在 1922 年，英国生理学家 Archibald Vivian Hill（希尔）以对两栖类动物离体肌肉能量代谢的出色研究，荣获了当年的医学和生理学诺贝尔奖。第二年，Hill 等人又进行了一系列的人体实验，发表了关于肌肉运动、乳酸及氧的供应和利用的论文，并首次提出机体利用氧的能力存在上限的观点。他认为，摄氧量在运动中会达到一个最大值，由于循环和呼吸系统的限制，摄氧量不会无限制地增加。Hill 和其同事在 1924 年的一部专著中详细地讨论了最大吸氧量的问题，确立了最大吸氧量的理论。Hill 提出，最大吸氧量受到心血管和呼吸系统的限制“超过此限度后，无论跑速如何增加，摄氧量都不会再增大，心脏、肺，以及氧到达运动肌纤维的扩散都已达到极限。此时机体对氧的需求不能得到满足，乳酸堆积，氧债连续增加，疲劳和力竭开始产生”。80 多年来 Hill 的最大吸氧量理论被大量的实验所证实，一直被广泛应用，大多数有关运动员运动能力的研究文献中都有最大吸氧量的指标。美国当代运动生理学 Fox 认为最大吸氧量即“机体在运动时单位时间内能摄取并利用的氧的最大值”，其大小以及它对运动的适应性对运动有重要意义，它反映了机体运输氧以及利用氧的能力，也就是呼吸系统、心血管系统和线粒体的功能和能力，是决定运动员的运动能力和成绩的重要因素。因此体育界的共识认为最大吸氧量理论是运动科学的基础之一。

1. 4 最大吸氧量的表示方法

最大吸氧量的表示方法有两种，即绝对值和相对值。绝对值用 L/min 表示，

即整个机体在单位时间内（每分钟）所能吸收的氧量。由于需氧量与体重成正比关系，而身高、体重存在个体差异，因此，用绝对值进行个体间的横向比较是不合适的，而常用人体最大吸氧量的相对值（ml/kg/min）来进行不同个体间最大吸氧量的比较。

1．5 影响最大吸氧量的因素

1．5．1 心脏的泵血功能　这是最大吸氧量的中央机制

吸氧量 = 心率×每搏输出量×动静脉氧差

1．5．2 肌肉利用氧的能力 这是最大吸氧量的外周机制，肌纤维类型影响肌肉的摄氧能力，慢肌纤维的形态和代谢特点有利于增强肌肉对氧的摄取。

1．5．3 遗传因素 Klissuras 等人 1972 年研究了 25 对双生子，发现最大摄氧量的遗传度为 93．4%。

1．5．4 年龄和性别因素 青春期前男女最大吸氧量的差异很小，12~13 岁之后差异逐渐增大。15~17 岁为女子峰值；18~20 岁为男子峰值。此后随年龄增加，男子一每年 2%，女子以每年 2。5%幅度下降，60 岁时降到峰值的 70%。成人男子最大吸氧量的相对值高于女子的 15%~20%。男女最大吸氧量的差异是由于两者的血液、心脏心输出量和内分泌等身体特点引起的。

1．6 有氧工作能力的测定

有氧工作能力的测定包括最大吸氧量的测定和次最大运动负荷的测定。最大吸氧量的测定有直接测定法和间接测定法。次最大运动负荷的测定包括哈佛台阶试验、PWC_{170} 和无氧阈等的测定。

1．6．1　最大吸氧量的直接测定法

一般采用活动跑台上的跑或走（Bruce 方案或 Taylor 方案），以及脚踏功率自行车（McArdle 法或 Astrand 法）来测定。采用 4~9 分钟的渐增性力竭运动的负荷量。如进行活动跑台试验，则常采用递增一定的坡度和速度的方法增加负荷量。如进行功率自行车，则常用改变每分钟所蹬的圈数和功率或阻力来控制运动负荷。在实验中收集活动时的呼出气体，经过气体分析仪计算出单位时间内氧的最大摄入量。

1．6．2　最大吸氧量的间接测定法

1．6．2．1 Astrand-Ryhing 列线法　依据受试者的体重，采用运动负荷递增的台阶实验的心率数据，对照 Astrand-Ryhing 计算图推测受试者的最大吸氧量。

1．6．2．2　台阶指数推测法　根据受试者的性别年龄和身高，进行相适应的登台阶实验，运动时间可为3~5min，测量出完成运动后恢复期第1、2、3min后30秒的脉搏数A、B、C，并代入公式：K＝（T ×100）/ 2（A＋B＋C)；其中T 的计算单位为秒。然后，再将台阶指数K 代入最大吸氧量的推算公式（即回归方程）。

每分钟摄氧量推算公式：Y（ml/min）＝13．25K＋1740．86（适用于男子）

Y（ml/min）＝13．94K＋514．37（适用于女子）

每分钟千克体重的摄氧量推算公式：Y（ml/kg/min）＝0．2588K＋24．170（适用于男子）

Y（ml/kg/min）＝0．1912K＋17．264（适用于女子）

1．6．2．3　Cooper12分钟跑测定法　由美国运动生理学家Cooper在1968年创立。受试者以匀速尽力跑完12min的距离，以实际所完成的距离的米数，对照相应的标准，查出所对应的最大吸氧量的相对值。

1．6．2．4　PWC_{170}　PWC_{170}（**Physical Work Capacity**）是一种次最大运动负荷测定法，指心率在每分钟170次时的身体工作能力。PWC_{170}测试可以采用功率自行车或活动跑台进行负荷运动。其原理是心率阈功率在一定的范围内（相当于心率在120~180次/min之间）呈线性关系。根据两次运动的不同负荷和运动心率，可以推算出PWC_{170}的值，再由PWC_{170}的值推算出最大吸氧量，从而测定与评价受试者的有氧工作能力。推算公式如下。

Y（ml/min）＝2.2PWC_{170}＋1070（适用于运动员）

Y（ml/min）＝1.7PWC_{170}＋1240（适用于一般人）

Y（L/min）＝0.00129PWC_{170}＋2.040（适用于男少年运动员）

Y（L/min）＝0.00112PWC_{170}＋2.094（适用于女少年运动员）

1．7　推测最大吸氧量的其他方法

Y（L/min）＝2.5970＋0.0120H（cm）- 0.0273HR（次/min）＋1.3217

2 人体的最大吸氧量（VO_{2max}）的场地测试

2．1　最大吸氧量（VO_{2max}）的场地测试的意义

人体的最大吸氧量（VO_{2max}）是有氧运动能力的有效指标，它体现了人体氧的运输系统与肌肉摄取、携载和利用氧的能力，被运动生理学界和体育界公认为是对人体的机能、运动员的锻炼水平进行动态评估的重要指标之一。VO_{2max}的测

试也常常被用于学校体育教学，作为对学生进行体质评估的依据，以及在体育教学研究中作为不同教学和锻炼方法之间进行效果比较的一项可靠的量化指标。

然而，VO_{2max}的直接测定法需要较复杂的实验条件，如活动跑台和自行车功量计等，还要对吸入和呼出的气体进行成分分析，尤其是要求受试者进行负荷渐增的力竭性运动，而一般大学生的体能和意志素质并不足以完成力竭性运动，对于有身体疾患的学生更具有一定的危险性。这些条件限制了 VO_{2max} 的直接测定法在一般学校体育教学中的应用。于是运动生理学界研究出了多种不需要复杂设备的亚极量运动负荷场地测试法，用于学校体育，特别是美国近年来对这些方法的应用较为普遍和深入。现对其中可用于中小学生的，信度和效度较高的 5 种场地 VO_{2max} 估测法的利弊、应用方法和相关系数等进行介绍。

2．2　5 种场地 VO_{2max} 估测法

2．2．1　**12 分钟跑距离估测 VO_{2max}**

2．2．1．1 测试公式　VO_{2max}（$ml \cdot kg^{-1} \cdot min^{-1}$）= [距离（Km）—0.5049] ÷0.0447

2．2．1．2 测试方法

在 400 m 标准跑道上，每 10 m 做一标记，以便较精确地计算出跑的距离。要求受试者在进行必要的准备活动后，以自己最大的努力和基本均匀的速度跑完 12 分钟，以秒表计时，由专人统一口哨声示意 12 分钟时间到，受试者立即根据自己当时所跑的圈数和跑道上的标记各自精确计算出 12 分钟内所跑的距离，并将距离以千米为单位带入上述公式，所计算出来的数值就是受试者的最大吸氧量估测值，单位是 $ml \cdot kg^{-1} \cdot min^{-1}$。

2．2．1．3　效度分析

这一方法对不同的受试对象来说，其相关系数差异是很大的。对体育专业的大学生效度较高，而对中小学生，特别是女生，一般体质较弱，缺乏良好的运动习惯和一定的运动技巧，运动意志不强，大多不能长时间维持较高的匀速，导致在 12 分钟内跑的总距离过短。此法的优点是对于体能较好或锻炼水平较高的受试者，能比较准确地估测其 VO_{2max}，缺点是不宜用于中小学生和体能较差或锻炼水平很低的大学生。

2. 2. 2　用 2400 m（标准跑道 6 圈，约 1.5 英里）跑的时间估测 VO_{2max}

2. 2. 2. 1　测试公式

$$VO_{2max}(ml \cdot kg^{-1} \cdot min^{-1}) = 88.02 + 3.716(G) - 0.1656(WT) - 2.767(T)$$

其中 G 为性别系数，男性为 1，女性为 0；WT= 体重（kg）；T= 时间（min）。

2. 2. 2. 2　测试方法

受试者在测试前先称自身体重（WT），以千克单位计。要求受试者在进行必要的准备活动后，以自己最大速度以下强度、且基本均匀的速度跑完 1.5 英里，约为 2400 m，折合 400m 标准跑道约 6 圈，计跑完这一距离的时间（T），以分钟为单位。将 G、WT 和 T 的值代入上述公式，即得出受试者 VO_{2max} 的估测值。需要注意的是受试者跑步时的运动心率不应低于 110 次/分，可以用跑完后即刻的运动心率为参考指标。

2. 2. 2. 3　效度分析

这种测试方法的相关系数是最高的（0.90）。这是一种在欧美国家的大学中较为流行的 VO_{2max} 估测方法，但是它要求受试者在完成测试的过程中付出很大的体力。对于那些未经锻炼和缺乏跑步经验的受试者来说，完成这样的测试是很困难的，因为他们往往不可能跑完 2400 m。然而，对于胜任这种测试法的人来说，用此法所估测出的 VO_{2max} 值可能比直接测定法所测出来的值要略低，但相对来说是最接近的。因此这种方法的优点是准确度较高，缺点是应用范围较窄，中小学生和大多数锻炼水平不高的大学生无法完成。

2. 2. 3　用 1600 m（标准跑道 4 圈）慢跑的时间和心率估测 VO_{2max}

2. 2. 3. 1　测试公式

$$VO_{2max}(ml \cdot kg^{-1} \cdot min^{-1}) = 100.5 + 8.344(G) - 0.1636(WT) - 1.438(T) - 0.1928(HR)$$

其中，G 为性别系数，男性为 1，女性为 0；WT= 体重（kg）；T= 时间（min），HR 为心率（次/分）。

2. 2. 3. 2　测试方法

受试者在测试前先称自身体重（WT），以千克单位计。要求受试者在进行必要的准备活动后，以自己适宜的基本稳定的、均匀的慢速跑完 1600 m（4 圈），计跑完这一距离的时间（T），以分钟为单位，并测定跑完 4 圈后即刻的运动心

率（HR），以次/分计。将 G、WT、T 和 HR 的值代入上述公式，即得出受试者 VO_{2max} 的估测值。在这一测试方法中，如受试者途中不能坚持慢跑，可以走一段距离后再跑，记录的时间为慢跑和走的总时间。

2．2．3．3 效度分析

这种测试方法的相关系数是较高的（0.89）。这是一种在欧美国家的大学和高中生中较为流行的 VO_{2max} 估测方法。这种测试方法的可行性与安全性比上一种方法要高，因为在这种方法中只要求受试者慢跑，而无须以很大的强度进行运动，所以受试者大部分能够按规定正常完成测试过程。在这一测试方法中虽然没有规定慢跑的速度，但跑速的变化对估测值的相对准确性的影响并不大。因为如果跑速慢，虽然时间 T 这一项增大了，但由于跑速慢，运动心率 HR 这一项的值则相应减小了，于是公式的总代数和还是没有什么变化。当然，只有在慢跑的速度适中时，公式得出的估测值才是最佳的。然而这一测试方法，由于其 1600 m 总距离的跑程，对于部分体质较差和锻炼水平很低的大中学生来说，仍然属于较大的运动量，这在一定程度上限制了这一方法更普遍的应用。

2．2．4 用 1600 m（标准跑道 4 圈）快步走的时间和心率估测 VO_{2max}

2．2．4．1 测试公式

VO_{2max}（$ml\cdot kg^{-1}\cdot min^{-1}$）=123.853＋6.315（G）−0.3877（A）−0.0768（WT）−3.2649（T）−0.1516（HR）

其中，G 为性别系数，男性为 1，女性为 0；A 为年龄，以实龄（岁）为单位；WT= 体重（kg）；T= 时间（min），HR 为心率（次/分）。

2．2．4．2 测试方法

受试者在测试前先称自身体重（WT），以千克单位计。要求受试者在进行必要的准备活动后，以自己适宜的基本稳定的速度快步走完 1600 m（标准跑道 4 圈），记录走完这一距离的时间（T），以分钟为单位，并测定走完 4 圈后即刻的运动心率（HR），以次/分计。将 G、A、WT、T 和 HR 的值代入上述公式，即得出受试者 VO_{2max} 的估测值。

2．2．4．3 效度分析

这种测试方法的相关系数也是较高的（男为 0.79，女为 0.71）。这是一种在欧美国家刚刚兴起的，对大学和高中生中体能较差或锻炼水平较低的受试者进行

的 VO_{2max} 估测方法。因此，这种测试方法的可行性与安全性比上述几种方法要高，因为在这种方法中只要求受试者快步走，连慢跑都不需要。于是几乎所有的受试者都能够按规定正常完成测试过程。在这一测试方法中虽然没有规定快步走的速度，但时间 T 和运动心率 HR 这两项的值保证了估测值的稳定性。而且这一测试法中引入了年龄参数，体现了这一测试法在应用范围上的年龄特异性。

2．2．5 用 400 m（标准跑道 1 圈）快步走的时间和心率估测 VO_{2max}

2．2．5．1　测试公式

VO_{2max}（$ml \cdot kg^{-1} \cdot min^{-1}$）=88.768 +8.892（G）-0.0434（WT）-1. 4537（T）-0.1149（HR）

其中，G 为性别系数，男性为 1，女性为 0; WT= 体重（kg）; T= 时间（min），HR 为心率（次/分）。

2．2．5．2　测试方法

受试者在测试前先称自身体重（WT），以千克单位计。要求受试者在进行必要的准备活动后，以自己适宜的基本稳定的速度快步走完 400 m（标准跑道 1 圈），记录走完这一距离的时间，将这一时间×4，得出的积为（T），以分钟为单位，并测定走完 1 圈后即刻的运动心率（HR），以次/分计。将 G、WT、T 和 HR 的值代入上述公式，即得出受试者 VO_{2max} 的估测值。

2．2．5．3　效度分析

种测试方法的相关系数也是较高的（男为 0.75，女为 0.65）。这是一种在欧美国家刚刚兴起的，改良式的 VO_{2max} 估测方法。虽然相对来说，相关系数比上述几种方法的相关系数低，但由于测试时间相对节省了（只要求走 1 圈），因此在学校体育教学和锻炼中非常实用。然而，由于这种方法的测试公式中没有引入年龄参数，所以该公式只适用于大学生或中学生，甚至是小学生。这种方法中没有强调走的速度，同样是因为时间 T 和运动心率 HR 这两项的值保证了估测值的稳定性。当然，在理想情况下，即受试者保持其最大均匀步速行走时，估算的值相对来说为最为准确。

3．儿童少年的有氧运动能力

3．1 儿童少年氧的运输系统的形态与机能特点

3．1．1 儿童少年的血液特点

儿童少年的血液总量少于成人，但每单位体重的血液量则比成人的多。儿童少年血液及其成分的数量特点见表 1。

表 1 不同时期儿童少年与成人血液指标的数量

指标	新生儿	7 岁	12 岁	15 岁	成人
血液总量（占体重%）	15	12	10	8	8
红细胞（万个/mm^3）	700	450	460	500	500
白细胞（个/mm^3）	16000~20000	8000	7000	5000	5000
血红蛋白（g %）	20.2	11.0	12.5	13.5	14.0

（引自邓树勋等，运动生理学，2005）

3．1．2 儿童少年心血管系统特点

心脏重量与容积　儿童少年心脏的重量和容积均小于成人，但与体重的比值则和成人相近。儿童少年的心脏发育及神经调节尚不够完善，交感神经兴奋占优势，故心率较快。儿童少年心肌纤维交织稀疏，弹性纤维较少，心脏收缩力较弱，心脏泵血能力较差，因而每搏和每分输出量均小于成人，但相对而言单位体重的心输出量较大，有关数据见表 2。由于儿童少年心脏收缩力较弱，动脉血管和毛细血管的口径比成人的宽，外周阻力较小，故儿童少年血压较低。青春发育期，由于内分泌因素的影响，血压上升，部分儿童少年可能出现“青春期高血压”的现象，主要表现为收缩压的升高，且多见于发育良好成长迅速的青少年。指导这些孩子进行体育锻炼时，不宜运动量过大。

表 2　不同时期儿童少年与成人血液指标的数量

年龄（岁）	心脏重量		每搏输出量		每分输出量	
	绝对值（g）	占体重%	绝对值（ml）	相对值（ml/kg）	绝对值（ml）	相对值（ml/kg）
8	96.0	0.44	25.0	0.98	2240	88
13	172.0	0.50	35.7	0.95	2850	76
15	200.0	0.48	41.5	0.95	3150	70
18	305.3	0.51	60.0	0.88	4300	63
成人	310.0	0.25	75.0	1.07	5000	71

（引自邓树勋等，运动生理学，2005）

3．1．3　儿童少年呼吸系统特点

儿童少年的呼吸肌力量较弱，胸廓狭小，气道不够宽阔，而呼吸时的弹性阻力和气道阻力较大，所以呼吸的深度不及成人，表现为呼吸频率较快，而肺活量

较小。在进行剧烈运动时，儿童少年的最大通气量和最大吸氧量的绝对值比成人低，但其相对值却不低于成人，甚至还略高于成人水平，其具体数据见表3。

表3 儿童少年的最大肺通气量与最大吸氧量

年龄（岁）	最大肺通气量		最大吸氧量	
	L/min	L/kg/min	L/min	L/kg/min
**4~6**	30~37	1.95	0.8 ~ 0.9	45~47
**7~9**	43~51	2.04	1.0 ~ 1.2	47~50
**10~11**	52~65	1.94	1.3 ~ 1.4	47~54
**12~13**	56~60	1.92	1.7 ~ 2.0	44~53
**14~15**	62~67	1.90	1.8 ~ 2.3	35 ~51
**16~18**	70~100	1.80	2.0 ~ 3.0	37~53
成人	70~120	1.90	2.0 ~ 3.0	36~50

（引自邓树勋等，运动生理学，2005）

3. 2 儿童少年的能量代谢

儿童少年新陈代谢旺盛，除了要维持身体各器官的正常生理活动外，还必须有利于成长发育的需要。因此儿童少年在进行体育运动时，能量的动用受到一定的限制。儿童少年肝糖原的储量比成人少，肌肉占体重的百分比和肌糖原也比成人少，而最大吸氧量水平又较低，因此糖的有氧氧化能力不及成人，在长时间肌肉工作中易发生血糖水平低下，耐久力差。同时儿童少年糖的酵解能力小于成人，血液中乳酸含量小于成人，血乳酸水平较低，所以其长时间大强度的肌肉工作能力也比成人差。

3. 3 国内外儿童少年有氧运动能力比较

3. 3. 1 国外儿童少年有氧运动能力及其测定

3. 3. 1. 1 主要测试方法与测试数据

国外早期也曾多用一些人体测量数据和12分钟跑等方法来间接估测儿童少年的最大吸氧量等指标。Cumming（1978）曾比较了用人体测量数据和12分钟跑的距离预测儿童少年最大吸氧量的相关系数与误差，详见表4和表5。

表4　由人体测量学数据和12分钟跑的距离预测最大吸氧量的比较

年龄（岁）	实际最大吸氧量（ml/kg/min）	人体测量学预测		12分钟跑预测		人体测量学 +12分钟跑预测	
		r	SE	r	SE	r	SE
男（10~17）	49.5 ± 6.0	0.77	4.9	0.53	5.3	0.80	4.3
女（10~17）	35.0 ± 7.1	0.73	5.4	0.74	4.9	0.90	3.5
径赛少年运动员							
男（13~16）	60.6 ± 6.1	0.67	8.5	0.75	4.2	0.98	4.0
女（13~16）	44.1 ± 6.9	0.47	13.3	0.87	4.8	0.90	4.0

（依 Cumming，1978）

表 5 Cooper12 分钟跑的距离与最大吸氧量之间的关系

跑的距离（km）	由公式推算成人最大吸氧量（ml/kg/min）	由公式推算儿童少年的最大吸氧量(ml/kg/min) 男孩	女孩
**<1.6**	<28.0	47.0	38.8
**1.6~2.0**	28.1~ 34.0	48.3	47.0
**2.0~2.4**	34.1~ 42.0	55.9	55.2
**2.4~2.8**	42.1~52.0	63.5	63.4
**>2.8**	>52.0	71.1	71.6

Cooper 的另一项研究提出也可用 12 分钟的跑速估算最大吸氧量，公式如下。最大吸氧量（ml/kg/min）= 7.7 + 2.9V，此公式适用于成人，其中 V 为 12 分钟跑的平均速度，单位为 km/h。如 12 分钟跑了 2.4km，换算为 12km/h，则 7.7 + 2.9 × 12 = 42.5（ml/kg/ml）。

符合 9~12 岁儿童的公式为：最大吸氧量（ml/kg/min）= 14.1 + 3.8V （适用于男孩）；

最大吸氧量（ml/kg/min）= 10.1 + 4.1V（适用于女孩）。10 岁时 12 分钟跑的标准得分男孩为 2km，女孩为 1.8km。

国外还曾采用活动跑台的 Bruce 测验法来推测儿童少年的最大吸氧量。具体方法是：分三个阶段，每个阶段历时 3min，进行负荷递增实验。第一阶段速度达到 2.72km，跑台坡度 10%；第二阶段速度达到 4km/h, 跑台坡度为 12%；第三阶段速度达到 5.44km/h，坡度为 14%。这样可以分别估测出三个阶段的最大吸氧量。这种方法的好处是对儿童少年来说比较安全。因为受试者可以根据自己的体能状况，在任何阶段停止。如停止在第一阶段，则 7 岁、9~10 岁、11~12 岁和 13~14 岁的的儿童少年的最大吸氧量分别为 20.2、18.6、18.1 和 18.2ml/kg/min（不分男女）；如停止在第二阶段，则 7 岁、9~10 岁、11~12 岁和 13~14 岁的的儿童少年的最大吸氧量分别为 26.4、25.5、24.4 和 28.3 ml/kg/min；停止在第三阶段，则 7 岁、9~10 岁、11~12 岁和 13~14 岁的的儿童少年的最大吸氧量分别为 40.2、36.5、36.8 和 39.2 ml/kg/min。还可以用在活动跑台上跑或走来估测儿童少年的最大吸氧量，这样就更安全了，关键是速度因素。如表 6 所示。

表 6 在活动跑台上跑和走的相关耗氧量

运动阶段	速度（km/h）	坡度（%）	儿童少年的耗氧量（ml/kg/min） 走	跑
**1**	2.72	10	20.8	30.5
**2**	4.00	12	28.0	35.0

3	5.44	14	40.5	41.0
4	6.72	16	48.0	57.5

(依 Roy. J. Shephard《Physical Activity and Growth》, 1982)

Shephard 还提供了北美白人儿童少年相应年龄的最大吸氧量大样本调查的总平均值，见表 7。

表 7　北美白人儿童少年相应年龄的最大吸氧量

性别	相应年龄的最大吸氧量（ml/kg/min）						
	6 岁	8 岁	10 岁	12 岁	14 岁	16 岁	18 岁
男孩	47	51	49	47	48	48	50
女孩	50	50	43	40	38	37	38

(依 Roy. J. Shephard《Physical Activity and Growth》, 1982)

在多数情况下，国外学者也不主张从儿童少年中直接或间接测定最大吸氧量，原因是受设备条件限制，缺乏较完善的医学监督救护。所以次最大运动强度的测定就显得较为普遍，其中最多的是测定 PWC_{170}，即心率 170 次/min 时的工作耐力。表 8 显示加拿大儿童青少年用自行车功量计所测定的 PWC_{170} 的数据。

表 8　加拿大不同年龄儿童青少年的 PWC_{170} 值

年龄（岁）	男孩		女孩	
	绝对值（w）	相对值（w/kg）	绝对值（w）	相对值（w/kg）
7	50	1.96	39	1.57
8	57	2.08	47	1..74
9	63	2.08	50	1.68
10	70	2.09	55	1.66
11	81	2.16	59	1.65
12	90	2.18	68	1.62
13	107	2.28	74	1.50
14	119	2.26	71	1.39
15	121	2.10	73	1.35
16	139	2.20	75	1.39
17	143	2.18	78	1.69

(依 Roy. J. Shephard《Physical Activity and Growth》, 1982)

3. 3. 1. 2 关于用最大吸氧量评价有氧运动能力的信度的研究

在 Boileau 等人于 1977 年研究了在男性青少年中用最大吸氧量来评价有氧运动能力的信度之后，James 等人又于 1996 年研究了在女性青少年中用最大吸氧量来评价有氧运动能力的信度问题。该研究用 32 名 10~16 的健康女孩为对象，以活动跑台实验测定最大吸氧量。受试者在相隔 7 天的情况下进行两次测试。测试时，用跑台的速度和坡度来渐进性增加运动负荷，每一个负荷阶段维持 1min。第一阶段从 2.3 英里/小时、0 坡度开始，速度为每分钟增加 0.5 英里/小时，直到

速度达到 6 英里/小时为止，坡度每分钟增加 3%。实验时用语言鼓励受试者尽可能完成工作到力竭。实验时，用气体分析仪收集并分析受试者运动过程中的氧耗和二氧化碳排出量。然后对相隔 7 天的所有数据进行方差分析。结果表明最大吸氧量的信度很高（0.90），其标准误仅为 1.5~4.5%。

3．3．2 国内儿童少年有氧运动能力及其测定

国内对儿童少年有氧运动能力及其测定方法的研究也有较长的历史，近 20 年来的研究更为深入。北京医科大学儿童青少年卫生研究所关明杰等在 1990 年代采用跑台 Bruce 法对 445 名 10~18 岁的男女学生进行最大吸氧量的直接测定，初步制订了该年龄段儿童青少年最大吸氧量的常模，并确定了评价等级，将最大吸氧量的值分为优（> X＋2S）、良（X＋S～X＋2S）、中（X － S～X＋S）、及格（X －2S～X － S）、差（< X －2S）五个等级。这五个等级对应的最大吸氧量的数值，本人以表 9 进行归纳总结。

表 9 国内不同年龄儿童青少年最大吸氧量等级与数值的对应表

性别	年龄（岁）	最大吸氧量（L/min）差	可	及格	良	优	最大吸氧量（ml/kg/min）差	及格	中	良	优
男	10	<1.18	1.18	1.42	1.95	2.21	<39.5	39.5	44.0	52.9	57.2
	11	<1.32	1.32	1.60	2.14	2.41	<40.5	40.5	45.5	55.2	60.2
	12	<1.52	1.52	1.82	24.1	2.70	<41.6	41.6	46.8	57.4	62.8
	13	<1.74	1.74	2.07	2.72	3.06	<42.2	42.2	48.	59.1	64.8
	14	<1.96	1.96	2.30	3.05	3.41	<43.0	43.0	48.8	60.2	66.1
	15	<2.18	2.18	2.55	3.34	3.73	<43.2	43.2	49.2	61.0	67.0
	16	<2.31	2.31	2.72	3.56	3.98	<43.3	43.3	49.5	61.3	67.3
	17	<2.42	2.42	2.84	3.67	4.09	<43.2	43.2	49.2	61.0	66.8
	18	<2.50	1.50	2.88	3.63	4.02	<43.0	43.0	48.8	60.2	66.0
女	10	<1.00	1.00	1.22	1.64	1.85	<37.9	37.9	41.8	49.1	52.9
	11	<1.24	1.24	1.46	1.91	2.13	<37.3	37.3	41.3	49.2	53.2
	12	<1.38	1.38	1.51	21.1	2.36	<36.5	36.5	40.9	49.0	53.1
	13	<1.42	1.42	1.71	2.25	2.52	<35.2	35.2	39.8	48.3	52.8
	14	<1.44	1.44	1.76	2.32	2.62	<34.0	34.0	38.4	47.3	52.0
	15	<1.43	1.43	1.74	2.38	2.69	<33.0	33.0	37.3	46.2	50.9
	16	<1.42	1.42	1.75	2.40	2.72	<32.7	32.7	36.9	45.7	50.1
	17	<1.43	1.43	1.76	2.41	2.73	<32.8	32.8	36.8	45.1	49.1
	18	<1.50	1.50	1.80	2.42	2.73	<33.8	33.8	37.2	45.1	49.0

国内另一项王步标等人的研究，报道中国南方长沙市 9~17 岁学生的最大吸氧量的测定结果，其中男生的绝对值低于北京医科大的调查结果，女生的绝对值与上述结果相吻合，这提示中国南北方的青少年不仅在形态方面，而且在机能方

面也有一定的差异。

上饶师范学院邱少霖等人2000年对153名大学生分别进行了PWC_{170}台阶试验、15min跑和12min跑测试，推算最大吸氧量。其研究结果表明，PWC_{170}台阶试验，并用卡尔普曼公式推算最大吸氧量较为合理和准确。

此外，解放军体育学院李新宇等人采用台阶实验分段追踪观察，推算了7~17岁男女学生的最大吸氧量近似值，并由此分析儿童少年有氧耐力发展的年龄、性别特征，反映了儿童少年心肺机能的纵向发展状况，提供了间接群体测量最大吸氧量的参考资料。

该研究所采用的台阶试验负荷为3min上下台阶共90次，台阶高度分别为25cm（男、女7~9岁），30cm（男10~12岁，女10~17岁），35cm（男13~17岁），40cm（女17岁且身高170cm以上），50cm（男17岁且身高160cm以上）。测试参数为试前10s心率，台阶试验后即刻10s心率，试验后2min前30s的心率，所有心率均折算为1min心率，再代入公式计算。

计算公式为：

功率N（kg·m/min）= $1.3\times P\times H\times n$ 。其中，P为体重（kg），H为台阶高度（m），n为30次/min。

最大吸氧量（绝对值）= $1.29\times\sqrt{N^e/(f-60)}-0.0084T$ ，其中N为功率，f为试验后即刻心率，T为年龄，e为自然对数。这一方法计算甚为复杂，但其结果则与一般较简易的推算结果非常接近，男、女最大吸氧量的均值范围分别为1.76~3.13和1.73~2.96 L/min。该研究指出，上述方法得出的推算值明显高于用PWC_{170}推算的数值。

4 儿童少年的BMI（体重指数）

4．1 BMI的意义与标准

BMI是Body Mass Index的简写，有直译为身体质量指数的，也有意译为体重指数的。BMI是指用一个人体重（千克）数除以身高（米）的平方的值。BMI是比利时科学家兰伯特·凯特勒（Lambert Quetelet）首先提出来的。美国人杰明·古尔德调查了美国南北战争时期的2万余名士兵的人体测量指标，证实BMI可以很好地反映身高与体重的内在关系。一般而言，身体高大者，体重也比较重，BMI正是按照身高与体重的关系来确定是否肥胖。目前BMI被国内外广泛用于

作为判断肥胖的重要标准之一，因为这种方法简便易行，在进行大规模的流行病学调查研究以及评价儿童青少年身体发育和身体成分时，更显示出优越性。

美国运动医学会（ACSM）1992 年规定：成人 BMI 低于 20 为体重过轻；在 20.1~25.0 之间为标准体重；其中在 21~23 之间为理想体重；大于 25.1 为超重；大于 30 为肥胖。而世界卫生组织 WHO1997 年规定见表 10。

表 10　WHO 关于 BMI 指数与肥胖诊断的标准

分类	BMI 值	发病危险
偏瘦	<18.5	相关的临床疾病危险性增加
正常体重	18.5~24	无直接相关的临床疾病危险
超重	>25	相关的临床疾病危险性潜在性增加
肥胖前期	25~29.9	相关的临床疾病危险性轻度增加
Ⅰ级肥胖	30~34.9	相关的临床疾病危险性中度增加
Ⅱ级肥胖	35~39.9	相关的临床疾病危险性高度增加
Ⅲ级肥胖	≥40	相关的临床疾病危险性极高

（依 Report of a WHO Consultation on Obesity，1997）

2002 年 2 月 WHO 西太区办事处、国际肥胖研究协会及国际肥胖问题专家组联合发布了《亚太地区肥胖的重新定义和处理》的指导性文件，该文件在 WHO 原来规定的基础上，针对亚太地区居民的形态和生理特点，重新修订了肥胖的标准，将 BMI 大于 23 和大于 25 分别定为超重和肥胖。

中国研究人员又根据中国人的形态和生理特点，在充分调查测试和研究以及上述标准的基础上又确定了适用于中国人的 BMI 肥胖标准。主要修订指标是确定 BMI 大于 24 为超重，大于 28 为肥胖。

有的学者认为上述指标只适用于成人，而不适用于儿童。有专家建议采用年龄相关的 BMI 值判断儿童的肥胖。国内王慧琴等人提出，采用男生 BMI 大于 18 和女生 BMI 大于 17.5 作为判定 6~12 岁学龄儿童的肥胖。

台湾学者林贵福提供了台闽地区男女性不同年龄的 BMI 正常值的测量数据，见表 11（只限儿童少年部分）。

表 11 台闽地区男女性不同年龄的 BMI 正常值的测量数据

性别	年龄	过轻	稍轻	正常	稍重	过重
	6	~15.4	15.5~15.8	15.9~16.7	16.8~18.7	18.8~
	7	~15.5	15.6~16.6	16.7~17.6	17.7~20.4	20.5~
	8	~15.2	15.3~16.5	16.6~17.6	17.7~19.9	20.0~
	9	~15.8	15.9~16.4	16.5~17.6	17.7~20.3	20.4~
	10	~16.1	16.2~17.0	17.1~18.9	19.0~22.0	22.1~
男	11	~16.8	18.9~18.3	18.4~19.9	20.0~22.7	22.8~
	12	~17.5	17.6~18.7	18.8~19.9	20.0~22.7	22.8~

	13	~17.9	18.0~19.0	19.1~20.4	20.5~23.3	23.4~
	14	~18.3	18.4~19.0	19.1~20.0	20.1~22.5	22.6~
	15	~19.0	19.1~20.3	20.4~21.5	21.6~23.5	23.6~
	16	~19.4	19.5~21.0	21.1~22.2	22.3~23.9	24.0~
	17	~19.8	19.9~21.4	21.5~22.3	22.4~24.5	24.6~
女	6	~15.0	15.1~15.7	15.8~16.5	16.6~17.9	18.0~
	7	~14.7	14.8~15.6	15.7~16.3	16.4~17.6	17.7~
	8	~15.3	15.4~16.2	16.3~16.0	17.0~18.9	19.0~
	9	~15.7	15.8~16.8	16.9~17.9	18.0~19.6	19.7~
	10	~16.0	16.1~17.2	17.3~18.7	18.8~21.2	21.3~
	11	~16.4	16.5~17.8	17.9~19.1	19.2~20.9	21.0~
	12	~17.1	17.2~18.5	18.6~19.6	19.7~21.3	21.4~
	13	~17.9	18.0~19.1	19.2~20.0	21.0~23.1	23.2~
	14	~18.0	18.1~19.4	19.5~21.3	21.4~22.9	23.0~
	15	~18.8	18.9~19.9	20.0~20.9	21.0~22.9	23.0~
	16	~19.3	19.4~20.4	20.5~22.0	22.1~24.0	24.1~
	17	~19.1	19.2~20.1	20.2~21.7	21.8~23.1	23.2~

（依林贵福，《体适能检测与评价》，2003 年）

4．2 关于用 BMI 反映肥胖程度的不同看法

对于用上述的 BMI 标准来判断肥胖的程度，国内外学者也提出了一些不同的意见或质疑。首先是按照 BMI 标准来判定会出现一些“假阳性”结果。比如，在用 BMI 标准被判定为肥胖的人中，如以水下称重法测定，却只有半数是真正属于肥胖的。因为 BMI 的决定因素只有体重与身高。体重是由内脏、骨骼、肌肉和脂肪等组织共同构成的。但所谓肥胖则主要是脂肪的问题。所以，当一个人因肌肉强壮而并非因脂肪较多所导致的体重增大时，也必然引起 BMI 值的增高，比如力量型的运动员。如对这样的人群，使用 BMI 做标准，就会把相当数量的身体脂肪较少而肌肉很强壮的人因 BMI 值较高而错判为肥胖。在相反的情况下，使用 BMI 为标准，也会引出“假阴性”的结果。比如，对于长期缺乏运动的人，由于严重的肌肉消瘦而使体重并未出现超重，导致 BMI 处于正常范围，但实际上其脂肪对肌肉的比例可能较高，如果用体脂百分比作为标准，反而属于肥胖，这就等于肥胖的漏判，造成了“假阴性”。还有人提出，儿童少年处于生长发育阶段，特别是青春期前的儿童少年身体尚未发育，水分、脂肪较多，肌肉较少，骨密度较低，若用成人的 BMI 标准来评价儿童少年，往往会过高估计其肥胖的程度。当然如果对原来的标准进行适当的调整，还是可以使用的。

5 儿童少年的肺活量

5．1 儿童少年的肺通气能力与肺活量

儿童少年的新陈代谢很旺盛，需氧量相对较大。6岁儿童每分钟每千克体重的需氧量为168 ml，14岁时需要128 ml，成人只需要96 ml。随着儿童年龄的增大，其肺泡数目不断增多，肺容量和肺活量也逐渐增大，因此其呼吸频率减慢，5岁儿童的呼吸频率为26次/ min，10岁儿童为17~22次/ min，到成人时为15~18次/ min。

因为儿童少年的胸廓比较狭窄，呼吸肌还不够发达，肌力较弱，肺泡数目较少，因此在功能上表现出呼吸表浅，肺容量和肺活量都比较小。7岁儿童的肺活量约为1000~1400 ml，11岁时达到2000 ml左右。在12岁以前男女儿童的肺活量的差异不大，男孩比女孩约多200 ml左右；13岁以后，男女肺活量的差异显著拉大，到成人时男子比女子的肺活量可大到1000 ml。我国小学生（7~12岁）的肺活量/身高指数为11.1~15.1ml/cm（男）和10.1~14.1ml/cm（女）；我国小学生肺活量/体重指数为63.2~65.2ml/kg（男）和59.1~59.5ml/kg（女）。

有资料表明（V. Seliger，1980），在有计划的学校体育锻炼活动过程中，在小学阶段时，耗氧量和肺通气量的增加幅度较小，而到初中以后体育锻炼对耗氧量和肺通气量的影响才较显著地体现出来。如表12所示。

表12　不同儿童少年运动锻炼前后心率、耗氧量和肺通气量的变化

变量	6岁		12岁		15岁	
	锻炼前	锻炼后	锻炼前	锻炼后	锻炼前	锻炼后
心率（次/min）	119	130	125	148	143	170
耗氧量（ml/kg/min）	11.1	15.2	14.2	16.6	17.4	24.0
肺通气量（l/min）	11.0	12.2	17.2	23.6	32.9	54.8

（依 Roy J. Shephard：《Physical Activity and Growth》1990）

5．2 体育锻炼对儿童少年肺活量的增强作用

肺组织在儿童时期生长发育很快，8岁时的肺容积为新生儿的8倍，到成年人时，肺容积的大小为刚出生时的20倍。适当的体育锻炼能使肺组织中的毛细血管增生，肺泡的数量增多，肺的容积增大，肺组织的弹性增强，因此肺的功能相应得到提高。

呼吸运动是在神经系统支配下，由呼吸肌的收缩与舒张而引起的胸廓节律性扩大和缩小的活动。儿童少年时期，呼吸运动的功能较差。如果经常参加体育锻炼，肌肉运动产生的 CO_2 有刺激呼吸中枢的作用，使呼吸加深加快，从而使肋

间肌、膈肌等呼吸肌得到锻炼，更加有力。呼吸肌的发育扩大了儿童少年胸廓活动的范围，使得胸腔容积增大。膈肌每下降 1 cm，胸腔容积增大 250~300 ml。

由于体育锻炼所引起的肺泡的增多，肺组织弹性的增强，呼吸肌力量的增强，胸腔活动范围的扩大等因素，共同产生的效应就是肺活量的增大。有资料表明，10 岁的少体校学生比同龄的普通学校小学生的肺活量平均大 350~400 ml。15~16 岁的少年运动员比同龄非运动员的肺活量平均大 515 ml。

6 青少年体质健康测评

6. 1 国外青少年体质健康测评

从体质测评的发展历史来看，国外的青少年体质健康测评的内容经历了从运动相关性指标向健康相关性指标转变的过程。

6. 1. 1 美国的体质健康测评

美国早在 1880~1900 年间就开始盛行体质检测法。20 世纪后美国采用田径项目广泛测定人体运动技能。1956 年美国总统艾森豪威尔成立了青少年健康总统委员会，肯尼迪期间改为健康总统委员会，约翰逊时代又改为健康与体育总统委员会。70 年代，美国政府教育局通过健康体育娱乐协会制订了《美国青少年身体素质测验标准》。1980 年初，美国总统卡特发表演说，鼓励美国人民参加体育活动，并提出一系列具体的指标。

目前，在美国比较普遍使用的体质健康测评方法，主要是有氧运动研究所 1987 年建立的、计算机程序化的一种健身计划（Fitnessgram）和“总统挑战”计划（见表 13 和表 14）。此外，由“美国健康、体育、娱乐和舞蹈协会”（American Alliance of Health，Physical Education, Recreation and Dance，AAHPERD）于 1959 年推出，并经 1965、1975、1980 和 1988 年的修订，以及美国政府教育主管部门认可的体质健康测试方案，也是目前美国最具影响力的检测方案（表 15）。从表中可以看出，最初对体质测试的理解仅限于运动能力方面，到 1985 年后选用的项目和指标都与人体健康有关，可以归纳为 4 个方面：（1）心肺功能；（2）肌肉力量和耐力；（3）身体柔韧性；（4）身体组成。经过 30 年的筛选，基本完成了从“测评运动技术指标”向“测评健康指标”的过渡。

表 13　美国健身计划中的体质测评项目

体质特性	测评项目
有氧运动能力	1 英里（相当于 1600 米）跑
肌力和肌耐力	仰卧起坐、俯卧撑、引体向上、屈臂悬垂

柔韧性	背弓撑起、单腿体前屈和双手肩后交叉
体脂含量	皮下脂肪测定、体重/身高比值

表 14　　总统挑战计划中的体质测评项目

体质特性	测评项目
有氧运动能力	1 英里（相当于 1600 米）跑或走
肌力和肌耐力	仰卧起坐、引体向上、屈臂悬垂
柔韧性	分腿坐式体前屈或并腿体前屈
灵敏性和爆发力	折返跑

表 15　　美国健康、体育、娱乐和舞蹈协会（AAHPERD）体质测评方案

体质特性	1959 年项目	1965 年项目	1975 年项目	1980 年项目	1988 年项目
心肺功能	600 码跑走	600 码跑走	600 码跑；<br>1600 m 跑或<br>9 分钟跑；<br>2400 m 跑或<br>12 分钟跑；	1600m 跑或<br>9 分钟跑；<br>2400 m 跑或<br>12 分钟跑；	1600 m 跑
身体组成				皮褶厚度总和（肱三头肌和肩胛下缘）	皮褶厚度总和（肱三头肌和肩胛下缘）或 BMI 指数
柔韧性				坐位体前屈	坐位体前屈
肌力和肌耐力	仰卧起坐<br>引体向上	仰卧起坐<br>引体向上（男）<br>屈臂悬垂（女）	1min 仰卧起坐<br>引体向上（男）<br>屈臂悬垂（女）	1min 仰卧起坐	1min 仰卧起坐<br>引体向上（男）
无氧耐力	立定跳远<br>50 码快跑	立定跳远<br>50 码快跑	立定跳远<br>50 码快跑		
灵敏性	折返跑	折返跑	折返跑		
运动技巧	垒球投掷	垒球投掷			

6．1．2　欧洲各国的体质健康测评

欧洲各国在 1977 年成立了欧盟体育振兴协会，并对学校体育提出两点要求：（1）欧洲各国学校体育采取统一标准；（2）由教师对学生体质健康测试和评价。欧洲体质测评的内容包括体格检查和体质测试，共选择了 10 个项目（见表 16），还制定了测评手册。

表 16　　欧洲各国体质测评项目

内容	身体素质	测评项目
体格检查		身高<br>体重<br>皮脂（肱二、三头肌、肩胛下、腰、小腿部）
全身耐力	心血管系统耐力	20 米往返跑或 PWC_{170} 机能实验
肌力	静态肌力	握力
	爆发肌力	立定跳远
肌耐力	上臂肌耐力	屈臂悬垂时间
	躯干肌耐力	30 秒引体向上
灵敏性	全身灵敏性	往返跑（5 米往返，10 次）

	上肢灵敏性	打击目标
柔韧性	躯干柔韧性	直腿坐位体前驱屈
平衡性	全身平衡性	闭眼单腿站立时间

6. 1. 3　日本的体质健康测评

日本政府对青少年的体质健康测评也很重视，文部省曾在 1961 年颁布过《体育振兴法》。和《体力测验》测试内容由体力诊断测试、基础运动能力测试和个别项目的运动能力测试三部分组成。日本的“体力测定”，基本上是国际体力研究会（ICPFR）和国际生物学发展规划理事会（IBP）两大国际组织有关测试项目与指标的综合，见表 17 所示。

表 17　美国、日本与 ICPFR 和 IBP 体质测试指标的对比

类别／项目	ICPFR	IBP	日本		美国	
			体力诊断测验	运动能力测验	1958 年指标	1985 年指标
耐力		哈佛式台阶测验	踏台上、下运动			
弹跳力	立定跳远	立定跳远	垂直跳	立定跳远	立定跳远	
速度	50 米疾跑	50 码疾跑		50 米疾跑	50 码冲刺跑	
力量	引体向上（男）<br>屈臂悬垂（女）	引体向上（男）<br>斜身引体（女）		引体向上（男）<br>斜身引体（女）	引体向上<br>引体向上	引体向上<br>引体向上
灵敏性	穿梭往返跑（10 米）	穿梭往返跑（10 码）	反复跨越	持、运球穿梭往返跑	往返跑	
腹部力量	仰卧起坐（30 秒）	仰卧起坐（1 分钟）			仰卧起坐	仰卧起坐（直腿变屈腿）
长距离跑	1000 或 2000 米跑（男）<br>800 或 1000 米跑（女）<br>600 米跑（儿童）	600 码		1500 米跑（男）<br>1000 米跑（女）	600 码	1 英里跑
测力器测验	握力	握力、背力，拉力、推力，伸腿力量<br>屈腿力量	握力<br>背力			
投掷力		投垒球		手（垒）球	手（垒）球	
柔韧性	立体体前屈		立体体前屈<br>俯卧上体后仰			坐体体前屈

（依于可红《国家中小学生体育与健康教育个体评价标准的研究》）

6. 2 中国的体质健康测评状况

2000 年我国颁布了国民体质健康监测指标，见表 18。

表 18 2000 年全国国民体质监测指标

内容	儿童	成人<br>20~39 岁	成人<br>40~59 岁（男）<br>40~54 岁（女）	老年人<br>60~69 岁（男）<br>55~69 岁（女）
身高	*	*	*	*
坐高	*			
体重	*	*	*	*
胸围	*	*	*	*
腰围		*	*	*
臀围		*	*	*
皮褶厚度	*	*	*	*
心率	*	*	*	*
血压		*	*	*
肺活量	*	*	*	*
台阶实验		*	*	
立定跳远	*			
网球掷远	*			
坐位体前屈	*	*	*	*
10 米×4 折返跑		*		
走平衡木	*			
双脚连续跳	*			
握力		*	*	*
背力		*	*	
纵跳		*		
闭目单足立		*	*	*
灯光反应时		*	*	*
俯卧撑		*		
1min 仰卧起坐		*		

（依王健《运动生理学研究技术》，2001 年）

注：* 者为测评项目，无* 者为非测评项目。

为了专门针对学生的体质健康测评，1987 年国家教委颁发了《中学生体育合格标准的试行办法》。1989 年 12 月 9 日，国务院批准《国家体育锻炼标准实施办法》，1990 年 1 月 6 日公布实施。1991 年修订了《中学生体育合格标准实施办法》，1990 年又颁发了《大学生体育合格标准》及《大学生体育合格标准实施办法》。这些政策和措施，对加强学校体育工作，提高青少年体质健康水平起到了很大的推动作用。然而随着时代的进步，这些措施与社会发展的要求还有些不相适应，如在测试的内容方面存在着一定的局限性，不能全面反映学生的体质和

健康状况。因此建立一个比较全面、科学、简单、实用的学生体质健康测评体系很有必要。

6．3 最新《学生体质健康标准（试行方案）》及《实施办法》

6．3．1 文件精神

2002 年 7 月 4 日，国家教育部和国家体育总局联合颁布教体艺 [2002]12 号文件，向全国印发《学生体质健康标准（试行方案）》及《〈学生体质健康标准（试行方案）〉实施办法》。

文件强调“《标准》是促进学生体制健康发展、激励学生积极进行身体锻炼的教育手段，是学生体质健康的个体评价标准，也是学生毕业的基本条件之一。各地教育行政部门和学校应把《标准》实施作为学校体育工作的重要内容，积极宣传、加强管理、认真执行。”文件指出，“《标准》是《国家体育锻炼标准》的组成部分，是《国家体育锻炼标准》在学校的具体实施。因此，在实施《标准》的同时，原《国家体育锻炼标准》的内容不再执行，各地教育行政部门和学校仍按照原《国家体育锻炼标准》的实施办法，向体育主管部门报送《标准》的达标数据。”文件还要求“从 2003 年新学年开始，各地实施《标准》的比例扩大到 50% 的中小学和所有高等学校”，并明确提示“教育部将于 2003 年开始，对各地实施《标准》的学校进行抽查，并公布抽查结果”。

6．3．2 《实施办法》中有关小学生组的规定

《学生体质健康标准》》及《实施办法》中对小学生的测评项目的规定以及各项目的分值比例如表 19。

表 19　小学组学生体质健康标准的测评项目与各项分值比例

年级	测评项目	分值比例	备注
一、二年级	身高标准体重	20	必测
	坐位体前屈，掷沙包	40	选测其中一项
	50 米跑、25 米×2 往返跑、立定跳远<br>跳绳、踢毽子	40	选测其中一项
三、四年级	身高标准体重	20	必测
	坐位体前屈、掷实心球、仰卧起坐	40	选测其中一项
	50 米跑、25×2 往返跑、立定跳远、跳绳	40	选测其中一项
五、六年级	身高标准体重	10	必测
	肺活量体重指数	20	必测
	400 米跑、50 米×8 往返跑、台阶试验	30	选测其中一项
	坐位体前屈、值时新球、仰卧起坐<br>握力体重指数	20	选测其中一项

50 米跑、跳绳、篮球运球<br>足球颠球、排球垫球	20	选测其中一项

上各年级的总得分均为 100 分。总分 86 分以上为优秀；76~65 分为良好；60~75 分为及格；59 分及以下为不及格。每一项的得分标准有《细则》规定，可以查对。

第二部分　实验研究

1 研究目的

有关成年人进行耐力锻炼,对其最大吸氧量(VO2max)的影响迄今已有很多的研究报道[1]。这些研究结果近乎一致地显示经过一段时间的有氧锻炼,VO2max 出现不同程度的增加。另外,对于相同的锻炼方案,男女之间的这种慢性运动锻炼反应似乎并不存在明显的差异[6],[2]但是对于儿童有关这方面的研究工作很少,而且得到的结果也不尽一致。然而文献资料表明儿童耐力锻炼反应是存在的,但对于同样性质的耐力锻炼,儿童的耐力锻炼反应大都比成年人的小，而对于多久的锻炼时间会对有氧工作能力产生影响是一个值得探讨的问题[7][3]。考虑到青春期前的儿童在身体形态、运动素质以及生理功能方面的性别差异不大，本研究不做男女性别差异之间的比较分析。研究工作的目的就是在不考虑性别差异的情况下，一个为期三个月的耐力锻炼方案对儿童有氧工作能力的影响。从而为就如何[4]提高小学生的有氧工作能力提供依据。

当前世界各国在注重体质的同时，都非常关注青少年、儿童的体质健康状况，我们国家自 1987 年以来在党和国家政府的领导下，先后组织了六次大规模的学生体质健康测试，反映出来的问题是学生的体质状况从 1995 年全国体质健康调查起身体素质在明显下降，特别反映在有氧工作能力和肥胖上[5]。国家加强了对学生体质的调查与研究，用科学的指标评价学生体质健康状况。目前，由于缺乏

1 Baxter J et al. The development of aerobic power in young athletes. J Appl Physiol 1993,75:1160-1167.
Kobayashi K et al. Aerobic power as related to body growth and training in Japanese boys: a longitudinal study. J Appl Physiol 1978,44:666-672.
Mahon AD,Vaccaro P. Cardiovascular adaptations in 8 to12 years old boys following a 14 week running program. Can J Appl Physiol 1994,19:139-150.
Kemper HCG,Van de Kop H.Entrainement de puissance maximale aerobie chez les enfants prepuberes et puberes. Science & Sport 1994,10:29-38.
Baquet G et al. Effects of high intensity intermittent training on peak VO(2) in prepubertal children.Int J Sports Med 2002,23:439-444.

2 Lewis DA et al. Physiological differences between gen-ders. Implications for sports conditioning. Sports Med 1986,3:357-369.
3 Rowland WT. Aerobic responses to endurance training in prepubescent children: a critical analysis. Med Sci Sports Exerc 1985,17:493-507.

5 中华人民共和国教育部　2005 年全国学生体质与健康调研结果公告 vol.4.no.1 2007

运动而引起的“现代文明病”的滋生与肥胖儿的不断增加，以及由此引发的脑血管病，冠心病等心血管疾病的增多。

2 研究对象与研究方法

2.1 研究对象

温州市瑞安安阳实验小学四年级学生,平均年龄 10.3 岁，共 122 人，男生 69 人，女生 53 人，在规定实验组人数的前提下随机分为实验组和对照组。其中对照组 93 人，（男 54 人，女 39 人）；实验组 29 人，（男 15 人，女 14 人）。锻炼后实验组有效人数为男生 15 人，女生 14 人。事先了解学生的身体状况，排除有身体外伤以及有慢性疾病的学生。

2.2 研究方法

2.2.1 文献资料法

本研究参阅了国内外的大量有关体育测量学，学生体质调研和评价以及统计学等方面的文献资料，这些文献为本文奠定了坚实的理论基础。资料来源，上海师范大学图书馆，上海师范大学体育学院资料室，信息网络等。

2.2.2 实验测试法

实验法是本文最重要的研究方法。在瑞安实验小学体育教师的指导下对实验组进行三个月的有氧耐力锻炼活动。所有的测试对象在锻炼前和锻炼后都要进行以下项目的检测：身高、体重、胸围、50 米、立定跳远、坐位体前屈、肺活量、PWC170 台阶试验。测试时间为 2008 年 9 月中旬-12 月中旬，三个月，地点温州市瑞安安阳实验小学。

2.2.3 数理统计法

所有数据均采用 Windows 2003 Excell 统计软件进行处理,测试结果均以“平均数±标准差”表示,两样本间差异检验采用独立样本 t 检验分析(Independent-t Test), 两样本间相关性用 Pearson 相关分析(r 值),并建立回归方程。以 $P<0.05$ 作为差异具有显著性的标准,以 $P<0.01$ 作为差异具有非常显著性的标准

2.2.4 比较分析法

对测试的数据进行统计学处理后，先将每组的前后数据进行比较分析，再将组与组之间进行比较分析，结果均进行 T 检验。

2.3 测试项目与测试方法

2.3.1 测试项目

直接测量项目：身高、体重、胸围、50 米跑、立定跳远、坐位体前屈、肺活量、PWC170 台阶试验。

间接推算和计算项目：最大吸氧量绝对值、最大吸氧量相对值、BMI

2.3.2 测试方法

所有直接测量项目参照《学生体质健康标准》所要求的进行测量。

BMI（身高体重指数）=体重/身高 ^{2}

间接推算最大吸氧量绝对值（L/min）：采用卡尔普曼公式[6],先求出 PWC170 台阶实验的功率(单位:kg.m/min),然后出功率推算 VO2max(L/min)，公式：

N1 或 N2=[(P×H×n)/t]×4/3 t

$PWC_{170}W$=[N1+(N1-N2)]×[（170-f1）/（f2-f1）]

$PWC_{170}VO_2max=1.7\times PWC_{170}+1240$

其中 P 为体重;H 为台阶高度；n 为上下台阶次数；T 为时间；f_1 和 f_2 分别为第一次负荷和第二次负荷后即刻 1 min 心率；N1 和 N2 为第一次和第二次负荷功率；$PWC_{170}VO_2max$ 为 PWC_{170} 台阶实验推算出的 VO_2max 值。

最大吸氧量相对值(ml/kg.min) = 最大吸氧量绝对值/体重

台阶实验具体方法：受试学生在测试前要求处于相对安静状态,在节律为每分钟 22.5 次的节奏下，按节拍器指挥完成前后两次负荷(上下台阶),第一次负荷台阶高度为 25cm,运动 3min,中间休息 3 min；第二次负荷台阶高度 35cm，运动 3min。在两次负荷后即刻分别测试立姿 10 s 脉搏，再乘以 6,为第 1 min 心率。台阶高度的选择参考于可红等著的《国家中小学体育与健康教育个体评价标准的研究》。

2.4 分组方法

2.4.1 实验组

每周 3、4 次耐力锻炼，锻炼项目包括跑步、跑步为主的游戏、跳绳等，一个项目或多个项目同时进行，时间为下午 4 点-6 点之间，强度要求达到 65%-70%左右的 HR_{max}，（最高心率=220—年龄数,），即心率达到 137-147 次/min，保持这

[6] 邱少霖等 PWC170 台阶实验、15min 跑和 12min 跑与间接推算 VO2max 的研究 北京体育大学学报 2001 年 3 月 第 24 卷第 1 期

刑文华等 体育测量与评价 北京体育学院出版社 1986 年 1 月

个心率运动至少 20 分钟。在锻炼进行中测量心率并以此为标准增减运动强度。运动后积极放松，包括肌肉韧带的拉伸和捶打。正常的上课学习不耽误。

2.4.2 对照组

进行日常的生活，除了学校安排的一周三次体育课以外，不要求任何与体育锻炼相关的身体活动。

3 实验研究与分析

3.1 锻炼前总体测试学生各指标值与全国相应年龄儿童少年常模值的比较

2005 年全国学生体质调研数据[7]反应了全国学生的体质状况，从一定意义上代表了近年全国学生各个体质测试项目的平均水平，本文将所测体质项目与之一一进行比较分析，具有研究意义。测试数据全部选择锻炼前的测量数据，比较情况见表 1 所示：

表 1 总体测试学生与全国男女学生的情况比较

	男		女	
	测试对象（69）	全国	测试对象（53）	全国
身高（cm）	141.4±6.28	141.07±6.66	135.21±5.8	141.48±7.16
体重 (kg)	34.16± 6.4*	36±8.63	31.07±3.43**	33.9±7.54
胸围 (cm)	63.86±5.43**	68±7.55	62.9±6.2	65.6±6.78
50 米 (s)	9.43±0.97*	9.71±1	10.9±1.18**	10.14±1.01
立定跳远 (cm)	163.33±18.82**	153±19.01	147.88±15.37*	142.45±18.51
坐位体前屈(cm)	8.69±7.17**	5.05±6.04	11.33±6.27*	9.13±5.98
肺活量 (ml)	1656.65±415.54	1641.2±463.1	1630.75±347.89**	1469.9±441.1

P<0.05 “ * ”,显著性差异； P<0.01 “**”，非常显著性差异。

如表 1 所示，测试七个项目中有两个显著性差异三个非常显著性差异。男生方面，胸围、立定跳远、坐位体前屈与全国的相比呈非常显著性差异（P<0.01），体重和 50 米为显著性差异（P<0.05）;女生方面，体重、50 米跑、肺活量与全国相比为非常显著性差异（P<0.01），立定跳远和坐位体体前屈为显著性差异（P<0.05）。其中男女都有差异的为体重、50 米、立定跳远和坐位体前屈。测试对象的胸围男女差别不到 1 厘米，而全国的差距在两厘米以上；50 米和立定跳远反映人体的速度和下肢爆发力，测试对象男女都比全国的好，其中 50 米女生差异性更显著，立定跳远男生差异性更显著；坐位体前屈主要反映人体的腰部柔韧性，检验结果显示，测试对象男女生的都比全国的好，其中测试女生与全国女

[7] 2005 年全国学生体质与健康调研报告 高等教育出版社。2005.12

生的差别程度更大。

结果表明，测试学生的身高与全国学生进行比较都没有统计学差异，但从平均水平上看出略比全国平均水平差；而在速度、下肢爆发力及腰部柔韧性上与全国相比具有统计学差异，比全国好。综合分析原因可能为，全国的测试包括南方和北方学生，而本文的测试对象全部为南方学生。南北方学生在身体形态，生理机能，身体素质上都存在一定的差异。全国及本文测试学生都是非运动员，都没有经过特殊的身体素质锻炼，本人推断两者在这两个项目上的差距很可能是因为体重的关系。北方学生在体型方面的数值，包括体重、胸围要比南方学生的大，这跟气候环境、饮食、生活习惯等因素有关。

3.2 锻炼前总体测试学生最大吸氧量的标准等级分布状况

本文的测试对象都为青春期前儿童，平均年龄为 10.3 岁，采用卡尔普曼 PWC170 台阶试验公式推算法对最大吸氧量的进行间接测定，这种预测价值是建立在这样一个基础上的:PWC170 的个体差异反映了每搏最大输出量的个体差异，也就是影响运动员 VO2max 个体差异的基本因素。用 PWC170 推算 VO2max 的方法在体育调查研究中一直被用作评定少年儿童有氧工作能力的指标，有研究指出在对象为 10~11 岁的儿童时，PWC170 是评价有氧工作能力的一种有效方法[8]。

本文采用北京医科大学关明杰[9]等所制定的10—18岁青少年最大有氧活动能力的评价标准，将在锻炼前所测得的所有男女生的最大吸氧量绝对值和相对值的值以百分比占有率的方式标出。关明杰等人采用的是直接测最大吸氧量的方式—呼吸测定法，应用 JaegerLE/6 型步行机(自动倒转固定跑道)进行运动，给予定量运动负荷，运动到极限负荷达力竭为止，测试过程中的气体分析使用 Jaeger 自动气体分析仪(Ergooxyscreen)。因此结果完全具有比较分析研究的意义。结果见表 2 。

表 2 总体测试学生 VO_{2max} 推算值不同等级百分比一览表

男生	人数（69）	百分比	女生	人数（53）	百分比
**最大吸氧量绝对值**			**最大吸氧量绝对值**		
及格（1.18—1.41）	11	15.9	及格（1.0—1.21）	7	13.2

[8] 李见刚 PWC170 对评价少年儿童有氧工作能力的有效性研究 山东体育科技 1996 年第 3 期

[9] 关明杰等，10~18 岁儿童青少年最大吸氧量正常值[J]，体育科学，1995（15）5：47~51

中 （1.42 —1.94）	29	42	中 （1.22—1.63）	24	45.3
良 （1.94—2.20）	21	30	良 （1.64—1.84）	18	34
优 （2.21 以上）	8	11.6	优 （1.85 以上）	4	7.5
**最大吸氧量相对值**			**最大吸氧量相对值**		
及格（40—43）	18	26.1	及格 （38—41）	7	13.2
中 （44—52）	17	24.6	中 （42—48）	19	35.8
良 （53—57）	25	36.2	良 （49—52）	21	39.6
优 （57 以上）	9	13	优 （53 以上）	6	11.3

如表 2 所示，男女生的最大吸氧量绝对值和相对值的各个等级所占百分比中，中等所占百分比最多。就具体各项而言，男生的最大吸氧量绝对值，中等和良所占百分比比较大，两者合起来达到了 72%，及格和优所占的百分比较低，优更低，11.6%，最大吸氧量相对值，良所占百分比最大，36.2%，及格和中等差不多，分别为 26.1%和 24.6%，优最少，13%。女生的最大吸氧量绝对值，中等所占百分比最大，将近占到一半，其次是良和及格，分别为 34%和 13.2%，优最少，仅有 7.5%；最大吸氧量相对值，中等和良所占百分比相差不大，分别为 35.8%和 39.6%，优最少，11.3%。就最大吸氧量绝对值与相对值的各等级所占比百分比而言，相对值的百分比情况比绝对值好。儿童的最大吸氧量绝对值随年龄的增长而增加，而最大吸氧量相对值却是随年龄的增长而下降的，因此青春期前的儿童最大吸氧量以相对值表示时偏大，有时甚至会超过大人[10]。所以在上表中最大吸氧量相对值所占百分比比最大吸氧量绝对值好是合理的，还有可能跟瑞安安阳实验小学的学生的体重较轻有关（见表 1 与全国同年龄男女学生的比较分析）。

3.3 锻炼前对照组和实验组各测试结果与分析：

测试项目来自《学生体质健康标准》有关小学组的规定项目，再加上与有氧工作能力相关的肺活量、PWC170、最大吸氧量绝对值和最大吸氧量相对值。测试值及其比较如表 3 所示：

表 3 锻炼前对照组和实验组各项测试结果一览表

	对照组		实验组	
	男（54）	女（39）	男（15）	女（14）
身高（cm）	141.6 ± 6.32	139.6 ±5.8	140.73 ± 6.3	140.8.±5.9
体重 (kg)	34.31 ± 6.63	31.08 ± 4.24	33.6 ± 5.69	31±3.14

[10] 邓树勋，王健，乔德才主编. 运动生理学[M]，北京，高等教育出版社，2005

胸围 (cm)	64.18 ± 5.64	62.79 ± 5.5	62.67 ± 4.76	63.28 ± 4.52
50 米 (s)	9. 47 ± 0. 95	10.92 ±31.03	9.27 ± 0.89	11.23 ± 1.23
立定跳远 (cm)	162. 1± 18. 58	148.44±14.7	167.54± 19.75	146.36 ± 17.58
坐位体前屈(cm)	9.99 ± 6	13.67±5.58	8.09 ± 6.9	11.81 ±5.73
肺活量 (ml)	1643.±8429.03	1625.39±367.64	1662.8±295.44	1644.86±165.36
PWC_{170} (kg.m/min)	258.8 ± 63.72	249.8±68.40	251.05±48.08	245.87 ±54.62
VO_{2max} (L/min)	1690.4±160.07	1630.32±122.27	1682.7±138.32	1625.2 ± 133.3
VO_{2max}(ml/kg.min)	45.76 ± 7.9	45.67±6.31	48.05± 6.9	48.47 ± 4.6
BMI	17.11 ± 2.93	15.6±1.96	16.97 ± 2.8	15.73 ± 1.05

对锻炼前实验组和对照组进行以身体形态、运动能力、身体机能三方面九个项目的测试，各个项目测试结果见表 3，各项指标之间没有显著性差异（P>0.05）。原因可能为，测试前排除了身体功能有缺陷的学生；对象都为在校有三节体育课的小学四年级学生，除此之外在校没有任何强加的锻炼。这样的结果有利于与经过锻炼的实验组的比较分析。实验组和对照组的最大吸氧量相对值没有统计学差异，但是从平均值上看实验组比对照组的数值稍大，从平均数上看最大吸氧量绝对值和体重都没有明显差异，可以初步解释为，试验组男女生的最大吸氧量初始水平比对照组稍好。在身体形态方面，对照组男女的身高体重胸围值比实验组好，女生在身体形态方面相差不大。

3.4 锻炼前后对照组的各指标差异性分析

对照组在三个月时间内照常生活学习，除了学校的体育课没有刻意要求参加于身体锻炼有关的活动，之后再测量各项指标，并与三个月前的各项指标进行对比，比较情况如表 4：

表 4 锻炼前后对照组的各指标差异性分析

	锻炼前		锻炼后	
	男(54)	女(39)	男(54)	女(39)
身高（cm）	141.6 ± 6.32	139.6 ±5.8	143.1±6.26 *	141.3±5.7
体重 (kg)	34.31 ± 6.63	31.08 ± 4.24	35.18±6.39 *	32.36±3.7 *
胸围 (cm)	64.18 ± 5.64	62.79 ± 5.5	64.25±5.8	63.78±5.06
50 米 (s)	9. 47 ± 0. 95	10.92 ±31.03	9.41±1.1	10.78±1.17
立定跳远 (cm)	162. 1± 18. 58	148.44±14.7	162.32±18.77	148.60±15.2
坐位体前屈(cm)	9.99 ± 6	13.67±5.58	10.02±5.88	13.45±5,56
肺活量 (ml)	1643.±8429.03	1625.39±367.64	1650.83±430	1630±166.74
PWC_{170}(kg.m/min)	258.8 ± 63.72	249.8±68.40	263.37±69.13	250.54±71.8
VO_{2max} (L/min)	1690.4±160.07	1630.32±122.27	1668.76±193.35	1641.21±120.1
VO_{2max}(ml/kg/min)	45.76 ± 7.9	45.67±6.31	45.81±8.2	48.75±5.

BMI	17.11 ± 2.93	15.6±1.96	16.87±3.5	16.58±2.9

P<0.05 " * ",显著性差异

男女生的身高体重在锻炼后都有一定的增长，不管是男生还是女生在三个月后身高和体重平均值都有较大的增长，男女生身高平均值分别增长 1.5cm、1.7cm，体重平均值分别增长 0.87kg、1.28kg。其中男生的身高和体重有显著性差异（P<0.05），女生的体重有显著性差异（P<0.05），身高没有统计学差异。其他各项指标都有一定的上下波动，变化幅度不大。

分析原因可能为，10 岁的男女学生处于生长发育期，身高体重有较快的增长，在没有任何附加因素影响下是生理自然增长的结果。儿童身体素质与有氧工作能力指标在自然增长情况下是波浪式、非等比的增长[11]。在没有其他施加因素的情况下，在三个月时间内，各数值的波动没有形成显著性差异，不具有代表性，不具有比较研究的价值。

3.5 锻炼后实验组和对照组各指标的差异性分析

3.5.1 比较情况

表 5 锻炼后实验组和对照组各指标的差异性分析

	实验组		对照组	
	男（15）	女（14）	男（54）	女（39）
身高（cm）	141.9±7.2	142.07±5.8	142.45±6.26	140.26±5.7
体重 (kg)	34.17±5.48	31.93±5.04	35.18±6.39	32.36±3.7
胸围 (cm)	63.73±4.4	64.14±5.0	64.25±5.8	63.78±5.06
50 米 (s)	8.87±0.78	10.8±1.14	9.41±1.1	10.78±1.17
立定跳远 (cm)	179.5±20.74	158±10.81	162.32±18.77 *	148.60±15.2*
坐位体前屈(cm)	8.25±8.52	11.44±4.97	10.02±5.88	13.45±5.56
肺活量 (ml)	1885.4±273.8	1764.79±184.42	1650.83±430*	1630±166.74*
PWC_{170}(kg.m/min)	285.95±32.1	299.08±75.43	263.37±69.13	250.54±71.8*
VO_{2max} (L/min)	1781.62±128.5	1728.31±127.68	1668.76±193.35*	1641.21±120.1*
VO_{2max}(ml/kg/min)	54.37 ±6.7	50.07±4.26	45.81±8.2*	48.75±5.
BMI	16.98±2.69	17.43±1.58	16.87±3.5	16.58±2.9

P<0.05 " * ",显著性差异； P<0.01 "**"，非常显著性差异。

经过三个月的锻炼，实验组在几个项目上的成绩明显比对照组好。实验组和对照组的男生在立定跳远、PWC170、最大吸氧量绝对值和相对值上有显著差异（P<0.05）；女生在立定跳远、肺活量、PWC170、最大吸氧量绝对值上有显著性

[11] 邓树勋，王健主编，高级运动生理学——理论与应用[M]，北京，高等教育出版社，2003

差异（p<0.05）。男女在身高体重上都没有统计学差异，但是在平均值上，对照组男生的身高体重比实验组大，实验组女生身高比对照组高，体重比对照组轻。

表 5 数据中，实验组和对照组男女生在身高和体重上都没有统计学差异，说明在三个月的时间内不管有没有受锻炼因素的影响，身高和体重的增长与生理的自然增长有很大的关系。从增长比例上看，对照组男生的身高体重胸围值都比实验组大，这可能是由对照组的原始数值必实验组大造成的（见表 3）。虽然长跑不属于速度、爆发力的特定锻炼项目，但能使腿部力量增长，关节灵活度增加，身体的协调能力加强，身体柔韧性提高，因此在一定程度上能够促进立定跳远成绩的提高。在 PWC17、最大吸氧量上的显著性差异更能说明，三个月的长跑锻炼对实验组起到了积极的影响，提高了他们的有氧工作能力。

3.6 锻炼前后实验组各指标测试值的差异性比较

表 6 锻炼前后实验组有氧工作能力相关指标的差异性分析

	男（15）		女（14）	
	前	后	前	后
肺活量(ml)	1662.8±295.43	1885.4±273.81*	1644.86±165.4	1764.48±184.4
PWC_{170}(kg.m/min)	251.06± 48.5	285.92± 42.21*	245.87±54.62	299.37 ±68.82*
VO_{2max} (L/min)	1682.6 ±138.32	1781.62±128.5*	1625.2 ±133.3	1728.3±127.68*
VO_{2max}(ml/min.kg)	48.05 ±6.9	54.37 ±6.7	48.47± 4.6	50.07 ±4.26

P<0.05 “ * ”,显著性差异；P<0.01 “**”，非常显著性差异。

如表 6 所示，男生在强化锻炼后肺活量、PWC170、最大吸氧量绝对值与锻炼前存在显著性差异（P<0.05），最大吸氧量相对值与锻炼前不存在显著性差异(P>0.05);女生方面，PWC170 和最大吸氧量绝对值与锻炼前存在显著性差异（P<0.05），肺活量和最大吸氧量相对值与锻炼前不存在显著性差异（P>0.05）。

结果表明，在经过三个月的锻炼，男女生 PWC170 值都增加了。分析原因为男生的肺活量受运动锻炼的影响比女生大；卡尔普曼公式推算 PWC170 受两个因素的影响，一是两次台阶试验后的一分钟心率以及两次心率的差，二是体重。PWC170 与体重成正比关系，体重越重所得的值越大；PWC170 与两次台阶的心率差成反比，差值越小所得值越大。锻炼后男女生的体重都有一定程度的增加，因此，PWC170 的提高主要在于心率的降低以及两次心率差的缩小，这表明，心血管在亚极限运动后的恢复能力增强了，人体的有氧工作能力得到提高，具有研究意义。

3.7 锻炼前后实验组相关性态与运动素质的测试值的差异性分析

表 7 锻炼前后实验组相关性态与运动素质的测试值的差异性分析

	男（15）		女（14）	
	前	后	前	后
身高(cm)	140.7±6.3	142.4±7.2	140.8±5.9	142.6±5.8
体重(kg)	33.6±5.69	34.17±5.48	31±5.14	31.93±5.04*
BMI	16.97±2.8	16.98±2.69	15.73±1.05	15.43±0.93
50 米（s）	9.27±0.89	8.87±0.78	11.23±1.23	10.3±1.14
立定跳远(m)	167.53±19.75	170.53±20.74	146.36±17.58	158±10.81
坐位体前屈(cm)	6.09±6.9	5.97±6.3	7.81±5.73	10.44±4.97

P<0.05 “ * ”,显著性差异

如表 7 所示，三个月的长跑锻炼对于速度、下肢爆发力、柔韧性没有显著的影响。除了女生的体重存在显著性差异外，P<0.05，其它的身体形态指标也没有统计学差异。但从平均数上看，男生的 BMI 基本没有变化，女生的体脂有轻微下降，其他各个项目都有一定的提高，在身高、体重、50 米、立定跳远和坐位体前屈上男生分别提高了 1.2cm、0.57kg、0.4s、3cm|、0.12cm；女生分别提高了 1.21cm、0.93kg、0.93s、11.64cm、2.63cm。

三个月的长跑锻炼没有使实验组前后的身高体重存在统计学差异。但是在与对照组的前后值进行比较，（见表 4）发现，实验组男女生身高体重提高的比例比对照组男女生的比例要大，对照组男女生身高增长值分别为，1.5cm、1.7cm，体重为，0.87kg、1.28kg；实验组男女生身高增长值分别为，1.7cm、1.8cm，体重为，0.57kg、0.93kg。分析原因为，在 10 岁的生长发育期，身高体重的增长属于自然增长，实验组在自然增长和锻炼因素的双重影响下增长值比对照组更大。说明，三个月的长跑锻炼从性质上来说，对身体形态有一定的积极影响，身高变高，体重变轻，但反应在具体的量化上时（包括锻炼时间、锻炼频率、锻炼强度等），尚未达到足够的刺激因此不能使前后数据具有显著性差异。针对长跑对身体形态方面的促进作用，怎样具体量化这个运动处方，国内外文献在方面的研究不是很多，也没有得出一个相对统一的标准，待做进一步的研究。长跑属于耐力锻炼对速度、下肢爆发力以及柔韧性没有直接的影响。如果没有受到相应运动方面的刺激，在儿童期，运动能力主要受生理年龄、肌肉力量以及身体形态大小的

影响。[12]

3.8 主要测试指标值之间的回归方程及其相关性分析

3.8.1 最大吸氧量绝对值与肺活量的回归方程及相关性分析

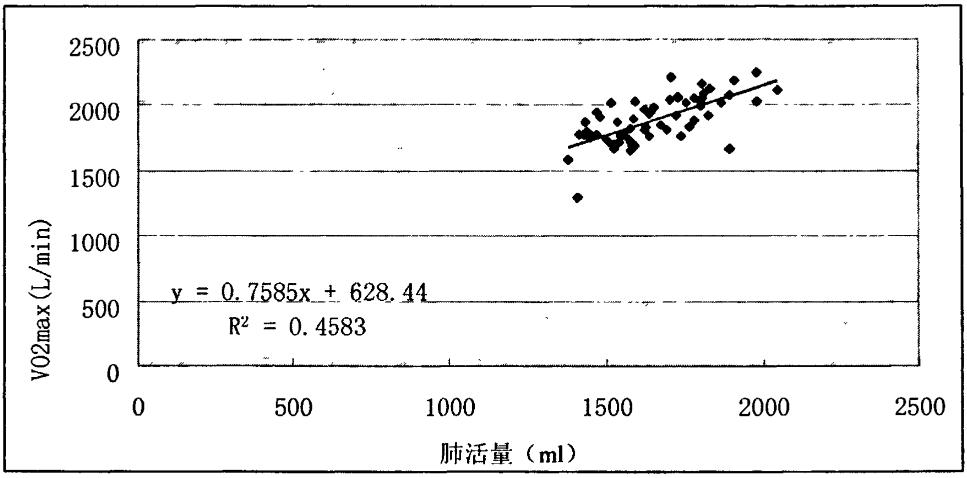

图 1 总体测试男生的最大吸氧量绝对值与肺活量的相关系分析

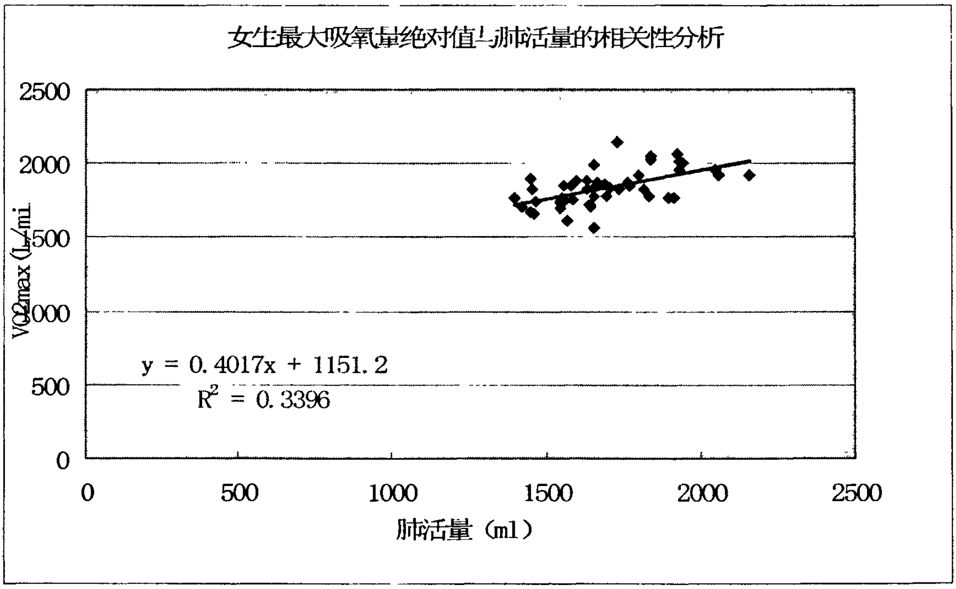

图 2 总体测试女生的最大吸氧量绝对值与肺活量的相关系分析

回归方程和相关系数 R 如下：

男生　Y=0.7585x+628.44　R=0.677

[12] 邓树勋，王健主编，高级运动生理学——理论与应用[M]，北京，高等教育出版社，2003

女生　Y=0.4017x+1151.2　R=0.583

X 为最大吸氧量绝对值(L/min) Y 为肺活量（ml）,男生的最大吸氧量绝对值和肺活量成高度相关，女生的最大吸氧量绝对值和肺活量成中度相关，男女都具有非常显著性差异，（P<0.01）。

最大吸氧量是反映人体在极量负荷时心肺功能水平的一个重要指标，主要由循环系统的功能来决定，它表示氧运转能力的总和，它由最大心输出量和最大氧吸取量来确定。因此，运动时的最大吸氧量首先反映出心输出量水平和心脏储备能力，而静息状态下的心输出量，肺毛细血管楔嵌压、射血分数、心脏大小都不可能预计出心脏的储备状态。其次，机体在运动状态下，心肺的运动是一个整体，是不可分割的，所以最大吸氧量也反映了肺的储备能力。

我们通常认为最大吸氧量与肺活量之间是正相关的,肺活量大的说明其肺功能好,因而最大吸氧量一定会大。其实不然，最大吸氧量是检测有氧活动能力好坏的指标，肺活量是评价肺功能的重要指标，也是影响运动耐力素质的重要指标，反映人体健康状况。肺活量大小可受年龄、身高、体重、体育锻炼水平、胸廓和肺弹性影响。肺活量大说明，被测者的心肺功能好，而肺活量的增大，与被测者是否经常进行各种运动有关。

3.8.2 最大吸氧量相对值与胸围的回归方程及相关性分析

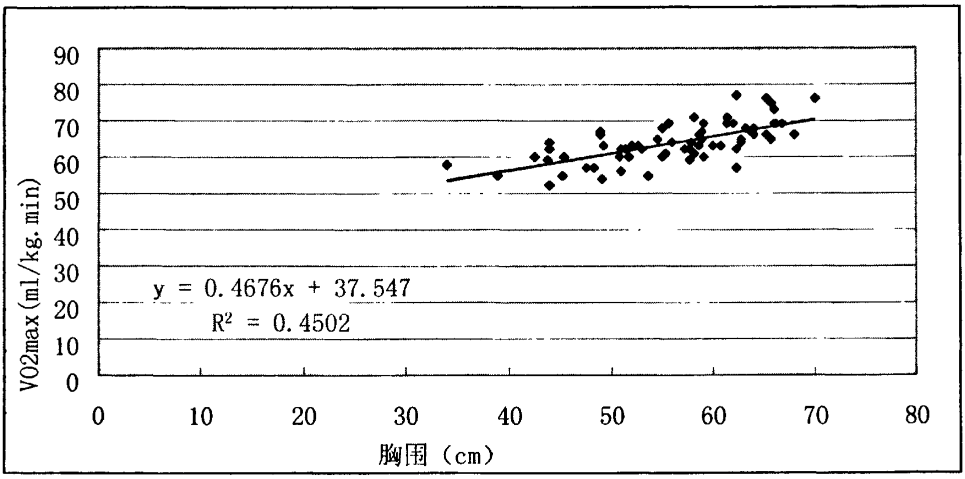

图 3 男生最大吸氧量相对值与胸围的相关性分析

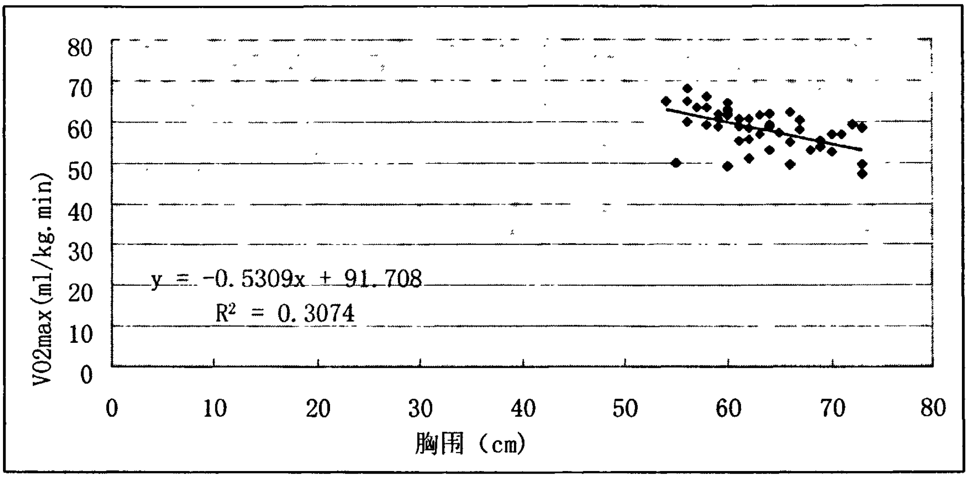

图 5 女生最大吸氧量相对值与胸围的相关性分析

通过计算机统计软件计算，我们得到男女生的最大吸氧量相对值与胸围之间的回归方程和相关系数 R，如下：

男生 Y=0.4676X+37.547 R=0.67

女生 Y=-0.5309X+91.708 R=0.554

X 为最大吸氧量相对值(ml/kg.min)，Y 为胸围(cm),男生最大吸氧量相对值与胸围成正相关，结果具有非常显著性差异，P<0.01；女生的最大吸氧量相对值与胸围成负相关，且结果具有非常显著性差异，P<0.01。但是就男女生两方面来说，两者的相关性一方为正相关，一方为负相关，不具有可比性，可以初步推测女生的最大吸氧量相对值与胸围没有统计学差异。

最大吸氧量相对值是最大吸氧量绝对值除以体重得出的，因此相对值更能反映个体的有氧工作能力情况。最大吸氧量绝对值与肺活量的相关性分析，上文已得出两者具有非常显著性差异的结论。大量的研究表明肺活量与胸围具有高度相关性，从一定程度上说胸围大肺的容积就大，肺活量相对也大。本文的研究得出男生最大吸氧量相对值与胸围成正比而女生的则反比，分析原因可能为，10 岁女生身体有了一定的发育，胸围的增大是因为胸部发育的关系，而不是因为肺容积的变大引起的。

4 讨论

4.1 身体形态

身高体重指数（BMI）

BMI 是一个广泛应用的评价体重是否合理的指标。其计算方法是用以千克为单位的体重值除以米为单位的身高值的平方。BMI=体重（kg）/身高^2（m^2）。BMI 是一个参照个体的身高来评价其体重是否合理的简便易行的指标。

4.2 运动能力

力量素质是指人体身体神经肌肉系统在工作时克服或对抗阻力的能力。快速爆发力属于力量素质的一种。速度素质是指人体快速运动的能力。速度素质包括反应速度、动作速度和位移速度。柔韧素质是指人体关节在不同方向上的运动能力以及肌肉、韧带等软组织的伸展能力。

Brook 和 Fahey 的研究表明，肌肉的发展程度和能力范围也相对依赖于神经系统的成熟程度。10 岁的儿童神经系统发育还未成熟，力量、能力和技能不可能达到最高水平。青少年儿童的爆发力的快速增长突增期，男孩在 10-14 岁，女孩在 8-12 岁。速度素质的快速增长突增期，男孩有两个阶段，7-9 岁和 12-15 岁，女孩只有一个阶段，7-11 岁。柔韧素质的高峰期发展出现 7-12 岁，之后 13-16 由于受身高体重快速增长的影响，柔韧性下降[13]！

4.3 有氧工作能力

反映人体有氧工作能力的生理学指标包括最大摄氧量、最大氧利用能力和有氧运动的效率，而与其相对应的运动生理学检测项目分别是最大吸氧量、通气阈和乳酸阈、有氧运动效率试验。其中最大吸氧量是其中的重要影响指标。肺活量是指在最大吸气后尽力所能呼出的最大气量。它是潮气量、补吸气量和补呼气量三者之和，反映了一次通气的最大能力，是最常用的测定肺通气机能指标之一。肺活量有较大的个体差异，与年龄、性别、体表面体位、呼吸肌力量强弱等有关。体育锻炼可以使呼吸肌发达、胸围增大、肺和胸廓弹性增强，肺活量加大。

4.3.1 最大吸氧量及其测定

最大吸氧量（Maximal oxygen intake,VO2max），又称最大有氧功率（Maximal aerobic power），是指人体在进行大量肌肉参加的长时间激烈运动中，心肺功能

[13] 邓树勋，王健主编，高级运动生理学——理论与应用[M]，北京，高等教育出版社，2003

和肌肉利用氧的能力达到本人极限水平时，单位时间(通常以分钟为单位)内所能摄取的氧量。最大吸氧量的表示方法有两种，即绝对值和相对值。绝对值用 L/min 表示，即整个机体在单位时间内（每分钟）所能吸收的氧量。由于需氧量与体重成正比关系，而身高、体重存在个体差异，因此，用绝对值进行个体间的横向比较是不合适的，而常用人体最大吸氧量的相对值（ml/kg/min）来进行不同个体间最大吸氧量的比较。由于人体的氧运输系统不能够大量存氧，所以通常情况下最大吸氧量等同于最大耗氧量（Maximal oxygen consumption），并均以 VO2max 表示。

最大吸氧量反映人体心肺功能对氧的转运能力和肌肉对氧的利用能力，是评定机体在极限负荷运动时心肺功能水平的一个重要指标，也是评定有氧代谢能力的重要依据。人体 VO2max 的大小与年龄、性别、身体健康状况及运动锻炼水平等因素，进行人体 VO2max 的评价必须兼顾以上因素的作用。

VO2max 可以通过极量运动试验直接测定，也可以根据亚极量运动时的心率、负荷量等参数间接测定。直接测定分为两种，心血管测定法和呼吸测定法，因为要达到极量运动，不管是哪种方法对身体都有一定的伤害，对青少年儿童进行测量时伤害更大。VO2max 的间接测定方法很多，包括最大负荷推算法和亚最大负荷推算法，其基本原理是 VO2 在一定范围内与受试者的负荷功率或 HR 反应之间呈线性相关，因此只要准确测量受试者的做功量或 HR 便可对 VO2max 作出准确的预测。

4.3.2 PWC170 台阶试验推算最大吸氧量

4.3.2.1 前人对 PWC170 台阶试验推算最大吸氧量的研究情况

有研究表明[14]，将用 PWC170 推算出的最大吸氧来量与实际测得的最大吸氧量以绝对值表示时，两者相关较强(相关系数分别为 0.71 和 0.70，前者为女性的）。但当测量结果以每公斤体重(即相对数值)来表示时，VO2max 和 PWC170 的相关强度大大下降(女性和男性少年的相关系数分别为 0.65 和 48)。这说明用 PWC170 推测最大吸氧来量以绝对值表示时存在一定的误差。但同时有文献资料指出用 PWC170 推算 VO2max 的方法在体育调查研究中一直被用作评定少年儿童有氧工作能力的指标，有研究指出在对象为 10~11 岁的儿童时，PWC170 是评价有氧

[14] 李见刚 PWC170 对评价少年儿童有氧工作能力的有效性研究 山东体育科技 1996 年第 3 期

工作能力的一种有效方法。

4.4 锻炼对最大吸氧量的影响

4.4.1 儿童呼吸系统的特点

儿童少年胸廓狭小，气道狭窄，呼吸时的弹性阻力和气道阻力都大，呼吸肌力量较弱，每次呼吸的深度不及成年人，所以肺活量小。但是儿童少年代谢旺盛，对氧的需要相对较多，因而呼吸频率快。在进行剧烈运动时，由于儿童少年的氧运动系统的功能不及成年人，因此其最大通气量和西大吸氧量的绝对值比成年人低，但其相对值却不比成年人低，甚至还略高于成年人。肺的通气与换气机能是影响人体吸氧能力的因素之一，通过科学的运动锻炼，非容积、肺活量增大，儿童少年的心肌收缩力量增强，每搏输出量增加，心力储备提高，呼吸功能增强，氧转运能力达到较高水平，表现为最大吸氧量增加。耐力锻炼不仅改善了氧运输系统的功能，而且也提高了肌肉利用氧的能力，如明显促进儿童少年线粒体内氧有氧代谢合成 ATP。科学的耐力锻炼配合高糖膳食，能有效促进糖原储备，提高运动能力。

4.4.2 锻炼因素对最大吸氧量的影响机理

最大吸氧量的影响因素包括遗传因素、年龄、从性别以及锻炼的影响，能够通过干预改善的只有锻炼，但是对于锻炼能否提高受试者的最大吸氧量一直是一个有争议的话题，而对于青春期发育前儿童的争议就更大了：某些作者报道经过一段有氧锻炼后,VO2max 可得到增加[15]；而另外一些作者则认为这类锻炼在青春期发育高峰之前是不会有任何显著效果的[16]。但是更多试验证明锻炼是可以提高最大吸氧来量的。Mirwald，提出青春期是机能发育的关键时期。在身高生长速度达峰值「PHV）前 1 年及以后，锻炼引起 V02max 的增加高于因年龄和青春期发育突增正常的增长量[17]。许多学者认为练对青春期 VO2max 有积极影响。王淑

[15] 季成叶林碗生张玉青，等.10 一 19 岁男性青少年最大有氧活动能力的间接测定.中国运动医学杂志
SuttonJR.Limitationstomaximaloxygenuptake.SportsMedielne.1992:13(2):]27 一 133
KlissourasV.HeritabilityofadaPtivevariation.JAPplPhysiol.1971:3!:338 一 344

[16] RutenfranzJ，MaeekM，Ander，enKL，etal.Therelationshipbetweenehangingbodyheightandgrowthrelat-edehangesinmaximalaerobiepower.E，irJApl)1Phvsiol.1990;60:282 一 287
5KemperHCG，VersehuurR.IJongitudinalstudyofma 九 malaerobiepowerinteenagers.AnnHumBiol.1987; 14(5):435 一 444
KrahenbuhlGS，MorganDw，PangroziRP.Longitudinaleha esindistanee 一 runningpe，formaneeofyoung males.IntJSportsMed.1989;10(2):92 一 96

[17] SjodinB，SvedenhagJ.ygenuptakeduringrunningasrelatedtobodymassineireumpubertalboys:alongi-tudinal，tudy.EurJApPIPhysiol.1992;65(2):150 一 157
IngjerF.Developmentofmaximaloxygenuptakeinyoungelitemaloeross 一 eountryskiers:alongitudinal

云等对 14-16 岁学生的研究表明，中长跑锻炼对提高机体的有氧代谢水平效果最好。锻炼对最大吸氧量提高的程度取决于锻炼强度、锻炼频率、每次锻炼持续的时间、锻炼期限以及个体初始的活动能力水平[18]，锻炼强度似乎是导致有氧活动能力提高的关键因素。最大吸氧量主要决定于心脏的泵血功能和肌肉利用氧的能力。故将心脏的泵血功能称为最大吸氧量的中央机制，而把肌肉利用氧的能力称为最大吸氧量的外周机制。影响最大吸氧量的主要机制是心脏的泵血功能。此功能的大小又取决于心脏容积和心肌收缩力。锻炼提高最大吸氧量的原因，是由于锻炼可增大心容积和心肌收缩力量，研究表明，一般人的心容积为 700—800，而耐力运动员可达 900—1000ml ，此外，锻炼可导致慢肌纤维线粒体增大、增多，线粒体氧化酶的活性增加，提高氧的摄取。同时，耐力锻炼在一定范围内可以导致快肌纤维的生理、生化代谢特征向慢肌纤维方向变化，提高摄氧和利用氧的能力。[19]

本文中的最大吸氧量绝对值是由PWC170推测出的，两者成正相关，PWC170的提高直接导致了最大吸氧量绝对值的提高。对于锻炼能否提高青少年儿童的最大吸氧量绝对值一直存在争议。SPrynarova[20]认为青春期锻炼组与非锻炼组V02max 的差别可能与青春期启动前的锻炼影响有关。无论生长速度峰值还是达生长速度峰值的年龄都不受锻炼的影响，而最大吸氧量、最大氧脉搏增长速度达峰值的年龄大致与机体测量指标相同。因此认为在青春期不同运动形式对心肺功能发育只存在潜在的影响[21]。在身高生长速度达峰值「PHV）前 1 年及以后，锻炼引起 V02max 的增加高于因年龄和青春期发育突增正常的增长量。许多学者认为锻炼对青春期 VO2max 有积极影响。

锻炼对儿童青少年最大有氧活动能力的影响程度取决于锻炼强度、锻炼频

study.JSPortsSei.1992;10(l):49 — 63

Mahonad，VaeearoP.VentilatorythresholdandVOZmaxehanges — nehildre[if(、llow — ngenduraneetral;ling. MedSeiSP()rtsExere.1989;21(4):425 — 431

MassieotteL)R，MaenabRBJ piratoryadaptationstotr::intngatspeeifiedintensitiesinehildrel:.Med SeiSP()rrs.19:6(4):242 — 246

18 王淑云等 运动训练对青少年最大吸氧量的影响 国家体委体育科学研究所 中国体育科技 1985 年第 16 期

19 邓树勋，王健，乔德才主编. 运动生理学[M]，北京，高等教育出版社，2005

邓树勋，王健主编，高级运动生理学——理论与应用[M]，北京，高等教育出版社，2003

20 Sprynarova5.1’ l 飞 einflueneeoftrainingonphy、iealandfunetionalgrowthbofore，duringandafterpuberty. E、— rJApplPhysiol.1987;56:719 — 724

21 wengerHA，BellGJ.Thelnteraetionsofintensity，frequeneyallddurati oliofexereisetraininginalteringear-diorespiratoryfitness.SportsMedieine.1986;3:346 — 456

率、每次锻炼持续的时间、锻炼期限以及个体初始的活动能力水平，锻炼强度似乎是导致有氧活动能力提高的关键因素。每周 2 次的锻炼频率对较低能力水平的有氧活动能力有明显改进，而增加到每周 4 次将产生最大效益。每次锻炼超过 35 分钟将对 VO2max 有最大影响.较低强度而较长时间的锻炼等同或大于较大强度而较短时间的锻炼效果。锻炼影响的程度还取决于个体初始能力水平，一般是按能力水平提高的程度有比例增加。建议运动强度达 V02max 的 90 一 100%，频率为每周 4 次，每次运动时间是 35 一 45 分钟的锻炼计划可获得 VO2max 的最大提高，但是这种强度对于没有经过系统科学锻炼的人有一定的危险，研究发现低强度的锻炼也能产生有效的变化，并可减少非运动员人群运动创伤的危险。

4.4.2.1 有氧耐力锻炼对儿童的影响

有氧耐力（aerobic capacity）是指人体长时间进行有氧工作（依靠糖、脂肪等有氧氧化功能）的能力。科学系统的有氧耐力锻炼能使心脏的形态与机能出现一系列适应性变化。主要表现为左心室心腔扩张，心容积增大，安静时心率减慢。美搏输出量增加。其表明心脏的泵血机能和工作效率得到提高，以适应长时间持续运动的需要。心输出量是决定最大摄氧量的中枢机制，而肌肉纤维类型的百分比组成及其本身的特点是决定最大吸氧量的外周机制。大量的研究证明，科学系统的有氧耐力锻炼能增加心输出量并使肌纤维类型及其本身特点向有利于有氧耐力的方向转换。系统的耐力锻炼，可以提高机体动用脂肪供能的能力。在长时间耐力锻炼练习中，随着运动时间的延长，脂肪供能的比例逐渐增大，从而节省糖原的利用。从形态上观察，经过科学系统锻炼的长跑耐力运动员，都是身材偏瘦的，但是由于瘦体重的增加，体重并不一定比选练前有很大的下降。

5 结论

1．对测试学生与全国学生的各项指标进行差异性分析，除了身高，各项都存在差异，部分存在非常显著性差异。

2．将测试对象的最大吸氧量值在全国标准中进行百分比占有率的划分，结果表明卡尔普曼公式推算儿童的最大吸氧量是可行的，并且反应出测试对象的最大吸氧量水平中等偏下，中等所占比例最大，以最大吸氧来量相对值计算比绝对值的成绩好。

3．对锻炼前后对照组的各项指标进行差异性分析表明：男女的身高体重都有一

定的增长，男生的身高体重存在显著性差异，分析原因，身高体重的增长是人体自然增长的结果。其他各项有上下波动，但都不存在统计学差异。

4. 对锻炼前后实验组与对照组的各项指标进行差异性分析表明：男女身高体重都不存在统计学差异，但是实验组的增长比例比对照组大，分析原因为，试验组对象受到自然增长和运动因素的双重刺激；男女在立定跳远、肺活量、PWC170上存在显著性差异，男生在最大吸氧量绝对值上存在显著性差异，女生在最大吸氧量相对值上存在显著性差异。说明，锻炼达到了提高人体有氧工作能力的效果，男女之间有差异。

5. 对锻炼前后实验组有氧工作能力相关指标的差异性分析，男女生在PWC170和最大吸氧量绝对值上都存在显著性差异，男生还在肺活量上存在显著性差异。对身体形态和运动素质指标的差异性分析，除了女生的体重存在显著性差异，其他各项均没有统计学差异。但是在各项的增长比例上均大于锻炼后的对照组的数值，说明在一定程度上，锻炼还是对身体形态和运动素质起到积极的作用。

6. 最大吸氧量绝对值与肺活量的回归方程与相关性系数R如下：

男生　$Y=0.7585x+628.44$　$R=0.677$

女生　$Y=0.4017x+1151.2$　$R=0.583$

男生的最大吸氧量绝对值和肺活量成高度相关，女生的最大吸氧量绝对值和肺活量成中度相关，男女都具有非常显著性差异，$P<0.01$。

最大吸氧量相对值与胸围之间的回归方程和相关系数R，如下：

男生　$Y=0.4676X+37.547$　$R=0.67$

女生　$Y=-0.5309X+91.708$　$R=0.554$

X为最大吸氧量相对值(ml/kg.min)，Y为胸围(cm),男生最大吸氧量相对值与胸围成正相关，结果具有非常显著性差异，$P<0.01$；女生的最大吸氧量相对值与胸围成负相关，且结果具有非常显著性差异，$P<0.01$。

7. 对小学生进行的耐力锻炼，研究表明三个月可以作为一种阶段性的措施，在一定程度上提高了小学生的有氧工作能力，对于身体形态和运动素质也有一定的正面影响。

6 致谢

本文是在我的导师卢昌亚教授的悉心指导和严格要求下独立完成的。在整

个课题研究的过程中，从课题的选择到论文的撰写、观点的形成等都得益于导师的学术思想。导师严谨的治学态度，精益求精的学术追求、一丝不苟的科学态度以及孜孜不倦的求学精神，给我留下了深刻印象。在老师的帮助下我的毕业论文顺利完成，更重要的是在整个过程中我学到了一篇论文从形成创作思路到写作完成的一系列的方法和技巧。在这三年的学习过程中，导师对我的影响深远而广泛，并将使我终身受益。在此，我表示衷心的感谢。

此外，我还要感谢周李民、潘国建和梁晓刚教授，他们给我的论文提出宝贵的建议。在进行论文试验部分时，也离不开温州市瑞安市安阳实验小学的四（2）班、四（6）班的全体学生和体育组全体体育教师的帮助及指导，没有他们的全力支持我也不能如此顺利的采集到论文数据，再次感谢他们。我还要感谢三年来的同窗好友，师弟师妹、上海师范大学学校和各学院的各位领导、老师们，他们在我三年的求学中不断给予鼓励与帮助，生活上无微不至的关怀与照顾。特别指出的是戴恩民同学，感谢他提供给我重要的数据资料以及在数据处理上的指导。

感谢我的家人和朋友，正是他们的辛勤劳动和大力支持才使我能安心学习，顺利完成学业，最后还要感谢课题中引用的论文和专著作者们，没有他们的前期研究工作，就难以有这篇文章的完成。

7 参考文献

[1] 邓树勋，王健，乔德才主编. 运动生理学[M]，北京，高等教育出版社，2005

[2] 邓树勋，王健主编，高级运动生理学——理论与应用[M]，北京，高等教育出版社，2003

[3] 姚泰主编，生理学（第六版）[M]，北京，人民卫生出版社，2004

[4] 杨锡让，傅浩坚主编，运动生理学进展[M]，北京体育大学出版社，1999

[5] 赵家琪等，实用运动生理学问答[M]，北京，人民体育出版社，1993

[6] 卢昌亚，李洁，龙之友主编，运动生理学[M]，广西师范大学出版社，2008 年 1 月

[7] 卢昌亚，曹可强主编，大学体育[M]，上海，华东理工大学出版社，2004 年 6 月

[8] 李洁，陈仁伟主编，人体运动能力检测与评定[M]，北京，人民体育出版社，2005

[9] 赵秋蓉等编著，体育测量评价[M]，陕西人民教育出版社，1992

[10] 王健主编，运动生理学研究技术[M]，浙江大学出版社，2001 年 4 月

[11] 肖国强，运动与能量代谢[M]，北京，人民体育出版社，1998

[12] 杨培禾主编，小学生生理卫生[M]，北京，科学出版社，2001 年 8 月

[13] 毛振明，杜俊娟主编，小学生体育考试与达标[M]，北京，人民体育出版社，1997

[14] 卢昌亚等，上海与美国南加州部分中学体育课健身指标变量值的比较研究[J]，体育科学 2004（24）1：37~37

[15] 卢昌亚，美国中小学体育教学的健身目标[J]，体育教学，2001（78）3：42~43

[16] 卢昌亚，论学校体育健身目标的量化与阶段性特点[J]，体育学刊 1996（7）3：13~15

[17] 卢昌亚，体育课健身目标的变量分析[J]，体育学刊 1997（10）2：19~21

[18] 关明杰等，10~18 岁儿童青少年最大吸氧量正常值[J]，体育科学，1995（15）5：47~51

[19] 蔡莹，对少年吸氧量变化规律的实验研究[J]，体育学刊，1998，3：43~45

[20] 李新宇等，由台阶试验推算儿少最大吸氧量的分段追踪观察[J]，北京体育大学学报 2001（21）1：54~55

[21] 龙海燕，王步标，10~12 岁男孩最大吸氧量间接测定法[J]，湖南大学学报 1992 年 1 期

[22] 颜凯，邓树勋，影响儿童青少年最大有氧活动能力的因素[J]，体育学刊，2001 年 5 期

[23] 杨茂林，影响儿童少年有氧活动能力发展的因素[J]，内蒙古体育科技，2007 年 4 期

[24] 林琬生等，儿童最大有氧活动能力的追踪研究[J]，体育科学，1997 年 2 期

[25] 王步标等，9~18 岁学生最大吸氧量的纵向研究[J]，体育学刊，1995 年 2 期

[26] 林琬生等，儿童最大有氧活动能力的发展特征[J]，人类学学报 1993：12（4）：383~389

[27] 中华人民共和国教育部 2005 年全国学生体质与健康调研结果公告 vol.4.no.1 2007

[28] 邱少霖等 PWC170 台阶实验、15min 跑和 12min 跑与间接推算 VO2max 的研究 北京体育大学学报 2001 年 3 月 第 24 卷第 1 期

[29] 刑文华等 体育测量与评价 北京体育学院出版社 1986 年 1 月

[30] 2005 年全国学生体质与健康调研报告 高等教育出版社。2005.12

[31] 李见刚 PWC170 对评价少年儿童有氧工作能力的有效性研究 山东体育科技 1996 年第 3 期

[32] 王淑云等 运动锻炼对青少年最大吸氧量的影响 国家体委体育科学研究所 中国体育科技 1985 年第 16 期

[33] Antonio S. Santo and Lawrence A. Golding. Predicting Maximum Oxygen Uptake From a Modified 3-Minute Step Test. Res. Quart. For Exercise and Sport.2003(74)1:110~115

[34] Timothy J. Bungum et al. One-Mile Run Performance and Body Mass Index in Asian and Pacific Youth: Passing Rates for the FITNESSGRAM. Res. Quart. For Exercise and Sport.1998(69)1:89~93

[35] James D. George. Alternative Approach to Maximal Exercise Testing and VO_2max Prediction in College Students. Res. Quart. For Exercise and Sport.1996(67)4:425~457

[36] Pat Vehrs, James D. George. Prediction of VO_2max Before, During, and After 16 weeks of Endurance Training. Res. Quart. For Exercise and Sport.1998(69)3:297~303

[37] Kemper H. C. et al. Longitudinal Study of Maximal Aerobic Power in Teenagers. Ann-Hum Biol. 1987(14)5:435~444

[38] Getchel K.L, et al. Prediction of Maximal Oxygen Uptake in Young Women Joggers. Res. Quart. For Exercise and Sport.1977(48):61~67

[39] George J. D, et al. VO_2max Estimation from a Submaximal 1-Mile Track Jog for Fit College-aged individuals. Med and Sci in Sports and Exercise. 1994(25):401~406

[40] Kline G. M, et al. Estimation of VO_2max from 1-Mile Track Walk, Gender, Age and Body Weight. Med and Sci in Sports and Exercise. 1978(19):253~259

[41] [1] Baxter J et al. The development of aerobic power inyoung athletes. J Appl Physiol 1993,75:1160-1167.

[42] Kobayashi K et al. Aerobic power as related to body growth and training in Japanese boys: a longitudinal • 85·study. J Appl Physiol 1978,44:666-672.

[43] Mahon AD,Vaccaro P. Cardiovascular adaptations in 8 to 12 years old boys following a 14 week running program.Can J Appl Physiol 1994,19:139-150.

[44] Kemper HCG,Van de Kop H.Entrainement de puissance maximale aerobie chez les enfants prepuberes et puberes.Science & Sport 1994,10:29-38.

[45] Baquet G et al. Effects of high intensity intermittent training on peak VO(2) in prepubertal children.Int J Sports Med 2002,23:439-444.

[46] Lewis DA et al. Physiological differences between gen-ders. Implications for sports conditioning. Sports Med 1986,3:357-369.

[47] Rowland WT. Aerobic responses to endurance training in prepubescent children: a critical analysis. Med Sci Sports Exerc 1985,17:493-507.

[48] SuttonJR. Limitationstomaximaloxygenuptake.SportsMedielne.1992:13(2):]27 — 133

[49] KlissourasV.HeritabilityofadaPtivevariation.JAPplPhysiol.1971:3!:338 — 344

[50] RutenfranzJ, MaeekM, Andeetal.Therelationshipbetweenehangingbodyheightandgrowthrelatedehangesinmaximalaerobiepower.E (Apl)1Phvsiol.1990;60:282 — 287

[51] KemperHCG, VersehuurR.IJongitudinalstudyofmamalaerobiepowerinteenagers.AnnHumBiol.1987; 14(5):435 — 444

[52] KrahenbuhlGS, MorganDw, PangroziRP.Longitudinaleha esindistanee runningpe, formaneeof

young males.IntJSportsMed.1989;10(2):92 — 96

[53]SjodinBSvedenhagJ.ygenuptake during running asrelatedto body mass ineireum pubertal boys:alongi-tudinal，tudy.EurJApPIPhysiol.1992;65(2):150 — 157

[54] IngjerF.Development of maximal oxygen uptaken younge litemalo erosseoun tryskiers:along tudinal study.JSPortsSei.1992;10(1):49 — 63

[55] Mahonad，VaeearoP.VentilatorythresholdandVO2max ehanges — nehildre ngendu raneetra ling. MedSeiSP rtsExere.1989;21(4):425 — 431

[56] MassieotteL)R，MaenabRBJ. Pirator yadap tationstotr:: intngat speeified inten sities inehildrel Med SeiSP()rrs.197:6(4):242 — 246

[57] Sprynarova einflueneeo ftrainingonphy、iealandfune tiona lgrowthbofore，during andaf terpuberty. E — rJApplPhysiol.1987;56:719 — 724

[58] wengerHA，BellGJ.TheInteraetionsofintensity frequene yallddura tiolio fexerei setrainin ginalter ingear-diorespiratory fitness.SportsMedieine.1986;3:346 — 456

万方数据 WANFANG DATA 文献链接

三个月耐力锻炼对小学四年级学生有氧能力为主的体质指标的影响

作者：　金海娜
学位授予单位：　上海师范大学
被引用次数：　1次

本文读者也读过(10条)

1. 李仕丰.张玉珍.李粲.李慧德 1995-2005年福建省中小学生耐力素质的动态分析[期刊论文]-福建体育科技 2006,25(4)
2. 陈猛醒.CHEN Mengxing 黎族小学生耐力素质的发展特点与提高策略[期刊论文]-海南师范大学学报（自然科学版）2009,22(1)
3. 孙传刚 浅析如何培养小学生的跑步兴趣[期刊论文]-中国科技博览2010(16)
4. 贺松磊 做好小学生思想品德工作的重要途径[期刊论文]-决策探索2007(22)
5. 万琼 武汉市中小学生耐力素质变化趋势分析[期刊论文]-中国学校卫生2007,28(2)
6. 刘青.刘加昌.姜利华.Liu Qing.Liu Jiachang.Jiang Lihua 青春期男女骨密度与性激素水平测定[期刊论文]-感染、炎症、修复2005,6(1)
7. 黄建春.HUANG Jian-chun 五个不同蘑菇品种出菇试验简报[期刊论文]-中国食用菌2000,19(4)
8. 游家水.You Jiashui 小学生自主意识和主动发展能力的培养模式[期刊论文]-教育导刊2009(2)
9. 黄娟 引导学生写出真实作文[期刊论文]-读与写（教育教学刊）2007,4(1)
10. 易洪文 浅谈小学生守则规范的贯彻实施[期刊论文]-科学咨询2010(3)

引证文献(1条)

1.邱小梅.陈慧.王珍妮.蒋龙 登山锻炼对小学三年级学生身体素质影响的研究[期刊论文]-山东体育科技 2012(2)

引用本文格式：金海娜 三个月耐力锻炼对小学四年级学生有氧能力为主的体质指标的影响[学位论文]硕士 2009
